# Phylogenetic Aspects of Antibiotic Resistance and Biofilm Formation of *P. aeruginosa* Isolated from Clinical Samples

**DOI:** 10.1155/2024/6213873

**Published:** 2024-01-13

**Authors:** Maryam Motevasel, Masoud Haghkhah, Negar Azimzadeh

**Affiliations:** ^1^Department of Pathobiology, School of Veterinary Medicine, Shiraz University, Shiraz, Iran; ^2^Department of Laboratory Sciences, School of Paramedical Sciences, University of Medical Sciences, Shiraz, Iran

## Abstract

**Introduction:**

Biofilm production and drug resistance phenomenon play a critical role in *P. aeruginosa* infections. Several genes, including *psl*, *pel*, *brlR*, and *mex*, are involved in the phenomenon. The aim of this study was to find the relationship between the mentioned genes and the sources of *P. aeruginosa* infections.

**Materials and Methods:**

Fifty-nine *P. aeruginosa* isolates detected from clinical specimens were used to determine antibiotic susceptibility patterns, prevalence of the genes using PCR, biofilm formation, biofilm eradication concentration assay (MBEC), and epidemiological characteristics using pulsed-field gel electrophoresis (PFGE).

**Results:**

The results showed that 35.6% and 16.94% of all the samples were isolated from urine and wounds, 81.33% of the isolates were biofilm producers, 27.11% were multidrug-resistant (MDR), and 100% of the main biofilm former genes belonged to *pslA*. 94.91% of the isolates possessed *brlR* and *mexA*, and 91.5% of them expressed *pslA*. It was also indicated that neither ciprofloxacin nor imipenem could eradicate the formed biofilms. Moreover, we could identify 81.4% distinctive restriction profiles among the isolates, using an 80% similarity cutoff point; *brlR* and *pel* genes were significantly (*P*=0.032; *P*=0.044) related to phylogenetic pulsotypes. Comparison of the dendrogram in the isolates revealed that the detected isolates from urine were present in 12 different pulsotypes.

**Conclusion:**

It was found that there was a relationship between MDR, biofilm production, and *brlR* and *pel genes* among the isolates. It is distinguished there were similar genetic patterns between detected isolates from urine and could be concluded that the urinary tract played a critical role in maintaining and transferring biofilm drug-resistant genes of *P. aeruginosa* in clinical sites. The study highlights the importance of urine in distribution of clinical biofilm formation and drug-resistant *P. aeruginosa* isolates.

## 1. Introduction

*P. aeruginosa* is a nosocomial MDR pathogen that poses a serious threat to public health [1, 2]. Biofilm-associated infections caused by *P. aeruginosa* are particularly challenging, as bacteria embedded in biofilms are more resistant to antibiotic treatments. The biofilm matrix of *P. aeruginosa* is composed of proteins, DNA, lipids, alginate, and essential exopolysaccharides such as *pel* and *psl*. Ma et al. demonstrated that the *psl* gene plays a critical role in adherence of cell surfaces [3]. Biofilm production by *P. aeruginosa* facilitates its persistence in the environment and can lead to severe bacterial infections, especially in hospitalized patients [4]. However, in vitro biofilm production among clinical strains varies, and *P. aeruginosa* can be classified as strong, moderate, weak, or nonbiofilm producers [5, 6].

Biofilm production and MDR *P. aeruginosa* infections are particularly problematic in immunocompromised individuals with respiratory, urinary tract, or chronic wound infections [7, 8]. The *brlR* gene contributes to drug tolerance and efflux pump in the formed biofilm of *P. aeruginosa*, while the *mex* operon is responsible for MDR *P. aeruginosa* [9, 10].

To investigate the relationship between drug resistance and biofilm formation in clinically recovered *P. aeruginosa* isolates, there is a need to find epidemiological similarity among the isolates. Molecular epidemiological typing techniques, such as PFGE, can be used for following the “transmission route” and “clonal analysis” of clinical bacterial infections [11].

In this study, the clinically recovered *P. aeruginosa* isolates were examined for susceptibility to common antibiotics, recognition of MDR strains, biofilm formation, the in vitro effect of different antibiotic concentrations on biofilm formation, and the prevalence of five genes: *pslA*, *pelA* and *pelB*, *brlR*, *mexA*, and *mexB*, and finally epidemiological relationship between the isolates.

## 2. Materials and Methods

### 2.1. Bacterial Isolates

A total of 59 *P. aeruginosa* isolates were collected from clinical samples using standard diagnostic bacteriological procedures [12]. The isolates were recovered from infectious sites such as blood, body fluids, sputum, urine, and wounds of hospitalized patients.

### 2.2. Molecular Identification of *P. aeruginosa* Isolates

#### 2.2.1. DNA Extraction

To confirm the molecular identification of the isolates, genomic DNA was extracted from all isolates using a simple boiling method. Briefly, a few bacterial colonies were suspended in 200 *µ*L of molecular grade water and heated for 15 min at 95°C, followed by centrifugation at 14000 × g for 10 min. The supernatant containing DNA was used for PCR amplification.

#### 2.2.2. Molecular Confirmation of the Isolates as *P. aeruginosa*

PCR primers designed in this study were employed to amplify the sequence of the genes of the isolates. Amplification was carried out in a total reaction volume of 25 *μ*L containing 2.5 *μ*L 10x PCR buffer, 1.5 mM MgCl_2_, 0.2 mM deoxynucleotide, 0.4 pmol/*µ*L of each primer (Bioneer, Seoul, South Korea), 0.2 U Taq DNA polymerase, and 3 *µ*L DNA. The thermocycler (Analytik Jena, model: Flex Cycler 96G) was set with the following conditions: initial denaturation for 2 min at 95°C, followed by 25 cycles of denaturation for 20 s at 94°C, annealing for 20 s at 58°C, an extension for 40 s at 72°C, and final extension for 1 min at 72°C. Electrophoresis was performed on a 1.5% agarose gel along with GeneRuler 100 bp DNA Ladder (Fermentas, Lithuania) and stained with 0.5 *μ*g/mL ethidium bromide.

*P. aeruginosa* ATCC 27853 was used as a positive control in all experiments.

### 2.3. Identification of Virulence Genes of *Pseudomonas aeruginosa*

To determine the pathogenic potential of *P. aeruginosa* strains among the isolates, PCR amplification was carried out using specific primers for five virulence genes *pslA*, *pelA* and *pelB*, *brlR*, *mexA*, and *mexB* (Table 1).

Amplification of the DNA fragments was carried out as described above, with the following thermal cycling profile: initial denaturation at 94°C for 5 min, followed by 35 cycles of denaturation at 94°C for 30 s, annealing at 52°C for 40 s, extension at 72°C for 50 s, and final extension for 10 min at 72°C.

### 2.4. Antimicrobial Susceptibility Tests

#### 2.4.1. Disk Diffusion Method

Disk diffusion method [13]: the isolates *P. aeruginosa* ATCC 27853 (as a positive control) and *E. coli* ATCC 25922 (as a negative control) were tested for amikacin (30 *μ*g), ciprofloxacin (5 *μ*g), imipenem (10 *μ*g), gentamicin (10 *μ*g), ceftazidime (30 *μ*g), ceftriaxone (30 *μ*g), and cefepime (30 *μ*g) (HiMedia Laboratories Pvt. Ltd., Mumbai, India). *P. aeruginosa* isolates resistant to more than three separate classes of antibiotics were considered as multidrug-resistant (MDR).

#### 2.4.2. Broth Dilution Method: MIC Determination

The minimum inhibitory concentration (MIC) of ciprofloxacin and imipenem was determined using the microbroth dilution method as described by NCCLS [14]. MIC was defined as the lowest concentration of antibiotic giving complete inhibition of visible growth.

### 2.5. *P. aeruginosa* Isolates' Biofilm

In vitro biofilm formation of the *P. aeruginosa* isolates, wildtype PAO1 and ATCC 27853 were used for culture, biofilm formation, and antibiotic-resistant biofilm experiments. Biofilm formation was assessed using a simple microtiter-plate assay in triplicates, and optical density was measured at 570 nm with an established cutoff value (ODc) for demonstrating strong, moderate, and weak biofilm formation [2, 15].

### 2.6. Biofilm Eradication Concentration Assay (MBEC) of Imipenem and Ciprofloxacin against Formed Biofilm

The minimum biofilm eradication concentration (MBEC) values of ciprofloxacin and imipenem against *P. aeruginosa* biofilms were determined [16]. Briefly, the isolates were grown in 96-well microplates for 24 h at 37°C in the Mueller–Hinton (MH) medium and then washed twice with 0.9% NaCl to remove the planktonic cells. The attached biofilms were then subjected to treatment with different concentrations of ciprofloxacin and imipenem at 2-fold dilutions from 2048 *μ*g/mL to 2 *μ*g/mL for each antibiotic. After incubation for 24 h at 37°C, the microplates were washed with 0.9% NaCl to remove any antimicrobial residue. The biofilm was scraped from the microplate wells and transferred into a sterile tube containing tryptic soy broth (TSB). Five microliters of the suspensions was cultured on the Mueller–Hinton agar plate and then incubated overnight at 37°C. MBEC values were determined to be the lowest antibiotic concentrations that prevented the bacterial growth from the treated biofilm.

### 2.7. Phylogenetic Assay: DNA Genomic Typing by Pulsed-Field Gel Electrophoresis (PFGE)

Using the highly discriminating method and according to the criteria developed by Durmaz et al. [17], briefly, examined “cell suspension preparation, plug preparation, lysis of cells in agarose plugs, restrictive digestion of DNA in agarose plugs, casting of the agarose gel, electrophoresis,” and restriction analysis; then, PFGE (PFGE CHEF-DR III System, Bio-Rad) of DNA was performed, and the band profiles of DNA were analyzed by using BioNumerics software (version 7.0, Applied Maths, Sint-Martens-Latem, Belgium). We analyzed 59 clinical isolates of *P. aeruginosa* recovered from patients, and one *P. aeruginosa* ATCC 27853 strain was used as a control. For molecular analysis of *P. aeruginosa* strain isolates, PFGE was performed according to the mentioned protocol. *Salmonella* serotype Branderup strain (H9812) ladder (Bio-Rad Laboratories) restricted with *XbaI* was used as a universal size marker. *SpeI* (MBI Fermentas, Vilnius, Lithuania) restriction enzyme was used to generate the banding patterns for the *P. aeruginosa* clinical isolates as well as the standard controls. The protocol included cell suspension preparation, plug preparation, lysis of the cells in agarose plugs, restrictive digestion of DNA in agarose plugs, casting of the agarose gel, electrophoresis, and restriction analysis. The band profiles of DNA were analyzed using BioNumerics software (version 7.0, Applied Maths, Sint-Martens-Latem, Belgium), and the developed criteria by Durmaz were followed.

### 2.8. Statistical Analysis

Statistical analysis was performed using the chi-square test through SPSS 16. *P* values <0.05 were considered statistically significant. The results, after then, were compared with PFGE band profiles gained from the similarity of examined specimens.

## 3. Results

### 3.1. Characteristics of *P. aeruginosa* Isolates

The collected isolates were confirmed as *P. aeruginosa* by specific PCR. Of 59 clinical samples, 21 (35.6%) and 10 (16.94%) *P. aeruginosa* isolates were recovered from urine and wound specimens, respectively. The remaining isolates were obtained from other sites of infection (Table 2). The antibiotic susceptibility patterns of *P. aeruginosa* isolates indicated that 16 (27.11%) were multidrug-resistant, with the highest number MDR recovered from the wound (11.76%) and urine (8.48%) samples (Table 2).

### 3.2. The Most Common Combination of Resistance Was Found to be CP, IPM, and CRO

Sixteen isolates showed resistance to three or more antibiotics with 8 (50%) isolates exhibiting resistance to three antibiotics and the other 8 (50%) isolates exhibiting resistance to more than five antibiotics. Overall, 16 isolates were classified as multiresistant as they were resistant to three or four different antibiotic classes.

### 3.3. Prevalence of Virulence Genes among *P. aeruginosa* Isolates

The distribution of five virulence genes, namely, *pslA*, *pelA* and *pelB*, and *brl*R of *P. aeruginosa* biofilms, as well as *mexA* and *mexB*, varied depending on the site of infection (Table 3).

Table 3 shows that the frequencies of virulence gene occurrence in all the isolates were as follows: *mexA*, *brlR*, *pslA*, *pelA* and *pelB*, and *mexB*, respectively.

### 3.4. In Vitro Biofilm Formation and Determination of MBEC

Overall, biofilm formation was observed in 81.33% of the *P. aeruginosa* isolates. Biofilm production was evaluated by the qualitative microplate method, which revealed 30 (50.84%) strong biofilm producers, 14 (23.72%) moderate producers, 4 (6.77%) weak producers, and 11(18.84%) nonbiofilm producers of *P. aeruginosa*. The distribution of biofilm producing *P. aeruginosa* isolates from different clinical samples is shown in Table 4.

When compared with the isolates from other specimens, the majority of the strains producing a strong biofilm belonged to wound infection (70%) and urinary tract infection (52.38%). Furthermore, 100% of wound isolates and 85.71% of urine isolates were biofilm producers (Table 4).

During the EMBEC test, we found that ciprofloxacin was unable to eliminate bacterial biofilms at any concentration ranging from 2 to 2048 *μ*g/mL. While for some strains, a reduction in biofilm formation was observed when using imipenem at a concentration of >512 *µ*g/mL, and it was still unable to eradicate the established biofilm.

### 3.5. Genotyping by PFGE

**Figure 1:**
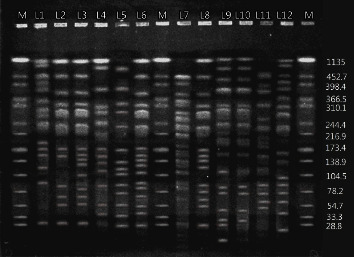
*P. aeruginosa* isolates analyzed by PFGE following *SpeI* digestion. Lanes: (M) molecular mass standard (lambda marker, kbp ladder).

Using this method, we could identify 13 distinctive restriction profiles, each with unique banding patterns among 48 *P. aeruginosa* isolates. This corresponded to a cutoff value of 80% identity for PFGE, and the PFGE profiles typically contained 12–17 bands. The distribution of the number of fragment differences is shown in Figure 1. However, the reaming 11 isolates had their own unique patterns that were not categorized into any of 13 distinctive profiles (Table 5). The *P. aeruginosa* ATCC 27853 control strain was also typed. The distribution of virulence genes among the 13 distinctive clones and 11 single clones is shown in Table 5.

### 3.6. Clonal Analysis

A dendrogram was constructed using BioNumerics software (version 7.0, Applied Maths, Sint-Martens-Latem, Belgium) to compare each of the PFGE banding patterns with all other banding patterns, and it demonstrated the rates of genomic similarity (Figure 1). Using an 80% similarity cutoff point, the analysis of PFGE patterns (pulsotypes) resulted in 13 pulsotypes and 10 unique singleton PFGE types.

## 4. Discussion

Drug-resistant pathogenic bacteria have created numerous problems in hospitals and patient care centers. Not only can opportunistic pathogenic bacteria, such as *P. aeruginosa,* can be drug-resistant and infective but also drug-resistant environmental *Pseudomonas* sp. can infect many accidental or traumatic patients [18]. Meng et al. expressed concern about antibiotic resistance in *Pseudomonas* spp. detected from “bulk-tank milk.” Surprisingly, they found that the bacteria were highly resistant to several important clinical antibiotics, such as imipenem (95.3%) and aztreonam (60.5%). Their mainly intrinsic resistance virulence and drug efflux pump genes in the bacteria [19] may pose a significant threat to healthcare units and hospitals that admit susceptible patients.

Currently, *P. aeruginosa* is the most significant challenge in the treatment of hospitalized *Pseudomonas* infections. Moreover, a relationship is believed to exist between drug resistance and the biofilm formation phenomenon in the *P. aeruginosa* isolates. There are many genes in the bacterium that play a role in drug resistance and/or biofilm formation. In the present study, we investigated several genes such as *mex* and *brlR* involved in drug resistance and such as *pslA*, *pelA* and *pelB*, and *brlR* genes of clinical *P. aeruginosa* isolates in biofilm formation.

Of 59 clinical isolates of *P. aeruginosa* obtained from several specimens, 35.6% of the isolates were recovered from urine, 25.42% from sputum, 16.64% from wound, 10.16% from blood, and 11.88% from other specimens. In another research, Karruli reported the frequency ratio of isolates from sputum, urine, surgery sites, and blood specimens separately.

As shown in Table 2, 27.11% of our isolates were MDR, which was similar to Sezadehghani's study which was 29.35% and Arta Karruli's report which was 33.1%. According to the European Antimicrobial Resistance Surveillance Network (EARS-Net), 9.7% of *P. aeruginosa* isolates were resistant to at least three of five antimicrobial groups and 3.1% were resistant to all five antimicrobial groups under surveillance [20].

Moreover, in our study, 70% of all the isolates from wound specimens were MDR; in a similar report from India, it was shown that 76.8% of the isolated *P. aeruginosa* were detected from burn patients [21]. However, in a report from the United States, MDR *P. aeruginosa* isolates, with prevalent carbapenem resistance, were detected from sputum and blood of patients.

Furthermore, a relatively high proportion of resistance to ceftazidime and cefepime (60%) was detected in our isolates from wound infections as well. It is almost different from Batt's findings that show that 76.79% and 71.4% of the burns' isolates were resistant to ceftazidime and cefepime separately [21]. Overall, in our study, most resistance was against ceftriaxone (45.76%) and then imipenem (45.76%), while in India, it was against ceftazidime (76.70%) and tobramycin (75%) [21]; in Europe, it was seen that most resistance was against fluoroquinolones like ciprofloxacin (19.6) and then piperacillin-tazobactam (18.8%) [22].

In the presence of environmental stresses, or under prolonged exposure to subinhibitory antibiotic concentrations, the majority of bacteria exist in surface-adherent biofilm. Biofilm formation, as an important virulence factor, plays a critical role in the pathogenesis of *P. aeruginosa*. It can protect bacteria from immune system clearance and from antibiotics [23]. In our study, analyses of in vitro biofilm production by clinical isolates of *P. aeruginosa* indicated that more than 81.33% of the isolates were biofilm producers in strong, moderate, and weak forms. Interestingly, 100% of the detected *P. aeruginosa* isolates from the wound specimen and 85.71% from the urine specimens were biofilm producers compared with the biofilm producers isolated from other infection sites in this study. Overall, the results of our study indicated that 50.84%, 23.72%, and 6.77% of the biofilm forming *P. aeruginosa* strains produced strong, moderate, and weak biofilms separately. Dolatshah and Tabatabaei in their investigation found that 69% of the *P. aeruginosa* isolates were biofilm producers, but only 9% of them formed strong biofilm, and the other strains were moderate and weak biofilm producers [24].

There are various environmental and genetic factors that can influence the biofilm formation of *P*. *aeruginosa*, but certainly *psl* and *pel* genes contribute to biofilm formation [25, 26]. Also, there are several genes such as *mexA* and *mexB* involved in drug resistance in *P. aeruginosa*, which act as the drug efflux pump, and *brlR* contributes to the high-level drug tolerance of *P. aeruginosa* biofilms [9, 10], as followed in our study.

We found that *pslA* gene was detected in 91.5% of the isolates, while *pelA* and *pelB* genes were found in 69.49% of the *P. aeruginosa* isolates. In a similar study, Dolatshah and Tabatabaei reported that “92% of the isolates possessed the *pslA* gene” [24]. However, Ghadaksaz showed that 83.7% of their isolates had *psl*, and 45.2% of them possessed *pelA* genes, which was different from our research. Based on our finding, we can conclude that the *pslA* gene was more effective in the biofilm formation of the isolated *P. aeruginosa* compared to the *pel* gene.

Although Márió Gajdács et al. found no relationships between biofilm formation and virulence factors of *P. aeruginosa*, in our study, the researchers investigated the relationship between biofilm formation and several virulence factors such as pigment production and motility, not the *pslA* gene under study.

Płókarz et al. have expressed that *pslA* and *pelA* play 60.1% and 38.7% roles in biofilm formation of *P. aeruginosa* in animals, respectively [27]. Ghadaksaz et al.' study also showed a significant relationship between *pslA* and *pelA* genes and biofilm formation [28]. These recent reports validate our findings.

In this study, the frequency of *mexA*, *mexB*, and *brlR* genes, which are involved in resistance phenomenon, efflux pump, and biofilm drug resistance in *P. aeruginosa*, was investigated. Table 3 shows that the distribution of the three genes was 94.91%, 50.84%, and 94.91%, respectively. Bhandari et al. reported that 70.6% of the isolates from all of the examined *P. aeruginosa* isolates possessed *mexA* and *mexB* which are involved in the efflux pump [29]. Based on our data, we can conclude that the *mexA* gene in our study played a critical role in causing drug resistance compared to *mexB*.

The *brlR* gene, as a member of the MerR family, “is expressed only in biofilms” and plays a significant role in drug tolerance in *P. aeruginosa*. It is found that the *brlR* gene acts as a drug efflux pump to resistance of antibiotics against *P. aeruginosa* [30].

In our study, *brlR* (94.91%) played an essential role in causing resistance to the formed biofilms. Our examined microbroth dilution tests for determining MIC and MBC levels against the two important antibiotics, imipenem and ciprofloxacin, revealed that none of the antibiotics were able to prevent biofilm formation at a concentration of <512 *µ*g/mL. For some strains, a decrease in biofilm formation was observed when imipenem at a concentration of >512 *µ*g/mL was used, but it was not able to eradicate biofilm formation. Musafer et al. showed that there was a strong link between biofilm formation and imipenem resistance in *P. aeruginosa* isolates [31].

Ciprofloxacin was not able to eradicate the bacteria that formed biofilms at any concentration in our study. In a similar report by Takrami and Ranji, it was observed that the *P. aeruginosa* isolates were resistant to ciprofloxacin due to the overexpression of *mexAB* operon [32].

For *P. aeruginosa* phenotyping and genotyping, different techniques have been developed; however, PFGE is still considered to be the gold standard molecular typing method for genotyping *P. aeruginosa* which has shown a high index of diversity [11].

According to Figure 1, the rate of genomic similarity, depending on using an 80% similarity cutoff point, reveals that PFGE pattern (pulsotypes) analysis resulted in 13 distinctive pulsotypes (81.35%) and 10 unique singletons (18.6%) of *P. aeruginosa* isolates. Karami et al. similarly used PFGE to genotype *P. aeruginosa* isolates and found a high level of diversity, with 22 pulsotypes identified [33].

Overall, a review of the total results in this study reveals that 35.6% of all the samples were isolated from urine and 16.94% from wound. Among the total MDR isolates (27.11%), 8.48% of the isolates were detected from urine and 11.86% from wound. 85.71% of the detected isolates from urine and 100% from the wound specimens were able to form biofilm, so that 52.38% and 70% of the formed biofilms separately were strong. Furthermore, Awan et al. reported a significantly higher prevalence of strong biofilm formation in MDR *P. aeruginosa* isolates when compared to our findings [34].

**Figure 2:**
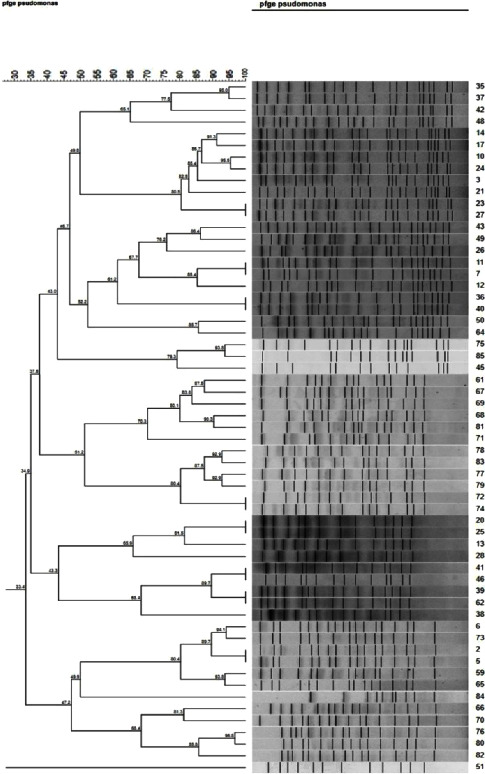
Dendrogram and cluster analysis of *P. aeruginosa* isolates detected in the present study by pulsed-field gel electrophoresis (PFGE).

Figure 2 and Table 6 demonstrate that in PFGE analysis, pulsotype 2 with 8 (13.6%) isolates was predominant among the others followed by pulsotypes 8, 11, and 7. In Nikbin et al.'s study, 84 resistance banding patterns were detected from two hospitals; there was the dominant pattern of 14 isolates at both hospitals [35].

Comparison of the dendrogram of the isolates revealed that isolated *P. aeruginosa* detected from urine specimens was present in 12 different pulsotypes, while the isolates recovered from wound specimens existed in only 6 separate pulsotypes. Based on the mentioned concepts, it may be concluded that biofilm formation causes a urinary tract infection. In the same direction, Uner-Kayabas announced that *P. aeruginosa* usually infects the urinary tract by strong adherence to the bladder uroepithelium, and *P. aeruginosa* usually affects the urinary tract through ascending infection and adheres strongly to the bladder uroepithelium.

Finally, according to our findings in urine exams, 100% of the main responsible genes in biofilm formation belonged to *pslA* and 95.23% of the MDR genes were *mexA*. We can conclude that the urinary tract can play a key role in maintaining and transferring biofilm drug-resistant *P. aeruginosa.* This is in the same line with Cholley's opinion that believed bacterial “cross-transmission” between infected patients was responsible for the isolated MDR presentation [36].

We believe that future studies on UTI among people prone to urinary tract infections, such as young children, pregnant and older individuals, or kidney failure patients, should be focused on the pattern of their genetic exchange, biofilm formation, and drug resistance in their UTI recurrence. It is also important to follow the patients to ensure successful treatment and negative growth of their urine culture to control the recurrence of the infection. For this purpose, it is suggested two urine cultures should be performed in prospective studies [37]. Briefly, a urine culture and an antibiotic susceptibility test, for detected bacteria, should be performed four days after starting the prescribed choice of antibiotics and ten days after stopping them, in cases of positive urine cultures. If the antibiotic therapy proves effective in both steps, it signifies a suitable treatment and reduces the chances of recurrent infection. Positive urine cultures at any stage of the tests can indicate a recurrent urinary tract infection because of biofilm presence centers in the urinary tract, which is hazardous to patients and poses a risk to public health.

### 4.1. Limitation

The main limitation of this study was the lack of compatibility between in vitro and in vivo experimental conditions. However, considering the urgency in treating drug-resistant infectious diseases in humans, we have no choice but to rely on laboratory experiments.

## Figures and Tables

**Table 1:** The specific primers of *pslA*, *pelA* and *pelB*, *brlR*, *mexA*, and *mexB* genes in PCR amplification.

Target	Primer sequence (5′ ⟶ 3′)	Product size (bp)	Annealing temperature (°C)	Reference
*pslA*	F: TGCCTGGAACATAATCACCGT	202	52	This study
	R: GTCGGTAGATAGCCTGTCGC			

*pelA*	F: GACTGGGTGGTGCTCGAAG	312	52	This study
	R: GCGCTCCTCGGCCTGTAG			

*brlR*	F: GCAACGACACCAGCACACC	269	52	This study
	R:CGCAGATGCCATAGGAGACC			

*mexA*	F: CTGAAGCTGGAGGACGGTAG	356	52	This study
	R: AGGCCTTCGGTAATGATCTTGT			

*mexB*	F: TGGGTGATCGCCTTGGTGA	307	52	This study
	R: GGCCAGTTGCAGCTTGTTC			

**Table 2:** Antibiotic susceptibility patterns of *P*. *aeruginosa* isolates recovered from clinical specimens.

Specimen	CP^R(%)^	IPM^R(%)^	GM^R(%)^	AN^R(%)^	CAZ^R(%)^	CRO^R(%)^	FEP^R(%)^	MDR (*N*/%)	Specimen number (%)
Urine	6	6	3	3	3	7	3	5 (8.48)	21 (35.6)
Skin	1	1	0	0	0	2	0	0 (0)	4 (6.8)
Sputum	6	6	0	0	0	6	0	2 (3.38)	15 (25.42)
Body fluid	1	1	0	0	0	1	0	0 (0)	3 (5.08)
Blood	3	4	1	1	2	2	1	2 (3.38)	6 (10.16)
Wound	8	9	6	5	6	9	6	7 (11.86)	10 (16.94)
Total	25 (42.37)	27 (45.76)	10 (16.94)	9 (15.25)	11 (18.64)	27 (45.76)	10 (16.94)	16 (27.11)	59 (100)

CP: ciprofloxacin, IPM: imipenem, GM: gentamicin, AN: amikacin, CAZ: ceftazidime, CRO: ceftriaxone, FEP: cefepime, and S/R: sensitive/resistance.

**Table 3:** Distribution of *pslA*, *pelA* and *pelB*, *brlR*, *mexA*, and *mexB* genes of *P. aeruginosa* in clinical specimens.

Specimens (number)	Virulence gene				
	*pslA* (*N*/%)	*pelA* and *pelB* (*N*/%)	*brlR* (*N*/%)	*mexA* (*N*/%)	*mexB* (*N*/%)
Urine (21)	21 (100)	12 (5.71)	1 (4.76)	20 (95.23)	11 (52.38)
Sputum (15)	14 (93.33)	12 (80)	14 (93.33)	13 (86.66)	7 (46.66)
Skin (4)	4 (100)	3 (75)	4 (100)	4 (100)	2 (50)
Body fluid (3)	3 (100)	3 (100)	3 (100)	3 (100)	2 (66.66)
Blood (6)	6 (100)	5 (83.33)	6 (100)	6 (100)	4 (66.66)
Wound (10)	6 (60)	6 (60)	10 (100)	10 (100)	4 (40)
Total (59)	54 (91.5%)	41 (69.49%)	38 (64.4%)	56 (94.91%)	30 (50.84%)

**Table 4:** Distribution of biofilm formation of *Pseudomonas aeruginosa* in clinical specimens.

Specimens (*N*)	Biofilm producing *Pseudomonas aeruginosa* (*N*/%)				Biofilm producer isolates (*N*/%)
	Strong	Moderate	Weak	None	
Urine (21)	11 (52.38)	5 (23.80)	2 (9.52)	3 (14.28)	18 (85.71)
Skin (4)	1 (25)	1 (25)	1 (25)	1 (25)	3 (75)
Sputum (15)	7 (46.66)	4 (26.66)	1 (6.66)	3 (20)	12 (80)
Body fluid (3)	1 (33.33)	1 (33.33)	0 (0)	1 (33.33)	2 (66.66)
Blood (6)	3 (50)	0 (0)	0 (0)	3 (50)	3 (50)
Wound (10)	7 (70)	3 (30)	0 (0)	0 (0)	10 (100)
Total (59)	30 (50.84)	14 (23.72)	4 (6.77)	11 (18.84)	48 (81.33)

**Table 5:** The distribution of the virulence genes among 13 distinctive clones and 11 single clones.

Clonal number	Number (%) of isolates in each clone	Number (%) with *pslA*gene	Number (%) with *pelA*gene	Number (%) with *brlR*gene
1	2 (3.4)	1 (50)	0 (0)	2 (100%)
2	8 (13.6)	8 (100)	4 (50)	8 (100%)
3	2 (3.4)	1 (50)	0 (0)	2 (100%)
4	3 (5.1)	3 (100)	1 (33.3)	3 (100%)
5	2 (3.4)	1 (50)	1 (50)	2 (100%)
6	2 (3.4)	2 (100)	2 (100)	2 (100%)
7	5 (8.5)	5 (100)	5 (100)	5 (100%)
8	6 (10.2)	6 (100)	6 (100)	4 (66.7%)
9	3 (5.1)	3 (100)	2 (66.7)	3 (100%)
10	4 (6.8)	3 (75)	3 (75)	4 (100%)
11	6 (10.2)	6 (100)	4 (66.7)	6 (100%)
12	2 (3.4)	2 (100)	1 (50)	2 (100%)
13	3 (5.1)	3 (100)	3 (100)	1 (33.3%)
Single clones	11 (18.6)	10 (90.9)	9 (81.8)	11 (100)

**Table 6:** Comparison of the dendrogram of the isolated *P. aeruginosa* from clinical specimens.

Pulsotypes	Dendrogram	Number of isolates	Type of sample (number)
I	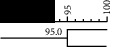	2	U.C (1), W.C (1)
II	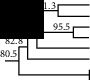	8	U.C (3), B.C (2), SP.C (1), S.C (1), W.C (1)
III	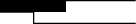	2	U.C (1), W.C (1)
IV	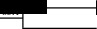	3	SP.C (2), U.C (1)
V	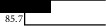	2	B.C (1), SP.C (1)
VI	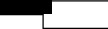	2	U.C (1). W.C (1)
VII	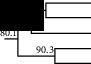	5	SP.C (2), U.C (2), F.C (1)
VIII	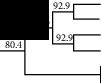	6	SP.C (2), U.C (2), W.C (1), F.C (1)
IX	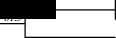	3	U.C (2), B.C (1)
X	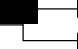 0	4	U.C (2), W.C (1), B.C (1)
XI	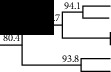	6	U.C (2), SP.C (3), F.C (1)
XII	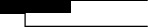	2	U.C (1), F.C (1)
XIII	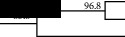	3	SP.C (1), U.C (1), S.C (1)

B: blood, C: culture, F: fluid, S: skin, SP: sputum, U: urine, and W: wound.

## Data Availability

The data used to support the findings of this study are included within the article.

## Supplementary Materials

Supplementary Materials Original pictures and primer-blast results.
