# Supplementary material for: Phylogenetic Aspects of Antibiotic Resistance and Biofilm Formation of P. aeruginosa Isolated from Clinical Samples
Source: Can J Infect Dis Med Microbiol. 2024 Jan 13;2024:6213873. doi: 10.1155/2024/6213873 (PMC10799695; doi:10.1155/2024/6213873)
Supplement: Supplementary Materials — Original pictures and primer-blast results. [file 6213873.f1.zip › brlR Primer-Blast results.pdf]

An official website of the United States government

Here's how you know

The .gov means it’s official.
Federal government websites often end in .gov or .mil. Before sharing sensitive information, make sure you’re on a federal government site.

The site is secure.
The https:// ensures that you are connecting to the official website and that any information you provide is encrypted and transmitted securely.

Skip to main page content

Access keys NCBI Homepage MyNCBI Homepage Main Content Main Navigation

Log in

Primer-BLAST

» JOB ID:6OI3d56ukwa0OIk9hF2tD_5GvD3TVacg0g

PrimerBLAST users!
We want to hear from you about how PrimerBLAST can be improved.
Contact us

Primer-BLAST Results
Help

•

Input PCR template
none

Specificity of primers
Target templates were found in selected database: Nucleotide collection (nt)

Other reports
Search Summary

Detailed primer reports
You can re-search for specific primers by accepting some of the unintended targets, check the box(es) next to the ones you accept and try again to re-search for specific primers Submit
Help

Primer pair 1

	Sequence (5'->3')	Length	Tm	GC%	Self complementarity	Self 3' complementarity
Forward primer	GCAACGACACCAGCACACC	19	62.18	63.16	2.00	0.00
Reverse primer	CGCAGATGCCATAGGAGACC	20	60.32	60.00	5.00	1.00

Products on intended targets

Products on allowed targets

Products on allowed transcript variants

Products on potentially unintended templates

Products on target templates

>CP127016.1 Pseudomonas aeruginosa strain TBCF10839 chromosome

```
product length = 269
Forward primer  1        GCAACGACACCAGCACACC  19
Template        5226091  ...................  5226073

Reverse primer  1        CGCAGATGCCATAGGAGACC  20
Template        5225823  ....................  5225842
```

>CP123792.1 Pseudomonas aeruginosa strain 2021CK-01658 chromosome, complete genome

```
product length = 269
Forward primer  1       GCAACGACACCAGCACACC  19
Template        963815  ...................  963797

Reverse primer  1       CGCAGATGCCATAGGAGACC  20
Template        963547  ....................  963566
```

>CP109683.1 Pseudomonas aeruginosa strain 2017-45-169 chromosome, complete genome

```
product length = 269
Forward primer  1        GCAACGACACCAGCACACC  19
Template        3496906  ...................  3496888

Reverse primer  1        CGCAGATGCCATAGGAGACC  20
Template        3496638  ....................  3496657
```

>CP123789.1 Pseudomonas aeruginosa strain 2021CK-01381 chromosome, complete genome

```
product length = 269
Forward primer  1       GCAACGACACCAGCACACC  19
Template        958421  ...................  958403

Reverse primer  1       CGCAGATGCCATAGGAGACC  20
Template        958153  ....................  958172
```

>CP127126.1 Pseudomonas aeruginosa PA14 strain MA3 isolate DTU_MIE chromosome, complete genome

```
product length = 269
Forward primer  1        GCAACGACACCAGCACACC  19
Template        5748791  ...................  5748809

Reverse primer  1        CGCAGATGCCATAGGAGACC  20
Template        5749059  ....................  5749040
```

>CP121766.1 Pseudomonas aeruginosa strain 22112 chromosome, complete genome

```
product length = 269
Forward primer  1        GCAACGACACCAGCACACC  19
Template        5405001  ...................  5405019

Reverse primer  1        CGCAGATGCCATAGGAGACC  20
Template        5405269  ....................  5405250
```

>CP109757.1 Pseudomonas aeruginosa strain 2017-45-85 chromosome, complete genome

```
product length = 269
Forward primer  1        GCAACGACACCAGCACACC  19
Template        6146924  ...................  6146942

Reverse primer  1        CGCAGATGCCATAGGAGACC  20
```

```
Template        6147192  ....................  6147173
```

>CP109685.1 Pseudomonas aeruginosa strain 2017-45-137A chromosome, complete genome

```
product length = 269
Forward primer  1        GCAACGACACCAGCACACC  19
Template        3982702  ...................  3982720

Reverse primer  1        CGCAGATGCCATAGGAGACC  20
Template        3982970  ....................  3982951
```

>CP061073.2 Pseudomonas aeruginosa strain PAD8 chromosome, complete genome

```
product length = 269
Forward primer  1        GCAACGACACCAGCACACC  19
Template        2442083  ...................  2442101

Reverse primer  1        CGCAGATGCCATAGGAGACC  20
Template        2442351  ....................  2442332
```

>CP123786.1 Pseudomonas aeruginosa strain 2021CK-01424 chromosome, complete genome

```
product length = 269
Forward primer  1        GCAACGACACCAGCACACC  19
Template        6162874  ...................  6162892

Reverse primer  1        CGCAGATGCCATAGGAGACC  20
Template        6163142  ....................  6163123
```

>CP123785.1 Pseudomonas aeruginosa strain 2021CK-01267 chromosome, complete genome

```
product length = 269
Forward primer  1        GCAACGACACCAGCACACC  19
Template        6163242  ...................  6163260

Reverse primer  1        CGCAGATGCCATAGGAGACC  20
Template        6163510  ....................  6163491
```

>CP123787.1 Pseudomonas aeruginosa strain 2020CK-00194 chromosome, complete genome

```
product length = 269
Forward primer  1        GCAACGACACCAGCACACC  19
Template        6307743  ...................  6307761

Reverse primer  1        CGCAGATGCCATAGGAGACC  20
Template        6308011  ....................  6307992
```

>CP123791.1 Pseudomonas aeruginosa strain 2021CK-01305 chromosome, complete genome

```
product length = 269
Forward primer  1        GCAACGACACCAGCACACC  19
Template        6106607  ...................  6106625
```

```
Reverse primer  1        CGCAGATGCCATAGGAGACC  20
Template        6106875  ....................  6106856
```

>CP123793.1 Pseudomonas aeruginosa strain 2021CK-01107 chromosome, complete genome

```
product length = 269
Forward primer  1        GCAACGACACCAGCACACC  19
Template        6112631  ...................  6112649

Reverse primer  1        CGCAGATGCCATAGGAGACC  20
Template        6112899  ....................  6112880
```

>CP096964.1 Pseudomonas aeruginosa strain NY13936 chromosome, complete genome

```
product length = 269
Forward primer  1        GCAACGACACCAGCACACC  19
Template        5952240  ...................  5952258

Reverse primer  1        CGCAGATGCCATAGGAGACC  20
Template        5952508  ....................  5952489
```

>CP096961.1 Pseudomonas aeruginosa strain NY13932 chromosome, complete genome

```
product length = 269
Forward primer  1        GCAACGACACCAGCACACC  19
Template        5624081  ...................  5624099

Reverse primer  1        CGCAGATGCCATAGGAGACC  20
Template        5624349  ....................  5624330
```

>CP096960.1 Pseudomonas aeruginosa strain NY11254 chromosome, complete genome

```
product length = 269
Forward primer  1        GCAACGACACCAGCACACC  19
Template        6221928  ...................  6221946

Reverse primer  1        CGCAGATGCCATAGGAGACC  20
Template        6222196  ....................  6222177
```

>CP096956.1 Pseudomonas aeruginosa strain NY11173 chromosome, complete genome

```
product length = 269
Forward primer  1        GCAACGACACCAGCACACC  19
Template        5776964  ...................  5776982

Reverse primer  1        CGCAGATGCCATAGGAGACC  20
Template        5777232  ....................  5777213
```

>CP096950.1 Pseudomonas aeruginosa strain NY5532 chromosome, complete genome

```
product length = 269
Forward primer  1        GCAACGACACCAGCACACC  19
Template        6004142  ...................  6004160
```

```
Reverse primer  1        CGCAGATGCCATAGGAGACC  20
Template        6004410  ....................  6004391
```

>CP096945.1 Pseudomonas aeruginosa strain NY5525 chromosome, complete genome

```
product length = 269
Forward primer  1        GCAACGACACCAGCACACC  19
Template        5921808  ...................  5921826

Reverse primer  1        CGCAGATGCCATAGGAGACC  20
Template        5922076  ....................  5922057
```

>CP096942.1 Pseudomonas aeruginosa strain NY5524 chromosome, complete genome

```
product length = 269
Forward primer  1        GCAACGACACCAGCACACC  19
Template        6383568  ...................  6383586

Reverse primer  1        CGCAGATGCCATAGGAGACC  20
Template        6383836  ....................  6383817
```

>CP096941.1 Pseudomonas aeruginosa strain NY5523 chromosome, complete genome

```
product length = 269
Forward primer  1        GCAACGACACCAGCACACC  19
Template        6095565  ...................  6095583

Reverse primer  1        CGCAGATGCCATAGGAGACC  20
Template        6095833  ....................  6095814
```

>CP096927.1 Pseudomonas aeruginosa strain NY5506 chromosome, complete genome

```
product length = 269
Forward primer  1        GCAACGACACCAGCACACC  19
Template        5539265  ...................  5539283

Reverse primer  1        CGCAGATGCCATAGGAGACC  20
Template        5539533  ....................  5539514
```

>CP084890.1 Pseudomonas aeruginosa strain CH1 chromosome

```
product length = 270
Forward primer  1       GCAACGACACCAGCACACC  19
Template        909719  ...................  909701

Reverse primer  1       CGCAGATGCCATAGGAGACC  20
Template        909450  ....................  909469
```

>CP124652.1 Pseudomonas aeruginosa strain 2020CK-00443 chromosome, complete genome

```
product length = 269
Forward primer  1       GCAACGACACCAGCACACC  19
```

```
Template        960178  ...................  960160

Reverse primer  1       CGCAGATGCCATAGGAGACC  20
Template        959910  ....................  959929
```

>CP124655.1 Pseudomonas aeruginosa strain 2022CK-00096 chromosome, complete genome

```
product length = 269
Forward primer  1       GCAACGACACCAGCACACC  19
Template        981442  ...................  981424

Reverse primer  1       CGCAGATGCCATAGGAGACC  20
Template        981174  ....................  981193
```

>CP124643.1 Pseudomonas aeruginosa strain 2021CK-01197 chromosome, complete genome

```
product length = 269
Forward primer  1        GCAACGACACCAGCACACC  19
Template        5702800  ...................  5702782

Reverse primer  1        CGCAGATGCCATAGGAGACC  20
Template        5702532  ....................  5702551
```

>CP124626.1 Pseudomonas aeruginosa strain 2021CK-01161 chromosome, complete genome

```
product length = 269
Forward primer  1        GCAACGACACCAGCACACC  19
Template        1941162  ...................  1941144

Reverse primer  1        CGCAGATGCCATAGGAGACC  20
Template        1940894  ....................  1940913
```

>CP124673.1 Pseudomonas aeruginosa strain 2022CK-00491 chromosome, complete genome

```
product length = 269
Forward primer  1        GCAACGACACCAGCACACC  19
Template        6274837  ...................  6274855

Reverse primer  1        CGCAGATGCCATAGGAGACC  20
Template        6275105  ....................  6275086
```

>CP124674.1 Pseudomonas aeruginosa strain 2022CK-00339 chromosome, complete genome

```
product length = 269
Forward primer  1        GCAACGACACCAGCACACC  19
Template        6268434  ...................  6268452

Reverse primer  1        CGCAGATGCCATAGGAGACC  20
Template        6268702  ....................  6268683
```

>CP124662.1 Pseudomonas aeruginosa strain 2021CK-01633 chromosome, complete genome

```
product length = 269
```

```
Forward primer  1        GCAACGACACCAGCACACC  19
Template        6288788  ...................  6288806

Reverse primer  1        CGCAGATGCCATAGGAGACC  20
Template        6289056  ....................  6289037
```

>CP124660.1 Pseudomonas aeruginosa strain 2022CK-00160 chromosome, complete genome

```
product length = 269
Forward primer  1        GCAACGACACCAGCACACC  19
Template        6322078  ...................  6322096

Reverse primer  1        CGCAGATGCCATAGGAGACC  20
Template        6322346  ....................  6322327
```

>CP124649.1 Pseudomonas aeruginosa strain 2020CK-00218 chromosome, complete genome

```
product length = 269
Forward primer  1        GCAACGACACCAGCACACC  19
Template        6291143  ...................  6291161

Reverse primer  1        CGCAGATGCCATAGGAGACC  20
Template        6291411  ....................  6291392
```

>CP124654.1 Pseudomonas aeruginosa strain 2021CK-01851 chromosome, complete genome

```
product length = 269
Forward primer  1        GCAACGACACCAGCACACC  19
Template        5646752  ...................  5646770

Reverse primer  1        CGCAGATGCCATAGGAGACC  20
Template        5647020  ....................  5647001
```

>CP124657.1 Pseudomonas aeruginosa strain 2022CK-00069 chromosome, complete genome

```
product length = 269
Forward primer  1        GCAACGACACCAGCACACC  19
Template        6045721  ...................  6045739

Reverse primer  1        CGCAGATGCCATAGGAGACC  20
Template        6045989  ....................  6045970
```

>CP124651.1 Pseudomonas aeruginosa strain 2020CK-00217 chromosome, complete genome

```
product length = 269
Forward primer  1        GCAACGACACCAGCACACC  19
Template        6249278  ...................  6249296

Reverse primer  1        CGCAGATGCCATAGGAGACC  20
Template        6249546  ....................  6249527
```

>CP124669.1 Pseudomonas aeruginosa strain 2021CK-01494 chromosome, complete genome

```
product length = 269
Forward primer  1        GCAACGACACCAGCACACC  19
Template        5773929  ...................  5773947

Reverse primer  1        CGCAGATGCCATAGGAGACC  20
Template        5774197  ....................  5774178
```

>CP124638.1 Pseudomonas aeruginosa strain 2021CK-01158 chromosome, complete genome

```
product length = 269
Forward primer  1        GCAACGACACCAGCACACC  19
Template        5681739  ...................  5681757

Reverse primer  1        CGCAGATGCCATAGGAGACC  20
Template        5682007  ....................  5681988
```

>CP124668.1 Pseudomonas aeruginosa strain 2021CK-01445 chromosome, complete genome

```
product length = 269
Forward primer  1        GCAACGACACCAGCACACC  19
Template        5772406  ...................  5772424

Reverse primer  1        CGCAGATGCCATAGGAGACC  20
Template        5772674  ....................  5772655
```

>CP124666.1 Pseudomonas aeruginosa strain 2021CK-01283 chromosome, complete genome

```
product length = 269
Forward primer  1        GCAACGACACCAGCACACC  19
Template        5695395  ...................  5695413

Reverse primer  1        CGCAGATGCCATAGGAGACC  20
Template        5695663  ....................  5695644
```

>CP124624.1 Pseudomonas aeruginosa strain 2021CK-01157 chromosome, complete genome

```
product length = 269
Forward primer  1        GCAACGACACCAGCACACC  19
Template        5682047  ...................  5682065

Reverse primer  1        CGCAGATGCCATAGGAGACC  20
Template        5682315  ....................  5682296
```

>CP124665.1 Pseudomonas aeruginosa strain 2021CK-01229 chromosome, complete genome

```
product length = 269
Forward primer  1        GCAACGACACCAGCACACC  19
Template        5680382  ...................  5680400

Reverse primer  1        CGCAGATGCCATAGGAGACC  20
Template        5680650  ....................  5680631
```

>CP124667.1 Pseudomonas aeruginosa strain 2021CK-01315 chromosome, complete genome

```
product length = 269
Forward primer  1        GCAACGACACCAGCACACC  19
Template        5804594  ...................  5804612

Reverse primer  1        CGCAGATGCCATAGGAGACC  20
Template        5804862  ....................  5804843
```

>CP124641.1 Pseudomonas aeruginosa strain 2021CK-01198 chromosome, complete genome

```
product length = 269
Forward primer  1        GCAACGACACCAGCACACC  19
Template        5681680  ...................  5681698

Reverse primer  1        CGCAGATGCCATAGGAGACC  20
Template        5681948  ....................  5681929
```

>CP124670.1 Pseudomonas aeruginosa strain 2021CK-01536 chromosome, complete genome

```
product length = 269
Forward primer  1        GCAACGACACCAGCACACC  19
Template        5572349  ...................  5572367

Reverse primer  1        CGCAGATGCCATAGGAGACC  20
Template        5572617  ....................  5572598
```

>CP124646.1 Pseudomonas aeruginosa strain 2020CK-00185 chromosome, complete genome

```
product length = 270
Forward primer  1        GCAACGACACCAGCACACC  19
Template        6033121  ...................  6033139

Reverse primer  1        CGCAGATGCCATAGGAGACC  20
Template        6033390  ....................  6033371
```

>CP124648.1 Pseudomonas aeruginosa strain 2020CK-00220 chromosome, complete genome

```
product length = 269
Forward primer  1        GCAACGACACCAGCACACC  19
Template        1733160  ...................  1733178

Reverse primer  1        CGCAGATGCCATAGGAGACC  20
Template        1733428  ....................  1733409
```

>CP124632.1 Pseudomonas aeruginosa strain 2021CK-01162 chromosome, complete genome

```
product length = 269
Forward primer  1        GCAACGACACCAGCACACC  19
Template        6627338  ...................  6627356

Reverse primer  1        CGCAGATGCCATAGGAGACC  20
Template        6627606  ....................  6627587
```

>CP124663.1 Pseudomonas aeruginosa strain 2021CK-01227 chromosome, complete genome

```
product length = 269
Forward primer  1        GCAACGACACCAGCACACC  19
Template        5681766  ...................  5681784

Reverse primer  1        CGCAGATGCCATAGGAGACC  20
Template        5682034  ....................  5682015
```

>CP124622.1 Pseudomonas aeruginosa strain 2021CK-01159 chromosome, complete genome

```
product length = 269
Forward primer  1        GCAACGACACCAGCACACC  19
Template        5735061  ...................  5735079

Reverse primer  1        CGCAGATGCCATAGGAGACC  20
Template        5735329  ....................  5735310
```

>CP124600.1 Pseudomonas aeruginosa strain Li010 chromosome, complete genome

```
product length = 269
Forward primer  1        GCAACGACACCAGCACACC  19
Template        6100244  ...................  6100262

Reverse primer  1        CGCAGATGCCATAGGAGACC  20
Template        6100512  ....................  6100493
```

>CP116682.1 Pseudomonas aeruginosa strain HS337 chromosome, complete genome

```
product length = 269
Forward primer  1        GCAACGACACCAGCACACC  19
Template        6066580  ...................  6066598

Reverse primer  1        CGCAGATGCCATAGGAGACC  20
Template        6066848  ....................  6066829
```

>CP110190.1 Pseudomonas aeruginosa strain HS204 chromosome, complete genome

```
product length = 269
Forward primer  1        GCAACGACACCAGCACACC  19
Template        6012529  ...................  6012547

Reverse primer  1        CGCAGATGCCATAGGAGACC  20
Template        6012797  ....................  6012778
```

>CP117300.1 Pseudomonas aeruginosa strain 0201761-1 chromosome, complete genome

```
product length = 269
Forward primer  1        GCAACGACACCAGCACACC  19
Template        6084881  ...................  6084899

Reverse primer  1        CGCAGATGCCATAGGAGACC  20
Template        6085149  ....................  6085130
```

>CP084321.1 Pseudomonas aeruginosa strain HS18-89 chromosome, complete genome

```
product length = 269
Forward primer  1        GCAACGACACCAGCACACC  19
Template        6262688  ...................  6262706

Reverse primer  1        CGCAGATGCCATAGGAGACC  20
Template        6262956  ....................  6262937
```

>CP075847.1 Pseudomonas aeruginosa strain PaLo3 chromosome, complete genome

```
product length = 269
Forward primer  1        GCAACGACACCAGCACACC  19
Template        1000823  ...................  1000805

Reverse primer  1        CGCAGATGCCATAGGAGACC  20
Template        1000555  ....................  1000574
```

>CP075846.1 Pseudomonas aeruginosa strain PaLo4 chromosome

```
product length = 269
Forward primer  1       GCAACGACACCAGCACACC  19
Template        902324  ...................  902306

Reverse primer  1       CGCAGATGCCATAGGAGACC  20
Template        902056  ....................  902075
```

>CP075832.1 Pseudomonas aeruginosa strain PaLo20 chromosome, complete genome

```
product length = 269
Forward primer  1        GCAACGACACCAGCACACC  19
Template        1278036  ...................  1278018

Reverse primer  1        CGCAGATGCCATAGGAGACC  20
Template        1277768  ....................  1277787
```

>CP117749.1 Pseudomonas aeruginosa strain 2022CK-00828 chromosome, complete genome

```
product length = 269
Forward primer  1        GCAACGACACCAGCACACC  19
Template        5936257  ...................  5936275

Reverse primer  1        CGCAGATGCCATAGGAGACC  20
Template        5936525  ....................  5936506
```

>CP117527.1 Pseudomonas aeruginosa strain MF1 chromosome, complete genome

```
product length = 269
Forward primer  1        GCAACGACACCAGCACACC  19
Template        6152110  ...................  6152128

Reverse primer  1        CGCAGATGCCATAGGAGACC  20
Template        6152378  ....................  6152359
```

>CP075851.1 Pseudomonas aeruginosa strain PaLo33 chromosome, complete genome

```
product length = 269
Forward primer  1        GCAACGACACCAGCACACC  19
Template        5993684  ...................  5993702

Reverse primer  1        CGCAGATGCCATAGGAGACC  20
Template        5993952  ....................  5993933
```

>CP075849.1 Pseudomonas aeruginosa strain PaLo1 chromosome, complete genome

```
product length = 269
Forward primer  1        GCAACGACACCAGCACACC  19
Template        5778416  ...................  5778434

Reverse primer  1        CGCAGATGCCATAGGAGACC  20
Template        5778684  ....................  5778665
```

>CP075848.1 Pseudomonas aeruginosa strain PaLo2 chromosome, complete genome

```
product length = 269
Forward primer  1        GCAACGACACCAGCACACC  19
Template        5416409  ...................  5416427

Reverse primer  1        CGCAGATGCCATAGGAGACC  20
Template        5416677  ....................  5416658
```

>CP075844.1 Pseudomonas aeruginosa strain PaLo6 chromosome, complete genome

```
product length = 269
Forward primer  1        GCAACGACACCAGCACACC  19
Template        5997888  ...................  5997906

Reverse primer  1        CGCAGATGCCATAGGAGACC  20
Template        5998156  ....................  5998137
```

>CP075843.1 Pseudomonas aeruginosa strain PaLo7 chromosome

```
product length = 269
Forward primer  1        GCAACGACACCAGCACACC  19
Template        5999887  ...................  5999905

Reverse primer  1        CGCAGATGCCATAGGAGACC  20
Template        6000155  ....................  6000136
```

>CP075841.1 Pseudomonas aeruginosa strain PaLo9 chromosome, complete genome

```
product length = 269
Forward primer  1        GCAACGACACCAGCACACC  19
Template        6143218  ...................  6143236

Reverse primer  1        CGCAGATGCCATAGGAGACC  20
Template        6143486  ....................  6143467
```

>CP075840.1 Pseudomonas aeruginosa strain PaLo10 chromosome, complete genome

```
product length = 269
Forward primer  1        GCAACGACACCAGCACACC  19
Template        5669792  ...................  5669810

Reverse primer  1        CGCAGATGCCATAGGAGACC  20
Template        5670060  ....................  5670041
```

>CP075838.1 Pseudomonas aeruginosa strain PaLo11 chromosome, complete genome

```
product length = 269
Forward primer  1        GCAACGACACCAGCACACC  19
Template        5667043  ...................  5667061

Reverse primer  1        CGCAGATGCCATAGGAGACC  20
Template        5667311  ....................  5667292
```

>CP075836.1 Pseudomonas aeruginosa strain PaLo12 chromosome, complete genome

```
product length = 269
Forward primer  1        GCAACGACACCAGCACACC  19
Template        5673839  ...................  5673857

Reverse primer  1        CGCAGATGCCATAGGAGACC  20
Template        5674107  ....................  5674088
```

>CP075835.1 Pseudomonas aeruginosa strain PaLo14 chromosome, complete genome

```
product length = 269
Forward primer  1        GCAACGACACCAGCACACC  19
Template        5996771  ...................  5996789

Reverse primer  1        CGCAGATGCCATAGGAGACC  20
Template        5997039  ....................  5997020
```

>CP075833.1 Pseudomonas aeruginosa strain PaLo17 chromosome, complete genome

```
product length = 269
Forward primer  1        GCAACGACACCAGCACACC  19
Template        5425772  ...................  5425790

Reverse primer  1        CGCAGATGCCATAGGAGACC  20
Template        5426040  ....................  5426021
```

>CP075831.1 Pseudomonas aeruginosa strain PaLo21 chromosome, complete genome

```
product length = 269
Forward primer  1        GCAACGACACCAGCACACC  19
Template        5584501  ...................  5584519

Reverse primer  1        CGCAGATGCCATAGGAGACC  20
Template        5584769  ....................  5584750
```

>CP075830.1 Pseudomonas aeruginosa strain PaLo22 chromosome, complete genome

```
product length = 269
Forward primer  1        GCAACGACACCAGCACACC  19
Template        5451642  ...................  5451660

Reverse primer  1        CGCAGATGCCATAGGAGACC  20
Template        5451910  ....................  5451891
```

>CP075828.1 Pseudomonas aeruginosa strain PaLo26 chromosome, complete genome

```
product length = 269
Forward primer  1        GCAACGACACCAGCACACC  19
Template        5707102  ...................  5707120

Reverse primer  1        CGCAGATGCCATAGGAGACC  20
Template        5707370  ....................  5707351
```

>CP075827.1 Pseudomonas aeruginosa strain PaLo27 chromosome, complete genome

```
product length = 269
Forward primer  1        GCAACGACACCAGCACACC  19
Template        6094073  ...................  6094091

Reverse primer  1        CGCAGATGCCATAGGAGACC  20
Template        6094341  ....................  6094322
```

>CP075825.1 Pseudomonas aeruginosa strain PaLo30 chromosome, complete genome

```
product length = 269
Forward primer  1        GCAACGACACCAGCACACC  19
Template        5468986  ...................  5469004

Reverse primer  1        CGCAGATGCCATAGGAGACC  20
Template        5469254  ....................  5469235
```

>CP075824.1 Pseudomonas aeruginosa strain PaLo31 chromosome, complete genome

```
product length = 269
Forward primer  1        GCAACGACACCAGCACACC  19
Template        6083951  ...................  6083969

Reverse primer  1        CGCAGATGCCATAGGAGACC  20
Template        6084219  ....................  6084200
```

>CP075823.1 Pseudomonas aeruginosa strain PaLo32 chromosome, complete genome

```
product length = 269
Forward primer  1        GCAACGACACCAGCACACC  19
Template        5987311  ...................  5987329

Reverse primer  1        CGCAGATGCCATAGGAGACC  20
Template        5987579  ....................  5987560
```

>CP075822.1 Pseudomonas aeruginosa strain PaLo34 chromosome, complete genome

```
product length = 269
Forward primer  1        GCAACGACACCAGCACACC  19
Template        5468983  ...................  5469001

Reverse primer  1        CGCAGATGCCATAGGAGACC  20
Template        5469251  ....................  5469232
```

>CP075821.1 Pseudomonas aeruginosa strain PaLo35 chromosome, complete genome

```
product length = 269
Forward primer  1        GCAACGACACCAGCACACC  19
Template        5869668  ...................  5869686

Reverse primer  1        CGCAGATGCCATAGGAGACC  20
Template        5869936  ....................  5869917
```

>CP075820.1 Pseudomonas aeruginosa strain PaLo36 chromosome, complete genome

```
product length = 269
Forward primer  1        GCAACGACACCAGCACACC  19
Template        5445761  ...................  5445779

Reverse primer  1        CGCAGATGCCATAGGAGACC  20
Template        5446029  ....................  5446010
```

>CP075819.1 Pseudomonas aeruginosa strain PaLo37 chromosome, complete genome

```
product length = 269
Forward primer  1        GCAACGACACCAGCACACC  19
Template        5278405  ...................  5278423

Reverse primer  1        CGCAGATGCCATAGGAGACC  20
Template        5278673  ....................  5278654
```

>CP075818.1 Pseudomonas aeruginosa strain PaLo38 chromosome, complete genome

```
product length = 269
Forward primer  1        GCAACGACACCAGCACACC  19
Template        5468962  ...................  5468980

Reverse primer  1        CGCAGATGCCATAGGAGACC  20
Template        5469230  ....................  5469211
```

>CP075816.1 Pseudomonas aeruginosa strain PaLo40 chromosome, complete genome

```
product length = 269
Forward primer  1        GCAACGACACCAGCACACC  19
Template        5586813  ...................  5586831

Reverse primer  1        CGCAGATGCCATAGGAGACC  20
Template        5587081  ....................  5587062
```

>CP075815.1 Pseudomonas aeruginosa strain PaLo43 chromosome, complete genome

```
product length = 269
Forward primer  1        GCAACGACACCAGCACACC  19
Template        5514114  ...................  5514132

Reverse primer  1        CGCAGATGCCATAGGAGACC  20
Template        5514382  ....................  5514363
```

>CP075813.1 Pseudomonas aeruginosa strain PaLo45 chromosome, complete genome

```
product length = 269
Forward primer  1        GCAACGACACCAGCACACC  19
Template        5462400  ...................  5462418

Reverse primer  1        CGCAGATGCCATAGGAGACC  20
Template        5462668  ....................  5462649
```

>CP075812.1 Pseudomonas aeruginosa strain PaLo46 chromosome, complete genome

```
product length = 269
Forward primer  1        GCAACGACACCAGCACACC  19
Template        6039153  ...................  6039171

Reverse primer  1        CGCAGATGCCATAGGAGACC  20
Template        6039421  ....................  6039402
```

>CP075810.1 Pseudomonas aeruginosa strain PaLo166 chromosome, complete genome

```
product length = 269
Forward primer  1        GCAACGACACCAGCACACC  19
Template        5699332  ...................  5699350

Reverse primer  1        CGCAGATGCCATAGGAGACC  20
Template        5699600  ....................  5699581
```

>CP075809.1 Pseudomonas aeruginosa strain PaLo170 chromosome, complete genome

```
product length = 269
Forward primer  1        GCAACGACACCAGCACACC  19
Template        5669824  ...................  5669842

Reverse primer  1        CGCAGATGCCATAGGAGACC  20
Template        5670092  ....................  5670073
```

>CP075806.1 Pseudomonas aeruginosa strain PaLo226 chromosome, complete genome

```
product length = 269
Forward primer  1        GCAACGACACCAGCACACC  19
Template        5483917  ...................  5483935

Reverse primer  1        CGCAGATGCCATAGGAGACC  20
Template        5484185  ....................  5484166
```

>CP075805.1 Pseudomonas aeruginosa strain PaLo227 chromosome, complete genome

```
product length = 269
Forward primer  1        GCAACGACACCAGCACACC  19
Template        5478710  ...................  5478728

Reverse primer  1        CGCAGATGCCATAGGAGACC  20
Template        5478978  ....................  5478959
```

>CP075804.1 Pseudomonas aeruginosa strain PaLo228 chromosome, complete genome

```
product length = 269
Forward primer  1        GCAACGACACCAGCACACC  19
Template        5475107  ...................  5475125

Reverse primer  1        CGCAGATGCCATAGGAGACC  20
Template        5475375  ....................  5475356
```

>CP075803.1 Pseudomonas aeruginosa strain PaLo229 chromosome, complete genome

```
product length = 269
Forward primer  1        GCAACGACACCAGCACACC  19
Template        5610189  ...................  5610207

Reverse primer  1        CGCAGATGCCATAGGAGACC  20
Template        5610457  ....................  5610438
```

>CP075802.1 Pseudomonas aeruginosa strain PaLo240 chromosome, complete genome

```
product length = 269
Forward primer  1        GCAACGACACCAGCACACC  19
Template        6079345  ...................  6079363

Reverse primer  1        CGCAGATGCCATAGGAGACC  20
Template        6079613  ....................  6079594
```

>CP075801.1 Pseudomonas aeruginosa strain PaLo249 chromosome, complete genome

```
product length = 269
Forward primer  1        GCAACGACACCAGCACACC  19
Template        5478708  ...................  5478726

Reverse primer  1        CGCAGATGCCATAGGAGACC  20
Template        5478976  ....................  5478957
```

>CP075800.1 Pseudomonas aeruginosa strain PaLo297 chromosome, complete genome

```
product length = 269
Forward primer  1        GCAACGACACCAGCACACC  19
Template        5614306  ...................  5614324

Reverse primer  1        CGCAGATGCCATAGGAGACC  20
Template        5614574  ....................  5614555
```

>CP075799.1 Pseudomonas aeruginosa strain PaLo310 chromosome, complete genome

```
product length = 269
Forward primer  1        GCAACGACACCAGCACACC  19
Template        5530135  ...................  5530153

Reverse primer  1        CGCAGATGCCATAGGAGACC  20
Template        5530403  ....................  5530384
```

>CP075796.1 Pseudomonas aeruginosa strain PaLo402 chromosome, complete genome

```
product length = 269
Forward primer  1        GCAACGACACCAGCACACC  19
Template        5552643  ...................  5552661

Reverse primer  1        CGCAGATGCCATAGGAGACC  20
Template        5552911  ....................  5552892
```

>CP075787.1 Pseudomonas aeruginosa strain PaLo504 chromosome, complete genome

```
product length = 269
Forward primer  1        GCAACGACACCAGCACACC  19
Template        5581753  ...................  5581771

Reverse primer  1        CGCAGATGCCATAGGAGACC  20
Template        5582021  ....................  5582002
```

>CP075785.1 Pseudomonas aeruginosa strain PaLo505 chromosome, complete genome

```
product length = 269
Forward primer  1        GCAACGACACCAGCACACC  19
Template        5987260  ...................  5987278

Reverse primer  1        CGCAGATGCCATAGGAGACC  20
Template        5987528  ....................  5987509
```

>CP075784.1 Pseudomonas aeruginosa strain PaLo507 chromosome, complete genome

```
product length = 269
Forward primer  1        GCAACGACACCAGCACACC  19
Template        5466768  ...................  5466786

Reverse primer  1        CGCAGATGCCATAGGAGACC  20
Template        5467036  ....................  5467017
```

>CP075783.1 Pseudomonas aeruginosa strain PaLo508 chromosome, complete genome

```
product length = 269
Forward primer  1        GCAACGACACCAGCACACC  19
Template        5801823  ...................  5801841

Reverse primer  1        CGCAGATGCCATAGGAGACC  20
Template        5802091  ....................  5802072
```

>CP075782.1 Pseudomonas aeruginosa strain PaLo509 chromosome, complete genome

```
product length = 269
Forward primer  1        GCAACGACACCAGCACACC  19
Template        5801821  ...................  5801839

Reverse primer  1        CGCAGATGCCATAGGAGACC  20
Template        5802089  ....................  5802070
```

>CP075781.1 Pseudomonas aeruginosa strain PaLo512 chromosome, complete genome

```
product length = 269
Forward primer  1        GCAACGACACCAGCACACC  19
Template        6049643  ...................  6049661

Reverse primer  1        CGCAGATGCCATAGGAGACC  20
Template        6049911  ....................  6049892
```

>CP075780.1 Pseudomonas aeruginosa strain PaLo524 chromosome, complete genome

```
product length = 269
Forward primer  1        GCAACGACACCAGCACACC  19
Template        5657896  ...................  5657914

Reverse primer  1        CGCAGATGCCATAGGAGACC  20
Template        5658164  ....................  5658145
```

>CP075779.1 Pseudomonas aeruginosa strain PaLo526 chromosome, complete genome

```
product length = 269
Forward primer  1        GCAACGACACCAGCACACC  19
Template        5564294  ...................  5564312

Reverse primer  1        CGCAGATGCCATAGGAGACC  20
Template        5564562  ....................  5564543
```

>CP075778.1 Pseudomonas aeruginosa strain PaLo527 chromosome, complete genome

```
product length = 269
Forward primer  1        GCAACGACACCAGCACACC  19
Template        5482713  ...................  5482731

Reverse primer  1        CGCAGATGCCATAGGAGACC  20
Template        5482981  ....................  5482962
```

>CP075777.1 Pseudomonas aeruginosa strain PaLo528 chromosome, complete genome

```
product length = 269
Forward primer  1        GCAACGACACCAGCACACC  19
Template        5630779  ...................  5630797

Reverse primer  1        CGCAGATGCCATAGGAGACC  20
Template        5631047  ....................  5631028
```

>CP075776.1 Pseudomonas aeruginosa strain PaLo529 chromosome, complete genome

```
product length = 269
Forward primer  1        GCAACGACACCAGCACACC  19
Template        5573356  ...................  5573374

Reverse primer  1        CGCAGATGCCATAGGAGACC  20
Template        5573624  ....................  5573605
```

>CP075773.1 Pseudomonas aeruginosa strain PaLo530 chromosome, complete genome

```
product length = 269
Forward primer  1        GCAACGACACCAGCACACC  19
Template        5820191  ...................  5820209

Reverse primer  1        CGCAGATGCCATAGGAGACC  20
Template        5820459  ....................  5820440
```

>CP075771.1 Pseudomonas aeruginosa strain PaLo532 chromosome, complete genome

```
product length = 269
Forward primer  1        GCAACGACACCAGCACACC  19
Template        6071536  ...................  6071554

Reverse primer  1        CGCAGATGCCATAGGAGACC  20
Template        6071804  ....................  6071785
```

>CP075769.1 Pseudomonas aeruginosa strain PaLo533 chromosome, complete genome

```
product length = 269
Forward primer  1        GCAACGACACCAGCACACC  19
Template        5968572  ...................  5968590

Reverse primer  1        CGCAGATGCCATAGGAGACC  20
Template        5968840  ....................  5968821
```

>CP075768.1 Pseudomonas aeruginosa strain PaLo535 chromosome

```
product length = 269
Forward primer  1        GCAACGACACCAGCACACC  19
Template        5454223  ...................  5454241

Reverse primer  1        CGCAGATGCCATAGGAGACC  20
Template        5454491  ....................  5454472
```

>CP075767.1 Pseudomonas aeruginosa strain PaLo536 chromosome, complete genome

```
product length = 269
Forward primer  1        GCAACGACACCAGCACACC  19
Template        5600196  ...................  5600214

Reverse primer  1        CGCAGATGCCATAGGAGACC  20
Template        5600464  ....................  5600445
```

>CP075766.1 Pseudomonas aeruginosa strain PaLo538 chromosome, complete genome

```
product length = 269
Forward primer  1        GCAACGACACCAGCACACC  19
Template        6265280  ...................  6265298

Reverse primer  1        CGCAGATGCCATAGGAGACC  20
Template        6265548  ....................  6265529
```

>CP075765.1 Pseudomonas aeruginosa strain PaLo539 chromosome, complete genome

```
product length = 269
Forward primer  1        GCAACGACACCAGCACACC  19
Template        5768320  ...................  5768338

Reverse primer  1        CGCAGATGCCATAGGAGACC  20
Template        5768588  ....................  5768569
```

>CP075764.1 Pseudomonas aeruginosa strain PaLo541 chromosome, complete genome

```
product length = 269
Forward primer  1        GCAACGACACCAGCACACC  19
Template        5491398  ...................  5491416

Reverse primer  1        CGCAGATGCCATAGGAGACC  20
Template        5491666  ....................  5491647
```

>CP075763.1 Pseudomonas aeruginosa strain PaLo543 chromosome, complete genome

```
product length = 269
Forward primer  1        GCAACGACACCAGCACACC  19
Template        6197594  ...................  6197612

Reverse primer  1        CGCAGATGCCATAGGAGACC  20
Template        6197862  ....................  6197843
```

>CP075762.1 Pseudomonas aeruginosa strain PaLo544 chromosome, complete genome

```
product length = 269
Forward primer  1        GCAACGACACCAGCACACC  19
Template        5579378  ...................  5579396

Reverse primer  1        CGCAGATGCCATAGGAGACC  20
Template        5579646  ....................  5579627
```

>CP075761.1 Pseudomonas aeruginosa strain PaLo545 chromosome

```
product length = 269
Forward primer  1        GCAACGACACCAGCACACC  19
Template        6184787  ...................  6184805

Reverse primer  1        CGCAGATGCCATAGGAGACC  20
Template        6185055  ....................  6185036
```

>CP075760.1 Pseudomonas aeruginosa strain PaLo550 chromosome, complete genome

```
product length = 269
Forward primer  1        GCAACGACACCAGCACACC  19
Template        5647782  ...................  5647800

Reverse primer  1        CGCAGATGCCATAGGAGACC  20
Template        5648050  ....................  5648031
```

>CP075755.1 Pseudomonas aeruginosa strain PaLo553 chromosome, complete genome

```
product length = 269
Forward primer  1        GCAACGACACCAGCACACC  19
Template        5917915  ...................  5917933

Reverse primer  1        CGCAGATGCCATAGGAGACC  20
Template        5918183  ....................  5918164
```

>CP075754.1 Pseudomonas aeruginosa strain PaLo555 chromosome, complete genome

```
product length = 269
Forward primer  1        GCAACGACACCAGCACACC  19
Template        5521485  ...................  5521503

Reverse primer  1        CGCAGATGCCATAGGAGACC  20
Template        5521753  ....................  5521734
```

>CP075753.1 Pseudomonas aeruginosa strain PaLo556 chromosome, complete genome

```
product length = 269
Forward primer  1        GCAACGACACCAGCACACC  19
Template        5718835  ...................  5718853

Reverse primer  1        CGCAGATGCCATAGGAGACC  20
Template        5719103  ....................  5719084
```

>CP075752.1 Pseudomonas aeruginosa strain PaLo557 chromosome, complete genome

```
product length = 269
Forward primer  1        GCAACGACACCAGCACACC  19
Template        6020497  ...................  6020515

Reverse primer  1        CGCAGATGCCATAGGAGACC  20
Template        6020765  ....................  6020746
```

>CP075751.1 Pseudomonas aeruginosa strain PaLo561 chromosome, complete genome

```
product length = 269
Forward primer  1        GCAACGACACCAGCACACC  19
Template        5710622  ...................  5710640

Reverse primer  1        CGCAGATGCCATAGGAGACC  20
Template        5710890  ....................  5710871
```

>CP075750.1 Pseudomonas aeruginosa strain PaLo563 chromosome, complete genome

```
product length = 269
Forward primer  1        GCAACGACACCAGCACACC  19
Template        6105618  ...................  6105636

Reverse primer  1        CGCAGATGCCATAGGAGACC  20
Template        6105886  ....................  6105867
```

>CP075749.1 Pseudomonas aeruginosa strain PaLo564 chromosome, complete genome

```
product length = 269
Forward primer  1        GCAACGACACCAGCACACC  19
Template        5877398  ...................  5877416

Reverse primer  1        CGCAGATGCCATAGGAGACC  20
Template        5877666  ....................  5877647
```

>CP075748.1 Pseudomonas aeruginosa strain PaLo565 chromosome, complete genome

```
product length = 269
Forward primer  1        GCAACGACACCAGCACACC  19
Template        6105645  ...................  6105663

Reverse primer  1        CGCAGATGCCATAGGAGACC  20
Template        6105913  ....................  6105894
```

>CP116723.1 Pseudomonas aeruginosa strain 2872 chromosome

```
product length = 269
Forward primer  1       GCAACGACACCAGCACACC  19
Template        877149  ...................  877131

Reverse primer  1       CGCAGATGCCATAGGAGACC  20
Template        876881  ....................  876900
```

>CP116725.1 Pseudomonas aeruginosa strain 2881 chromosome, complete genome

```
product length = 269
Forward primer  1        GCAACGACACCAGCACACC  19
Template        6242773  ...................  6242791

Reverse primer  1        CGCAGATGCCATAGGAGACC  20
Template        6243041  ....................  6243022
```

>CP116722.1 Pseudomonas aeruginosa strain 2868 chromosome, complete genome

```
product length = 269
Forward primer  1        GCAACGACACCAGCACACC  19
Template        6442369  ...................  6442387

Reverse primer  1        CGCAGATGCCATAGGAGACC  20
Template        6442637  ....................  6442618
```

>CP116717.1 Pseudomonas aeruginosa strain 2857 chromosome, complete genome

```
product length = 269
Forward primer  1        GCAACGACACCAGCACACC  19
Template        6299512  ...................  6299530

Reverse primer  1        CGCAGATGCCATAGGAGACC  20
Template        6299780  ....................  6299761
```

>CP116727.1 Pseudomonas aeruginosa strain 2875 chromosome, complete genome

```
product length = 269
Forward primer  1        GCAACGACACCAGCACACC  19
Template        6342797  ...................  6342815

Reverse primer  1        CGCAGATGCCATAGGAGACC  20
Template        6343065  ....................  6343046
```

>CP116718.1 Pseudomonas aeruginosa strain 2858 chromosome, complete genome

```
product length = 269
Forward primer  1        GCAACGACACCAGCACACC  19
Template        6138207  ...................  6138225

Reverse primer  1        CGCAGATGCCATAGGAGACC  20
Template        6138475  ....................  6138456
```

>CP116720.1 Pseudomonas aeruginosa strain 2866 chromosome, complete genome

```
product length = 269
Forward primer  1        GCAACGACACCAGCACACC  19
Template        6217330  ...................  6217348

Reverse primer  1        CGCAGATGCCATAGGAGACC  20
Template        6217598  ....................  6217579
```

>CP116724.1 Pseudomonas aeruginosa strain 2880 chromosome, complete genome

```
product length = 269
Forward primer  1        GCAACGACACCAGCACACC  19
Template        6281626  ...................  6281644

Reverse primer  1        CGCAGATGCCATAGGAGACC  20
Template        6281894  ....................  6281875
```

>CP116721.1 Pseudomonas aeruginosa strain 2867 chromosome, complete genome

```
product length = 269
Forward primer  1        GCAACGACACCAGCACACC  19
Template        6253311  ...................  6253329

Reverse primer  1        CGCAGATGCCATAGGAGACC  20
Template        6253579  ....................  6253560
```

>CP116715.1 Pseudomonas aeruginosa strain 2856 chromosome, complete genome

```
product length = 269
Forward primer  1        GCAACGACACCAGCACACC  19
Template        6209751  ...................  6209769

Reverse primer  1        CGCAGATGCCATAGGAGACC  20
Template        6210019  ....................  6210000
```

>CP109931.1 Pseudomonas aeruginosa strain PALA42 chromosome, complete genome

```
product length = 269
Forward primer  1        GCAACGACACCAGCACACC  19
Template        1314486  ...................  1314468

Reverse primer  1        CGCAGATGCCATAGGAGACC  20
Template        1314218  ....................  1314237
```

>CP107275.1 Pseudomonas aeruginosa strain PALA22 chromosome, complete genome

```
product length = 269
Forward primer  1        GCAACGACACCAGCACACC  19
Template        1310397  ...................  1310379

Reverse primer  1        CGCAGATGCCATAGGAGACC  20
Template        1310129  ....................  1310148
```

>CP107064.1 Pseudomonas aeruginosa strain PALA20 chromosome, complete genome

```
product length = 269
Forward primer  1        GCAACGACACCAGCACACC  19
Template        2488335  ...................  2488317

Reverse primer  1        CGCAGATGCCATAGGAGACC  20
Template        2488067  ....................  2488086
```

>CP107029.1 Pseudomonas aeruginosa strain PALA19 chromosome, complete genome

```
product length = 269
Forward primer  1        GCAACGACACCAGCACACC  19
Template        1304269  ...................  1304251

Reverse primer  1        CGCAGATGCCATAGGAGACC  20
Template        1304001  ....................  1304020
```

>CP106742.1 Pseudomonas aeruginosa strain PALA14 chromosome, complete genome

```
product length = 269
Forward primer  1        GCAACGACACCAGCACACC  19
Template        1363512  ...................  1363494

Reverse primer  1        CGCAGATGCCATAGGAGACC  20
Template        1363244  ....................  1363263
```

>CP106681.1 Pseudomonas aeruginosa strain PALA12 chromosome, complete genome

```
product length = 269
Forward primer  1        GCAACGACACCAGCACACC  19
Template        6424827  ...................  6424809

Reverse primer  1        CGCAGATGCCATAGGAGACC  20
Template        6424559  ....................  6424578
```

>CP106784.1 Pseudomonas aeruginosa strain NY5085 chromosome, complete genome

```
product length = 269
Forward primer  1        GCAACGACACCAGCACACC  19
Template        5758161  ...................  5758179

Reverse primer  1        CGCAGATGCCATAGGAGACC  20
Template        5758429  ....................  5758410
```

>CP096913.1 Pseudomonas aeruginosa strain NY7610 chromosome, complete genome

```
product length = 269
Forward primer  1        GCAACGACACCAGCACACC  19
Template        6274081  ...................  6274099

Reverse primer  1        CGCAGATGCCATAGGAGACC  20
Template        6274349  ....................  6274330
```

>CP096912.1 Pseudomonas aeruginosa strain NY7770 chromosome, complete genome

```
product length = 269
Forward primer  1        GCAACGACACCAGCACACC  19
Template        5687785  ...................  5687803

Reverse primer  1        CGCAGATGCCATAGGAGACC  20
Template        5688053  ....................  5688034
```

>CP096909.1 Pseudomonas aeruginosa strain NY8688 chromosome, complete genome

```
product length = 269
Forward primer  1        GCAACGACACCAGCACACC  19
Template        6057416  ...................  6057434

Reverse primer  1        CGCAGATGCCATAGGAGACC  20
Template        6057684  ....................  6057665
```

>CP096822.1 Pseudomonas aeruginosa strain NY8709 chromosome, complete genome

```
product length = 269
Forward primer  1        GCAACGACACCAGCACACC  19
Template        5755131  ...................  5755149

Reverse primer  1        CGCAGATGCCATAGGAGACC  20
Template        5755399  ....................  5755380
```

>CP111030.1 Pseudomonas aeruginosa strain PALA38 chromosome, complete genome

```
product length = 269
Forward primer  1        GCAACGACACCAGCACACC  19
Template        5919770  ...................  5919788

Reverse primer  1        CGCAGATGCCATAGGAGACC  20
Template        5920038  ....................  5920019
```

>CP111032.1 Pseudomonas aeruginosa strain PALA54 chromosome, complete genome

```
product length = 269
Forward primer  1        GCAACGACACCAGCACACC  19
Template        5962944  ...................  5962962

Reverse primer  1        CGCAGATGCCATAGGAGACC  20
Template        5963212  ....................  5963193
```

>CP111034.1 Pseudomonas aeruginosa strain PALA50 chromosome, complete genome

```
product length = 269
Forward primer  1        GCAACGACACCAGCACACC  19
Template        5628337  ...................  5628355

Reverse primer  1        CGCAGATGCCATAGGAGACC  20
Template        5628605  ....................  5628586
```

>CP110353.1 Pseudomonas aeruginosa strain PALA48 chromosome, complete genome

```
product length = 269
Forward primer  1        GCAACGACACCAGCACACC  19
Template        5744859  ...................  5744877

Reverse primer  1        CGCAGATGCCATAGGAGACC  20
Template        5745127  ....................  5745108
```

>CP110351.1 Pseudomonas aeruginosa strain PALA45 chromosome, complete genome

```
product length = 269
Forward primer  1        GCAACGACACCAGCACACC  19
Template        5740381  ...................  5740399

Reverse primer  1        CGCAGATGCCATAGGAGACC  20
Template        5740649  ....................  5740630
```

>CP110350.1 Pseudomonas aeruginosa strain PALA44 chromosome, complete genome

```
product length = 269
Forward primer  1       GCAACGACACCAGCACACC  19
Template        300836  ...................  300854

Reverse primer  1       CGCAGATGCCATAGGAGACC  20
Template        301104  ....................  301085
```

>CP110349.1 Pseudomonas aeruginosa strain PALA40 chromosome, complete genome

```
product length = 269
Forward primer  1        GCAACGACACCAGCACACC  19
Template        6283984  ...................  6284002

Reverse primer  1        CGCAGATGCCATAGGAGACC  20
Template        6284252  ....................  6284233
```

>CP109920.1 Pseudomonas aeruginosa strain PALA39 chromosome, complete genome

```
product length = 269
Forward primer  1        GCAACGACACCAGCACACC  19
Template        6161490  ...................  6161508

Reverse primer  1        CGCAGATGCCATAGGAGACC  20
Template        6161758  ....................  6161739
```

>CP110348.1 Pseudomonas aeruginosa strain PALA36 chromosome, complete genome

```
product length = 269
Forward primer  1        GCAACGACACCAGCACACC  19
Template        6048810  ...................  6048828

Reverse primer  1        CGCAGATGCCATAGGAGACC  20
Template        6049078  ....................  6049059
```

>CP109919.1 Pseudomonas aeruginosa strain PALA56 chromosome, complete genome

```
product length = 269
Forward primer  1        GCAACGACACCAGCACACC  19
Template        5536334  ...................  5536352

Reverse primer  1        CGCAGATGCCATAGGAGACC  20
Template        5536602  ....................  5536583
```

>CP109918.1 Pseudomonas aeruginosa strain PALA55 chromosome, complete genome

```
product length = 269
Forward primer  1        GCAACGACACCAGCACACC  19
Template        5668723  ...................  5668741

Reverse primer  1        CGCAGATGCCATAGGAGACC  20
Template        5668991  ....................  5668972
```

>CP109856.1 Pseudomonas aeruginosa strain PALA53 chromosome, complete genome

```
product length = 269
Forward primer  1        GCAACGACACCAGCACACC  19
Template        5499154  ...................  5499172

Reverse primer  1        CGCAGATGCCATAGGAGACC  20
Template        5499422  ....................  5499403
```

>CP109851.1 Pseudomonas aeruginosa strain PALA51 chromosome, complete genome

```
product length = 269
Forward primer  1        GCAACGACACCAGCACACC  19
Template        5628555  ...................  5628573

Reverse primer  1        CGCAGATGCCATAGGAGACC  20
Template        5628823  ....................  5628804
```

>CP110346.1 Pseudomonas aeruginosa strain PALA35 chromosome, complete genome

```
product length = 269
Forward primer  1        GCAACGACACCAGCACACC  19
Template        5912914  ...................  5912932

Reverse primer  1        CGCAGATGCCATAGGAGACC  20
Template        5913182  ....................  5913163
```

>CP109849.1 Pseudomonas aeruginosa strain PALA34 chromosome, complete genome

```
product length = 269
Forward primer  1        GCAACGACACCAGCACACC  19
Template        6097672  ...................  6097690

Reverse primer  1        CGCAGATGCCATAGGAGACC  20
Template        6097940  ....................  6097921
```

>CP109845.1 Pseudomonas aeruginosa strain PALA33 chromosome, complete genome

```
product length = 269
Forward primer  1        GCAACGACACCAGCACACC  19
Template        6149179  ...................  6149197

Reverse primer  1        CGCAGATGCCATAGGAGACC  20
Template        6149447  ....................  6149428
```

>CP109844.1 Pseudomonas aeruginosa strain PALA32 chromosome, complete genome

```
product length = 269
Forward primer  1        GCAACGACACCAGCACACC  19
Template        5768049  ...................  5768067

Reverse primer  1        CGCAGATGCCATAGGAGACC  20
Template        5768317  ....................  5768298
```

>CP110345.1 Pseudomonas aeruginosa strain PALA30 chromosome, complete genome

```
product length = 269
Forward primer  1        GCAACGACACCAGCACACC  19
Template        6014484  ...................  6014502

Reverse primer  1        CGCAGATGCCATAGGAGACC  20
Template        6014752  ....................  6014733
```

>CP109843.1 Pseudomonas aeruginosa strain PALA29 chromosome, complete genome

```
product length = 269
Forward primer  1        GCAACGACACCAGCACACC  19
Template        5898134  ...................  5898152

Reverse primer  1        CGCAGATGCCATAGGAGACC  20
Template        5898402  ....................  5898383
```

>CP109835.1 Pseudomonas aeruginosa strain PALA26 chromosome, complete genome

```
product length = 269
Forward primer  1        GCAACGACACCAGCACACC  19
Template        5940845  ...................  5940863

Reverse primer  1        CGCAGATGCCATAGGAGACC  20
Template        5941113  ....................  5941094
```

>CP109834.1 Pseudomonas aeruginosa strain PALA25 chromosome, complete genome

```
product length = 269
Forward primer  1        GCAACGACACCAGCACACC  19
Template        5495991  ...................  5496009

Reverse primer  1        CGCAGATGCCATAGGAGACC  20
Template        5496259  ....................  5496240
```

>CP110344.1 Pseudomonas aeruginosa strain PALA24 chromosome, complete genome

```
product length = 269
Forward primer  1        GCAACGACACCAGCACACC  19
Template        5957927  ...................  5957945

Reverse primer  1        CGCAGATGCCATAGGAGACC  20
Template        5958195  ....................  5958176
```

>CP106745.1 Pseudomonas aeruginosa strain PALA17 chromosome, complete genome

```
product length = 269
Forward primer  1        GCAACGACACCAGCACACC  19
Template        5380241  ...................  5380259

Reverse primer  1        CGCAGATGCCATAGGAGACC  20
Template        5380509  ....................  5380490
```

>CP106744.1 Pseudomonas aeruginosa strain PALA16 chromosome, complete genome

```
product length = 269
Forward primer  1        GCAACGACACCAGCACACC  19
Template        6080346  ...................  6080364

Reverse primer  1        CGCAGATGCCATAGGAGACC  20
Template        6080614  ....................  6080595
```

>CP106743.1 Pseudomonas aeruginosa strain PALA15 chromosome, complete genome

```
product length = 269
Forward primer  1        GCAACGACACCAGCACACC  19
Template        5563625  ...................  5563643

Reverse primer  1        CGCAGATGCCATAGGAGACC  20
Template        5563893  ....................  5563874
```

>CP106682.1 Pseudomonas aeruginosa strain PALA13 chromosome, complete genome

```
product length = 269
Forward primer  1        GCAACGACACCAGCACACC  19
Template        6080224  ...................  6080242

Reverse primer  1        CGCAGATGCCATAGGAGACC  20
Template        6080492  ....................  6080473
```

>CP104870.1 Pseudomonas aeruginosa strain PALA9 chromosome, complete genome

```
product length = 269
Forward primer  1        GCAACGACACCAGCACACC  19
Template        6318117  ...................  6318135

Reverse primer  1        CGCAGATGCCATAGGAGACC  20
Template        6318385  ....................  6318366
```

>CP104869.1 Pseudomonas aeruginosa strain PALA8 chromosome, complete genome

```
product length = 269
Forward primer  1        GCAACGACACCAGCACACC  19
Template        5874165  ...................  5874183

Reverse primer  1        CGCAGATGCCATAGGAGACC  20
Template        5874433  ....................  5874414
```

>CP104867.1 Pseudomonas aeruginosa strain PALA6 chromosome, complete genome

```
product length = 269
Forward primer  1        GCAACGACACCAGCACACC  19
Template        5470001  ...................  5470019

Reverse primer  1        CGCAGATGCCATAGGAGACC  20
Template        5470269  ....................  5470250
```

>CP104866.1 Pseudomonas aeruginosa strain PALA4 chromosome, complete genome

```
product length = 269
Forward primer  1        GCAACGACACCAGCACACC  19
Template        6037931  ...................  6037949

Reverse primer  1        CGCAGATGCCATAGGAGACC  20
Template        6038199  ....................  6038180
```

>CP104865.1 Pseudomonas aeruginosa strain PALA2 chromosome, complete genome

```
product length = 269
Forward primer  1        GCAACGACACCAGCACACC  19
Template        5411565  ...................  5411583

Reverse primer  1        CGCAGATGCCATAGGAGACC  20
Template        5411833  ....................  5411814
```

>CP104254.1 Pseudomonas aeruginosa strain PALA1 chromosome, complete genome

```
product length = 269
Forward primer  1        GCAACGACACCAGCACACC  19
Template        5846464  ...................  5846482

Reverse primer  1        CGCAGATGCCATAGGAGACC  20
Template        5846732  ....................  5846713
```

>CP114761.1 Pseudomonas aeruginosa strain NF143349 chromosome, complete genome

```
product length = 269
Forward primer  1        GCAACGACACCAGCACACC  19
Template        6016453  ...................  6016471

Reverse primer  1        CGCAGATGCCATAGGAGACC  20
Template        6016721  ....................  6016702
```

>CP114374.1 Pseudomonas aeruginosa strain Jade-X chromosome, complete genome

```
product length = 269
Forward primer  1        GCAACGACACCAGCACACC  19
Template        5709223  ...................  5709241

Reverse primer  1        CGCAGATGCCATAGGAGACC  20
Template        5709491  ....................  5709472
```

>CP113974.1 Pseudomonas aeruginosa strain M6A146 chromosome, complete genome

```
product length = 269
Forward primer  1        GCAACGACACCAGCACACC  19
Template        4244337  ...................  4244355

Reverse primer  1        CGCAGATGCCATAGGAGACC  20
Template        4244605  ....................  4244586
```

>CP097560.1 Pseudomonas aeruginosa strain C4.2 chromosome, complete genome

```
product length = 270
Forward primer  1        GCAACGACACCAGCACACC  19
Template        6452541  ...................  6452559

Reverse primer  1        CGCAGATGCCATAGGAGACC  20
Template        6452810  ....................  6452791
```

>CP113230.1 Pseudomonas aeruginosa strain BIAI 160 chromosome, complete genome

```
product length = 269
Forward primer  1        GCAACGACACCAGCACACC  19
Template        2567832  ...................  2567814

Reverse primer  1        CGCAGATGCCATAGGAGACC  20
Template        2567564  ....................  2567583
```

>CP113246.1 Pseudomonas aeruginosa strain SMC4386 chromosome, complete genome

```
product length = 269
Forward primer  1       GCAACGACACCAGCACACC  19
Template        804161  ...................  804143

Reverse primer  1       CGCAGATGCCATAGGAGACC  20
Template        803893  ....................  803912
```

>CP113106.1 Pseudomonas aeruginosa strain BIAI 157 chromosome, complete genome

```
product length = 269
Forward primer  1        GCAACGACACCAGCACACC  19
Template        6622284  ...................  6622266

Reverse primer  1        CGCAGATGCCATAGGAGACC  20
Template        6622016  ....................  6622035
```

>CP097857.1 Pseudomonas sp. B111 chromosome, complete genome

```
product length = 269
Forward primer  1        GCAACGACACCAGCACACC  19
Template        4596085  ...................  4596103

Reverse primer  1        CGCAGATGCCATAGGAGACC  20
Template        4596353  ....................  4596334
```

>CP036492.1 Pseudomonas aeruginosa strain Paer4 chromosome, complete genome

```
product length = 269
Forward primer  1       GCAACGACACCAGCACACC  19
Template        792439  ...................  792421

Reverse primer  1       CGCAGATGCCATAGGAGACC  20
Template        792171  ....................  792190
```

>CP102441.2 Pseudomonas aeruginosa strain PA30 chromosome, complete genome

```
product length = 269
Forward primer  1        GCAACGACACCAGCACACC  19
Template        6115710  ...................  6115728

Reverse primer  1        CGCAGATGCCATAGGAGACC  20
Template        6115978  ....................  6115959
```

>CP083358.1 Pseudomonas aeruginosa strain KPA151 chromosome, complete genome

```
product length = 269
Forward primer  1        GCAACGACACCAGCACACC  19
Template        1529145  ...................  1529127

Reverse primer  1        CGCAGATGCCATAGGAGACC  20
Template        1528877  ....................  1528896
```

>CP083360.1 Pseudomonas aeruginosa strain KPA83 chromosome, complete genome

```
product length = 269
Forward primer  1        GCAACGACACCAGCACACC  19
Template        6200880  ...................  6200862

Reverse primer  1        CGCAGATGCCATAGGAGACC  20
Template        6200612  ....................  6200631
```

>CP083356.1 Pseudomonas aeruginosa strain KPA140 chromosome, complete genome

```
product length = 268
Forward primer  1        GCAACGACACCAGCACACC  19
Template        1569434  ...................  1569416

Reverse primer  1        CGCAGATGCCATAGGAGACC  20
Template        1569167  ....................  1569186
```

>CP083352.1 Pseudomonas aeruginosa strain KPA119 chromosome, complete genome

```
product length = 269
Forward primer  1       GCAACGACACCAGCACACC  19
Template        568004  ...................  567986

Reverse primer  1       CGCAGATGCCATAGGAGACC  20
Template        567736  ....................  567755
```

>CP107257.1 Pseudomonas aeruginosa strain 2019CK-00034 chromosome, complete genome

```
product length = 269
Forward primer  1        GCAACGACACCAGCACACC  19
Template        1755300  ...................  1755282

Reverse primer  1        CGCAGATGCCATAGGAGACC  20
Template        1755032  ....................  1755051
```

>CP083357.1 Pseudomonas aeruginosa strain KPA143 chromosome, complete genome

```
product length = 269
Forward primer  1        GCAACGACACCAGCACACC  19
Template        2142457  ...................  2142475

Reverse primer  1        CGCAGATGCCATAGGAGACC  20
Template        2142725  ....................  2142706
```

>CP083359.1 Pseudomonas aeruginosa strain KPA159 chromosome, complete genome

```
product length = 269
Forward primer  1        GCAACGACACCAGCACACC  19
Template        2033358  ...................  2033376

Reverse primer  1        CGCAGATGCCATAGGAGACC  20
Template        2033626  ....................  2033607
```

>CP083355.1 Pseudomonas aeruginosa strain KPA134 chromosome, complete genome

```
product length = 269
Forward primer  1       GCAACGACACCAGCACACC  19
Template        208769  ...................  208787

Reverse primer  1       CGCAGATGCCATAGGAGACC  20
Template        209037  ....................  209018
```

>CP083353.1 Pseudomonas aeruginosa strain KPA120 chromosome, complete genome

```
product length = 268
Forward primer  1        GCAACGACACCAGCACACC  19
Template        2041897  ...................  2041915

Reverse primer  1        CGCAGATGCCATAGGAGACC  20
Template        2042164  ....................  2042145
```

>CP083354.1 Pseudomonas aeruginosa strain KPA124 chromosome, complete genome

```
product length = 268
Forward primer  1       GCAACGACACCAGCACACC  19
Template        243864  ...................  243882

Reverse primer  1       CGCAGATGCCATAGGAGACC  20
Template        244131  ....................  244112
```

>CP104565.1 Pseudomonas aeruginosa strain HS_121 chromosome, complete genome

```
product length = 269
Forward primer  1        GCAACGACACCAGCACACC  19
Template        5870998  ...................  5871016

Reverse primer  1        CGCAGATGCCATAGGAGACC  20
Template        5871266  ....................  5871247
```

>CP104567.1 Pseudomonas aeruginosa strain HS_13 chromosome, complete genome

```
product length = 269
Forward primer  1        GCAACGACACCAGCACACC  19
Template        5837827  ...................  5837845

Reverse primer  1        CGCAGATGCCATAGGAGACC  20
Template        5838095  ....................  5838076
```

>CP107042.1 Pseudomonas aeruginosa strain GIMC5035:PA21/2013 chromosome

```
product length = 269
Forward primer  1        GCAACGACACCAGCACACC  19
Template        4737210  ...................  4737228

Reverse primer  1        CGCAGATGCCATAGGAGACC  20
Template        4737478  ....................  4737459
```

>CP086213.1 Pseudomonas aeruginosa strain Pa3 chromosome, complete genome

```
product length = 269
Forward primer  1        GCAACGACACCAGCACACC  19
Template        6385019  ...................  6385037

Reverse primer  1        CGCAGATGCCATAGGAGACC  20
Template        6385287  ....................  6385268
```

>CP104586.1 Pseudomonas aeruginosa strain WTJH6 chromosome, complete genome

```
product length = 269
Forward primer  1        GCAACGACACCAGCACACC  19
Template        3986797  ...................  3986779

Reverse primer  1        CGCAGATGCCATAGGAGACC  20
Template        3986529  ....................  3986548
```

>CP096207.1 Pseudomonas aeruginosa TBCF10839 chromosome, complete genome

```
product length = 269
Forward primer  1        GCAACGACACCAGCACACC  19
Template        6419198  ...................  6419180

Reverse primer  1        CGCAGATGCCATAGGAGACC  20
Template        6418930  ....................  6418949
```

>CP101885.1 Pseudomonas aeruginosa strain M27432 chromosome, complete genome

```
product length = 269
Forward primer  1        GCAACGACACCAGCACACC  19
Template        1092450  ...................  1092432

Reverse primer  1        CGCAGATGCCATAGGAGACC  20
Template        1092182  ....................  1092201
```

>CP094677.1 Pseudomonas aeruginosa strain Pa150 chromosome, complete genome

```
product length = 269
Forward primer  1        GCAACGACACCAGCACACC  19
Template        3879748  ...................  3879730

Reverse primer  1        CGCAGATGCCATAGGAGACC  20
Template        3879480  ....................  3879499
```

>CP079712.1 Pseudomonas aeruginosa strain PAO1-UW chromosome, complete genome

```
product length = 269
Forward primer  1        GCAACGACACCAGCACACC  19
Template        5484461  ...................  5484479

Reverse primer  1        CGCAGATGCCATAGGAGACC  20
Template        5484729  ....................  5484710
```

>CP085082.1 Pseudomonas aeruginosa strain PAO1-Holloway chromosome, complete genome

```
product length = 269
Forward primer  1        GCAACGACACCAGCACACC  19
Template        5473837  ...................  5473855

Reverse primer  1        CGCAGATGCCATAGGAGACC  20
Template        5474105  ....................  5474086
```

>CP103307.1 Pseudomonas aeruginosa strain PLL01 chromosome, complete genome

```
product length = 269
Forward primer  1        GCAACGACACCAGCACACC  19
Template        5469449  ...................  5469467

Reverse primer  1        CGCAGATGCCATAGGAGACC  20
Template        5469717  ....................  5469698
```

>CP102946.1 Pseudomonas aeruginosa strain SCAID WND1-2022 (148) chromosome, complete genome

```
product length = 269
Forward primer  1        GCAACGACACCAGCACACC  19
Template        5714647  ...................  5714665

Reverse primer  1        CGCAGATGCCATAGGAGACC  20
Template        5714915  ....................  5714896
```

>CP102944.1 Pseudomonas aeruginosa strain SCAID TCT1-2022 (325) chromosome, complete genome

```
product length = 269
Forward primer  1        GCAACGACACCAGCACACC  19
Template        5994323  ...................  5994341

Reverse primer  1        CGCAGATGCCATAGGAGACC  20
Template        5994591  ....................  5994572
```

>CP102174.1 Pseudomonas aeruginosa strain PA5083 chromosome, complete genome

```
product length = 269
Forward primer  1        GCAACGACACCAGCACACC  19
Template        6079515  ...................  6079533

Reverse primer  1        CGCAGATGCCATAGGAGACC  20
```

```
Template        6079783  ....................  6079764
```

>CP101656.1 Pseudomonas aeruginosa strain L1a chromosome, complete genome

```
product length = 269
Forward primer  1       GCAACGACACCAGCACACC  19
Template        209918  ...................  209936

Reverse primer  1       CGCAGATGCCATAGGAGACC  20
Template        210186  ....................  210167
```

>CP101912.1 Pseudomonas aeruginosa strain ATCC 27853 chromosome, complete genome

```
product length = 269
Forward primer  1        GCAACGACACCAGCACACC  19
Template        6031907  ...................  6031925

Reverse primer  1        CGCAGATGCCATAGGAGACC  20
Template        6032175  ....................  6032156
```

>CP101911.1 Pseudomonas aeruginosa strain NWRC-1223 chromosome, complete genome

```
product length = 269
Forward primer  1        GCAACGACACCAGCACACC  19
Template        5522439  ...................  5522457

Reverse primer  1        CGCAGATGCCATAGGAGACC  20
Template        5522707  ....................  5522688
```

>CP101540.1 Pseudomonas aeruginosa strain D-2 chromosome, complete genome

```
product length = 269
Forward primer  1        GCAACGACACCAGCACACC  19
Template        5571718  ...................  5571736

Reverse primer  1        CGCAGATGCCATAGGAGACC  20
Template        5571986  ....................  5571967
```

>CP094851.1 Pseudomonas aeruginosa strain R20-14 chromosome, complete genome

```
product length = 269
Forward primer  1        GCAACGACACCAGCACACC  19
Template        5548094  ...................  5548112

Reverse primer  1        CGCAGATGCCATAGGAGACC  20
Template        5548362  ....................  5548343
```

>CP100760.1 Pseudomonas aeruginosa strain AX0001 chromosome

```
product length = 269
Forward primer  1       GCAACGACACCAGCACACC  19
Template        208258  ...................  208276
```

```
Reverse primer  1       CGCAGATGCCATAGGAGACC  20
Template        208526  ....................  208507
```

>CP100761.1 Pseudomonas aeruginosa strain PA0011 chromosome

```
product length = 269
Forward primer  1       GCAACGACACCAGCACACC  19
Template        208256  ...................  208274

Reverse primer  1       CGCAGATGCCATAGGAGACC  20
Template        208524  ....................  208505
```

>CP097710.1 Pseudomonas aeruginosa strain PA-2 chromosome, complete genome

```
product length = 269
Forward primer  1        GCAACGACACCAGCACACC  19
Template        6197841  ...................  6197859

Reverse primer  1        CGCAGATGCCATAGGAGACC  20
Template        6198109  ....................  6198090
```

>CP097709.1 Pseudomonas aeruginosa strain PA-1 chromosome, complete genome

```
product length = 269
Forward primer  1        GCAACGACACCAGCACACC  19
Template        6196728  ...................  6196746

Reverse primer  1        CGCAGATGCCATAGGAGACC  20
Template        6196996  ....................  6196977
```

>CP099798.1 Pseudomonas aeruginosa strain PAO1-L chromosome, complete genome

```
product length = 269
Forward primer  1        GCAACGACACCAGCACACC  19
Template        5486095  ...................  5486113

Reverse primer  1        CGCAGATGCCATAGGAGACC  20
Template        5486363  ....................  5486344
```

>CP099797.1 Pseudomonas aeruginosa strain PAO1-N chromosome, complete genome

```
product length = 269
Forward primer  1        GCAACGACACCAGCACACC  19
Template        5427552  ...................  5427570

Reverse primer  1        CGCAGATGCCATAGGAGACC  20
Template        5427820  ....................  5427801
```

>CP096665.1 Pseudomonas aeruginosa strain PAO1_Mat-X-1 chromosome, complete genome

```
product length = 269
Forward primer  1        GCAACGACACCAGCACACC  19
Template        5224509  ...................  5224527
```

```
Reverse primer  1        CGCAGATGCCATAGGAGACC  20
Template        5224777  ....................  5224758
```

>CP096664.1 Pseudomonas aeruginosa strain PAO1_Kat-X-2 chromosome, complete genome

```
product length = 269
Forward primer  1        GCAACGACACCAGCACACC  19
Template        5125442  ...................  5125460

Reverse primer  1        CGCAGATGCCATAGGAGACC  20
Template        5125710  ....................  5125691
```

>CP091880.1 Pseudomonas aeruginosa strain US449 chromosome, complete genome

```
product length = 269
Forward primer  1        GCAACGACACCAGCACACC  19
Template        5655166  ...................  5655184

Reverse primer  1        CGCAGATGCCATAGGAGACC  20
Template        5655434  ....................  5655415
```

>CP097575.1 Pseudomonas aeruginosa strain UNC_PaerCF25 chromosome, complete genome

```
product length = 269
Forward primer  1        GCAACGACACCAGCACACC  19
Template        3868355  ...................  3868337

Reverse primer  1        CGCAGATGCCATAGGAGACC  20
Template        3868087  ....................  3868106
```

>CP097383.1 Pseudomonas aeruginosa strain L00-a chromosome, complete genome

```
product length = 269
Forward primer  1        GCAACGACACCAGCACACC  19
Template        6131971  ...................  6131989

Reverse primer  1        CGCAGATGCCATAGGAGACC  20
Template        6132239  ....................  6132220
```

>CP097256.1 Pseudomonas aeruginosa strain D5 chromosome, complete genome

```
product length = 269
Forward primer  1        GCAACGACACCAGCACACC  19
Template        5538977  ...................  5538995

Reverse primer  1        CGCAGATGCCATAGGAGACC  20
Template        5539245  ....................  5539226
```

>CP096813.1 Pseudomonas aeruginosa strain 8D chromosome, complete genome

```
product length = 269
Forward primer  1       GCAACGACACCAGCACACC  19
```

```
Template        551471  ...................  551489

Reverse primer  1       CGCAGATGCCATAGGAGACC  20
Template        551739  ....................  551720
```

>CP095772.2 Pseudomonas aeruginosa strain 34Pae23 chromosome

```
product length = 269
Forward primer  1        GCAACGACACCAGCACACC  19
Template        6050831  ...................  6050849

Reverse primer  1        CGCAGATGCCATAGGAGACC  20
Template        6051099  ....................  6051080
```

>CP095923.1 Pseudomonas aeruginosa strain AR19438 chromosome, complete genome

```
product length = 269
Forward primer  1        GCAACGACACCAGCACACC  19
Template        5739079  ...................  5739097

Reverse primer  1        CGCAGATGCCATAGGAGACC  20
Template        5739347  ....................  5739328
```

>CP095920.1 Pseudomonas aeruginosa strain AR19640 chromosome, complete genome

```
product length = 269
Forward primer  1        GCAACGACACCAGCACACC  19
Template        5579526  ...................  5579544

Reverse primer  1        CGCAGATGCCATAGGAGACC  20
Template        5579794  ....................  5579775
```

>CP095922.1 Pseudomonas aeruginosa strain AR19583 chromosome, complete genome

```
product length = 269
Forward primer  1        GCAACGACACCAGCACACC  19
Template        5570927  ...................  5570945

Reverse primer  1        CGCAGATGCCATAGGAGACC  20
Template        5571195  ....................  5571176
```

>CP095770.1 Pseudomonas aeruginosa strain 34Pae36 chromosome, complete genome

```
product length = 269
Forward primer  1        GCAACGACACCAGCACACC  19
Template        6530889  ...................  6530907

Reverse primer  1        CGCAGATGCCATAGGAGACC  20
Template        6531157  ....................  6531138
```

>CP095774.1 Pseudomonas aeruginosa strain 34Pae8 chromosome, complete genome

```
product length = 269
```

```
Forward primer  1        GCAACGACACCAGCACACC  19
Template        6044793  ...................  6044811

Reverse primer  1        CGCAGATGCCATAGGAGACC  20
Template        6045061  ....................  6045042
```

>CP090649.1 Pseudomonas aeruginosa strain PA1609 chromosome, complete genome

```
product length = 269
Forward primer  1        GCAACGACACCAGCACACC  19
Template        5918492  ...................  5918510

Reverse primer  1        CGCAGATGCCATAGGAGACC  20
Template        5918760  ....................  5918741
```

>CP090648.1 Pseudomonas aeruginosa strain PA1616 chromosome, complete genome

```
product length = 269
Forward primer  1        GCAACGACACCAGCACACC  19
Template        1750209  ...................  1750227

Reverse primer  1        CGCAGATGCCATAGGAGACC  20
Template        1750477  ....................  1750458
```

>CP090647.1 Pseudomonas aeruginosa strain PA1681 chromosome, complete genome

```
product length = 269
Forward primer  1        GCAACGACACCAGCACACC  19
Template        5708058  ...................  5708076

Reverse primer  1        CGCAGATGCCATAGGAGACC  20
Template        5708326  ....................  5708307
```

>CP050149.1 Pseudomonas aeruginosa strain CHA chromosome

```
product length = 269
Forward primer  1        GCAACGACACCAGCACACC  19
Template        5717646  ...................  5717664

Reverse primer  1        CGCAGATGCCATAGGAGACC  20
Template        5717914  ....................  5717895
```

>CP050148.1 Pseudomonas aeruginosa strain AA43 chromosome, complete genome

```
product length = 269
Forward primer  1        GCAACGACACCAGCACACC  19
Template        5481055  ...................  5481073

Reverse primer  1        CGCAGATGCCATAGGAGACC  20
Template        5481323  ....................  5481304
```

>CP064391.1 Pseudomonas aeruginosa strain PartH-Paeruginosa-RM8376 chromosome, complete genome

```
product length = 269
Forward primer  1        GCAACGACACCAGCACACC  19
Template        4434239  ...................  4434257

Reverse primer  1        CGCAGATGCCATAGGAGACC  20
Template        4434507  ....................  4434488
```

>CP063387.1 Pseudomonas aeruginosa strain ST1076_d100blood2 chromosome, complete genome

```
product length = 269
Forward primer  1        GCAACGACACCAGCACACC  19
Template        5847135  ...................  5847153

Reverse primer  1        CGCAGATGCCATAGGAGACC  20
Template        5847403  ....................  5847384
```

>CP047643.1 Pseudomonas aeruginosa CI27 chromosome, complete genome

```
product length = 269
Forward primer  1        GCAACGACACCAGCACACC  19
Template        6085808  ...................  6085826

Reverse primer  1        CGCAGATGCCATAGGAGACC  20
Template        6086076  ....................  6086057
```

>CP063396.1 Pseudomonas aeruginosa strain ST167_d26burn chromosome, complete genome

```
product length = 269
Forward primer  1        GCAACGACACCAGCACACC  19
Template        5833172  ...................  5833190

Reverse primer  1        CGCAGATGCCATAGGAGACC  20
Template        5833440  ....................  5833421
```

>CP063395.1 Pseudomonas aeruginosa strain ST167_d57blood chromosome, complete genome

```
product length = 269
Forward primer  1        GCAACGACACCAGCACACC  19
Template        5833170  ...................  5833188

Reverse primer  1        CGCAGATGCCATAGGAGACC  20
Template        5833438  ....................  5833419
```

>CP063394.1 Pseudomonas aeruginosa strain ST167_d67burn1 chromosome, complete genome

```
product length = 269
Forward primer  1        GCAACGACACCAGCACACC  19
Template        5833647  ...................  5833665

Reverse primer  1        CGCAGATGCCATAGGAGACC  20
Template        5833915  ....................  5833896
```

>CP063393.1 Pseudomonas aeruginosa strain ST167_d67burn2 chromosome, complete genome

```
product length = 269
Forward primer  1        GCAACGACACCAGCACACC  19
Template        5833158  ...................  5833176

Reverse primer  1        CGCAGATGCCATAGGAGACC  20
Template        5833426  ....................  5833407
```

>CP063392.1 Pseudomonas aeruginosa strain ST167_d68blood1 chromosome, complete genome

```
product length = 269
Forward primer  1        GCAACGACACCAGCACACC  19
Template        5833175  ...................  5833193

Reverse primer  1        CGCAGATGCCATAGGAGACC  20
Template        5833443  ....................  5833424
```

>CP063391.1 Pseudomonas aeruginosa strain ST167_d68blood2 chromosome, complete genome

```
product length = 269
Forward primer  1        GCAACGACACCAGCACACC  19
Template        5833171  ...................  5833189

Reverse primer  1        CGCAGATGCCATAGGAGACC  20
Template        5833439  ....................  5833420
```

>CP063390.1 Pseudomonas aeruginosa strain ST1076_d97burn1 chromosome, complete genome

```
product length = 269
Forward primer  1        GCAACGACACCAGCACACC  19
Template        5848204  ...................  5848222

Reverse primer  1        CGCAGATGCCATAGGAGACC  20
Template        5848472  ....................  5848453
```

>CP063389.1 Pseudomonas aeruginosa strain ST1076_d97burn2 chromosome, complete genome

```
product length = 269
Forward primer  1        GCAACGACACCAGCACACC  19
Template        5847130  ...................  5847148

Reverse primer  1        CGCAGATGCCATAGGAGACC  20
Template        5847398  ....................  5847379
```

>CP063388.1 Pseudomonas aeruginosa strain ST1076_d100blood1 chromosome, complete genome

```
product length = 269
Forward primer  1        GCAACGACACCAGCACACC  19
Template        5848189  ...................  5848207

Reverse primer  1        CGCAGATGCCATAGGAGACC  20
Template        5848457  ....................  5848438
```

>CP063386.1 Pseudomonas aeruginosa strain ST1076_d118limb1 chromosome, complete genome

```
product length = 269
Forward primer  1        GCAACGACACCAGCACACC  19
Template        5840399  ...................  5840417

Reverse primer  1        CGCAGATGCCATAGGAGACC  20
Template        5840667  ....................  5840648
```

>CP063385.1 Pseudomonas aeruginosa strain St1076_d123blood chromosome, complete genome

```
product length = 269
Forward primer  1        GCAACGACACCAGCACACC  19
Template        5848192  ...................  5848210

Reverse primer  1        CGCAGATGCCATAGGAGACC  20
Template        5848460  ....................  5848441
```

>CP093395.1 Pseudomonas aeruginosa strain PA1_NCHU chromosome, complete genome

```
product length = 269
Forward primer  1        GCAACGACACCAGCACACC  19
Template        5446644  ...................  5446626

Reverse primer  1        CGCAGATGCCATAGGAGACC  20
Template        5446376  ....................  5446395
```

>CP093024.1 Pseudomonas aeruginosa strain H06 chromosome

```
product length = 269
Forward primer  1        GCAACGACACCAGCACACC  19
Template        4364725  ...................  4364707

Reverse primer  1        CGCAGATGCCATAGGAGACC  20
Template        4364457  ....................  4364476
```

>CP093016.1 Pseudomonas aeruginosa strain H15 chromosome, complete genome

```
product length = 269
Forward primer  1        GCAACGACACCAGCACACC  19
Template        2480360  ...................  2480342

Reverse primer  1        CGCAGATGCCATAGGAGACC  20
Template        2480092  ....................  2480111
```

>CP093967.1 Pseudomonas aeruginosa strain NY4605 chromosome, complete genome

```
product length = 269
Forward primer  1        GCAACGACACCAGCACACC  19
Template        5619486  ...................  5619504

Reverse primer  1        CGCAGATGCCATAGGAGACC  20
Template        5619754  ....................  5619735
```

>CP093965.1 Pseudomonas aeruginosa strain ATCC BAA-2108 chromosome, complete genome

```
product length = 269
Forward primer  1        GCAACGACACCAGCACACC  19
Template        5582321  ...................  5582339

Reverse primer  1        CGCAGATGCCATAGGAGACC  20
Template        5582589  ....................  5582570
```

>CP093966.1 Pseudomonas aeruginosa strain ATCC BAA-2114 chromosome, complete genome

```
product length = 269
Forward primer  1        GCAACGACACCAGCACACC  19
Template        5456909  ...................  5456927

Reverse primer  1        CGCAGATGCCATAGGAGACC  20
Template        5457177  ....................  5457158
```

>CP093358.1 Pseudomonas aeruginosa strain E167 chromosome, complete genome

```
product length = 269
Forward primer  1        GCAACGACACCAGCACACC  19
Template        5982032  ...................  5982050

Reverse primer  1        CGCAGATGCCATAGGAGACC  20
Template        5982300  ....................  5982281
```

>CP093356.1 Pseudomonas aeruginosa strain E125 chromosome, complete genome

```
product length = 269
Forward primer  1        GCAACGACACCAGCACACC  19
Template        6052015  ...................  6052033

Reverse primer  1        CGCAGATGCCATAGGAGACC  20
Template        6052283  ....................  6052264
```

>CP093357.1 Pseudomonas aeruginosa strain E131 chromosome, complete genome

```
product length = 269
Forward primer  1        GCAACGACACCAGCACACC  19
Template        5816051  ...................  5816069

Reverse primer  1        CGCAGATGCCATAGGAGACC  20
Template        5816319  ....................  5816300
```

>CP093355.1 Pseudomonas aeruginosa strain E104 chromosome, complete genome

```
product length = 269
Forward primer  1        GCAACGACACCAGCACACC  19
Template        5829805  ...................  5829823

Reverse primer  1        CGCAGATGCCATAGGAGACC  20
Template        5830073  ....................  5830054
```

>CP093354.1 Pseudomonas aeruginosa strain E113 chromosome, complete genome

```
product length = 269
Forward primer  1        GCAACGACACCAGCACACC  19
Template        6019535  ...................  6019553

Reverse primer  1        CGCAGATGCCATAGGAGACC  20
Template        6019803  ....................  6019784
```

>CP093028.1 Pseudomonas aeruginosa strain H05 chromosome, complete genome

```
product length = 269
Forward primer  1        GCAACGACACCAGCACACC  19
Template        6210200  ...................  6210218

Reverse primer  1        CGCAGATGCCATAGGAGACC  20
Template        6210468  ....................  6210449
```

>CP093030.1 Pseudomonas aeruginosa strain H04 chromosome, complete genome

```
product length = 269
Forward primer  1        GCAACGACACCAGCACACC  19
Template        6134155  ...................  6134173

Reverse primer  1        CGCAGATGCCATAGGAGACC  20
Template        6134423  ....................  6134404
```

>CP093032.1 Pseudomonas aeruginosa strain H02 chromosome, complete genome

```
product length = 269
Forward primer  1        GCAACGACACCAGCACACC  19
Template        6216350  ...................  6216368

Reverse primer  1        CGCAGATGCCATAGGAGACC  20
Template        6216618  ....................  6216599
```

>CP093031.1 Pseudomonas aeruginosa strain H03 chromosome, complete genome

```
product length = 269
Forward primer  1        GCAACGACACCAGCACACC  19
Template        6075189  ...................  6075207

Reverse primer  1        CGCAGATGCCATAGGAGACC  20
Template        6075457  ....................  6075438
```

>CP093023.1 Pseudomonas aeruginosa strain H07 chromosome, complete genome

```
product length = 269
Forward primer  1        GCAACGACACCAGCACACC  19
Template        5711528  ...................  5711546

Reverse primer  1        CGCAGATGCCATAGGAGACC  20
Template        5711796  ....................  5711777
```

>CP093018.1 Pseudomonas aeruginosa strain H11 chromosome

```
product length = 269
Forward primer  1        GCAACGACACCAGCACACC  19
Template        2071019  ...................  2071037

Reverse primer  1        CGCAGATGCCATAGGAGACC  20
Template        2071287  ....................  2071268
```

>CP093015.1 Pseudomonas aeruginosa strain H16 chromosome, complete genome

```
product length = 269
Forward primer  1        GCAACGACACCAGCACACC  19
Template        5873781  ...................  5873799

Reverse primer  1        CGCAGATGCCATAGGAGACC  20
Template        5874049  ....................  5874030
```

>CP093020.1 Pseudomonas aeruginosa strain H10 chromosome, complete genome

```
product length = 269
Forward primer  1        GCAACGACACCAGCACACC  19
Template        5869428  ...................  5869446

Reverse primer  1        CGCAGATGCCATAGGAGACC  20
Template        5869696  ....................  5869677
```

>CP093012.1 Pseudomonas aeruginosa strain H20 chromosome, complete genome

```
product length = 269
Forward primer  1        GCAACGACACCAGCACACC  19
Template        5533739  ...................  5533757

Reverse primer  1        CGCAGATGCCATAGGAGACC  20
Template        5534007  ....................  5533988
```

>CP093014.1 Pseudomonas aeruginosa strain H17 chromosome, complete genome

```
product length = 269
Forward primer  1        GCAACGACACCAGCACACC  19
Template        5555266  ...................  5555284

Reverse primer  1        CGCAGATGCCATAGGAGACC  20
Template        5555534  ....................  5555515
```

>CP092629.1 Pseudomonas aeruginosa strain HU20 chromosome, complete genome

```
product length = 269
Forward primer  1        GCAACGACACCAGCACACC  19
Template        2282409  ...................  2282391

Reverse primer  1        CGCAGATGCCATAGGAGACC  20
Template        2282141  ....................  2282160
```

>CP080405.1 Pseudomonas aeruginosa strain PES_P749 chromosome, complete genome

```
product length = 269
Forward primer  1        GCAACGACACCAGCACACC  19
Template        5583621  ...................  5583639

Reverse primer  1        CGCAGATGCCATAGGAGACC  20
Template        5583889  ....................  5583870
```

>CP081148.1 Pseudomonas aeruginosa strain NDM1_2 chromosome

```
product length = 269
Forward primer  1        GCAACGACACCAGCACACC  19
Template        6143043  ...................  6143061

Reverse primer  1        CGCAGATGCCATAGGAGACC  20
Template        6143311  ....................  6143292
```

>CP092634.1 Pseudomonas aeruginosa strain LS.2c chromosome, complete genome

```
product length = 269
Forward primer  1        GCAACGACACCAGCACACC  19
Template        5555080  ...................  5555098

Reverse primer  1        CGCAGATGCCATAGGAGACC  20
Template        5555348  ....................  5555329
```

>CP092032.1 Pseudomonas aeruginosa strain ZS-PA-05 chromosome, complete genome

```
product length = 269
Forward primer  1        GCAACGACACCAGCACACC  19
Template        6401803  ...................  6401821

Reverse primer  1        CGCAGATGCCATAGGAGACC  20
Template        6402071  ....................  6402052
```

>CP089067.2 Pseudomonas aeruginosa strain UNC_PaerCF19 chromosome, complete genome

```
product length = 269
Forward primer  1        GCAACGACACCAGCACACC  19
Template        1813726  ...................  1813708

Reverse primer  1        CGCAGATGCCATAGGAGACC  20
Template        1813458  ....................  1813477
```

>CP089065.2 Pseudomonas aeruginosa strain UNC_PaerCF34 chromosome, complete genome

```
product length = 269
Forward primer  1        GCAACGACACCAGCACACC  19
Template        1524747  ...................  1524729

Reverse primer  1        CGCAGATGCCATAGGAGACC  20
Template        1524479  ....................  1524498
```

>CP069331.1 Pseudomonas aeruginosa strain R09 chromosome, complete genome

```
product length = 269
Forward primer  1        GCAACGACACCAGCACACC  19
Template        1271648  ...................  1271630

Reverse primer  1        CGCAGATGCCATAGGAGACC  20
Template        1271380  ....................  1271399
```

>CP069332.1 Pseudomonas aeruginosa strain R08 chromosome, complete genome

```
product length = 269
Forward primer  1        GCAACGACACCAGCACACC  19
Template        1271650  ...................  1271632

Reverse primer  1        CGCAGATGCCATAGGAGACC  20
Template        1271382  ....................  1271401
```

>CP069329.1 Pseudomonas aeruginosa strain R10 chromosome, complete genome

```
product length = 269
Forward primer  1        GCAACGACACCAGCACACC  19
Template        1271565  ...................  1271547

Reverse primer  1        CGCAGATGCCATAGGAGACC  20
Template        1271297  ....................  1271316
```

>CP069325.1 Pseudomonas aeruginosa strain R02 chromosome, complete genome

```
product length = 269
Forward primer  1        GCAACGACACCAGCACACC  19
Template        1271680  ...................  1271662

Reverse primer  1        CGCAGATGCCATAGGAGACC  20
Template        1271412  ....................  1271431
```

>CP089849.1 Pseudomonas aeruginosa strain PA0523 chromosome, complete genome

```
product length = 269
Forward primer  1        GCAACGACACCAGCACACC  19
Template        5667402  ...................  5667420

Reverse primer  1        CGCAGATGCCATAGGAGACC  20
Template        5667670  ....................  5667651
```

>CP089745.1 Pseudomonas aeruginosa strain Pa608 chromosome, complete genome

```
product length = 269
Forward primer  1        GCAACGACACCAGCACACC  19
Template        5558085  ...................  5558103

Reverse primer  1        CGCAGATGCCATAGGAGACC  20
Template        5558353  ....................  5558334
```

>CP089238.1 Pseudomonas aeruginosa strain JNQH-PA033 chromosome, complete genome

```
product length = 269
Forward primer  1        GCAACGACACCAGCACACC  19
Template        5609952  ...................  5609970

Reverse primer  1        CGCAGATGCCATAGGAGACC  20
Template        5610220  ....................  5610201
```

>CP089236.1 Pseudomonas aeruginosa strain JNQH-PA027 chromosome, complete genome

```
product length = 269
Forward primer  1        GCAACGACACCAGCACACC  19
Template        6048250  ...................  6048268

Reverse primer  1        CGCAGATGCCATAGGAGACC  20
Template        6048518  ....................  6048499
```

>CP087675.1 Pseudomonas aeruginosa strain P93127 chromosome, complete genome

```
product length = 269
Forward primer  1        GCAACGACACCAGCACACC  19
Template        6100790  ...................  6100808

Reverse primer  1        CGCAGATGCCATAGGAGACC  20
Template        6101058  ....................  6101039
```

>CP087674.1 Pseudomonas aeruginosa strain P4970C chromosome, complete genome

```
product length = 269
Forward primer  1        GCAACGACACCAGCACACC  19
Template        6127214  ...................  6127232

Reverse primer  1        CGCAGATGCCATAGGAGACC  20
Template        6127482  ....................  6127463
```

>CP087673.1 Pseudomonas aeruginosa strain P96131 chromosome, complete genome

```
product length = 269
Forward primer  1        GCAACGACACCAGCACACC  19
Template        6162573  ...................  6162591

Reverse primer  1        CGCAGATGCCATAGGAGACC  20
Template        6162841  ....................  6162822
```

>CP080369.1 Pseudomonas aeruginosa SG17M chromosome, complete genome

```
product length = 269
Forward primer  1        GCAACGACACCAGCACACC  19
Template        6303754  ...................  6303772

Reverse primer  1        CGCAGATGCCATAGGAGACC  20
Template        6304022  ....................  6304003
```

>CP086064.1 Pseudomonas aeruginosa strain CCBH28525 chromosome, complete genome

```
product length = 269
Forward primer  1        GCAACGACACCAGCACACC  19
Template        6148428  ...................  6148446

Reverse primer  1        CGCAGATGCCATAGGAGACC  20
Template        6148696  ....................  6148677
```

>CP086010.1 Pseudomonas aeruginosa isolate KB-PA_F19 chromosome, complete genome

```
product length = 269
Forward primer  1        GCAACGACACCAGCACACC  19
Template        5819140  ...................  5819158

Reverse primer  1        CGCAGATGCCATAGGAGACC  20
Template        5819408  ....................  5819389
```

>CP084484.1 Pseudomonas sp. PS1(2021) chromosome, complete genome

```
product length = 270
Forward primer  1        GCAACGACACCAGCACACC  19
Template        5039769  ...................  5039787

Reverse primer  1        CGCAGATGCCATAGGAGACC  20
Template        5040038  ....................  5040019
```

>CP083366.1 Pseudomonas aeruginosa strain PS1793 chromosome, complete genome

```
product length = 269
Forward primer  1        GCAACGACACCAGCACACC  19
Template        6055777  ...................  6055795

Reverse primer  1        CGCAGATGCCATAGGAGACC  20
Template        6056045  ....................  6056026
```

>CP080511.1 Pseudomonas aeruginosa strain DJ06 chromosome, complete genome

```
product length = 269
Forward primer  1        GCAACGACACCAGCACACC  19
Template        1411950  ...................  1411932

Reverse primer  1        CGCAGATGCCATAGGAGACC  20
Template        1411682  ....................  1411701
```

>CP080288.1 Pseudomonas aeruginosa strain UNC_PaerCF05 chromosome, complete genome

```
product length = 269
Forward primer  1        GCAACGACACCAGCACACC  19
Template        1512127  ...................  1512109

Reverse primer  1        CGCAGATGCCATAGGAGACC  20
Template        1511859  ....................  1511878
```

>CP080285.1 Pseudomonas aeruginosa strain UNC_PaerCF14 chromosome, complete genome

```
product length = 269
Forward primer  1        GCAACGACACCAGCACACC  19
Template        1545621  ...................  1545603

Reverse primer  1        CGCAGATGCCATAGGAGACC  20
Template        1545353  ....................  1545372
```

>CP080280.1 Pseudomonas aeruginosa strain UNC_PaerCF20 chromosome, complete genome

```
product length = 269
Forward primer  1       GCAACGACACCAGCACACC  19
Template        583161  ...................  583143

Reverse primer  1       CGCAGATGCCATAGGAGACC  20
Template        582893  ....................  582912
```

>CP080518.1 Pseudomonas aeruginosa strain YY322 chromosome, complete genome

```
product length = 269
Forward primer  1        GCAACGACACCAGCACACC  19
Template        5575015  ...................  5575033

Reverse primer  1        CGCAGATGCCATAGGAGACC  20
Template        5575283  ....................  5575264
```

>CP071947.1 Pseudomonas aeruginosa strain 2020HL-00861 chromosome, complete genome

```
product length = 269
Forward primer  1       GCAACGACACCAGCACACC  19
Template        699270  ...................  699288

Reverse primer  1       CGCAGATGCCATAGGAGACC  20
Template        699538  ....................  699519
```

>CP080289.1 Pseudomonas aeruginosa strain PA2207 chromosome, complete genome

```
product length = 269
Forward primer  1        GCAACGACACCAGCACACC  19
Template        6150262  ...................  6150280

Reverse primer  1        CGCAGATGCCATAGGAGACC  20
Template        6150530  ....................  6150511
```

>CP080287.1 Pseudomonas aeruginosa strain UNC_PaerCF11 chromosome, complete genome

```
product length = 269
Forward primer  1        GCAACGACACCAGCACACC  19
Template        3807359  ...................  3807377

Reverse primer  1        CGCAGATGCCATAGGAGACC  20
Template        3807627  ....................  3807608
```

>CP080282.1 Pseudomonas aeruginosa strain UNC_PaerCF16 chromosome, complete genome

```
product length = 269
Forward primer  1       GCAACGACACCAGCACACC  19
Template        691342  ...................  691360

Reverse primer  1       CGCAGATGCCATAGGAGACC  20
Template        691610  ....................  691591
```

>CP080281.1 Pseudomonas aeruginosa strain UNC_PaerCF17 chromosome, complete genome

```
product length = 269
Forward primer  1       GCAACGACACCAGCACACC  19
Template        680050  ...................  680068

Reverse primer  1       CGCAGATGCCATAGGAGACC  20
Template        680318  ....................  680299
```

>CP080011.1 Pseudomonas aeruginosa strain TL3773 chromosome, complete genome

```
product length = 269
Forward primer  1        GCAACGACACCAGCACACC  19
Template        2926818  ...................  2926836

Reverse primer  1        CGCAGATGCCATAGGAGACC  20
Template        2927086  ....................  2927067
```

>CP080007.1 Pseudomonas aeruginosa strain S-1 chromosome, complete genome

```
product length = 269
Forward primer  1        GCAACGACACCAGCACACC  19
Template        5521785  ...................  5521803

Reverse primer  1        CGCAGATGCCATAGGAGACC  20
Template        5522053  ....................  5522034
```

>CP053390.1 Pseudomonas aeruginosa strain TL1285 chromosome, complete genome

```
product length = 269
Forward primer  1        GCAACGACACCAGCACACC  19
Template        2028683  ...................  2028701

Reverse primer  1        CGCAGATGCCATAGGAGACC  20
Template        2028951  ....................  2028932
```

>CP075176.1 Pseudomonas aeruginosa strain PA790 chromosome, complete genome

```
product length = 269
Forward primer  1        GCAACGACACCAGCACACC  19
Template        6046805  ...................  6046823

Reverse primer  1        CGCAGATGCCATAGGAGACC  20
Template        6047073  ....................  6047054
```

>CP065947.1 Pseudomonas aeruginosa strain PAS6 chromosome, complete genome

```
product length = 269
Forward primer  1        GCAACGACACCAGCACACC  19
Template        2878017  ...................  2877999

Reverse primer  1        CGCAGATGCCATAGGAGACC  20
Template        2877749  ....................  2877768
```

>CP065948.1 Pseudomonas aeruginosa strain PAM68 chromosome, complete genome

```
product length = 269
Forward primer  1        GCAACGACACCAGCACACC  19
Template        4521450  ...................  4521468

Reverse primer  1        CGCAGATGCCATAGGAGACC  20
Template        4521718  ....................  4521699
```

>CP065374.1 Pseudomonas aeruginosa strain PAG7 chromosome, complete genome

```
product length = 269
Forward primer  1        GCAACGACACCAGCACACC  19
Template        2751079  ...................  2751097

Reverse primer  1        CGCAGATGCCATAGGAGACC  20
Template        2751347  ....................  2751328
```

>CP073080.1 Pseudomonas aeruginosa strain NDTH9845 chromosome, complete genome

```
product length = 269
Forward primer  1        GCAACGACACCAGCACACC  19
Template        6321163  ...................  6321181

Reverse primer  1        CGCAGATGCCATAGGAGACC  20
Template        6321431  ....................  6321412
```

>CP073082.1 Pseudomonas aeruginosa strain WTJH17 chromosome, complete genome

```
product length = 269
Forward primer  1        GCAACGACACCAGCACACC  19
Template        5572656  ...................  5572674

Reverse primer  1        CGCAGATGCCATAGGAGACC  20
Template        5572924  ....................  5572905
```

>CP070471.1 Pseudomonas aeruginosa strain B17932 chromosome, complete genome

```
product length = 269
Forward primer  1        GCAACGACACCAGCACACC  19
Template        1037465  ...................  1037447

Reverse primer  1        CGCAGATGCCATAGGAGACC  20
Template        1037197  ....................  1037216
```

>CP070467.1 Pseudomonas aeruginosa strain B17416 chromosome, complete genome

```
product length = 269
Forward primer  1        GCAACGACACCAGCACACC  19
Template        1276109  ...................  1276091

Reverse primer  1        CGCAGATGCCATAGGAGACC  20
Template        1275841  ....................  1275860
```

>CP071731.1 Pseudomonas aeruginosa strain LYSZa2 chromosome, complete genome

```
product length = 269
Forward primer  1        GCAACGACACCAGCACACC  19
Template        1812014  ...................  1812032

Reverse primer  1        CGCAGATGCCATAGGAGACC  20
Template        1812282  ....................  1812263
```

>CP071730.1 Pseudomonas aeruginosa strain LYSZa5 chromosome, complete genome

```
product length = 269
Forward primer  1        GCAACGACACCAGCACACC  19
Template        1812023  ...................  1812041

Reverse primer  1        CGCAGATGCCATAGGAGACC  20
Template        1812291  ....................  1812272
```

>AP024513.1 Pseudomonas aeruginosa Pa12 DNA, complete genome

```
product length = 269
Forward primer  1        GCAACGACACCAGCACACC  19
Template        5601414  ...................  5601432

Reverse primer  1        CGCAGATGCCATAGGAGACC  20
Template        5601682  ....................  5601663
```

>CP069198.1 Pseudomonas aeruginosa strain 152962 chromosome, complete genome

```
product length = 269
Forward primer  1        GCAACGACACCAGCACACC  19
Template        6241564  ...................  6241546

Reverse primer  1        CGCAGATGCCATAGGAGACC  20
Template        6241296  ....................  6241315
```

>CP024024.1 Pseudomonas aeruginosa strain PARM801 chromosome, complete genome

```
product length = 269
Forward primer  1        GCAACGACACCAGCACACC  19
Template        4113105  ...................  4113123

Reverse primer  1        CGCAGATGCCATAGGAGACC  20
Template        4113373  ....................  4113354
```

>CP068239.1 Pseudomonas aeruginosa strain PA19-3047 chromosome, complete genome

```
product length = 269
Forward primer  1        GCAACGACACCAGCACACC  19
Template        1025506  ...................  1025488

Reverse primer  1        CGCAGATGCCATAGGAGACC  20
Template        1025238  ....................  1025257
```

>CP061699.1 Pseudomonas aeruginosa strain LYSZa7 chromosome, complete genome

```
product length = 269
Forward primer  1        GCAACGACACCAGCACACC  19
Template        1070395  ...................  1070377

Reverse primer  1        CGCAGATGCCATAGGAGACC  20
Template        1070127  ....................  1070146
```

>CP060392.1 Pseudomonas aeruginosa strain 1903031130 chromosome, complete genome

```
product length = 269
Forward primer  1        GCAACGACACCAGCACACC  19
Template        6082661  ...................  6082679

Reverse primer  1        CGCAGATGCCATAGGAGACC  20
Template        6082929  ....................  6082910
```

>CP054845.1 Pseudomonas aeruginosa strain SE5429 chromosome, complete genome

```
product length = 269
Forward primer  1        GCAACGACACCAGCACACC  19
Template        6122291  ...................  6122309

Reverse primer  1        CGCAGATGCCATAGGAGACC  20
Template        6122559  ....................  6122540
```

>CP054844.1 Pseudomonas aeruginosa strain SE5357 chromosome, complete genome

```
product length = 269
Forward primer  1        GCAACGACACCAGCACACC  19
Template        6163064  ...................  6163082

Reverse primer  1        CGCAGATGCCATAGGAGACC  20
Template        6163332  ....................  6163313
```

>CP054843.1 Pseudomonas aeruginosa strain SE5352 chromosome, complete genome

```
product length = 269
Forward primer  1        GCAACGACACCAGCACACC  19
Template        6082683  ...................  6082701

Reverse primer  1        CGCAGATGCCATAGGAGACC  20
Template        6082951  ....................  6082932
```

>CP054581.1 Pseudomonas aeruginosa strain YTSEY8 chromosome, complete genome

```
product length = 269
Forward primer  1        GCAACGACACCAGCACACC  19
Template        6347043  ...................  6347061

Reverse primer  1        CGCAGATGCCATAGGAGACC  20
Template        6347311  ....................  6347292
```

>CP065848.1 Pseudomonas aeruginosa strain CMC-097 chromosome, complete genome

```
product length = 269
Forward primer  1        GCAACGACACCAGCACACC  19
Template        1534745  ...................  1534727

Reverse primer  1        CGCAGATGCCATAGGAGACC  20
Template        1534477  ....................  1534496
```

>CP060241.1 Pseudomonas aeruginosa strain C-I-1 chromosome, complete genome

```
product length = 269
Forward primer  1       GCAACGACACCAGCACACC  19
Template        804053  ...................  804035

Reverse primer  1       CGCAGATGCCATAGGAGACC  20
Template        803785  ....................  803804
```

>CP054623.1 Pseudomonas aeruginosa strain DL201330 chromosome, complete genome

```
product length = 269
Forward primer  1        GCAACGACACCAGCACACC  19
Template        5728159  ...................  5728177

Reverse primer  1        CGCAGATGCCATAGGAGACC  20
Template        5728427  ....................  5728408
```

>CP065966.1 Pseudomonas aeruginosa strain FDAARGOS_1041 chromosome, complete genome

```
product length = 269
Forward primer  1        GCAACGACACCAGCACACC  19
Template        2780671  ...................  2780689

Reverse primer  1        CGCAGATGCCATAGGAGACC  20
Template        2780939  ....................  2780920
```

>CP027857.1 Pseudomonas aeruginosa strain MPAO1 chromosome, complete genome

```
product length = 269
Forward primer  1        GCAACGACACCAGCACACC  19
Template        5484595  ...................  5484613

Reverse primer  1        CGCAGATGCCATAGGAGACC  20
Template        5484863  ....................  5484844
```

>CP065867.1 Pseudomonas aeruginosa strain TJ2014-049 chromosome, complete genome

```
product length = 269
Forward primer  1        GCAACGACACCAGCACACC  19
Template        5861086  ...................  5861104

Reverse primer  1        CGCAGATGCCATAGGAGACC  20
Template        5861354  ....................  5861335
```

>CP065866.1 Pseudomonas aeruginosa strain TJ2019-017 chromosome, complete genome

```
product length = 269
Forward primer  1        GCAACGACACCAGCACACC  19
Template        5655725  ...................  5655743

Reverse primer  1        CGCAGATGCCATAGGAGACC  20
Template        5655993  ....................  5655974
```

>CP065417.1 Pseudomonas aeruginosa isolate P23 chromosome, complete genome

```
product length = 269
Forward primer  1        GCAACGACACCAGCACACC  19
Template        6110884  ...................  6110902

Reverse primer  1        CGCAGATGCCATAGGAGACC  20
Template        6111152  ....................  6111133
```

>CP065412.1 Pseudomonas aeruginosa isolate P33 chromosome, complete genome

```
product length = 269
Forward primer  1        GCAACGACACCAGCACACC  19
Template        6153763  ...................  6153781

Reverse primer  1        CGCAGATGCCATAGGAGACC  20
Template        6154031  ....................  6154012
```

>CP046402.2 Pseudomonas aeruginosa strain SE5331 chromosome, complete genome

```
product length = 269
Forward primer  1        GCAACGACACCAGCACACC  19
Template        6234892  ...................  6234910

Reverse primer  1        CGCAGATGCCATAGGAGACC  20
Template        6235160  ....................  6235141
```

>CP046406.2 Pseudomonas aeruginosa strain SE5458 chromosome, complete genome

```
product length = 269
Forward primer  1        GCAACGACACCAGCACACC  19
Template        6259456  ...................  6259474

Reverse primer  1        CGCAGATGCCATAGGAGACC  20
Template        6259724  ....................  6259705
```

>CP045552.2 Pseudomonas aeruginosa strain YT12746 chromosome, complete genome

```
product length = 269
Forward primer  1        GCAACGACACCAGCACACC  19
Template        6040660  ...................  6040678

Reverse primer  1        CGCAGATGCCATAGGAGACC  20
Template        6040928  ....................  6040909
```

>CP060243.1 Pseudomonas aeruginosa strain A-I-1 chromosome, complete genome

```
product length = 269
Forward primer  1        GCAACGACACCAGCACACC  19
Template        6301338  ...................  6301356

Reverse primer  1        CGCAGATGCCATAGGAGACC  20
Template        6301606  ....................  6301587
```

>CP060242.1 Pseudomonas aeruginosa strain B-I-1 chromosome, complete genome

```
product length = 269
Forward primer  1        GCAACGACACCAGCACACC  19
Template        5426029  ...................  5426047

Reverse primer  1        CGCAGATGCCATAGGAGACC  20
Template        5426297  ....................  5426278
```

>CP060240.1 Pseudomonas aeruginosa strain G-I-1 chromosome, complete genome

```
product length = 269
Forward primer  1        GCAACGACACCAGCACACC  19
Template        5797729  ...................  5797747

Reverse primer  1        CGCAGATGCCATAGGAGACC  20
Template        5797997  ....................  5797978
```

>LR898867.1 Pseudomonas aeruginosa isolate MINF_3A-sc-2280432 genome assembly, chromosome: 1

```
product length = 269
Forward primer  1        GCAACGACACCAGCACACC  19
Template        6107019  ...................  6107037

Reverse primer  1        CGCAGATGCCATAGGAGACC  20
Template        6107287  ....................  6107268
```

>LR890619.1 Pseudomonas aeruginosa isolate MINF_7A-sc-2280434 genome assembly, chromosome: 1

```
product length = 269
Forward primer  1        GCAACGACACCAGCACACC  19
Template        5468855  ...................  5468873

Reverse primer  1        CGCAGATGCCATAGGAGACC  20
```

```
Template        5469123  ....................  5469104
```

>CP059063.1 Pseudomonas aeruginosa strain GIMC5034:PA52Ts32 chromosome

```
product length = 269
Forward primer  1       GCAACGACACCAGCACACC  19
Template        968235  ...................  968217

Reverse primer  1       CGCAGATGCCATAGGAGACC  20
Template        967967  ....................  967986
```

>CP058333.1 Pseudomonas aeruginosa strain ACR20 chromosome, complete genome

```
product length = 269
Forward primer  1        GCAACGACACCAGCACACC  19
Template        6442901  ...................  6442883

Reverse primer  1        CGCAGATGCCATAGGAGACC  20
Template        6442633  ....................  6442652
```

>CP058332.1 Pseudomonas aeruginosa strain B18 chromosome, complete genome

```
product length = 269
Forward primer  1        GCAACGACACCAGCACACC  19
Template        2507569  ...................  2507551

Reverse primer  1        CGCAGATGCCATAGGAGACC  20
Template        2507301  ....................  2507320
```

>CP058323.1 Pseudomonas aeruginosa strain LV chromosome

```
product length = 269
Forward primer  1       GCAACGACACCAGCACACC  19
Template        460519  ...................  460501

Reverse primer  1       CGCAGATGCCATAGGAGACC  20
Template        460251  ....................  460270
```

>CP056774.1 Pseudomonas aeruginosa strain CDN129 chromosome, complete genome

```
product length = 269
Forward primer  1        GCAACGACACCAGCACACC  19
Template        6563929  ...................  6563911

Reverse primer  1        CGCAGATGCCATAGGAGACC  20
Template        6563661  ....................  6563680
```

>CP056090.1 Pseudomonas aeruginosa strain PABCH42 chromosome

```
product length = 269
Forward primer  1        GCAACGACACCAGCACACC  19
Template        2160326  ...................  2160308
```

```
Reverse primer  1        CGCAGATGCCATAGGAGACC  20
Template        2160058  ....................  2160077
```

>CP056089.1 Pseudomonas aeruginosa strain PABCH46 chromosome

```
product length = 269
Forward primer  1        GCAACGACACCAGCACACC  19
Template        1076627  ...................  1076609

Reverse primer  1        CGCAGATGCCATAGGAGACC  20
Template        1076359  ....................  1076378
```

>CP060703.1 Pseudomonas aeruginosa strain NRD619 chromosome, complete genome

```
product length = 269
Forward primer  1        GCAACGACACCAGCACACC  19
Template        5643594  ...................  5643612

Reverse primer  1        CGCAGATGCCATAGGAGACC  20
Template        5643862  ....................  5643843
```

>CP060086.1 Pseudomonas aeruginosa strain JNQH-PA57 chromosome, complete genome

```
product length = 269
Forward primer  1        GCAACGACACCAGCACACC  19
Template        5935374  ...................  5935392

Reverse primer  1        CGCAGATGCCATAGGAGACC  20
Template        5935642  ....................  5935623
```

>CP058331.1 Pseudomonas aeruginosa strain ACR22 chromosome, complete genome

```
product length = 269
Forward primer  1        GCAACGACACCAGCACACC  19
Template        6256523  ...................  6256541

Reverse primer  1        CGCAGATGCCATAGGAGACC  20
Template        6256791  ....................  6256772
```

>CP053119.1 Pseudomonas aeruginosa strain A17CT chromosome

```
product length = 269
Forward primer  1        GCAACGACACCAGCACACC  19
Template        5474392  ...................  5474410

Reverse primer  1        CGCAGATGCCATAGGAGACC  20
Template        5474660  ....................  5474641
```

>CP053118.1 Pseudomonas aeruginosa strain A17PBS chromosome

```
product length = 269
Forward primer  1        GCAACGACACCAGCACACC  19
Template        5474013  ...................  5474031
```

```
Reverse primer  1        CGCAGATGCCATAGGAGACC  20
Template        5474281  ....................  5474262
```

>CP053117.1 Pseudomonas aeruginosa strain P16CT chromosome

```
product length = 269
Forward primer  1        GCAACGACACCAGCACACC  19
Template        5474112  ...................  5474130

Reverse primer  1        CGCAGATGCCATAGGAGACC  20
Template        5474380  ....................  5474361
```

>CP053116.1 Pseudomonas aeruginosa strain P16PBS chromosome

```
product length = 269
Forward primer  1        GCAACGACACCAGCACACC  19
Template        5474033  ...................  5474051

Reverse primer  1        CGCAGATGCCATAGGAGACC  20
Template        5474301  ....................  5474282
```

>CP053115.1 Pseudomonas aeruginosa strain P4CT chromosome

```
product length = 269
Forward primer  1        GCAACGACACCAGCACACC  19
Template        5474043  ...................  5474061

Reverse primer  1        CGCAGATGCCATAGGAGACC  20
Template        5474311  ....................  5474292
```

>CP053114.1 Pseudomonas aeruginosa strain P4PBS chromosome

```
product length = 269
Forward primer  1        GCAACGACACCAGCACACC  19
Template        5474085  ...................  5474103

Reverse primer  1        CGCAGATGCCATAGGAGACC  20
Template        5474353  ....................  5474334
```

>CP053113.1 Pseudomonas aeruginosa strain PAO1CT chromosome

```
product length = 269
Forward primer  1        GCAACGACACCAGCACACC  19
Template        5474090  ...................  5474108

Reverse primer  1        CGCAGATGCCATAGGAGACC  20
Template        5474358  ....................  5474339
```

>CP053112.1 Pseudomonas aeruginosa strain PAO1PBS chromosome

```
product length = 269
Forward primer  1        GCAACGACACCAGCACACC  19
```

```
Template        5474046  ...................  5474064

Reverse primer  1        CGCAGATGCCATAGGAGACC  20
Template        5474314  ....................  5474295
```

>CP053111.1 Pseudomonas aeruginosa strain UAB2CT chromosome

```
product length = 269
Forward primer  1        GCAACGACACCAGCACACC  19
Template        5474003  ...................  5474021

Reverse primer  1        CGCAGATGCCATAGGAGACC  20
Template        5474271  ....................  5474252
```

>CP053110.1 Pseudomonas aeruginosa strain UAB2PBS chromosome

```
product length = 269
Forward primer  1        GCAACGACACCAGCACACC  19
Template        5474029  ...................  5474047

Reverse primer  1        CGCAGATGCCATAGGAGACC  20
Template        5474297  ....................  5474278
```

>CP046405.1 Pseudomonas aeruginosa strain SE5443 chromosome, complete genome

```
product length = 269
Forward primer  1        GCAACGACACCAGCACACC  19
Template        5787628  ...................  5787646

Reverse primer  1        CGCAGATGCCATAGGAGACC  20
Template        5787896  ....................  5787877
```

>CP046404.1 Pseudomonas aeruginosa strain SE5416 chromosome, complete genome

```
product length = 269
Forward primer  1        GCAACGACACCAGCACACC  19
Template        5989686  ...................  5989704

Reverse primer  1        CGCAGATGCCATAGGAGACC  20
Template        5989954  ....................  5989935
```

>CP046403.1 Pseudomonas aeruginosa strain SE5369 chromosome, complete genome

```
product length = 269
Forward primer  1        GCAACGACACCAGCACACC  19
Template        6353385  ...................  6353403

Reverse primer  1        CGCAGATGCCATAGGAGACC  20
Template        6353653  ....................  6353634
```

>CP056100.1 Pseudomonas aeruginosa strain PABCH01 chromosome

```
product length = 269
```

```
Forward primer  1        GCAACGACACCAGCACACC  19
Template        6197195  ...................  6197213

Reverse primer  1        CGCAGATGCCATAGGAGACC  20
Template        6197463  ....................  6197444
```

>CP056095.1 Pseudomonas aeruginosa strain PABCH09 chromosome

```
product length = 269
Forward primer  1        GCAACGACACCAGCACACC  19
Template        5706344  ...................  5706362

Reverse primer  1        CGCAGATGCCATAGGAGACC  20
Template        5706612  ....................  5706593
```

>CP056092.1 Pseudomonas aeruginosa strain PABCH14 chromosome

```
product length = 269
Forward primer  1        GCAACGACACCAGCACACC  19
Template        6292691  ...................  6292709

Reverse primer  1        CGCAGATGCCATAGGAGACC  20
Template        6292959  ....................  6292940
```

>CP056101.1 Pseudomonas aeruginosa strain PABCH45 chromosome

```
product length = 269
Forward primer  1        GCAACGACACCAGCACACC  19
Template        5366412  ...................  5366430

Reverse primer  1        CGCAGATGCCATAGGAGACC  20
Template        5366680  ....................  5366661
```

>CP056098.1 Pseudomonas aeruginosa strain PABCH05 chromosome

```
product length = 269
Forward primer  1        GCAACGACACCAGCACACC  19
Template        5471778  ...................  5471796

Reverse primer  1        CGCAGATGCCATAGGAGACC  20
Template        5472046  ....................  5472027
```

>CP056094.1 Pseudomonas aeruginosa strain PABCH10 chromosome

```
product length = 269
Forward primer  1        GCAACGACACCAGCACACC  19
Template        6211900  ...................  6211918

Reverse primer  1        CGCAGATGCCATAGGAGACC  20
Template        6212168  ....................  6212149
```

>CP056093.1 Pseudomonas aeruginosa strain PABCH13 chromosome

```
product length = 269
Forward primer  1        GCAACGACACCAGCACACC  19
Template        5668541  ...................  5668559

Reverse primer  1        CGCAGATGCCATAGGAGACC  20
Template        5668809  ....................  5668790
```

>CP054591.1 Pseudomonas aeruginosa strain CDN118 chromosome, complete genome

```
product length = 269
Forward primer  1       GCAACGACACCAGCACACC  19
Template        661644  ...................  661626

Reverse primer  1       CGCAGATGCCATAGGAGACC  20
Template        661376  ....................  661395
```

>CP050326.1 Pseudomonas aeruginosa strain DVT423 chromosome, complete genome

```
product length = 269
Forward primer  1        GCAACGACACCAGCACACC  19
Template        1336293  ...................  1336275

Reverse primer  1        CGCAGATGCCATAGGAGACC  20
Template        1336025  ....................  1336044
```

>CP050322.1 Pseudomonas aeruginosa strain DVT729 chromosome, complete genome

```
product length = 269
Forward primer  1       GCAACGACACCAGCACACC  19
Template        625772  ...................  625754

Reverse primer  1       CGCAGATGCCATAGGAGACC  20
Template        625504  ....................  625523
```

>CP054572.1 Pseudomonas sp. FDAARGOS_761 chromosome, complete genome

```
product length = 269
Forward primer  1        GCAACGACACCAGCACACC  19
Template        6374005  ...................  6373987

Reverse primer  1        CGCAGATGCCATAGGAGACC  20
Template        6373737  ....................  6373756
```

>CP053917.1 Pseudomonas aeruginosa strain PSE6684 chromosome, complete genome

```
product length = 269
Forward primer  1        GCAACGACACCAGCACACC  19
Template        3748079  ...................  3748061

Reverse primer  1        CGCAGATGCCATAGGAGACC  20
Template        3747811  ....................  3747830
```

>CP051770.1 Pseudomonas aeruginosa strain GIMC5021:PA52Ts17, complete sequence

```
product length = 269
Forward primer  1       GCAACGACACCAGCACACC  19
Template        963856  ...................  963838

Reverse primer  1       CGCAGATGCCATAGGAGACC  20
Template        963588  ....................  963607
```

>CP051768.1 Pseudomonas aeruginosa strain GIMC5020:PA52Ts2, complete sequence

```
product length = 269
Forward primer  1       GCAACGACACCAGCACACC  19
Template        966829  ...................  966811

Reverse primer  1       CGCAGATGCCATAGGAGACC  20
Template        966561  ....................  966580
```

>CP051766.1 Pseudomonas aeruginosa strain GIMC5019:PA52Ts1, complete sequence

```
product length = 269
Forward primer  1       GCAACGACACCAGCACACC  19
Template        966847  ...................  966829

Reverse primer  1       CGCAGATGCCATAGGAGACC  20
Template        966579  ....................  966598
```

>CP034908.2 Pseudomonas aeruginosa strain PA0750 chromosome, complete genome

```
product length = 269
Forward primer  1        GCAACGACACCAGCACACC  19
Template        5452427  ...................  5452445

Reverse primer  1        CGCAGATGCCATAGGAGACC  20
Template        5452695  ....................  5452676
```

>CP050335.1 Pseudomonas aeruginosa strain DVT401 chromosome, complete genome

```
product length = 269
Forward primer  1        GCAACGACACCAGCACACC  19
Template        5762196  ...................  5762214

Reverse primer  1        CGCAGATGCCATAGGAGACC  20
Template        5762464  ....................  5762445
```

>CP050334.1 Pseudomonas aeruginosa strain DVT410 chromosome, complete genome

```
product length = 269
Forward primer  1        GCAACGACACCAGCACACC  19
Template        5432352  ...................  5432370

Reverse primer  1        CGCAGATGCCATAGGAGACC  20
Template        5432620  ....................  5432601
```

>CP050333.1 Pseudomonas aeruginosa strain DVT412 chromosome, complete genome

```
product length = 269
Forward primer  1        GCAACGACACCAGCACACC  19
Template        5595901  ...................  5595919

Reverse primer  1        CGCAGATGCCATAGGAGACC  20
Template        5596169  ....................  5596150
```

>CP050332.1 Pseudomonas aeruginosa strain DVT413 chromosome, complete genome

```
product length = 269
Forward primer  1        GCAACGACACCAGCACACC  19
Template        6137177  ...................  6137195

Reverse primer  1        CGCAGATGCCATAGGAGACC  20
Template        6137445  ....................  6137426
```

>CP050331.1 Pseudomonas aeruginosa strain DVT414 chromosome, complete genome

```
product length = 269
Forward primer  1        GCAACGACACCAGCACACC  19
Template        5723874  ...................  5723892

Reverse primer  1        CGCAGATGCCATAGGAGACC  20
Template        5724142  ....................  5724123
```

>CP050330.1 Pseudomonas aeruginosa strain DVT779 chromosome, complete genome

```
product length = 269
Forward primer  1        GCAACGACACCAGCACACC  19
Template        5593345  ...................  5593363

Reverse primer  1        CGCAGATGCCATAGGAGACC  20
Template        5593613  ....................  5593594
```

>CP050329.1 Pseudomonas aeruginosa strain DVT417 chromosome, complete genome

```
product length = 269
Forward primer  1        GCAACGACACCAGCACACC  19
Template        5495955  ...................  5495973

Reverse primer  1        CGCAGATGCCATAGGAGACC  20
Template        5496223  ....................  5496204
```

>CP050328.1 Pseudomonas aeruginosa strain DVT419 chromosome, complete genome

```
product length = 269
Forward primer  1        GCAACGACACCAGCACACC  19
Template        5256023  ...................  5256041

Reverse primer  1        CGCAGATGCCATAGGAGACC  20
Template        5256291  ....................  5256272
```

>CP050327.1 Pseudomonas aeruginosa strain DVT421 chromosome, complete genome

```
product length = 269
Forward primer  1        GCAACGACACCAGCACACC  19
Template        5270819  ...................  5270837

Reverse primer  1        CGCAGATGCCATAGGAGACC  20
Template        5271087  ....................  5271068
```

>CP050325.1 Pseudomonas aeruginosa strain DVT425 chromosome, complete genome

```
product length = 269
Forward primer  1        GCAACGACACCAGCACACC  19
Template        5708883  ...................  5708901

Reverse primer  1        CGCAGATGCCATAGGAGACC  20
Template        5709151  ....................  5709132
```

>CP050324.1 Pseudomonas aeruginosa strain DVT427 chromosome, complete genome

```
product length = 269
Forward primer  1        GCAACGACACCAGCACACC  19
Template        5649603  ...................  5649621

Reverse primer  1        CGCAGATGCCATAGGAGACC  20
Template        5649871  ....................  5649852
```

>CP054473.1 Pseudomonas aeruginosa strain PAAK095 chromosome, complete genome

```
product length = 269
Forward primer  1        GCAACGACACCAGCACACC  19
Template        5972563  ...................  5972581

Reverse primer  1        CGCAGATGCCATAGGAGACC  20
Template        5972831  ....................  5972812
```

>CP054472.1 Pseudomonas aeruginosa strain PAAK088 chromosome, complete genome

```
product length = 269
Forward primer  1        GCAACGACACCAGCACACC  19
Template        5591179  ...................  5591197

Reverse primer  1        CGCAGATGCCATAGGAGACC  20
Template        5591447  ....................  5591428
```

>CP050052.1 Pseudomonas aeruginosa strain LIUYANG-E chromosome, complete genome

```
product length = 269
Forward primer  1        GCAACGACACCAGCACACC  19
Template        5484264  ...................  5484282

Reverse primer  1        CGCAGATGCCATAGGAGACC  20
Template        5484532  ....................  5484513
```

>CP050054.1 Pseudomonas aeruginosa strain LIUYANG-A chromosome, complete genome

```
product length = 269
Forward primer  1        GCAACGACACCAGCACACC  19
Template        5484242  ...................  5484260

Reverse primer  1        CGCAGATGCCATAGGAGACC  20
Template        5484510  ....................  5484491
```

>CP050053.1 Pseudomonas aeruginosa strain LIUYANG-C chromosome, complete genome

```
product length = 269
Forward primer  1        GCAACGACACCAGCACACC  19
Template        5475865  ...................  5475883

Reverse primer  1        CGCAGATGCCATAGGAGACC  20
Template        5476133  ....................  5476114
```

>CP053922.1 Pseudomonas aeruginosa strain YD001 chromosome, complete genome

```
product length = 269
Forward primer  1        GCAACGACACCAGCACACC  19
Template        5704345  ...................  5704363

Reverse primer  1        CGCAGATGCCATAGGAGACC  20
Template        5704613  ....................  5704594
```

>CP053706.1 Pseudomonas aeruginosa strain PAC1 chromosome, complete genome

```
product length = 269
Forward primer  1        GCAACGACACCAGCACACC  19
Template        4685065  ...................  4685083

Reverse primer  1        CGCAGATGCCATAGGAGACC  20
Template        4685333  ....................  4685314
```

>CP053705.1 Pseudomonas aeruginosa strain PAC6 chromosome, complete genome

```
product length = 269
Forward primer  1        GCAACGACACCAGCACACC  19
Template        5731460  ...................  5731478

Reverse primer  1        CGCAGATGCCATAGGAGACC  20
Template        5731728  ....................  5731709
```

>CP053686.1 Pseudomonas aeruginosa strain SCAID PHRX1-2019 chromosome

```
product length = 269
Forward primer  1        GCAACGACACCAGCACACC  19
Template        2486050  ...................  2486068

Reverse primer  1        CGCAGATGCCATAGGAGACC  20
Template        2486318  ....................  2486299
```

>CP044533.1 Pseudomonas aeruginosa strain Ps33 chromosome

```
product length = 269
Forward primer  1        GCAACGACACCAGCACACC  19
Template        4261900  ...................  4261918

Reverse primer  1        CGCAGATGCCATAGGAGACC  20
Template        4262168  ....................  4262149
```

>CP053028.1 Pseudomonas aeruginosa PAO1 chromosome, complete genome

```
product length = 269
Forward primer  1        GCAACGACACCAGCACACC  19
Template        5427512  ...................  5427530

Reverse primer  1        CGCAGATGCCATAGGAGACC  20
Template        5427780  ....................  5427761
```

>CP052759.1 Pseudomonas aeruginosa strain LYT4 chromosome, complete genome

```
product length = 269
Forward primer  1        GCAACGACACCAGCACACC  19
Template        5884395  ...................  5884413

Reverse primer  1        CGCAGATGCCATAGGAGACC  20
Template        5884663  ....................  5884644
```

>CP051547.1 Pseudomonas aeruginosa strain AA2 chromosome, complete genome

```
product length = 269
Forward primer  1        GCAACGACACCAGCACACC  19
Template        5491917  ...................  5491935

Reverse primer  1        CGCAGATGCCATAGGAGACC  20
Template        5492185  ....................  5492166
```

>CP048791.1 Pseudomonas aeruginosa strain VIT PC9 chromosome, complete genome

```
product length = 268
Forward primer  1        GCAACGACACCAGCACACC  19
Template        1563515  ...................  1563497

Reverse primer  1        CGCAGATGCCATAGGAGACC  20
Template        1563248  ....................  1563267
```

>CP045002.1 Pseudomonas aeruginosa strain PAG5 chromosome, complete genome

```
product length = 269
Forward primer  1        GCAACGACACCAGCACACC  19
Template        5884224  ...................  5884242

Reverse primer  1        CGCAGATGCCATAGGAGACC  20
Template        5884492  ....................  5884473
```

>CP021380.2 Pseudomonas aeruginosa strain CCBH4851 genome

```
product length = 269
Forward primer  1        GCAACGACACCAGCACACC  19
Template        6022075  ...................  6022093

Reverse primer  1        CGCAGATGCCATAGGAGACC  20
Template        6022343  ....................  6022324
```

>CP049161.1 Pseudomonas aeruginosa strain MS14403 chromosome, complete genome

```
product length = 269
Forward primer  1        GCAACGACACCAGCACACC  19
Template        5483590  ...................  5483608

Reverse primer  1        CGCAGATGCCATAGGAGACC  20
Template        5483858  ....................  5483839
```

>LR739071.1 Pseudomonas aeruginosa strain C7-25 genome assembly, chromosome: C7-25

```
product length = 269
Forward primer  1        GCAACGACACCAGCACACC  19
Template        5661775  ...................  5661793

Reverse primer  1        CGCAGATGCCATAGGAGACC  20
Template        5662043  ....................  5662024
```

>LR739069.1 Pseudomonas aeruginosa strain PcyII-40 genome assembly, chromosome: PcyII-40

```
product length = 269
Forward primer  1        GCAACGACACCAGCACACC  19
Template        5984065  ...................  5984083

Reverse primer  1        CGCAGATGCCATAGGAGACC  20
Template        5984333  ....................  5984314
```

>CP047697.1 Pseudomonas aeruginosa strain RD1-3 chromosome, complete genome

```
product length = 269
Forward primer  1        GCAACGACACCAGCACACC  19
Template        1296060  ...................  1296042

Reverse primer  1        CGCAGATGCCATAGGAGACC  20
Template        1295792  ....................  1295811
```

>CP047592.1 Pseudomonas aeruginosa strain INP-43 chromosome, complete genome

```
product length = 269
Forward primer  1       GCAACGACACCAGCACACC  19
Template        385263  ...................  385281

Reverse primer  1       CGCAGATGCCATAGGAGACC  20
Template        385531  ....................  385512
```

>CP028132.1 Pseudomonas aeruginosa strain YB01 chromosome, complete genome

```
product length = 269
Forward primer  1        GCAACGACACCAGCACACC  19
Template        5498219  ...................  5498237

Reverse primer  1        CGCAGATGCCATAGGAGACC  20
Template        5498487  ....................  5498468
```

>CP025056.3 Pseudomonas aeruginosa strain PB367 chromosome, complete genome

```
product length = 269
Forward primer  1        GCAACGACACCAGCACACC  19
Template        5868404  ...................  5868422

Reverse primer  1        CGCAGATGCCATAGGAGACC  20
Template        5868672  ....................  5868653
```

>CP025055.2 Pseudomonas aeruginosa strain PB350 chromosome, complete genome

```
product length = 269
Forward primer  1        GCAACGACACCAGCACACC  19
Template        5868404  ...................  5868422

Reverse primer  1        CGCAGATGCCATAGGAGACC  20
Template        5868672  ....................  5868653
```

>CP047069.1 Pseudomonas aeruginosa strain Environ_1 chromosome

```
product length = 269
Forward primer  1        GCAACGACACCAGCACACC  19
Template        5339866  ...................  5339884

Reverse primer  1        CGCAGATGCCATAGGAGACC  20
Template        5340134  ....................  5340115
```

>CP047063.1 Pseudomonas aeruginosa strain delta6_4 chromosome

```
product length = 269
Forward primer  1        GCAACGACACCAGCACACC  19
Template        5474015  ...................  5474033

Reverse primer  1        CGCAGATGCCATAGGAGACC  20
Template        5474283  ....................  5474264
```

>CP047070.1 Pseudomonas aeruginosa strain Environ_2 chromosome

```
product length = 269
Forward primer  1        GCAACGACACCAGCACACC  19
Template        5473961  ...................  5473979

Reverse primer  1        CGCAGATGCCATAGGAGACC  20
Template        5474229  ....................  5474210
```

>CP047064.1 Pseudomonas aeruginosa strain delta6_5 chromosome

```
product length = 269
Forward primer  1        GCAACGACACCAGCACACC  19
Template        5474033  ...................  5474051

Reverse primer  1        CGCAGATGCCATAGGAGACC  20
Template        5474301  ....................  5474282
```

>CP047061.1 Pseudomonas aeruginosa strain delta6_2 chromosome

```
product length = 269
Forward primer  1        GCAACGACACCAGCACACC  19
Template        5474008  ...................  5474026

Reverse primer  1        CGCAGATGCCATAGGAGACC  20
Template        5474276  ....................  5474257
```

>CP047062.1 Pseudomonas aeruginosa strain delta6_3 chromosome

```
product length = 269
Forward primer  1        GCAACGACACCAGCACACC  19
Template        5474008  ...................  5474026

Reverse primer  1        CGCAGATGCCATAGGAGACC  20
Template        5474276  ....................  5474257
```

>CP047067.1 Pseudomonas aeruginosa strain Cas9_1 chromosome

```
product length = 269
Forward primer  1        GCAACGACACCAGCACACC  19
Template        5474027  ...................  5474045

Reverse primer  1        CGCAGATGCCATAGGAGACC  20
Template        5474295  ....................  5474276
```

>CP047066.1 Pseudomonas aeruginosa strain delta10 chromosome

```
product length = 269
Forward primer  1        GCAACGACACCAGCACACC  19
Template        5474034  ...................  5474052

Reverse primer  1        CGCAGATGCCATAGGAGACC  20
Template        5474302  ....................  5474283
```

>CP047068.1 Pseudomonas aeruginosa strain Cas9_2 chromosome

```
product length = 269
Forward primer  1        GCAACGACACCAGCACACC  19
Template        5474027  ...................  5474045

Reverse primer  1        CGCAGATGCCATAGGAGACC  20
Template        5474295  ....................  5474276
```

>CP047065.1 Pseudomonas aeruginosa strain delta6_6 chromosome

```
product length = 269
Forward primer  1        GCAACGACACCAGCACACC  19
Template        5474015  ...................  5474033

Reverse primer  1        CGCAGATGCCATAGGAGACC  20
Template        5474283  ....................  5474264
```

>CP039990.1 Pseudomonas aeruginosa strain T2101 chromosome, complete genome

```
product length = 269
Forward primer  1        GCAACGACACCAGCACACC  19
Template        5720491  ...................  5720509

Reverse primer  1        CGCAGATGCCATAGGAGACC  20
Template        5720759  ....................  5720740
```

>CP039988.1 Pseudomonas aeruginosa strain T2436 chromosome, complete genome

```
product length = 269
Forward primer  1        GCAACGACACCAGCACACC  19
Template        5987688  ...................  5987706

Reverse primer  1        CGCAGATGCCATAGGAGACC  20
Template        5987956  ....................  5987937
```

>CP046069.1 Pseudomonas aeruginosa strain KRP1 chromosome, complete genome

```
product length = 269
Forward primer  1        GCAACGACACCAGCACACC  19
Template        5850188  ...................  5850206

Reverse primer  1        CGCAGATGCCATAGGAGACC  20
Template        5850456  ....................  5850437
```

>CP046060.1 Pseudomonas aeruginosa strain 1811-18R001 chromosome, complete genome

```
product length = 269
Forward primer  1        GCAACGACACCAGCACACC  19
Template        6502756  ...................  6502774

Reverse primer  1        CGCAGATGCCATAGGAGACC  20
Template        6503024  ....................  6503005
```

>CP046061.1 Pseudomonas aeruginosa strain 1811-13R031 chromosome, complete genome

```
product length = 269
Forward primer  1        GCAACGACACCAGCACACC  19
Template        6502552  ...................  6502570

Reverse primer  1        CGCAGATGCCATAGGAGACC  20
Template        6502820  ....................  6502801
```

>CP041945.1 Pseudomonas aeruginosa strain ST773 chromosome, complete genome

```
product length = 269
Forward primer  1        GCAACGACACCAGCACACC  19
Template        5909145  ...................  5909163

Reverse primer  1        CGCAGATGCCATAGGAGACC  20
Template        5909413  ....................  5909394
```

>CP045739.1 Pseudomonas aeruginosa strain AG1 chromosome, complete genome

```
product length = 269
Forward primer  1        GCAACGACACCAGCACACC  19
Template        6321303  ...................  6321321

Reverse primer  1        CGCAGATGCCATAGGAGACC  20
Template        6321571  ....................  6321552
```

>CP045768.1 Pseudomonas aeruginosa strain CFSAN084950 chromosome, complete genome

```
product length = 269
Forward primer  1        GCAACGACACCAGCACACC  19
Template        4618784  ...................  4618802

Reverse primer  1        CGCAGATGCCATAGGAGACC  20
Template        4619052  ....................  4619033
```

>CP042967.1 Pseudomonas aeruginosa PA99 chromosome, complete genome

```
product length = 269
Forward primer  1        GCAACGACACCAGCACACC  19
Template        2197817  ...................  2197835

Reverse primer  1        CGCAGATGCCATAGGAGACC  20
Template        2198085  ....................  2198066
```

>CP024630.1 Pseudomonas aeruginosa strain PA59 chromosome, complete genome

```
product length = 269
Forward primer  1        GCAACGACACCAGCACACC  19
Template        6105153  ...................  6105171

Reverse primer  1        CGCAGATGCCATAGGAGACC  20
Template        6105421  ....................  6105402
```

>CP043549.1 Pseudomonas aeruginosa strain GIMC5002:PAT-169 chromosome

```
product length = 269
Forward primer  1       GCAACGACACCAGCACACC  19
Template        686763  ...................  686745

Reverse primer  1       CGCAGATGCCATAGGAGACC  20
Template        686495  ....................  686514
```

>CP040684.1 Pseudomonas aeruginosa strain C79 chromosome, complete genome

```
product length = 269
Forward primer  1        GCAACGACACCAGCACACC  19
Template        5505526  ...................  5505508

Reverse primer  1        CGCAGATGCCATAGGAGACC  20
Template        5505258  ....................  5505277
```

>CP044006.1 Pseudomonas aeruginosa strain E90 chromosome, complete genome

```
product length = 269
Forward primer  1        GCAACGACACCAGCACACC  19
Template        6009577  ...................  6009595

Reverse primer  1        CGCAGATGCCATAGGAGACC  20
Template        6009845  ....................  6009826
```

>CP043483.1 Pseudomonas aeruginosa strain GIMC5001:PAT-23 chromosome

```
product length = 269
Forward primer  1        GCAACGACACCAGCACACC  19
Template        5606827  ...................  5606845

Reverse primer  1        CGCAGATGCCATAGGAGACC  20
Template        5607095  ....................  5607076
```

>CP042269.1 Pseudomonas aeruginosa strain HOU1 chromosome, complete genome

```
product length = 269
Forward primer  1        GCAACGACACCAGCACACC  19
Template        5327291  ...................  5327309

Reverse primer  1        CGCAGATGCCATAGGAGACC  20
Template        5327559  ....................  5327540
```

>CP043328.1 Pseudomonas aeruginosa strain CCUG 51971 chromosome, complete genome

```
product length = 269
Forward primer  1        GCAACGACACCAGCACACC  19
Template        6155219  ...................  6155237

Reverse primer  1        CGCAGATGCCATAGGAGACC  20
Template        6155487  ....................  6155468
```

>CP028959.1 Pseudomonas aeruginosa strain IMP66 chromosome, complete genome

```
product length = 269
Forward primer  1        GCAACGACACCAGCACACC  19
Template        5685974  ...................  5685992

Reverse primer  1        CGCAGATGCCATAGGAGACC  20
Template        5686242  ....................  5686223
```

>CP028848.1 Pseudomonas aeruginosa strain IMP67 chromosome, complete genome

```
product length = 269
Forward primer  1        GCAACGACACCAGCACACC  19
Template        5671764  ...................  5671782

Reverse primer  1        CGCAGATGCCATAGGAGACC  20
Template        5672032  ....................  5672013
```

>CP028849.1 Pseudomonas aeruginosa strain IMP68 chromosome, complete genome

```
product length = 269
Forward primer  1        GCAACGACACCAGCACACC  19
Template        5671057  ...................  5671075

Reverse primer  1        CGCAGATGCCATAGGAGACC  20
Template        5671325  ....................  5671306
```

>CP041785.1 Pseudomonas aeruginosa strain SCAID WND3-2019 chromosome

```
product length = 269
Forward primer  1        GCAACGACACCAGCACACC  19
Template        4627405  ...................  4627423

Reverse primer  1        CGCAGATGCCATAGGAGACC  20
Template        4627673  ....................  4627654
```

>CP041787.1 Pseudomonas aeruginosa strain SCAID WND1-2019 chromosome

```
product length = 269
Forward primer  1        GCAACGACACCAGCACACC  19
Template        3486378  ...................  3486396

Reverse primer  1        CGCAGATGCCATAGGAGACC  20
Template        3486646  ....................  3486627
```

>CP041786.1 Pseudomonas aeruginosa strain SCAID WND2-2019 chromosome

```
product length = 269
Forward primer  1        GCAACGACACCAGCACACC  19
Template        5387765  ...................  5387783

Reverse primer  1        CGCAGATGCCATAGGAGACC  20
Template        5388033  ....................  5388014
```

>CP041773.1 Pseudomonas aeruginosa strain 519119 chromosome, complete genome

```
product length = 269
Forward primer  1        GCAACGACACCAGCACACC  19
Template        1586411  ...................  1586429

Reverse primer  1        CGCAGATGCCATAGGAGACC  20
Template        1586679  ....................  1586660
```

>CP041772.1 Pseudomonas aeruginosa strain 243931 chromosome, complete genome

```
product length = 269
Forward primer  1        GCAACGACACCAGCACACC  19
Template        1547107  ...................  1547125

Reverse primer  1        CGCAGATGCCATAGGAGACC  20
Template        1547375  ....................  1547356
```

>CP041771.1 Pseudomonas aeruginosa strain A681 chromosome, complete genome

```
product length = 269
Forward primer  1        GCAACGACACCAGCACACC  19
Template        5817400  ...................  5817418

Reverse primer  1        CGCAGATGCCATAGGAGACC  20
Template        5817668  ....................  5817649
```

>CP041774.1 Pseudomonas aeruginosa strain 60503 chromosome, complete genome

```
product length = 269
Forward primer  1        GCAACGACACCAGCACACC  19
Template        6014835  ...................  6014853

Reverse primer  1        CGCAGATGCCATAGGAGACC  20
Template        6015103  ....................  6015084
```

>LR657304.1 Pseudomonas aeruginosa strain PAK genome assembly, chromosome: 1

```
product length = 269
Forward primer  1        GCAACGACACCAGCACACC  19
Template        5594080  ...................  5594098

Reverse primer  1        CGCAGATGCCATAGGAGACC  20
Template        5594348  ....................  5594329
```

>CP034244.1 Pseudomonas aeruginosa UCBPP-PA14 chromosome

```
product length = 269
Forward primer  1        GCAACGACACCAGCACACC  19
Template        5744379  ...................  5744397

Reverse primer  1        CGCAGATGCCATAGGAGACC  20
Template        5744647  ....................  5744628
```

>CP041008.1 Pseudomonas aeruginosa strain FDAARGOS_767 chromosome, complete genome

```
product length = 269
Forward primer  1       GCAACGACACCAGCACACC  19
Template        250608  ...................  250590

Reverse primer  1       CGCAGATGCCATAGGAGACC  20
Template        250340  ....................  250359
```

>CP028331.1 Pseudomonas aeruginosa strain PA-VAP-2 chromosome

```
product length = 269
Forward primer  1       GCAACGACACCAGCACACC  19
Template        804032  ...................  804014

Reverse primer  1       CGCAGATGCCATAGGAGACC  20
Template        803764  ....................  803783
```

>CP032569.2 Pseudomonas aeruginosa strain BA7823 chromosome, complete genome

```
product length = 269
Forward primer  1        GCAACGACACCAGCACACC  19
Template        5538403  ...................  5538421

Reverse primer  1        CGCAGATGCCATAGGAGACC  20
Template        5538671  ....................  5538652
```

>CP040127.1 Pseudomonas aeruginosa strain PA298 chromosome, complete genome

```
product length = 269
Forward primer  1        GCAACGACACCAGCACACC  19
Template        5741474  ...................  5741492

Reverse primer  1        CGCAGATGCCATAGGAGACC  20
Template        5741742  ....................  5741723
```

>LR590474.1 Pseudomonas aeruginosa strain NCTC13618 genome assembly, chromosome: 1

```
product length = 269
Forward primer  1        GCAACGACACCAGCACACC  19
Template        5937943  ...................  5937961

Reverse primer  1        CGCAGATGCCATAGGAGACC  20
Template        5938211  ....................  5938192
```

>LR590472.1 Pseudomonas aeruginosa strain NCTC13620 genome assembly, chromosome: 1

```
product length = 269
Forward primer  1        GCAACGACACCAGCACACC  19
Template        5979496  ...................  5979514

Reverse primer  1        CGCAGATGCCATAGGAGACC  20
Template        5979764  ....................  5979745
```

>CP039293.1 Pseudomonas aeruginosa strain PABL048 chromosome, complete genome

```
product length = 269
Forward primer  1        GCAACGACACCAGCACACC  19
Template        6063334  ...................  6063352

Reverse primer  1        CGCAGATGCCATAGGAGACC  20
Template        6063602  ....................  6063583
```

>CP038661.1 Pseudomonas aeruginosa strain AJ D 2 chromosome

```
product length = 269
Forward primer  1        GCAACGACACCAGCACACC  19
Template        5488595  ...................  5488613

Reverse primer  1        CGCAGATGCCATAGGAGACC  20
Template        5488863  ....................  5488844
```

>CP037925.1 Pseudomonas aeruginosa strain AES1M chromosome, complete genome

```
product length = 269
Forward primer  1        GCAACGACACCAGCACACC  19
Template        5579338  ...................  5579356

Reverse primer  1        CGCAGATGCCATAGGAGACC  20
Template        5579606  ....................  5579587
```

>CP037926.1 Pseudomonas aeruginosa strain AES1R chromosome, complete genome

```
product length = 269
Forward primer  1        GCAACGACACCAGCACACC  19
Template        5580111  ...................  5580129

Reverse primer  1        CGCAGATGCCATAGGAGACC  20
Template        5580379  ....................  5580360
```

>CP028332.1 Pseudomonas aeruginosa strain PA-VAP-1 chromosome

```
product length = 269
Forward primer  1        GCAACGACACCAGCACACC  19
Template        5590639  ...................  5590657

Reverse primer  1        CGCAGATGCCATAGGAGACC  20
Template        5590907  ....................  5590888
```

>CP028330.1 Pseudomonas aeruginosa strain PA-VAP-3 chromosome

```
product length = 269
Forward primer  1        GCAACGACACCAGCACACC  19
Template        2861321  ...................  2861339

Reverse primer  1        CGCAGATGCCATAGGAGACC  20
Template        2861589  ....................  2861570
```

>LR134308.1 Pseudomonas aeruginosa strain NCTC11445 genome assembly, chromosome: 1

```
product length = 269
Forward primer  1        GCAACGACACCAGCACACC  19
Template        6325987  ...................  6325969

Reverse primer  1        CGCAGATGCCATAGGAGACC  20
Template        6325719  ....................  6325738
```

>CP034435.1 Pseudomonas aeruginosa strain B14130 chromosome, complete genome

```
product length = 269
Forward primer  1       GCAACGACACCAGCACACC  19
Template        615271  ...................  615253

Reverse primer  1       CGCAGATGCCATAGGAGACC  20
Template        615003  ....................  615022
```

>CP034436.1 Pseudomonas aeruginosa strain B17932 chromosome, complete genome

```
product length = 269
Forward primer  1       GCAACGACACCAGCACACC  19
Template        505978  ...................  505960

Reverse primer  1       CGCAGATGCCATAGGAGACC  20
Template        505710  ....................  505729
```

>LR130528.1 Pseudomonas aeruginosa isolate paerg000 genome assembly, chromosome: 0

```
product length = 269
Forward primer  1        GCAACGACACCAGCACACC  19
Template        1336726  ...................  1336708

Reverse primer  1        CGCAGATGCCATAGGAGACC  20
Template        1336458  ....................  1336477
```

>CP033835.1 Pseudomonas aeruginosa strain FDAARGOS_570 chromosome, complete genome

```
product length = 269
Forward primer  1       GCAACGACACCAGCACACC  19
Template        909162  ...................  909144

Reverse primer  1       CGCAGATGCCATAGGAGACC  20
Template        908894  ....................  908913
```

>CP033439.1 Pseudomonas aeruginosa strain SP4528 chromosome, complete genome

```
product length = 269
Forward primer  1       GCAACGACACCAGCACACC  19
Template        678990  ...................  678972

Reverse primer  1       CGCAGATGCCATAGGAGACC  20
Template        678722  ....................  678741
```

>CP033432.1 Pseudomonas aeruginosa strain BA15561 chromosome, complete genome

```
product length = 269
Forward primer  1        GCAACGACACCAGCACACC  19
Template        5708328  ...................  5708310

Reverse primer  1        CGCAGATGCCATAGGAGACC  20
Template        5708060  ....................  5708079
```

>CP033084.1 Pseudomonas aeruginosa strain PA-3 chromosome, complete genome

```
product length = 269
Forward primer  1        GCAACGACACCAGCACACC  19
Template        5339103  ...................  5339085

Reverse primer  1        CGCAGATGCCATAGGAGACC  20
Template        5338835  ....................  5338854
```

>CP031877.1 Pseudomonas aeruginosa strain WPB100 chromosome

```
product length = 269
Forward primer  1       GCAACGACACCAGCACACC  19
Template        954817  ...................  954799

Reverse primer  1       CGCAGATGCCATAGGAGACC  20
Template        954549  ....................  954568
```

>CP031876.1 Pseudomonas aeruginosa strain WPB101 chromosome

```
product length = 269
Forward primer  1       GCAACGACACCAGCACACC  19
Template        923792  ...................  923774

Reverse primer  1       CGCAGATGCCATAGGAGACC  20
Template        923524  ....................  923543
```

>CP031878.1 Pseudomonas aeruginosa strain WPB099 chromosome

```
product length = 269
Forward primer  1       GCAACGACACCAGCACACC  19
Template        923975  ...................  923957

Reverse primer  1       CGCAGATGCCATAGGAGACC  20
Template        923707  ....................  923726
```

>CP031879.1 Pseudomonas aeruginosa strain WPB098 chromosome

```
product length = 269
Forward primer  1       GCAACGACACCAGCACACC  19
Template        778966  ...................  778948

Reverse primer  1       CGCAGATGCCATAGGAGACC  20
Template        778698  ....................  778717
```

>CP035739.1 Pseudomonas aeruginosa strain 1334/14 chromosome, complete genome

```
product length = 269
Forward primer  1        GCAACGACACCAGCACACC  19
Template        4983748  ...................  4983730

Reverse primer  1        CGCAGATGCCATAGGAGACC  20
Template        4983480  ....................  4983499
```

>CP031677.1 Pseudomonas aeruginosa strain E80 chromosome, complete genome

```
product length = 269
Forward primer  1        GCAACGACACCAGCACACC  19
Template        5987493  ...................  5987511

Reverse primer  1        CGCAGATGCCATAGGAGACC  20
Template        5987761  ....................  5987742
```

>CP032541.1 Pseudomonas aeruginosa strain PGN5 chromosome

```
product length = 269
Forward primer  1        GCAACGACACCAGCACACC  19
Template        5474055  ...................  5474073

Reverse primer  1        CGCAGATGCCATAGGAGACC  20
Template        5474323  ....................  5474304
```

>CP032540.1 Pseudomonas aeruginosa strain PGN4 chromosome

```
product length = 269
Forward primer  1        GCAACGACACCAGCACACC  19
Template        5474037  ...................  5474055

Reverse primer  1        CGCAGATGCCATAGGAGACC  20
Template        5474305  ....................  5474286
```

>CP034434.1 Pseudomonas aeruginosa strain SP2230 chromosome, complete genome

```
product length = 269
Forward primer  1        GCAACGACACCAGCACACC  19
Template        4911719  ...................  4911737

Reverse primer  1        CGCAGATGCCATAGGAGACC  20
Template        4911987  ....................  4911968
```

>CP034409.1 Pseudomonas aeruginosa strain SP4527 chromosome, complete genome

```
product length = 269
Forward primer  1        GCAACGACACCAGCACACC  19
Template        4279618  ...................  4279636

Reverse primer  1        CGCAGATGCCATAGGAGACC  20
Template        4279886  ....................  4279867
```

>CP034369.1 Pseudomonas aeruginosa strain SP4371 chromosome, complete genome

```
product length = 269
Forward primer  1        GCAACGACACCAGCACACC  19
Template        5182236  ...................  5182254

Reverse primer  1        CGCAGATGCCATAGGAGACC  20
Template        5182504  ....................  5182485
```

>CP034368.1 Pseudomonas aeruginosa strain B41226 chromosome, complete genome

```
product length = 269
Forward primer  1        GCAACGACACCAGCACACC  19
Template        5447511  ...................  5447529

Reverse primer  1        CGCAGATGCCATAGGAGACC  20
Template        5447779  ....................  5447760
```

>CP034354.1 Pseudomonas aeruginosa strain IMP-13 chromosome, complete genome

```
product length = 269
Forward primer  1        GCAACGACACCAGCACACC  19
Template        2298193  ...................  2298211

Reverse primer  1        CGCAGATGCCATAGGAGACC  20
Template        2298461  ....................  2298442
```

>LR130537.1 Pseudomonas aeruginosa isolate paerg012 genome assembly, chromosome: 0

```
product length = 269
Forward primer  1        GCAACGACACCAGCACACC  19
Template        5631930  ...................  5631948

Reverse primer  1        CGCAGATGCCATAGGAGACC  20
Template        5632198  ....................  5632179
```

>LR130536.1 Pseudomonas aeruginosa isolate paerg010 genome assembly, chromosome: 0

```
product length = 269
Forward primer  1        GCAACGACACCAGCACACC  19
Template        5631904  ...................  5631922

Reverse primer  1        CGCAGATGCCATAGGAGACC  20
Template        5632172  ....................  5632153
```

>LR130535.1 Pseudomonas aeruginosa isolate paerg011 genome assembly, chromosome: 0

```
product length = 269
Forward primer  1        GCAACGACACCAGCACACC  19
Template        5632047  ...................  5632065

Reverse primer  1        CGCAGATGCCATAGGAGACC  20
Template        5632315  ....................  5632296
```

>LR130534.1 Pseudomonas aeruginosa isolate paerg005 genome assembly, chromosome: 0

```
product length = 269
Forward primer  1        GCAACGACACCAGCACACC  19
Template        6090138  ...................  6090156

Reverse primer  1        CGCAGATGCCATAGGAGACC  20
Template        6090406  ....................  6090387
```

>LR130533.1 Pseudomonas aeruginosa isolate paerg009 genome assembly, chromosome: 0

```
product length = 269
Forward primer  1        GCAACGACACCAGCACACC  19
Template        2479612  ...................  2479630

Reverse primer  1        CGCAGATGCCATAGGAGACC  20
Template        2479880  ....................  2479861
```

>LR130531.1 Pseudomonas aeruginosa isolate paerg004 genome assembly, chromosome: 0

```
product length = 269
Forward primer  1        GCAACGACACCAGCACACC  19
Template        2949620  ...................  2949638

Reverse primer  1        CGCAGATGCCATAGGAGACC  20
Template        2949888  ....................  2949869
```

>LR130530.1 Pseudomonas aeruginosa isolate paerg003 genome assembly, chromosome: 0

```
product length = 269
Forward primer  1        GCAACGACACCAGCACACC  19
Template        5631907  ...................  5631925

Reverse primer  1        CGCAGATGCCATAGGAGACC  20
Template        5632175  ....................  5632156
```

>LR130527.1 Pseudomonas aeruginosa isolate paerg002 genome assembly, chromosome: 0

```
product length = 269
Forward primer  1        GCAACGACACCAGCACACC  19
Template        2312504  ...................  2312522

Reverse primer  1        CGCAGATGCCATAGGAGACC  20
Template        2312772  ....................  2312753
```

>CP033832.1 Pseudomonas aeruginosa strain FDAARGOS_505 chromosome, complete genome

```
product length = 269
Forward primer  1       GCAACGACACCAGCACACC  19
Template        544913  ...................  544931

Reverse primer  1       CGCAGATGCCATAGGAGACC  20
Template        545181  ....................  545162
```

>CP033833.1 Pseudomonas aeruginosa strain FDAARGOS_571 chromosome, complete genome

```
product length = 269
Forward primer  1        GCAACGACACCAGCACACC  19
Template        2251438  ...................  2251456

Reverse primer  1        CGCAGATGCCATAGGAGACC  20
Template        2251706  ....................  2251687
```

>CP033843.1 Pseudomonas aeruginosa strain FDAARGOS_501 chromosome, complete genome

```
product length = 269
Forward primer  1        GCAACGACACCAGCACACC  19
Template        1609236  ...................  1609254

Reverse primer  1        CGCAGATGCCATAGGAGACC  20
Template        1609504  ....................  1609485
```

>CP033771.1 Pseudomonas aeruginosa strain FDAARGOS_532 chromosome, complete genome

```
product length = 269
Forward primer  1        GCAACGACACCAGCACACC  19
Template        3843958  ...................  3843976

Reverse primer  1        CGCAGATGCCATAGGAGACC  20
Template        3844226  ....................  3844207
```

>CP033684.1 Pseudomonas aeruginosa strain H26027 chromosome, complete genome

```
product length = 269
Forward primer  1        GCAACGACACCAGCACACC  19
Template        6222614  ...................  6222632

Reverse primer  1        CGCAGATGCCATAGGAGACC  20
Template        6222882  ....................  6222863
```

>CP033686.1 Pseudomonas aeruginosa strain H25883 chromosome, complete genome

```
product length = 269
Forward primer  1        GCAACGACACCAGCACACC  19
Template        5848091  ...................  5848109

Reverse primer  1        CGCAGATGCCATAGGAGACC  20
Template        5848359  ....................  5848340
```

>CP033685.1 Pseudomonas aeruginosa strain H26023 chromosome, complete genome

```
product length = 269
Forward primer  1        GCAACGACACCAGCACACC  19
Template        5900500  ...................  5900518

Reverse primer  1        CGCAGATGCCATAGGAGACC  20
Template        5900768  ....................  5900749
```

>CP029713.1 Pseudomonas aeruginosa strain BH9 chromosome

```
product length = 269
Forward primer  1        GCAACGACACCAGCACACC  19
Template        6295612  ...................  6295630

Reverse primer  1        CGCAGATGCCATAGGAGACC  20
Template        6295880  ....................  6295861
```

>CP030075.1 Pseudomonas aeruginosa strain 6762 chromosome

```
product length = 269
Forward primer  1        GCAACGACACCAGCACACC  19
Template        1283097  ...................  1283115

Reverse primer  1        CGCAGATGCCATAGGAGACC  20
Template        1283365  ....................  1283346
```

>CP032552.1 Pseudomonas aeruginosa strain PA34 chromosome, complete genome

```
product length = 269
Forward primer  1        GCAACGACACCAGCACACC  19
Template        6001033  ...................  6001051

Reverse primer  1        CGCAGATGCCATAGGAGACC  20
Template        6001301  ....................  6001282
```

>CP032761.1 Pseudomonas aeruginosa strain 268 chromosome, complete genome

```
product length = 269
Forward primer  1        GCAACGACACCAGCACACC  19
Template        6209009  ...................  6209027

Reverse primer  1        CGCAGATGCCATAGGAGACC  20
Template        6209277  ....................  6209258
```

>CP028584.2 Pseudomonas aeruginosa strain WCHPA075019 chromosome, complete genome

```
product length = 269
Forward primer  1        GCAACGACACCAGCACACC  19
Template        6078834  ...................  6078852

Reverse primer  1        CGCAGATGCCATAGGAGACC  20
Template        6079102  ....................  6079083
```

>CP032257.1 Pseudomonas aeruginosa strain AR_0111 chromosome, complete genome

```
product length = 269
Forward primer  1        GCAACGACACCAGCACACC  19
Template        2412084  ...................  2412102

Reverse primer  1        CGCAGATGCCATAGGAGACC  20
Template        2412352  ....................  2412333
```

>CP029605.1 Pseudomonas aeruginosa strain 24Pae112 chromosome, complete genome

```
product length = 269
Forward primer  1        GCAACGACACCAGCACACC  19
Template        6238684  ...................  6238702

Reverse primer  1        CGCAGATGCCATAGGAGACC  20
Template        6238952  ....................  6238933
```

>CP031659.1 Pseudomonas aeruginosa strain PABL012 chromosome, complete genome

```
product length = 269
Forward primer  1        GCAACGACACCAGCACACC  19
Template        5750512  ...................  5750530

Reverse primer  1        CGCAGATGCCATAGGAGACC  20
Template        5750780  ....................  5750761
```

>CP030911.1 Pseudomonas aeruginosa strain Y71 chromosome, complete genome

```
product length = 269
Forward primer  1        GCAACGACACCAGCACACC  19
Template        6075977  ...................  6075995

Reverse primer  1        CGCAGATGCCATAGGAGACC  20
Template        6076245  ....................  6076226
```

>CP031449.2 Pseudomonas aeruginosa strain 97 chromosome, complete genome

```
product length = 269
Forward primer  1        GCAACGACACCAGCACACC  19
Template        6117038  ...................  6117056

Reverse primer  1        CGCAGATGCCATAGGAGACC  20
Template        6117306  ....................  6117287
```

>LS998783.1 Pseudomonas aeruginosa isolate 1 genome assembly, chromosome: 1

```
product length = 269
Forward primer  1        GCAACGACACCAGCACACC  19
Template        6327168  ...................  6327186

Reverse primer  1        CGCAGATGCCATAGGAGACC  20
Template        6327436  ....................  6327417
```

>CP032126.1 Pseudomonas aeruginosa strain PAO1161 chromosome, complete genome

```
product length = 269
Forward primer  1        GCAACGACACCAGCACACC  19
Template        5593428  ...................  5593446

Reverse primer  1        CGCAGATGCCATAGGAGACC  20
Template        5593696  ....................  5593677
```

>CP030861.1 Pseudomonas aeruginosa strain HS9 chromosome, complete genome

```
product length = 269
Forward primer  1        GCAACGACACCAGCACACC  19
Template        3388802  ...................  3388784

Reverse primer  1        CGCAGATGCCATAGGAGACC  20
Template        3388534  ....................  3388553
```

>CP030327.1 Pseudomonas aeruginosa strain AR_458 chromosome, complete genome

```
product length = 269
Forward primer  1        GCAACGACACCAGCACACC  19
Template        3858684  ...................  3858666

Reverse primer  1        CGCAGATGCCATAGGAGACC  20
Template        3858416  ....................  3858435
```

>CP029745.1 Pseudomonas aeruginosa strain AR_0110 chromosome, complete genome

```
product length = 269
Forward primer  1        GCAACGACACCAGCACACC  19
Template        1603712  ...................  1603694

Reverse primer  1        CGCAGATGCCATAGGAGACC  20
Template        1603444  ....................  1603463
```

>CP029097.1 Pseudomonas aeruginosa strain AR439 chromosome, complete genome

```
product length = 269
Forward primer  1        GCAACGACACCAGCACACC  19
Template        1062156  ...................  1062138

Reverse primer  1        CGCAGATGCCATAGGAGACC  20
Template        1061888  ....................  1061907
```

>CP028917.1 Pseudomonas aeruginosa strain JB2 chromosome, complete genome

```
product length = 269
Forward primer  1        GCAACGACACCAGCACACC  19
Template        1300692  ...................  1300674

Reverse primer  1        CGCAGATGCCATAGGAGACC  20
Template        1300424  ....................  1300443
```

>CP027538.1 Pseudomonas aeruginosa strain AR_0095 chromosome, complete genome

```
product length = 269
Forward primer  1        GCAACGACACCAGCACACC  19
Template        3886048  ...................  3886030

Reverse primer  1        CGCAGATGCCATAGGAGACC  20
Template        3885780  ....................  3885799
```

>CP027166.1 Pseudomonas aeruginosa strain AR_0357 chromosome, complete genome

```
product length = 269
Forward primer  1        GCAACGACACCAGCACACC  19
Template        2592800  ...................  2592782

Reverse primer  1        CGCAGATGCCATAGGAGACC  20
Template        2592532  ....................  2592551
```

>CP027172.1 Pseudomonas aeruginosa strain AR_0353 chromosome, complete genome

```
product length = 269
Forward primer  1       GCAACGACACCAGCACACC  19
Template        960879  ...................  960861

Reverse primer  1       CGCAGATGCCATAGGAGACC  20
Template        960611  ....................  960630
```

>CP022478.1 Pseudomonas aeruginosa strain LW chromosome, complete genome

```
product length = 269
Forward primer  1        GCAACGACACCAGCACACC  19
Template        4723479  ...................  4723497

Reverse primer  1        CGCAGATGCCATAGGAGACC  20
Template        4723747  ....................  4723728
```

>CP034430.1 Pseudomonas aeruginosa strain GIMC5016:PA1840 chromosome

```
product length = 269
Forward primer  1        GCAACGACACCAGCACACC  19
Template        5850642  ...................  5850660

Reverse primer  1        CGCAGATGCCATAGGAGACC  20
Template        5850910  ....................  5850891
```

>CP034429.1 Pseudomonas aeruginosa strain GIMC5015:PAKB6, complete sequence

```
product length = 269
Forward primer  1        GCAACGACACCAGCACACC  19
Template        5472781  ...................  5472799

Reverse primer  1        CGCAGATGCCATAGGAGACC  20
Template        5473049  ....................  5473030
```

>LR134342.1 Pseudomonas aeruginosa strain NCTC10728 genome assembly, chromosome: 1

```
product length = 269
Forward primer  1        GCAACGACACCAGCACACC  19
Template        2175163  ...................  2175181

Reverse primer  1        CGCAGATGCCATAGGAGACC  20
Template        2175431  ....................  2175412
```

>LR134330.1 Pseudomonas aeruginosa strain NCTC13715 genome assembly, chromosome: 1

```
product length = 269
Forward primer  1       GCAACGACACCAGCACACC  19
Template        979253  ...................  979271

Reverse primer  1       CGCAGATGCCATAGGAGACC  20
Template        979521  ....................  979502
```

>LR134309.1 Pseudomonas aeruginosa strain NCTC12903 genome assembly, chromosome: 1

```
product length = 269
Forward primer  1        GCAACGACACCAGCACACC  19
Template        6032125  ...................  6032143

Reverse primer  1        CGCAGATGCCATAGGAGACC  20
Template        6032393  ....................  6032374
```

>LR134300.1 Pseudomonas fluorescens strain NCTC10783 genome assembly, chromosome: 1

```
product length = 269
Forward primer  1        GCAACGACACCAGCACACC  19
Template        4831896  ...................  4831914

Reverse primer  1        CGCAGATGCCATAGGAGACC  20
Template        4832164  ....................  4832145
```

>CP030913.1 Pseudomonas aeruginosa strain Y89 chromosome, complete genome

```
product length = 269
Forward primer  1        GCAACGACACCAGCACACC  19
Template        6004509  ...................  6004527

Reverse primer  1        CGCAGATGCCATAGGAGACC  20
Template        6004777  ....................  6004758
```

>CP030912.1 Pseudomonas aeruginosa strain Y82 chromosome, complete genome

```
product length = 269
Forward primer  1        GCAACGACACCAGCACACC  19
Template        6284479  ...................  6284497

Reverse primer  1        CGCAGATGCCATAGGAGACC  20
Template        6284747  ....................  6284728
```

>CP030910.1 Pseudomonas aeruginosa strain Y31 chromosome, complete genome

```
product length = 269
Forward primer  1        GCAACGACACCAGCACACC  19
Template        5999226  ...................  5999244

Reverse primer  1        CGCAGATGCCATAGGAGACC  20
Template        5999494  ....................  5999475
```

>CP030351.1 Pseudomonas aeruginosa strain AR_460 chromosome, complete genome

```
product length = 269
Forward primer  1        GCAACGACACCAGCACACC  19
Template        3353731  ...................  3353749

Reverse primer  1        CGCAGATGCCATAGGAGACC  20
Template        3353999  ....................  3353980
```

>CP030328.1 Pseudomonas aeruginosa strain AR_455 chromosome, complete genome

```
product length = 269
Forward primer  1       GCAACGACACCAGCACACC  19
Template        530842  ...................  530860

Reverse primer  1       CGCAGATGCCATAGGAGACC  20
Template        531110  ....................  531091
```

>LS483497.1 Pseudomonas aeruginosa strain NCTC9433 genome assembly, chromosome: 1

```
product length = 269
Forward primer  1        GCAACGACACCAGCACACC  19
Template        5637221  ...................  5637239

Reverse primer  1        CGCAGATGCCATAGGAGACC  20
Template        5637489  ....................  5637470
```

>CP029707.1 Pseudomonas aeruginosa strain K34-7 chromosome, complete genome

```
product length = 269
Forward primer  1        GCAACGACACCAGCACACC  19
Template        4623460  ...................  4623478

Reverse primer  1        CGCAGATGCCATAGGAGACC  20
Template        4623728  ....................  4623709
```

>CP023255.1 Pseudomonas aeruginosa strain CCUG 70744 chromosome, complete genome

```
product length = 269
Forward primer  1        GCAACGACACCAGCACACC  19
Template        3970638  ...................  3970656

Reverse primer  1        CGCAGATGCCATAGGAGACC  20
Template        3970906  ....................  3970887
```

>CP029148.1 Pseudomonas aeruginosa strain AR_0440 chromosome

```
product length = 269
Forward primer  1        GCAACGACACCAGCACACC  19
Template        5261208  ...................  5261226

Reverse primer  1        CGCAGATGCCATAGGAGACC  20
Template        5261476  ....................  5261457
```

>CP029147.1 Pseudomonas aeruginosa strain AR_0443 chromosome

```
product length = 269
Forward primer  1        GCAACGACACCAGCACACC  19
Template        2626365  ...................  2626383

Reverse primer  1        CGCAGATGCCATAGGAGACC  20
Template        2626633  ....................  2626614
```

>CP029090.1 Pseudomonas aeruginosa strain AR442 chromosome, complete genome

```
product length = 269
Forward primer  1        GCAACGACACCAGCACACC  19
Template        5370482  ...................  5370500

Reverse primer  1        CGCAGATGCCATAGGAGACC  20
Template        5370750  ....................  5370731
```

>CP029089.1 Pseudomonas aeruginosa strain AR444 chromosome, complete genome

```
product length = 269
Forward primer  1        GCAACGACACCAGCACACC  19
Template        4813140  ...................  4813158

Reverse primer  1        CGCAGATGCCATAGGAGACC  20
Template        4813408  ....................  4813389
```

>CP029088.1 Pseudomonas aeruginosa strain AR445 chromosome, complete genome

```
product length = 269
Forward primer  1        GCAACGACACCAGCACACC  19
Template        6274583  ...................  6274601

Reverse primer  1        CGCAGATGCCATAGGAGACC  20
Template        6274851  ....................  6274832
```

>CP023316.1 Pseudomonas aeruginosa strain PPF-1 chromosome, complete genome

```
product length = 269
Forward primer  1        GCAACGACACCAGCACACC  19
Template        6078810  ...................  6078828

Reverse primer  1        CGCAGATGCCATAGGAGACC  20
Template        6079078  ....................  6079059
```

>CP028162.1 Pseudomonas aeruginosa strain MRSN12280 chromosome, complete genome

```
product length = 269
Forward primer  1        GCAACGACACCAGCACACC  19
Template        6220052  ...................  6220070

Reverse primer  1        CGCAGATGCCATAGGAGACC  20
Template        6220320  ....................  6220301
```

>CP027174.1 Pseudomonas aeruginosa strain AR_0230 chromosome, complete genome

```
product length = 269
Forward primer  1        GCAACGACACCAGCACACC  19
Template        4665451  ...................  4665469

Reverse primer  1        CGCAGATGCCATAGGAGACC  20
Template        4665719  ....................  4665700
```

>CP027171.1 Pseudomonas aeruginosa strain AR_0354 chromosome, complete genome

```
product length = 269
Forward primer  1        GCAACGACACCAGCACACC  19
Template        3793061  ...................  3793079

Reverse primer  1        CGCAGATGCCATAGGAGACC  20
Template        3793329  ....................  3793310
```

>CP027165.1 Pseudomonas aeruginosa strain AR_0360 chromosome, complete genome

```
product length = 269
Forward primer  1        GCAACGACACCAGCACACC  19
Template        4443588  ...................  4443606

Reverse primer  1        CGCAGATGCCATAGGAGACC  20
Template        4443856  ....................  4443837
```

>CP025229.1 Pseudomonas sp. AK6U chromosome, complete genome

```
product length = 269
Forward primer  1        GCAACGACACCAGCACACC  19
Template        5833515  ...................  5833497

Reverse primer  1        CGCAGATGCCATAGGAGACC  20
Template        5833247  ....................  5833266
```

>CP022525.1 Pseudomonas aeruginosa strain Ocean-1175, complete genome

```
product length = 269
Forward primer  1        GCAACGACACCAGCACACC  19
Template        2697640  ...................  2697622

Reverse primer  1        CGCAGATGCCATAGGAGACC  20
Template        2697372  ....................  2697391
```

>CP026680.1 Pseudomonas aeruginosa strain F5677 chromosome, complete genome

```
product length = 269
Forward primer  1        GCAACGACACCAGCACACC  19
Template        5786352  ...................  5786370

Reverse primer  1        CGCAGATGCCATAGGAGACC  20
Template        5786620  ....................  5786601
```

>LT969520.1 Pseudomonas aeruginosa isolate RW109 genome assembly, chromosome: Main_chromosome

```
product length = 269
Forward primer  1        GCAACGACACCAGCACACC  19
Template        6205065  ...................  6205083

Reverse primer  1        CGCAGATGCCATAGGAGACC  20
Template        6205333  ....................  6205314
```

>CP025051.1 Pseudomonas aeruginosa strain PB353 chromosome, complete genome

```
product length = 269
Forward primer  1        GCAACGACACCAGCACACC  19
Template        5634695  ...................  5634713

Reverse primer  1        CGCAGATGCCATAGGAGACC  20
Template        5634963  ....................  5634944
```

>CP025050.1 Pseudomonas aeruginosa strain PB368 chromosome, complete genome

```
product length = 269
Forward primer  1        GCAACGACACCAGCACACC  19
Template        5777995  ...................  5778013

Reverse primer  1        CGCAGATGCCATAGGAGACC  20
Template        5778263  ....................  5778244
```

>CP025049.1 Pseudomonas aeruginosa strain PB369 chromosome, complete genome

```
product length = 269
Forward primer  1        GCAACGACACCAGCACACC  19
Template        5666764  ...................  5666782

Reverse primer  1        CGCAGATGCCATAGGAGACC  20
Template        5667032  ....................  5667013
```

>CP025053.1 Pseudomonas aeruginosa strain PB354 chromosome, complete genome

```
product length = 269
Forward primer  1        GCAACGACACCAGCACACC  19
Template        5631532  ...................  5631550

Reverse primer  1        CGCAGATGCCATAGGAGACC  20
Template        5631800  ....................  5631781
```

>CP024477.1 Pseudomonas aeruginosa strain 12939 chromosome, complete genome

```
product length = 269
Forward primer  1        GCAACGACACCAGCACACC  19
Template        5751899  ...................  5751917

Reverse primer  1        CGCAGATGCCATAGGAGACC  20
Template        5752167  ....................  5752148
```

>CP017306.1 Pseudomonas aeruginosa strain PA_150577 chromosome, complete genome

```
product length = 269
Forward primer  1        GCAACGACACCAGCACACC  19
Template        5531926  ...................  5531944

Reverse primer  1        CGCAGATGCCATAGGAGACC  20
Template        5532194  ....................  5532175
```

>CP022526.1 Pseudomonas aeruginosa strain Ocean-1155, complete genome

```
product length = 269
Forward primer  1        GCAACGACACCAGCACACC  19
Template        2916952  ...................  2916970

Reverse primer  1        CGCAGATGCCATAGGAGACC  20
Template        2917220  ....................  2917201
```

>CP022002.1 Pseudomonas aeruginosa strain Pa1242, complete genome

```
product length = 269
Forward primer  1        GCAACGACACCAGCACACC  19
Template        1320347  ...................  1320329

Reverse primer  1        CGCAGATGCCATAGGAGACC  20
Template        1320079  ....................  1320098
```

>CP022001.1 Pseudomonas aeruginosa strain Pa1207, complete genome

```
product length = 269
Forward primer  1        GCAACGACACCAGCACACC  19
Template        1329653  ...................  1329635

Reverse primer  1        CGCAGATGCCATAGGAGACC  20
Template        1329385  ....................  1329404
```

>CP015648.1 Pseudomonas aeruginosa strain M8A4 genome

```
product length = 269
Forward primer  1       GCAACGACACCAGCACACC  19
Template        677253  ...................  677235

Reverse primer  1       CGCAGATGCCATAGGAGACC  20
Template        676985  ....................  677004
```

>CP016955.1 Pseudomonas aeruginosa strain RIVM-EMC2982, complete genome

```
product length = 269
Forward primer  1       GCAACGACACCAGCACACC  19
Template        866160  ...................  866142

Reverse primer  1       CGCAGATGCCATAGGAGACC  20
Template        865892  ....................  865911
```

>CP019338.1 Pseudomonas aeruginosa strain L10, complete genome

```
product length = 269
Forward primer  1        GCAACGACACCAGCACACC  19
Template        5869269  ...................  5869287

Reverse primer  1        CGCAGATGCCATAGGAGACC  20
Template        5869537  ....................  5869518
```

>CP017293.1 Pseudomonas aeruginosa strain PA83, complete genome

```
product length = 269
Forward primer  1        GCAACGACACCAGCACACC  19
Template        5927162  ...................  5927180

Reverse primer  1        CGCAGATGCCATAGGAGACC  20
Template        5927430  ....................  5927411
```

>CP022000.1 Pseudomonas aeruginosa strain Pa127, complete genome

```
product length = 269
Forward primer  1        GCAACGACACCAGCACACC  19
Template        6212146  ...................  6212164

Reverse primer  1        CGCAGATGCCATAGGAGACC  20
Template        6212414  ....................  6212395
```

>CP021999.1 Pseudomonas aeruginosa strain Pa84, complete genome

```
product length = 269
Forward primer  1        GCAACGACACCAGCACACC  19
Template        5773673  ...................  5773691

Reverse primer  1        CGCAGATGCCATAGGAGACC  20
Template        5773941  ....................  5773922
```

>LT883143.1 Pseudomonas aeruginosa C-NN2 isolate early isolate NN2 (clone C) genome assembly, chromosome: I

```
product length = 269
Forward primer  1        GCAACGACACCAGCACACC  19
Template        6103695  ...................  6103713

Reverse primer  1        CGCAGATGCCATAGGAGACC  20
Template        6103963  ....................  6103944
```

>CP021774.1 Pseudomonas aeruginosa strain Pa124, complete genome

```
product length = 269
Forward primer  1        GCAACGACACCAGCACACC  19
Template        6081657  ...................  6081675

Reverse primer  1        CGCAGATGCCATAGGAGACC  20
Template        6081925  ....................  6081906
```

>CP021775.1 Pseudomonas aeruginosa strain Pa58, complete genome

```
product length = 269
Forward primer  1        GCAACGACACCAGCACACC  19
Template        6432740  ...................  6432758

Reverse primer  1        CGCAGATGCCATAGGAGACC  20
```

```
Template        6433008  ....................  6432989
```

>CP015650.1 Pseudomonas aeruginosa strain Pb18 genome

```
product length = 269
Forward primer  1        GCAACGACACCAGCACACC  19
Template        3631103  ...................  3631121

Reverse primer  1        CGCAGATGCCATAGGAGACC  20
Template        3631371  ....................  3631352
```

>CP015649.1 Pseudomonas aeruginosa strain M28A1 genome

```
product length = 269
Forward primer  1        GCAACGACACCAGCACACC  19
Template        2491767  ...................  2491785

Reverse primer  1        CGCAGATGCCATAGGAGACC  20
Template        2492035  ....................  2492016
```

>CP015647.1 Pseudomonas aeruginosa strain M8A1 genome

```
product length = 269
Forward primer  1        GCAACGACACCAGCACACC  19
Template        5518662  ...................  5518680

Reverse primer  1        CGCAGATGCCATAGGAGACC  20
Template        5518930  ....................  5518911
```

>CP020704.1 Pseudomonas aeruginosa strain PASGNDM699, complete genome

```
product length = 269
Forward primer  1        GCAACGACACCAGCACACC  19
Template        6141152  ...................  6141170

Reverse primer  1        CGCAGATGCCATAGGAGACC  20
Template        6141420  ....................  6141401
```

>CP020703.1 Pseudomonas aeruginosa strain PASGNDM345, complete genome

```
product length = 269
Forward primer  1        GCAACGACACCAGCACACC  19
Template        6049215  ...................  6049233

Reverse primer  1        CGCAGATGCCATAGGAGACC  20
Template        6049483  ....................  6049464
```

>CP008858.2 Pseudomonas aeruginosa strain F63912 chromosome, complete genome

```
product length = 269
Forward primer  1        GCAACGACACCAGCACACC  19
Template        5811540  ...................  5811558
```

```
Reverse primer  1        CGCAGATGCCATAGGAGACC  20
Template        5811808  ....................  5811789
```

>CP020659.1 Pseudomonas aeruginosa PAK chromosome, complete genome

```
product length = 269
Forward primer  1        GCAACGACACCAGCACACC  19
Template        4785962  ...................  4785980

Reverse primer  1        CGCAGATGCCATAGGAGACC  20
Template        4786230  ....................  4786211
```

>CP008872.2 Pseudomonas aeruginosa strain X78812 chromosome, complete genome

```
product length = 269
Forward primer  1        GCAACGACACCAGCACACC  19
Template        5537159  ...................  5537177

Reverse primer  1        CGCAGATGCCATAGGAGACC  20
Template        5537427  ....................  5537408
```

>CP008871.2 Pseudomonas aeruginosa strain W45909 chromosome, complete genome

```
product length = 269
Forward primer  1        GCAACGACACCAGCACACC  19
Template        5927408  ...................  5927426

Reverse primer  1        CGCAGATGCCATAGGAGACC  20
Template        5927676  ....................  5927657
```

>CP008870.2 Pseudomonas aeruginosa strain W36662 chromosome, complete genome

```
product length = 269
Forward primer  1        GCAACGACACCAGCACACC  19
Template        5990816  ...................  5990834

Reverse primer  1        CGCAGATGCCATAGGAGACC  20
Template        5991084  ....................  5991065
```

>CP008869.2 Pseudomonas aeruginosa strain W16407 chromosome, complete genome

```
product length = 269
Forward primer  1        GCAACGACACCAGCACACC  19
Template        5934377  ...................  5934395

Reverse primer  1        CGCAGATGCCATAGGAGACC  20
Template        5934645  ....................  5934626
```

>CP008866.2 Pseudomonas aeruginosa strain T38079 chromosome, complete genome

```
product length = 269
Forward primer  1        GCAACGACACCAGCACACC  19
Template        5962118  ...................  5962136
```

```
Reverse primer  1        CGCAGATGCCATAGGAGACC  20
Template        5962386  ....................  5962367
```

>CP008865.2 Pseudomonas aeruginosa strain S86968 chromosome, complete genome

```
product length = 269
Forward primer  1        GCAACGACACCAGCACACC  19
Template        6085039  ...................  6085057

Reverse primer  1        CGCAGATGCCATAGGAGACC  20
Template        6085307  ....................  6085288
```

>CP008864.2 Pseudomonas aeruginosa strain W60856 chromosome, complete genome

```
product length = 269
Forward primer  1        GCAACGACACCAGCACACC  19
Template        6093625  ...................  6093643

Reverse primer  1        CGCAGATGCCATAGGAGACC  20
Template        6093893  ....................  6093874
```

>CP008862.2 Pseudomonas aeruginosa strain M1608 chromosome, complete genome

```
product length = 269
Forward primer  1        GCAACGACACCAGCACACC  19
Template        5667832  ...................  5667850

Reverse primer  1        CGCAGATGCCATAGGAGACC  20
Template        5668100  ....................  5668081
```

>CP008859.2 Pseudomonas aeruginosa strain H5708 chromosome, complete genome

```
product length = 269
Forward primer  1        GCAACGACACCAGCACACC  19
Template        5531300  ...................  5531318

Reverse primer  1        CGCAGATGCCATAGGAGACC  20
Template        5531568  ....................  5531549
```

>CP008856.2 Pseudomonas aeruginosa strain F23197 chromosome, complete genome

```
product length = 269
Forward primer  1        GCAACGACACCAGCACACC  19
Template        5723661  ...................  5723679

Reverse primer  1        CGCAGATGCCATAGGAGACC  20
Template        5723929  ....................  5723910
```

>CP020603.1 Pseudomonas aeruginosa strain E6130952, complete genome

```
product length = 269
Forward primer  1        GCAACGACACCAGCACACC  19
```

```
Template        6154582  ...................  6154600

Reverse primer  1        CGCAGATGCCATAGGAGACC  20
Template        6154850  ....................  6154831
```

>CP014866.1 Pseudomonas aeruginosa strain PA_154197 chromosome, complete genome

```
product length = 269
Forward primer  1        GCAACGACACCAGCACACC  19
Template        5644647  ...................  5644665

Reverse primer  1        CGCAGATGCCATAGGAGACC  20
Template        5644915  ....................  5644896
```

>LT673656.1 Pseudomonas aeruginosa isolate PcyII-10 genome assembly, chromosome: PcyII-10

```
product length = 269
Forward primer  1        GCAACGACACCAGCACACC  19
Template        5487450  ...................  5487468

Reverse primer  1        CGCAGATGCCATAGGAGACC  20
Template        5487718  ....................  5487699
```

>CP013479.1 Pseudomonas aeruginosa strain NHmuc chromosome, complete genome

```
product length = 269
Forward primer  1        GCAACGACACCAGCACACC  19
Template        5421090  ...................  5421108

Reverse primer  1        CGCAGATGCCATAGGAGACC  20
Template        5421358  ....................  5421339
```

>CP013478.1 Pseudomonas aeruginosa strain SCVJan chromosome, complete genome

```
product length = 269
Forward primer  1        GCAACGACACCAGCACACC  19
Template        5420843  ...................  5420861

Reverse primer  1        CGCAGATGCCATAGGAGACC  20
Template        5421111  ....................  5421092
```

>CP013477.1 Pseudomonas aeruginosa strain SCVFeb chromosome, complete genome

```
product length = 269
Forward primer  1        GCAACGACACCAGCACACC  19
Template        5420840  ...................  5420858

Reverse primer  1        CGCAGATGCCATAGGAGACC  20
Template        5421108  ....................  5421089
```

>CP015877.1 Pseudomonas aeruginosa SJTD-1 chromosome, complete genome

```
product length = 269
```

```
Forward primer  1        GCAACGACACCAGCACACC  19
Template        1810824  ...................  1810806

Reverse primer  1        CGCAGATGCCATAGGAGACC  20
Template        1810556  ....................  1810575
```

>OX638610.1 Pseudomonas aeruginosa strain 3541 genome assembly, chromosome: 3541

```
product length = 269
Forward primer  1        GCAACGACACCAGCACACC  19
Template        1517120  ...................  1517102

Reverse primer  1        CGCAGATGCCATAGGAGACC  20
Template        1516852  ....................  1516871
```

>CP013113.1 Pseudomonas aeruginosa strain PAER4_119 chromosome, complete genome

```
product length = 269
Forward primer  1        GCAACGACACCAGCACACC  19
Template        5694423  ...................  5694441

Reverse primer  1        CGCAGATGCCATAGGAGACC  20
Template        5694691  ....................  5694672
```

>CP017969.1 Pseudomonas aeruginosa isolate B10W chromosome, complete genome

```
product length = 269
Forward primer  1        GCAACGACACCAGCACACC  19
Template        5673566  ...................  5673584

Reverse primer  1        CGCAGATGCCATAGGAGACC  20
Template        5673834  ....................  5673815
```

>CP014999.1 Pseudomonas aeruginosa strain PA7790, complete genome

```
product length = 269
Forward primer  1        GCAACGACACCAGCACACC  19
Template        6165489  ...................  6165507

Reverse primer  1        CGCAGATGCCATAGGAGACC  20
Template        6165757  ....................  6165738
```

>CP015003.1 Pseudomonas aeruginosa strain PA11803 chromosome, complete genome

```
product length = 269
Forward primer  1        GCAACGACACCAGCACACC  19
Template        6194411  ...................  6194429

Reverse primer  1        CGCAGATGCCATAGGAGACC  20
Template        6194679  ....................  6194660
```

>CP015002.1 Pseudomonas aeruginosa strain PA8281 chromosome, complete genome

```
product length = 269
Forward primer  1        GCAACGACACCAGCACACC  19
Template        6101739  ...................  6101757

Reverse primer  1        CGCAGATGCCATAGGAGACC  20
Template        6102007  ....................  6101988
```

>CP015001.1 Pseudomonas aeruginosa strain PA1088 chromosome, complete genome

```
product length = 269
Forward primer  1        GCAACGACACCAGCACACC  19
Template        5909348  ...................  5909366

Reverse primer  1        CGCAGATGCCATAGGAGACC  20
Template        5909616  ....................  5909597
```

>CP017353.1 Pseudomonas aeruginosa strain FA-HZ1 chromosome, complete genome

```
product length = 269
Forward primer  1        GCAACGACACCAGCACACC  19
Template        1846080  ...................  1846098

Reverse primer  1        CGCAGATGCCATAGGAGACC  20
Template        1846348  ....................  1846329
```

>CP017149.1 Pseudomonas aeruginosa strain ATCC 15692, complete genome

```
product length = 269
Forward primer  1        GCAACGACACCAGCACACC  19
Template        5485586  ...................  5485604

Reverse primer  1        CGCAGATGCCATAGGAGACC  20
Template        5485854  ....................  5485835
```

>CP017099.1 Pseudomonas aeruginosa strain DN1, complete genome

```
product length = 269
Forward primer  1        GCAACGACACCAGCACACC  19
Template        5848207  ...................  5848225

Reverse primer  1        CGCAGATGCCATAGGAGACC  20
Template        5848475  ....................  5848456
```

>LT608330.1 Pseudomonas aeruginosa isolate PA14Or_reads genome assembly, chromosome: PA14OR

```
product length = 269
Forward primer  1        GCAACGACACCAGCACACC  19
Template        5749273  ...................  5749291

Reverse primer  1        CGCAGATGCCATAGGAGACC  20
Template        5749541  ....................  5749522
```

>CP011857.1 Pseudomonas aeruginosa strain ATCC 27853, complete genome

```
product length = 269
Forward primer  1        GCAACGACACCAGCACACC  19
Template        6025328  ...................  6025346

Reverse primer  1        CGCAGATGCCATAGGAGACC  20
Template        6025596  ....................  6025577
```

>CP015117.1 Pseudomonas aeruginosa strain ATCC 27853 chromosome, complete genome

```
product length = 269
Forward primer  1        GCAACGACACCAGCACACC  19
Template        2784047  ...................  2784065

Reverse primer  1        CGCAGATGCCATAGGAGACC  20
Template        2784315  ....................  2784296
```

>CP014948.1 Pseudomonas aeruginosa strain N17-1, complete genome

```
product length = 269
Forward primer  1        GCAACGACACCAGCACACC  19
Template        5571476  ...................  5571494

Reverse primer  1        CGCAGATGCCATAGGAGACC  20
Template        5571744  ....................  5571725
```

>OX638701.1 Pseudomonas aeruginosa strain 4782MK genome assembly, chromosome: 4782

```
product length = 269
Forward primer  1       GCAACGACACCAGCACACC  19
Template        355297  ...................  355315

Reverse primer  1       CGCAGATGCCATAGGAGACC  20
Template        355565  ....................  355546
```

>OX638564.1 Pseudomonas aeruginosa strain 3796A genome assembly, chromosome: 3796A

```
product length = 269
Forward primer  1        GCAACGACACCAGCACACC  19
Template        6193804  ...................  6193822

Reverse primer  1        CGCAGATGCCATAGGAGACC  20
Template        6194072  ....................  6194053
```

>CP008873.1 Pseudomonas aeruginosa strain F9670 chromosome, complete genome

```
product length = 269
Forward primer  1        GCAACGACACCAGCACACC  19
Template        2217327  ...................  2217309

Reverse primer  1        CGCAGATGCCATAGGAGACC  20
Template        2217059  ....................  2217078
```

>CP008861.1 Pseudomonas aeruginosa strain H47921 chromosome, complete genome

```
product length = 270
Forward primer  1        GCAACGACACCAGCACACC  19
Template        4681110  ...................  4681092

Reverse primer  1        CGCAGATGCCATAGGAGACC  20
Template        4680841  ....................  4680860
```

>CP012901.1 Pseudomonas aeruginosa strain N15-01092 chromosome, complete genome

```
product length = 269
Forward primer  1       GCAACGACACCAGCACACC  19
Template        811850  ...................  811832

Reverse primer  1       CGCAGATGCCATAGGAGACC  20
Template        811582  ....................  811601
```

>CP013993.1 Pseudomonas aeruginosa DHS01 chromosome, complete genome

```
product length = 269
Forward primer  1        GCAACGACACCAGCACACC  19
Template        6215990  ...................  6216008

Reverse primer  1        CGCAGATGCCATAGGAGACC  20
Template        6216258  ....................  6216239
```

>CP013989.1 Pseudomonas aeruginosa strain USDA-ARS-USMARC-41639 chromosome, complete genome

```
product length = 269
Forward primer  1        GCAACGACACCAGCACACC  19
Template        5595524  ...................  5595542

Reverse primer  1        CGCAGATGCCATAGGAGACC  20
Template        5595792  ....................  5595773
```

>CP008868.1 Pseudomonas aeruginosa strain T63266 chromosome, complete genome

```
product length = 269
Forward primer  1       GCAACGACACCAGCACACC  19
Template        851265  ...................  851283

Reverse primer  1       CGCAGATGCCATAGGAGACC  20
Template        851533  ....................  851514
```

>CP008863.1 Pseudomonas aeruginosa strain M37351 chromosome, complete genome

```
product length = 269
Forward primer  1        GCAACGACACCAGCACACC  19
Template        2759743  ...................  2759761

Reverse primer  1        CGCAGATGCCATAGGAGACC  20
```

```
Template        2760011  ....................  2759992
```

>CP008857.1 Pseudomonas aeruginosa strain F30658 chromosome, complete genome

```
product length = 269
Forward primer  1        GCAACGACACCAGCACACC  19
Template        3739880  ...................  3739898

Reverse primer  1        CGCAGATGCCATAGGAGACC  20
Template        3740148  ....................  3740129
```

>CP013696.1 Pseudomonas aeruginosa strain 12-4-4(59) chromosome, complete genome

```
product length = 269
Forward primer  1       GCAACGACACCAGCACACC  19
Template        701893  ...................  701911

Reverse primer  1       CGCAGATGCCATAGGAGACC  20
Template        702161  ....................  702142
```

>CP124672.1 Pseudomonas aeruginosa strain 2022CK-00451 chromosome, complete genome

```
product length = 269
Forward primer  1       GCAACGACACCAGCACACC  19
Template        729096  ...................  729114

Reverse primer  1       CGCAGATGCCATAGGAGACC  20
Template        729364  ....................  729345
```

>CP125367.1 Pseudomonas aeruginosa strain ZY1710 chromosome, complete genome

```
product length = 269
Forward primer  1        GCAACGACACCAGCACACC  19
Template        6201507  ...................  6201525

Reverse primer  1        CGCAGATGCCATAGGAGACC  20
Template        6201775  ....................  6201756
```

>CP125365.1 Pseudomonas aeruginosa strain ZY36 chromosome, complete genome

```
product length = 269
Forward primer  1        GCAACGACACCAGCACACC  19
Template        6201903  ...................  6201921

Reverse primer  1        CGCAGATGCCATAGGAGACC  20
Template        6202171  ....................  6202152
```

>CP125363.1 Pseudomonas aeruginosa strain ZY156 chromosome, complete genome

```
product length = 269
Forward primer  1        GCAACGACACCAGCACACC  19
Template        6200982  ...................  6201000
```

```
Reverse primer  1        CGCAGATGCCATAGGAGACC  20
Template        6201250  ....................  6201231
```

>CP125361.1 Pseudomonas aeruginosa strain ZY94 chromosome, complete genome

```
product length = 269
Forward primer  1        GCAACGACACCAGCACACC  19
Template        6190760  ...................  6190778

Reverse primer  1        CGCAGATGCCATAGGAGACC  20
Template        6191028  ....................  6191009
```

>CP125288.1 Pseudomonas aeruginosa strain SF416 chromosome, complete genome

```
product length = 269
Forward primer  1        GCAACGACACCAGCACACC  19
Template        5606920  ...................  5606938

Reverse primer  1        CGCAGATGCCATAGGAGACC  20
Template        5607188  ....................  5607169
```

>CP013144.1 Pseudomonas aeruginosa strain Cu1510 chromosome, complete genome

```
product length = 269
Forward primer  1        GCAACGACACCAGCACACC  19
Template        5107051  ...................  5107033

Reverse primer  1        CGCAGATGCCATAGGAGACC  20
Template        5106783  ....................  5106802
```

>CP013245.1 Pseudomonas aeruginosa strain VA-134 chromosome, complete genome

```
product length = 269
Forward primer  1        GCAACGACACCAGCACACC  19
Template        3016921  ...................  3016903

Reverse primer  1        CGCAGATGCCATAGGAGACC  20
Template        3016653  ....................  3016672
```

>CP123953.1 Pseudomonas aeruginosa strain 59 chromosome, complete genome

```
product length = 269
Forward primer  1        GCAACGACACCAGCACACC  19
Template        6467515  ...................  6467533

Reverse primer  1        CGCAGATGCCATAGGAGACC  20
Template        6467783  ....................  6467764
```

>AP017302.1 Pseudomonas aeruginosa DNA, complete genome, strain: IOMTU 133

```
product length = 269
Forward primer  1        GCAACGACACCAGCACACC  19
Template        6028313  ...................  6028331
```

```
Reverse primer  1        CGCAGATGCCATAGGAGACC  20
Template        6028581  ....................  6028562
```

>LN870292.1 Pseudomonas aeruginosa DK1 genome assembly Pseudomonas aeruginosa DK1 substr. NH57388A, chromosome : I

```
product length = 269
Forward primer  1        GCAACGACACCAGCACACC  19
Template        5420827  ...................  5420845

Reverse primer  1        CGCAGATGCCATAGGAGACC  20
Template        5421095  ....................  5421076
```

>CP012679.1 Pseudomonas aeruginosa strain PA1RG chromosome, complete genome

```
product length = 269
Forward primer  1        GCAACGACACCAGCACACC  19
Template        5698547  ...................  5698565

Reverse primer  1        CGCAGATGCCATAGGAGACC  20
Template        5698815  ....................  5698796
```

>CP004054.2 Pseudomonas aeruginosa PA1, complete genome

```
product length = 269
Forward primer  1        GCAACGACACCAGCACACC  19
Template        5696181  ...................  5696199

Reverse primer  1        CGCAGATGCCATAGGAGACC  20
Template        5696449  ....................  5696430
```

>CP012066.1 Pseudomonas aeruginosa strain F9676, complete genome

```
product length = 269
Forward primer  1       GCAACGACACCAGCACACC  19
Template        374920  ...................  374902

Reverse primer  1       CGCAGATGCCATAGGAGACC  20
Template        374652  ....................  374671
```

>CP118638.1 Pseudomonas aeruginosa strain P9 chromosome, complete genome

```
product length = 269
Forward primer  1        GCAACGACACCAGCACACC  19
Template        5679857  ...................  5679875

Reverse primer  1        CGCAGATGCCATAGGAGACC  20
Template        5680125  ....................  5680106
```

>CP118641.1 Pseudomonas aeruginosa strain P23 chromosome, complete genome

```
product length = 269
```

```
Forward primer  1        GCAACGACACCAGCACACC  19
Template        6102436  ...................  6102454

Reverse primer  1        CGCAGATGCCATAGGAGACC  20
Template        6102704  ....................  6102685
```

>LN871187.1 Pseudomonas aeruginosa genome assembly PAO1OR, chromosome : I

```
product length = 269
Forward primer  1        GCAACGACACCAGCACACC  19
Template        5486079  ...................  5486097

Reverse primer  1        CGCAGATGCCATAGGAGACC  20
Template        5486347  ....................  5486328
```

>AP014839.2 Pseudomonas aeruginosa DNA, complete genome, strain: 8380

```
product length = 269
Forward primer  1        GCAACGACACCAGCACACC  19
Template        5807545  ...................  5807563

Reverse primer  1        CGCAGATGCCATAGGAGACC  20
Template        5807813  ....................  5807794
```

>CP012001.1 Pseudomonas aeruginosa DSM 50071, complete genome

```
product length = 269
Forward primer  1        GCAACGACACCAGCACACC  19
Template        5520830  ...................  5520848

Reverse primer  1        CGCAGATGCCATAGGAGACC  20
Template        5521098  ....................  5521079
```

>CP011369.1 Pseudomonas aeruginosa strain S04 90 chromosome

```
product length = 269
Forward primer  1        GCAACGACACCAGCACACC  19
Template        6264221  ...................  6264239

Reverse primer  1        CGCAGATGCCATAGGAGACC  20
Template        6264489  ....................  6264470
```

>CP011317.1 Pseudomonas aeruginosa strain Carb01 63, complete genome

```
product length = 269
Forward primer  1        GCAACGACACCAGCACACC  19
Template        6631515  ...................  6631533

Reverse primer  1        CGCAGATGCCATAGGAGACC  20
Template        6631783  ....................  6631764
```

>LN831024.1 Pseudomonas aeruginosa genome assembly NCTC10332, chromosome : 1

```
product length = 269
Forward primer  1        GCAACGACACCAGCACACC  19
Template        5520293  ...................  5520311

Reverse primer  1        CGCAGATGCCATAGGAGACC  20
Template        5520561  ....................  5520542
```

>CP010555.1 Pseudomonas aeruginosa strain FRD1, complete genome

```
product length = 269
Forward primer  1        GCAACGACACCAGCACACC  19
Template        5092478  ...................  5092460

Reverse primer  1        CGCAGATGCCATAGGAGACC  20
Template        5092210  ....................  5092229
```

>CP104982.1 Pseudomonas aeruginosa PA14 isolate Gamma chromosome

```
product length = 269
Forward primer  1        GCAACGACACCAGCACACC  19
Template        5745372  ...................  5745390

Reverse primer  1        CGCAGATGCCATAGGAGACC  20
Template        5745640  ....................  5745621
```

>CP104984.1 Pseudomonas aeruginosa PA14 isolate Alpha chromosome

```
product length = 269
Forward primer  1        GCAACGACACCAGCACACC  19
Template        5745377  ...................  5745395

Reverse primer  1        CGCAGATGCCATAGGAGACC  20
Template        5745645  ....................  5745626
```

>CP104980.1 Pseudomonas aeruginosa PA14 isolate Epsilon chromosome

```
product length = 269
Forward primer  1        GCAACGACACCAGCACACC  19
Template        5745376  ...................  5745394

Reverse primer  1        CGCAGATGCCATAGGAGACC  20
Template        5745644  ....................  5745625
```

>CP104985.1 Pseudomonas aeruginosa PA14 chromosome

```
product length = 269
Forward primer  1        GCAACGACACCAGCACACC  19
Template        5745374  ...................  5745392

Reverse primer  1        CGCAGATGCCATAGGAGACC  20
Template        5745642  ....................  5745623
```

>CP104981.1 Pseudomonas aeruginosa PA14 isolate Delta chromosome

```
product length = 269
Forward primer  1        GCAACGACACCAGCACACC  19
Template        5745369  ...................  5745387

Reverse primer  1        CGCAGATGCCATAGGAGACC  20
Template        5745637  ....................  5745618
```

>CP104983.1 Pseudomonas aeruginosa PA14 isolate Beta chromosome

```
product length = 269
Forward primer  1        GCAACGACACCAGCACACC  19
Template        5745371  ...................  5745389

Reverse primer  1        CGCAGATGCCATAGGAGACC  20
Template        5745639  ....................  5745620
```

>CP104913.1 Pseudomonas aeruginosa strain PA-AUTBAM chromosome, complete genome

```
product length = 269
Forward primer  1        GCAACGACACCAGCACACC  19
Template        5474017  ...................  5474035

Reverse primer  1        CGCAGATGCCATAGGAGACC  20
Template        5474285  ....................  5474266
```

>CP104695.1 Pseudomonas aeruginosa strain 2021CK-01281 chromosome

```
product length = 269
Forward primer  1        GCAACGACACCAGCACACC  19
Template        3905031  ...................  3905049

Reverse primer  1        CGCAGATGCCATAGGAGACC  20
Template        3905299  ....................  3905280
```

>CP104720.1 Pseudomonas aeruginosa strain NY4593 chromosome, complete genome

```
product length = 269
Forward primer  1        GCAACGACACCAGCACACC  19
Template        5278771  ...................  5278789

Reverse primer  1        CGCAGATGCCATAGGAGACC  20
Template        5279039  ....................  5279020
```

>CP104590.1 Pseudomonas aeruginosa strain WTJH36 chromosome, complete genome

```
product length = 269
Forward primer  1        GCAACGACACCAGCACACC  19
Template        3637433  ...................  3637451

Reverse primer  1        CGCAGATGCCATAGGAGACC  20
Template        3637701  ....................  3637682
```

>CP104588.1 Pseudomonas aeruginosa strain WTJH32 chromosome, complete genome

```
product length = 269
Forward primer  1        GCAACGACACCAGCACACC  19
Template        6092560  ...................  6092578

Reverse primer  1        CGCAGATGCCATAGGAGACC  20
Template        6092828  ....................  6092809
```

>CP104584.1 Pseudomonas aeruginosa strain WTJH2 chromosome, complete genome

```
product length = 269
Forward primer  1        GCAACGACACCAGCACACC  19
Template        6099004  ...................  6099022

Reverse primer  1        CGCAGATGCCATAGGAGACC  20
Template        6099272  ....................  6099253
```

>CP092972.1 Pseudomonas aeruginosa strain PA01135 chromosome, complete genome

```
product length = 269
Forward primer  1        GCAACGACACCAGCACACC  19
Template        5462074  ...................  5462092

Reverse primer  1        CGCAGATGCCATAGGAGACC  20
Template        5462342  ....................  5462323
```

>CP079758.1 Pseudomonas aeruginosa strain PA0386 chromosome, complete genome

```
product length = 269
Forward primer  1        GCAACGACACCAGCACACC  19
Template        5456492  ...................  5456510

Reverse primer  1        CGCAGATGCCATAGGAGACC  20
Template        5456760  ....................  5456741
```

>CP079757.1 Pseudomonas aeruginosa strain PA0200 chromosome, complete genome

```
product length = 269
Forward primer  1        GCAACGACACCAGCACACC  19
Template        5469712  ...................  5469730

Reverse primer  1        CGCAGATGCCATAGGAGACC  20
Template        5469980  ....................  5469961
```

>CP104301.1 Pseudomonas aeruginosa strain PLL01 chromosome, complete genome

```
product length = 269
Forward primer  1        GCAACGACACCAGCACACC  19
Template        5474855  ...................  5474873

Reverse primer  1        CGCAGATGCCATAGGAGACC  20
Template        5475123  ....................  5475104
```

>AP014651.1 Pseudomonas aeruginosa DNA, complete genome, strain: NCGM257

```
product length = 269
Forward primer  1        GCAACGACACCAGCACACC  19
Template        6224639  ...................  6224657

Reverse primer  1        CGCAGATGCCATAGGAGACC  20
Template        6224907  ....................  6224888
```

>CP007399.1 Pseudomonas aeruginosa strain F22031, complete genome

```
product length = 269
Forward primer  1        GCAACGACACCAGCACACC  19
Template        1731968  ...................  1731986

Reverse primer  1        CGCAGATGCCATAGGAGACC  20
Template        1732236  ....................  1732217
```

>AP014646.1 Pseudomonas aeruginosa DNA, complete genome, strain: NCGM 1984

```
product length = 269
Forward primer  1        GCAACGACACCAGCACACC  19
Template        5926913  ...................  5926931

Reverse primer  1        CGCAGATGCCATAGGAGACC  20
Template        5927181  ....................  5927162
```

>HG974234.1 Pseudomonas aeruginosa strain PSE305, genome

```
product length = 269
Forward primer  1        GCAACGACACCAGCACACC  19
Template        5033737  ...................  5033755

Reverse primer  1        CGCAGATGCCATAGGAGACC  20
Template        5034005  ....................  5033986
```

>CP089068.2 Pseudomonas aeruginosa strain UNC_PaerCF13 chromosome, complete genome

```
product length = 269
Forward primer  1       GCAACGACACCAGCACACC  19
Template        708693  ...................  708711

Reverse primer  1       CGCAGATGCCATAGGAGACC  20
Template        708961  ....................  708942
```

>CP069337.1 Pseudomonas aeruginosa strain E04 chromosome, complete genome

```
product length = 269
Forward primer  1        GCAACGACACCAGCACACC  19
Template        5611641  ...................  5611659

Reverse primer  1        CGCAGATGCCATAGGAGACC  20
Template        5611909  ....................  5611890
```

>CP069336.1 Pseudomonas aeruginosa strain E01 chromosome, complete genome

```
product length = 269
Forward primer  1        GCAACGACACCAGCACACC  19
Template        5523604  ...................  5523622

Reverse primer  1        CGCAGATGCCATAGGAGACC  20
Template        5523872  ....................  5523853
```

>CP069335.1 Pseudomonas aeruginosa strain E02 chromosome, complete genome

```
product length = 269
Forward primer  1        GCAACGACACCAGCACACC  19
Template        5537839  ...................  5537857

Reverse primer  1        CGCAGATGCCATAGGAGACC  20
Template        5538107  ....................  5538088
```

>CP069334.1 Pseudomonas aeruginosa strain E03 chromosome, complete genome

```
product length = 269
Forward primer  1        GCAACGACACCAGCACACC  19
Template        5523605  ...................  5523623

Reverse primer  1        CGCAGATGCCATAGGAGACC  20
Template        5523873  ....................  5523854
```

>CP069333.1 Pseudomonas aeruginosa strain R01 chromosome, complete genome

```
product length = 269
Forward primer  1        GCAACGACACCAGCACACC  19
Template        5477939  ...................  5477957

Reverse primer  1        CGCAGATGCCATAGGAGACC  20
Template        5478207  ....................  5478188
```

>CP069330.1 Pseudomonas aeruginosa strain R07 chromosome, complete genome

```
product length = 269
Forward primer  1        GCAACGACACCAGCACACC  19
Template        5478139  ...................  5478157

Reverse primer  1        CGCAGATGCCATAGGAGACC  20
Template        5478407  ....................  5478388
```

>CP069328.1 Pseudomonas aeruginosa strain R04 chromosome, complete genome

```
product length = 269
Forward primer  1        GCAACGACACCAGCACACC  19
Template        5477628  ...................  5477646

Reverse primer  1        CGCAGATGCCATAGGAGACC  20
Template        5477896  ....................  5477877
```

>CP069327.1 Pseudomonas aeruginosa strain R03 chromosome, complete genome

```
product length = 269
Forward primer  1        GCAACGACACCAGCACACC  19
Template        5476748  ...................  5476766

Reverse primer  1        CGCAGATGCCATAGGAGACC  20
Template        5477016  ....................  5476997
```

>CP069326.1 Pseudomonas aeruginosa strain R11 chromosome, complete genome

```
product length = 269
Forward primer  1        GCAACGACACCAGCACACC  19
Template        5477898  ...................  5477916

Reverse primer  1        CGCAGATGCCATAGGAGACC  20
Template        5478166  ....................  5478147
```

>CP069324.1 Pseudomonas aeruginosa strain R05 chromosome, complete genome

```
product length = 269
Forward primer  1        GCAACGACACCAGCACACC  19
Template        5463448  ...................  5463466

Reverse primer  1        CGCAGATGCCATAGGAGACC  20
Template        5463716  ....................  5463697
```

>CP069323.1 Pseudomonas aeruginosa strain R06 chromosome, complete genome

```
product length = 269
Forward primer  1        GCAACGACACCAGCACACC  19
Template        5477443  ...................  5477461

Reverse primer  1        CGCAGATGCCATAGGAGACC  20
Template        5477711  ....................  5477692
```

>CP068678.1 Pseudomonas aeruginosa strain NCCP15783 chromosome, complete genome

```
product length = 269
Forward primer  1       GCAACGACACCAGCACACC  19
Template        661613  ...................  661631

Reverse primer  1       CGCAGATGCCATAGGAGACC  20
Template        661881  ....................  661862
```

>CP008739.2 Pseudomonas aeruginosa VRFPA04, complete genome

```
product length = 269
Forward primer  1       GCAACGACACCAGCACACC  19
Template        698418  ...................  698400

Reverse primer  1       CGCAGATGCCATAGGAGACC  20
Template        698150  ....................  698169
```

>CP054794.1 Pseudomonas aeruginosa strain A0002 chromosome, complete genome

```
product length = 269
Forward primer  1        GCAACGACACCAGCACACC  19
Template        5752932  ...................  5752950

Reverse primer  1        CGCAGATGCCATAGGAGACC  20
Template        5753200  ....................  5753181
```

>CP054793.1 Pseudomonas aeruginosa strain SE5452 chromosome, complete genome

```
product length = 269
Forward primer  1        GCAACGACACCAGCACACC  19
Template        5650471  ...................  5650489

Reverse primer  1        CGCAGATGCCATAGGAGACC  20
Template        5650739  ....................  5650720
```

>CP054792.1 Pseudomonas aeruginosa strain SE5431 chromosome, complete genome

```
product length = 269
Forward primer  1        GCAACGACACCAGCACACC  19
Template        5811462  ...................  5811480

Reverse primer  1        CGCAGATGCCATAGGAGACC  20
Template        5811730  ....................  5811711
```

>CP054791.1 Pseudomonas aeruginosa strain SE5430 chromosome, complete genome

```
product length = 269
Forward primer  1        GCAACGACACCAGCACACC  19
Template        5943849  ...................  5943867

Reverse primer  1        CGCAGATGCCATAGGAGACC  20
Template        5944117  ....................  5944098
```

>CP054790.1 Pseudomonas aeruginosa strain SE5418 chromosome, complete genome

```
product length = 269
Forward primer  1        GCAACGACACCAGCACACC  19
Template        6094739  ...................  6094757

Reverse primer  1        CGCAGATGCCATAGGAGACC  20
Template        6095007  ....................  6094988
```

>CP054789.1 Pseudomonas aeruginosa strain SE5381 chromosome, complete genome

```
product length = 269
Forward primer  1        GCAACGACACCAGCACACC  19
Template        5934674  ...................  5934692

Reverse primer  1        CGCAGATGCCATAGGAGACC  20
Template        5934942  ....................  5934923
```

>CP054788.1 Pseudomonas aeruginosa strain YTSY4 chromosome, complete genome

```
product length = 269
Forward primer  1        GCAACGACACCAGCACACC  19
Template        5930772  ...................  5930790

Reverse primer  1        CGCAGATGCCATAGGAGACC  20
Template        5931040  ....................  5931021
```

>CP054787.1 Pseudomonas aeruginosa strain HB2011305RE chromosome, complete genome

```
product length = 269
Forward primer  1        GCAACGACACCAGCACACC  19
Template        5982065  ...................  5982083

Reverse primer  1        CGCAGATGCCATAGGAGACC  20
Template        5982333  ....................  5982314
```

>CP054786.1 Pseudomonas aeruginosa strain DL201330 chromosome, complete genome

```
product length = 269
Forward primer  1        GCAACGACACCAGCACACC  19
Template        3643818  ...................  3643836

Reverse primer  1        CGCAGATGCCATAGGAGACC  20
Template        3644086  ....................  3644067
```

>CP086122.1 Pseudomonas aeruginosa strain MIN-155 chromosome, complete genome

```
product length = 269
Forward primer  1        GCAACGACACCAGCACACC  19
Template        5484300  ...................  5484318

Reverse primer  1        CGCAGATGCCATAGGAGACC  20
Template        5484568  ....................  5484549
```

>CP081477.2 Pseudomonas aeruginosa strain P8W chromosome, complete genome

```
product length = 269
Forward primer  1        GCAACGACACCAGCACACC  19
Template        5423695  ...................  5423713

Reverse primer  1        CGCAGATGCCATAGGAGACC  20
Template        5423963  ....................  5423944
```

>CP053747.1 Pseudomonas aeruginosa strain Pae1255-NDM1 chromosome, complete genome

```
product length = 269
Forward primer  1        GCAACGACACCAGCACACC  19
Template        6089298  ...................  6089316

Reverse primer  1        CGCAGATGCCATAGGAGACC  20
Template        6089566  ....................  6089547
```

>CP089063.2 Pseudomonas aeruginosa strain UNC_PaerCF37 chromosome, complete genome

```
product length = 269
Forward primer  1        GCAACGACACCAGCACACC  19
Template        5448496  ...................  5448514

Reverse primer  1        CGCAGATGCCATAGGAGACC  20
Template        5448764  ....................  5448745
```

>CP089064.2 Pseudomonas aeruginosa strain UNC_PaerCF35 chromosome, complete genome

```
product length = 269
Forward primer  1        GCAACGACACCAGCACACC  19
Template        2010051  ...................  2010069

Reverse primer  1        CGCAGATGCCATAGGAGACC  20
Template        2010319  ....................  2010300
```

>CP089062.2 Pseudomonas aeruginosa strain UNC_PaerCF38 chromosome, complete genome

```
product length = 269
Forward primer  1       GCAACGACACCAGCACACC  19
Template        374032  ...................  374050

Reverse primer  1       CGCAGATGCCATAGGAGACC  20
Template        374300  ....................  374281
```

>CP089061.2 Pseudomonas aeruginosa strain UNC_PaerCF41 chromosome, complete genome

```
product length = 269
Forward primer  1        GCAACGACACCAGCACACC  19
Template        2100170  ...................  2100188

Reverse primer  1        CGCAGATGCCATAGGAGACC  20
Template        2100438  ....................  2100419
```

>AP014622.1 Pseudomonas aeruginosa DNA, complete genome, strain: NCGM 1900

```
product length = 269
Forward primer  1        GCAACGACACCAGCACACC  19
Template        5926897  ...................  5926915

Reverse primer  1        CGCAGATGCCATAGGAGACC  20
Template        5927165  ....................  5927146
```

>CP008749.1 Pseudomonas aeruginosa PAO1H2O genome

```
product length = 269
Forward primer  1        GCAACGACACCAGCACACC  19
Template        5474017  ...................  5474035

Reverse primer  1        CGCAGATGCCATAGGAGACC  20
Template        5474285  ....................  5474266
```

>CP007147.1 Pseudomonas aeruginosa YL84, complete genome

```
product length = 269
Forward primer  1        GCAACGACACCAGCACACC  19
Template        1631322  ...................  1631304

Reverse primer  1        CGCAGATGCCATAGGAGACC  20
Template        1631054  ....................  1631073
```

>CP081202.1 Pseudomonas aeruginosa strain P9W chromosome, complete genome

```
product length = 269
Forward primer  1        GCAACGACACCAGCACACC  19
Template        6470180  ...................  6470162

Reverse primer  1        CGCAGATGCCATAGGAGACC  20
Template        6469912  ....................  6469931
```

>CP082821.1 Pseudomonas aeruginosa strain SCAID PLC1-2021 (16/222) chromosome, complete genome

```
product length = 269
Forward primer  1        GCAACGACACCAGCACACC  19
Template        6330211  ...................  6330229

Reverse primer  1        CGCAGATGCCATAGGAGACC  20
Template        6330479  ....................  6330460
```

>CP082822.1 Pseudomonas aeruginosa strain SCAID WND1-2021 (9/195) chromosome, complete genome

```
product length = 269
Forward primer  1        GCAACGACACCAGCACACC  19
Template        6301394  ...................  6301412

Reverse primer  1        CGCAGATGCCATAGGAGACC  20
Template        6301662  ....................  6301643
```

>CP082823.1 Pseudomonas aeruginosa strain SCAID TST-2021 (7/157) chromosome, complete genome

```
product length = 269
Forward primer  1        GCAACGACACCAGCACACC  19
Template        6378626  ...................  6378644

Reverse primer  1        CGCAGATGCCATAGGAGACC  20
Template        6378894  ....................  6378875
```

>CP078009.1 Pseudomonas aeruginosa strain FAHZU31 chromosome, complete genome

```
product length = 269
Forward primer  1        GCAACGACACCAGCACACC  19
Template        5805591  ...................  5805609

Reverse primer  1        CGCAGATGCCATAGGAGACC  20
```

```
Template        5805859  ....................  5805840
```

>CP078007.1 Pseudomonas aeruginosa strain FAHZU40 chromosome, complete genome

```
product length = 269
Forward primer  1        GCAACGACACCAGCACACC  19
Template        5765141  ...................  5765159

Reverse primer  1        CGCAGATGCCATAGGAGACC  20
Template        5765409  ....................  5765390
```

>CP078006.1 Pseudomonas aeruginosa strain NDTH7329 chromosome, complete genome

```
product length = 269
Forward primer  1        GCAACGACACCAGCACACC  19
Template        6330925  ...................  6330943

Reverse primer  1        CGCAGATGCCATAGGAGACC  20
Template        6331193  ....................  6331174
```

>CP078004.1 Pseudomonas aeruginosa strain QZPH16 chromosome, complete genome

```
product length = 269
Forward primer  1        GCAACGACACCAGCACACC  19
Template        6156087  ...................  6156105

Reverse primer  1        CGCAGATGCCATAGGAGACC  20
Template        6156355  ....................  6156336
```

>CP078002.1 Pseudomonas aeruginosa strain QZPH21 chromosome, complete genome

```
product length = 269
Forward primer  1        GCAACGACACCAGCACACC  19
Template        5951596  ...................  5951614

Reverse primer  1        CGCAGATGCCATAGGAGACC  20
Template        5951864  ....................  5951845
```

>CP077999.1 Pseudomonas aeruginosa strain SRRSH1120 chromosome, complete genome

```
product length = 269
Forward primer  1        GCAACGACACCAGCACACC  19
Template        6162487  ...................  6162505

Reverse primer  1        CGCAGATGCCATAGGAGACC  20
Template        6162755  ....................  6162736
```

>CP077997.1 Pseudomonas aeruginosa strain SRRSH1521 chromosome, complete genome

```
product length = 269
Forward primer  1        GCAACGACACCAGCACACC  19
Template        5730129  ...................  5730147
```

```
Reverse primer  1        CGCAGATGCCATAGGAGACC  20
Template        5730397  ....................  5730378
```

>CP077994.1 Pseudomonas aeruginosa strain SRRSH2790 chromosome, complete genome

```
product length = 269
Forward primer  1        GCAACGACACCAGCACACC  19
Template        6154958  ...................  6154976

Reverse primer  1        CGCAGATGCCATAGGAGACC  20
Template        6155226  ....................  6155207
```

>CP077981.1 Pseudomonas aeruginosa strain ZPPH14 chromosome, complete genome

```
product length = 269
Forward primer  1        GCAACGACACCAGCACACC  19
Template        5592048  ...................  5592066

Reverse primer  1        CGCAGATGCCATAGGAGACC  20
Template        5592316  ....................  5592297
```

>CP077977.1 Pseudomonas aeruginosa strain ZPPH29 chromosome, complete genome

```
product length = 269
Forward primer  1        GCAACGACACCAGCACACC  19
Template        5800188  ...................  5800206

Reverse primer  1        CGCAGATGCCATAGGAGACC  20
Template        5800456  ....................  5800437
```

>CP064403.1 Pseudomonas aeruginosa strain WTJH12 chromosome, complete genome

```
product length = 269
Forward primer  1        GCAACGACACCAGCACACC  19
Template        5756519  ...................  5756537

Reverse primer  1        CGCAGATGCCATAGGAGACC  20
Template        5756787  ....................  5756768
```

>CP064401.1 Pseudomonas aeruginosa strain NDTH10366 chromosome, complete genome

```
product length = 269
Forward primer  1        GCAACGACACCAGCACACC  19
Template        6159119  ...................  6159137

Reverse primer  1        CGCAGATGCCATAGGAGACC  20
Template        6159387  ....................  6159368
```

>CP064399.1 Pseudomonas aeruginosa strain QZPH41 chromosome, complete genome

```
product length = 269
Forward primer  1        GCAACGACACCAGCACACC  19
Template        5753264  ...................  5753282
```

```
Reverse primer  1        CGCAGATGCCATAGGAGACC  20
Template        5753532  ....................  5753513
```

>CP064397.1 Pseudomonas aeruginosa strain SRRSH1002 chromosome, complete genome

```
product length = 269
Forward primer  1        GCAACGACACCAGCACACC  19
Template        6111611  ...................  6111629

Reverse primer  1        CGCAGATGCCATAGGAGACC  20
Template        6111879  ....................  6111860
```

>CP064395.1 Pseudomonas aeruginosa strain SRRSH1408 chromosome, complete genome

```
product length = 269
Forward primer  1        GCAACGACACCAGCACACC  19
Template        6098473  ...................  6098491

Reverse primer  1        CGCAGATGCCATAGGAGACC  20
Template        6098741  ....................  6098722
```

>CP064393.1 Pseudomonas aeruginosa strain SRRSH1101 chromosome, complete genome

```
product length = 269
Forward primer  1        GCAACGACACCAGCACACC  19
Template        6005652  ...................  6005670

Reverse primer  1        CGCAGATGCCATAGGAGACC  20
Template        6005920  ....................  6005901
```

>CP064392.1 Pseudomonas aeruginosa strain SRRSH15 chromosome, complete genome

```
product length = 269
Forward primer  1        GCAACGACACCAGCACACC  19
Template        5815436  ...................  5815454

Reverse primer  1        CGCAGATGCCATAGGAGACC  20
Template        5815704  ....................  5815685
```

>CP006985.1 Pseudomonas aeruginosa LESlike4 sequence

```
product length = 269
Forward primer  1        GCAACGACACCAGCACACC  19
Template        5728621  ...................  5728639

Reverse primer  1        CGCAGATGCCATAGGAGACC  20
Template        5728889  ....................  5728870
```

>CP006984.1 Pseudomonas aeruginosa LESlike1 chromosome

```
product length = 269
Forward primer  1        GCAACGACACCAGCACACC  19
```

```
Template        5713653  ...................  5713671

Reverse primer  1        CGCAGATGCCATAGGAGACC  20
Template        5713921  ....................  5713902
```

>CP006983.1 Pseudomonas aeruginosa LESB65 sequence

```
product length = 269
Forward primer  1        GCAACGACACCAGCACACC  19
Template        5731574  ...................  5731592

Reverse primer  1        CGCAGATGCCATAGGAGACC  20
Template        5731842  ....................  5731823
```

>CP006982.1 Pseudomonas aeruginosa LES400 sequence

```
product length = 269
Forward primer  1        GCAACGACACCAGCACACC  19
Template        5795899  ...................  5795917

Reverse primer  1        CGCAGATGCCATAGGAGACC  20
Template        5796167  ....................  5796148
```

>CP006981.1 Pseudomonas aeruginosa LESlike7 sequence

```
product length = 269
Forward primer  1        GCAACGACACCAGCACACC  19
Template        5672481  ...................  5672499

Reverse primer  1        CGCAGATGCCATAGGAGACC  20
Template        5672749  ....................  5672730
```

>CP006980.1 Pseudomonas aeruginosa LESlike5 sequence

```
product length = 269
Forward primer  1        GCAACGACACCAGCACACC  19
Template        5747916  ...................  5747934

Reverse primer  1        CGCAGATGCCATAGGAGACC  20
Template        5748184  ....................  5748165
```

>HG530068.1 Pseudomonas aeruginosa PA38182, complete genome

```
product length = 269
Forward primer  1        GCAACGACACCAGCACACC  19
Template        5660574  ...................  5660592

Reverse primer  1        CGCAGATGCCATAGGAGACC  20
Template        5660842  ....................  5660823
```

>CP006931.1 Pseudomonas aeruginosa SCV20265, complete genome

```
product length = 269
```

```
Forward primer  1        GCAACGACACCAGCACACC  19
Template        5887422  ...................  5887440

Reverse primer  1        CGCAGATGCCATAGGAGACC  20
Template        5887690  ....................  5887671
```

>CP006937.1 Pseudomonas aeruginosa LES431, complete genome

```
product length = 269
Forward primer  1        GCAACGACACCAGCACACC  19
Template        5754655  ...................  5754673

Reverse primer  1        CGCAGATGCCATAGGAGACC  20
Template        5754923  ....................  5754904
```

>CP006853.1 Pseudomonas aeruginosa MTB-1, complete genome

```
product length = 269
Forward primer  1        GCAACGACACCAGCACACC  19
Template        5739145  ...................  5739163

Reverse primer  1        CGCAGATGCCATAGGAGACC  20
Template        5739413  ....................  5739394
```

>CP004055.1 Pseudomonas aeruginosa PA1R, complete genome

```
product length = 269
Forward primer  1        GCAACGACACCAGCACACC  19
Template        3117547  ...................  3117565

Reverse primer  1        CGCAGATGCCATAGGAGACC  20
Template        3117815  ....................  3117796
```

>CP081345.1 Pseudomonas aeruginosa strain F291007 chromosome, complete genome

```
product length = 269
Forward primer  1        GCAACGACACCAGCACACC  19
Template        6081990  ...................  6082008

Reverse primer  1        CGCAGATGCCATAGGAGACC  20
Template        6082258  ....................  6082239
```

>CP081346.1 Pseudomonas aeruginosa strain SE5419 chromosome, complete genome

```
product length = 269
Forward primer  1        GCAACGACACCAGCACACC  19
Template        5917557  ...................  5917575

Reverse primer  1        CGCAGATGCCATAGGAGACC  20
Template        5917825  ....................  5917806
```

>CP006832.1 Pseudomonas aeruginosa PAO1-VE13 genome

```
product length = 269
Forward primer  1        GCAACGACACCAGCACACC  19
Template        5474012  ...................  5474030

Reverse primer  1        CGCAGATGCCATAGGAGACC  20
Template        5474280  ....................  5474261
```

>CP006831.1 Pseudomonas aeruginosa PAO1-VE2 genome

```
product length = 269
Forward primer  1        GCAACGACACCAGCACACC  19
Template        5475097  ...................  5475115

Reverse primer  1        CGCAGATGCCATAGGAGACC  20
Template        5475365  ....................  5475346
```

>CP006705.1 Pseudomonas aeruginosa PAO581 genome

```
product length = 269
Forward primer  1        GCAACGACACCAGCACACC  19
Template        5253598  ...................  5253616

Reverse primer  1        CGCAGATGCCATAGGAGACC  20
Template        5253866  ....................  5253847
```

>CP006728.1 Pseudomonas aeruginosa c7447m genome

```
product length = 269
Forward primer  1        GCAACGACACCAGCACACC  19
Template        5471819  ...................  5471837

Reverse primer  1        CGCAGATGCCATAGGAGACC  20
Template        5472087  ....................  5472068
```

>CP006245.1 Pseudomonas aeruginosa RP73, complete genome

```
product length = 269
Forward primer  1        GCAACGACACCAGCACACC  19
Template        5544820  ...................  5544838

Reverse primer  1        CGCAGATGCCATAGGAGACC  20
Template        5545088  ....................  5545069
```

>CP074424.1 Pseudomonas aeruginosa strain 88A chromosome

```
product length = 268
Forward primer  1        GCAACGACACCAGCACACC  19
Template        3079909  ...................  3079927

Reverse primer  1        CGCAGATGCCATAGGAGACC  20
Template        3080176  ....................  3080157
```

>CP061780.1 Pseudomonas aeruginosa strain ZBX-P11 chromosome, complete genome

```
product length = 269
Forward primer  1        GCAACGACACCAGCACACC  19
Template        1775931  ...................  1775949

Reverse primer  1        CGCAGATGCCATAGGAGACC  20
Template        1776199  ....................  1776180
```

>CP061779.1 Pseudomonas aeruginosa strain ZBX-P12 chromosome, complete genome

```
product length = 269
Forward primer  1        GCAACGACACCAGCACACC  19
Template        5098802  ...................  5098820

Reverse primer  1        CGCAGATGCCATAGGAGACC  20
Template        5099070  ....................  5099051
```

>CP061778.1 Pseudomonas aeruginosa strain ZBX-P13 chromosome, complete genome

```
product length = 269
Forward primer  1       GCAACGACACCAGCACACC  19
Template        680125  ...................  680143

Reverse primer  1       CGCAGATGCCATAGGAGACC  20
Template        680393  ....................  680374
```

>CP061777.1 Pseudomonas aeruginosa strain ZBX-P23 chromosome, complete genome

```
product length = 269
Forward primer  1        GCAACGACACCAGCACACC  19
Template        5940151  ...................  5940169

Reverse primer  1        CGCAGATGCCATAGGAGACC  20
Template        5940419  ....................  5940400
```

>CP004061.1 Pseudomonas aeruginosa B136-33, complete genome

```
product length = 269
Forward primer  1        GCAACGACACCAGCACACC  19
Template        5626744  ...................  5626762

Reverse primer  1        CGCAGATGCCATAGGAGACC  20
Template        5627012  ....................  5626993
```

>CP070355.1 Pseudomonas aeruginosa strain PDNC003 chromosome

```
product length = 269
Forward primer  1        GCAACGACACCAGCACACC  19
Template        4342891  ...................  4342909

Reverse primer  1        CGCAGATGCCATAGGAGACC  20
Template        4343159  ....................  4343140
```

>CP003149.1 Pseudomonas aeruginosa DK2, complete genome

```
product length = 269
Forward primer  1        GCAACGACACCAGCACACC  19
Template        5574107  ...................  5574125

Reverse primer  1        CGCAGATGCCATAGGAGACC  20
Template        5574375  ....................  5574356
```

>CP063237.1 Pseudomonas aeruginosa strain mPA08-31 chromosome

```
product length = 269
Forward primer  1        GCAACGACACCAGCACACC  19
Template        5655496  ...................  5655514

Reverse primer  1        CGCAGATGCCATAGGAGACC  20
Template        5655764  ....................  5655745
```

>CP062219.1 Pseudomonas aeruginosa strain JT86 chromosome, complete genome

```
product length = 269
Forward primer  1        GCAACGACACCAGCACACC  19
Template        3960842  ...................  3960860

Reverse primer  1        CGCAGATGCCATAGGAGACC  20
Template        3961110  ....................  3961091
```

>CP061850.1 Pseudomonas aeruginosa strain R31 chromosome, complete genome

```
product length = 269
Forward primer  1        GCAACGACACCAGCACACC  19
Template        5394735  ...................  5394753

Reverse primer  1        CGCAGATGCCATAGGAGACC  20
Template        5395003  ....................  5394984
```

>CP058257.1 Pseudomonas aeruginosa strain PA179 chromosome

```
product length = 269
Forward primer  1        GCAACGACACCAGCACACC  19
Template        5719916  ...................  5719934

Reverse primer  1        CGCAGATGCCATAGGAGACC  20
Template        5720184  ....................  5720165
```

>AP012280.1 Pseudomonas aeruginosa NCGM2.S1 DNA, complete genome

```
product length = 269
Forward primer  1       GCAACGACACCAGCACACC  19
Template        723934  ...................  723916

Reverse primer  1       CGCAGATGCCATAGGAGACC  20
Template        723666  ....................  723685
```

>CP002496.1 Pseudomonas aeruginosa M18, complete genome

```
product length = 269
Forward primer  1        GCAACGACACCAGCACACC  19
Template        5525207  ...................  5525225

Reverse primer  1        CGCAGATGCCATAGGAGACC  20
Template        5525475  ....................  5525456
```

>FM209186.1 Pseudomonas aeruginosa LESB58 complete genome sequence

```
product length = 269
Forward primer  1        GCAACGACACCAGCACACC  19
Template        5806323  ...................  5806341

Reverse primer  1        CGCAGATGCCATAGGAGACC  20
Template        5806591  ....................  5806572
```

>CP000438.1 Pseudomonas aeruginosa UCBPP-PA14, complete genome

```
product length = 269
Forward primer  1        GCAACGACACCAGCACACC  19
Template        5745425  ...................  5745443

Reverse primer  1        CGCAGATGCCATAGGAGACC  20
Template        5745693  ....................  5745674
```

>AE004091.2 Pseudomonas aeruginosa PAO1, complete genome

```
product length = 269
Forward primer  1        GCAACGACACCAGCACACC  19
Template        5474017  ...................  5474035

Reverse primer  1        CGCAGATGCCATAGGAGACC  20
Template        5474285  ....................  5474266
```

>AY658535.1 Synthetic construct Peudomonas aeruginosa clone FLH035136.01F PA4878 gene, partial cds

```
product length = 269
Forward primer  1    GCAACGACACCAGCACACC  19
Template        252  ...................  270

Reverse primer  1    CGCAGATGCCATAGGAGACC  20
Template        520  ....................  501
```

>CP109850.1 Pseudomonas aeruginosa strain PALA37 chromosome, complete genome

```
product length = 269
Forward primer  1        GCAACGACACCAGCACACC  19
Template        3238653  .....A.............  3238635

Reverse primer  1        CGCAGATGCCATAGGAGACC  20
Template        3238385  ....................  3238404
```

>CP096958.1 Pseudomonas aeruginosa strain NY11210 chromosome, complete genome

```
product length = 269
Forward primer  1        GCAACGACACCAGCACACC  19
Template        6062369  ...................  6062387

Reverse primer  1        CGCAGATGCCATAGGAGACC  20
Template        6062637  ..........G.........  6062618
```

>CP096953.1 Pseudomonas aeruginosa strain NY5535 chromosome, complete genome

```
product length = 269
Forward primer  1        GCAACGACACCAGCACACC  19
Template        6013799  ...................  6013817

Reverse primer  1        CGCAGATGCCATAGGAGACC  20
Template        6014067  ..........G.........  6014048
```

>CP096946.1 Pseudomonas aeruginosa strain NY5530 chromosome, complete genome

```
product length = 269
Forward primer  1        GCAACGACACCAGCACACC  19
Template        6048102  ...................  6048120

Reverse primer  1        CGCAGATGCCATAGGAGACC  20
Template        6048370  ..........G.........  6048351
```

>CP096937.1 Pseudomonas aeruginosa strain NY5520 chromosome, complete genome

```
product length = 269
Forward primer  1        GCAACGACACCAGCACACC  19
Template        5788337  ...................  5788355

Reverse primer  1        CGCAGATGCCATAGGAGACC  20
Template        5788605  ..........G.........  5788586
```

>CP096934.1 Pseudomonas aeruginosa strain NY5511 chromosome, complete genome

```
product length = 269
Forward primer  1        GCAACGACACCAGCACACC  19
Template        6037769  ...................  6037787

Reverse primer  1        CGCAGATGCCATAGGAGACC  20
Template        6038037  ..........G.........  6038018
```

>CP096932.1 Pseudomonas aeruginosa strain NY5510 chromosome, complete genome

```
product length = 269
Forward primer  1        GCAACGACACCAGCACACC  19
Template        5941389  ...................  5941407

Reverse primer  1        CGCAGATGCCATAGGAGACC  20
Template        5941657  ..........G.........  5941638
```

>CP096929.1 Pseudomonas aeruginosa strain NY5507 chromosome, complete genome

```
product length = 269
Forward primer  1        GCAACGACACCAGCACACC  19
Template        6048772  ...................  6048790

Reverse primer  1        CGCAGATGCCATAGGAGACC  20
Template        6049040  ..........G.........  6049021
```

>CP124658.1 Pseudomonas aeruginosa strain 2022CK-00068 chromosome, complete genome

```
product length = 269
Forward primer  1        GCAACGACACCAGCACACC  19
Template        6077100  ...................  6077118

Reverse primer  1        CGCAGATGCCATAGGAGACC  20
Template        6077368  ..........G.........  6077349
```

>CP124664.1 Pseudomonas aeruginosa strain 2021CK-01256 chromosome, complete genome

```
product length = 269
Forward primer  1        GCAACGACACCAGCACACC  19
Template        5752262  ...................  5752280

Reverse primer  1        CGCAGATGCCATAGGAGACC  20
Template        5752530  ..........G.........  5752511
```

>CP119298.1 Pseudomonas aeruginosa strain SNDPR-01 chromosome, complete genome

```
product length = 269
Forward primer  1        GCAACGACACCAGCACACC  19
Template        5593779  ...................  5593797

Reverse primer  1        CGCAGATGCCATAGGAGACC  20
Template        5594047  T...................  5594028
```

>CP117974.1 Pseudomonas aeruginosa strain B-3509 chromosome, complete genome

```
product length = 269
Forward primer  1        GCAACGACACCAGCACACC  19
Template        6336972  ...................  6336954

Reverse primer  1        CGCAGATGCCATAGGAGACC  20
Template        6336704  ..........G.........  6336723
```

>CP075834.1 Pseudomonas aeruginosa strain PaLo15 chromosome, complete genome

```
product length = 269
Forward primer  1        GCAACGACACCAGCACACC  19
Template        1311853  ...................  1311835

Reverse primer  1        CGCAGATGCCATAGGAGACC  20
Template        1311585  ...................G  1311604
```

>CP075817.1 Pseudomonas aeruginosa strain PaLo39 chromosome, complete genome

```
product length = 269
Forward primer  1        GCAACGACACCAGCACACC  19
Template        1351426  ...................  1351408

Reverse primer  1        CGCAGATGCCATAGGAGACC  20
Template        1351158  ..........G.........  1351177
```

>CP075829.1 Pseudomonas aeruginosa strain PaLo25 chromosome, complete genome

```
product length = 269
Forward primer  1        GCAACGACACCAGCACACC  19
Template        5470335  ...................  5470353

Reverse primer  1        CGCAGATGCCATAGGAGACC  20
Template        5470603  ...................G  5470584
```

>CP075826.1 Pseudomonas aeruginosa strain PaLo29 chromosome, complete genome

```
product length = 269
Forward primer  1        GCAACGACACCAGCACACC  19
Template        5858512  ...................  5858530

Reverse primer  1        CGCAGATGCCATAGGAGACC  20
Template        5858780  ..........G.........  5858761
```

>CP075814.1 Pseudomonas aeruginosa strain PaLo44 chromosome, complete genome

```
product length = 269
Forward primer  1        GCAACGACACCAGCACACC  19
Template        5513197  ...................  5513215

Reverse primer  1        CGCAGATGCCATAGGAGACC  20
Template        5513465  ..........G.........  5513446
```

>CP075808.1 Pseudomonas aeruginosa strain PaLo185 chromosome

```
product length = 269
Forward primer  1        GCAACGACACCAGCACACC  19
Template        6201543  ...................  6201561

Reverse primer  1        CGCAGATGCCATAGGAGACC  20
Template        6201811  ..........G.........  6201792
```

>CP075807.1 Pseudomonas aeruginosa strain PaLo191 chromosome, complete genome

```
product length = 269
Forward primer  1        GCAACGACACCAGCACACC  19
Template        5678441  ...................  5678459

Reverse primer  1        CGCAGATGCCATAGGAGACC  20
Template        5678709  ..........G.........  5678690
```

>CP075798.1 Pseudomonas aeruginosa strain PaLo323 chromosome, complete genome

```
product length = 269
Forward primer  1        GCAACGACACCAGCACACC  19
Template        5668381  ...................  5668399

Reverse primer  1        CGCAGATGCCATAGGAGACC  20
Template        5668649  ..........G.........  5668630
```

>CP075797.1 Pseudomonas aeruginosa strain PaLo326 chromosome, complete genome

```
product length = 269
Forward primer  1        GCAACGACACCAGCACACC  19
Template        5576047  ...................  5576065

Reverse primer  1        CGCAGATGCCATAGGAGACC  20
Template        5576315  ...................G  5576296
```

>CP075757.1 Pseudomonas aeruginosa strain PaLo552 chromosome, complete genome

```
product length = 269
Forward primer  1        GCAACGACACCAGCACACC  19
Template        5926139  ...................  5926157

Reverse primer  1        CGCAGATGCCATAGGAGACC  20
Template        5926407  ..........G.........  5926388
```

>CP110352.1 Pseudomonas aeruginosa strain PALA47 chromosome, complete genome

```
product length = 269
Forward primer  1        GCAACGACACCAGCACACC  19
Template        5778868  ...................  5778886

Reverse primer  1        CGCAGATGCCATAGGAGACC  20
Template        5779136  ..........G.........  5779117
```

>CP109932.1 Pseudomonas aeruginosa strain PALA43 chromosome, complete genome

```
product length = 269
Forward primer  1       GCAACGACACCAGCACACC  19
Template        292378  ...................  292396

Reverse primer  1       CGCAGATGCCATAGGAGACC  20
Template        292646  ..........G.........  292627
```

>CP110347.1 Pseudomonas aeruginosa strain PALA52 chromosome, complete genome

```
product length = 269
Forward primer  1        GCAACGACACCAGCACACC  19
Template        5450539  ...................  5450557

Reverse primer  1        CGCAGATGCCATAGGAGACC  20
Template        5450807  ...................G  5450788
```

>CP109833.1 Pseudomonas aeruginosa strain PALA23 chromosome, complete genome

```
product length = 269
Forward primer  1        GCAACGACACCAGCACACC  19
Template        5573559  ...................  5573577

Reverse primer  1        CGCAGATGCCATAGGAGACC  20
Template        5573827  ..........G.........  5573808
```

>CP106680.1 Pseudomonas aeruginosa strain PALA11 chromosome, complete genome

```
product length = 269
Forward primer  1        GCAACGACACCAGCACACC  19
Template        5535025  ...................  5535043

Reverse primer  1        CGCAGATGCCATAGGAGACC  20
Template        5535293  ..........G.........  5535274
```

>CP104868.1 Pseudomonas aeruginosa strain PALA7 chromosome, complete genome

```
product length = 269
Forward primer  1        GCAACGACACCAGCACACC  19
Template        5776773  ...................  5776791

Reverse primer  1        CGCAGATGCCATAGGAGACC  20
Template        5777041  ..........G.........  5777022
```

>CP097555.1 Pseudomonas aeruginosa strain B1.2 chromosome, complete genome

```
product length = 269
Forward primer  1        GCAACGACACCAGCACACC  19
Template        5550145  ...................  5550163

Reverse primer  1        CGCAGATGCCATAGGAGACC  20
Template        5550413  ..........G.........  5550394
```

>CP097556.1 Pseudomonas aeruginosa strain B2.1 chromosome, complete genome

```
product length = 269
Forward primer  1        GCAACGACACCAGCACACC  19
Template        5550132  ...................  5550150

Reverse primer  1        CGCAGATGCCATAGGAGACC  20
Template        5550400  ..........G.........  5550381
```

>CP097557.1 Pseudomonas aeruginosa strain C1.3 chromosome, complete genome

```
product length = 269
Forward primer  1        GCAACGACACCAGCACACC  19
Template        6252801  ...................  6252819

Reverse primer  1        CGCAGATGCCATAGGAGACC  20
Template        6253069  ..........G.........  6253050
```

>CP104170.1 Pseudomonas aeruginosa strain HW001G chromosome, complete genome

```
product length = 269
Forward primer  1        GCAACGACACCAGCACACC  19
Template        5102853  ...................  5102871

Reverse primer  1        CGCAGATGCCATAGGAGACC  20
Template        5103121  ..........G.........  5103102
```

>CP100759.1 Pseudomonas aeruginosa strain PA0009 chromosome

```
product length = 269
Forward primer  1        GCAACGACACCAGCACACC  19
Template        1609164  ...................  1609146

Reverse primer  1        CGCAGATGCCATAGGAGACC  20
Template        1608896  ...................G  1608915
```

>CP069177.1 Pseudomonas aeruginosa strain Z154 chromosome, complete genome

```
product length = 269
Forward primer  1        GCAACGACACCAGCACACC  19
Template        1300337  ...................  1300319

Reverse primer  1        CGCAGATGCCATAGGAGACC  20
Template        1300069  ..........G.........  1300088
```

>CP050147.1 Pseudomonas aeruginosa strain A5803 chromosome, complete genome

```
product length = 269
Forward primer  1        GCAACGACACCAGCACACC  19
Template        6017763  ...................  6017781

Reverse primer  1        CGCAGATGCCATAGGAGACC  20
Template        6018031  ..........G.........  6018012
```

>CP093013.1 Pseudomonas aeruginosa strain H19 chromosome

```
product length = 269
Forward primer  1        GCAACGACACCAGCACACC  19
Template        3693599  ...................  3693581

Reverse primer  1        CGCAGATGCCATAGGAGACC  20
Template        3693331  ..........G.........  3693350
```

>CP093022.1 Pseudomonas aeruginosa strain H08 chromosome, complete genome

```
product length = 269
Forward primer  1        GCAACGACACCAGCACACC  19
Template        6017996  ...................  6018014

Reverse primer  1        CGCAGATGCCATAGGAGACC  20
Template        6018264  ..........G.........  6018245
```

>CP093021.1 Pseudomonas aeruginosa strain H09 chromosome, complete genome

```
product length = 269
Forward primer  1        GCAACGACACCAGCACACC  19
Template        5605082  ...................  5605100

Reverse primer  1        CGCAGATGCCATAGGAGACC  20
Template        5605350  ...................G  5605331
```

>CP090348.1 Pseudomonas aeruginosa strain PA8329 chromosome, complete genome

```
product length = 269
Forward primer  1        GCAACGACACCAGCACACC  19
Template        5582697  ...................  5582715

Reverse primer  1        CGCAGATGCCATAGGAGACC  20
Template        5582965  ..........G.........  5582946
```

>CP077988.1 Pseudomonas aeruginosa strain ZPPH1 chromosome, complete genome

```
product length = 269
Forward primer  1        GCAACGACACCAGCACACC  19
Template        5763887  ...................  5763905

Reverse primer  1        CGCAGATGCCATAGGAGACC  20
Template        5764155  ..........G.........  5764136
```

>CP077985.1 Pseudomonas aeruginosa strain ZPPH2 chromosome, complete genome

```
product length = 269
Forward primer  1        GCAACGACACCAGCACACC  19
Template        5839914  ...................  5839932

Reverse primer  1        CGCAGATGCCATAGGAGACC  20
Template        5840182  ..........G.........  5840163
```

>CP077971.1 Pseudomonas aeruginosa strain ZPPH33 chromosome, complete genome

```
product length = 269
Forward primer  1        GCAACGACACCAGCACACC  19
Template        5695662  ...................  5695680

Reverse primer  1        CGCAGATGCCATAGGAGACC  20
Template        5695930  ..........G.........  5695911
```

>CP081287.1 Pseudomonas aeruginosa strain F092021 chromosome, complete genome

```
product length = 269
Forward primer  1        GCAACGACACCAGCACACC  19
Template        5852331  ...................  5852349

Reverse primer  1        CGCAGATGCCATAGGAGACC  20
Template        5852599  ..........G.........  5852580
```

>CP078564.1 Pseudomonas aeruginosa strain Colony464 chromosome

```
product length = 269
Forward primer  1        GCAACGACACCAGCACACC  19
Template        5671001  ...................  5670983

Reverse primer  1        CGCAGATGCCATAGGAGACC  20
Template        5670733  ..........G.........  5670752
```

>CP061376.1 Pseudomonas aeruginosa strain HS17-127 chromosome, complete genome

```
product length = 269
Forward primer  1        GCAACGACACCAGCACACC  19
Template        5588293  ...................  5588311

Reverse primer  1        CGCAGATGCCATAGGAGACC  20
Template        5588561  ..........G.........  5588542
```

>CP065865.1 Pseudomonas aeruginosa strain TJ2019-022 chromosome, complete genome

```
product length = 269
Forward primer  1        GCAACGACACCAGCACACC  19
Template        6300344  ...................  6300362

Reverse primer  1        CGCAGATGCCATAGGAGACC  20
Template        6300612  ..........G.........  6300593
```

>CP059852.1 Pseudomonas aeruginosa strain ZM03 chromosome, complete genome

```
product length = 269
Forward primer  1        GCAACGACACCAGCACACC  19
Template        5114401  ...................  5114383

Reverse primer  1        CGCAGATGCCATAGGAGACC  20
Template        5114133  ..........G.........  5114152
```

>CP063047.1 Pseudomonas aeruginosa strain KC-Tt-1 chromosome, complete genome

```
product length = 269
Forward primer  1        GCAACGACACCAGCACACC  19
Template        4700536  ...................  4700554

Reverse primer  1        CGCAGATGCCATAGGAGACC  20
Template        4700804  ..........G.........  4700785
```

>CP061034.1 Pseudomonas aeruginosa strain PA3 chromosome, complete genome

```
product length = 269
Forward primer  1        GCAACGACACCAGCACACC  19
Template        5716683  ...................  5716701

Reverse primer  1        CGCAGATGCCATAGGAGACC  20
Template        5716951  ..........G.........  5716932
```

>CP050323.1 Pseudomonas aeruginosa strain DVT429 chromosome, complete genome

```
product length = 269
Forward primer  1        GCAACGACACCAGCACACC  19
Template        1283811  ...................  1283793

Reverse primer  1        CGCAGATGCCATAGGAGACC  20
Template        1283543  ..........G.........  1283562
```

>CP053687.1 Pseudomonas aeruginosa strain K19PSE24 chromosome

```
product length = 269
Forward primer  1        GCAACGACACCAGCACACC  19
Template        5145741  ...................  5145759

Reverse primer  1        CGCAGATGCCATAGGAGACC  20
Template        5146009  ..........G.........  5145990
```

>CP046602.1 Pseudomonas aeruginosa strain CMC-115 chromosome, complete genome

```
product length = 269
Forward primer  1        GCAACGACACCAGCACACC  19
Template        5592644  ...................  5592662

Reverse primer  1        CGCAGATGCCATAGGAGACC  20
Template        5592912  ...................G  5592893
```

>CP045916.1 Pseudomonas aeruginosa strain CF39S chromosome, complete genome

```
product length = 269
Forward primer  1        GCAACGACACCAGCACACC  19
Template        5967484  ...................  5967502

Reverse primer  1        CGCAGATGCCATAGGAGACC  20
Template        5967752  ..........G.........  5967733
```

>LR739068.1 Pseudomonas aeruginosa strain PcyII-29 genome assembly, chromosome: PcyII-29

```
product length = 269
Forward primer  1        GCAACGACACCAGCACACC  19
Template        5825042  ...................  5825060

Reverse primer  1        CGCAGATGCCATAGGAGACC  20
Template        5825310  ..........G.........  5825291
```

>LR700248.1 Pseudomonas aeruginosa isolate ID40 genome assembly, chromosome: ID40_omosome

```
product length = 269
Forward primer  1        GCAACGACACCAGCACACC  19
Template        3071312  ...................  3071330

Reverse primer  1        CGCAGATGCCATAGGAGACC  20
```

```
Template        3071580  .........G.........  3071561
```

>CP041013.1 Pseudomonas aeruginosa strain FDAARGOS_610 chromosome, complete genome

```
product length = 269
Forward primer  1       GCAACGACACCAGCACACC  19
Template        847207  ...................  847189

Reverse primer  1       CGCAGATGCCATAGGAGACC  20
Template        846939  .........G.........  846958
```

>LR590473.1 Pseudomonas aeruginosa strain NCTC13359 genome assembly, chromosome: 1

```
product length = 269
Forward primer  1        GCAACGACACCAGCACACC  19
Template        6381512  ...................  6381530

Reverse primer  1        CGCAGATGCCATAGGAGACC  20
Template        6381780  .........G.........  6381761
```

>CP039749.1 Pseudomonas aeruginosa strain PRD-10 chromosome

```
product length = 269
Forward primer  1        GCAACGACACCAGCACACC  19
Template        5270507  ...................  5270525

Reverse primer  1        CGCAGATGCCATAGGAGACC  20
Template        5270775  .........G.........  5270756
```

>CP031660.1 Pseudomonas aeruginosa strain PABL017 chromosome, complete genome

```
product length = 269
Forward primer  1        GCAACGACACCAGCACACC  19
Template        5712654  ...................  5712672

Reverse primer  1        CGCAGATGCCATAGGAGACC  20
Template        5712922  .........G.........  5712903
```

>CP008860.2 Pseudomonas aeruginosa strain H27930 chromosome, complete genome

```
product length = 269
Forward primer  1        GCAACGACACCAGCACACC  19
Template        5772054  ...................  5772072

Reverse primer  1        CGCAGATGCCATAGGAGACC  20
Template        5772322  .........G.........  5772303
```

>CP012582.1 Pseudomonas aeruginosa strain PA_D21, complete genome

```
product length = 269
Forward primer  1        GCAACGACACCAGCACACC  19
Template        5771114  ...................  5771132
```

```
Reverse primer  1        CGCAGATGCCATAGGAGACC  20
Template        5771382  ..........G.........  5771363
```

>CP012579.1 Pseudomonas aeruginosa strain PA_D5, complete genome

```
product length = 269
Forward primer  1        GCAACGACACCAGCACACC  19
Template        5813997  ...................  5814015

Reverse primer  1        CGCAGATGCCATAGGAGACC  20
Template        5814265  ..........G.........  5814246
```

>CP012584.1 Pseudomonas aeruginosa strain PA_D25, complete genome

```
product length = 269
Forward primer  1        GCAACGACACCAGCACACC  19
Template        5815210  ...................  5815228

Reverse primer  1        CGCAGATGCCATAGGAGACC  20
Template        5815478  ..........G.........  5815459
```

>CP012583.1 Pseudomonas aeruginosa strain PA_D22, complete genome

```
product length = 269
Forward primer  1        GCAACGACACCAGCACACC  19
Template        5813986  ...................  5814004

Reverse primer  1        CGCAGATGCCATAGGAGACC  20
Template        5814254  ..........G.........  5814235
```

>CP012581.1 Pseudomonas aeruginosa strain PA_D16, complete genome

```
product length = 269
Forward primer  1        GCAACGACACCAGCACACC  19
Template        5813980  ...................  5813998

Reverse primer  1        CGCAGATGCCATAGGAGACC  20
Template        5814248  ..........G.........  5814229
```

>CP012580.1 Pseudomonas aeruginosa strain PA_D9, complete genome

```
product length = 269
Forward primer  1        GCAACGACACCAGCACACC  19
Template        5777482  ...................  5777500

Reverse primer  1        CGCAGATGCCATAGGAGACC  20
Template        5777750  ..........G.........  5777731
```

>CP012578.1 Pseudomonas aeruginosa strain PA_D2, complete genome

```
product length = 269
Forward primer  1        GCAACGACACCAGCACACC  19
Template        5775001  ...................  5775019
```

```
Reverse primer  1        CGCAGATGCCATAGGAGACC  20
Template        5775269  ..........G.........  5775250
```

>CP012585.1 Pseudomonas aeruginosa strain PA_D1, complete genome

```
product length = 269
Forward primer  1        GCAACGACACCAGCACACC  19
Template        5775828  ...................  5775846

Reverse primer  1        CGCAGATGCCATAGGAGACC  20
Template        5776096  ..........G.........  5776077
```

>CP016214.1 Pseudomonas aeruginosa strain PA121617, complete genome

```
product length = 269
Forward primer  1        GCAACGACACCAGCACACC  19
Template        2417007  ...................  2417025

Reverse primer  1        CGCAGATGCCATAGGAGACC  20
Template        2417275  ..........G.........  2417256
```

>CP015377.1 Pseudomonas aeruginosa strain BAMCPA07-48 chromosome, complete genome

```
product length = 269
Forward primer  1        GCAACGACACCAGCACACC  19
Template        1249814  ...................  1249832

Reverse primer  1        CGCAGATGCCATAGGAGACC  20
Template        1250082  ..........G.........  1250063
```

>CP008867.1 Pseudomonas aeruginosa strain T52373 chromosome, complete genome

```
product length = 269
Forward primer  1        GCAACGACACCAGCACACC  19
Template        1693406  ...................  1693424

Reverse primer  1        CGCAGATGCCATAGGAGACC  20
Template        1693674  ..........G.........  1693655
```

>CP007224.1 Pseudomonas aeruginosa PA96 genome

```
product length = 269
Forward primer  1        GCAACGACACCAGCACACC  19
Template        5640523  ...................  5640541

Reverse primer  1        CGCAGATGCCATAGGAGACC  20
Template        5640791  ..........G.........  5640772
```

If you want to allow any of the unintended targets, check the box(es) next to the ones you accept and try again to re-search for specific primers Submit

Help

FOLLOW NCBI

Connect with NLM

National Library of Medicine
8600 Rockville Pike
Bethesda, MD 20894

Web Policies
FOIA
HHS Vulnerability Disclosure

Help
Accessibility
Careers

NLM NIH HHS USA.gov
