# Supplementary material for: Phylogenetic Aspects of Antibiotic Resistance and Biofilm Formation of P. aeruginosa Isolated from Clinical Samples
Source: Can J Infect Dis Med Microbiol. 2024 Jan 13;2024:6213873. doi: 10.1155/2024/6213873 (PMC10799695; doi:10.1155/2024/6213873)
Supplement: Supplementary Materials — Original pictures and primer-blast results. [file 6213873.f1.zip › Mex-A Primer-Blast results.pdf]

An official website of the United States government

Here's how you know

The .gov means it’s official.
Federal government websites often end in .gov or .mil. Before sharing sensitive information, make sure you’re on a federal government site.

The site is secure.
The https:// ensures that you are connecting to the official website and that any information you provide is encrypted and transmitted securely.

Skip to main page content

Access keys NCBI Homepage MyNCBI Homepage Main Content Main Navigation

Log in

Primer-BLAST

» JOB ID:rKZypwRvCccu-Qz8AZwoznuHOfxWlCLhVw

PrimerBLAST users!
We want to hear from you about how PrimerBLAST can be improved.
Contact us

Primer-BLAST Results
Help

•

Input PCR template
none

Specificity of primers
Target templates were found in selected database: Nucleotide collection (nt)

Other reports
Search Summary

Detailed primer reports
You can re-search for specific primers by accepting some of the unintended targets, check the box(es) next to the ones you accept and try again to re-search for specific primers Submit
Help

Primer pair 1

	Sequence (5'->3')	Length	Tm	GC%	Self complementarity	Self 3' complementarity
Forward primer	CTGAAGCTGGAGGACGGTAG	20	59.54	60.00	4.00	0.00
Reverse primer	AGGCCTTCGGTAATGATCTTGT	22	59.49	45.45	6.00	2.00

Products on intended targets

Products on allowed targets

Products on allowed transcript variants

Products on potentially unintended templates

Products on target templates

>CP127126.1 Pseudomonas aeruginosa PA14 strain MA3 isolate DTU_MIE chromosome, complete genome

```
product length = 356
Forward primer  1       CTGAAGCTGGAGGACGGTAG  20
Template        486983  ....................  487002

Reverse primer  1       AGGCCTTCGGTAATGATCTTGT  22
Template        487338  ......................  487317
```

>CP121766.1 Pseudomonas aeruginosa strain 22112 chromosome, complete genome

```
product length = 356
Forward primer  1        CTGAAGCTGGAGGACGGTAG  20
Template        6831492  ....................  6831511

Reverse primer  1        AGGCCTTCGGTAATGATCTTGT  22
Template        6831847  ......................  6831826
```

>CP127016.1 Pseudomonas aeruginosa strain TBCF10839 chromosome

```
product length = 356
Forward primer  1        CTGAAGCTGGAGGACGGTAG  20
Template        3853779  ....................  3853760

Reverse primer  1        AGGCCTTCGGTAATGATCTTGT  22
Template        3853424  ......................  3853445
```

>CP123792.1 Pseudomonas aeruginosa strain 2021CK-01658 chromosome, complete genome

```
product length = 356
Forward primer  1        CTGAAGCTGGAGGACGGTAG  20
Template        6600550  ....................  6600531

Reverse primer  1        AGGCCTTCGGTAATGATCTTGT  22
Template        6600195  ......................  6600216
```

>CP109757.1 Pseudomonas aeruginosa strain 2017-45-85 chromosome, complete genome

```
product length = 356
Forward primer  1       CTGAAGCTGGAGGACGGTAG  20
Template        267918  ....................  267937

Reverse primer  1       AGGCCTTCGGTAATGATCTTGT  22
Template        268273  ......................  268252
```

>CP109683.1 Pseudomonas aeruginosa strain 2017-45-169 chromosome, complete genome

```
product length = 356
Forward primer  1        CTGAAGCTGGAGGACGGTAG  20
Template        2210162  ....................  2210143

Reverse primer  1        AGGCCTTCGGTAATGATCTTGT  22
Template        2209807  ......................  2209828
```

>CP109685.1 Pseudomonas aeruginosa strain 2017-45-137A chromosome, complete genome

```
product length = 356
Forward primer  1        CTGAAGCTGGAGGACGGTAG  20
Template        5509920  ....................  5509939

Reverse primer  1        AGGCCTTCGGTAATGATCTTGT  22
Template        5510275  ......................  5510254
```

>CP061073.2 Pseudomonas aeruginosa strain PAD8 chromosome, complete genome

```
product length = 356
Forward primer  1        CTGAAGCTGGAGGACGGTAG  20
Template        3715148  ....................  3715167

Reverse primer  1        AGGCCTTCGGTAATGATCTTGT  22
Template        3715503  ......................  3715482
```

>CP123786.1 Pseudomonas aeruginosa strain 2021CK-01424 chromosome, complete genome

```
product length = 356
Forward primer  1       CTGAAGCTGGAGGACGGTAG  20
Template        646098  ....................  646117

Reverse primer  1       AGGCCTTCGGTAATGATCTTGT  22
Template        646453  ......................  646432
```

>CP123785.1 Pseudomonas aeruginosa strain 2021CK-01267 chromosome, complete genome

```
product length = 356
Forward primer  1       CTGAAGCTGGAGGACGGTAG  20
Template        646098  ....................  646117

Reverse primer  1       AGGCCTTCGGTAATGATCTTGT  22
Template        646453  ......................  646432
```

>CP123787.1 Pseudomonas aeruginosa strain 2020CK-00194 chromosome, complete genome

```
product length = 356
Forward primer  1       CTGAAGCTGGAGGACGGTAG  20
Template        606461  ....................  606480

Reverse primer  1       AGGCCTTCGGTAATGATCTTGT  22
Template        606816  ......................  606795
```

>CP123789.1 Pseudomonas aeruginosa strain 2021CK-01381 chromosome, complete genome

```
product length = 356
Forward primer  1        CTGAAGCTGGAGGACGGTAG  20
Template        6502937  ....................  6502918

Reverse primer  1        AGGCCTTCGGTAATGATCTTGT  22
Template        6502582  ......................  6502603
```

>CP123791.1 Pseudomonas aeruginosa strain 2021CK-01305 chromosome, complete genome

```
product length = 356
Forward primer  1       CTGAAGCTGGAGGACGGTAG  20
Template        606557  ....................  606576

Reverse primer  1       AGGCCTTCGGTAATGATCTTGT  22
Template        606912  ......................  606891
```

>CP123793.1 Pseudomonas aeruginosa strain 2021CK-01107 chromosome, complete genome

```
product length = 356
Forward primer  1       CTGAAGCTGGAGGACGGTAG  20
Template        606086  ....................  606105

Reverse primer  1       AGGCCTTCGGTAATGATCTTGT  22
Template        606441  ......................  606420
```

>CP096964.1 Pseudomonas aeruginosa strain NY13936 chromosome, complete genome

```
product length = 356
Forward primer  1       CTGAAGCTGGAGGACGGTAG  20
Template        473998  ....................  474017

Reverse primer  1       AGGCCTTCGGTAATGATCTTGT  22
Template        474353  ......................  474332
```

>CP096961.1 Pseudomonas aeruginosa strain NY13932 chromosome, complete genome

```
product length = 356
Forward primer  1       CTGAAGCTGGAGGACGGTAG  20
Template        467194  ....................  467213

Reverse primer  1       AGGCCTTCGGTAATGATCTTGT  22
Template        467549  ......................  467528
```

>CP096960.1 Pseudomonas aeruginosa strain NY11254 chromosome, complete genome

```
product length = 356
Forward primer  1       CTGAAGCTGGAGGACGGTAG  20
Template        509732  ....................  509751

Reverse primer  1       AGGCCTTCGGTAATGATCTTGT  22
Template        510087  ......................  510066
```

>CP096958.1 Pseudomonas aeruginosa strain NY11210 chromosome, complete genome

```
product length = 356
Forward primer  1       CTGAAGCTGGAGGACGGTAG  20
Template        513171  ....................  513190

Reverse primer  1       AGGCCTTCGGTAATGATCTTGT  22
Template        513526  ......................  513505
```

>CP096953.1 Pseudomonas aeruginosa strain NY5535 chromosome, complete genome

```
product length = 356
Forward primer  1       CTGAAGCTGGAGGACGGTAG  20
Template        513197  ....................  513216

Reverse primer  1       AGGCCTTCGGTAATGATCTTGT  22
Template        513552  ......................  513531
```

>CP096950.1 Pseudomonas aeruginosa strain NY5532 chromosome, complete genome

```
product length = 356
Forward primer  1       CTGAAGCTGGAGGACGGTAG  20
Template        484167  ....................  484186

Reverse primer  1       AGGCCTTCGGTAATGATCTTGT  22
Template        484522  ......................  484501
```

>CP096946.1 Pseudomonas aeruginosa strain NY5530 chromosome, complete genome

```
product length = 356
Forward primer  1       CTGAAGCTGGAGGACGGTAG  20
Template        512915  ....................  512934

Reverse primer  1       AGGCCTTCGGTAATGATCTTGT  22
Template        513270  ......................  513249
```

>CP096945.1 Pseudomonas aeruginosa strain NY5525 chromosome, complete genome

```
product length = 356
Forward primer  1       CTGAAGCTGGAGGACGGTAG  20
Template        523008  ....................  523027

Reverse primer  1       AGGCCTTCGGTAATGATCTTGT  22
Template        523363  ......................  523342
```

>CP096942.1 Pseudomonas aeruginosa strain NY5524 chromosome, complete genome

```
product length = 356
Forward primer  1       CTGAAGCTGGAGGACGGTAG  20
Template        521147  ....................  521166

Reverse primer  1       AGGCCTTCGGTAATGATCTTGT  22
Template        521502  ......................  521481
```

>CP096941.1 Pseudomonas aeruginosa strain NY5523 chromosome, complete genome

```
product length = 356
Forward primer  1       CTGAAGCTGGAGGACGGTAG  20
Template        509734  ....................  509753

Reverse primer  1       AGGCCTTCGGTAATGATCTTGT  22
Template        510089  ......................  510068
```

>CP096937.1 Pseudomonas aeruginosa strain NY5520 chromosome, complete genome

```
product length = 356
Forward primer  1       CTGAAGCTGGAGGACGGTAG  20
Template        512897  ....................  512916

Reverse primer  1       AGGCCTTCGGTAATGATCTTGT  22
Template        513252  ......................  513231
```

>CP096934.1 Pseudomonas aeruginosa strain NY5511 chromosome, complete genome

```
product length = 356
Forward primer  1       CTGAAGCTGGAGGACGGTAG  20
Template        513196  ....................  513215

Reverse primer  1       AGGCCTTCGGTAATGATCTTGT  22
Template        513551  ......................  513530
```

>CP096932.1 Pseudomonas aeruginosa strain NY5510 chromosome, complete genome

```
product length = 356
Forward primer  1       CTGAAGCTGGAGGACGGTAG  20
Template        513196  ....................  513215

Reverse primer  1       AGGCCTTCGGTAATGATCTTGT  22
Template        513551  ......................  513530
```

>CP096929.1 Pseudomonas aeruginosa strain NY5507 chromosome, complete genome

```
product length = 356
Forward primer  1       CTGAAGCTGGAGGACGGTAG  20
Template        513178  ....................  513197

Reverse primer  1       AGGCCTTCGGTAATGATCTTGT  22
Template        513533  ......................  513512
```

>CP096927.1 Pseudomonas aeruginosa strain NY5506 chromosome, complete genome

```
product length = 356
Forward primer  1       CTGAAGCTGGAGGACGGTAG  20
Template        469204  ....................  469223

Reverse primer  1       AGGCCTTCGGTAATGATCTTGT  22
Template        469559  ......................  469538
```

>CP124673.1 Pseudomonas aeruginosa strain 2022CK-00491 chromosome, complete genome

```
product length = 356
Forward primer  1       CTGAAGCTGGAGGACGGTAG  20
Template        607821  ....................  607840

Reverse primer  1       AGGCCTTCGGTAATGATCTTGT  22
Template        608176  ......................  608155
```

>CP124674.1 Pseudomonas aeruginosa strain 2022CK-00339 chromosome, complete genome

```
product length = 356
Forward primer  1       CTGAAGCTGGAGGACGGTAG  20
Template        468858  ....................  468877

Reverse primer  1       AGGCCTTCGGTAATGATCTTGT  22
Template        469213  ......................  469192
```

>CP084890.1 Pseudomonas aeruginosa strain CH1 chromosome

```
product length = 356
Forward primer  1        CTGAAGCTGGAGGACGGTAG  20
Template        1859144  ....................  1859125

Reverse primer  1        AGGCCTTCGGTAATGATCTTGT  22
Template        1858789  ......................  1858810
```

>CP124662.1 Pseudomonas aeruginosa strain 2021CK-01633 chromosome, complete genome

```
product length = 356
Forward primer  1       CTGAAGCTGGAGGACGGTAG  20
Template        606461  ....................  606480

Reverse primer  1       AGGCCTTCGGTAATGATCTTGT  22
Template        606816  ......................  606795
```

>CP124660.1 Pseudomonas aeruginosa strain 2022CK-00160 chromosome, complete genome

```
product length = 356
Forward primer  1       CTGAAGCTGGAGGACGGTAG  20
Template        524554  ....................  524573

Reverse primer  1       AGGCCTTCGGTAATGATCTTGT  22
Template        524909  ......................  524888
```

>CP124652.1 Pseudomonas aeruginosa strain 2020CK-00443 chromosome, complete genome

```
product length = 356
Forward primer  1        CTGAAGCTGGAGGACGGTAG  20
Template        6308088  ....................  6308069

Reverse primer  1        AGGCCTTCGGTAATGATCTTGT  22
Template        6307733  ......................  6307754
```

>CP124649.1 Pseudomonas aeruginosa strain 2020CK-00218 chromosome, complete genome

```
product length = 356
Forward primer  1       CTGAAGCTGGAGGACGGTAG  20
Template        548695  ....................  548714

Reverse primer  1       AGGCCTTCGGTAATGATCTTGT  22
Template        549050  ......................  549029
```

>CP124664.1 Pseudomonas aeruginosa strain 2021CK-01256 chromosome, complete genome

```
product length = 356
Forward primer  1       CTGAAGCTGGAGGACGGTAG  20
Template        457511  ....................  457530

Reverse primer  1       AGGCCTTCGGTAATGATCTTGT  22
Template        457866  ......................  457845
```

>CP124655.1 Pseudomonas aeruginosa strain 2022CK-00096 chromosome, complete genome

```
product length = 356
Forward primer  1        CTGAAGCTGGAGGACGGTAG  20
Template        6388149  ....................  6388130

Reverse primer  1        AGGCCTTCGGTAATGATCTTGT  22
Template        6387794  ......................  6387815
```

>CP124654.1 Pseudomonas aeruginosa strain 2021CK-01851 chromosome, complete genome

```
product length = 356
Forward primer  1       CTGAAGCTGGAGGACGGTAG  20
Template        455597  ....................  455616

Reverse primer  1       AGGCCTTCGGTAATGATCTTGT  22
Template        455952  ......................  455931
```

>CP124657.1 Pseudomonas aeruginosa strain 2022CK-00069 chromosome, complete genome

```
product length = 356
Forward primer  1       CTGAAGCTGGAGGACGGTAG  20
Template        548365  ....................  548384

Reverse primer  1       AGGCCTTCGGTAATGATCTTGT  22
Template        548720  ......................  548699
```

>CP124651.1 Pseudomonas aeruginosa strain 2020CK-00217 chromosome, complete genome

```
product length = 356
Forward primer  1       CTGAAGCTGGAGGACGGTAG  20
Template        606461  ....................  606480

Reverse primer  1       AGGCCTTCGGTAATGATCTTGT  22
Template        606816  ......................  606795
```

>CP124669.1 Pseudomonas aeruginosa strain 2021CK-01494 chromosome, complete genome

```
product length = 356
Forward primer  1       CTGAAGCTGGAGGACGGTAG  20
Template        463705  ....................  463724

Reverse primer  1       AGGCCTTCGGTAATGATCTTGT  22
Template        464060  ......................  464039
```

>CP124638.1 Pseudomonas aeruginosa strain 2021CK-01158 chromosome, complete genome

```
product length = 356
Forward primer  1       CTGAAGCTGGAGGACGGTAG  20
Template        472280  ....................  472299

Reverse primer  1       AGGCCTTCGGTAATGATCTTGT  22
Template        472635  ......................  472614
```

>CP124668.1 Pseudomonas aeruginosa strain 2021CK-01445 chromosome, complete genome

```
product length = 356
Forward primer  1       CTGAAGCTGGAGGACGGTAG  20
Template        463701  ....................  463720

Reverse primer  1       AGGCCTTCGGTAATGATCTTGT  22
Template        464056  ......................  464035
```

>CP124666.1 Pseudomonas aeruginosa strain 2021CK-01283 chromosome, complete genome

```
product length = 356
Forward primer  1       CTGAAGCTGGAGGACGGTAG  20
Template        486144  ....................  486163

Reverse primer  1       AGGCCTTCGGTAATGATCTTGT  22
Template        486499  ......................  486478
```

>CP124624.1 Pseudomonas aeruginosa strain 2021CK-01157 chromosome, complete genome

```
product length = 356
Forward primer  1       CTGAAGCTGGAGGACGGTAG  20
Template        472282  ....................  472301

Reverse primer  1       AGGCCTTCGGTAATGATCTTGT  22
Template        472637  ......................  472616
```

>CP124665.1 Pseudomonas aeruginosa strain 2021CK-01229 chromosome, complete genome

```
product length = 356
Forward primer  1       CTGAAGCTGGAGGACGGTAG  20
Template        486144  ....................  486163

Reverse primer  1       AGGCCTTCGGTAATGATCTTGT  22
Template        486499  ......................  486478
```

>CP124667.1 Pseudomonas aeruginosa strain 2021CK-01315 chromosome, complete genome

```
product length = 356
Forward primer  1       CTGAAGCTGGAGGACGGTAG  20
Template        480531  ....................  480550

Reverse primer  1       AGGCCTTCGGTAATGATCTTGT  22
Template        480886  ......................  480865
```

>CP124641.1 Pseudomonas aeruginosa strain 2021CK-01198 chromosome, complete genome

```
product length = 356
Forward primer  1       CTGAAGCTGGAGGACGGTAG  20
Template        472285  ....................  472304

Reverse primer  1       AGGCCTTCGGTAATGATCTTGT  22
Template        472640  ......................  472619
```

>CP124670.1 Pseudomonas aeruginosa strain 2021CK-01536 chromosome, complete genome

```
product length = 356
Forward primer  1       CTGAAGCTGGAGGACGGTAG  20
Template        475978  ....................  475997

Reverse primer  1       AGGCCTTCGGTAATGATCTTGT  22
Template        476333  ......................  476312
```

>CP124646.1 Pseudomonas aeruginosa strain 2020CK-00185 chromosome, complete genome

```
product length = 356
Forward primer  1       CTGAAGCTGGAGGACGGTAG  20
Template        489209  ....................  489228

Reverse primer  1       AGGCCTTCGGTAATGATCTTGT  22
Template        489564  ......................  489543
```

>CP124648.1 Pseudomonas aeruginosa strain 2020CK-00220 chromosome, complete genome

```
product length = 356
Forward primer  1        CTGAAGCTGGAGGACGGTAG  20
Template        2980742  ....................  2980761

Reverse primer  1        AGGCCTTCGGTAATGATCTTGT  22
Template        2981097  ......................  2981076
```

>CP124643.1 Pseudomonas aeruginosa strain 2021CK-01197 chromosome, complete genome

```
product length = 356
Forward primer  1        CTGAAGCTGGAGGACGGTAG  20
Template        4268026  ....................  4268007

Reverse primer  1        AGGCCTTCGGTAATGATCTTGT  22
Template        4267671  ......................  4267692
```

>CP124626.1 Pseudomonas aeruginosa strain 2021CK-01161 chromosome, complete genome

```
product length = 356
Forward primer  1       CTGAAGCTGGAGGACGGTAG  20
Template        386749  ....................  386730

Reverse primer  1       AGGCCTTCGGTAATGATCTTGT  22
Template        386394  ......................  386415
```

>CP124632.1 Pseudomonas aeruginosa strain 2021CK-01162 chromosome, complete genome

```
product length = 356
Forward primer  1       CTGAAGCTGGAGGACGGTAG  20
Template        891196  ....................  891215

Reverse primer  1       AGGCCTTCGGTAATGATCTTGT  22
Template        891551  ......................  891530
```

>CP124663.1 Pseudomonas aeruginosa strain 2021CK-01227 chromosome, complete genome

```
product length = 356
Forward primer  1       CTGAAGCTGGAGGACGGTAG  20
Template        486144  ....................  486163

Reverse primer  1       AGGCCTTCGGTAATGATCTTGT  22
Template        486499  ......................  486478
```

>CP124622.1 Pseudomonas aeruginosa strain 2021CK-01159 chromosome, complete genome

```
product length = 356
Forward primer  1       CTGAAGCTGGAGGACGGTAG  20
Template        480531  ....................  480550

Reverse primer  1       AGGCCTTCGGTAATGATCTTGT  22
Template        480886  ......................  480865
```

>CP124600.1 Pseudomonas aeruginosa strain Li010 chromosome, complete genome

```
product length = 356
Forward primer  1       CTGAAGCTGGAGGACGGTAG  20
Template        477520  ....................  477539

Reverse primer  1       AGGCCTTCGGTAATGATCTTGT  22
Template        477875  ......................  477854
```

>CP123953.1 Pseudomonas aeruginosa strain 59 chromosome, complete genome

```
product length = 356
Forward primer  1       CTGAAGCTGGAGGACGGTAG  20
Template        468144  ....................  468163

Reverse primer  1       AGGCCTTCGGTAATGATCTTGT  22
Template        468499  ......................  468478
```

>CP116682.1 Pseudomonas aeruginosa strain HS337 chromosome, complete genome

```
product length = 356
Forward primer  1       CTGAAGCTGGAGGACGGTAG  20
Template        484095  ....................  484114

Reverse primer  1       AGGCCTTCGGTAATGATCTTGT  22
Template        484450  ......................  484429
```

>CP110190.1 Pseudomonas aeruginosa strain HS204 chromosome, complete genome

```
product length = 356
Forward primer  1       CTGAAGCTGGAGGACGGTAG  20
Template        480615  ....................  480634

Reverse primer  1       AGGCCTTCGGTAATGATCTTGT  22
Template        480970  ......................  480949
```

>CP118638.1 Pseudomonas aeruginosa strain P9 chromosome, complete genome

```
product length = 356
Forward primer  1       CTGAAGCTGGAGGACGGTAG  20
Template        466405  ....................  466424

Reverse primer  1       AGGCCTTCGGTAATGATCTTGT  22
Template        466760  ......................  466739
```

>CP118641.1 Pseudomonas aeruginosa strain P23 chromosome, complete genome

```
product length = 356
Forward primer  1       CTGAAGCTGGAGGACGGTAG  20
Template        481064  ....................  481083

Reverse primer  1       AGGCCTTCGGTAATGATCTTGT  22
Template        481419  ......................  481398
```

>CP119298.1 Pseudomonas aeruginosa strain SNDPR-01 chromosome, complete genome

```
product length = 356
Forward primer  1       CTGAAGCTGGAGGACGGTAG  20
Template        498429  ....................  498448

Reverse primer  1       AGGCCTTCGGTAATGATCTTGT  22
Template        498784  ......................  498763
```

>CP117300.1 Pseudomonas aeruginosa strain 0201761-1 chromosome, complete genome

```
product length = 356
Forward primer  1       CTGAAGCTGGAGGACGGTAG  20
Template        474015  ....................  474034

Reverse primer  1       AGGCCTTCGGTAATGATCTTGT  22
Template        474370  ......................  474349
```

>CP084321.1 Pseudomonas aeruginosa strain HS18-89 chromosome, complete genome

```
product length = 356
Forward primer  1       CTGAAGCTGGAGGACGGTAG  20
Template        505631  ....................  505650

Reverse primer  1       AGGCCTTCGGTAATGATCTTGT  22
Template        505986  ......................  505965
```

>CP117974.1 Pseudomonas aeruginosa strain B-3509 chromosome, complete genome

```
product length = 356
Forward primer  1        CTGAAGCTGGAGGACGGTAG  20
Template        5070540  ....................  5070521

Reverse primer  1        AGGCCTTCGGTAATGATCTTGT  22
Template        5070185  ......................  5070206
```

>CP117749.1 Pseudomonas aeruginosa strain 2022CK-00828 chromosome, complete genome

```
product length = 356
Forward primer  1       CTGAAGCTGGAGGACGGTAG  20
Template        463439  ....................  463458

Reverse primer  1       AGGCCTTCGGTAATGATCTTGT  22
Template        463794  ......................  463773
```

>CP117527.1 Pseudomonas aeruginosa strain MF1 chromosome, complete genome

```
product length = 356
Forward primer  1       CTGAAGCTGGAGGACGGTAG  20
Template        551250  ....................  551269

Reverse primer  1       AGGCCTTCGGTAATGATCTTGT  22
Template        551605  ......................  551584
```

>CP075851.1 Pseudomonas aeruginosa strain PaLo33 chromosome, complete genome

```
product length = 356
Forward primer  1       CTGAAGCTGGAGGACGGTAG  20
Template        517990  ....................  518009

Reverse primer  1       AGGCCTTCGGTAATGATCTTGT  22
Template        518345  ......................  518324
```

>CP075849.1 Pseudomonas aeruginosa strain PaLo1 chromosome, complete genome

```
product length = 356
Forward primer  1       CTGAAGCTGGAGGACGGTAG  20
Template        487803  ....................  487822

Reverse primer  1       AGGCCTTCGGTAATGATCTTGT  22
Template        488158  ......................  488137
```

>CP075848.1 Pseudomonas aeruginosa strain PaLo2 chromosome, complete genome

```
product length = 356
Forward primer  1       CTGAAGCTGGAGGACGGTAG  20
Template        468921  ....................  468940

Reverse primer  1       AGGCCTTCGGTAATGATCTTGT  22
Template        469276  ......................  469255
```

>CP075847.1 Pseudomonas aeruginosa strain PaLo3 chromosome, complete genome

```
product length = 356
Forward primer  1       CTGAAGCTGGAGGACGGTAG  20
Template        465896  ....................  465915

Reverse primer  1       AGGCCTTCGGTAATGATCTTGT  22
Template        466251  ......................  466230
```

>CP075846.1 Pseudomonas aeruginosa strain PaLo4 chromosome

```
product length = 356
Forward primer  1       CTGAAGCTGGAGGACGGTAG  20
Template        367215  ....................  367234

Reverse primer  1       AGGCCTTCGGTAATGATCTTGT  22
Template        367570  ......................  367549
```

>CP075844.1 Pseudomonas aeruginosa strain PaLo6 chromosome, complete genome

```
product length = 356
Forward primer  1       CTGAAGCTGGAGGACGGTAG  20
Template        473207  ....................  473226

Reverse primer  1       AGGCCTTCGGTAATGATCTTGT  22
Template        473562  ......................  473541
```

>CP075843.1 Pseudomonas aeruginosa strain PaLo7 chromosome

```
product length = 356
Forward primer  1       CTGAAGCTGGAGGACGGTAG  20
Template        506654  ....................  506673

Reverse primer  1       AGGCCTTCGGTAATGATCTTGT  22
Template        507009  ......................  506988
```

>CP075840.1 Pseudomonas aeruginosa strain PaLo10 chromosome, complete genome

```
product length = 356
Forward primer  1       CTGAAGCTGGAGGACGGTAG  20
Template        466050  ....................  466069

Reverse primer  1       AGGCCTTCGGTAATGATCTTGT  22
Template        466405  ......................  466384
```

>CP075838.1 Pseudomonas aeruginosa strain PaLo11 chromosome, complete genome

```
product length = 356
Forward primer  1       CTGAAGCTGGAGGACGGTAG  20
Template        467253  ....................  467272

Reverse primer  1       AGGCCTTCGGTAATGATCTTGT  22
Template        467608  ......................  467587
```

>CP075836.1 Pseudomonas aeruginosa strain PaLo12 chromosome, complete genome

```
product length = 356
Forward primer  1       CTGAAGCTGGAGGACGGTAG  20
Template        469362  ....................  469381

Reverse primer  1       AGGCCTTCGGTAATGATCTTGT  22
Template        469717  ......................  469696
```

>CP075835.1 Pseudomonas aeruginosa strain PaLo14 chromosome, complete genome

```
product length = 356
Forward primer  1       CTGAAGCTGGAGGACGGTAG  20
Template        517880  ....................  517899

Reverse primer  1       AGGCCTTCGGTAATGATCTTGT  22
Template        518235  ......................  518214
```

>CP075834.1 Pseudomonas aeruginosa strain PaLo15 chromosome, complete genome

```
product length = 356
Forward primer  1       CTGAAGCTGGAGGACGGTAG  20
Template        514541  ....................  514560

Reverse primer  1       AGGCCTTCGGTAATGATCTTGT  22
Template        514896  ......................  514875
```

>CP075833.1 Pseudomonas aeruginosa strain PaLo17 chromosome, complete genome

```
product length = 356
Forward primer  1       CTGAAGCTGGAGGACGGTAG  20
Template        468879  ....................  468898

Reverse primer  1       AGGCCTTCGGTAATGATCTTGT  22
Template        469234  ......................  469213
```

>CP075832.1 Pseudomonas aeruginosa strain PaLo20 chromosome, complete genome

```
product length = 356
Forward primer  1       CTGAAGCTGGAGGACGGTAG  20
Template        470644  ....................  470663

Reverse primer  1       AGGCCTTCGGTAATGATCTTGT  22
Template        470999  ......................  470978
```

>CP075831.1 Pseudomonas aeruginosa strain PaLo21 chromosome, complete genome

```
product length = 356
Forward primer  1       CTGAAGCTGGAGGACGGTAG  20
Template        467059  ....................  467078

Reverse primer  1       AGGCCTTCGGTAATGATCTTGT  22
Template        467414  ......................  467393
```

>CP075830.1 Pseudomonas aeruginosa strain PaLo22 chromosome, complete genome

```
product length = 356
Forward primer  1       CTGAAGCTGGAGGACGGTAG  20
Template        464877  ....................  464896

Reverse primer  1       AGGCCTTCGGTAATGATCTTGT  22
Template        465232  ......................  465211
```

>CP075829.1 Pseudomonas aeruginosa strain PaLo25 chromosome, complete genome

```
product length = 356
Forward primer  1       CTGAAGCTGGAGGACGGTAG  20
Template        476239  ....................  476258

Reverse primer  1       AGGCCTTCGGTAATGATCTTGT  22
Template        476594  ......................  476573
```

>CP075828.1 Pseudomonas aeruginosa strain PaLo26 chromosome, complete genome

```
product length = 356
Forward primer  1       CTGAAGCTGGAGGACGGTAG  20
Template        470339  ....................  470358

Reverse primer  1       AGGCCTTCGGTAATGATCTTGT  22
Template        470694  ......................  470673
```

>CP075826.1 Pseudomonas aeruginosa strain PaLo29 chromosome, complete genome

```
product length = 356
Forward primer  1       CTGAAGCTGGAGGACGGTAG  20
Template        421551  ....................  421570

Reverse primer  1       AGGCCTTCGGTAATGATCTTGT  22
Template        421906  ......................  421885
```

>CP075825.1 Pseudomonas aeruginosa strain PaLo30 chromosome, complete genome

```
product length = 356
Forward primer  1       CTGAAGCTGGAGGACGGTAG  20
Template        468911  ....................  468930

Reverse primer  1       AGGCCTTCGGTAATGATCTTGT  22
Template        469266  ......................  469245
```

>CP075824.1 Pseudomonas aeruginosa strain PaLo31 chromosome, complete genome

```
product length = 356
Forward primer  1       CTGAAGCTGGAGGACGGTAG  20
Template        507594  ....................  507613

Reverse primer  1       AGGCCTTCGGTAATGATCTTGT  22
Template        507949  ......................  507928
```

>CP075823.1 Pseudomonas aeruginosa strain PaLo32 chromosome, complete genome

```
product length = 356
Forward primer  1       CTGAAGCTGGAGGACGGTAG  20
Template        517983  ....................  518002

Reverse primer  1       AGGCCTTCGGTAATGATCTTGT  22
Template        518338  ......................  518317
```

>CP075822.1 Pseudomonas aeruginosa strain PaLo34 chromosome, complete genome

```
product length = 356
Forward primer  1       CTGAAGCTGGAGGACGGTAG  20
Template        468911  ....................  468930

Reverse primer  1       AGGCCTTCGGTAATGATCTTGT  22
Template        469266  ......................  469245
```

>CP075821.1 Pseudomonas aeruginosa strain PaLo35 chromosome, complete genome

```
product length = 356
Forward primer  1       CTGAAGCTGGAGGACGGTAG  20
Template        464368  ....................  464387

Reverse primer  1       AGGCCTTCGGTAATGATCTTGT  22
Template        464723  ......................  464702
```

>CP075820.1 Pseudomonas aeruginosa strain PaLo36 chromosome, complete genome

```
product length = 356
Forward primer  1       CTGAAGCTGGAGGACGGTAG  20
Template        467331  ....................  467350

Reverse primer  1       AGGCCTTCGGTAATGATCTTGT  22
Template        467686  ......................  467665
```

>CP075819.1 Pseudomonas aeruginosa strain PaLo37 chromosome, complete genome

```
product length = 356
Forward primer  1       CTGAAGCTGGAGGACGGTAG  20
Template        468873  ....................  468892

Reverse primer  1       AGGCCTTCGGTAATGATCTTGT  22
Template        469228  ......................  469207
```

>CP075818.1 Pseudomonas aeruginosa strain PaLo38 chromosome, complete genome

```
product length = 356
Forward primer  1       CTGAAGCTGGAGGACGGTAG  20
Template        468904  ....................  468923

Reverse primer  1       AGGCCTTCGGTAATGATCTTGT  22
Template        469259  ......................  469238
```

>CP075816.1 Pseudomonas aeruginosa strain PaLo40 chromosome, complete genome

```
product length = 356
Forward primer  1       CTGAAGCTGGAGGACGGTAG  20
Template        468913  ....................  468932

Reverse primer  1       AGGCCTTCGGTAATGATCTTGT  22
Template        469268  ......................  469247
```

>CP075815.1 Pseudomonas aeruginosa strain PaLo43 chromosome, complete genome

```
product length = 356
Forward primer  1       CTGAAGCTGGAGGACGGTAG  20
Template        468817  ....................  468836

Reverse primer  1       AGGCCTTCGGTAATGATCTTGT  22
Template        469172  ......................  469151
```

>CP075813.1 Pseudomonas aeruginosa strain PaLo45 chromosome, complete genome

```
product length = 356
Forward primer  1       CTGAAGCTGGAGGACGGTAG  20
Template        465852  ....................  465871

Reverse primer  1       AGGCCTTCGGTAATGATCTTGT  22
Template        466207  ......................  466186
```

>CP075812.1 Pseudomonas aeruginosa strain PaLo46 chromosome, complete genome

```
product length = 356
Forward primer  1       CTGAAGCTGGAGGACGGTAG  20
Template        517930  ....................  517949

Reverse primer  1       AGGCCTTCGGTAATGATCTTGT  22
Template        518285  ......................  518264
```

>CP075810.1 Pseudomonas aeruginosa strain PaLo166 chromosome, complete genome

```
product length = 356
Forward primer  1       CTGAAGCTGGAGGACGGTAG  20
Template        476359  ....................  476378

Reverse primer  1       AGGCCTTCGGTAATGATCTTGT  22
Template        476714  ......................  476693
```

>CP075809.1 Pseudomonas aeruginosa strain PaLo170 chromosome, complete genome

```
product length = 356
Forward primer  1       CTGAAGCTGGAGGACGGTAG  20
Template        467437  ....................  467456

Reverse primer  1       AGGCCTTCGGTAATGATCTTGT  22
Template        467792  ......................  467771
```

>CP075808.1 Pseudomonas aeruginosa strain PaLo185 chromosome

```
product length = 356
Forward primer  1       CTGAAGCTGGAGGACGGTAG  20
Template        467908  ....................  467927

Reverse primer  1       AGGCCTTCGGTAATGATCTTGT  22
Template        468263  ......................  468242
```

>CP075807.1 Pseudomonas aeruginosa strain PaLo191 chromosome, complete genome

```
product length = 356
Forward primer  1       CTGAAGCTGGAGGACGGTAG  20
Template        466159  ....................  466178

Reverse primer  1       AGGCCTTCGGTAATGATCTTGT  22
Template        466514  ......................  466493
```

>CP075806.1 Pseudomonas aeruginosa strain PaLo226 chromosome, complete genome

```
product length = 356
Forward primer  1       CTGAAGCTGGAGGACGGTAG  20
Template        465119  ....................  465138

Reverse primer  1       AGGCCTTCGGTAATGATCTTGT  22
Template        465474  ......................  465453
```

>CP075805.1 Pseudomonas aeruginosa strain PaLo227 chromosome, complete genome

```
product length = 356
Forward primer  1       CTGAAGCTGGAGGACGGTAG  20
Template        465130  ....................  465149

Reverse primer  1       AGGCCTTCGGTAATGATCTTGT  22
Template        465485  ......................  465464
```

>CP075804.1 Pseudomonas aeruginosa strain PaLo228 chromosome, complete genome

```
product length = 356
Forward primer  1       CTGAAGCTGGAGGACGGTAG  20
Template        465119  ....................  465138

Reverse primer  1       AGGCCTTCGGTAATGATCTTGT  22
Template        465474  ......................  465453
```

>CP075803.1 Pseudomonas aeruginosa strain PaLo229 chromosome, complete genome

```
product length = 356
Forward primer  1       CTGAAGCTGGAGGACGGTAG  20
Template        465127  ....................  465146

Reverse primer  1       AGGCCTTCGGTAATGATCTTGT  22
Template        465482  ......................  465461
```

>CP075802.1 Pseudomonas aeruginosa strain PaLo240 chromosome, complete genome

```
product length = 356
Forward primer  1       CTGAAGCTGGAGGACGGTAG  20
Template        518001  ....................  518020

Reverse primer  1       AGGCCTTCGGTAATGATCTTGT  22
Template        518356  ......................  518335
```

>CP075801.1 Pseudomonas aeruginosa strain PaLo249 chromosome, complete genome

```
product length = 356
Forward primer  1       CTGAAGCTGGAGGACGGTAG  20
Template        465124  ....................  465143

Reverse primer  1       AGGCCTTCGGTAATGATCTTGT  22
Template        465479  ......................  465458
```

>CP075800.1 Pseudomonas aeruginosa strain PaLo297 chromosome, complete genome

```
product length = 356
Forward primer  1       CTGAAGCTGGAGGACGGTAG  20
Template        466693  ....................  466712

Reverse primer  1       AGGCCTTCGGTAATGATCTTGT  22
Template        467048  ......................  467027
```

>CP075799.1 Pseudomonas aeruginosa strain PaLo310 chromosome, complete genome

```
product length = 356
Forward primer  1       CTGAAGCTGGAGGACGGTAG  20
Template        468915  ....................  468934

Reverse primer  1       AGGCCTTCGGTAATGATCTTGT  22
Template        469270  ......................  469249
```

>CP075798.1 Pseudomonas aeruginosa strain PaLo323 chromosome, complete genome

```
product length = 356
Forward primer  1       CTGAAGCTGGAGGACGGTAG  20
Template        466204  ....................  466223

Reverse primer  1       AGGCCTTCGGTAATGATCTTGT  22
Template        466559  ......................  466538
```

>CP075796.1 Pseudomonas aeruginosa strain PaLo402 chromosome, complete genome

```
product length = 356
Forward primer  1       CTGAAGCTGGAGGACGGTAG  20
Template        454912  ....................  454931

Reverse primer  1       AGGCCTTCGGTAATGATCTTGT  22
Template        455267  ......................  455246
```

>CP075787.1 Pseudomonas aeruginosa strain PaLo504 chromosome, complete genome

```
product length = 356
Forward primer  1       CTGAAGCTGGAGGACGGTAG  20
Template        479569  ....................  479588

Reverse primer  1       AGGCCTTCGGTAATGATCTTGT  22
Template        479924  ......................  479903
```

>CP075785.1 Pseudomonas aeruginosa strain PaLo505 chromosome, complete genome

```
product length = 356
Forward primer  1       CTGAAGCTGGAGGACGGTAG  20
Template        471896  ....................  471915

Reverse primer  1       AGGCCTTCGGTAATGATCTTGT  22
Template        472251  ......................  472230
```

>CP075784.1 Pseudomonas aeruginosa strain PaLo507 chromosome, complete genome

```
product length = 356
Forward primer  1       CTGAAGCTGGAGGACGGTAG  20
Template        470134  ....................  470153

Reverse primer  1       AGGCCTTCGGTAATGATCTTGT  22
Template        470489  ......................  470468
```

>CP075783.1 Pseudomonas aeruginosa strain PaLo508 chromosome, complete genome

```
product length = 356
Forward primer  1       CTGAAGCTGGAGGACGGTAG  20
Template        473808  ....................  473827

Reverse primer  1       AGGCCTTCGGTAATGATCTTGT  22
Template        474163  ......................  474142
```

>CP075782.1 Pseudomonas aeruginosa strain PaLo509 chromosome, complete genome

```
product length = 356
Forward primer  1       CTGAAGCTGGAGGACGGTAG  20
Template        473772  ....................  473791

Reverse primer  1       AGGCCTTCGGTAATGATCTTGT  22
Template        474127  ......................  474106
```

>CP075781.1 Pseudomonas aeruginosa strain PaLo512 chromosome, complete genome

```
product length = 356
Forward primer  1       CTGAAGCTGGAGGACGGTAG  20
Template        467353  ....................  467372

Reverse primer  1       AGGCCTTCGGTAATGATCTTGT  22
Template        467708  ......................  467687
```

>CP075780.1 Pseudomonas aeruginosa strain PaLo524 chromosome, complete genome

```
product length = 356
Forward primer  1       CTGAAGCTGGAGGACGGTAG  20
Template        465320  ....................  465339

Reverse primer  1       AGGCCTTCGGTAATGATCTTGT  22
Template        465675  ......................  465654
```

>CP075779.1 Pseudomonas aeruginosa strain PaLo526 chromosome, complete genome

```
product length = 356
Forward primer  1       CTGAAGCTGGAGGACGGTAG  20
Template        469805  ....................  469824

Reverse primer  1       AGGCCTTCGGTAATGATCTTGT  22
Template        470160  ......................  470139
```

>CP075777.1 Pseudomonas aeruginosa strain PaLo528 chromosome, complete genome

```
product length = 356
Forward primer  1       CTGAAGCTGGAGGACGGTAG  20
Template        471774  ....................  471793

Reverse primer  1       AGGCCTTCGGTAATGATCTTGT  22
Template        472129  ......................  472108
```

>CP075776.1 Pseudomonas aeruginosa strain PaLo529 chromosome, complete genome

```
product length = 356
Forward primer  1       CTGAAGCTGGAGGACGGTAG  20
Template        478524  ....................  478543

Reverse primer  1       AGGCCTTCGGTAATGATCTTGT  22
Template        478879  ......................  478858
```

>CP075773.1 Pseudomonas aeruginosa strain PaLo530 chromosome, complete genome

```
product length = 356
Forward primer  1       CTGAAGCTGGAGGACGGTAG  20
Template        520655  ....................  520674

Reverse primer  1       AGGCCTTCGGTAATGATCTTGT  22
Template        521010  ......................  520989
```

>CP075771.1 Pseudomonas aeruginosa strain PaLo532 chromosome, complete genome

```
product length = 356
Forward primer  1       CTGAAGCTGGAGGACGGTAG  20
Template        467361  ....................  467380

Reverse primer  1       AGGCCTTCGGTAATGATCTTGT  22
Template        467716  ......................  467695
```

>CP075769.1 Pseudomonas aeruginosa strain PaLo533 chromosome, complete genome

```
product length = 356
Forward primer  1       CTGAAGCTGGAGGACGGTAG  20
Template        747521  ....................  747540

Reverse primer  1       AGGCCTTCGGTAATGATCTTGT  22
Template        747876  ......................  747855
```

>CP075768.1 Pseudomonas aeruginosa strain PaLo535 chromosome

```
product length = 356
Forward primer  1       CTGAAGCTGGAGGACGGTAG  20
Template        371664  ....................  371683

Reverse primer  1       AGGCCTTCGGTAATGATCTTGT  22
Template        372019  ......................  371998
```

>CP075767.1 Pseudomonas aeruginosa strain PaLo536 chromosome, complete genome

```
product length = 356
Forward primer  1       CTGAAGCTGGAGGACGGTAG  20
Template        466190  ....................  466209

Reverse primer  1       AGGCCTTCGGTAATGATCTTGT  22
Template        466545  ......................  466524
```

>CP075766.1 Pseudomonas aeruginosa strain PaLo538 chromosome, complete genome

```
product length = 356
Forward primer  1       CTGAAGCTGGAGGACGGTAG  20
Template        509425  ....................  509444

Reverse primer  1       AGGCCTTCGGTAATGATCTTGT  22
Template        509780  ......................  509759
```

>CP075765.1 Pseudomonas aeruginosa strain PaLo539 chromosome, complete genome

```
product length = 356
Forward primer  1       CTGAAGCTGGAGGACGGTAG  20
Template        471698  ....................  471717

Reverse primer  1       AGGCCTTCGGTAATGATCTTGT  22
Template        472053  ......................  472032
```

>CP075764.1 Pseudomonas aeruginosa strain PaLo541 chromosome, complete genome

```
product length = 356
Forward primer  1       CTGAAGCTGGAGGACGGTAG  20
Template        463760  ....................  463779

Reverse primer  1       AGGCCTTCGGTAATGATCTTGT  22
Template        464115  ......................  464094
```

>CP075763.1 Pseudomonas aeruginosa strain PaLo543 chromosome, complete genome

```
product length = 356
Forward primer  1       CTGAAGCTGGAGGACGGTAG  20
Template        559150  ....................  559169

Reverse primer  1       AGGCCTTCGGTAATGATCTTGT  22
Template        559505  ......................  559484
```

>CP075762.1 Pseudomonas aeruginosa strain PaLo544 chromosome, complete genome

```
product length = 356
Forward primer  1       CTGAAGCTGGAGGACGGTAG  20
Template        468745  ....................  468764

Reverse primer  1       AGGCCTTCGGTAATGATCTTGT  22
Template        469100  ......................  469079
```

>CP075761.1 Pseudomonas aeruginosa strain PaLo545 chromosome

```
product length = 356
Forward primer  1       CTGAAGCTGGAGGACGGTAG  20
Template        469969  ....................  469988

Reverse primer  1       AGGCCTTCGGTAATGATCTTGT  22
Template        470324  ......................  470303
```

>CP075760.1 Pseudomonas aeruginosa strain PaLo550 chromosome, complete genome

```
product length = 356
Forward primer  1       CTGAAGCTGGAGGACGGTAG  20
Template        467634  ....................  467653

Reverse primer  1       AGGCCTTCGGTAATGATCTTGT  22
Template        467989  ......................  467968
```

>CP075757.1 Pseudomonas aeruginosa strain PaLo552 chromosome, complete genome

```
product length = 356
Forward primer  1       CTGAAGCTGGAGGACGGTAG  20
Template        477171  ....................  477190

Reverse primer  1       AGGCCTTCGGTAATGATCTTGT  22
Template        477526  ......................  477505
```

>CP075755.1 Pseudomonas aeruginosa strain PaLo553 chromosome, complete genome

```
product length = 356
Forward primer  1       CTGAAGCTGGAGGACGGTAG  20
Template        486978  ....................  486997

Reverse primer  1       AGGCCTTCGGTAATGATCTTGT  22
Template        487333  ......................  487312
```

>CP075754.1 Pseudomonas aeruginosa strain PaLo555 chromosome, complete genome

```
product length = 356
Forward primer  1       CTGAAGCTGGAGGACGGTAG  20
Template        473521  ....................  473540

Reverse primer  1       AGGCCTTCGGTAATGATCTTGT  22
Template        473876  ......................  473855
```

>CP075753.1 Pseudomonas aeruginosa strain PaLo556 chromosome, complete genome

```
product length = 356
Forward primer  1       CTGAAGCTGGAGGACGGTAG  20
Template        468850  ....................  468869

Reverse primer  1       AGGCCTTCGGTAATGATCTTGT  22
Template        469205  ......................  469184
```

>CP075752.1 Pseudomonas aeruginosa strain PaLo557 chromosome, complete genome

```
product length = 356
Forward primer  1       CTGAAGCTGGAGGACGGTAG  20
Template        556054  ....................  556073

Reverse primer  1       AGGCCTTCGGTAATGATCTTGT  22
Template        556409  ......................  556388
```

>CP075751.1 Pseudomonas aeruginosa strain PaLo561 chromosome, complete genome

```
product length = 356
Forward primer  1       CTGAAGCTGGAGGACGGTAG  20
Template        474684  ....................  474703

Reverse primer  1       AGGCCTTCGGTAATGATCTTGT  22
Template        475039  ......................  475018
```

>CP075750.1 Pseudomonas aeruginosa strain PaLo563 chromosome, complete genome

```
product length = 356
Forward primer  1       CTGAAGCTGGAGGACGGTAG  20
Template        473132  ....................  473151

Reverse primer  1       AGGCCTTCGGTAATGATCTTGT  22
Template        473487  ......................  473466
```

>CP075749.1 Pseudomonas aeruginosa strain PaLo564 chromosome, complete genome

```
product length = 356
Forward primer  1       CTGAAGCTGGAGGACGGTAG  20
Template        507442  ....................  507461

Reverse primer  1       AGGCCTTCGGTAATGATCTTGT  22
Template        507797  ......................  507776
```

>CP075748.1 Pseudomonas aeruginosa strain PaLo565 chromosome, complete genome

```
product length = 356
Forward primer  1       CTGAAGCTGGAGGACGGTAG  20
Template        473133  ....................  473152

Reverse primer  1       AGGCCTTCGGTAATGATCTTGT  22
Template        473488  ......................  473467
```

>CP116723.1 Pseudomonas aeruginosa strain 2872 chromosome

```
product length = 356
Forward primer  1        CTGAAGCTGGAGGACGGTAG  20
Template        6655388  ....................  6655369

Reverse primer  1        AGGCCTTCGGTAATGATCTTGT  22
Template        6655033  ......................  6655054
```

>CP116725.1 Pseudomonas aeruginosa strain 2881 chromosome, complete genome

```
product length = 356
Forward primer  1       CTGAAGCTGGAGGACGGTAG  20
Template        530068  ....................  530087

Reverse primer  1       AGGCCTTCGGTAATGATCTTGT  22
Template        530423  ......................  530402
```

>CP116722.1 Pseudomonas aeruginosa strain 2868 chromosome, complete genome

```
product length = 356
Forward primer  1       CTGAAGCTGGAGGACGGTAG  20
Template        535443  ....................  535462

Reverse primer  1       AGGCCTTCGGTAATGATCTTGT  22
Template        535798  ......................  535777
```

>CP116717.1 Pseudomonas aeruginosa strain 2857 chromosome, complete genome

```
product length = 356
Forward primer  1       CTGAAGCTGGAGGACGGTAG  20
Template        549681  ....................  549700

Reverse primer  1       AGGCCTTCGGTAATGATCTTGT  22
Template        550036  ......................  550015
```

>CP116727.1 Pseudomonas aeruginosa strain 2875 chromosome, complete genome

```
product length = 356
Forward primer  1       CTGAAGCTGGAGGACGGTAG  20
Template        532625  ....................  532644

Reverse primer  1       AGGCCTTCGGTAATGATCTTGT  22
Template        532980  ......................  532959
```

>CP116718.1 Pseudomonas aeruginosa strain 2858 chromosome, complete genome

```
product length = 356
Forward primer  1       CTGAAGCTGGAGGACGGTAG  20
Template        509278  ....................  509297

Reverse primer  1       AGGCCTTCGGTAATGATCTTGT  22
Template        509633  ......................  509612
```

>CP116720.1 Pseudomonas aeruginosa strain 2866 chromosome, complete genome

```
product length = 356
Forward primer  1       CTGAAGCTGGAGGACGGTAG  20
Template        529453  ....................  529472

Reverse primer  1       AGGCCTTCGGTAATGATCTTGT  22
Template        529808  ......................  529787
```

>CP116724.1 Pseudomonas aeruginosa strain 2880 chromosome, complete genome

```
product length = 356
Forward primer  1       CTGAAGCTGGAGGACGGTAG  20
Template        545153  ....................  545172

Reverse primer  1       AGGCCTTCGGTAATGATCTTGT  22
Template        545508  ......................  545487
```

>CP116721.1 Pseudomonas aeruginosa strain 2867 chromosome, complete genome

```
product length = 356
Forward primer  1       CTGAAGCTGGAGGACGGTAG  20
Template        552322  ....................  552341

Reverse primer  1       AGGCCTTCGGTAATGATCTTGT  22
Template        552677  ......................  552656
```

>CP116715.1 Pseudomonas aeruginosa strain 2856 chromosome, complete genome

```
product length = 356
Forward primer  1       CTGAAGCTGGAGGACGGTAG  20
Template        532433  ....................  532452

Reverse primer  1       AGGCCTTCGGTAATGATCTTGT  22
Template        532788  ......................  532767
```

>CP106784.1 Pseudomonas aeruginosa strain NY5085 chromosome, complete genome

```
product length = 356
Forward primer  1       CTGAAGCTGGAGGACGGTAG  20
Template        478266  ....................  478285

Reverse primer  1       AGGCCTTCGGTAATGATCTTGT  22
Template        478621  ......................  478600
```

>CP096913.1 Pseudomonas aeruginosa strain NY7610 chromosome, complete genome

```
product length = 356
Forward primer  1       CTGAAGCTGGAGGACGGTAG  20
Template        489886  ....................  489905

Reverse primer  1       AGGCCTTCGGTAATGATCTTGT  22
Template        490241  ......................  490220
```

>CP096912.1 Pseudomonas aeruginosa strain NY7770 chromosome, complete genome

```
product length = 356
Forward primer  1       CTGAAGCTGGAGGACGGTAG  20
Template        455743  ....................  455762

Reverse primer  1       AGGCCTTCGGTAATGATCTTGT  22
Template        456098  ......................  456077
```

>CP096909.1 Pseudomonas aeruginosa strain NY8688 chromosome, complete genome

```
product length = 356
Forward primer  1       CTGAAGCTGGAGGACGGTAG  20
Template        496241  ....................  496260

Reverse primer  1       AGGCCTTCGGTAATGATCTTGT  22
Template        496596  ......................  496575
```

>CP111030.1 Pseudomonas aeruginosa strain PALA38 chromosome, complete genome

```
product length = 356
Forward primer  1       CTGAAGCTGGAGGACGGTAG  20
Template        488165  ....................  488184

Reverse primer  1       AGGCCTTCGGTAATGATCTTGT  22
Template        488520  ......................  488499
```

>CP111032.1 Pseudomonas aeruginosa strain PALA54 chromosome, complete genome

```
product length = 356
Forward primer  1       CTGAAGCTGGAGGACGGTAG  20
Template        489280  ....................  489299

Reverse primer  1       AGGCCTTCGGTAATGATCTTGT  22
Template        489635  ......................  489614
```

>CP111034.1 Pseudomonas aeruginosa strain PALA50 chromosome, complete genome

```
product length = 356
Forward primer  1       CTGAAGCTGGAGGACGGTAG  20
Template        502337  ....................  502356

Reverse primer  1       AGGCCTTCGGTAATGATCTTGT  22
Template        502692  ......................  502671
```

>CP110353.1 Pseudomonas aeruginosa strain PALA48 chromosome, complete genome

```
product length = 356
Forward primer  1       CTGAAGCTGGAGGACGGTAG  20
Template        466021  ....................  466040

Reverse primer  1       AGGCCTTCGGTAATGATCTTGT  22
Template        466376  ......................  466355
```

>CP110352.1 Pseudomonas aeruginosa strain PALA47 chromosome, complete genome

```
product length = 356
Forward primer  1       CTGAAGCTGGAGGACGGTAG  20
Template        477651  ....................  477670

Reverse primer  1       AGGCCTTCGGTAATGATCTTGT  22
Template        478006  ......................  477985
```

>CP110351.1 Pseudomonas aeruginosa strain PALA45 chromosome, complete genome

```
product length = 356
Forward primer  1       CTGAAGCTGGAGGACGGTAG  20
Template        465933  ....................  465952

Reverse primer  1       AGGCCTTCGGTAATGATCTTGT  22
Template        466288  ......................  466267
```

>CP110350.1 Pseudomonas aeruginosa strain PALA44 chromosome, complete genome

```
product length = 356
Forward primer  1        CTGAAGCTGGAGGACGGTAG  20
Template        1583783  ....................  1583802

Reverse primer  1        AGGCCTTCGGTAATGATCTTGT  22
Template        1584138  ......................  1584117
```

>CP109932.1 Pseudomonas aeruginosa strain PALA43 chromosome, complete genome

```
product length = 356
Forward primer  1        CTGAAGCTGGAGGACGGTAG  20
Template        1548224  ....................  1548243

Reverse primer  1        AGGCCTTCGGTAATGATCTTGT  22
Template        1548579  ......................  1548558
```

>CP109931.1 Pseudomonas aeruginosa strain PALA42 chromosome, complete genome

```
product length = 356
Forward primer  1       CTGAAGCTGGAGGACGGTAG  20
Template        465316  ....................  465335

Reverse primer  1       AGGCCTTCGGTAATGATCTTGT  22
Template        465671  ......................  465650
```

>CP110349.1 Pseudomonas aeruginosa strain PALA40 chromosome, complete genome

```
product length = 356
Forward primer  1       CTGAAGCTGGAGGACGGTAG  20
Template        579514  ....................  579533

Reverse primer  1       AGGCCTTCGGTAATGATCTTGT  22
Template        579869  ......................  579848
```

>CP109920.1 Pseudomonas aeruginosa strain PALA39 chromosome, complete genome

```
product length = 356
Forward primer  1       CTGAAGCTGGAGGACGGTAG  20
Template        465027  ....................  465046

Reverse primer  1       AGGCCTTCGGTAATGATCTTGT  22
Template        465382  ......................  465361
```

>CP110348.1 Pseudomonas aeruginosa strain PALA36 chromosome, complete genome

```
product length = 356
Forward primer  1       CTGAAGCTGGAGGACGGTAG  20
Template        487408  ....................  487427

Reverse primer  1       AGGCCTTCGGTAATGATCTTGT  22
Template        487763  ......................  487742
```

>CP109919.1 Pseudomonas aeruginosa strain PALA56 chromosome, complete genome

```
product length = 356
Forward primer  1       CTGAAGCTGGAGGACGGTAG  20
Template        466022  ....................  466041

Reverse primer  1       AGGCCTTCGGTAATGATCTTGT  22
Template        466377  ......................  466356
```

>CP109918.1 Pseudomonas aeruginosa strain PALA55 chromosome, complete genome

```
product length = 356
Forward primer  1       CTGAAGCTGGAGGACGGTAG  20
Template        470061  ....................  470080

Reverse primer  1       AGGCCTTCGGTAATGATCTTGT  22
Template        470416  ......................  470395
```

>CP109856.1 Pseudomonas aeruginosa strain PALA53 chromosome, complete genome

```
product length = 356
Forward primer  1       CTGAAGCTGGAGGACGGTAG  20
Template        473514  ....................  473533

Reverse primer  1       AGGCCTTCGGTAATGATCTTGT  22
Template        473869  ......................  473848
```

>CP109851.1 Pseudomonas aeruginosa strain PALA51 chromosome, complete genome

```
product length = 356
Forward primer  1       CTGAAGCTGGAGGACGGTAG  20
Template        502858  ....................  502877

Reverse primer  1       AGGCCTTCGGTAATGATCTTGT  22
Template        503213  ......................  503192
```

>CP109849.1 Pseudomonas aeruginosa strain PALA34 chromosome, complete genome

```
product length = 356
Forward primer  1       CTGAAGCTGGAGGACGGTAG  20
Template        482400  ....................  482419

Reverse primer  1       AGGCCTTCGGTAATGATCTTGT  22
Template        482755  ......................  482734
```

>CP109845.1 Pseudomonas aeruginosa strain PALA33 chromosome, complete genome

```
product length = 356
Forward primer  1       CTGAAGCTGGAGGACGGTAG  20
Template        567868  ....................  567887

Reverse primer  1       AGGCCTTCGGTAATGATCTTGT  22
Template        568223  ......................  568202
```

>CP109844.1 Pseudomonas aeruginosa strain PALA32 chromosome, complete genome

```
product length = 356
Forward primer  1       CTGAAGCTGGAGGACGGTAG  20
Template        474616  ....................  474635

Reverse primer  1       AGGCCTTCGGTAATGATCTTGT  22
Template        474971  ......................  474950
```

>CP110345.1 Pseudomonas aeruginosa strain PALA30 chromosome, complete genome

```
product length = 356
Forward primer  1       CTGAAGCTGGAGGACGGTAG  20
Template        547800  ....................  547819

Reverse primer  1       AGGCCTTCGGTAATGATCTTGT  22
Template        548155  ......................  548134
```

>CP109843.1 Pseudomonas aeruginosa strain PALA29 chromosome, complete genome

```
product length = 356
Forward primer  1       CTGAAGCTGGAGGACGGTAG  20
Template        471443  ....................  471462

Reverse primer  1       AGGCCTTCGGTAATGATCTTGT  22
Template        471798  ......................  471777
```

>CP109835.1 Pseudomonas aeruginosa strain PALA26 chromosome, complete genome

```
product length = 356
Forward primer  1       CTGAAGCTGGAGGACGGTAG  20
Template        486983  ....................  487002

Reverse primer  1       AGGCCTTCGGTAATGATCTTGT  22
Template        487338  ......................  487317
```

>CP109834.1 Pseudomonas aeruginosa strain PALA25 chromosome, complete genome

```
product length = 356
Forward primer  1       CTGAAGCTGGAGGACGGTAG  20
Template        473546  ....................  473565

Reverse primer  1       AGGCCTTCGGTAATGATCTTGT  22
Template        473901  ......................  473880
```

>CP110344.1 Pseudomonas aeruginosa strain PALA24 chromosome, complete genome

```
product length = 356
Forward primer  1       CTGAAGCTGGAGGACGGTAG  20
Template        474046  ....................  474065

Reverse primer  1       AGGCCTTCGGTAATGATCTTGT  22
Template        474401  ......................  474380
```

>CP109833.1 Pseudomonas aeruginosa strain PALA23 chromosome, complete genome

```
product length = 356
Forward primer  1       CTGAAGCTGGAGGACGGTAG  20
Template        481730  ....................  481749

Reverse primer  1       AGGCCTTCGGTAATGATCTTGT  22
Template        482085  ......................  482064
```

>CP107275.1 Pseudomonas aeruginosa strain PALA22 chromosome, complete genome

```
product length = 356
Forward primer  1       CTGAAGCTGGAGGACGGTAG  20
Template        468831  ....................  468850

Reverse primer  1       AGGCCTTCGGTAATGATCTTGT  22
Template        469186  ......................  469165
```

>CP107064.1 Pseudomonas aeruginosa strain PALA20 chromosome, complete genome

```
product length = 356
Forward primer  1        CTGAAGCTGGAGGACGGTAG  20
Template        1089641  ....................  1089622

Reverse primer  1        AGGCCTTCGGTAATGATCTTGT  22
Template        1089286  ......................  1089307
```

>CP107029.1 Pseudomonas aeruginosa strain PALA19 chromosome, complete genome

```
product length = 356
Forward primer  1       CTGAAGCTGGAGGACGGTAG  20
Template        464742  ....................  464761

Reverse primer  1       AGGCCTTCGGTAATGATCTTGT  22
Template        465097  ......................  465076
```

>CP106745.1 Pseudomonas aeruginosa strain PALA17 chromosome, complete genome

```
product length = 356
Forward primer  1        CTGAAGCTGGAGGACGGTAG  20
Template        6187503  ....................  6187484

Reverse primer  1        AGGCCTTCGGTAATGATCTTGT  22
Template        6187148  ......................  6187169
```

>CP106744.1 Pseudomonas aeruginosa strain PALA16 chromosome, complete genome

```
product length = 356
Forward primer  1       CTGAAGCTGGAGGACGGTAG  20
Template        482374  ....................  482393

Reverse primer  1       AGGCCTTCGGTAATGATCTTGT  22
Template        482729  ......................  482708
```

>CP106743.1 Pseudomonas aeruginosa strain PALA15 chromosome, complete genome

```
product length = 356
Forward primer  1       CTGAAGCTGGAGGACGGTAG  20
Template        473412  ....................  473431

Reverse primer  1       AGGCCTTCGGTAATGATCTTGT  22
Template        473767  ......................  473746
```

>CP106742.1 Pseudomonas aeruginosa strain PALA14 chromosome, complete genome

```
product length = 356
Forward primer  1       CTGAAGCTGGAGGACGGTAG  20
Template        507852  ....................  507871

Reverse primer  1       AGGCCTTCGGTAATGATCTTGT  22
Template        508207  ......................  508186
```

>CP106682.1 Pseudomonas aeruginosa strain PALA13 chromosome, complete genome

```
product length = 356
Forward primer  1       CTGAAGCTGGAGGACGGTAG  20
Template        518001  ....................  518020

Reverse primer  1       AGGCCTTCGGTAATGATCTTGT  22
Template        518356  ......................  518335
```

>CP106681.1 Pseudomonas aeruginosa strain PALA12 chromosome, complete genome

```
product length = 356
Forward primer  1        CTGAAGCTGGAGGACGGTAG  20
Template        5066653  ....................  5066634

Reverse primer  1        AGGCCTTCGGTAATGATCTTGT  22
Template        5066298  ......................  5066319
```

>CP106680.1 Pseudomonas aeruginosa strain PALA11 chromosome, complete genome

```
product length = 356
Forward primer  1       CTGAAGCTGGAGGACGGTAG  20
Template        466584  ....................  466603

Reverse primer  1       AGGCCTTCGGTAATGATCTTGT  22
Template        466939  ......................  466918
```

>CP104870.1 Pseudomonas aeruginosa strain PALA9 chromosome, complete genome

```
product length = 356
Forward primer  1       CTGAAGCTGGAGGACGGTAG  20
Template        544777  ....................  544796

Reverse primer  1       AGGCCTTCGGTAATGATCTTGT  22
Template        545132  ......................  545111
```

>CP104869.1 Pseudomonas aeruginosa strain PALA8 chromosome, complete genome

```
product length = 356
Forward primer  1       CTGAAGCTGGAGGACGGTAG  20
Template        477912  ....................  477931

Reverse primer  1       AGGCCTTCGGTAATGATCTTGT  22
Template        478267  ......................  478246
```

>CP104868.1 Pseudomonas aeruginosa strain PALA7 chromosome, complete genome

```
product length = 356
Forward primer  1       CTGAAGCTGGAGGACGGTAG  20
Template        477651  ....................  477670

Reverse primer  1       AGGCCTTCGGTAATGATCTTGT  22
Template        478006  ......................  477985
```

>CP104867.1 Pseudomonas aeruginosa strain PALA6 chromosome, complete genome

```
product length = 356
Forward primer  1       CTGAAGCTGGAGGACGGTAG  20
Template        471561  ....................  471580

Reverse primer  1       AGGCCTTCGGTAATGATCTTGT  22
Template        471916  ......................  471895
```

>CP104866.1 Pseudomonas aeruginosa strain PALA4 chromosome, complete genome

```
product length = 356
Forward primer  1        CTGAAGCTGGAGGACGGTAG  20
Template        4108487  ....................  4108468

Reverse primer  1        AGGCCTTCGGTAATGATCTTGT  22
Template        4108132  ......................  4108153
```

>CP104865.1 Pseudomonas aeruginosa strain PALA2 chromosome, complete genome

```
product length = 356
Forward primer  1       CTGAAGCTGGAGGACGGTAG  20
Template        474016  ....................  474035

Reverse primer  1       AGGCCTTCGGTAATGATCTTGT  22
Template        474371  ......................  474350
```

>CP104254.1 Pseudomonas aeruginosa strain PALA1 chromosome, complete genome

```
product length = 356
Forward primer  1       CTGAAGCTGGAGGACGGTAG  20
Template        463780  ....................  463799

Reverse primer  1       AGGCCTTCGGTAATGATCTTGT  22
Template        464135  ......................  464114
```

>CP114374.1 Pseudomonas aeruginosa strain Jade-X chromosome, complete genome

```
product length = 356
Forward primer  1       CTGAAGCTGGAGGACGGTAG  20
Template        468253  ....................  468272

Reverse primer  1       AGGCCTTCGGTAATGATCTTGT  22
Template        468608  ......................  468587
```

>CP113974.1 Pseudomonas aeruginosa strain M6A146 chromosome, complete genome

```
product length = 356
Forward primer  1        CTGAAGCTGGAGGACGGTAG  20
Template        5562032  ....................  5562051

Reverse primer  1        AGGCCTTCGGTAATGATCTTGT  22
Template        5562387  ......................  5562366
```

>CP097555.1 Pseudomonas aeruginosa strain B1.2 chromosome, complete genome

```
product length = 356
Forward primer  1       CTGAAGCTGGAGGACGGTAG  20
Template        480582  ....................  480601

Reverse primer  1       AGGCCTTCGGTAATGATCTTGT  22
Template        480937  ......................  480916
```

>CP097556.1 Pseudomonas aeruginosa strain B2.1 chromosome, complete genome

```
product length = 356
Forward primer  1       CTGAAGCTGGAGGACGGTAG  20
Template        480582  ....................  480601

Reverse primer  1       AGGCCTTCGGTAATGATCTTGT  22
Template        480937  ......................  480916
```

>CP097560.1 Pseudomonas aeruginosa strain C4.2 chromosome, complete genome

```
product length = 356
Forward primer  1       CTGAAGCTGGAGGACGGTAG  20
Template        530359  ....................  530378

Reverse primer  1       AGGCCTTCGGTAATGATCTTGT  22
Template        530714  ......................  530693
```

>CP113230.1 Pseudomonas aeruginosa strain BIAI 160 chromosome, complete genome

```
product length = 356
Forward primer  1        CTGAAGCTGGAGGACGGTAG  20
Template        1220063  ....................  1220044

Reverse primer  1        AGGCCTTCGGTAATGATCTTGT  22
Template        1219708  ......................  1219729
```

>CP113246.1 Pseudomonas aeruginosa strain SMC4386 chromosome, complete genome

```
product length = 356
Forward primer  1        CTGAAGCTGGAGGACGGTAG  20
Template        5786670  ....................  5786651

Reverse primer  1        AGGCCTTCGGTAATGATCTTGT  22
Template        5786315  ......................  5786336
```

>CP113106.1 Pseudomonas aeruginosa strain BIAI 157 chromosome, complete genome

```
product length = 356
Forward primer  1        CTGAAGCTGGAGGACGGTAG  20
Template        5274512  ....................  5274493

Reverse primer  1        AGGCCTTCGGTAATGATCTTGT  22
Template        5274157  ......................  5274178
```

>CP097857.1 Pseudomonas sp. B111 chromosome, complete genome

```
product length = 356
Forward primer  1        CTGAAGCTGGAGGACGGTAG  20
Template        5877171  ....................  5877190

Reverse primer  1        AGGCCTTCGGTAATGATCTTGT  22
Template        5877526  ......................  5877505
```

>CP102441.2 Pseudomonas aeruginosa strain PA30 chromosome, complete genome

```
product length = 356
Forward primer  1       CTGAAGCTGGAGGACGGTAG  20
Template        504441  ....................  504460

Reverse primer  1       AGGCCTTCGGTAATGATCTTGT  22
Template        504796  ......................  504775
```

>CP036492.1 Pseudomonas aeruginosa strain Paer4 chromosome, complete genome

```
product length = 356
Forward primer  1        CTGAAGCTGGAGGACGGTAG  20
Template        5804804  ....................  5804785

Reverse primer  1        AGGCCTTCGGTAATGATCTTGT  22
Template        5804449  ......................  5804470
```

>CP083357.1 Pseudomonas aeruginosa strain KPA143 chromosome, complete genome

```
product length = 356
Forward primer  1        CTGAAGCTGGAGGACGGTAG  20
Template        3471149  ....................  3471168

Reverse primer  1        AGGCCTTCGGTAATGATCTTGT  22
Template        3471504  ......................  3471483
```

>CP083359.1 Pseudomonas aeruginosa strain KPA159 chromosome, complete genome

```
product length = 356
Forward primer  1        CTGAAGCTGGAGGACGGTAG  20
Template        3293714  ....................  3293733

Reverse primer  1        AGGCCTTCGGTAATGATCTTGT  22
Template        3294069  ......................  3294048
```

>CP083358.1 Pseudomonas aeruginosa strain KPA151 chromosome, complete genome

```
product length = 356
Forward primer  1       CTGAAGCTGGAGGACGGTAG  20
Template        247975  ....................  247956

Reverse primer  1       AGGCCTTCGGTAATGATCTTGT  22
Template        247620  ......................  247641
```

>CP083360.1 Pseudomonas aeruginosa strain KPA83 chromosome, complete genome

```
product length = 355
Forward primer  1        CTGAAGCTGGAGGACGGTAG  20
Template        4924110  ....................  4924091

Reverse primer  1        AGGCCTTCGGTAATGATCTTGT  22
Template        4923756  ......................  4923777
```

>CP083355.1 Pseudomonas aeruginosa strain KPA134 chromosome, complete genome

```
product length = 356
Forward primer  1        CTGAAGCTGGAGGACGGTAG  20
Template        1462105  ....................  1462124

Reverse primer  1        AGGCCTTCGGTAATGATCTTGT  22
Template        1462460  ......................  1462439
```

>CP083356.1 Pseudomonas aeruginosa strain KPA140 chromosome, complete genome

```
product length = 356
Forward primer  1       CTGAAGCTGGAGGACGGTAG  20
Template        244560  ....................  244541

Reverse primer  1       AGGCCTTCGGTAATGATCTTGT  22
Template        244205  ......................  244226
```

>CP083353.1 Pseudomonas aeruginosa strain KPA120 chromosome, complete genome

```
product length = 357
Forward primer  1        CTGAAGCTGGAGGACGGTAG  20
Template        3315054  ....................  3315073

Reverse primer  1        AGGCCTTCGGTAATGATCTTGT  22
Template        3315410  ......................  3315389
```

>CP083354.1 Pseudomonas aeruginosa strain KPA124 chromosome, complete genome

```
product length = 355
Forward primer  1        CTGAAGCTGGAGGACGGTAG  20
Template        1510980  ....................  1510999

Reverse primer  1        AGGCCTTCGGTAATGATCTTGT  22
Template        1511334  ......................  1511313
```

>CP083352.1 Pseudomonas aeruginosa strain KPA119 chromosome, complete genome

```
product length = 355
Forward primer  1        CTGAAGCTGGAGGACGGTAG  20
Template        5671371  ....................  5671352

Reverse primer  1        AGGCCTTCGGTAATGATCTTGT  22
Template        5671017  ......................  5671038
```

>CP107257.1 Pseudomonas aeruginosa strain 2019CK-00034 chromosome, complete genome

```
product length = 356
Forward primer  1       CTGAAGCTGGAGGACGGTAG  20
Template        402620  ....................  402601

Reverse primer  1       AGGCCTTCGGTAATGATCTTGT  22
Template        402265  ......................  402286
```

>CP104565.1 Pseudomonas aeruginosa strain HS_121 chromosome, complete genome

```
product length = 356
Forward primer  1       CTGAAGCTGGAGGACGGTAG  20
Template        460705  ....................  460724

Reverse primer  1       AGGCCTTCGGTAATGATCTTGT  22
Template        461060  ......................  461039
```

>CP104567.1 Pseudomonas aeruginosa strain HS_13 chromosome, complete genome

```
product length = 356
Forward primer  1       CTGAAGCTGGAGGACGGTAG  20
Template        460705  ....................  460724

Reverse primer  1       AGGCCTTCGGTAATGATCTTGT  22
Template        461060  ......................  461039
```

>CP107042.1 Pseudomonas aeruginosa strain GIMC5035:PA21/2013 chromosome

```
product length = 356
Forward primer  1        CTGAAGCTGGAGGACGGTAG  20
Template        6068009  ....................  6068028

Reverse primer  1        AGGCCTTCGGTAATGATCTTGT  22
Template        6068364  ......................  6068343
```

>CP086213.1 Pseudomonas aeruginosa strain Pa3 chromosome, complete genome

```
product length = 356
Forward primer  1       CTGAAGCTGGAGGACGGTAG  20
Template        591718  ....................  591737

Reverse primer  1       AGGCCTTCGGTAATGATCTTGT  22
Template        592073  ......................  592052
```

>CP104982.1 Pseudomonas aeruginosa PA14 isolate Gamma chromosome

```
product length = 356
Forward primer  1       CTGAAGCTGGAGGACGGTAG  20
Template        487465  ....................  487484

Reverse primer  1       AGGCCTTCGGTAATGATCTTGT  22
Template        487820  ......................  487799
```

>CP104984.1 Pseudomonas aeruginosa PA14 isolate Alpha chromosome

```
product length = 356
Forward primer  1       CTGAAGCTGGAGGACGGTAG  20
Template        487465  ....................  487484

Reverse primer  1       AGGCCTTCGGTAATGATCTTGT  22
Template        487820  ......................  487799
```

>CP104980.1 Pseudomonas aeruginosa PA14 isolate Epsilon chromosome

```
product length = 356
Forward primer  1       CTGAAGCTGGAGGACGGTAG  20
Template        487465  ....................  487484

Reverse primer  1       AGGCCTTCGGTAATGATCTTGT  22
Template        487820  ......................  487799
```

>CP104985.1 Pseudomonas aeruginosa PA14 chromosome

```
product length = 356
Forward primer  1       CTGAAGCTGGAGGACGGTAG  20
Template        487465  ....................  487484

Reverse primer  1       AGGCCTTCGGTAATGATCTTGT  22
Template        487820  ......................  487799
```

>CP104981.1 Pseudomonas aeruginosa PA14 isolate Delta chromosome

```
product length = 356
Forward primer  1       CTGAAGCTGGAGGACGGTAG  20
Template        487465  ....................  487484

Reverse primer  1       AGGCCTTCGGTAATGATCTTGT  22
Template        487820  ......................  487799
```

>CP104983.1 Pseudomonas aeruginosa PA14 isolate Beta chromosome

```
product length = 356
Forward primer  1       CTGAAGCTGGAGGACGGTAG  20
Template        487465  ....................  487484

Reverse primer  1       AGGCCTTCGGTAATGATCTTGT  22
Template        487820  ......................  487799
```

>CP104913.1 Pseudomonas aeruginosa strain PA-AUTBAM chromosome, complete genome

```
product length = 356
Forward primer  1       CTGAAGCTGGAGGACGGTAG  20
Template        472714  ....................  472733

Reverse primer  1       AGGCCTTCGGTAATGATCTTGT  22
Template        473069  ......................  473048
```

>CP104695.1 Pseudomonas aeruginosa strain 2021CK-01281 chromosome

```
product length = 356
Forward primer  1        CTGAAGCTGGAGGACGGTAG  20
Template        5168828  ....................  5168847

Reverse primer  1        AGGCCTTCGGTAATGATCTTGT  22
Template        5169183  ......................  5169162
```

>CP104720.1 Pseudomonas aeruginosa strain NY4593 chromosome, complete genome

```
product length = 356
Forward primer  1       CTGAAGCTGGAGGACGGTAG  20
Template        466891  ....................  466910

Reverse primer  1       AGGCCTTCGGTAATGATCTTGT  22
Template        467246  ......................  467225
```

>CP104590.1 Pseudomonas aeruginosa strain WTJH36 chromosome, complete genome

```
product length = 356
Forward primer  1        CTGAAGCTGGAGGACGGTAG  20
Template        4990076  ....................  4990095

Reverse primer  1        AGGCCTTCGGTAATGATCTTGT  22
Template        4990431  ......................  4990410
```

>CP104588.1 Pseudomonas aeruginosa strain WTJH32 chromosome, complete genome

```
product length = 356
Forward primer  1       CTGAAGCTGGAGGACGGTAG  20
Template        523727  ....................  523746

Reverse primer  1       AGGCCTTCGGTAATGATCTTGT  22
Template        524082  ......................  524061
```

>CP104584.1 Pseudomonas aeruginosa strain WTJH2 chromosome, complete genome

```
product length = 356
Forward primer  1       CTGAAGCTGGAGGACGGTAG  20
Template        523733  ....................  523752

Reverse primer  1       AGGCCTTCGGTAATGATCTTGT  22
Template        524088  ......................  524067
```

>CP104301.1 Pseudomonas aeruginosa strain PLL01 chromosome, complete genome

```
product length = 356
Forward primer  1       CTGAAGCTGGAGGACGGTAG  20
Template        472714  ....................  472733

Reverse primer  1       AGGCCTTCGGTAATGATCTTGT  22
Template        473069  ......................  473048
```

>CP104170.1 Pseudomonas aeruginosa strain HW001G chromosome, complete genome

```
product length = 356
Forward primer  1        CTGAAGCTGGAGGACGGTAG  20
Template        6434978  ....................  6434997

Reverse primer  1        AGGCCTTCGGTAATGATCTTGT  22
Template        6435333  ......................  6435312
```

>CP096207.1 Pseudomonas aeruginosa TBCF10839 chromosome, complete genome

```
product length = 356
Forward primer  1        CTGAAGCTGGAGGACGGTAG  20
Template        5046888  ....................  5046869

Reverse primer  1        AGGCCTTCGGTAATGATCTTGT  22
Template        5046533  ......................  5046554
```

>CP079712.1 Pseudomonas aeruginosa strain PAO1-UW chromosome, complete genome

```
product length = 356
Forward primer  1       CTGAAGCTGGAGGACGGTAG  20
Template        472092  ....................  472111

Reverse primer  1       AGGCCTTCGGTAATGATCTTGT  22
Template        472447  ......................  472426
```

>CP085082.1 Pseudomonas aeruginosa strain PAO1-Holloway chromosome, complete genome

```
product length = 356
Forward primer  1       CTGAAGCTGGAGGACGGTAG  20
Template        472493  ....................  472512

Reverse primer  1       AGGCCTTCGGTAATGATCTTGT  22
Template        472848  ......................  472827
```

>CP101885.1 Pseudomonas aeruginosa strain M27432 chromosome, complete genome

```
product length = 356
Forward primer  1       CTGAAGCTGGAGGACGGTAG  20
Template        473201  ....................  473220

Reverse primer  1       AGGCCTTCGGTAATGATCTTGT  22
Template        473556  ......................  473535
```

>CP094677.1 Pseudomonas aeruginosa strain Pa150 chromosome, complete genome

```
product length = 356
Forward primer  1        CTGAAGCTGGAGGACGGTAG  20
Template        2594442  ....................  2594423

Reverse primer  1        AGGCCTTCGGTAATGATCTTGT  22
Template        2594087  ......................  2594108
```

>CP103307.1 Pseudomonas aeruginosa strain PLL01 chromosome, complete genome

```
product length = 356
Forward primer  1       CTGAAGCTGGAGGACGGTAG  20
Template        472714  ....................  472733

Reverse primer  1       AGGCCTTCGGTAATGATCTTGT  22
Template        473069  ......................  473048
```

>CP102946.1 Pseudomonas aeruginosa strain SCAID WND1-2022 (148) chromosome, complete genome

```
product length = 356
Forward primer  1       CTGAAGCTGGAGGACGGTAG  20
Template        465739  ....................  465758

Reverse primer  1       AGGCCTTCGGTAATGATCTTGT  22
```

```
Template        466094  ......................  466073
```

>CP102944.1 Pseudomonas aeruginosa strain SCAID TCT1-2022 (325) chromosome, complete genome

```
product length = 356
Forward primer  1       CTGAAGCTGGAGGACGGTAG  20
Template        627741  ....................  627760

Reverse primer  1       AGGCCTTCGGTAATGATCTTGT  22
Template        628096  ......................  628075
```

>CP102174.1 Pseudomonas aeruginosa strain PA5083 chromosome, complete genome

```
product length = 356
Forward primer  1       CTGAAGCTGGAGGACGGTAG  20
Template        509461  ....................  509480

Reverse primer  1       AGGCCTTCGGTAATGATCTTGT  22
Template        509816  ......................  509795
```

>CP101656.1 Pseudomonas aeruginosa strain L1a chromosome, complete genome

```
product length = 356
Forward primer  1        CTGAAGCTGGAGGACGGTAG  20
Template        1473028  ....................  1473047

Reverse primer  1        AGGCCTTCGGTAATGATCTTGT  22
Template        1473383  ......................  1473362
```

>CP101912.1 Pseudomonas aeruginosa strain ATCC 27853 chromosome, complete genome

```
product length = 356
Forward primer  1       CTGAAGCTGGAGGACGGTAG  20
Template        482382  ....................  482401

Reverse primer  1       AGGCCTTCGGTAATGATCTTGT  22
Template        482737  ......................  482716
```

>CP101911.1 Pseudomonas aeruginosa strain NWRC-1223 chromosome, complete genome

```
product length = 356
Forward primer  1       CTGAAGCTGGAGGACGGTAG  20
Template        465614  ....................  465633

Reverse primer  1       AGGCCTTCGGTAATGATCTTGT  22
Template        465969  ......................  465948
```

>CP101540.1 Pseudomonas aeruginosa strain D-2 chromosome, complete genome

```
product length = 356
Forward primer  1       CTGAAGCTGGAGGACGGTAG  20
Template        455958  ....................  455977
```

```
Reverse primer  1       AGGCCTTCGGTAATGATCTTGT  22
Template        456313  ......................  456292
```

>CP094851.1 Pseudomonas aeruginosa strain R20-14 chromosome, complete genome

```
product length = 356
Forward primer  1       CTGAAGCTGGAGGACGGTAG  20
Template        471844  ....................  471863

Reverse primer  1       AGGCCTTCGGTAATGATCTTGT  22
Template        472199  ......................  472178
```

>CP100760.1 Pseudomonas aeruginosa strain AX0001 chromosome

```
product length = 356
Forward primer  1        CTGAAGCTGGAGGACGGTAG  20
Template        1483690  ....................  1483709

Reverse primer  1        AGGCCTTCGGTAATGATCTTGT  22
Template        1484045  ......................  1484024
```

>CP100761.1 Pseudomonas aeruginosa strain PA0011 chromosome

```
product length = 356
Forward primer  1        CTGAAGCTGGAGGACGGTAG  20
Template        1483690  ....................  1483709

Reverse primer  1        AGGCCTTCGGTAATGATCTTGT  22
Template        1484045  ......................  1484024
```

>CP097710.1 Pseudomonas aeruginosa strain PA-2 chromosome, complete genome

```
product length = 356
Forward primer  1       CTGAAGCTGGAGGACGGTAG  20
Template        488479  ....................  488498

Reverse primer  1       AGGCCTTCGGTAATGATCTTGT  22
Template        488834  ......................  488813
```

>CP097709.1 Pseudomonas aeruginosa strain PA-1 chromosome, complete genome

```
product length = 356
Forward primer  1       CTGAAGCTGGAGGACGGTAG  20
Template        488479  ....................  488498

Reverse primer  1       AGGCCTTCGGTAATGATCTTGT  22
Template        488834  ......................  488813
```

>CP100653.1 Pseudomonas aeruginosa strain F13 chromosome, complete genome

```
product length = 356
Forward primer  1       CTGAAGCTGGAGGACGGTAG  20
```

```
Template        466610  ....................  466629

Reverse primer  1       AGGCCTTCGGTAATGATCTTGT  22
Template        466965  ......................  466944
```

>CP099798.1 Pseudomonas aeruginosa strain PAO1-L chromosome, complete genome

```
product length = 356
Forward primer  1       CTGAAGCTGGAGGACGGTAG  20
Template        472712  ....................  472731

Reverse primer  1       AGGCCTTCGGTAATGATCTTGT  22
Template        473067  ......................  473046
```

>CP099797.1 Pseudomonas aeruginosa strain PAO1-N chromosome, complete genome

```
product length = 356
Forward primer  1       CTGAAGCTGGAGGACGGTAG  20
Template        472718  ....................  472737

Reverse primer  1       AGGCCTTCGGTAATGATCTTGT  22
Template        473073  ......................  473052
```

>CP096665.1 Pseudomonas aeruginosa strain PAO1_Mat-X-1 chromosome, complete genome

```
product length = 356
Forward primer  1       CTGAAGCTGGAGGACGGTAG  20
Template        472712  ....................  472731

Reverse primer  1       AGGCCTTCGGTAATGATCTTGT  22
Template        473067  ......................  473046
```

>CP096664.1 Pseudomonas aeruginosa strain PAO1_Kat-X-2 chromosome, complete genome

```
product length = 356
Forward primer  1       CTGAAGCTGGAGGACGGTAG  20
Template        472672  ....................  472691

Reverse primer  1       AGGCCTTCGGTAATGATCTTGT  22
Template        473027  ......................  473006
```

>CP091880.1 Pseudomonas aeruginosa strain US449 chromosome, complete genome

```
product length = 356
Forward primer  1       CTGAAGCTGGAGGACGGTAG  20
Template        469824  ....................  469843

Reverse primer  1       AGGCCTTCGGTAATGATCTTGT  22
Template        470179  ......................  470158
```

>CP069177.1 Pseudomonas aeruginosa strain Z154 chromosome, complete genome

```
product length = 356
```

```
Forward primer  1       CTGAAGCTGGAGGACGGTAG  20
Template        469442  ....................  469461

Reverse primer  1       AGGCCTTCGGTAATGATCTTGT  22
Template        469797  ......................  469776
```

>CP097575.1 Pseudomonas aeruginosa strain UNC_PaerCF25 chromosome, complete genome

```
product length = 356
Forward primer  1        CTGAAGCTGGAGGACGGTAG  20
Template        2607120  ....................  2607101

Reverse primer  1        AGGCCTTCGGTAATGATCTTGT  22
Template        2606765  ......................  2606786
```

>CP097383.1 Pseudomonas aeruginosa strain L00-a chromosome, complete genome

```
product length = 356
Forward primer  1       CTGAAGCTGGAGGACGGTAG  20
Template        480382  ....................  480401

Reverse primer  1       AGGCCTTCGGTAATGATCTTGT  22
Template        480737  ......................  480716
```

>CP097256.1 Pseudomonas aeruginosa strain D5 chromosome, complete genome

```
product length = 356
Forward primer  1       CTGAAGCTGGAGGACGGTAG  20
Template        464747  ....................  464766

Reverse primer  1       AGGCCTTCGGTAATGATCTTGT  22
Template        465102  ......................  465081
```

>CP096813.1 Pseudomonas aeruginosa strain 8D chromosome, complete genome

```
product length = 356
Forward primer  1        CTGAAGCTGGAGGACGGTAG  20
Template        1848324  ....................  1848343

Reverse primer  1        AGGCCTTCGGTAATGATCTTGT  22
Template        1848679  ......................  1848658
```

>CP095772.2 Pseudomonas aeruginosa strain 34Pae23 chromosome

```
product length = 356
Forward primer  1       CTGAAGCTGGAGGACGGTAG  20
Template        509707  ....................  509726

Reverse primer  1       AGGCCTTCGGTAATGATCTTGT  22
Template        510062  ......................  510041
```

>CP095923.1 Pseudomonas aeruginosa strain AR19438 chromosome, complete genome

```
product length = 356
Forward primer  1       CTGAAGCTGGAGGACGGTAG  20
Template        510738  ....................  510757

Reverse primer  1       AGGCCTTCGGTAATGATCTTGT  22
Template        511093  ......................  511072
```

>CP095920.1 Pseudomonas aeruginosa strain AR19640 chromosome, complete genome

```
product length = 356
Forward primer  1       CTGAAGCTGGAGGACGGTAG  20
Template        478777  ....................  478796

Reverse primer  1       AGGCCTTCGGTAATGATCTTGT  22
Template        479132  ......................  479111
```

>CP095922.1 Pseudomonas aeruginosa strain AR19583 chromosome, complete genome

```
product length = 356
Forward primer  1       CTGAAGCTGGAGGACGGTAG  20
Template        476149  ....................  476168

Reverse primer  1       AGGCCTTCGGTAATGATCTTGT  22
Template        476504  ......................  476483
```

>CP095770.1 Pseudomonas aeruginosa strain 34Pae36 chromosome, complete genome

```
product length = 356
Forward primer  1        CTGAAGCTGGAGGACGGTAG  20
Template        1661558  ....................  1661539

Reverse primer  1        AGGCCTTCGGTAATGATCTTGT  22
Template        1661203  ......................  1661224
```

>CP095774.1 Pseudomonas aeruginosa strain 34Pae8 chromosome, complete genome

```
product length = 356
Forward primer  1       CTGAAGCTGGAGGACGGTAG  20
Template        509715  ....................  509734

Reverse primer  1       AGGCCTTCGGTAATGATCTTGT  22
Template        510070  ......................  510049
```

>CP090649.1 Pseudomonas aeruginosa strain PA1609 chromosome, complete genome

```
product length = 356
Forward primer  1       CTGAAGCTGGAGGACGGTAG  20
Template        483546  ....................  483565

Reverse primer  1       AGGCCTTCGGTAATGATCTTGT  22
Template        483901  ......................  483880
```

>CP090648.1 Pseudomonas aeruginosa strain PA1616 chromosome, complete genome

```
product length = 356
Forward primer  1        CTGAAGCTGGAGGACGGTAG  20
Template        3009968  ....................  3009987

Reverse primer  1        AGGCCTTCGGTAATGATCTTGT  22
Template        3010323  ......................  3010302
```

>CP090647.1 Pseudomonas aeruginosa strain PA1681 chromosome, complete genome

```
product length = 356
Forward primer  1       CTGAAGCTGGAGGACGGTAG  20
Template        472174  ....................  472193

Reverse primer  1       AGGCCTTCGGTAATGATCTTGT  22
Template        472529  ......................  472508
```

>CP050149.1 Pseudomonas aeruginosa strain CHA chromosome

```
product length = 356
Forward primer  1       CTGAAGCTGGAGGACGGTAG  20
Template        466291  ....................  466310

Reverse primer  1       AGGCCTTCGGTAATGATCTTGT  22
Template        466646  ......................  466625
```

>CP050148.1 Pseudomonas aeruginosa strain AA43 chromosome, complete genome

```
product length = 356
Forward primer  1       CTGAAGCTGGAGGACGGTAG  20
Template        476205  ....................  476224

Reverse primer  1       AGGCCTTCGGTAATGATCTTGT  22
Template        476560  ......................  476539
```

>CP050147.1 Pseudomonas aeruginosa strain A5803 chromosome, complete genome

```
product length = 356
Forward primer  1       CTGAAGCTGGAGGACGGTAG  20
Template        468281  ....................  468300

Reverse primer  1       AGGCCTTCGGTAATGATCTTGT  22
Template        468636  ......................  468615
```

>CP064391.1 Pseudomonas aeruginosa strain PartH-Paeruginosa-RM8376 chromosome, complete genome

```
product length = 356
Forward primer  1        CTGAAGCTGGAGGACGGTAG  20
Template        5697341  ....................  5697360

Reverse primer  1        AGGCCTTCGGTAATGATCTTGT  22
Template        5697696  ......................  5697675
```

>CP063387.1 Pseudomonas aeruginosa strain ST1076_d100blood2 chromosome, complete genome

```
product length = 356
Forward primer  1       CTGAAGCTGGAGGACGGTAG  20
Template        472594  ....................  472613

Reverse primer  1       AGGCCTTCGGTAATGATCTTGT  22
Template        472949  ......................  472928
```

>CP047643.1 Pseudomonas aeruginosa CI27 chromosome, complete genome

```
product length = 356
Forward primer  1       CTGAAGCTGGAGGACGGTAG  20
Template        486977  ....................  486996

Reverse primer  1       AGGCCTTCGGTAATGATCTTGT  22
Template        487332  ......................  487311
```

>CP063396.1 Pseudomonas aeruginosa strain ST167_d26burn chromosome, complete genome

```
product length = 356
Forward primer  1       CTGAAGCTGGAGGACGGTAG  20
Template        516452  ....................  516471

Reverse primer  1       AGGCCTTCGGTAATGATCTTGT  22
Template        516807  ......................  516786
```

>CP063395.1 Pseudomonas aeruginosa strain ST167_d57blood chromosome, complete genome

```
product length = 356
Forward primer  1       CTGAAGCTGGAGGACGGTAG  20
Template        516452  ....................  516471

Reverse primer  1       AGGCCTTCGGTAATGATCTTGT  22
Template        516807  ......................  516786
```

>CP063394.1 Pseudomonas aeruginosa strain ST167_d67burn1 chromosome, complete genome

```
product length = 356
Forward primer  1       CTGAAGCTGGAGGACGGTAG  20
Template        516923  ....................  516942

Reverse primer  1       AGGCCTTCGGTAATGATCTTGT  22
Template        517278  ......................  517257
```

>CP063393.1 Pseudomonas aeruginosa strain ST167_d67burn2 chromosome, complete genome

```
product length = 356
Forward primer  1       CTGAAGCTGGAGGACGGTAG  20
Template        516452  ....................  516471

Reverse primer  1       AGGCCTTCGGTAATGATCTTGT  22
Template        516807  ......................  516786
```

>CP063392.1 Pseudomonas aeruginosa strain ST167_d68blood1 chromosome, complete genome

```
product length = 356
Forward primer  1       CTGAAGCTGGAGGACGGTAG  20
Template        516452  ....................  516471

Reverse primer  1       AGGCCTTCGGTAATGATCTTGT  22
Template        516807  ......................  516786
```

>CP063391.1 Pseudomonas aeruginosa strain ST167_d68blood2 chromosome, complete genome

```
product length = 356
Forward primer  1       CTGAAGCTGGAGGACGGTAG  20
Template        516452  ....................  516471

Reverse primer  1       AGGCCTTCGGTAATGATCTTGT  22
Template        516807  ......................  516786
```

>CP063390.1 Pseudomonas aeruginosa strain ST1076_d97burn1 chromosome, complete genome

```
product length = 356
Forward primer  1       CTGAAGCTGGAGGACGGTAG  20
Template        472594  ....................  472613

Reverse primer  1       AGGCCTTCGGTAATGATCTTGT  22
Template        472949  ......................  472928
```

>CP063389.1 Pseudomonas aeruginosa strain ST1076_d97burn2 chromosome, complete genome

```
product length = 356
Forward primer  1       CTGAAGCTGGAGGACGGTAG  20
Template        472594  ....................  472613

Reverse primer  1       AGGCCTTCGGTAATGATCTTGT  22
Template        472949  ......................  472928
```

>CP063388.1 Pseudomonas aeruginosa strain ST1076_d100blood1 chromosome, complete genome

```
product length = 356
Forward primer  1       CTGAAGCTGGAGGACGGTAG  20
Template        472594  ....................  472613

Reverse primer  1       AGGCCTTCGGTAATGATCTTGT  22
Template        472949  ......................  472928
```

>CP063386.1 Pseudomonas aeruginosa strain ST1076_d118limb1 chromosome, complete genome

```
product length = 356
Forward primer  1       CTGAAGCTGGAGGACGGTAG  20
Template        472595  ....................  472614

Reverse primer  1       AGGCCTTCGGTAATGATCTTGT  22
Template        472950  ......................  472929
```

>CP063385.1 Pseudomonas aeruginosa strain St1076_d123blood chromosome, complete genome

```
product length = 356
Forward primer  1       CTGAAGCTGGAGGACGGTAG  20
Template        472594  ....................  472613

Reverse primer  1       AGGCCTTCGGTAATGATCTTGT  22
Template        472949  ......................  472928
```

>CP093967.1 Pseudomonas aeruginosa strain NY4605 chromosome, complete genome

```
product length = 356
Forward primer  1       CTGAAGCTGGAGGACGGTAG  20
Template        459224  ....................  459243

Reverse primer  1       AGGCCTTCGGTAATGATCTTGT  22
Template        459579  ......................  459558
```

>CP093965.1 Pseudomonas aeruginosa strain ATCC BAA-2108 chromosome, complete genome

```
product length = 356
Forward primer  1       CTGAAGCTGGAGGACGGTAG  20
Template        478703  ....................  478722

Reverse primer  1       AGGCCTTCGGTAATGATCTTGT  22
Template        479058  ......................  479037
```

>CP093966.1 Pseudomonas aeruginosa strain ATCC BAA-2114 chromosome, complete genome

```
product length = 356
Forward primer  1       CTGAAGCTGGAGGACGGTAG  20
Template        466363  ....................  466382

Reverse primer  1       AGGCCTTCGGTAATGATCTTGT  22
Template        466718  ......................  466697
```

>CP093395.1 Pseudomonas aeruginosa strain PA1_NCHU chromosome, complete genome

```
product length = 356
Forward primer  1        CTGAAGCTGGAGGACGGTAG  20
Template        4139799  ....................  4139780

Reverse primer  1        AGGCCTTCGGTAATGATCTTGT  22
Template        4139444  ......................  4139465
```

>CP093358.1 Pseudomonas aeruginosa strain E167 chromosome, complete genome

```
product length = 356
Forward primer  1       CTGAAGCTGGAGGACGGTAG  20
Template        471424  ....................  471443

Reverse primer  1       AGGCCTTCGGTAATGATCTTGT  22
Template        471779  ......................  471758
```

>CP093356.1 Pseudomonas aeruginosa strain E125 chromosome, complete genome

```
product length = 356
Forward primer  1       CTGAAGCTGGAGGACGGTAG  20
Template        517988  ....................  518007

Reverse primer  1       AGGCCTTCGGTAATGATCTTGT  22
Template        518343  ......................  518322
```

>CP093357.1 Pseudomonas aeruginosa strain E131 chromosome, complete genome

```
product length = 356
Forward primer  1       CTGAAGCTGGAGGACGGTAG  20
Template        471453  ....................  471472

Reverse primer  1       AGGCCTTCGGTAATGATCTTGT  22
Template        471808  ......................  471787
```

>CP093355.1 Pseudomonas aeruginosa strain E104 chromosome, complete genome

```
product length = 356
Forward primer  1       CTGAAGCTGGAGGACGGTAG  20
Template        471473  ....................  471492

Reverse primer  1       AGGCCTTCGGTAATGATCTTGT  22
Template        471828  ......................  471807
```

>CP093354.1 Pseudomonas aeruginosa strain E113 chromosome, complete genome

```
product length = 356
Forward primer  1       CTGAAGCTGGAGGACGGTAG  20
Template        504552  ....................  504571

Reverse primer  1       AGGCCTTCGGTAATGATCTTGT  22
Template        504907  ......................  504886
```

>CP093028.1 Pseudomonas aeruginosa strain H05 chromosome, complete genome

```
product length = 356
Forward primer  1       CTGAAGCTGGAGGACGGTAG  20
Template        463379  ....................  463398

Reverse primer  1       AGGCCTTCGGTAATGATCTTGT  22
Template        463734  ......................  463713
```

>CP093030.1 Pseudomonas aeruginosa strain H04 chromosome, complete genome

```
product length = 356
Forward primer  1       CTGAAGCTGGAGGACGGTAG  20
Template        465003  ....................  465022

Reverse primer  1       AGGCCTTCGGTAATGATCTTGT  22
Template        465358  ......................  465337
```

>CP093032.1 Pseudomonas aeruginosa strain H02 chromosome, complete genome

```
product length = 356
Forward primer  1       CTGAAGCTGGAGGACGGTAG  20
Template        487007  ....................  487026

Reverse primer  1       AGGCCTTCGGTAATGATCTTGT  22
Template        487362  ......................  487341
```

>CP093031.1 Pseudomonas aeruginosa strain H03 chromosome, complete genome

```
product length = 356
Forward primer  1       CTGAAGCTGGAGGACGGTAG  20
Template        517479  ....................  517498

Reverse primer  1       AGGCCTTCGGTAATGATCTTGT  22
Template        517834  ......................  517813
```

>CP093024.1 Pseudomonas aeruginosa strain H06 chromosome

```
product length = 356
Forward primer  1        CTGAAGCTGGAGGACGGTAG  20
Template        3021012  ....................  3020993

Reverse primer  1        AGGCCTTCGGTAATGATCTTGT  22
Template        3020657  ......................  3020678
```

>CP093013.1 Pseudomonas aeruginosa strain H19 chromosome

```
product length = 356
Forward primer  1        CTGAAGCTGGAGGACGGTAG  20
Template        2303013  ....................  2302994

Reverse primer  1        AGGCCTTCGGTAATGATCTTGT  22
Template        2302658  ......................  2302679
```

>CP093022.1 Pseudomonas aeruginosa strain H08 chromosome, complete genome

```
product length = 356
Forward primer  1       CTGAAGCTGGAGGACGGTAG  20
Template        468283  ....................  468302

Reverse primer  1       AGGCCTTCGGTAATGATCTTGT  22
Template        468638  ......................  468617
```

>CP093023.1 Pseudomonas aeruginosa strain H07 chromosome, complete genome

```
product length = 356
Forward primer  1       CTGAAGCTGGAGGACGGTAG  20
Template        468950  ....................  468969

Reverse primer  1       AGGCCTTCGGTAATGATCTTGT  22
Template        469305  ......................  469284
```

>CP093018.1 Pseudomonas aeruginosa strain H11 chromosome

```
product length = 356
Forward primer  1        CTGAAGCTGGAGGACGGTAG  20
Template        3330950  ....................  3330969

Reverse primer  1        AGGCCTTCGGTAATGATCTTGT  22
Template        3331305  ......................  3331284
```

>CP093016.1 Pseudomonas aeruginosa strain H15 chromosome, complete genome

```
product length = 356
Forward primer  1        CTGAAGCTGGAGGACGGTAG  20
Template        1095586  ....................  1095567

Reverse primer  1        AGGCCTTCGGTAATGATCTTGT  22
Template        1095231  ......................  1095252
```

>CP093015.1 Pseudomonas aeruginosa strain H16 chromosome, complete genome

```
product length = 356
Forward primer  1       CTGAAGCTGGAGGACGGTAG  20
Template        494775  ....................  494794

Reverse primer  1       AGGCCTTCGGTAATGATCTTGT  22
Template        495130  ......................  495109
```

>CP093020.1 Pseudomonas aeruginosa strain H10 chromosome, complete genome

```
product length = 356
Forward primer  1       CTGAAGCTGGAGGACGGTAG  20
Template        471443  ....................  471462

Reverse primer  1       AGGCCTTCGGTAATGATCTTGT  22
Template        471798  ......................  471777
```

>CP093012.1 Pseudomonas aeruginosa strain H20 chromosome, complete genome

```
product length = 356
Forward primer  1       CTGAAGCTGGAGGACGGTAG  20
Template        461138  ....................  461157

Reverse primer  1       AGGCCTTCGGTAATGATCTTGT  22
Template        461493  ......................  461472
```

>CP093014.1 Pseudomonas aeruginosa strain H17 chromosome, complete genome

```
product length = 356
Forward primer  1       CTGAAGCTGGAGGACGGTAG  20
Template        485059  ....................  485078

Reverse primer  1       AGGCCTTCGGTAATGATCTTGT  22
Template        485414  ......................  485393
```

>CP080405.1 Pseudomonas aeruginosa strain PES_P749 chromosome, complete genome

```
product length = 356
Forward primer  1       CTGAAGCTGGAGGACGGTAG  20
Template        471216  ....................  471235

Reverse primer  1       AGGCCTTCGGTAATGATCTTGT  22
Template        471571  ......................  471550
```

>CP081148.1 Pseudomonas aeruginosa strain NDM1_2 chromosome

```
product length = 356
Forward primer  1       CTGAAGCTGGAGGACGGTAG  20
Template        480166  ....................  480185

Reverse primer  1       AGGCCTTCGGTAATGATCTTGT  22
Template        480521  ......................  480500
```

>CP092634.1 Pseudomonas aeruginosa strain LS.2c chromosome, complete genome

```
product length = 356
Forward primer  1       CTGAAGCTGGAGGACGGTAG  20
Template        464483  ....................  464502

Reverse primer  1       AGGCCTTCGGTAATGATCTTGT  22
Template        464838  ......................  464817
```

>CP092629.1 Pseudomonas aeruginosa strain HU20 chromosome, complete genome

```
product length = 356
Forward primer  1        CTGAAGCTGGAGGACGGTAG  20
Template        1021610  ....................  1021591

Reverse primer  1        AGGCCTTCGGTAATGATCTTGT  22
Template        1021255  ......................  1021276
```

>CP092032.1 Pseudomonas aeruginosa strain ZS-PA-05 chromosome, complete genome

```
product length = 356
Forward primer  1       CTGAAGCTGGAGGACGGTAG  20
Template        472743  ....................  472762

Reverse primer  1       AGGCCTTCGGTAATGATCTTGT  22
Template        473098  ......................  473077
```

>CP054794.1 Pseudomonas aeruginosa strain A0002 chromosome, complete genome

```
product length = 356
Forward primer  1       CTGAAGCTGGAGGACGGTAG  20
Template        458368  ....................  458387

Reverse primer  1       AGGCCTTCGGTAATGATCTTGT  22
Template        458723  ......................  458702
```

>CP054793.1 Pseudomonas aeruginosa strain SE5452 chromosome, complete genome

```
product length = 356
Forward primer  1       CTGAAGCTGGAGGACGGTAG  20
Template        476445  ....................  476464

Reverse primer  1       AGGCCTTCGGTAATGATCTTGT  22
Template        476800  ......................  476779
```

>CP054792.1 Pseudomonas aeruginosa strain SE5431 chromosome, complete genome

```
product length = 356
Forward primer  1       CTGAAGCTGGAGGACGGTAG  20
Template        470942  ....................  470961

Reverse primer  1       AGGCCTTCGGTAATGATCTTGT  22
Template        471297  ......................  471276
```

>CP054791.1 Pseudomonas aeruginosa strain SE5430 chromosome, complete genome

```
product length = 356
Forward primer  1       CTGAAGCTGGAGGACGGTAG  20
Template        508308  ....................  508327

Reverse primer  1       AGGCCTTCGGTAATGATCTTGT  22
Template        508663  ......................  508642
```

>CP054790.1 Pseudomonas aeruginosa strain SE5418 chromosome, complete genome

```
product length = 356
Forward primer  1       CTGAAGCTGGAGGACGGTAG  20
Template        504122  ....................  504141

Reverse primer  1       AGGCCTTCGGTAATGATCTTGT  22
Template        504477  ......................  504456
```

>CP054789.1 Pseudomonas aeruginosa strain SE5381 chromosome, complete genome

```
product length = 356
Forward primer  1       CTGAAGCTGGAGGACGGTAG  20
Template        504117  ....................  504136

Reverse primer  1       AGGCCTTCGGTAATGATCTTGT  22
Template        504472  ......................  504451
```

>CP054788.1 Pseudomonas aeruginosa strain YTSY4 chromosome, complete genome

```
product length = 356
Forward primer  1        CTGAAGCTGGAGGACGGTAG  20
Template        3431427  ....................  3431408

Reverse primer  1        AGGCCTTCGGTAATGATCTTGT  22
Template        3431072  ......................  3431093
```

>CP054787.1 Pseudomonas aeruginosa strain HB2011305RE chromosome, complete genome

```
product length = 356
Forward primer  1       CTGAAGCTGGAGGACGGTAG  20
Template        519799  ....................  519818

Reverse primer  1       AGGCCTTCGGTAATGATCTTGT  22
Template        520154  ......................  520133
```

>CP054786.1 Pseudomonas aeruginosa strain DL201330 chromosome, complete genome

```
product length = 356
Forward primer  1        CTGAAGCTGGAGGACGGTAG  20
Template        4951574  ....................  4951593

Reverse primer  1        AGGCCTTCGGTAATGATCTTGT  22
Template        4951929  ......................  4951908
```

>CP086122.1 Pseudomonas aeruginosa strain MIN-155 chromosome, complete genome

```
product length = 356
Forward primer  1       CTGAAGCTGGAGGACGGTAG  20
Template        472177  ....................  472196

Reverse primer  1       AGGCCTTCGGTAATGATCTTGT  22
Template        472532  ......................  472511
```

>CP081477.2 Pseudomonas aeruginosa strain P8W chromosome, complete genome

```
product length = 356
Forward primer  1        CTGAAGCTGGAGGACGGTAG  20
Template        6733508  ....................  6733527

Reverse primer  1        AGGCCTTCGGTAATGATCTTGT  22
Template        6733863  ......................  6733842
```

>CP090348.1 Pseudomonas aeruginosa strain PA8329 chromosome, complete genome

```
product length = 356
Forward primer  1       CTGAAGCTGGAGGACGGTAG  20
Template        466036  ....................  466055

Reverse primer  1       AGGCCTTCGGTAATGATCTTGT  22
Template        466391  ......................  466370
```

>CP053747.1 Pseudomonas aeruginosa strain Pae1255-NDM1 chromosome, complete genome

```
product length = 356
Forward primer  1       CTGAAGCTGGAGGACGGTAG  20
Template        562169  ....................  562188

Reverse primer  1       AGGCCTTCGGTAATGATCTTGT  22
Template        562524  ......................  562503
```

>CP089063.2 Pseudomonas aeruginosa strain UNC_PaerCF37 chromosome, complete genome

```
product length = 356
Forward primer  1        CTGAAGCTGGAGGACGGTAG  20
Template        6260773  ....................  6260754

Reverse primer  1        AGGCCTTCGGTAATGATCTTGT  22
Template        6260418  ......................  6260439
```

>CP089064.2 Pseudomonas aeruginosa strain UNC_PaerCF35 chromosome, complete genome

```
product length = 356
Forward primer  1        CTGAAGCTGGAGGACGGTAG  20
Template        3293218  ....................  3293237

Reverse primer  1        AGGCCTTCGGTAATGATCTTGT  22
Template        3293573  ......................  3293552
```

>CP089062.2 Pseudomonas aeruginosa strain UNC_PaerCF38 chromosome, complete genome

```
product length = 356
Forward primer  1        CTGAAGCTGGAGGACGGTAG  20
Template        1696990  ....................  1697009

Reverse primer  1        AGGCCTTCGGTAATGATCTTGT  22
Template        1697345  ......................  1697324
```

>CP089061.2 Pseudomonas aeruginosa strain UNC_PaerCF41 chromosome, complete genome

```
product length = 356
Forward primer  1        CTGAAGCTGGAGGACGGTAG  20
Template        3368309  ....................  3368328

Reverse primer  1        AGGCCTTCGGTAATGATCTTGT  22
Template        3368664  ......................  3368643
```

>CP089849.1 Pseudomonas aeruginosa strain PA0523 chromosome, complete genome

```
product length = 356
Forward primer  1       CTGAAGCTGGAGGACGGTAG  20
Template        511492  ....................  511511

Reverse primer  1       AGGCCTTCGGTAATGATCTTGT  22
Template        511847  ......................  511826
```

>CP089745.1 Pseudomonas aeruginosa strain Pa608 chromosome, complete genome

```
product length = 356
Forward primer  1       CTGAAGCTGGAGGACGGTAG  20
Template        461683  ....................  461702

Reverse primer  1       AGGCCTTCGGTAATGATCTTGT  22
Template        462038  ......................  462017
```

>CP089238.1 Pseudomonas aeruginosa strain JNQH-PA033 chromosome, complete genome

```
product length = 356
Forward primer  1       CTGAAGCTGGAGGACGGTAG  20
Template        486803  ....................  486822

Reverse primer  1       AGGCCTTCGGTAATGATCTTGT  22
Template        487158  ......................  487137
```

>CP089236.1 Pseudomonas aeruginosa strain JNQH-PA027 chromosome, complete genome

```
product length = 356
Forward primer  1       CTGAAGCTGGAGGACGGTAG  20
Template        524896  ....................  524915

Reverse primer  1       AGGCCTTCGGTAATGATCTTGT  22
Template        525251  ......................  525230
```

>CP087675.1 Pseudomonas aeruginosa strain P93127 chromosome, complete genome

```
product length = 356
Forward primer  1       CTGAAGCTGGAGGACGGTAG  20
Template        488484  ....................  488503

Reverse primer  1       AGGCCTTCGGTAATGATCTTGT  22
Template        488839  ......................  488818
```

>CP087674.1 Pseudomonas aeruginosa strain P4970C chromosome, complete genome

```
product length = 356
Forward primer  1       CTGAAGCTGGAGGACGGTAG  20
Template        488478  ....................  488497

Reverse primer  1       AGGCCTTCGGTAATGATCTTGT  22
Template        488833  ......................  488812
```

>CP087673.1 Pseudomonas aeruginosa strain P96131 chromosome, complete genome

```
product length = 356
Forward primer  1       CTGAAGCTGGAGGACGGTAG  20
Template        475004  ....................  475023

Reverse primer  1       AGGCCTTCGGTAATGATCTTGT  22
Template        475359  ......................  475338
```

>CP080369.1 Pseudomonas aeruginosa SG17M chromosome, complete genome

```
product length = 356
Forward primer  1       CTGAAGCTGGAGGACGGTAG  20
Template        743178  ....................  743197

Reverse primer  1       AGGCCTTCGGTAATGATCTTGT  22
Template        743533  ......................  743512
```

>CP086016.1 Pseudomonas aeruginosa isolate KB-PA_3 chromosome, complete genome

```
product length = 356
Forward primer  1       CTGAAGCTGGAGGACGGTAG  20
Template        499764  ....................  499783

Reverse primer  1       AGGCCTTCGGTAATGATCTTGT  22
Template        500119  ......................  500098
```

>CP086010.1 Pseudomonas aeruginosa isolate KB-PA_F19 chromosome, complete genome

```
product length = 356
Forward primer  1       CTGAAGCTGGAGGACGGTAG  20
Template        470467  ....................  470486

Reverse primer  1       AGGCCTTCGGTAATGATCTTGT  22
Template        470822  ......................  470801
```

>CP084484.1 Pseudomonas sp. PS1(2021) chromosome, complete genome

```
product length = 356
Forward primer  1        CTGAAGCTGGAGGACGGTAG  20
Template        6591624  ....................  6591643

Reverse primer  1        AGGCCTTCGGTAATGATCTTGT  22
Template        6591979  ......................  6591958
```

>CP083366.1 Pseudomonas aeruginosa strain PS1793 chromosome, complete genome

```
product length = 356
Forward primer  1       CTGAAGCTGGAGGACGGTAG  20
Template        464604  ....................  464623

Reverse primer  1       AGGCCTTCGGTAATGATCTTGT  22
Template        464959  ......................  464938
```

>CP082821.1 Pseudomonas aeruginosa strain SCAID PLC1-2021 (16/222) chromosome, complete genome

```
product length = 356
Forward primer  1       CTGAAGCTGGAGGACGGTAG  20
Template        479280  ....................  479299

Reverse primer  1       AGGCCTTCGGTAATGATCTTGT  22
Template        479635  ......................  479614
```

>CP082822.1 Pseudomonas aeruginosa strain SCAID WND1-2021 (9/195) chromosome, complete genome

```
product length = 356
Forward primer  1       CTGAAGCTGGAGGACGGTAG  20
Template        497392  ....................  497411
```

```
Reverse primer  1       AGGCCTTCGGTAATGATCTTGT  22
Template        497747  ......................  497726
```

>CP082823.1 Pseudomonas aeruginosa strain SCAID TST-2021 (7/157) chromosome, complete genome

```
product length = 356
Forward primer  1       CTGAAGCTGGAGGACGGTAG  20
Template        479277  ....................  479296

Reverse primer  1       AGGCCTTCGGTAATGATCTTGT  22
Template        479632  ......................  479611
```

>CP078009.1 Pseudomonas aeruginosa strain FAHZU31 chromosome, complete genome

```
product length = 356
Forward primer  1       CTGAAGCTGGAGGACGGTAG  20
Template        467635  ....................  467654

Reverse primer  1       AGGCCTTCGGTAATGATCTTGT  22
Template        467990  ......................  467969
```

>CP078007.1 Pseudomonas aeruginosa strain FAHZU40 chromosome, complete genome

```
product length = 356
Forward primer  1       CTGAAGCTGGAGGACGGTAG  20
Template        480181  ....................  480200

Reverse primer  1       AGGCCTTCGGTAATGATCTTGT  22
Template        480536  ......................  480515
```

>CP078006.1 Pseudomonas aeruginosa strain NDTH7329 chromosome, complete genome

```
product length = 356
Forward primer  1       CTGAAGCTGGAGGACGGTAG  20
Template        504528  ....................  504547

Reverse primer  1       AGGCCTTCGGTAATGATCTTGT  22
Template        504883  ......................  504862
```

>CP078004.1 Pseudomonas aeruginosa strain QZPH16 chromosome, complete genome

```
product length = 356
Forward primer  1       CTGAAGCTGGAGGACGGTAG  20
Template        504447  ....................  504466

Reverse primer  1       AGGCCTTCGGTAATGATCTTGT  22
Template        504802  ......................  504781
```

>CP078002.1 Pseudomonas aeruginosa strain QZPH21 chromosome, complete genome

```
product length = 356
Forward primer  1       CTGAAGCTGGAGGACGGTAG  20
```

```
Template        504447  ....................  504466

Reverse primer  1       AGGCCTTCGGTAATGATCTTGT  22
Template        504802  ......................  504781
```

>CP077999.1 Pseudomonas aeruginosa strain SRRSH1120 chromosome, complete genome

```
product length = 356
Forward primer  1       CTGAAGCTGGAGGACGGTAG  20
Template        504528  ....................  504547

Reverse primer  1       AGGCCTTCGGTAATGATCTTGT  22
Template        504883  ......................  504862
```

>CP077997.1 Pseudomonas aeruginosa strain SRRSH1521 chromosome, complete genome

```
product length = 356
Forward primer  1       CTGAAGCTGGAGGACGGTAG  20
Template        467638  ....................  467657

Reverse primer  1       AGGCCTTCGGTAATGATCTTGT  22
Template        467993  ......................  467972
```

>CP077994.1 Pseudomonas aeruginosa strain SRRSH2790 chromosome, complete genome

```
product length = 356
Forward primer  1       CTGAAGCTGGAGGACGGTAG  20
Template        504453  ....................  504472

Reverse primer  1       AGGCCTTCGGTAATGATCTTGT  22
Template        504808  ......................  504787
```

>CP077988.1 Pseudomonas aeruginosa strain ZPPH1 chromosome, complete genome

```
product length = 356
Forward primer  1       CTGAAGCTGGAGGACGGTAG  20
Template        514647  ....................  514666

Reverse primer  1       AGGCCTTCGGTAATGATCTTGT  22
Template        515002  ......................  514981
```

>CP077985.1 Pseudomonas aeruginosa strain ZPPH2 chromosome, complete genome

```
product length = 356
Forward primer  1       CTGAAGCTGGAGGACGGTAG  20
Template        514653  ....................  514672

Reverse primer  1       AGGCCTTCGGTAATGATCTTGT  22
Template        515008  ......................  514987
```

>CP077981.1 Pseudomonas aeruginosa strain ZPPH14 chromosome, complete genome

```
product length = 356
```

```
Forward primer  1       CTGAAGCTGGAGGACGGTAG  20
Template        473480  ....................  473499

Reverse primer  1       AGGCCTTCGGTAATGATCTTGT  22
Template        473835  ......................  473814
```

>CP077977.1 Pseudomonas aeruginosa strain ZPPH29 chromosome, complete genome

```
product length = 356
Forward primer  1       CTGAAGCTGGAGGACGGTAG  20
Template        468882  ....................  468901

Reverse primer  1       AGGCCTTCGGTAATGATCTTGT  22
Template        469237  ......................  469216
```

>CP077971.1 Pseudomonas aeruginosa strain ZPPH33 chromosome, complete genome

```
product length = 356
Forward primer  1       CTGAAGCTGGAGGACGGTAG  20
Template        514647  ....................  514666

Reverse primer  1       AGGCCTTCGGTAATGATCTTGT  22
Template        515002  ......................  514981
```

>CP064403.1 Pseudomonas aeruginosa strain WTJH12 chromosome, complete genome

```
product length = 356
Forward primer  1       CTGAAGCTGGAGGACGGTAG  20
Template        472282  ....................  472301

Reverse primer  1       AGGCCTTCGGTAATGATCTTGT  22
Template        472637  ......................  472616
```

>CP064401.1 Pseudomonas aeruginosa strain NDTH10366 chromosome, complete genome

```
product length = 356
Forward primer  1       CTGAAGCTGGAGGACGGTAG  20
Template        508258  ....................  508277

Reverse primer  1       AGGCCTTCGGTAATGATCTTGT  22
Template        508613  ......................  508592
```

>CP064399.1 Pseudomonas aeruginosa strain QZPH41 chromosome, complete genome

```
product length = 356
Forward primer  1       CTGAAGCTGGAGGACGGTAG  20
Template        456587  ....................  456606

Reverse primer  1       AGGCCTTCGGTAATGATCTTGT  22
Template        456942  ......................  456921
```

>CP064397.1 Pseudomonas aeruginosa strain SRRSH1002 chromosome, complete genome

```
product length = 356
Forward primer  1       CTGAAGCTGGAGGACGGTAG  20
Template        505269  ....................  505288

Reverse primer  1       AGGCCTTCGGTAATGATCTTGT  22
Template        505624  ......................  505603
```

>CP064395.1 Pseudomonas aeruginosa strain SRRSH1408 chromosome, complete genome

```
product length = 356
Forward primer  1       CTGAAGCTGGAGGACGGTAG  20
Template        504430  ....................  504449

Reverse primer  1       AGGCCTTCGGTAATGATCTTGT  22
Template        504785  ......................  504764
```

>CP064393.1 Pseudomonas aeruginosa strain SRRSH1101 chromosome, complete genome

```
product length = 356
Forward primer  1       CTGAAGCTGGAGGACGGTAG  20
Template        504441  ....................  504460

Reverse primer  1       AGGCCTTCGGTAATGATCTTGT  22
Template        504796  ......................  504775
```

>CP064392.1 Pseudomonas aeruginosa strain SRRSH15 chromosome, complete genome

```
product length = 356
Forward primer  1       CTGAAGCTGGAGGACGGTAG  20
Template        467644  ....................  467663

Reverse primer  1       AGGCCTTCGGTAATGATCTTGT  22
Template        467999  ......................  467978
```

>CP080518.1 Pseudomonas aeruginosa strain YY322 chromosome, complete genome

```
product length = 356
Forward primer  1       CTGAAGCTGGAGGACGGTAG  20
Template        461569  ....................  461588

Reverse primer  1       AGGCCTTCGGTAATGATCTTGT  22
Template        461924  ......................  461903
```

>CP080511.1 Pseudomonas aeruginosa strain DJ06 chromosome, complete genome

```
product length = 356
Forward primer  1       CTGAAGCTGGAGGACGGTAG  20
Template        146747  ....................  146728

Reverse primer  1       AGGCCTTCGGTAATGATCTTGT  22
Template        146392  ......................  146413
```

>CP071947.1 Pseudomonas aeruginosa strain 2020HL-00861 chromosome, complete genome

```
product length = 356
Forward primer  1        CTGAAGCTGGAGGACGGTAG  20
Template        1975546  ....................  1975565

Reverse primer  1        AGGCCTTCGGTAATGATCTTGT  22
Template        1975901  ......................  1975880
```

>CP080289.1 Pseudomonas aeruginosa strain PA2207 chromosome, complete genome

```
product length = 356
Forward primer  1       CTGAAGCTGGAGGACGGTAG  20
Template        504441  ....................  504460

Reverse primer  1       AGGCCTTCGGTAATGATCTTGT  22
Template        504796  ......................  504775
```

>CP080287.1 Pseudomonas aeruginosa strain UNC_PaerCF11 chromosome, complete genome

```
product length = 356
Forward primer  1        CTGAAGCTGGAGGACGGTAG  20
Template        5069400  ....................  5069419

Reverse primer  1        AGGCCTTCGGTAATGATCTTGT  22
Template        5069755  ......................  5069734
```

>CP080288.1 Pseudomonas aeruginosa strain UNC_PaerCF05 chromosome, complete genome

```
product length = 356
Forward primer  1       CTGAAGCTGGAGGACGGTAG  20
Template        237967  ....................  237948

Reverse primer  1       AGGCCTTCGGTAATGATCTTGT  22
Template        237612  ......................  237633
```

>CP080282.1 Pseudomonas aeruginosa strain UNC_PaerCF16 chromosome, complete genome

```
product length = 356
Forward primer  1        CTGAAGCTGGAGGACGGTAG  20
Template        1967219  ....................  1967238

Reverse primer  1        AGGCCTTCGGTAATGATCTTGT  22
Template        1967574  ......................  1967553
```

>CP080285.1 Pseudomonas aeruginosa strain UNC_PaerCF14 chromosome, complete genome

```
product length = 356
Forward primer  1       CTGAAGCTGGAGGACGGTAG  20
Template        271435  ....................  271416

Reverse primer  1       AGGCCTTCGGTAATGATCTTGT  22
Template        271080  ......................  271101
```

>CP080280.1 Pseudomonas aeruginosa strain UNC_PaerCF20 chromosome, complete genome

```
product length = 356
Forward primer  1        CTGAAGCTGGAGGACGGTAG  20
Template        6042247  ....................  6042228

Reverse primer  1        AGGCCTTCGGTAATGATCTTGT  22
Template        6041892  ......................  6041913
```

>CP080281.1 Pseudomonas aeruginosa strain UNC_PaerCF17 chromosome, complete genome

```
product length = 356
Forward primer  1        CTGAAGCTGGAGGACGGTAG  20
Template        1946505  ....................  1946524

Reverse primer  1        AGGCCTTCGGTAATGATCTTGT  22
Template        1946860  ......................  1946839
```

>CP080011.1 Pseudomonas aeruginosa strain TL3773 chromosome, complete genome

```
product length = 356
Forward primer  1        CTGAAGCTGGAGGACGGTAG  20
Template        4249081  ....................  4249100

Reverse primer  1        AGGCCTTCGGTAATGATCTTGT  22
Template        4249436  ......................  4249415
```

>CP080007.1 Pseudomonas aeruginosa strain S-1 chromosome, complete genome

```
product length = 356
Forward primer  1       CTGAAGCTGGAGGACGGTAG  20
Template        481865  ....................  481884

Reverse primer  1       AGGCCTTCGGTAATGATCTTGT  22
Template        482220  ......................  482199
```

>CP061376.1 Pseudomonas aeruginosa strain HS17-127 chromosome, complete genome

```
product length = 356
Forward primer  1       CTGAAGCTGGAGGACGGTAG  20
Template        494565  ....................  494584

Reverse primer  1       AGGCCTTCGGTAATGATCTTGT  22
Template        494920  ......................  494899
```

>CP078564.1 Pseudomonas aeruginosa strain Colony464 chromosome

```
product length = 356
Forward primer  1        CTGAAGCTGGAGGACGGTAG  20
Template        4844215  ....................  4844234

Reverse primer  1        AGGCCTTCGGTAATGATCTTGT  22
Template        4844570  ......................  4844549
```

>CP053390.1 Pseudomonas aeruginosa strain TL1285 chromosome, complete genome

```
product length = 356
Forward primer  1        CTGAAGCTGGAGGACGGTAG  20
Template        3341532  ....................  3341551

Reverse primer  1        AGGCCTTCGGTAATGATCTTGT  22
Template        3341887  ......................  3341866
```

>CP075176.1 Pseudomonas aeruginosa strain PA790 chromosome, complete genome

```
product length = 356
Forward primer  1       CTGAAGCTGGAGGACGGTAG  20
Template        486711  ....................  486730

Reverse primer  1       AGGCCTTCGGTAATGATCTTGT  22
Template        487066  ......................  487045
```

>CP065948.1 Pseudomonas aeruginosa strain PAM68 chromosome, complete genome

```
product length = 356
Forward primer  1        CTGAAGCTGGAGGACGGTAG  20
Template        5783879  ....................  5783898

Reverse primer  1        AGGCCTTCGGTAATGATCTTGT  22
Template        5784234  ......................  5784213
```

>CP065947.1 Pseudomonas aeruginosa strain PAS6 chromosome, complete genome

```
product length = 356
Forward primer  1        CTGAAGCTGGAGGACGGTAG  20
Template        1580126  ....................  1580107

Reverse primer  1        AGGCCTTCGGTAATGATCTTGT  22
Template        1579771  ......................  1579792
```

>CP065374.1 Pseudomonas aeruginosa strain PAG7 chromosome, complete genome

```
product length = 356
Forward primer  1        CTGAAGCTGGAGGACGGTAG  20
Template        4048935  ....................  4048954

Reverse primer  1        AGGCCTTCGGTAATGATCTTGT  22
Template        4049290  ......................  4049269
```

>CP073080.1 Pseudomonas aeruginosa strain NDTH9845 chromosome, complete genome

```
product length = 356
Forward primer  1       CTGAAGCTGGAGGACGGTAG  20
Template        505631  ....................  505650

Reverse primer  1       AGGCCTTCGGTAATGATCTTGT  22
Template        505986  ......................  505965
```

>CP073082.1 Pseudomonas aeruginosa strain WTJH17 chromosome, complete genome

```
product length = 356
Forward primer  1       CTGAAGCTGGAGGACGGTAG  20
Template        465928  ....................  465947

Reverse primer  1       AGGCCTTCGGTAATGATCTTGT  22
Template        466283  ......................  466262
```

>CP071731.1 Pseudomonas aeruginosa strain LYSZa2 chromosome, complete genome

```
product length = 356
Forward primer  1        CTGAAGCTGGAGGACGGTAG  20
Template        3087474  ....................  3087493

Reverse primer  1        AGGCCTTCGGTAATGATCTTGT  22
Template        3087829  ......................  3087808
```

>CP071730.1 Pseudomonas aeruginosa strain LYSZa5 chromosome, complete genome

```
product length = 356
Forward primer  1        CTGAAGCTGGAGGACGGTAG  20
Template        3087485  ....................  3087504

Reverse primer  1        AGGCCTTCGGTAATGATCTTGT  22
Template        3087840  ......................  3087819
```

>AP024513.1 Pseudomonas aeruginosa Pa12 DNA, complete genome

```
product length = 356
Forward primer  1       CTGAAGCTGGAGGACGGTAG  20
Template        471502  ....................  471521

Reverse primer  1       AGGCCTTCGGTAATGATCTTGT  22
Template        471857  ......................  471836
```

>CP024024.1 Pseudomonas aeruginosa strain PARM801 chromosome, complete genome

```
product length = 356
Forward primer  1        CTGAAGCTGGAGGACGGTAG  20
Template        5388188  ....................  5388207

Reverse primer  1        AGGCCTTCGGTAATGATCTTGT  22
Template        5388543  ......................  5388522
```

>CP069198.1 Pseudomonas aeruginosa strain 152962 chromosome, complete genome

```
product length = 356
Forward primer  1        CTGAAGCTGGAGGACGGTAG  20
Template        4868111  ....................  4868092

Reverse primer  1        AGGCCTTCGGTAATGATCTTGT  22
Template        4867756  ......................  4867777
```

>CP060392.1 Pseudomonas aeruginosa strain 1903031130 chromosome, complete genome

```
product length = 356
Forward primer  1       CTGAAGCTGGAGGACGGTAG  20
Template        509467  ....................  509486

Reverse primer  1       AGGCCTTCGGTAATGATCTTGT  22
Template        509822  ......................  509801
```

>CP054845.1 Pseudomonas aeruginosa strain SE5429 chromosome, complete genome

```
product length = 356
Forward primer  1       CTGAAGCTGGAGGACGGTAG  20
Template        473041  ....................  473060

Reverse primer  1       AGGCCTTCGGTAATGATCTTGT  22
Template        473396  ......................  473375
```

>CP054844.1 Pseudomonas aeruginosa strain SE5357 chromosome, complete genome

```
product length = 356
Forward primer  1       CTGAAGCTGGAGGACGGTAG  20
Template        485147  ....................  485166

Reverse primer  1       AGGCCTTCGGTAATGATCTTGT  22
Template        485502  ......................  485481
```

>CP054843.1 Pseudomonas aeruginosa strain SE5352 chromosome, complete genome

```
product length = 356
Forward primer  1       CTGAAGCTGGAGGACGGTAG  20
Template        509452  ....................  509471

Reverse primer  1       AGGCCTTCGGTAATGATCTTGT  22
Template        509807  ......................  509786
```

>CP054581.1 Pseudomonas aeruginosa strain YTSEY8 chromosome, complete genome

```
product length = 356
Forward primer  1       CTGAAGCTGGAGGACGGTAG  20
Template        482805  ....................  482824

Reverse primer  1       AGGCCTTCGGTAATGATCTTGT  22
Template        483160  ......................  483139
```

>CP068239.1 Pseudomonas aeruginosa strain PA19-3047 chromosome, complete genome

```
product length = 356
Forward primer  1        CTGAAGCTGGAGGACGGTAG  20
Template        6531631  ....................  6531612

Reverse primer  1        AGGCCTTCGGTAATGATCTTGT  22
Template        6531276  ......................  6531297
```

>CP054623.1 Pseudomonas aeruginosa strain DL201330 chromosome, complete genome

```
product length = 356
Forward primer  1       CTGAAGCTGGAGGACGGTAG  20
Template        483232  ....................  483251

Reverse primer  1       AGGCCTTCGGTAATGATCTTGT  22
Template        483587  ......................  483566
```

>CP065966.1 Pseudomonas aeruginosa strain FDAARGOS_1041 chromosome, complete genome

```
product length = 356
Forward primer  1        CTGAAGCTGGAGGACGGTAG  20
Template        4070907  ....................  4070926

Reverse primer  1        AGGCCTTCGGTAATGATCTTGT  22
Template        4071262  ......................  4071241
```

>CP027857.1 Pseudomonas aeruginosa strain MPAO1 chromosome, complete genome

```
product length = 356
Forward primer  1       CTGAAGCTGGAGGACGGTAG  20
Template        472230  ....................  472249

Reverse primer  1       AGGCCTTCGGTAATGATCTTGT  22
Template        472585  ......................  472564
```

>CP065867.1 Pseudomonas aeruginosa strain TJ2014-049 chromosome, complete genome

```
product length = 356
Forward primer  1       CTGAAGCTGGAGGACGGTAG  20
Template        468800  ....................  468819

Reverse primer  1       AGGCCTTCGGTAATGATCTTGT  22
Template        469155  ......................  469134
```

>CP065865.1 Pseudomonas aeruginosa strain TJ2019-022 chromosome, complete genome

```
product length = 356
Forward primer  1       CTGAAGCTGGAGGACGGTAG  20
Template        474264  ....................  474283

Reverse primer  1       AGGCCTTCGGTAATGATCTTGT  22
Template        474619  ......................  474598
```

>CP065866.1 Pseudomonas aeruginosa strain TJ2019-017 chromosome, complete genome

```
product length = 356
Forward primer  1       CTGAAGCTGGAGGACGGTAG  20
Template        471640  ....................  471659

Reverse primer  1       AGGCCTTCGGTAATGATCTTGT  22
Template        471995  ......................  471974
```

>CP065848.1 Pseudomonas aeruginosa strain CMC-097 chromosome, complete genome

```
product length = 356
Forward primer  1       CTGAAGCTGGAGGACGGTAG  20
Template        487457  ....................  487476

Reverse primer  1       AGGCCTTCGGTAATGATCTTGT  22
Template        487812  ......................  487791
```

>CP065417.1 Pseudomonas aeruginosa isolate P23 chromosome, complete genome

```
product length = 356
Forward primer  1       CTGAAGCTGGAGGACGGTAG  20
Template        504441  ....................  504460

Reverse primer  1       AGGCCTTCGGTAATGATCTTGT  22
Template        504796  ......................  504775
```

>CP065412.1 Pseudomonas aeruginosa isolate P33 chromosome, complete genome

```
product length = 356
Forward primer  1       CTGAAGCTGGAGGACGGTAG  20
Template        504479  ....................  504498

Reverse primer  1       AGGCCTTCGGTAATGATCTTGT  22
Template        504834  ......................  504813
```

>CP046402.2 Pseudomonas aeruginosa strain SE5331 chromosome, complete genome

```
product length = 356
Forward primer  1        CTGAAGCTGGAGGACGGTAG  20
Template        2636263  ....................  2636244

Reverse primer  1        AGGCCTTCGGTAATGATCTTGT  22
Template        2635908  ......................  2635929
```

>CP046406.2 Pseudomonas aeruginosa strain SE5458 chromosome, complete genome

```
product length = 356
Forward primer  1       CTGAAGCTGGAGGACGGTAG  20
Template        473045  ....................  473064

Reverse primer  1       AGGCCTTCGGTAATGATCTTGT  22
Template        473400  ......................  473379
```

>CP045552.2 Pseudomonas aeruginosa strain YT12746 chromosome, complete genome

```
product length = 356
Forward primer  1       CTGAAGCTGGAGGACGGTAG  20
Template        482795  ....................  482814

Reverse primer  1       AGGCCTTCGGTAATGATCTTGT  22
Template        483150  ......................  483129
```

>CP060243.1 Pseudomonas aeruginosa strain A-I-1 chromosome, complete genome

```
product length = 356
Forward primer  1       CTGAAGCTGGAGGACGGTAG  20
Template        570334  ....................  570353

Reverse primer  1       AGGCCTTCGGTAATGATCTTGT  22
Template        570689  ......................  570668
```

>CP060242.1 Pseudomonas aeruginosa strain B-I-1 chromosome, complete genome

```
product length = 356
Forward primer  1       CTGAAGCTGGAGGACGGTAG  20
Template        944411  ....................  944430

Reverse primer  1       AGGCCTTCGGTAATGATCTTGT  22
Template        944766  ......................  944745
```

>CP060241.1 Pseudomonas aeruginosa strain C-I-1 chromosome, complete genome

```
product length = 356
Forward primer  1        CTGAAGCTGGAGGACGGTAG  20
Template        5838436  ....................  5838417

Reverse primer  1        AGGCCTTCGGTAATGATCTTGT  22
Template        5838081  ......................  5838102
```

>CP060240.1 Pseudomonas aeruginosa strain G-I-1 chromosome, complete genome

```
product length = 356
Forward primer  1       CTGAAGCTGGAGGACGGTAG  20
Template        591109  ....................  591128

Reverse primer  1       AGGCCTTCGGTAATGATCTTGT  22
Template        591464  ......................  591443
```

>LR898867.1 Pseudomonas aeruginosa isolate MINF_3A-sc-2280432 genome assembly, chromosome: 1

```
product length = 356
Forward primer  1       CTGAAGCTGGAGGACGGTAG  20
Template        478437  ....................  478456

Reverse primer  1       AGGCCTTCGGTAATGATCTTGT  22
Template        478792  ......................  478771
```

>LR890619.1 Pseudomonas aeruginosa isolate MINF_7A-sc-2280434 genome assembly, chromosome: 1

```
product length = 356
Forward primer  1       CTGAAGCTGGAGGACGGTAG  20
Template        471409  ....................  471428

Reverse primer  1       AGGCCTTCGGTAATGATCTTGT  22
```

```
Template        471764  ......................  471743
```

>CP061034.1 Pseudomonas aeruginosa strain PA3 chromosome, complete genome

```
product length = 356
Forward primer  1       CTGAAGCTGGAGGACGGTAG  20
Template        477672  ....................  477691

Reverse primer  1       AGGCCTTCGGTAATGATCTTGT  22
Template        478027  ......................  478006
```

>CP060703.1 Pseudomonas aeruginosa strain NRD619 chromosome, complete genome

```
product length = 356
Forward primer  1       CTGAAGCTGGAGGACGGTAG  20
Template        464943  ....................  464962

Reverse primer  1       AGGCCTTCGGTAATGATCTTGT  22
Template        465298  ......................  465277
```

>CP060086.1 Pseudomonas aeruginosa strain JNQH-PA57 chromosome, complete genome

```
product length = 356
Forward primer  1       CTGAAGCTGGAGGACGGTAG  20
Template        496062  ....................  496081

Reverse primer  1       AGGCCTTCGGTAATGATCTTGT  22
Template        496417  ......................  496396
```

>CP059995.1 Pseudomonas aeruginosa strain NY3045 chromosome, complete genome

```
product length = 356
Forward primer  1       CTGAAGCTGGAGGACGGTAG  20
Template        499405  ....................  499424

Reverse primer  1       AGGCCTTCGGTAATGATCTTGT  22
Template        499760  ......................  499739
```

>CP059063.1 Pseudomonas aeruginosa strain GIMC5034:PA52Ts32 chromosome

```
product length = 356
Forward primer  1        CTGAAGCTGGAGGACGGTAG  20
Template        6320210  ....................  6320191

Reverse primer  1        AGGCCTTCGGTAATGATCTTGT  22
Template        6319855  ......................  6319876
```

>CP058331.1 Pseudomonas aeruginosa strain ACR22 chromosome, complete genome

```
product length = 356
Forward primer  1        CTGAAGCTGGAGGACGGTAG  20
Template        1031697  ....................  1031716
```

```
Reverse primer  1        AGGCCTTCGGTAATGATCTTGT  22
Template        1032052  ......................  1032031
```

>CP058333.1 Pseudomonas aeruginosa strain ACR20 chromosome, complete genome

```
product length = 356
Forward primer  1        CTGAAGCTGGAGGACGGTAG  20
Template        5177712  ....................  5177693

Reverse primer  1        AGGCCTTCGGTAATGATCTTGT  22
Template        5177357  ......................  5177378
```

>CP053119.1 Pseudomonas aeruginosa strain A17CT chromosome

```
product length = 356
Forward primer  1       CTGAAGCTGGAGGACGGTAG  20
Template        472826  ....................  472845

Reverse primer  1       AGGCCTTCGGTAATGATCTTGT  22
Template        473181  ......................  473160
```

>CP053118.1 Pseudomonas aeruginosa strain A17PBS chromosome

```
product length = 356
Forward primer  1       CTGAAGCTGGAGGACGGTAG  20
Template        472718  ....................  472737

Reverse primer  1       AGGCCTTCGGTAATGATCTTGT  22
Template        473073  ......................  473052
```

>CP053117.1 Pseudomonas aeruginosa strain P16CT chromosome

```
product length = 356
Forward primer  1       CTGAAGCTGGAGGACGGTAG  20
Template        472720  ....................  472739

Reverse primer  1       AGGCCTTCGGTAATGATCTTGT  22
Template        473075  ......................  473054
```

>CP053116.1 Pseudomonas aeruginosa strain P16PBS chromosome

```
product length = 356
Forward primer  1       CTGAAGCTGGAGGACGGTAG  20
Template        472718  ....................  472737

Reverse primer  1       AGGCCTTCGGTAATGATCTTGT  22
Template        473073  ......................  473052
```

>CP053115.1 Pseudomonas aeruginosa strain P4CT chromosome

```
product length = 356
Forward primer  1       CTGAAGCTGGAGGACGGTAG  20
Template        472718  ....................  472737
```

```
Reverse primer  1       AGGCCTTCGGTAATGATCTTGT  22
Template        473073  ......................  473052
```

>CP053114.1 Pseudomonas aeruginosa strain P4PBS chromosome

```
product length = 356
Forward primer  1       CTGAAGCTGGAGGACGGTAG  20
Template        472718  ....................  472737

Reverse primer  1       AGGCCTTCGGTAATGATCTTGT  22
Template        473073  ......................  473052
```

>CP053113.1 Pseudomonas aeruginosa strain PAO1CT chromosome

```
product length = 356
Forward primer  1       CTGAAGCTGGAGGACGGTAG  20
Template        472772  ....................  472791

Reverse primer  1       AGGCCTTCGGTAATGATCTTGT  22
Template        473127  ......................  473106
```

>CP053112.1 Pseudomonas aeruginosa strain PAO1PBS chromosome

```
product length = 356
Forward primer  1       CTGAAGCTGGAGGACGGTAG  20
Template        472718  ....................  472737

Reverse primer  1       AGGCCTTCGGTAATGATCTTGT  22
Template        473073  ......................  473052
```

>CP053111.1 Pseudomonas aeruginosa strain UAB2CT chromosome

```
product length = 356
Forward primer  1       CTGAAGCTGGAGGACGGTAG  20
Template        472723  ....................  472742

Reverse primer  1       AGGCCTTCGGTAATGATCTTGT  22
Template        473078  ......................  473057
```

>CP053110.1 Pseudomonas aeruginosa strain UAB2PBS chromosome

```
product length = 356
Forward primer  1       CTGAAGCTGGAGGACGGTAG  20
Template        472718  ....................  472737

Reverse primer  1       AGGCCTTCGGTAATGATCTTGT  22
Template        473073  ......................  473052
```

>CP058332.1 Pseudomonas aeruginosa strain B18 chromosome, complete genome

```
product length = 356
Forward primer  1        CTGAAGCTGGAGGACGGTAG  20
```

```
Template        1242378  ....................  1242359

Reverse primer  1        AGGCCTTCGGTAATGATCTTGT  22
Template        1242023  ......................  1242044
```

>CP058323.1 Pseudomonas aeruginosa strain LV chromosome

```
product length = 356
Forward primer  1        CTGAAGCTGGAGGACGGTAG  20
Template        1114881  ....................  1114900

Reverse primer  1        AGGCCTTCGGTAATGATCTTGT  22
Template        1115236  ......................  1115215
```

>CP046405.1 Pseudomonas aeruginosa strain SE5443 chromosome, complete genome

```
product length = 356
Forward primer  1       CTGAAGCTGGAGGACGGTAG  20
Template        481106  ....................  481125

Reverse primer  1       AGGCCTTCGGTAATGATCTTGT  22
Template        481461  ......................  481440
```

>CP046404.1 Pseudomonas aeruginosa strain SE5416 chromosome, complete genome

```
product length = 356
Forward primer  1       CTGAAGCTGGAGGACGGTAG  20
Template        481256  ....................  481275

Reverse primer  1       AGGCCTTCGGTAATGATCTTGT  22
Template        481611  ......................  481590
```

>CP046403.1 Pseudomonas aeruginosa strain SE5369 chromosome, complete genome

```
product length = 356
Forward primer  1       CTGAAGCTGGAGGACGGTAG  20
Template        530661  ....................  530680

Reverse primer  1       AGGCCTTCGGTAATGATCTTGT  22
Template        531016  ......................  530995
```

>CP056774.1 Pseudomonas aeruginosa strain CDN129 chromosome, complete genome

```
product length = 356
Forward primer  1        CTGAAGCTGGAGGACGGTAG  20
Template        5131778  ....................  5131759

Reverse primer  1        AGGCCTTCGGTAATGATCTTGT  22
Template        5131423  ......................  5131444
```

>CP056100.1 Pseudomonas aeruginosa strain PABCH01 chromosome

```
product length = 356
```

```
Forward primer  1       CTGAAGCTGGAGGACGGTAG  20
Template        484121  ....................  484140

Reverse primer  1       AGGCCTTCGGTAATGATCTTGT  22
Template        484476  ......................  484455
```

>CP056090.1 Pseudomonas aeruginosa strain PABCH42 chromosome

```
product length = 356
Forward primer  1       CTGAAGCTGGAGGACGGTAG  20
Template        735182  ....................  735163

Reverse primer  1       AGGCCTTCGGTAATGATCTTGT  22
Template        734827  ......................  734848
```

>CP056089.1 Pseudomonas aeruginosa strain PABCH46 chromosome

```
product length = 356
Forward primer  1       CTGAAGCTGGAGGACGGTAG  20
Template        522501  ....................  522520

Reverse primer  1       AGGCCTTCGGTAATGATCTTGT  22
Template        522856  ......................  522835
```

>CP056095.1 Pseudomonas aeruginosa strain PABCH09 chromosome

```
product length = 356
Forward primer  1       CTGAAGCTGGAGGACGGTAG  20
Template        526421  ....................  526440

Reverse primer  1       AGGCCTTCGGTAATGATCTTGT  22
Template        526776  ......................  526755
```

>CP056092.1 Pseudomonas aeruginosa strain PABCH14 chromosome

```
product length = 356
Forward primer  1       CTGAAGCTGGAGGACGGTAG  20
Template        624807  ....................  624826

Reverse primer  1       AGGCCTTCGGTAATGATCTTGT  22
Template        625162  ......................  625141
```

>CP056101.1 Pseudomonas aeruginosa strain PABCH45 chromosome

```
product length = 356
Forward primer  1       CTGAAGCTGGAGGACGGTAG  20
Template        482247  ....................  482266

Reverse primer  1       AGGCCTTCGGTAATGATCTTGT  22
Template        482602  ......................  482581
```

>CP056098.1 Pseudomonas aeruginosa strain PABCH05 chromosome

```
product length = 356
Forward primer  1       CTGAAGCTGGAGGACGGTAG  20
Template        461916  ....................  461935

Reverse primer  1       AGGCCTTCGGTAATGATCTTGT  22
Template        462271  ......................  462250
```

>CP056094.1 Pseudomonas aeruginosa strain PABCH10 chromosome

```
product length = 356
Forward primer  1       CTGAAGCTGGAGGACGGTAG  20
Template        474400  ....................  474419

Reverse primer  1       AGGCCTTCGGTAATGATCTTGT  22
Template        474755  ......................  474734
```

>CP056093.1 Pseudomonas aeruginosa strain PABCH13 chromosome

```
product length = 356
Forward primer  1       CTGAAGCTGGAGGACGGTAG  20
Template        480780  ....................  480799

Reverse primer  1       AGGCCTTCGGTAATGATCTTGT  22
Template        481135  ......................  481114
```

>CP054591.1 Pseudomonas aeruginosa strain CDN118 chromosome, complete genome

```
product length = 356
Forward primer  1        CTGAAGCTGGAGGACGGTAG  20
Template        6047095  ....................  6047076

Reverse primer  1        AGGCCTTCGGTAATGATCTTGT  22
Template        6046740  ......................  6046761
```

>CP050335.1 Pseudomonas aeruginosa strain DVT401 chromosome, complete genome

```
product length = 356
Forward primer  1       CTGAAGCTGGAGGACGGTAG  20
Template        466239  ....................  466258

Reverse primer  1       AGGCCTTCGGTAATGATCTTGT  22
Template        466594  ......................  466573
```

>CP050334.1 Pseudomonas aeruginosa strain DVT410 chromosome, complete genome

```
product length = 356
Forward primer  1       CTGAAGCTGGAGGACGGTAG  20
Template        467618  ....................  467637

Reverse primer  1       AGGCCTTCGGTAATGATCTTGT  22
Template        467973  ......................  467952
```

>CP050333.1 Pseudomonas aeruginosa strain DVT412 chromosome, complete genome

```
product length = 356
Forward primer  1       CTGAAGCTGGAGGACGGTAG  20
Template        482494  ....................  482513

Reverse primer  1       AGGCCTTCGGTAATGATCTTGT  22
Template        482849  ......................  482828
```

>CP050332.1 Pseudomonas aeruginosa strain DVT413 chromosome, complete genome

```
product length = 356
Forward primer  1       CTGAAGCTGGAGGACGGTAG  20
Template        540906  ....................  540925

Reverse primer  1       AGGCCTTCGGTAATGATCTTGT  22
Template        541261  ......................  541240
```

>CP050331.1 Pseudomonas aeruginosa strain DVT414 chromosome, complete genome

```
product length = 356
Forward primer  1       CTGAAGCTGGAGGACGGTAG  20
Template        466509  ....................  466528

Reverse primer  1       AGGCCTTCGGTAATGATCTTGT  22
Template        466864  ......................  466843
```

>CP050329.1 Pseudomonas aeruginosa strain DVT417 chromosome, complete genome

```
product length = 356
Forward primer  1       CTGAAGCTGGAGGACGGTAG  20
Template        471963  ....................  471982

Reverse primer  1       AGGCCTTCGGTAATGATCTTGT  22
Template        472318  ......................  472297
```

>CP050328.1 Pseudomonas aeruginosa strain DVT419 chromosome, complete genome

```
product length = 356
Forward primer  1       CTGAAGCTGGAGGACGGTAG  20
Template        461793  ....................  461812

Reverse primer  1       AGGCCTTCGGTAATGATCTTGT  22
Template        462148  ......................  462127
```

>CP050327.1 Pseudomonas aeruginosa strain DVT421 chromosome, complete genome

```
product length = 356
Forward primer  1       CTGAAGCTGGAGGACGGTAG  20
Template        465799  ....................  465818

Reverse primer  1       AGGCCTTCGGTAATGATCTTGT  22
Template        466154  ......................  466133
```

>CP050325.1 Pseudomonas aeruginosa strain DVT425 chromosome, complete genome

```
product length = 356
Forward primer  1        CTGAAGCTGGAGGACGGTAG  20
Template        3891789  ....................  3891770

Reverse primer  1        AGGCCTTCGGTAATGATCTTGT  22
Template        3891434  ......................  3891455
```

>CP050324.1 Pseudomonas aeruginosa strain DVT427 chromosome, complete genome

```
product length = 356
Forward primer  1       CTGAAGCTGGAGGACGGTAG  20
Template        465392  ....................  465411

Reverse primer  1       AGGCCTTCGGTAATGATCTTGT  22
Template        465747  ......................  465726
```

>CP050323.1 Pseudomonas aeruginosa strain DVT429 chromosome, complete genome

```
product length = 356
Forward primer  1       CTGAAGCTGGAGGACGGTAG  20
Template        459227  ....................  459246

Reverse primer  1       AGGCCTTCGGTAATGATCTTGT  22
Template        459582  ......................  459561
```

>CP050322.1 Pseudomonas aeruginosa strain DVT729 chromosome, complete genome

```
product length = 356
Forward primer  1        CTGAAGCTGGAGGACGGTAG  20
Template        5850412  ....................  5850393

Reverse primer  1        AGGCCTTCGGTAATGATCTTGT  22
Template        5850057  ......................  5850078
```

>CP054572.1 Pseudomonas sp. FDAARGOS_761 chromosome, complete genome

```
product length = 356
Forward primer  1        CTGAAGCTGGAGGACGGTAG  20
Template        5114159  ....................  5114140

Reverse primer  1        AGGCCTTCGGTAATGATCTTGT  22
Template        5113804  ......................  5113825
```

>CP054473.1 Pseudomonas aeruginosa strain PAAK095 chromosome, complete genome

```
product length = 356
Forward primer  1       CTGAAGCTGGAGGACGGTAG  20
Template        348251  ....................  348270

Reverse primer  1       AGGCCTTCGGTAATGATCTTGT  22
Template        348606  ......................  348585
```

>CP054472.1 Pseudomonas aeruginosa strain PAAK088 chromosome, complete genome

```
product length = 356
Forward primer  1       CTGAAGCTGGAGGACGGTAG  20
Template        469358  ....................  469377

Reverse primer  1       AGGCCTTCGGTAATGATCTTGT  22
Template        469713  ......................  469692
```

>CP050052.1 Pseudomonas aeruginosa strain LIUYANG-E chromosome, complete genome

```
product length = 356
Forward primer  1       CTGAAGCTGGAGGACGGTAG  20
Template        472230  ....................  472249

Reverse primer  1       AGGCCTTCGGTAATGATCTTGT  22
Template        472585  ......................  472564
```

>CP050054.1 Pseudomonas aeruginosa strain LIUYANG-A chromosome, complete genome

```
product length = 356
Forward primer  1       CTGAAGCTGGAGGACGGTAG  20
Template        472230  ....................  472249

Reverse primer  1       AGGCCTTCGGTAATGATCTTGT  22
Template        472585  ......................  472564
```

>CP050053.1 Pseudomonas aeruginosa strain LIUYANG-C chromosome, complete genome

```
product length = 356
Forward primer  1       CTGAAGCTGGAGGACGGTAG  20
Template        472230  ....................  472249

Reverse primer  1       AGGCCTTCGGTAATGATCTTGT  22
Template        472585  ......................  472564
```

>CP053922.1 Pseudomonas aeruginosa strain YD001 chromosome, complete genome

```
product length = 356
Forward primer  1       CTGAAGCTGGAGGACGGTAG  20
Template        483217  ....................  483236

Reverse primer  1       AGGCCTTCGGTAATGATCTTGT  22
Template        483572  ......................  483551
```

>CP053917.1 Pseudomonas aeruginosa strain PSE6684 chromosome, complete genome

```
product length = 356
Forward primer  1        CTGAAGCTGGAGGACGGTAG  20
Template        2375995  ....................  2375976

Reverse primer  1        AGGCCTTCGGTAATGATCTTGT  22
Template        2375640  ......................  2375661
```

>CP053706.1 Pseudomonas aeruginosa strain PAC1 chromosome, complete genome

```
product length = 356
Forward primer  1        CTGAAGCTGGAGGACGGTAG  20
Template        5986202  ....................  5986221

Reverse primer  1        AGGCCTTCGGTAATGATCTTGT  22
Template        5986557  ......................  5986536
```

>CP053705.1 Pseudomonas aeruginosa strain PAC6 chromosome, complete genome

```
product length = 356
Forward primer  1       CTGAAGCTGGAGGACGGTAG  20
Template        480833  ....................  480852

Reverse primer  1       AGGCCTTCGGTAATGATCTTGT  22
Template        481188  ......................  481167
```

>CP053686.1 Pseudomonas aeruginosa strain SCAID PHRX1-2019 chromosome

```
product length = 356
Forward primer  1        CTGAAGCTGGAGGACGGTAG  20
Template        3752968  ....................  3752987

Reverse primer  1        AGGCCTTCGGTAATGATCTTGT  22
Template        3753323  ......................  3753302
```

>CP044533.1 Pseudomonas aeruginosa strain Ps33 chromosome

```
product length = 356
Forward primer  1        CTGAAGCTGGAGGACGGTAG  20
Template        5060475  ....................  5060494

Reverse primer  1        AGGCCTTCGGTAATGATCTTGT  22
Template        5060830  ......................  5060809
```

>CP051770.1 Pseudomonas aeruginosa strain GIMC5021:PA52Ts17, complete sequence

```
product length = 356
Forward primer  1        CTGAAGCTGGAGGACGGTAG  20
Template        6347470  ....................  6347451

Reverse primer  1        AGGCCTTCGGTAATGATCTTGT  22
Template        6347115  ......................  6347136
```

>CP051768.1 Pseudomonas aeruginosa strain GIMC5020:PA52Ts2, complete sequence

```
product length = 356
Forward primer  1        CTGAAGCTGGAGGACGGTAG  20
Template        6310799  ....................  6310780

Reverse primer  1        AGGCCTTCGGTAATGATCTTGT  22
Template        6310444  ......................  6310465
```

>CP051766.1 Pseudomonas aeruginosa strain GIMC5019:PA52Ts1, complete sequence

```
product length = 356
Forward primer  1        CTGAAGCTGGAGGACGGTAG  20
Template        6356372  ....................  6356353

Reverse primer  1        AGGCCTTCGGTAATGATCTTGT  22
Template        6356017  ......................  6356038
```

>CP053028.1 Pseudomonas aeruginosa PAO1 chromosome, complete genome

```
product length = 356
Forward primer  1       CTGAAGCTGGAGGACGGTAG  20
Template        472714  ....................  472733

Reverse primer  1       AGGCCTTCGGTAATGATCTTGT  22
Template        473069  ......................  473048
```

>CP052759.1 Pseudomonas aeruginosa strain LYT4 chromosome, complete genome

```
product length = 356
Forward primer  1       CTGAAGCTGGAGGACGGTAG  20
Template        476029  ....................  476048

Reverse primer  1       AGGCCTTCGGTAATGATCTTGT  22
Template        476384  ......................  476363
```

>CP051547.1 Pseudomonas aeruginosa strain AA2 chromosome, complete genome

```
product length = 356
Forward primer  1       CTGAAGCTGGAGGACGGTAG  20
Template        476205  ....................  476224

Reverse primer  1       AGGCCTTCGGTAATGATCTTGT  22
Template        476560  ......................  476539
```

>CP046602.1 Pseudomonas aeruginosa strain CMC-115 chromosome, complete genome

```
product length = 356
Forward primer  1       CTGAAGCTGGAGGACGGTAG  20
Template        481931  ....................  481950

Reverse primer  1       AGGCCTTCGGTAATGATCTTGT  22
Template        482286  ......................  482265
```

>CP045916.1 Pseudomonas aeruginosa strain CF39S chromosome, complete genome

```
product length = 356
Forward primer  1       CTGAAGCTGGAGGACGGTAG  20
Template        478175  ....................  478194

Reverse primer  1       AGGCCTTCGGTAATGATCTTGT  22
Template        478530  ......................  478509
```

>CP045002.1 Pseudomonas aeruginosa strain PAG5 chromosome, complete genome

```
product length = 356
Forward primer  1       CTGAAGCTGGAGGACGGTAG  20
Template        480499  ....................  480518

Reverse primer  1       AGGCCTTCGGTAATGATCTTGT  22
Template        480854  ......................  480833
```

>CP049161.1 Pseudomonas aeruginosa strain MS14403 chromosome, complete genome

```
product length = 356
Forward primer  1       CTGAAGCTGGAGGACGGTAG  20
Template        472028  ....................  472047

Reverse primer  1       AGGCCTTCGGTAATGATCTTGT  22
Template        472383  ......................  472362
```

>CP048791.1 Pseudomonas aeruginosa strain VIT PC9 chromosome, complete genome

```
product length = 356
Forward primer  1       CTGAAGCTGGAGGACGGTAG  20
Template        258061  ....................  258042

Reverse primer  1       AGGCCTTCGGTAATGATCTTGT  22
Template        257706  ......................  257727
```

>LR739071.1 Pseudomonas aeruginosa strain C7-25 genome assembly, chromosome: C7-25

```
product length = 356
Forward primer  1       CTGAAGCTGGAGGACGGTAG  20
Template        469211  ....................  469230

Reverse primer  1       AGGCCTTCGGTAATGATCTTGT  22
Template        469566  ......................  469545
```

>LR739069.1 Pseudomonas aeruginosa strain PcyII-40 genome assembly, chromosome: PcyII-40

```
product length = 356
Forward primer  1       CTGAAGCTGGAGGACGGTAG  20
Template        549292  ....................  549311

Reverse primer  1       AGGCCTTCGGTAATGATCTTGT  22
Template        549647  ......................  549626
```

>LR739068.1 Pseudomonas aeruginosa strain PcyII-29 genome assembly, chromosome: PcyII-29

```
product length = 356
Forward primer  1       CTGAAGCTGGAGGACGGTAG  20
Template        478147  ....................  478166

Reverse primer  1       AGGCCTTCGGTAATGATCTTGT  22
Template        478502  ......................  478481
```

>CP047697.1 Pseudomonas aeruginosa strain RD1-3 chromosome, complete genome

```
product length = 356
Forward primer  1       CTGAAGCTGGAGGACGGTAG  20
Template        466911  ....................  466930

Reverse primer  1       AGGCCTTCGGTAATGATCTTGT  22
Template        467266  ......................  467245
```

>CP047592.1 Pseudomonas aeruginosa strain INP-43 chromosome, complete genome

```
product length = 356
Forward primer  1        CTGAAGCTGGAGGACGGTAG  20
Template        1659751  ....................  1659770

Reverse primer  1        AGGCCTTCGGTAATGATCTTGT  22
Template        1660106  ......................  1660085
```

>CP028132.1 Pseudomonas aeruginosa strain YB01 chromosome, complete genome

```
product length = 356
Forward primer  1       CTGAAGCTGGAGGACGGTAG  20
Template        469143  ....................  469162

Reverse primer  1       AGGCCTTCGGTAATGATCTTGT  22
Template        469498  ......................  469477
```

>CP025056.3 Pseudomonas aeruginosa strain PB367 chromosome, complete genome

```
product length = 356
Forward primer  1       CTGAAGCTGGAGGACGGTAG  20
Template        473672  ....................  473691

Reverse primer  1       AGGCCTTCGGTAATGATCTTGT  22
Template        474027  ......................  474006
```

>CP025055.2 Pseudomonas aeruginosa strain PB350 chromosome, complete genome

```
product length = 356
Forward primer  1       CTGAAGCTGGAGGACGGTAG  20
Template        473672  ....................  473691

Reverse primer  1       AGGCCTTCGGTAATGATCTTGT  22
Template        474027  ......................  474006
```

>CP047069.1 Pseudomonas aeruginosa strain Environ_1 chromosome

```
product length = 356
Forward primer  1       CTGAAGCTGGAGGACGGTAG  20
Template        469107  ....................  469126

Reverse primer  1       AGGCCTTCGGTAATGATCTTGT  22
Template        469462  ......................  469441
```

>CP047063.1 Pseudomonas aeruginosa strain delta6_4 chromosome

```
product length = 356
Forward primer  1       CTGAAGCTGGAGGACGGTAG  20
Template        472716  ....................  472735

Reverse primer  1       AGGCCTTCGGTAATGATCTTGT  22
Template        473071  ......................  473050
```

>CP047070.1 Pseudomonas aeruginosa strain Environ_2 chromosome

```
product length = 356
Forward primer  1       CTGAAGCTGGAGGACGGTAG  20
Template        472712  ....................  472731

Reverse primer  1       AGGCCTTCGGTAATGATCTTGT  22
Template        473067  ......................  473046
```

>CP047064.1 Pseudomonas aeruginosa strain delta6_5 chromosome

```
product length = 356
Forward primer  1       CTGAAGCTGGAGGACGGTAG  20
Template        472716  ....................  472735

Reverse primer  1       AGGCCTTCGGTAATGATCTTGT  22
Template        473071  ......................  473050
```

>CP047061.1 Pseudomonas aeruginosa strain delta6_2 chromosome

```
product length = 356
Forward primer  1       CTGAAGCTGGAGGACGGTAG  20
Template        472714  ....................  472733

Reverse primer  1       AGGCCTTCGGTAATGATCTTGT  22
Template        473069  ......................  473048
```

>CP047062.1 Pseudomonas aeruginosa strain delta6_3 chromosome

```
product length = 356
Forward primer  1       CTGAAGCTGGAGGACGGTAG  20
Template        472715  ....................  472734

Reverse primer  1       AGGCCTTCGGTAATGATCTTGT  22
Template        473070  ......................  473049
```

>CP047067.1 Pseudomonas aeruginosa strain Cas9_1 chromosome

```
product length = 356
Forward primer  1       CTGAAGCTGGAGGACGGTAG  20
Template        472712  ....................  472731

Reverse primer  1       AGGCCTTCGGTAATGATCTTGT  22
Template        473067  ......................  473046
```

>CP047066.1 Pseudomonas aeruginosa strain delta10 chromosome

```
product length = 356
Forward primer  1       CTGAAGCTGGAGGACGGTAG  20
Template        472712  ....................  472731

Reverse primer  1       AGGCCTTCGGTAATGATCTTGT  22
Template        473067  ......................  473046
```

>CP047068.1 Pseudomonas aeruginosa strain Cas9_2 chromosome

```
product length = 356
Forward primer  1       CTGAAGCTGGAGGACGGTAG  20
Template        472712  ....................  472731

Reverse primer  1       AGGCCTTCGGTAATGATCTTGT  22
Template        473067  ......................  473046
```

>CP047065.1 Pseudomonas aeruginosa strain delta6_6 chromosome

```
product length = 356
Forward primer  1       CTGAAGCTGGAGGACGGTAG  20
Template        472713  ....................  472732

Reverse primer  1       AGGCCTTCGGTAATGATCTTGT  22
Template        473068  ......................  473047
```

>CP039990.1 Pseudomonas aeruginosa strain T2101 chromosome, complete genome

```
product length = 356
Forward primer  1       CTGAAGCTGGAGGACGGTAG  20
Template        488009  ....................  488028

Reverse primer  1       AGGCCTTCGGTAATGATCTTGT  22
Template        488364  ......................  488343
```

>CP039988.1 Pseudomonas aeruginosa strain T2436 chromosome, complete genome

```
product length = 356
Forward primer  1       CTGAAGCTGGAGGACGGTAG  20
Template        485123  ....................  485142

Reverse primer  1       AGGCCTTCGGTAATGATCTTGT  22
Template        485478  ......................  485457
```

>CP046069.1 Pseudomonas aeruginosa strain KRP1 chromosome, complete genome

```
product length = 356
Forward primer  1       CTGAAGCTGGAGGACGGTAG  20
Template        471460  ....................  471479

Reverse primer  1       AGGCCTTCGGTAATGATCTTGT  22
Template        471815  ......................  471794
```

>CP046060.1 Pseudomonas aeruginosa strain 1811-18R001 chromosome, complete genome

```
product length = 356
Forward primer  1       CTGAAGCTGGAGGACGGTAG  20
Template        465004  ....................  465023

Reverse primer  1       AGGCCTTCGGTAATGATCTTGT  22
Template        465359  ......................  465338
```

>CP046061.1 Pseudomonas aeruginosa strain 1811-13R031 chromosome, complete genome

```
product length = 356
Forward primer  1       CTGAAGCTGGAGGACGGTAG  20
Template        465004  ....................  465023

Reverse primer  1       AGGCCTTCGGTAATGATCTTGT  22
Template        465359  ......................  465338
```

>CP041945.1 Pseudomonas aeruginosa strain ST773 chromosome, complete genome

```
product length = 356
Forward primer  1       CTGAAGCTGGAGGACGGTAG  20
Template        486069  ....................  486088

Reverse primer  1       AGGCCTTCGGTAATGATCTTGT  22
Template        486424  ......................  486403
```

>CP045739.1 Pseudomonas aeruginosa strain AG1 chromosome, complete genome

```
product length = 356
Forward primer  1       CTGAAGCTGGAGGACGGTAG  20
Template        556782  ....................  556801

Reverse primer  1       AGGCCTTCGGTAATGATCTTGT  22
Template        557137  ......................  557116
```

>CP045768.1 Pseudomonas aeruginosa strain CFSAN084950 chromosome, complete genome

```
product length = 356
Forward primer  1        CTGAAGCTGGAGGACGGTAG  20
Template        5892976  ....................  5892995

Reverse primer  1        AGGCCTTCGGTAATGATCTTGT  22
Template        5893331  ......................  5893310
```

>CP042967.1 Pseudomonas aeruginosa PA99 chromosome, complete genome

```
product length = 356
Forward primer  1        CTGAAGCTGGAGGACGGTAG  20
Template        3563339  ....................  3563358

Reverse primer  1        AGGCCTTCGGTAATGATCTTGT  22
Template        3563694  ......................  3563673
```

>CP024630.1 Pseudomonas aeruginosa strain PA59 chromosome, complete genome

```
product length = 356
Forward primer  1       CTGAAGCTGGAGGACGGTAG  20
Template        465982  ....................  466001

Reverse primer  1       AGGCCTTCGGTAATGATCTTGT  22
Template        466337  ......................  466316
```

>CP043549.1 Pseudomonas aeruginosa strain GIMC5002:PAT-169 chromosome

```
product length = 356
Forward primer  1        CTGAAGCTGGAGGACGGTAG  20
Template        5834308  ....................  5834289

Reverse primer  1        AGGCCTTCGGTAATGATCTTGT  22
Template        5833953  ......................  5833974
```

>CP043483.1 Pseudomonas aeruginosa strain GIMC5001:PAT-23 chromosome

```
product length = 356
Forward primer  1       CTGAAGCTGGAGGACGGTAG  20
Template        508230  ....................  508249

Reverse primer  1       AGGCCTTCGGTAATGATCTTGT  22
Template        508585  ......................  508564
```

>LR700248.1 Pseudomonas aeruginosa isolate ID40 genome assembly, chromosome: ID40_omosome

```
product length = 356
Forward primer  1        CTGAAGCTGGAGGACGGTAG  20
Template        4385118  ....................  4385137

Reverse primer  1        AGGCCTTCGGTAATGATCTTGT  22
Template        4385473  ......................  4385452
```

>CP042269.1 Pseudomonas aeruginosa strain HOU1 chromosome, complete genome

```
product length = 356
Forward primer  1       CTGAAGCTGGAGGACGGTAG  20
Template        480619  ....................  480638

Reverse primer  1       AGGCCTTCGGTAATGATCTTGT  22
Template        480974  ......................  480953
```

>CP043328.1 Pseudomonas aeruginosa strain CCUG 51971 chromosome, complete genome

```
product length = 356
Forward primer  1       CTGAAGCTGGAGGACGGTAG  20
Template        473134  ....................  473153

Reverse primer  1       AGGCCTTCGGTAATGATCTTGT  22
Template        473489  ......................  473468
```

>CP028959.1 Pseudomonas aeruginosa strain IMP66 chromosome, complete genome

```
product length = 356
Forward primer  1       CTGAAGCTGGAGGACGGTAG  20
Template        476816  ....................  476835

Reverse primer  1       AGGCCTTCGGTAATGATCTTGT  22
Template        477171  ......................  477150
```

>CP028848.1 Pseudomonas aeruginosa strain IMP67 chromosome, complete genome

```
product length = 356
Forward primer  1       CTGAAGCTGGAGGACGGTAG  20
Template        476816  ....................  476835

Reverse primer  1       AGGCCTTCGGTAATGATCTTGT  22
Template        477171  ......................  477150
```

>CP028849.1 Pseudomonas aeruginosa strain IMP68 chromosome, complete genome

```
product length = 356
Forward primer  1       CTGAAGCTGGAGGACGGTAG  20
Template        476814  ....................  476833

Reverse primer  1       AGGCCTTCGGTAATGATCTTGT  22
Template        477169  ......................  477148
```

>CP040684.1 Pseudomonas aeruginosa strain C79 chromosome, complete genome

```
product length = 356
Forward primer  1        CTGAAGCTGGAGGACGGTAG  20
Template        4247497  ....................  4247478

Reverse primer  1        AGGCCTTCGGTAATGATCTTGT  22
Template        4247142  ......................  4247163
```

>CP041785.1 Pseudomonas aeruginosa strain SCAID WND3-2019 chromosome

```
product length = 356
Forward primer  1        CTGAAGCTGGAGGACGGTAG  20
Template        5904458  ....................  5904477

Reverse primer  1        AGGCCTTCGGTAATGATCTTGT  22
Template        5904813  ......................  5904792
```

>CP041787.1 Pseudomonas aeruginosa strain SCAID WND1-2019 chromosome

```
product length = 356
Forward primer  1        CTGAAGCTGGAGGACGGTAG  20
Template        4754512  ....................  4754531

Reverse primer  1        AGGCCTTCGGTAATGATCTTGT  22
Template        4754867  ......................  4754846
```

>CP041786.1 Pseudomonas aeruginosa strain SCAID WND2-2019 chromosome

```
product length = 356
Forward primer  1       CTGAAGCTGGAGGACGGTAG  20
Template        469770  ....................  469789

Reverse primer  1       AGGCCTTCGGTAATGATCTTGT  22
Template        470125  ......................  470104
```

>CP041772.1 Pseudomonas aeruginosa strain 243931 chromosome, complete genome

```
product length = 356
Forward primer  1        CTGAAGCTGGAGGACGGTAG  20
Template        2883988  ....................  2884007

Reverse primer  1        AGGCCTTCGGTAATGATCTTGT  22
Template        2884343  ......................  2884322
```

>CP041771.1 Pseudomonas aeruginosa strain A681 chromosome, complete genome

```
product length = 356
Forward primer  1       CTGAAGCTGGAGGACGGTAG  20
Template        469597  ....................  469616

Reverse primer  1       AGGCCTTCGGTAATGATCTTGT  22
Template        469952  ......................  469931
```

>CP041774.1 Pseudomonas aeruginosa strain 60503 chromosome, complete genome

```
product length = 356
Forward primer  1       CTGAAGCTGGAGGACGGTAG  20
Template        486851  ....................  486870

Reverse primer  1       AGGCCTTCGGTAATGATCTTGT  22
Template        487206  ......................  487185
```

>LR657304.1 Pseudomonas aeruginosa strain PAK genome assembly, chromosome: 1

```
product length = 356
Forward primer  1       CTGAAGCTGGAGGACGGTAG  20
Template        461221  ....................  461240

Reverse primer  1       AGGCCTTCGGTAATGATCTTGT  22
Template        461576  ......................  461555
```

>CP034244.1 Pseudomonas aeruginosa UCBPP-PA14 chromosome

```
product length = 356
Forward primer  1       CTGAAGCTGGAGGACGGTAG  20
Template        487403  ....................  487422

Reverse primer  1       AGGCCTTCGGTAATGATCTTGT  22
Template        487758  ......................  487737
```

>CP041013.1 Pseudomonas aeruginosa strain FDAARGOS_610 chromosome, complete genome

```
product length = 356
Forward primer  1        CTGAAGCTGGAGGACGGTAG  20
Template        6372990  ....................  6372971

Reverse primer  1        AGGCCTTCGGTAATGATCTTGT  22
Template        6372635  ......................  6372656
```

>CP041008.1 Pseudomonas aeruginosa strain FDAARGOS_767 chromosome, complete genome

```
product length = 356
Forward primer  1        CTGAAGCTGGAGGACGGTAG  20
Template        5251182  ....................  5251163

Reverse primer  1        AGGCCTTCGGTAATGATCTTGT  22
Template        5250827  ......................  5250848
```

>CP032569.2 Pseudomonas aeruginosa strain BA7823 chromosome, complete genome

```
product length = 356
Forward primer  1       CTGAAGCTGGAGGACGGTAG  20
Template        350642  ....................  350661

Reverse primer  1       AGGCCTTCGGTAATGATCTTGT  22
Template        350997  ......................  350976
```

>LR590474.1 Pseudomonas aeruginosa strain NCTC13618 genome assembly, chromosome: 1

```
product length = 356
Forward primer  1       CTGAAGCTGGAGGACGGTAG  20
Template        471518  ....................  471537

Reverse primer  1       AGGCCTTCGGTAATGATCTTGT  22
Template        471873  ......................  471852
```

>LR590473.1 Pseudomonas aeruginosa strain NCTC13359 genome assembly, chromosome: 1

```
product length = 356
Forward primer  1       CTGAAGCTGGAGGACGGTAG  20
Template        855615  ....................  855634

Reverse primer  1       AGGCCTTCGGTAATGATCTTGT  22
Template        855970  ......................  855949
```

>LR590472.1 Pseudomonas aeruginosa strain NCTC13620 genome assembly, chromosome: 1

```
product length = 357
Forward primer  1       CTGAAGCTGGAGGACGGTAG  20
Template        518000  ....................  518019

Reverse primer  1       AGGCCTTCGGTAATGATCTTGT  22
Template        518356  ......................  518335
```

>CP039749.1 Pseudomonas aeruginosa strain PRD-10 chromosome

```
product length = 356
Forward primer  1        CTGAAGCTGGAGGACGGTAG  20
Template        6536932  ....................  6536951

Reverse primer  1        AGGCCTTCGGTAATGATCTTGT  22
Template        6537287  ......................  6537266
```

>CP039293.1 Pseudomonas aeruginosa strain PABL048 chromosome, complete genome

```
product length = 356
Forward primer  1       CTGAAGCTGGAGGACGGTAG  20
Template        464466  ....................  464485

Reverse primer  1       AGGCCTTCGGTAATGATCTTGT  22
Template        464821  ......................  464800
```

>CP038661.1 Pseudomonas aeruginosa strain AJ D 2 chromosome

```
product length = 356
Forward primer  1       CTGAAGCTGGAGGACGGTAG  20
Template        475493  ....................  475512

Reverse primer  1       AGGCCTTCGGTAATGATCTTGT  22
Template        475848  ......................  475827
```

>CP037925.1 Pseudomonas aeruginosa strain AES1M chromosome, complete genome

```
product length = 356
Forward primer  1       CTGAAGCTGGAGGACGGTAG  20
Template        466539  ....................  466558

Reverse primer  1       AGGCCTTCGGTAATGATCTTGT  22
Template        466894  ......................  466873
```

>CP037926.1 Pseudomonas aeruginosa strain AES1R chromosome, complete genome

```
product length = 356
Forward primer  1       CTGAAGCTGGAGGACGGTAG  20
Template        466475  ....................  466494

Reverse primer  1       AGGCCTTCGGTAATGATCTTGT  22
Template        466830  ......................  466809
```

>CP028332.1 Pseudomonas aeruginosa strain PA-VAP-1 chromosome

```
product length = 356
Forward primer  1       CTGAAGCTGGAGGACGGTAG  20
Template        463190  ....................  463209

Reverse primer  1       AGGCCTTCGGTAATGATCTTGT  22
Template        463545  ......................  463524
```

>CP028331.1 Pseudomonas aeruginosa strain PA-VAP-2 chromosome

```
product length = 356
Forward primer  1        CTGAAGCTGGAGGACGGTAG  20
Template        5809471  ....................  5809452

Reverse primer  1        AGGCCTTCGGTAATGATCTTGT  22
Template        5809116  ......................  5809137
```

>CP028330.1 Pseudomonas aeruginosa strain PA-VAP-3 chromosome

```
product length = 356
Forward primer  1        CTGAAGCTGGAGGACGGTAG  20
Template        4878730  ....................  4878711

Reverse primer  1        AGGCCTTCGGTAATGATCTTGT  22
Template        4878375  ......................  4878396
```

>CP022478.1 Pseudomonas aeruginosa strain LW chromosome, complete genome

```
product length = 356
Forward primer  1        CTGAAGCTGGAGGACGGTAG  20
Template        5995189  ....................  5995208

Reverse primer  1        AGGCCTTCGGTAATGATCTTGT  22
Template        5995544  ......................  5995523
```

>CP034430.1 Pseudomonas aeruginosa strain GIMC5016:PA1840 chromosome

```
product length = 356
Forward primer  1       CTGAAGCTGGAGGACGGTAG  20
Template        615644  ....................  615663

Reverse primer  1       AGGCCTTCGGTAATGATCTTGT  22
Template        615999  ......................  615978
```

>CP034429.1 Pseudomonas aeruginosa strain GIMC5015:PAKB6, complete sequence

```
product length = 356
Forward primer  1       CTGAAGCTGGAGGACGGTAG  20
Template        472230  ....................  472249

Reverse primer  1       AGGCCTTCGGTAATGATCTTGT  22
Template        472585  ......................  472564
```

>LR134342.1 Pseudomonas aeruginosa strain NCTC10728 genome assembly, chromosome: 1

```
product length = 356
Forward primer  1        CTGAAGCTGGAGGACGGTAG  20
Template        3451419  ....................  3451438

Reverse primer  1        AGGCCTTCGGTAATGATCTTGT  22
Template        3451774  ......................  3451753
```

>LR134330.1 Pseudomonas aeruginosa strain NCTC13715 genome assembly, chromosome: 1

```
product length = 356
Forward primer  1        CTGAAGCTGGAGGACGGTAG  20
Template        2330939  ....................  2330958

Reverse primer  1        AGGCCTTCGGTAATGATCTTGT  22
Template        2331294  ......................  2331273
```

>LR134309.1 Pseudomonas aeruginosa strain NCTC12903 genome assembly, chromosome: 1

```
product length = 356
Forward primer  1       CTGAAGCTGGAGGACGGTAG  20
Template        482376  ....................  482395

Reverse primer  1       AGGCCTTCGGTAATGATCTTGT  22
Template        482731  ......................  482710
```

>LR134308.1 Pseudomonas aeruginosa strain NCTC11445 genome assembly, chromosome: 1

```
product length = 356
Forward primer  1        CTGAAGCTGGAGGACGGTAG  20
Template        5053566  ....................  5053547

Reverse primer  1        AGGCCTTCGGTAATGATCTTGT  22
Template        5053211  ......................  5053232
```

>LR134300.1 Pseudomonas fluorescens strain NCTC10783 genome assembly, chromosome: 1

```
product length = 356
Forward primer  1        CTGAAGCTGGAGGACGGTAG  20
Template        6130157  ....................  6130176

Reverse primer  1        AGGCCTTCGGTAATGATCTTGT  22
Template        6130512  ......................  6130491
```

>CP032541.1 Pseudomonas aeruginosa strain PGN5 chromosome

```
product length = 356
Forward primer  1       CTGAAGCTGGAGGACGGTAG  20
Template        472712  ....................  472731

Reverse primer  1       AGGCCTTCGGTAATGATCTTGT  22
Template        473067  ......................  473046
```

>CP032540.1 Pseudomonas aeruginosa strain PGN4 chromosome

```
product length = 356
Forward primer  1       CTGAAGCTGGAGGACGGTAG  20
Template        472707  ....................  472726

Reverse primer  1       AGGCCTTCGGTAATGATCTTGT  22
Template        473062  ......................  473041
```

>CP034434.1 Pseudomonas aeruginosa strain SP2230 chromosome, complete genome

```
product length = 356
Forward primer  1        CTGAAGCTGGAGGACGGTAG  20
Template        6532186  ....................  6532205

Reverse primer  1        AGGCCTTCGGTAATGATCTTGT  22
Template        6532541  ......................  6532520
```

>CP034435.1 Pseudomonas aeruginosa strain B14130 chromosome, complete genome

```
product length = 356
Forward primer  1        CTGAAGCTGGAGGACGGTAG  20
Template        5889753  ....................  5889734

Reverse primer  1        AGGCCTTCGGTAATGATCTTGT  22
Template        5889398  ......................  5889419
```

>CP034436.1 Pseudomonas aeruginosa strain B17932 chromosome, complete genome

```
product length = 356
Forward primer  1        CTGAAGCTGGAGGACGGTAG  20
Template        5756504  ....................  5756485

Reverse primer  1        AGGCCTTCGGTAATGATCTTGT  22
Template        5756149  ......................  5756170
```

>CP034409.1 Pseudomonas aeruginosa strain SP4527 chromosome, complete genome

```
product length = 356
Forward primer  1        CTGAAGCTGGAGGACGGTAG  20
Template        5824650  ....................  5824669

Reverse primer  1        AGGCCTTCGGTAATGATCTTGT  22
Template        5825005  ......................  5824984
```

>CP034369.1 Pseudomonas aeruginosa strain SP4371 chromosome, complete genome

```
product length = 356
Forward primer  1        CTGAAGCTGGAGGACGGTAG  20
Template        6718671  ....................  6718690

Reverse primer  1        AGGCCTTCGGTAATGATCTTGT  22
Template        6719026  ......................  6719005
```

>CP034368.1 Pseudomonas aeruginosa strain B41226 chromosome, complete genome

```
product length = 356
Forward primer  1       CTGAAGCTGGAGGACGGTAG  20
Template        179644  ....................  179663

Reverse primer  1       AGGCCTTCGGTAATGATCTTGT  22
Template        179999  ......................  179978
```

>CP034354.1 Pseudomonas aeruginosa strain IMP-13 chromosome, complete genome

```
product length = 356
Forward primer  1        CTGAAGCTGGAGGACGGTAG  20
Template        3570456  ....................  3570475

Reverse primer  1        AGGCCTTCGGTAATGATCTTGT  22
Template        3570811  ......................  3570790
```

>LR130537.1 Pseudomonas aeruginosa isolate paerg012 genome assembly, chromosome: 0

```
product length = 356
Forward primer  1       CTGAAGCTGGAGGACGGTAG  20
Template        476172  ....................  476191

Reverse primer  1       AGGCCTTCGGTAATGATCTTGT  22
Template        476527  ......................  476506
```

>LR130536.1 Pseudomonas aeruginosa isolate paerg010 genome assembly, chromosome: 0

```
product length = 356
Forward primer  1       CTGAAGCTGGAGGACGGTAG  20
Template        476192  ....................  476211

Reverse primer  1       AGGCCTTCGGTAATGATCTTGT  22
Template        476547  ......................  476526
```

>LR130535.1 Pseudomonas aeruginosa isolate paerg011 genome assembly, chromosome: 0

```
product length = 356
Forward primer  1       CTGAAGCTGGAGGACGGTAG  20
Template        476197  ....................  476216

Reverse primer  1       AGGCCTTCGGTAATGATCTTGT  22
Template        476552  ......................  476531
```

>LR130534.1 Pseudomonas aeruginosa isolate paerg005 genome assembly, chromosome: 0

```
product length = 356
Forward primer  1       CTGAAGCTGGAGGACGGTAG  20
Template        548787  ....................  548806

Reverse primer  1       AGGCCTTCGGTAATGATCTTGT  22
Template        549142  ......................  549121
```

>LR130533.1 Pseudomonas aeruginosa isolate paerg009 genome assembly, chromosome: 0

```
product length = 356
Forward primer  1        CTGAAGCTGGAGGACGGTAG  20
Template        3867796  ....................  3867815

Reverse primer  1        AGGCCTTCGGTAATGATCTTGT  22
Template        3868151  ......................  3868130
```

>LR130531.1 Pseudomonas aeruginosa isolate paerg004 genome assembly, chromosome: 0

```
product length = 356
Forward primer  1        CTGAAGCTGGAGGACGGTAG  20
Template        4227804  ....................  4227823

Reverse primer  1        AGGCCTTCGGTAATGATCTTGT  22
Template        4228159  ......................  4228138
```

>LR130530.1 Pseudomonas aeruginosa isolate paerg003 genome assembly, chromosome: 0

```
product length = 356
Forward primer  1       CTGAAGCTGGAGGACGGTAG  20
Template        476155  ....................  476174

Reverse primer  1       AGGCCTTCGGTAATGATCTTGT  22
Template        476510  ......................  476489
```

>LR130528.1 Pseudomonas aeruginosa isolate paerg000 genome assembly, chromosome: 0

```
product length = 356
Forward primer  1       CTGAAGCTGGAGGACGGTAG  20
Template        465901  ....................  465920

Reverse primer  1       AGGCCTTCGGTAATGATCTTGT  22
Template        466256  ......................  466235
```

>LR130527.1 Pseudomonas aeruginosa isolate paerg002 genome assembly, chromosome: 0

```
product length = 356
Forward primer  1        CTGAAGCTGGAGGACGGTAG  20
Template        3590690  ....................  3590709

Reverse primer  1        AGGCCTTCGGTAATGATCTTGT  22
Template        3591045  ......................  3591024
```

>CP033832.1 Pseudomonas aeruginosa strain FDAARGOS_505 chromosome, complete genome

```
product length = 356
Forward primer  1        CTGAAGCTGGAGGACGGTAG  20
Template        1971963  ....................  1971982

Reverse primer  1        AGGCCTTCGGTAATGATCTTGT  22
Template        1972318  ......................  1972297
```

>CP033835.1 Pseudomonas aeruginosa strain FDAARGOS_570 chromosome, complete genome

```
product length = 356
Forward primer  1        CTGAAGCTGGAGGACGGTAG  20
Template        6692457  ....................  6692438

Reverse primer  1        AGGCCTTCGGTAATGATCTTGT  22
Template        6692102  ......................  6692123
```

>CP033833.1 Pseudomonas aeruginosa strain FDAARGOS_571 chromosome, complete genome

```
product length = 356
Forward primer  1        CTGAAGCTGGAGGACGGTAG  20
Template        3740704  ....................  3740723

Reverse primer  1        AGGCCTTCGGTAATGATCTTGT  22
Template        3741059  ......................  3741038
```

>CP033843.1 Pseudomonas aeruginosa strain FDAARGOS_501 chromosome, complete genome

```
product length = 356
Forward primer  1        CTGAAGCTGGAGGACGGTAG  20
Template        2206683  ....................  2206664

Reverse primer  1        AGGCCTTCGGTAATGATCTTGT  22
Template        2206328  ......................  2206349
```

>CP033771.1 Pseudomonas aeruginosa strain FDAARGOS_532 chromosome, complete genome

```
product length = 356
Forward primer  1        CTGAAGCTGGAGGACGGTAG  20
Template        5303693  ....................  5303712

Reverse primer  1        AGGCCTTCGGTAATGATCTTGT  22
Template        5304048  ......................  5304027
```

>CP033684.1 Pseudomonas aeruginosa strain H26027 chromosome, complete genome

```
product length = 356
Forward primer  1       CTGAAGCTGGAGGACGGTAG  20
Template        517989  ....................  518008

Reverse primer  1       AGGCCTTCGGTAATGATCTTGT  22
Template        518344  ......................  518323
```

>CP033686.1 Pseudomonas aeruginosa strain H25883 chromosome, complete genome

```
product length = 356
Forward primer  1       CTGAAGCTGGAGGACGGTAG  20
Template        472569  ....................  472588

Reverse primer  1       AGGCCTTCGGTAATGATCTTGT  22
Template        472924  ......................  472903
```

>CP033685.1 Pseudomonas aeruginosa strain H26023 chromosome, complete genome

```
product length = 356
Forward primer  1       CTGAAGCTGGAGGACGGTAG  20
Template        486977  ....................  486996

Reverse primer  1       AGGCCTTCGGTAATGATCTTGT  22
Template        487332  ......................  487311
```

>CP029713.1 Pseudomonas aeruginosa strain BH9 chromosome

```
product length = 356
Forward primer  1       CTGAAGCTGGAGGACGGTAG  20
Template        465951  ....................  465970

Reverse primer  1       AGGCCTTCGGTAATGATCTTGT  22
Template        466306  ......................  466285
```

>CP033439.1 Pseudomonas aeruginosa strain SP4528 chromosome, complete genome

```
product length = 356
Forward primer  1        CTGAAGCTGGAGGACGGTAG  20
Template        6032830  ....................  6032811

Reverse primer  1        AGGCCTTCGGTAATGATCTTGT  22
Template        6032475  ......................  6032496
```

>CP033432.1 Pseudomonas aeruginosa strain BA15561 chromosome, complete genome

```
product length = 356
Forward primer  1        CTGAAGCTGGAGGACGGTAG  20
Template        4176598  ....................  4176579

Reverse primer  1        AGGCCTTCGGTAATGATCTTGT  22
Template        4176243  ......................  4176264
```

>CP033084.1 Pseudomonas aeruginosa strain PA-3 chromosome, complete genome

```
product length = 356
Forward primer  1        CTGAAGCTGGAGGACGGTAG  20
Template        4067392  ....................  4067373

Reverse primer  1        AGGCCTTCGGTAATGATCTTGT  22
Template        4067037  ......................  4067058
```

>CP030075.1 Pseudomonas aeruginosa strain 6762 chromosome

```
product length = 356
Forward primer  1        CTGAAGCTGGAGGACGGTAG  20
Template        2582630  ....................  2582649

Reverse primer  1        AGGCCTTCGGTAATGATCTTGT  22
Template        2582985  ......................  2582964
```

>CP032552.1 Pseudomonas aeruginosa strain PA34 chromosome, complete genome

```
product length = 356
Forward primer  1       CTGAAGCTGGAGGACGGTAG  20
Template        477417  ....................  477436

Reverse primer  1       AGGCCTTCGGTAATGATCTTGT  22
Template        477772  ......................  477751
```

>CP032761.1 Pseudomonas aeruginosa strain 268 chromosome, complete genome

```
product length = 356
Forward primer  1        CTGAAGCTGGAGGACGGTAG  20
Template        557938   ....................  557957

Reverse primer  1        AGGCCTTCGGTAATGATCTTGT  22
Template        558293   ......................  558272
```

>CP032257.1 Pseudomonas aeruginosa strain AR_0111 chromosome, complete genome

```
product length = 356
Forward primer  1         CTGAAGCTGGAGGACGGTAG  20
Template        3817784   ....................  3817803

Reverse primer  1         AGGCCTTCGGTAATGATCTTGT  22
Template        3818139   ......................  3818118
```

>CP031877.1 Pseudomonas aeruginosa strain WPB100 chromosome

```
product length = 356
Forward primer  1        CTGAAGCTGGAGGACGGTAG  20
Template        274380   ....................  274361

Reverse primer  1        AGGCCTTCGGTAATGATCTTGT  22
Template        274025   ......................  274046
```

>CP031876.1 Pseudomonas aeruginosa strain WPB101 chromosome

```
product length = 356
Forward primer  1        CTGAAGCTGGAGGACGGTAG  20
Template        242230   ....................  242211

Reverse primer  1        AGGCCTTCGGTAATGATCTTGT  22
Template        241875   ......................  241896
```

>CP031878.1 Pseudomonas aeruginosa strain WPB099 chromosome

```
product length = 356
Forward primer  1        CTGAAGCTGGAGGACGGTAG  20
Template        242230   ....................  242211

Reverse primer  1        AGGCCTTCGGTAATGATCTTGT  22
Template        241875   ......................  241896
```

>CP031879.1 Pseudomonas aeruginosa strain WPB098 chromosome

```
product length = 356
Forward primer  1         CTGAAGCTGGAGGACGGTAG  20
Template        4722846   ....................  4722827

Reverse primer  1         AGGCCTTCGGTAATGATCTTGT  22
Template        4722491   ......................  4722512
```

>CP029605.1 Pseudomonas aeruginosa strain 24Pae112 chromosome, complete genome

```
product length = 356
Forward primer  1       CTGAAGCTGGAGGACGGTAG  20
Template        510747  ....................  510766

Reverse primer  1       AGGCCTTCGGTAATGATCTTGT  22
Template        511102  ......................  511081
```

>CP031660.1 Pseudomonas aeruginosa strain PABL017 chromosome, complete genome

```
product length = 356
Forward primer  1       CTGAAGCTGGAGGACGGTAG  20
Template        470951  ....................  470970

Reverse primer  1       AGGCCTTCGGTAATGATCTTGT  22
Template        471306  ......................  471285
```

>CP031659.1 Pseudomonas aeruginosa strain PABL012 chromosome, complete genome

```
product length = 356
Forward primer  1       CTGAAGCTGGAGGACGGTAG  20
Template        475452  ....................  475471

Reverse primer  1       AGGCCTTCGGTAATGATCTTGT  22
Template        475807  ......................  475786
```

>CP035739.1 Pseudomonas aeruginosa strain 1334/14 chromosome, complete genome

```
product length = 356
Forward primer  1        CTGAAGCTGGAGGACGGTAG  20
Template        3612048  ....................  3612029

Reverse primer  1        AGGCCTTCGGTAATGATCTTGT  22
Template        3611693  ......................  3611714
```

>CP031449.2 Pseudomonas aeruginosa strain 97 chromosome, complete genome

```
product length = 356
Forward primer  1       CTGAAGCTGGAGGACGGTAG  20
Template        487827  ....................  487846

Reverse primer  1       AGGCCTTCGGTAATGATCTTGT  22
Template        488182  ......................  488161
```

>LS998783.1 Pseudomonas aeruginosa isolate 1 genome assembly, chromosome: 1

```
product length = 356
Forward primer  1       CTGAAGCTGGAGGACGGTAG  20
Template        530814  ....................  530833

Reverse primer  1       AGGCCTTCGGTAATGATCTTGT  22
Template        531169  ......................  531148
```

>CP032126.1 Pseudomonas aeruginosa strain PAO1161 chromosome, complete genome

```
product length = 356
Forward primer  1       CTGAAGCTGGAGGACGGTAG  20
Template        472712  ....................  472731

Reverse primer  1       AGGCCTTCGGTAATGATCTTGT  22
Template        473067  ......................  473046
```

>CP030912.1 Pseudomonas aeruginosa strain Y82 chromosome, complete genome

```
product length = 356
Forward primer  1       CTGAAGCTGGAGGACGGTAG  20
Template        534309  ....................  534328

Reverse primer  1       AGGCCTTCGGTAATGATCTTGT  22
Template        534664  ......................  534643
```

>CP030910.1 Pseudomonas aeruginosa strain Y31 chromosome, complete genome

```
product length = 356
Forward primer  1       CTGAAGCTGGAGGACGGTAG  20
Template        470322  ....................  470341

Reverse primer  1       AGGCCTTCGGTAATGATCTTGT  22
Template        470677  ......................  470656
```

>CP030861.1 Pseudomonas aeruginosa strain HS9 chromosome, complete genome

```
product length = 356
Forward primer  1        CTGAAGCTGGAGGACGGTAG  20
Template        2065477  ....................  2065458

Reverse primer  1        AGGCCTTCGGTAATGATCTTGT  22
Template        2065122  ......................  2065143
```

>CP030327.1 Pseudomonas aeruginosa strain AR_458 chromosome, complete genome

```
product length = 356
Forward primer  1        CTGAAGCTGGAGGACGGTAG  20
Template        2569241  ....................  2569222

Reverse primer  1        AGGCCTTCGGTAATGATCTTGT  22
Template        2568886  ......................  2568907
```

>CP030351.1 Pseudomonas aeruginosa strain AR_460 chromosome, complete genome

```
product length = 356
Forward primer  1        CTGAAGCTGGAGGACGGTAG  20
Template        4173018  ....................  4172999

Reverse primer  1        AGGCCTTCGGTAATGATCTTGT  22
Template        4172663  ......................  4172684
```

>CP030328.1 Pseudomonas aeruginosa strain AR_455 chromosome, complete genome

```
product length = 356
Forward primer  1        CTGAAGCTGGAGGACGGTAG  20
Template        1837799  ....................  1837818

Reverse primer  1        AGGCCTTCGGTAATGATCTTGT  22
Template        1838154  ......................  1838133
```

>CP029707.1 Pseudomonas aeruginosa strain K34-7 chromosome, complete genome

```
product length = 356
Forward primer  1        CTGAAGCTGGAGGACGGTAG  20
Template        6055480  ....................  6055499

Reverse primer  1        AGGCCTTCGGTAATGATCTTGT  22
Template        6055835  ......................  6055814
```

>CP029745.1 Pseudomonas aeruginosa strain AR_0110 chromosome, complete genome

```
product length = 356
Forward primer  1       CTGAAGCTGGAGGACGGTAG  20
Template        215695  ....................  215676

Reverse primer  1       AGGCCTTCGGTAATGATCTTGT  22
Template        215340  ......................  215361
```

>CP023255.1 Pseudomonas aeruginosa strain CCUG 70744 chromosome, complete genome

```
product length = 356
Forward primer  1        CTGAAGCTGGAGGACGGTAG  20
Template        5254681  ....................  5254700

Reverse primer  1        AGGCCTTCGGTAATGATCTTGT  22
Template        5255036  ......................  5255015
```

>CP029148.1 Pseudomonas aeruginosa strain AR_0440 chromosome

```
product length = 356
Forward primer  1        CTGAAGCTGGAGGACGGTAG  20
Template        6703929  ....................  6703948

Reverse primer  1        AGGCCTTCGGTAATGATCTTGT  22
Template        6704284  ......................  6704263
```

>CP029147.1 Pseudomonas aeruginosa strain AR_0443 chromosome

```
product length = 356
Forward primer  1        CTGAAGCTGGAGGACGGTAG  20
Template        4134813  ....................  4134832

Reverse primer  1        AGGCCTTCGGTAATGATCTTGT  22
Template        4135168  ......................  4135147
```

>CP029097.1 Pseudomonas aeruginosa strain AR439 chromosome, complete genome

```
product length = 356
Forward primer  1        CTGAAGCTGGAGGACGGTAG  20
Template        6816644  ....................  6816625

Reverse primer  1        AGGCCTTCGGTAATGATCTTGT  22
Template        6816289  ......................  6816310
```

>CP029090.1 Pseudomonas aeruginosa strain AR442 chromosome, complete genome

```
product length = 356
Forward primer  1        CTGAAGCTGGAGGACGGTAG  20
Template        6705161  ....................  6705180

Reverse primer  1        AGGCCTTCGGTAATGATCTTGT  22
Template        6705516  ......................  6705495
```

>CP029089.1 Pseudomonas aeruginosa strain AR444 chromosome, complete genome

```
product length = 356
Forward primer  1        CTGAAGCTGGAGGACGGTAG  20
Template        6212062  ....................  6212081

Reverse primer  1        AGGCCTTCGGTAATGATCTTGT  22
Template        6212417  ......................  6212396
```

>CP029088.1 Pseudomonas aeruginosa strain AR445 chromosome, complete genome

```
product length = 356
Forward primer  1       CTGAAGCTGGAGGACGGTAG  20
Template        526903  ....................  526922

Reverse primer  1       AGGCCTTCGGTAATGATCTTGT  22
Template        527258  ......................  527237
```

>CP028917.1 Pseudomonas aeruginosa strain JB2 chromosome, complete genome

```
product length = 356
Forward primer  1       CTGAAGCTGGAGGACGGTAG  20
Template        459448  ....................  459467

Reverse primer  1       AGGCCTTCGGTAATGATCTTGT  22
Template        459803  ......................  459782
```

>CP023316.1 Pseudomonas aeruginosa strain PPF-1 chromosome, complete genome

```
product length = 356
Forward primer  1       CTGAAGCTGGAGGACGGTAG  20
Template        536215  ....................  536234

Reverse primer  1       AGGCCTTCGGTAATGATCTTGT  22
Template        536570  ......................  536549
```

>CP028162.1 Pseudomonas aeruginosa strain MRSN12280 chromosome, complete genome

```
product length = 356
Forward primer  1        CTGAAGCTGGAGGACGGTAG  20
Template        512700   ....................  512719

Reverse primer  1        AGGCCTTCGGTAATGATCTTGT  22
Template        513055   ......................  513034
```

>CP027538.1 Pseudomonas aeruginosa strain AR_0095 chromosome, complete genome

```
product length = 356
Forward primer  1        CTGAAGCTGGAGGACGGTAG  20
Template        2567204  ....................  2567185

Reverse primer  1        AGGCCTTCGGTAATGATCTTGT  22
Template        2566849  ......................  2566870
```

>CP027166.1 Pseudomonas aeruginosa strain AR_0357 chromosome, complete genome

```
product length = 356
Forward primer  1        CTGAAGCTGGAGGACGGTAG  20
Template        1196689  ....................  1196670

Reverse primer  1        AGGCCTTCGGTAATGATCTTGT  22
Template        1196334  ......................  1196355
```

>CP027174.1 Pseudomonas aeruginosa strain AR_0230 chromosome, complete genome

```
product length = 356
Forward primer  1        CTGAAGCTGGAGGACGGTAG  20
Template        6066378  ....................  6066397

Reverse primer  1        AGGCCTTCGGTAATGATCTTGT  22
Template        6066733  ......................  6066712
```

>CP027172.1 Pseudomonas aeruginosa strain AR_0353 chromosome, complete genome

```
product length = 356
Forward primer  1        CTGAAGCTGGAGGACGGTAG  20
Template        6806933  ....................  6806914

Reverse primer  1        AGGCCTTCGGTAATGATCTTGT  22
Template        6806578  ......................  6806599
```

>CP027171.1 Pseudomonas aeruginosa strain AR_0354 chromosome, complete genome

```
product length = 356
Forward primer  1        CTGAAGCTGGAGGACGGTAG  20
Template        5135508  ....................  5135527

Reverse primer  1        AGGCCTTCGGTAATGATCTTGT  22
Template        5135863  ......................  5135842
```

>CP027165.1 Pseudomonas aeruginosa strain AR_0360 chromosome, complete genome

```
product length = 356
Forward primer  1        CTGAAGCTGGAGGACGGTAG  20
Template        5702373  ....................  5702392

Reverse primer  1        AGGCCTTCGGTAATGATCTTGT  22
Template        5702728  ......................  5702707
```

>CP026680.1 Pseudomonas aeruginosa strain F5677 chromosome, complete genome

```
product length = 356
Forward primer  1       CTGAAGCTGGAGGACGGTAG  20
Template        476267  ....................  476286

Reverse primer  1       AGGCCTTCGGTAATGATCTTGT  22
Template        476622  ......................  476601
```

>LT969520.1 Pseudomonas aeruginosa isolate RW109 genome assembly, chromosome: Main_chromosome

```
product length = 356
Forward primer  1       CTGAAGCTGGAGGACGGTAG  20
Template        485676  ....................  485695

Reverse primer  1       AGGCCTTCGGTAATGATCTTGT  22
Template        486031  ......................  486010
```

>CP025229.1 Pseudomonas sp. AK6U chromosome, complete genome

```
product length = 356
Forward primer  1        CTGAAGCTGGAGGACGGTAG  20
Template        4537740  ....................  4537721

Reverse primer  1        AGGCCTTCGGTAATGATCTTGT  22
Template        4537385  ......................  4537406
```

>CP025051.1 Pseudomonas aeruginosa strain PB353 chromosome, complete genome

```
product length = 356
Forward primer  1       CTGAAGCTGGAGGACGGTAG  20
Template        468866  ....................  468885

Reverse primer  1       AGGCCTTCGGTAATGATCTTGT  22
Template        469221  ......................  469200
```

>CP025050.1 Pseudomonas aeruginosa strain PB368 chromosome, complete genome

```
product length = 356
Forward primer  1       CTGAAGCTGGAGGACGGTAG  20
Template        355677  ....................  355658

Reverse primer  1       AGGCCTTCGGTAATGATCTTGT  22
```

```
Template        355322  ......................  355343
```

>CP025049.1 Pseudomonas aeruginosa strain PB369 chromosome, complete genome

```
product length = 356
Forward primer  1       CTGAAGCTGGAGGACGGTAG  20
Template        457339  ....................  457358

Reverse primer  1       AGGCCTTCGGTAATGATCTTGT  22
Template        457694  ......................  457673
```

>CP025053.1 Pseudomonas aeruginosa strain PB354 chromosome, complete genome

```
product length = 356
Forward primer  1       CTGAAGCTGGAGGACGGTAG  20
Template        468866  ....................  468885

Reverse primer  1       AGGCCTTCGGTAATGATCTTGT  22
Template        469221  ......................  469200
```

>CP024477.1 Pseudomonas aeruginosa strain 12939 chromosome, complete genome

```
product length = 356
Forward primer  1       CTGAAGCTGGAGGACGGTAG  20
Template        477118  ....................  477137

Reverse primer  1       AGGCCTTCGGTAATGATCTTGT  22
Template        477473  ......................  477452
```

>CP017306.1 Pseudomonas aeruginosa strain PA_150577 chromosome, complete genome

```
product length = 356
Forward primer  1       CTGAAGCTGGAGGACGGTAG  20
Template        466518  ....................  466537

Reverse primer  1       AGGCCTTCGGTAATGATCTTGT  22
Template        466873  ......................  466852
```

>CP022526.1 Pseudomonas aeruginosa strain Ocean-1155, complete genome

```
product length = 356
Forward primer  1        CTGAAGCTGGAGGACGGTAG  20
Template        4227034  ....................  4227053

Reverse primer  1        AGGCCTTCGGTAATGATCTTGT  22
Template        4227389  ......................  4227368
```

>CP022525.1 Pseudomonas aeruginosa strain Ocean-1175, complete genome

```
product length = 356
Forward primer  1        CTGAAGCTGGAGGACGGTAG  20
Template        1387557  ....................  1387538
```

```
Reverse primer  1        AGGCCTTCGGTAATGATCTTGT  22
Template        1387202  ......................  1387223
```

>CP019338.1 Pseudomonas aeruginosa strain L10, complete genome

```
product length = 356
Forward primer  1       CTGAAGCTGGAGGACGGTAG  20
Template        486940  ....................  486959

Reverse primer  1       AGGCCTTCGGTAATGATCTTGT  22
Template        487295  ......................  487274
```

>CP017293.1 Pseudomonas aeruginosa strain PA83, complete genome

```
product length = 356
Forward primer  1       CTGAAGCTGGAGGACGGTAG  20
Template        514580  ....................  514599

Reverse primer  1       AGGCCTTCGGTAATGATCTTGT  22
Template        514935  ......................  514914
```

>CP022001.1 Pseudomonas aeruginosa strain Pa1207, complete genome

```
product length = 356
Forward primer  1       CTGAAGCTGGAGGACGGTAG  20
Template        485297  ....................  485316

Reverse primer  1       AGGCCTTCGGTAATGATCTTGT  22
Template        485652  ......................  485631
```

>CP022000.1 Pseudomonas aeruginosa strain Pa127, complete genome

```
product length = 356
Forward primer  1       CTGAAGCTGGAGGACGGTAG  20
Template        606741  ....................  606760

Reverse primer  1       AGGCCTTCGGTAATGATCTTGT  22
Template        607096  ......................  607075
```

>CP021999.1 Pseudomonas aeruginosa strain Pa84, complete genome

```
product length = 356
Forward primer  1       CTGAAGCTGGAGGACGGTAG  20
Template        491255  ....................  491274

Reverse primer  1       AGGCCTTCGGTAATGATCTTGT  22
Template        491610  ......................  491589
```

>LT883143.1 Pseudomonas aeruginosa C-NN2 isolate early isolate NN2 (clone C) genome assembly, chromosome: I

```
product length = 356
Forward primer  1       CTGAAGCTGGAGGACGGTAG  20
```

```
Template        518478  ....................  518497

Reverse primer  1       AGGCCTTCGGTAATGATCTTGT  22
Template        518833  ......................  518812
```

>CP021774.1 Pseudomonas aeruginosa strain Pa124, complete genome

```
product length = 356
Forward primer  1       CTGAAGCTGGAGGACGGTAG  20
Template        606998  ....................  607017

Reverse primer  1       AGGCCTTCGGTAATGATCTTGT  22
Template        607353  ......................  607332
```

>CP021775.1 Pseudomonas aeruginosa strain Pa58, complete genome

```
product length = 356
Forward primer  1       CTGAAGCTGGAGGACGGTAG  20
Template        549224  ....................  549243

Reverse primer  1       AGGCCTTCGGTAATGATCTTGT  22
Template        549579  ......................  549558
```

>CP015650.1 Pseudomonas aeruginosa strain Pb18 genome

```
product length = 356
Forward primer  1        CTGAAGCTGGAGGACGGTAG  20
Template        4895953  ....................  4895972

Reverse primer  1        AGGCCTTCGGTAATGATCTTGT  22
Template        4896308  ......................  4896287
```

>CP015649.1 Pseudomonas aeruginosa strain M28A1 genome

```
product length = 356
Forward primer  1        CTGAAGCTGGAGGACGGTAG  20
Template        3774123  ....................  3774142

Reverse primer  1        AGGCCTTCGGTAATGATCTTGT  22
Template        3774478  ......................  3774457
```

>CP015648.1 Pseudomonas aeruginosa strain M8A4 genome

```
product length = 356
Forward primer  1        CTGAAGCTGGAGGACGGTAG  20
Template        5926297  ....................  5926278

Reverse primer  1        AGGCCTTCGGTAATGATCTTGT  22
Template        5925942  ......................  5925963
```

>CP015647.1 Pseudomonas aeruginosa strain M8A1 genome

```
product length = 356
```

```
Forward primer  1       CTGAAGCTGGAGGACGGTAG  20
Template        412866  ....................  412885

Reverse primer  1       AGGCCTTCGGTAATGATCTTGT  22
Template        413221  ......................  413200
```

>CP020704.1 Pseudomonas aeruginosa strain PASGNDM699, complete genome

```
product length = 356
Forward primer  1       CTGAAGCTGGAGGACGGTAG  20
Template        478393  ....................  478412

Reverse primer  1       AGGCCTTCGGTAATGATCTTGT  22
Template        478748  ......................  478727
```

>CP020703.1 Pseudomonas aeruginosa strain PASGNDM345, complete genome

```
product length = 356
Forward primer  1       CTGAAGCTGGAGGACGGTAG  20
Template        478391  ....................  478410

Reverse primer  1       AGGCCTTCGGTAATGATCTTGT  22
Template        478746  ......................  478725
```

>CP008858.2 Pseudomonas aeruginosa strain F63912 chromosome, complete genome

```
product length = 356
Forward primer  1       CTGAAGCTGGAGGACGGTAG  20
Template        467335  ....................  467354

Reverse primer  1       AGGCCTTCGGTAATGATCTTGT  22
Template        467690  ......................  467669
```

>CP020659.1 Pseudomonas aeruginosa PAK chromosome, complete genome

```
product length = 356
Forward primer  1        CTGAAGCTGGAGGACGGTAG  20
Template        6038436  ....................  6038455

Reverse primer  1        AGGCCTTCGGTAATGATCTTGT  22
Template        6038791  ......................  6038770
```

>CP008872.2 Pseudomonas aeruginosa strain X78812 chromosome, complete genome

```
product length = 356
Forward primer  1       CTGAAGCTGGAGGACGGTAG  20
Template        474381  ....................  474400

Reverse primer  1       AGGCCTTCGGTAATGATCTTGT  22
Template        474736  ......................  474715
```

>CP008871.2 Pseudomonas aeruginosa strain W45909 chromosome, complete genome

```
product length = 356
Forward primer  1       CTGAAGCTGGAGGACGGTAG  20
Template        471942  ....................  471961

Reverse primer  1       AGGCCTTCGGTAATGATCTTGT  22
Template        472297  ......................  472276
```

>CP008870.2 Pseudomonas aeruginosa strain W36662 chromosome, complete genome

```
product length = 356
Forward primer  1       CTGAAGCTGGAGGACGGTAG  20
Template        579845  ....................  579864

Reverse primer  1       AGGCCTTCGGTAATGATCTTGT  22
Template        580200  ......................  580179
```

>CP008869.2 Pseudomonas aeruginosa strain W16407 chromosome, complete genome

```
product length = 356
Forward primer  1       CTGAAGCTGGAGGACGGTAG  20
Template        468026  ....................  468045

Reverse primer  1       AGGCCTTCGGTAATGATCTTGT  22
Template        468381  ......................  468360
```

>CP008866.2 Pseudomonas aeruginosa strain T38079 chromosome, complete genome

```
product length = 356
Forward primer  1       CTGAAGCTGGAGGACGGTAG  20
Template        485310  ....................  485329

Reverse primer  1       AGGCCTTCGGTAATGATCTTGT  22
Template        485665  ......................  485644
```

>CP008865.2 Pseudomonas aeruginosa strain S86968 chromosome, complete genome

```
product length = 356
Forward primer  1       CTGAAGCTGGAGGACGGTAG  20
Template        484083  ....................  484102

Reverse primer  1       AGGCCTTCGGTAATGATCTTGT  22
Template        484438  ......................  484417
```

>CP008864.2 Pseudomonas aeruginosa strain W60856 chromosome, complete genome

```
product length = 356
Forward primer  1       CTGAAGCTGGAGGACGGTAG  20
Template        482240  ....................  482259

Reverse primer  1       AGGCCTTCGGTAATGATCTTGT  22
Template        482595  ......................  482574
```

>CP008862.2 Pseudomonas aeruginosa strain M1608 chromosome, complete genome

```
product length = 356
Forward primer  1       CTGAAGCTGGAGGACGGTAG  20
Template        525598  ....................  525617

Reverse primer  1       AGGCCTTCGGTAATGATCTTGT  22
Template        525953  ......................  525932
```

>CP008860.2 Pseudomonas aeruginosa strain H27930 chromosome, complete genome

```
product length = 356
Forward primer  1       CTGAAGCTGGAGGACGGTAG  20
Template        483174  ....................  483193

Reverse primer  1       AGGCCTTCGGTAATGATCTTGT  22
Template        483529  ......................  483508
```

>CP008859.2 Pseudomonas aeruginosa strain H5708 chromosome, complete genome

```
product length = 356
Forward primer  1       CTGAAGCTGGAGGACGGTAG  20
Template        507262  ....................  507281

Reverse primer  1       AGGCCTTCGGTAATGATCTTGT  22
Template        507617  ......................  507596
```

>CP008856.2 Pseudomonas aeruginosa strain F23197 chromosome, complete genome

```
product length = 356
Forward primer  1       CTGAAGCTGGAGGACGGTAG  20
Template        476501  ....................  476520

Reverse primer  1       AGGCCTTCGGTAATGATCTTGT  22
Template        476856  ......................  476835
```

>CP020603.1 Pseudomonas aeruginosa strain E6130952, complete genome

```
product length = 356
Forward primer  1       CTGAAGCTGGAGGACGGTAG  20
Template        473606  ....................  473625

Reverse primer  1       AGGCCTTCGGTAATGATCTTGT  22
Template        473961  ......................  473940
```

>CP016955.1 Pseudomonas aeruginosa strain RIVM-EMC2982, complete genome

```
product length = 356
Forward primer  1        CTGAAGCTGGAGGACGGTAG  20
Template        6825029  ....................  6825010

Reverse primer  1        AGGCCTTCGGTAATGATCTTGT  22
Template        6824674  ......................  6824695
```

>CP014866.1 Pseudomonas aeruginosa strain PA_154197 chromosome, complete genome

```
product length = 356
Forward primer  1       CTGAAGCTGGAGGACGGTAG  20
Template        460826  ....................  460845

Reverse primer  1       AGGCCTTCGGTAATGATCTTGT  22
Template        461181  ......................  461160
```

>LT673656.1 Pseudomonas aeruginosa isolate PcyII-10 genome assembly, chromosome: PcyII-10

```
product length = 356
Forward primer  1       CTGAAGCTGGAGGACGGTAG  20
Template        465113  ....................  465132

Reverse primer  1       AGGCCTTCGGTAATGATCTTGT  22
Template        465468  ......................  465447
```

>CP013479.1 Pseudomonas aeruginosa strain NHmuc chromosome, complete genome

```
product length = 356
Forward primer  1       CTGAAGCTGGAGGACGGTAG  20
Template        478382  ....................  478401

Reverse primer  1       AGGCCTTCGGTAATGATCTTGT  22
Template        478737  ......................  478716
```

>CP013478.1 Pseudomonas aeruginosa strain SCVJan chromosome, complete genome

```
product length = 356
Forward primer  1       CTGAAGCTGGAGGACGGTAG  20
Template        478382  ....................  478401

Reverse primer  1       AGGCCTTCGGTAATGATCTTGT  22
Template        478737  ......................  478716
```

>CP013477.1 Pseudomonas aeruginosa strain SCVFeb chromosome, complete genome

```
product length = 356
Forward primer  1       CTGAAGCTGGAGGACGGTAG  20
Template        478382  ....................  478401

Reverse primer  1       AGGCCTTCGGTAATGATCTTGT  22
Template        478737  ......................  478716
```

>CP013113.1 Pseudomonas aeruginosa strain PAER4_119 chromosome, complete genome

```
product length = 356
Forward primer  1       CTGAAGCTGGAGGACGGTAG  20
Template        474281  ....................  474300

Reverse primer  1       AGGCCTTCGGTAATGATCTTGT  22
Template        474636  ......................  474615
```

>CP017969.1 Pseudomonas aeruginosa isolate B10W chromosome, complete genome

```
product length = 356
Forward primer  1       CTGAAGCTGGAGGACGGTAG  20
Template        222024  ....................  222043

Reverse primer  1       AGGCCTTCGGTAATGATCTTGT  22
Template        222379  ......................  222358
```

>CP017353.1 Pseudomonas aeruginosa strain FA-HZ1 chromosome, complete genome

```
product length = 356
Forward primer  1        CTGAAGCTGGAGGACGGTAG  20
Template        3169367  ....................  3169386

Reverse primer  1        AGGCCTTCGGTAATGATCTTGT  22
Template        3169722  ......................  3169701
```

>CP017149.1 Pseudomonas aeruginosa strain ATCC 15692, complete genome

```
product length = 356
Forward primer  1       CTGAAGCTGGAGGACGGTAG  20
Template        472235  ....................  472254

Reverse primer  1       AGGCCTTCGGTAATGATCTTGT  22
Template        472590  ......................  472569
```

>CP012582.1 Pseudomonas aeruginosa strain PA_D21, complete genome

```
product length = 356
Forward primer  1       CTGAAGCTGGAGGACGGTAG  20
Template        472785  ....................  472804

Reverse primer  1       AGGCCTTCGGTAATGATCTTGT  22
Template        473140  ......................  473119
```

>CP012579.1 Pseudomonas aeruginosa strain PA_D5, complete genome

```
product length = 356
Forward primer  1       CTGAAGCTGGAGGACGGTAG  20
Template        472785  ....................  472804

Reverse primer  1       AGGCCTTCGGTAATGATCTTGT  22
Template        473140  ......................  473119
```

>CP017099.1 Pseudomonas aeruginosa strain DN1, complete genome

```
product length = 356
Forward primer  1       CTGAAGCTGGAGGACGGTAG  20
Template        477631  ....................  477650

Reverse primer  1       AGGCCTTCGGTAATGATCTTGT  22
Template        477986  ......................  477965
```

>CP012584.1 Pseudomonas aeruginosa strain PA_D25, complete genome

```
product length = 356
Forward primer  1       CTGAAGCTGGAGGACGGTAG  20
Template        472783  ....................  472802

Reverse primer  1       AGGCCTTCGGTAATGATCTTGT  22
Template        473138  ......................  473117
```

>CP012583.1 Pseudomonas aeruginosa strain PA_D22, complete genome

```
product length = 356
Forward primer  1       CTGAAGCTGGAGGACGGTAG  20
Template        472785  ....................  472804

Reverse primer  1       AGGCCTTCGGTAATGATCTTGT  22
Template        473140  ......................  473119
```

>CP012581.1 Pseudomonas aeruginosa strain PA_D16, complete genome

```
product length = 356
Forward primer  1       CTGAAGCTGGAGGACGGTAG  20
Template        472785  ....................  472804

Reverse primer  1       AGGCCTTCGGTAATGATCTTGT  22
Template        473140  ......................  473119
```

>CP012580.1 Pseudomonas aeruginosa strain PA_D9, complete genome

```
product length = 356
Forward primer  1       CTGAAGCTGGAGGACGGTAG  20
Template        472785  ....................  472804

Reverse primer  1       AGGCCTTCGGTAATGATCTTGT  22
Template        473140  ......................  473119
```

>CP012578.1 Pseudomonas aeruginosa strain PA_D2, complete genome

```
product length = 356
Forward primer  1       CTGAAGCTGGAGGACGGTAG  20
Template        472785  ....................  472804

Reverse primer  1       AGGCCTTCGGTAATGATCTTGT  22
Template        473140  ......................  473119
```

>CP012585.1 Pseudomonas aeruginosa strain PA_D1, complete genome

```
product length = 356
Forward primer  1       CTGAAGCTGGAGGACGGTAG  20
Template        472785  ....................  472804

Reverse primer  1       AGGCCTTCGGTAATGATCTTGT  22
Template        473140  ......................  473119
```

>LT608330.1 Pseudomonas aeruginosa isolate PA14Or_reads genome assembly, chromosome: PA14OR

```
product length = 356
Forward primer  1       CTGAAGCTGGAGGACGGTAG  20
Template        487465  ....................  487484

Reverse primer  1       AGGCCTTCGGTAATGATCTTGT  22
Template        487820  ......................  487799
```

>CP011857.1 Pseudomonas aeruginosa strain ATCC 27853, complete genome

```
product length = 356
Forward primer  1       CTGAAGCTGGAGGACGGTAG  20
Template        482374  ....................  482393

Reverse primer  1       AGGCCTTCGGTAATGATCTTGT  22
Template        482729  ......................  482708
```

>CP016214.1 Pseudomonas aeruginosa strain PA121617, complete genome

```
product length = 356
Forward primer  1        CTGAAGCTGGAGGACGGTAG  20
Template        3686887  ....................  3686906

Reverse primer  1        AGGCCTTCGGTAATGATCTTGT  22
Template        3687242  ......................  3687221
```

>CP015877.1 Pseudomonas aeruginosa SJTD-1 chromosome, complete genome

```
product length = 356
Forward primer  1       CTGAAGCTGGAGGACGGTAG  20
Template        550713  ....................  550694

Reverse primer  1       AGGCCTTCGGTAATGATCTTGT  22
Template        550358  ......................  550379
```

>CP015117.1 Pseudomonas aeruginosa strain ATCC 27853 chromosome, complete genome

```
product length = 356
Forward primer  1        CTGAAGCTGGAGGACGGTAG  20
Template        4073922  ....................  4073941

Reverse primer  1        AGGCCTTCGGTAATGATCTTGT  22
Template        4074277  ......................  4074256
```

>CP014948.1 Pseudomonas aeruginosa strain N17-1, complete genome

```
product length = 356
Forward primer  1       CTGAAGCTGGAGGACGGTAG  20
Template        466114  ....................  466133

Reverse primer  1       AGGCCTTCGGTAATGATCTTGT  22
```

```
Template        466469  ......................  466448
```

>CP014210.1 Pseudomonas aeruginosa strain KU, partial genome

```
product length = 356
Forward primer  1       CTGAAGCTGGAGGACGGTAG  20
Template        487465  ....................  487484

Reverse primer  1       AGGCCTTCGGTAATGATCTTGT  22
Template        487820  ......................  487799
```

>OX638701.1 Pseudomonas aeruginosa strain 4782MK genome assembly, chromosome: 4782

```
product length = 356
Forward primer  1        CTGAAGCTGGAGGACGGTAG  20
Template        1748220  ....................  1748239

Reverse primer  1        AGGCCTTCGGTAATGATCTTGT  22
Template        1748575  ......................  1748554
```

>OX638610.1 Pseudomonas aeruginosa strain 3541 genome assembly, chromosome: 3541

```
product length = 356
Forward primer  1       CTGAAGCTGGAGGACGGTAG  20
Template        231204  ....................  231185

Reverse primer  1       AGGCCTTCGGTAATGATCTTGT  22
Template        230849  ......................  230870
```

>OX638564.1 Pseudomonas aeruginosa strain 3796A genome assembly, chromosome: 3796A

```
product length = 356
Forward primer  1       CTGAAGCTGGAGGACGGTAG  20
Template        478168  ....................  478187

Reverse primer  1       AGGCCTTCGGTAATGATCTTGT  22
Template        478523  ......................  478502
```

>CP008873.1 Pseudomonas aeruginosa strain F9670 chromosome, complete genome

```
product length = 356
Forward primer  1       CTGAAGCTGGAGGACGGTAG  20
Template        898420  ....................  898401

Reverse primer  1       AGGCCTTCGGTAATGATCTTGT  22
Template        898065  ......................  898086
```

>CP013993.1 Pseudomonas aeruginosa DHS01 chromosome, complete genome

```
product length = 356
Forward primer  1       CTGAAGCTGGAGGACGGTAG  20
Template        465516  ....................  465535
```

```
Reverse primer  1       AGGCCTTCGGTAATGATCTTGT  22
Template        465871  ......................  465850
```

>CP013989.1 Pseudomonas aeruginosa strain USDA-ARS-USMARC-41639 chromosome, complete genome

```
product length = 356
Forward primer  1       CTGAAGCTGGAGGACGGTAG  20
Template        495393  ....................  495412

Reverse primer  1       AGGCCTTCGGTAATGATCTTGT  22
Template        495748  ......................  495727
```

>CP008868.1 Pseudomonas aeruginosa strain T63266 chromosome, complete genome

```
product length = 356
Forward primer  1        CTGAAGCTGGAGGACGGTAG  20
Template        2130133  ....................  2130152

Reverse primer  1        AGGCCTTCGGTAATGATCTTGT  22
Template        2130488  ......................  2130467
```

>CP008867.1 Pseudomonas aeruginosa strain T52373 chromosome, complete genome

```
product length = 356
Forward primer  1        CTGAAGCTGGAGGACGGTAG  20
Template        2961691  ....................  2961710

Reverse primer  1        AGGCCTTCGGTAATGATCTTGT  22
Template        2962046  ......................  2962025
```

>CP008863.1 Pseudomonas aeruginosa strain M37351 chromosome, complete genome

```
product length = 356
Forward primer  1        CTGAAGCTGGAGGACGGTAG  20
Template        4077534  ....................  4077553

Reverse primer  1        AGGCCTTCGGTAATGATCTTGT  22
Template        4077889  ......................  4077868
```

>CP008861.1 Pseudomonas aeruginosa strain H47921 chromosome, complete genome

```
product length = 356
Forward primer  1        CTGAAGCTGGAGGACGGTAG  20
Template        3256040  ....................  3256021

Reverse primer  1        AGGCCTTCGGTAATGATCTTGT  22
Template        3255685  ......................  3255706
```

>CP008857.1 Pseudomonas aeruginosa strain F30658 chromosome, complete genome

```
product length = 356
Forward primer  1        CTGAAGCTGGAGGACGGTAG  20
```

```
Template        5150980  ....................  5150999

Reverse primer  1        AGGCCTTCGGTAATGATCTTGT  22
Template        5151335  ......................  5151314
```

>CP012901.1 Pseudomonas aeruginosa strain N15-01092 chromosome, complete genome

```
product length = 356
Forward primer  1        CTGAAGCTGGAGGACGGTAG  20
Template        6432003  ....................  6431984

Reverse primer  1        AGGCCTTCGGTAATGATCTTGT  22
Template        6431648  ......................  6431669
```

>CP013696.1 Pseudomonas aeruginosa strain 12-4-4(59) chromosome, complete genome

```
product length = 356
Forward primer  1        CTGAAGCTGGAGGACGGTAG  20
Template        1979122  ....................  1979141

Reverse primer  1        AGGCCTTCGGTAATGATCTTGT  22
Template        1979477  ......................  1979456
```

>CP124672.1 Pseudomonas aeruginosa strain 2022CK-00451 chromosome, complete genome

```
product length = 356
Forward primer  1        CTGAAGCTGGAGGACGGTAG  20
Template        2155600  ....................  2155619

Reverse primer  1        AGGCCTTCGGTAATGATCTTGT  22
Template        2155955  ......................  2155934
```

>CP125367.1 Pseudomonas aeruginosa strain ZY1710 chromosome, complete genome

```
product length = 356
Forward primer  1       CTGAAGCTGGAGGACGGTAG  20
Template        504446  ....................  504465

Reverse primer  1       AGGCCTTCGGTAATGATCTTGT  22
Template        504801  ......................  504780
```

>CP125365.1 Pseudomonas aeruginosa strain ZY36 chromosome, complete genome

```
product length = 356
Forward primer  1       CTGAAGCTGGAGGACGGTAG  20
Template        504447  ....................  504466

Reverse primer  1       AGGCCTTCGGTAATGATCTTGT  22
Template        504802  ......................  504781
```

>CP125363.1 Pseudomonas aeruginosa strain ZY156 chromosome, complete genome

```
product length = 356
```

```
Forward primer  1       CTGAAGCTGGAGGACGGTAG  20
Template        504447  ....................  504466

Reverse primer  1       AGGCCTTCGGTAATGATCTTGT  22
Template        504802  ......................  504781
```

>CP125361.1 Pseudomonas aeruginosa strain ZY94 chromosome, complete genome

```
product length = 356
Forward primer  1       CTGAAGCTGGAGGACGGTAG  20
Template        504441  ....................  504460

Reverse primer  1       AGGCCTTCGGTAATGATCTTGT  22
Template        504796  ......................  504775
```

>CP125288.1 Pseudomonas aeruginosa strain SF416 chromosome, complete genome

```
product length = 356
Forward primer  1       CTGAAGCTGGAGGACGGTAG  20
Template        490588  ....................  490607

Reverse primer  1       AGGCCTTCGGTAATGATCTTGT  22
Template        490943  ......................  490922
```

>CP013144.1 Pseudomonas aeruginosa strain Cu1510 chromosome, complete genome

```
product length = 356
Forward primer  1        CTGAAGCTGGAGGACGGTAG  20
Template        4306258  ....................  4306277

Reverse primer  1        AGGCCTTCGGTAATGATCTTGT  22
Template        4306613  ......................  4306592
```

>AP017302.1 Pseudomonas aeruginosa DNA, complete genome, strain: IOMTU 133

```
product length = 356
Forward primer  1       CTGAAGCTGGAGGACGGTAG  20
Template        607875  ....................  607894

Reverse primer  1       AGGCCTTCGGTAATGATCTTGT  22
Template        608230  ......................  608209
```

>CP013245.1 Pseudomonas aeruginosa strain VA-134 chromosome, complete genome

```
product length = 356
Forward primer  1        CTGAAGCTGGAGGACGGTAG  20
Template        1746747  ....................  1746728

Reverse primer  1        AGGCCTTCGGTAATGATCTTGT  22
Template        1746392  ......................  1746413
```

>LN870292.1 Pseudomonas aeruginosa DK1 genome assembly Pseudomonas aeruginosa DK1 substr. NH57388A, chromosome : I

```
product length = 356
Forward primer  1       CTGAAGCTGGAGGACGGTAG  20
Template        478864  ....................  478883

Reverse primer  1       AGGCCTTCGGTAATGATCTTGT  22
Template        479219  ......................  479198
```

>CP012679.1 Pseudomonas aeruginosa strain PA1RG chromosome, complete genome

```
product length = 356
Forward primer  1       CTGAAGCTGGAGGACGGTAG  20
Template        470171  ....................  470190

Reverse primer  1       AGGCCTTCGGTAATGATCTTGT  22
Template        470526  ......................  470505
```

>CP004054.2 Pseudomonas aeruginosa PA1, complete genome

```
product length = 356
Forward primer  1       CTGAAGCTGGAGGACGGTAG  20
Template        470171  ....................  470190

Reverse primer  1       AGGCCTTCGGTAATGATCTTGT  22
Template        470526  ......................  470505
```

>LN871187.1 Pseudomonas aeruginosa genome assembly PAO1OR, chromosome : I

```
product length = 356
Forward primer  1       CTGAAGCTGGAGGACGGTAG  20
Template        472718  ....................  472737

Reverse primer  1       AGGCCTTCGGTAATGATCTTGT  22
Template        473073  ......................  473052
```

>AP014839.2 Pseudomonas aeruginosa DNA, complete genome, strain: 8380

```
product length = 356
Forward primer  1       CTGAAGCTGGAGGACGGTAG  20
Template        468024  ....................  468043

Reverse primer  1       AGGCCTTCGGTAATGATCTTGT  22
Template        468379  ......................  468358
```

>CP012066.1 Pseudomonas aeruginosa strain F9676, complete genome

```
product length = 356
Forward primer  1        CTGAAGCTGGAGGACGGTAG  20
Template        5464883  ....................  5464864

Reverse primer  1        AGGCCTTCGGTAATGATCTTGT  22
Template        5464528  ......................  5464549
```

>CP012001.1 Pseudomonas aeruginosa DSM 50071, complete genome

```
product length = 356
Forward primer  1       CTGAAGCTGGAGGACGGTAG  20
Template        464379  ....................  464398

Reverse primer  1       AGGCCTTCGGTAATGATCTTGT  22
Template        464734  ......................  464713
```

>CP011369.1 Pseudomonas aeruginosa strain S04 90 chromosome

```
product length = 356
Forward primer  1       CTGAAGCTGGAGGACGGTAG  20
Template        478608  ....................  478627

Reverse primer  1       AGGCCTTCGGTAATGATCTTGT  22
Template        478963  ......................  478942
```

>CP011317.1 Pseudomonas aeruginosa strain Carb01 63, complete genome

```
product length = 356
Forward primer  1       CTGAAGCTGGAGGACGGTAG  20
Template        555899  ....................  555918

Reverse primer  1       AGGCCTTCGGTAATGATCTTGT  22
Template        556254  ......................  556233
```

>LN831024.1 Pseudomonas aeruginosa genome assembly NCTC10332, chromosome : 1

```
product length = 356
Forward primer  1       CTGAAGCTGGAGGACGGTAG  20
Template        463892  ....................  463911

Reverse primer  1       AGGCCTTCGGTAATGATCTTGT  22
Template        464247  ......................  464226
```

>AP014651.1 Pseudomonas aeruginosa DNA, complete genome, strain: NCGM257

```
product length = 356
Forward primer  1       CTGAAGCTGGAGGACGGTAG  20
Template        559576  ....................  559595

Reverse primer  1       AGGCCTTCGGTAATGATCTTGT  22
Template        559931  ......................  559910
```

>CP010555.1 Pseudomonas aeruginosa strain FRD1, complete genome

```
product length = 356
Forward primer  1        CTGAAGCTGGAGGACGGTAG  20
Template        4282364  ....................  4282383

Reverse primer  1        AGGCCTTCGGTAATGATCTTGT  22
Template        4282719  ......................  4282698
```

>CP007399.1 Pseudomonas aeruginosa strain F22031, complete genome

```
product length = 356
Forward primer  1        CTGAAGCTGGAGGACGGTAG  20
Template        3017475  ....................  3017494

Reverse primer  1        AGGCCTTCGGTAATGATCTTGT  22
Template        3017830  ......................  3017809
```

>AP014646.1 Pseudomonas aeruginosa DNA, complete genome, strain: NCGM 1984

```
product length = 356
Forward primer  1       CTGAAGCTGGAGGACGGTAG  20
Template        473648  ....................  473667

Reverse primer  1       AGGCCTTCGGTAATGATCTTGT  22
Template        474003  ......................  473982
```

>HG974234.1 Pseudomonas aeruginosa strain PSE305, genome

```
product length = 356
Forward primer  1        CTGAAGCTGGAGGACGGTAG  20
Template        6380309  ....................  6380328

Reverse primer  1        AGGCCTTCGGTAATGATCTTGT  22
Template        6380664  ......................  6380643
```

>CP089067.2 Pseudomonas aeruginosa strain UNC_PaerCF19 chromosome, complete genome

```
product length = 356
Forward primer  1       CTGAAGCTGGAGGACGGTAG  20
Template        495912  ....................  495893

Reverse primer  1       AGGCCTTCGGTAATGATCTTGT  22
Template        495557  ......................  495578
```

>CP089068.2 Pseudomonas aeruginosa strain UNC_PaerCF13 chromosome, complete genome

```
product length = 356
Forward primer  1        CTGAAGCTGGAGGACGGTAG  20
Template        1989253  ....................  1989272

Reverse primer  1        AGGCCTTCGGTAATGATCTTGT  22
Template        1989608  ......................  1989587
```

>CP089065.2 Pseudomonas aeruginosa strain UNC_PaerCF34 chromosome, complete genome

```
product length = 356
Forward primer  1       CTGAAGCTGGAGGACGGTAG  20
Template        253932  ....................  253913

Reverse primer  1       AGGCCTTCGGTAATGATCTTGT  22
Template        253577  ......................  253598
```

>CP069331.1 Pseudomonas aeruginosa strain R09 chromosome, complete genome

```
product length = 356
Forward primer  1       CTGAAGCTGGAGGACGGTAG  20
Template        453318  ....................  453337

Reverse primer  1       AGGCCTTCGGTAATGATCTTGT  22
Template        453673  ......................  453652
```

>CP069337.1 Pseudomonas aeruginosa strain E04 chromosome, complete genome

```
product length = 356
Forward primer  1       CTGAAGCTGGAGGACGGTAG  20
Template        504091  ....................  504110

Reverse primer  1       AGGCCTTCGGTAATGATCTTGT  22
Template        504446  ......................  504425
```

>CP069336.1 Pseudomonas aeruginosa strain E01 chromosome, complete genome

```
product length = 356
Forward primer  1       CTGAAGCTGGAGGACGGTAG  20
Template        453366  ....................  453385

Reverse primer  1       AGGCCTTCGGTAATGATCTTGT  22
Template        453721  ......................  453700
```

>CP069335.1 Pseudomonas aeruginosa strain E02 chromosome, complete genome

```
product length = 356
Forward primer  1       CTGAAGCTGGAGGACGGTAG  20
Template        453367  ....................  453386

Reverse primer  1       AGGCCTTCGGTAATGATCTTGT  22
Template        453722  ......................  453701
```

>CP069334.1 Pseudomonas aeruginosa strain E03 chromosome, complete genome

```
product length = 356
Forward primer  1       CTGAAGCTGGAGGACGGTAG  20
Template        453367  ....................  453386

Reverse primer  1       AGGCCTTCGGTAATGATCTTGT  22
Template        453722  ......................  453701
```

>CP069333.1 Pseudomonas aeruginosa strain R01 chromosome, complete genome

```
product length = 356
Forward primer  1       CTGAAGCTGGAGGACGGTAG  20
Template        453363  ....................  453382

Reverse primer  1       AGGCCTTCGGTAATGATCTTGT  22
Template        453718  ......................  453697
```

>CP069332.1 Pseudomonas aeruginosa strain R08 chromosome, complete genome

```
product length = 356
Forward primer  1       CTGAAGCTGGAGGACGGTAG  20
Template        453318  ....................  453337

Reverse primer  1       AGGCCTTCGGTAATGATCTTGT  22
Template        453673  ......................  453652
```

>CP069330.1 Pseudomonas aeruginosa strain R07 chromosome, complete genome

```
product length = 356
Forward primer  1       CTGAAGCTGGAGGACGGTAG  20
Template        454024  ....................  454043

Reverse primer  1       AGGCCTTCGGTAATGATCTTGT  22
Template        454379  ......................  454358
```

>CP069329.1 Pseudomonas aeruginosa strain R10 chromosome, complete genome

```
product length = 356
Forward primer  1       CTGAAGCTGGAGGACGGTAG  20
Template        453318  ....................  453337

Reverse primer  1       AGGCCTTCGGTAATGATCTTGT  22
Template        453673  ......................  453652
```

>CP069328.1 Pseudomonas aeruginosa strain R04 chromosome, complete genome

```
product length = 356
Forward primer  1       CTGAAGCTGGAGGACGGTAG  20
Template        453345  ....................  453364

Reverse primer  1       AGGCCTTCGGTAATGATCTTGT  22
Template        453700  ......................  453679
```

>CP069327.1 Pseudomonas aeruginosa strain R03 chromosome, complete genome

```
product length = 356
Forward primer  1       CTGAAGCTGGAGGACGGTAG  20
Template        453336  ....................  453355

Reverse primer  1       AGGCCTTCGGTAATGATCTTGT  22
Template        453691  ......................  453670
```

>CP069326.1 Pseudomonas aeruginosa strain R11 chromosome, complete genome

```
product length = 356
Forward primer  1       CTGAAGCTGGAGGACGGTAG  20
Template        453327  ....................  453346

Reverse primer  1       AGGCCTTCGGTAATGATCTTGT  22
Template        453682  ......................  453661
```

>CP069325.1 Pseudomonas aeruginosa strain R02 chromosome, complete genome

```
product length = 356
Forward primer  1       CTGAAGCTGGAGGACGGTAG  20
Template        453360  ....................  453379

Reverse primer  1       AGGCCTTCGGTAATGATCTTGT  22
Template        453715  ......................  453694
```

>CP069324.1 Pseudomonas aeruginosa strain R05 chromosome, complete genome

```
product length = 356
Forward primer  1       CTGAAGCTGGAGGACGGTAG  20
Template        453339  ....................  453358

Reverse primer  1       AGGCCTTCGGTAATGATCTTGT  22
Template        453694  ......................  453673
```

>CP069323.1 Pseudomonas aeruginosa strain R06 chromosome, complete genome

```
product length = 356
Forward primer  1       CTGAAGCTGGAGGACGGTAG  20
Template        453339  ....................  453358

Reverse primer  1       AGGCCTTCGGTAATGATCTTGT  22
Template        453694  ......................  453673
```

>CP008739.2 Pseudomonas aeruginosa VRFPA04, complete genome

```
product length = 356
Forward primer  1        CTGAAGCTGGAGGACGGTAG  20
Template        6230748  ....................  6230729

Reverse primer  1        AGGCCTTCGGTAATGATCTTGT  22
Template        6230393  ......................  6230414
```

>AP014622.1 Pseudomonas aeruginosa DNA, complete genome, strain: NCGM 1900

```
product length = 356
Forward primer  1       CTGAAGCTGGAGGACGGTAG  20
Template        473646  ....................  473665

Reverse primer  1       AGGCCTTCGGTAATGATCTTGT  22
Template        474001  ......................  473980
```

>CP008749.1 Pseudomonas aeruginosa PAO1H2O genome

```
product length = 356
Forward primer  1       CTGAAGCTGGAGGACGGTAG  20
Template        472714  ....................  472733

Reverse primer  1       AGGCCTTCGGTAATGATCTTGT  22
Template        473069  ......................  473048
```

>CP007224.1 Pseudomonas aeruginosa PA96 genome

```
product length = 356
Forward primer  1       CTGAAGCTGGAGGACGGTAG  20
Template        467467  ....................  467486

Reverse primer  1       AGGCCTTCGGTAATGATCTTGT  22
Template        467822  ......................  467801
```

>CP006985.1 Pseudomonas aeruginosa LESlike4 sequence

```
product length = 356
Forward primer  1       CTGAAGCTGGAGGACGGTAG  20
Template        466004  ....................  466023

Reverse primer  1       AGGCCTTCGGTAATGATCTTGT  22
Template        466359  ......................  466338
```

>CP006984.1 Pseudomonas aeruginosa LESlike1 chromosome

```
product length = 356
Forward primer  1       CTGAAGCTGGAGGACGGTAG  20
Template        466560  ....................  466579

Reverse primer  1       AGGCCTTCGGTAATGATCTTGT  22
Template        466915  ......................  466894
```

>CP006983.1 Pseudomonas aeruginosa LESB65 sequence

```
product length = 356
Forward primer  1       CTGAAGCTGGAGGACGGTAG  20
Template        466067  ....................  466086

Reverse primer  1       AGGCCTTCGGTAATGATCTTGT  22
Template        466422  ......................  466401
```

>CP006982.1 Pseudomonas aeruginosa LES400 sequence

```
product length = 356
Forward primer  1       CTGAAGCTGGAGGACGGTAG  20
Template        466229  ....................  466248

Reverse primer  1       AGGCCTTCGGTAATGATCTTGT  22
Template        466584  ......................  466563
```

>CP006981.1 Pseudomonas aeruginosa LESlike7 sequence

```
product length = 356
Forward primer  1       CTGAAGCTGGAGGACGGTAG  20
Template        466437  ....................  466456

Reverse primer  1       AGGCCTTCGGTAATGATCTTGT  22
Template        466792  ......................  466771
```

>CP006980.1 Pseudomonas aeruginosa LESlike5 sequence

```
product length = 356
Forward primer  1       CTGAAGCTGGAGGACGGTAG  20
Template        466523  ....................  466542

Reverse primer  1       AGGCCTTCGGTAATGATCTTGT  22
Template        466878  ......................  466857
```

>CP007147.1 Pseudomonas aeruginosa YL84, complete genome

```
product length = 356
Forward primer  1       CTGAAGCTGGAGGACGGTAG  20
Template        358629  ....................  358610

Reverse primer  1       AGGCCTTCGGTAATGATCTTGT  22
Template        358274  ......................  358295
```

>HG530068.1 Pseudomonas aeruginosa PA38182, complete genome

```
product length = 356
Forward primer  1       CTGAAGCTGGAGGACGGTAG  20
Template        499683  ....................  499702

Reverse primer  1       AGGCCTTCGGTAATGATCTTGT  22
Template        500038  ......................  500017
```

>CP006931.1 Pseudomonas aeruginosa SCV20265, complete genome

```
product length = 356
Forward primer  1       CTGAAGCTGGAGGACGGTAG  20
Template        472563  ....................  472582

Reverse primer  1       AGGCCTTCGGTAATGATCTTGT  22
Template        472918  ......................  472897
```

>CP006937.1 Pseudomonas aeruginosa LES431, complete genome

```
product length = 356
Forward primer  1       CTGAAGCTGGAGGACGGTAG  20
Template        466450  ....................  466469

Reverse primer  1       AGGCCTTCGGTAATGATCTTGT  22
Template        466805  ......................  466784
```

>CP006853.1 Pseudomonas aeruginosa MTB-1, complete genome

```
product length = 356
Forward primer  1       CTGAAGCTGGAGGACGGTAG  20
Template        473449  ....................  473468

Reverse primer  1       AGGCCTTCGGTAATGATCTTGT  22
Template        473804  ......................  473783
```

>CP004055.1 Pseudomonas aeruginosa PA1R, complete genome

```
product length = 356
Forward primer  1        CTGAAGCTGGAGGACGGTAG  20
Template        4389215  ....................  4389234

Reverse primer  1        AGGCCTTCGGTAATGATCTTGT  22
Template        4389570  ......................  4389549
```

>CP081345.1 Pseudomonas aeruginosa strain F291007 chromosome, complete genome

```
product length = 356
Forward primer  1       CTGAAGCTGGAGGACGGTAG  20
Template        509730  ....................  509749

Reverse primer  1       AGGCCTTCGGTAATGATCTTGT  22
Template        510085  ......................  510064
```

>CP081346.1 Pseudomonas aeruginosa strain SE5419 chromosome, complete genome

```
product length = 356
Forward primer  1       CTGAAGCTGGAGGACGGTAG  20
Template        481256  ....................  481275

Reverse primer  1       AGGCCTTCGGTAATGATCTTGT  22
Template        481611  ......................  481590
```

>CP081287.1 Pseudomonas aeruginosa strain F092021 chromosome, complete genome

```
product length = 356
Forward primer  1       CTGAAGCTGGAGGACGGTAG  20
Template        513184  ....................  513203

Reverse primer  1       AGGCCTTCGGTAATGATCTTGT  22
Template        513539  ......................  513518
```

>CP081202.1 Pseudomonas aeruginosa strain P9W chromosome, complete genome

```
product length = 356
Forward primer  1        CTGAAGCTGGAGGACGGTAG  20
Template        5160307  ....................  5160288

Reverse primer  1        AGGCCTTCGGTAATGATCTTGT  22
Template        5159952  ......................  5159973
```

>CP006832.1 Pseudomonas aeruginosa PAO1-VE13 genome

```
product length = 356
Forward primer  1       CTGAAGCTGGAGGACGGTAG  20
Template        472714  ....................  472733

Reverse primer  1       AGGCCTTCGGTAATGATCTTGT  22
Template        473069  ......................  473048
```

>CP006831.1 Pseudomonas aeruginosa PAO1-VE2 genome

```
product length = 356
Forward primer  1       CTGAAGCTGGAGGACGGTAG  20
Template        472714  ....................  472733

Reverse primer  1       AGGCCTTCGGTAATGATCTTGT  22
Template        473069  ......................  473048
```

>CP006705.1 Pseudomonas aeruginosa PAO581 genome

```
product length = 356
Forward primer  1       CTGAAGCTGGAGGACGGTAG  20
Template        472590  ....................  472609

Reverse primer  1       AGGCCTTCGGTAATGATCTTGT  22
Template        472945  ......................  472924
```

>CP006728.1 Pseudomonas aeruginosa c7447m genome

```
product length = 356
Forward primer  1       CTGAAGCTGGAGGACGGTAG  20
Template        472691  ....................  472710

Reverse primer  1       AGGCCTTCGGTAATGATCTTGT  22
Template        473046  ......................  473025
```

>CP006245.1 Pseudomonas aeruginosa RP73, complete genome

```
product length = 356
Forward primer  1       CTGAAGCTGGAGGACGGTAG  20
Template        467332  ....................  467351

Reverse primer  1       AGGCCTTCGGTAATGATCTTGT  22
Template        467687  ......................  467666
```

>CP074424.1 Pseudomonas aeruginosa strain 88A chromosome

```
product length = 356
Forward primer  1        CTGAAGCTGGAGGACGGTAG  20
Template        4371094  ....................  4371113

Reverse primer  1        AGGCCTTCGGTAATGATCTTGT  22
Template        4371449  ......................  4371428
```

>CP061780.1 Pseudomonas aeruginosa strain ZBX-P11 chromosome, complete genome

```
product length = 356
Forward primer  1        CTGAAGCTGGAGGACGGTAG  20
Template        3078285  ....................  3078304

Reverse primer  1        AGGCCTTCGGTAATGATCTTGT  22
Template        3078640  ......................  3078619
```

>CP061779.1 Pseudomonas aeruginosa strain ZBX-P12 chromosome, complete genome

```
product length = 356
Forward primer  1        CTGAAGCTGGAGGACGGTAG  20
Template        6509389  ....................  6509408

Reverse primer  1        AGGCCTTCGGTAATGATCTTGT  22
Template        6509744  ......................  6509723
```

>CP061778.1 Pseudomonas aeruginosa strain ZBX-P13 chromosome, complete genome

```
product length = 356
Forward primer  1        CTGAAGCTGGAGGACGGTAG  20
Template        2219643  ....................  2219662

Reverse primer  1        AGGCCTTCGGTAATGATCTTGT  22
Template        2219998  ......................  2219977
```

>CP061777.1 Pseudomonas aeruginosa strain ZBX-P23 chromosome, complete genome

```
product length = 356
Forward primer  1       CTGAAGCTGGAGGACGGTAG  20
Template        183719  ....................  183738

Reverse primer  1       AGGCCTTCGGTAATGATCTTGT  22
Template        184074  ......................  184053
```

>CP004061.1 Pseudomonas aeruginosa B136-33, complete genome

```
product length = 356
Forward primer  1       CTGAAGCTGGAGGACGGTAG  20
Template        464577  ....................  464596

Reverse primer  1       AGGCCTTCGGTAATGATCTTGT  22
Template        464932  ......................  464911
```

>CP070471.1 Pseudomonas aeruginosa strain B17932 chromosome, complete genome

```
product length = 356
Forward primer  1        CTGAAGCTGGAGGACGGTAG  20
Template        6334673  ....................  6334654

Reverse primer  1        AGGCCTTCGGTAATGATCTTGT  22
Template        6334318  ......................  6334339
```

>CP070467.1 Pseudomonas aeruginosa strain B17416 chromosome, complete genome

```
product length = 356
Forward primer  1        CTGAAGCTGGAGGACGGTAG  20
Template        6573313  ....................  6573294

Reverse primer  1        AGGCCTTCGGTAATGATCTTGT  22
Template        6572958  ......................  6572979
```

>CP070355.1 Pseudomonas aeruginosa strain PDNC003 chromosome

```
product length = 356
Forward primer  1        CTGAAGCTGGAGGACGGTAG  20
Template        5620708  ....................  5620727

Reverse primer  1        AGGCCTTCGGTAATGATCTTGT  22
Template        5621063  ......................  5621042
```

>CP003149.1 Pseudomonas aeruginosa DK2, complete genome

```
product length = 358
Forward primer  1       CTGAAGCTGGAGGACGGTAG  20
Template        466720  ....................  466739

Reverse primer  1       AGGCCTTCGGTAATGATCTTGT  22
Template        467077  ......................  467056
```

>CP063237.1 Pseudomonas aeruginosa strain mPA08-31 chromosome

```
product length = 356
Forward primer  1       CTGAAGCTGGAGGACGGTAG  20
Template        414729  ....................  414748

Reverse primer  1       AGGCCTTCGGTAATGATCTTGT  22
Template        415084  ......................  415063
```

>CP063047.1 Pseudomonas aeruginosa strain KC-Tt-1 chromosome, complete genome

```
product length = 356
Forward primer  1        CTGAAGCTGGAGGACGGTAG  20
Template        5957455  ....................  5957474

Reverse primer  1        AGGCCTTCGGTAATGATCTTGT  22
Template        5957810  ......................  5957789
```

>CP062219.1 Pseudomonas aeruginosa strain JT86 chromosome, complete genome

```
product length = 356
Forward primer  1        CTGAAGCTGGAGGACGGTAG  20
Template        5245530  ....................  5245549

Reverse primer  1        AGGCCTTCGGTAATGATCTTGT  22
Template        5245885  ......................  5245864
```

>CP061850.1 Pseudomonas aeruginosa strain R31 chromosome, complete genome

```
product length = 356
Forward primer  1        CTGAAGCTGGAGGACGGTAG  20
Template        6779538  ....................  6779557

Reverse primer  1        AGGCCTTCGGTAATGATCTTGT  22
Template        6779893  ......................  6779872
```

>CP058257.1 Pseudomonas aeruginosa strain PA179 chromosome

```
product length = 356
Forward primer  1       CTGAAGCTGGAGGACGGTAG  20
Template        577689  ....................  577708

Reverse primer  1       AGGCCTTCGGTAATGATCTTGT  22
Template        578044  ......................  578023
```

>CP059852.1 Pseudomonas aeruginosa strain ZM03 chromosome, complete genome

```
product length = 356
Forward primer  1        CTGAAGCTGGAGGACGGTAG  20
Template        3777532  ....................  3777513

Reverse primer  1        AGGCCTTCGGTAATGATCTTGT  22
Template        3777177  ......................  3777198
```

>AP012280.1 Pseudomonas aeruginosa NCGM2.S1 DNA, complete genome

```
product length = 356
Forward primer  1        CTGAAGCTGGAGGACGGTAG  20
Template        6157938  ....................  6157919

Reverse primer  1        AGGCCTTCGGTAATGATCTTGT  22
Template        6157583  ......................  6157604
```

>CP002496.1 Pseudomonas aeruginosa M18, complete genome

```
product length = 356
Forward primer  1       CTGAAGCTGGAGGACGGTAG  20
Template        466238  ....................  466257

Reverse primer  1       AGGCCTTCGGTAATGATCTTGT  22
Template        466593  ......................  466572
```

>FM209186.1 Pseudomonas aeruginosa LESB58 complete genome sequence

```
product length = 356
Forward primer  1       CTGAAGCTGGAGGACGGTAG  20
Template        466455  ....................  466474

Reverse primer  1       AGGCCTTCGGTAATGATCTTGT  22
Template        466810  ......................  466789
```

>CP000438.1 Pseudomonas aeruginosa UCBPP-PA14, complete genome

```
product length = 356
Forward primer  1       CTGAAGCTGGAGGACGGTAG  20
Template        487465  ....................  487484

Reverse primer  1       AGGCCTTCGGTAATGATCTTGT  22
Template        487820  ......................  487799
```

>AE004091.2 Pseudomonas aeruginosa PAO1, complete genome

```
product length = 356
Forward primer  1       CTGAAGCTGGAGGACGGTAG  20
Template        472714  ....................  472733

Reverse primer  1       AGGCCTTCGGTAATGATCTTGT  22
Template        473069  ......................  473048
```

>AF092566.1 Pseudomonas aeruginosa multidrug resistance protein MexA precursor (mexA) and multidrug resistance protein MexB (mexB) genes, partial cds

```
product length = 356
Forward primer  1    CTGAAGCTGGAGGACGGTAG  20
Template        148  ....................  167

Reverse primer  1    AGGCCTTCGGTAATGATCTTGT  22
Template        503  ......................  482
```

>L11616.1 Pseudomonas aeruginosa mexA and mexB genes, complete cds and outer membrane protein (oprM) gene, partial cds

```
product length = 356
Forward primer  1     CTGAAGCTGGAGGACGGTAG  20
Template        1093  ....................  1112

Reverse primer  1     AGGCCTTCGGTAATGATCTTGT  22
Template        1448  ......................  1427
```

>CP096956.1 Pseudomonas aeruginosa strain NY11173 chromosome, complete genome

```
product length = 356
Forward primer  1       CTGAAGCTGGAGGACGGTAG  20
Template        471532  ...........A........  471551

Reverse primer  1       AGGCCTTCGGTAATGATCTTGT  22
Template        471887  ......................  471866
```

>CP124658.1 Pseudomonas aeruginosa strain 2022CK-00068 chromosome, complete genome

```
product length = 356
Forward primer  1       CTGAAGCTGGAGGACGGTAG  20
Template        473524  ......T.............  473543

Reverse primer  1       AGGCCTTCGGTAATGATCTTGT  22
Template        473879  ......................  473858
```

>CP075827.1 Pseudomonas aeruginosa strain PaLo27 chromosome, complete genome

```
product length = 356
Forward primer  1       CTGAAGCTGGAGGACGGTAG  20
Template        533276  .............G......  533295

Reverse primer  1       AGGCCTTCGGTAATGATCTTGT  22
```

```
Template        533631  ......................  533610
```

>CP075817.1 Pseudomonas aeruginosa strain PaLo39 chromosome, complete genome

```
product length = 356
Forward primer  1       CTGAAGCTGGAGGACGGTAG  20
Template        477216  .C..................  477235

Reverse primer  1       AGGCCTTCGGTAATGATCTTGT  22
Template        477571  ......................  477550
```

>CP075814.1 Pseudomonas aeruginosa strain PaLo44 chromosome, complete genome

```
product length = 356
Forward primer  1       CTGAAGCTGGAGGACGGTAG  20
Template        477223  .C..................  477242

Reverse primer  1       AGGCCTTCGGTAATGATCTTGT  22
Template        477578  ......................  477557
```

>CP075811.1 Pseudomonas aeruginosa strain PaLo152 chromosome, complete genome

```
product length = 356
Forward primer  1       CTGAAGCTGGAGGACGGTAG  20
Template        469738  ...........A........  469757

Reverse primer  1       AGGCCTTCGGTAATGATCTTGT  22
Template        470093  ......................  470072
```

>CP075797.1 Pseudomonas aeruginosa strain PaLo326 chromosome, complete genome

```
product length = 356
Forward primer  1       CTGAAGCTGGAGGACGGTAG  20
Template        473800  ..............T.....  473819

Reverse primer  1       AGGCCTTCGGTAATGATCTTGT  22
Template        474155  ......................  474134
```

>CP075794.1 Pseudomonas aeruginosa strain PaLo418 chromosome, complete genome

```
product length = 356
Forward primer  1       CTGAAGCTGGAGGACGGTAG  20
Template        466245  ...........A........  466264

Reverse primer  1       AGGCCTTCGGTAATGATCTTGT  22
Template        466600  ......................  466579
```

>CP075793.1 Pseudomonas aeruginosa strain PaLo419 chromosome, complete genome

```
product length = 356
Forward primer  1       CTGAAGCTGGAGGACGGTAG  20
Template        466245  ...........A........  466264
```

```
Reverse primer  1       AGGCCTTCGGTAATGATCTTGT  22
Template        466600  ......................  466579
```

>CP075792.1 Pseudomonas aeruginosa strain PaLo422 chromosome, complete genome

```
product length = 356
Forward primer  1       CTGAAGCTGGAGGACGGTAG  20
Template        532358  ...........A........  532377

Reverse primer  1       AGGCCTTCGGTAATGATCTTGT  22
Template        532713  ......................  532692
```

>CP075788.1 Pseudomonas aeruginosa strain PaLo502 chromosome, complete genome

```
product length = 356
Forward primer  1       CTGAAGCTGGAGGACGGTAG  20
Template        449637  ...........A........  449656

Reverse primer  1       AGGCCTTCGGTAATGATCTTGT  22
Template        449992  ......................  449971
```

>CP075778.1 Pseudomonas aeruginosa strain PaLo527 chromosome, complete genome

```
product length = 356
Forward primer  1       CTGAAGCTGGAGGACGGTAG  20
Template        466422  ..T.................  466441

Reverse primer  1       AGGCCTTCGGTAATGATCTTGT  22
Template        466777  ......................  466756
```

>CP096822.1 Pseudomonas aeruginosa strain NY8709 chromosome, complete genome

```
product length = 356
Forward primer  1       CTGAAGCTGGAGGACGGTAG  20
Template        478262  ...........A........  478281

Reverse primer  1       AGGCCTTCGGTAATGATCTTGT  22
Template        478617  ......................  478596
```

>CP109850.1 Pseudomonas aeruginosa strain PALA37 chromosome, complete genome

```
product length = 356
Forward primer  1        CTGAAGCTGGAGGACGGTAG  20
Template        1915671  ...........A........  1915652

Reverse primer  1        AGGCCTTCGGTAATGATCTTGT  22
Template        1915316  ......................  1915337
```

>CP110346.1 Pseudomonas aeruginosa strain PALA35 chromosome, complete genome

```
product length = 356
Forward primer  1       CTGAAGCTGGAGGACGGTAG  20
Template        465380  ...........A........  465399
```

```
Reverse primer  1       AGGCCTTCGGTAATGATCTTGT  22
Template        465735  ......................  465714
```

>CP114761.1 Pseudomonas aeruginosa strain NF143349 chromosome, complete genome

```
product length = 356
Forward primer  1       CTGAAGCTGGAGGACGGTAG  20
Template        537540  ...........A........  537559

Reverse primer  1       AGGCCTTCGGTAATGATCTTGT  22
Template        537895  ......................  537874
```

>CP097557.1 Pseudomonas aeruginosa strain C1.3 chromosome, complete genome

```
product length = 356
Forward primer  1       CTGAAGCTGGAGGACGGTAG  20
Template        462539  ......T.............  462558

Reverse primer  1       AGGCCTTCGGTAATGATCTTGT  22
Template        462894  ......................  462873
```

>CP109657.1 Pseudomonas aeruginosa strain Zw26 chromosome, complete genome

```
product length = 356
Forward primer  1       CTGAAGCTGGAGGACGGTAG  20
Template        439963  ...........A........  439982

Reverse primer  1       AGGCCTTCGGTAATGATCTTGT  22
Template        440318  ......................  440297
```

>CP104586.1 Pseudomonas aeruginosa strain WTJH6 chromosome, complete genome

```
product length = 356
Forward primer  1        CTGAAGCTGGAGGACGGTAG  20
Template        2693962  ...........A........  2693943

Reverse primer  1        AGGCCTTCGGTAATGATCTTGT  22
Template        2693607  ......................  2693628
```

>CP100759.1 Pseudomonas aeruginosa strain PA0009 chromosome

```
product length = 356
Forward primer  1       CTGAAGCTGGAGGACGGTAG  20
Template        320336  ..............T.....  320317

Reverse primer  1       AGGCCTTCGGTAATGATCTTGT  22
Template        319981  ......................  320002
```

>CP093021.1 Pseudomonas aeruginosa strain H09 chromosome, complete genome

```
product length = 356
Forward primer  1       CTGAAGCTGGAGGACGGTAG  20
```

```
Template        474534  ..............T.....  474553

Reverse primer  1       AGGCCTTCGGTAATGATCTTGT  22
Template        474889  ......................  474868
```

>CP086064.1 Pseudomonas aeruginosa strain CCBH28525 chromosome, complete genome

```
product length = 356
Forward primer  1       CTGAAGCTGGAGGACGGTAG  20
Template        471299  ...........A........  471318

Reverse primer  1       AGGCCTTCGGTAATGATCTTGT  22
Template        471654  ......................  471633
```

>CP080286.1 Pseudomonas aeruginosa strain UNC_PaerCF12 chromosome, complete genome

```
product length = 356
Forward primer  1        CTGAAGCTGGAGGACGGTAG  20
Template        1730147  ...........A........  1730166

Reverse primer  1        AGGCCTTCGGTAATGATCTTGT  22
Template        1730502  ......................  1730481
```

>CP072783.1 Pseudomonas aeruginosa strain LICME WGH-6 chromosome, complete genome

```
product length = 356
Forward primer  1       CTGAAGCTGGAGGACGGTAG  20
Template        469799  ...........A........  469818

Reverse primer  1       AGGCCTTCGGTAATGATCTTGT  22
Template        470154  ......................  470133
```

>CP068238.1 Pseudomonas aeruginosa strain A39-1 chromosome, complete genome

```
product length = 356
Forward primer  1       CTGAAGCTGGAGGACGGTAG  20
Template        430809  ...........A........  430828

Reverse primer  1       AGGCCTTCGGTAATGATCTTGT  22
Template        431164  ......................  431143
```

>CP061699.1 Pseudomonas aeruginosa strain LYSZa7 chromosome, complete genome

```
product length = 356
Forward primer  1        CTGAAGCTGGAGGACGGTAG  20
Template        6273356  ...........A........  6273337

Reverse primer  1        AGGCCTTCGGTAATGATCTTGT  22
Template        6273001  ......................  6273022
```

>CP050330.1 Pseudomonas aeruginosa strain DVT779 chromosome, complete genome

```
product length = 356
```

```
Forward primer  1       CTGAAGCTGGAGGACGGTAG  20
Template        476422  ...........A........  476441

Reverse primer  1       AGGCCTTCGGTAATGATCTTGT  22
Template        476777  ......................  476756
```

>CP053687.1 Pseudomonas aeruginosa strain K19PSE24 chromosome

```
product length = 356
Forward primer  1        CTGAAGCTGGAGGACGGTAG  20
Template        6416552  ......T.............  6416571

Reverse primer  1        AGGCCTTCGGTAATGATCTTGT  22
Template        6416907  ......................  6416886
```

>CP021380.2 Pseudomonas aeruginosa strain CCBH4851 genome

```
product length = 356
Forward primer  1       CTGAAGCTGGAGGACGGTAG  20
Template        471718  ...........A........  471737

Reverse primer  1       AGGCCTTCGGTAATGATCTTGT  22
Template        472073  ......................  472052
```

>CP041773.1 Pseudomonas aeruginosa strain 519119 chromosome, complete genome

```
product length = 356
Forward primer  1        CTGAAGCTGGAGGACGGTAG  20
Template        2973141  ...........A........  2973160

Reverse primer  1        AGGCCTTCGGTAATGATCTTGT  22
Template        2973496  ......................  2973475
```

>CP041354.1 Pseudomonas aeruginosa strain AZPAE15042 chromosome, complete genome

```
product length = 356
Forward primer  1       CTGAAGCTGGAGGACGGTAG  20
Template        441658  ...........A........  441677

Reverse primer  1       AGGCCTTCGGTAATGATCTTGT  22
Template        442013  ......................  441992
```

>CP040127.1 Pseudomonas aeruginosa strain PA298 chromosome, complete genome

```
product length = 356
Forward primer  1       CTGAAGCTGGAGGACGGTAG  20
Template        472279  ...........A........  472298

Reverse primer  1       AGGCCTTCGGTAATGATCTTGT  22
Template        472634  ......................  472613
```

>CP028368.1 Pseudomonas aeruginosa strain PA-VAP-4 chromosome

```
product length = 356
Forward primer  1        CTGAAGCTGGAGGACGGTAG  20
Template        3154593  ...........A........  3154612

Reverse primer  1        AGGCCTTCGGTAATGATCTTGT  22
Template        3154948  ......................  3154927
```

>CP031677.1 Pseudomonas aeruginosa strain E80 chromosome, complete genome

```
product length = 356
Forward primer  1       CTGAAGCTGGAGGACGGTAG  20
Template        579853  ...........A........  579872

Reverse primer  1       AGGCCTTCGGTAATGATCTTGT  22
Template        580208  ......................  580187
```

>CP028584.2 Pseudomonas aeruginosa strain WCHPA075019 chromosome, complete genome

```
product length = 356
Forward primer  1       CTGAAGCTGGAGGACGGTAG  20
Template        471240  ...........A........  471259

Reverse primer  1       AGGCCTTCGGTAATGATCTTGT  22
Template        471595  ......................  471574
```

>CP030911.1 Pseudomonas aeruginosa strain Y71 chromosome, complete genome

```
product length = 356
Forward primer  1       CTGAAGCTGGAGGACGGTAG  20
Template        490343  ...........A........  490362

Reverse primer  1       AGGCCTTCGGTAATGATCTTGT  22
Template        490698  ......................  490677
```

>CP030913.1 Pseudomonas aeruginosa strain Y89 chromosome, complete genome

```
product length = 356
Forward primer  1       CTGAAGCTGGAGGACGGTAG  20
Template        490996  ...........A........  491015

Reverse primer  1       AGGCCTTCGGTAATGATCTTGT  22
Template        491351  ......................  491330
```

>LS483497.1 Pseudomonas aeruginosa strain NCTC9433 genome assembly, chromosome: 1

```
product length = 356
Forward primer  1       CTGAAGCTGGAGGACGGTAG  20
Template        454052  ...........A........  454071

Reverse primer  1       AGGCCTTCGGTAATGATCTTGT  22
Template        454407  ......................  454386
```

>CP029660.1 Pseudomonas aeruginosa strain AR_0446 chromosome, complete genome

```
product length = 356
Forward primer  1        CTGAAGCTGGAGGACGGTAG  20
Template        5618214  ...........A........  5618233

Reverse primer  1        AGGCCTTCGGTAATGATCTTGT  22
Template        5618569  ......................  5618548
```

>CP029093.1 Pseudomonas paraeruginosa strain AR441 chromosome, complete genome

```
product length = 356
Forward primer  1       CTGAAGCTGGAGGACGGTAG  20
Template        501757  ...........A........  501776

Reverse primer  1       AGGCCTTCGGTAATGATCTTGT  22
Template        502112  ......................  502091
```

>CP020560.1 Pseudomonas paraeruginosa strain Cr1 chromosome, complete genome

```
product length = 356
Forward primer  1       CTGAAGCTGGAGGACGGTAG  20
Template        446914  ...........A........  446933

Reverse primer  1       AGGCCTTCGGTAATGATCTTGT  22
Template        447269  ......................  447248
```

>CP027169.1 Pseudomonas paraeruginosa strain AR_0356 chromosome, complete genome

```
product length = 356
Forward primer  1        CTGAAGCTGGAGGACGGTAG  20
Template        1762075  ...........A........  1762094

Reverse primer  1        AGGCCTTCGGTAATGATCTTGT  22
Template        1762430  ......................  1762409
```

>CP022002.1 Pseudomonas aeruginosa strain Pa1242, complete genome

```
product length = 356
Forward primer  1       CTGAAGCTGGAGGACGGTAG  20
Template        471708  ...........A........  471727

Reverse primer  1       AGGCCTTCGGTAATGATCTTGT  22
Template        472063  ......................  472042
```

>CP014999.1 Pseudomonas aeruginosa strain PA7790, complete genome

```
product length = 356
Forward primer  1       CTGAAGCTGGAGGACGGTAG  20
Template        471763  ...........A........  471782

Reverse primer  1       AGGCCTTCGGTAATGATCTTGT  22
Template        472118  ......................  472097
```

>CP015003.1 Pseudomonas aeruginosa strain PA11803 chromosome, complete genome

```
product length = 356
Forward primer  1       CTGAAGCTGGAGGACGGTAG  20
Template        471750  ...........A........  471769

Reverse primer  1       AGGCCTTCGGTAATGATCTTGT  22
Template        472105  ......................  472084
```

>CP015002.1 Pseudomonas aeruginosa strain PA8281 chromosome, complete genome

```
product length = 356
Forward primer  1       CTGAAGCTGGAGGACGGTAG  20
Template        471757  ...........A........  471776

Reverse primer  1       AGGCCTTCGGTAATGATCTTGT  22
Template        472112  ......................  472091
```

>CP015001.1 Pseudomonas aeruginosa strain PA1088 chromosome, complete genome

```
product length = 356
Forward primer  1       CTGAAGCTGGAGGACGGTAG  20
Template        471757  ...........A........  471776

Reverse primer  1       AGGCCTTCGGTAATGATCTTGT  22
Template        472112  ......................  472091
```

>CP015377.1 Pseudomonas aeruginosa strain BAMCPA07-48 chromosome, complete genome

```
product length = 356
Forward primer  1        CTGAAGCTGGAGGACGGTAG  20
Template        2557092  ......T.............  2557111

Reverse primer  1        AGGCCTTCGGTAATGATCTTGT  22
Template        2557447  ......................  2557426
```

>CP068678.1 Pseudomonas aeruginosa strain NCCP15783 chromosome, complete genome

```
product length = 356
Forward primer  1        CTGAAGCTGGAGGACGGTAG  20
Template        1979822  ...........A........  1979841

Reverse primer  1        AGGCCTTCGGTAATGATCTTGT  22
Template        1980177  ......................  1980156
```

>CP000744.1 Pseudomonas aeruginosa PA7, complete genome

```
product length = 356
Forward primer  1       CTGAAGCTGGAGGACGGTAG  20
Template        539087  ...........A........  539106

Reverse primer  1       AGGCCTTCGGTAATGATCTTGT  22
Template        539442  ......................  539421
```

>CP050326.1 Pseudomonas aeruginosa strain DVT423 chromosome, complete genome

```
product length = 356
Forward primer  1       CTGAAGCTGGAGGACGGTAG  20
Template        518001  ....................  518020

Reverse primer  1       AGGCCTTCGGTAATGATCTTGT  22
Template        518356  .......A..............  518335
```

>DQ235693.1 Acinetobacter baumannii MexA-like protein (mexA) gene, complete sequence

```
product length = 356
Forward primer  1    CTGAAGCTGGAGGACGGTAG  20
Template        707  .................C..  726

Reverse primer  1     AGGCCTTCGGTAATGATCTTGT  22
Template        1062  ...................G..  1041
```

>CP076630.1 Cercospora sojina strain CCC 172-09/ARG_09_172 voucher ARG_09_172 chromosome II

```
product length = 2154
Reverse primer  1        AGGCCTTCGGTAATGATCTTGT  22
Template        5665660  .........C.G..........  5665639

Reverse primer  1        AGGCCTTCGGTAATGATCTTGT  22
Template        5663507  .........C.G..........  5663528


product length = 2343
Reverse primer  1        AGGCCTTCGGTAATGATCTTGT  22
Template        3599595  .........C.G..........  3599574

Reverse primer  1        AGGCCTTCGGTAATGATCTTGT  22
Template        3597253  .........C.G..........  3597274
```

>CP036208.1 Cercospora sojina strain RACE15 chromosome II, complete sequence

```
product length = 2154
Reverse primer  1        AGGCCTTCGGTAATGATCTTGT  22
Template        5665538  .........C.G..........  5665517

Reverse primer  1        AGGCCTTCGGTAATGATCTTGT  22
Template        5663385  .........C.G..........  5663406


product length = 2343
Reverse primer  1        AGGCCTTCGGTAATGATCTTGT  22
Template        3599895  .........C.G..........  3599874

Reverse primer  1        AGGCCTTCGGTAATGATCTTGT  22
Template        3597553  .........C.G..........  3597574
```

If you want to allow any of the unintended targets, check the box(es) next to the ones you accept and try again to re-search for specific primers Submit

Help

FOLLOW NCBI

Connect with NLM

National Library of Medicine
8600 Rockville Pike
Bethesda, MD 20894

Web Policies
FOIA
HHS Vulnerability Disclosure

Help
Accessibility
Careers

NLM NIH HHS USA.gov
