# Supplementary material for: Phylogenetic Aspects of Antibiotic Resistance and Biofilm Formation of P. aeruginosa Isolated from Clinical Samples
Source: Can J Infect Dis Med Microbiol. 2024 Jan 13;2024:6213873. doi: 10.1155/2024/6213873 (PMC10799695; doi:10.1155/2024/6213873)
Supplement: Supplementary Materials — Original pictures and primer-blast results. [file 6213873.f1.zip › Mex-B Primer-Blast results.pdf]

An official website of the United States government

Here's how you know

The .gov means it’s official.
Federal government websites often end in .gov or .mil. Before sharing sensitive information, make sure you’re on a federal government site.

The site is secure.
The https:// ensures that you are connecting to the official website and that any information you provide is encrypted and transmitted securely.

Skip to main page content

Access keys NCBI Homepage MyNCBI Homepage Main Content Main Navigation

Log in

Primer-BLAST

» JOB ID:UliMRqCkrQyKNj0zMFMZAUpICDNnWxMuZg

PrimerBLAST users!
We want to hear from you about how PrimerBLAST can be improved.
Contact us

Primer-BLAST Results
Help

•

Input PCR template
none

Specificity of primers
Target templates were found in selected database: Nucleotide collection (nt)

Other reports
Search Summary

Detailed primer reports

You can re-search for specific primers by accepting some of the unintended targets, check the box(es) next to the ones you accept and try again to re-search for specific primers Submit

Help

Primer pair 1

	Sequence (5'->3')	Length	Tm	GC%	Self complementarity	Self 3' complementarity
Forward primer	TGGGTGATCGCCTTGGTGA	19	61.22	57.89	6.00	4.00
Reverse primer	GGCCAGTTGCAGCTTGTTC	19	60.01	57.89	6.00	2.00

Products on intended targets

Products on allowed targets

Products on allowed transcript variants

Products on potentially unintended templates

Products on target templates

>CP127126.1 Pseudomonas aeruginosa PA14 strain MA3 isolate DTU_MIE chromosome, complete genome

```
product length = 306
Forward primer  1       TGGGTGATCGCCTTGGTGA  19
Template        487496  ...................  487514

Reverse primer  1       GGCCAGTTGCAGCTTGTTC  19
Template        487801  ...................  487783
```

>CP121766.1 Pseudomonas aeruginosa strain 22112 chromosome, complete genome

```
product length = 306
Forward primer  1        TGGGTGATCGCCTTGGTGA  19
Template        6832005  ...................  6832023

Reverse primer  1        GGCCAGTTGCAGCTTGTTC  19
Template        6832310  ...................  6832292
```

>CP127016.1 Pseudomonas aeruginosa strain TBCF10839 chromosome

```
product length = 306
Forward primer  1        TGGGTGATCGCCTTGGTGA  19
Template        3853266  ...................  3853248

Reverse primer  1        GGCCAGTTGCAGCTTGTTC  19
Template        3852961  ...................  3852979
```

>CP123792.1 Pseudomonas aeruginosa strain 2021CK-01658 chromosome, complete genome

```
product length = 306
Forward primer  1        TGGGTGATCGCCTTGGTGA  19
Template        6600037  ...................  6600019

Reverse primer  1        GGCCAGTTGCAGCTTGTTC  19
Template        6599732  ...................  6599750
```

>CP109757.1 Pseudomonas aeruginosa strain 2017-45-85 chromosome, complete genome

```
product length = 306
Forward primer  1       TGGGTGATCGCCTTGGTGA  19
Template        268431  ...................  268449

Reverse primer  1       GGCCAGTTGCAGCTTGTTC  19
Template        268736  ...................  268718
```

>CP109683.1 Pseudomonas aeruginosa strain 2017-45-169 chromosome, complete genome

```
product length = 306
Forward primer  1        TGGGTGATCGCCTTGGTGA  19
Template        2209649  ...................  2209631

Reverse primer  1        GGCCAGTTGCAGCTTGTTC  19
Template        2209344  ...................  2209362
```

>CP109685.1 Pseudomonas aeruginosa strain 2017-45-137A chromosome, complete genome

```
product length = 306
Forward primer  1        TGGGTGATCGCCTTGGTGA  19
Template        5510433  ...................  5510451

Reverse primer  1        GGCCAGTTGCAGCTTGTTC  19
Template        5510738  ...................  5510720
```

>CP061073.2 Pseudomonas aeruginosa strain PAD8 chromosome, complete genome

```
product length = 306
Forward primer  1        TGGGTGATCGCCTTGGTGA  19
Template        3715661  ...................  3715679

Reverse primer  1        GGCCAGTTGCAGCTTGTTC  19
Template        3715966  ...................  3715948
```

>CP123786.1 Pseudomonas aeruginosa strain 2021CK-01424 chromosome, complete genome

```
product length = 306
Forward primer  1       TGGGTGATCGCCTTGGTGA  19
Template        646611  ...................  646629

Reverse primer  1       GGCCAGTTGCAGCTTGTTC  19
Template        646916  ...................  646898
```

>CP123785.1 Pseudomonas aeruginosa strain 2021CK-01267 chromosome, complete genome

```
product length = 306
Forward primer  1       TGGGTGATCGCCTTGGTGA  19
Template        646611  ...................  646629

Reverse primer  1       GGCCAGTTGCAGCTTGTTC  19
Template        646916  ...................  646898
```

>CP123787.1 Pseudomonas aeruginosa strain 2020CK-00194 chromosome, complete genome

```
product length = 306
Forward primer  1       TGGGTGATCGCCTTGGTGA  19
Template        606974  ...................  606992

Reverse primer  1       GGCCAGTTGCAGCTTGTTC  19
Template        607279  ...................  607261
```

>CP123789.1 Pseudomonas aeruginosa strain 2021CK-01381 chromosome, complete genome

```
product length = 306
Forward primer  1        TGGGTGATCGCCTTGGTGA  19
Template        6502424  ...................  6502406

Reverse primer  1        GGCCAGTTGCAGCTTGTTC  19
Template        6502119  ...................  6502137
```

>CP123791.1 Pseudomonas aeruginosa strain 2021CK-01305 chromosome, complete genome

```
product length = 306
Forward primer  1       TGGGTGATCGCCTTGGTGA  19
Template        607070  ...................  607088

Reverse primer  1       GGCCAGTTGCAGCTTGTTC  19
Template        607375  ...................  607357
```

>CP123793.1 Pseudomonas aeruginosa strain 2021CK-01107 chromosome, complete genome

```
product length = 306
Forward primer  1       TGGGTGATCGCCTTGGTGA  19
Template        606599  ...................  606617

Reverse primer  1       GGCCAGTTGCAGCTTGTTC  19
Template        606904  ...................  606886
```

>CP096964.1 Pseudomonas aeruginosa strain NY13936 chromosome, complete genome

```
product length = 306
Forward primer  1       TGGGTGATCGCCTTGGTGA  19
Template        474511  ...................  474529

Reverse primer  1       GGCCAGTTGCAGCTTGTTC  19
Template        474816  ...................  474798
```

>CP096961.1 Pseudomonas aeruginosa strain NY13932 chromosome, complete genome

```
product length = 306
Forward primer  1       TGGGTGATCGCCTTGGTGA  19
Template        467707  ...................  467725

Reverse primer  1       GGCCAGTTGCAGCTTGTTC  19
Template        468012  ...................  467994
```

>CP096960.1 Pseudomonas aeruginosa strain NY11254 chromosome, complete genome

```
product length = 306
Forward primer  1       TGGGTGATCGCCTTGGTGA  19
Template        510245  ...................  510263

Reverse primer  1       GGCCAGTTGCAGCTTGTTC  19
Template        510550  ...................  510532
```

>CP096958.1 Pseudomonas aeruginosa strain NY11210 chromosome, complete genome

```
product length = 306
Forward primer  1       TGGGTGATCGCCTTGGTGA  19
Template        513684  ...................  513702

Reverse primer  1       GGCCAGTTGCAGCTTGTTC  19
Template        513989  ...................  513971
```

>CP096956.1 Pseudomonas aeruginosa strain NY11173 chromosome, complete genome

```
product length = 306
Forward primer  1       TGGGTGATCGCCTTGGTGA  19
Template        472045  ...................  472063

Reverse primer  1       GGCCAGTTGCAGCTTGTTC  19
Template        472350  ...................  472332
```

>CP096953.1 Pseudomonas aeruginosa strain NY5535 chromosome, complete genome

```
product length = 306
Forward primer  1       TGGGTGATCGCCTTGGTGA  19
Template        513710  ...................  513728

Reverse primer  1       GGCCAGTTGCAGCTTGTTC  19
Template        514015  ...................  513997
```

>CP096950.1 Pseudomonas aeruginosa strain NY5532 chromosome, complete genome

```
product length = 306
Forward primer  1       TGGGTGATCGCCTTGGTGA  19
Template        484680  ...................  484698

Reverse primer  1       GGCCAGTTGCAGCTTGTTC  19
Template        484985  ...................  484967
```

>CP096946.1 Pseudomonas aeruginosa strain NY5530 chromosome, complete genome

```
product length = 306
Forward primer  1       TGGGTGATCGCCTTGGTGA  19
Template        513428  ...................  513446

Reverse primer  1       GGCCAGTTGCAGCTTGTTC  19
Template        513733  ...................  513715
```

>CP096945.1 Pseudomonas aeruginosa strain NY5525 chromosome, complete genome

```
product length = 306
Forward primer  1       TGGGTGATCGCCTTGGTGA  19
Template        523521  ...................  523539

Reverse primer  1       GGCCAGTTGCAGCTTGTTC  19
Template        523826  ...................  523808
```

>CP096942.1 Pseudomonas aeruginosa strain NY5524 chromosome, complete genome

```
product length = 306
Forward primer  1       TGGGTGATCGCCTTGGTGA  19
Template        521660  ...................  521678

Reverse primer  1       GGCCAGTTGCAGCTTGTTC  19
Template        521965  ...................  521947
```

>CP096941.1 Pseudomonas aeruginosa strain NY5523 chromosome, complete genome

```
product length = 306
Forward primer  1       TGGGTGATCGCCTTGGTGA  19
Template        510247  ...................  510265

Reverse primer  1       GGCCAGTTGCAGCTTGTTC  19
Template        510552  ...................  510534
```

>CP096937.1 Pseudomonas aeruginosa strain NY5520 chromosome, complete genome

```
product length = 306
Forward primer  1       TGGGTGATCGCCTTGGTGA  19
Template        513410  ...................  513428

Reverse primer  1       GGCCAGTTGCAGCTTGTTC  19
Template        513715  ...................  513697
```

>CP096934.1 Pseudomonas aeruginosa strain NY5511 chromosome, complete genome

```
product length = 306
Forward primer  1       TGGGTGATCGCCTTGGTGA  19
Template        513709  ...................  513727

Reverse primer  1       GGCCAGTTGCAGCTTGTTC  19
Template        514014  ...................  513996
```

>CP096932.1 Pseudomonas aeruginosa strain NY5510 chromosome, complete genome

```
product length = 306
Forward primer  1       TGGGTGATCGCCTTGGTGA  19
Template        513709  ...................  513727

Reverse primer  1       GGCCAGTTGCAGCTTGTTC  19
Template        514014  ...................  513996
```

>CP096929.1 Pseudomonas aeruginosa strain NY5507 chromosome, complete genome

```
product length = 306
Forward primer  1       TGGGTGATCGCCTTGGTGA  19
Template        513691  ...................  513709

Reverse primer  1       GGCCAGTTGCAGCTTGTTC  19
Template        513996  ...................  513978
```

>CP096927.1 Pseudomonas aeruginosa strain NY5506 chromosome, complete genome

```
product length = 306
Forward primer  1       TGGGTGATCGCCTTGGTGA  19
Template        469717  ...................  469735

Reverse primer  1       GGCCAGTTGCAGCTTGTTC  19
Template        470022  ...................  470004
```

>CP124673.1 Pseudomonas aeruginosa strain 2022CK-00491 chromosome, complete genome

```
product length = 306
Forward primer  1       TGGGTGATCGCCTTGGTGA  19
Template        608334  ...................  608352

Reverse primer  1       GGCCAGTTGCAGCTTGTTC  19
Template        608639  ...................  608621
```

>CP124674.1 Pseudomonas aeruginosa strain 2022CK-00339 chromosome, complete genome

```
product length = 306
Forward primer  1       TGGGTGATCGCCTTGGTGA  19
Template        469371  ...................  469389

Reverse primer  1       GGCCAGTTGCAGCTTGTTC  19
Template        469676  ...................  469658
```

>CP084890.1 Pseudomonas aeruginosa strain CH1 chromosome

```
product length = 306
Forward primer  1        TGGGTGATCGCCTTGGTGA  19
Template        1858631  ...................  1858613

Reverse primer  1        GGCCAGTTGCAGCTTGTTC  19
Template        1858326  ...................  1858344
```

>CP124658.1 Pseudomonas aeruginosa strain 2022CK-00068 chromosome, complete genome

```
product length = 306
Forward primer  1       TGGGTGATCGCCTTGGTGA  19
Template        474037  ...................  474055

Reverse primer  1       GGCCAGTTGCAGCTTGTTC  19
Template        474342  ...................  474324
```

>CP124662.1 Pseudomonas aeruginosa strain 2021CK-01633 chromosome, complete genome

```
product length = 306
Forward primer  1       TGGGTGATCGCCTTGGTGA  19
Template        606974  ...................  606992

Reverse primer  1       GGCCAGTTGCAGCTTGTTC  19
Template        607279  ...................  607261
```

>CP124660.1 Pseudomonas aeruginosa strain 2022CK-00160 chromosome, complete genome

```
product length = 306
Forward primer  1       TGGGTGATCGCCTTGGTGA  19
Template        525067  ...................  525085

Reverse primer  1       GGCCAGTTGCAGCTTGTTC  19
Template        525372  ...................  525354
```

>CP124652.1 Pseudomonas aeruginosa strain 2020CK-00443 chromosome, complete genome

```
product length = 306
Forward primer  1        TGGGTGATCGCCTTGGTGA  19
Template        6307575  ...................  6307557

Reverse primer  1        GGCCAGTTGCAGCTTGTTC  19
Template        6307270  ...................  6307288
```

>CP124649.1 Pseudomonas aeruginosa strain 2020CK-00218 chromosome, complete genome

```
product length = 306
Forward primer  1       TGGGTGATCGCCTTGGTGA  19
Template        549208  ...................  549226

Reverse primer  1       GGCCAGTTGCAGCTTGTTC  19
Template        549513  ...................  549495
```

>CP124664.1 Pseudomonas aeruginosa strain 2021CK-01256 chromosome, complete genome

```
product length = 306
Forward primer  1       TGGGTGATCGCCTTGGTGA  19
Template        458024  ...................  458042

Reverse primer  1       GGCCAGTTGCAGCTTGTTC  19
Template        458329  ...................  458311
```

>CP124655.1 Pseudomonas aeruginosa strain 2022CK-00096 chromosome, complete genome

```
product length = 306
Forward primer  1        TGGGTGATCGCCTTGGTGA  19
Template        6387636  ...................  6387618

Reverse primer  1        GGCCAGTTGCAGCTTGTTC  19
Template        6387331  ...................  6387349
```

>CP124654.1 Pseudomonas aeruginosa strain 2021CK-01851 chromosome, complete genome

```
product length = 306
Forward primer  1       TGGGTGATCGCCTTGGTGA  19
Template        456110  ...................  456128

Reverse primer  1       GGCCAGTTGCAGCTTGTTC  19
Template        456415  ...................  456397
```

>CP124657.1 Pseudomonas aeruginosa strain 2022CK-00069 chromosome, complete genome

```
product length = 306
Forward primer  1       TGGGTGATCGCCTTGGTGA  19
Template        548878  ...................  548896

Reverse primer  1       GGCCAGTTGCAGCTTGTTC  19
Template        549183  ...................  549165
```

>CP124651.1 Pseudomonas aeruginosa strain 2020CK-00217 chromosome, complete genome

```
product length = 306
Forward primer  1       TGGGTGATCGCCTTGGTGA  19
Template        606974  ...................  606992

Reverse primer  1       GGCCAGTTGCAGCTTGTTC  19
Template        607279  ...................  607261
```

>CP124669.1 Pseudomonas aeruginosa strain 2021CK-01494 chromosome, complete genome

```
product length = 306
Forward primer  1       TGGGTGATCGCCTTGGTGA  19
Template        464218  ...................  464236

Reverse primer  1       GGCCAGTTGCAGCTTGTTC  19
Template        464523  ...................  464505
```

>CP124638.1 Pseudomonas aeruginosa strain 2021CK-01158 chromosome, complete genome

```
product length = 306
Forward primer  1       TGGGTGATCGCCTTGGTGA  19
Template        472793  ...................  472811

Reverse primer  1       GGCCAGTTGCAGCTTGTTC  19
Template        473098  ...................  473080
```

>CP124668.1 Pseudomonas aeruginosa strain 2021CK-01445 chromosome, complete genome

```
product length = 306
Forward primer  1       TGGGTGATCGCCTTGGTGA  19
Template        464214  ...................  464232

Reverse primer  1       GGCCAGTTGCAGCTTGTTC  19
Template        464519  ...................  464501
```

>CP124666.1 Pseudomonas aeruginosa strain 2021CK-01283 chromosome, complete genome

```
product length = 306
Forward primer  1       TGGGTGATCGCCTTGGTGA  19
Template        486657  ...................  486675

Reverse primer  1       GGCCAGTTGCAGCTTGTTC  19
Template        486962  ...................  486944
```

>CP124624.1 Pseudomonas aeruginosa strain 2021CK-01157 chromosome, complete genome

```
product length = 306
Forward primer  1       TGGGTGATCGCCTTGGTGA  19
Template        472795  ...................  472813

Reverse primer  1       GGCCAGTTGCAGCTTGTTC  19
Template        473100  ...................  473082
```

>CP124665.1 Pseudomonas aeruginosa strain 2021CK-01229 chromosome, complete genome

```
product length = 306
Forward primer  1       TGGGTGATCGCCTTGGTGA  19
Template        486657  ...................  486675

Reverse primer  1       GGCCAGTTGCAGCTTGTTC  19
Template        486962  ...................  486944
```

>CP124667.1 Pseudomonas aeruginosa strain 2021CK-01315 chromosome, complete genome

```
product length = 306
Forward primer  1       TGGGTGATCGCCTTGGTGA  19
Template        481044  ...................  481062

Reverse primer  1       GGCCAGTTGCAGCTTGTTC  19
Template        481349  ...................  481331
```

>CP124641.1 Pseudomonas aeruginosa strain 2021CK-01198 chromosome, complete genome

```
product length = 306
Forward primer  1       TGGGTGATCGCCTTGGTGA  19
Template        472798  ...................  472816

Reverse primer  1       GGCCAGTTGCAGCTTGTTC  19
Template        473103  ...................  473085
```

>CP124670.1 Pseudomonas aeruginosa strain 2021CK-01536 chromosome, complete genome

```
product length = 306
Forward primer  1       TGGGTGATCGCCTTGGTGA  19
Template        476491  ...................  476509

Reverse primer  1       GGCCAGTTGCAGCTTGTTC  19
Template        476796  ...................  476778
```

>CP124646.1 Pseudomonas aeruginosa strain 2020CK-00185 chromosome, complete genome

```
product length = 306
Forward primer  1       TGGGTGATCGCCTTGGTGA  19
Template        489722  ...................  489740

Reverse primer  1       GGCCAGTTGCAGCTTGTTC  19
Template        490027  ...................  490009
```

>CP124648.1 Pseudomonas aeruginosa strain 2020CK-00220 chromosome, complete genome

```
product length = 306
Forward primer  1        TGGGTGATCGCCTTGGTGA  19
Template        2981255  ...................  2981273

Reverse primer  1        GGCCAGTTGCAGCTTGTTC  19
Template        2981560  ...................  2981542
```

>CP124643.1 Pseudomonas aeruginosa strain 2021CK-01197 chromosome, complete genome

```
product length = 306
Forward primer  1        TGGGTGATCGCCTTGGTGA  19
Template        4267513  ...................  4267495

Reverse primer  1        GGCCAGTTGCAGCTTGTTC  19
Template        4267208  ...................  4267226
```

>CP124626.1 Pseudomonas aeruginosa strain 2021CK-01161 chromosome, complete genome

```
product length = 306
Forward primer  1       TGGGTGATCGCCTTGGTGA  19
Template        386236  ...................  386218

Reverse primer  1       GGCCAGTTGCAGCTTGTTC  19
Template        385931  ...................  385949
```

>CP124632.1 Pseudomonas aeruginosa strain 2021CK-01162 chromosome, complete genome

```
product length = 306
Forward primer  1       TGGGTGATCGCCTTGGTGA  19
Template        891709  ...................  891727

Reverse primer  1       GGCCAGTTGCAGCTTGTTC  19
Template        892014  ...................  891996
```

>CP124663.1 Pseudomonas aeruginosa strain 2021CK-01227 chromosome, complete genome

```
product length = 306
Forward primer  1       TGGGTGATCGCCTTGGTGA  19
Template        486657  ...................  486675

Reverse primer  1       GGCCAGTTGCAGCTTGTTC  19
Template        486962  ...................  486944
```

>CP124622.1 Pseudomonas aeruginosa strain 2021CK-01159 chromosome, complete genome

```
product length = 306
Forward primer  1       TGGGTGATCGCCTTGGTGA  19
Template        481044  ...................  481062

Reverse primer  1       GGCCAGTTGCAGCTTGTTC  19
Template        481349  ...................  481331
```

>CP124600.1 Pseudomonas aeruginosa strain Li010 chromosome, complete genome

```
product length = 306
Forward primer  1       TGGGTGATCGCCTTGGTGA  19
Template        478033  ...................  478051

Reverse primer  1       GGCCAGTTGCAGCTTGTTC  19
Template        478338  ...................  478320
```

>CP123953.1 Pseudomonas aeruginosa strain 59 chromosome, complete genome

```
product length = 306
Forward primer  1       TGGGTGATCGCCTTGGTGA  19
Template        468657  ...................  468675

Reverse primer  1       GGCCAGTTGCAGCTTGTTC  19
Template        468962  ...................  468944
```

>CP116682.1 Pseudomonas aeruginosa strain HS337 chromosome, complete genome

```
product length = 306
Forward primer  1       TGGGTGATCGCCTTGGTGA  19
Template        484608  ...................  484626

Reverse primer  1       GGCCAGTTGCAGCTTGTTC  19
Template        484913  ...................  484895
```

>CP110190.1 Pseudomonas aeruginosa strain HS204 chromosome, complete genome

```
product length = 306
Forward primer  1       TGGGTGATCGCCTTGGTGA  19
Template        481128  ...................  481146

Reverse primer  1       GGCCAGTTGCAGCTTGTTC  19
Template        481433  ...................  481415
```

>CP118638.1 Pseudomonas aeruginosa strain P9 chromosome, complete genome

```
product length = 306
Forward primer  1       TGGGTGATCGCCTTGGTGA  19
Template        466918  ...................  466936

Reverse primer  1       GGCCAGTTGCAGCTTGTTC  19
Template        467223  ...................  467205
```

>CP118641.1 Pseudomonas aeruginosa strain P23 chromosome, complete genome

```
product length = 306
Forward primer  1       TGGGTGATCGCCTTGGTGA  19
Template        481577  ...................  481595

Reverse primer  1       GGCCAGTTGCAGCTTGTTC  19
Template        481882  ...................  481864
```

>CP119298.1 Pseudomonas aeruginosa strain SNDPR-01 chromosome, complete genome

```
product length = 306
Forward primer  1       TGGGTGATCGCCTTGGTGA  19
Template        498942  ...................  498960

Reverse primer  1       GGCCAGTTGCAGCTTGTTC  19
Template        499247  ...................  499229
```

>CP117300.1 Pseudomonas aeruginosa strain 0201761-1 chromosome, complete genome

```
product length = 306
Forward primer  1       TGGGTGATCGCCTTGGTGA  19
Template        474528  ...................  474546

Reverse primer  1       GGCCAGTTGCAGCTTGTTC  19
Template        474833  ...................  474815
```

>CP084321.1 Pseudomonas aeruginosa strain HS18-89 chromosome, complete genome

```
product length = 306
Forward primer  1       TGGGTGATCGCCTTGGTGA  19
Template        506144  ...................  506162

Reverse primer  1       GGCCAGTTGCAGCTTGTTC  19
Template        506449  ...................  506431
```

>CP117974.1 Pseudomonas aeruginosa strain B-3509 chromosome, complete genome

```
product length = 306
Forward primer  1        TGGGTGATCGCCTTGGTGA  19
Template        5070027  ...................  5070009

Reverse primer  1        GGCCAGTTGCAGCTTGTTC  19
Template        5069722  ...................  5069740
```

>CP117749.1 Pseudomonas aeruginosa strain 2022CK-00828 chromosome, complete genome

```
product length = 306
Forward primer  1       TGGGTGATCGCCTTGGTGA  19
Template        463952  ...................  463970

Reverse primer  1       GGCCAGTTGCAGCTTGTTC  19
Template        464257  ...................  464239
```

>CP117527.1 Pseudomonas aeruginosa strain MF1 chromosome, complete genome

```
product length = 306
Forward primer  1       TGGGTGATCGCCTTGGTGA  19
Template        551763  ...................  551781

Reverse primer  1       GGCCAGTTGCAGCTTGTTC  19
Template        552068  ...................  552050
```

>CP075851.1 Pseudomonas aeruginosa strain PaLo33 chromosome, complete genome

```
product length = 306
Forward primer  1       TGGGTGATCGCCTTGGTGA  19
Template        518503  ...................  518521

Reverse primer  1       GGCCAGTTGCAGCTTGTTC  19
Template        518808  ...................  518790
```

>CP075849.1 Pseudomonas aeruginosa strain PaLo1 chromosome, complete genome

```
product length = 306
Forward primer  1       TGGGTGATCGCCTTGGTGA  19
Template        488316  ...................  488334

Reverse primer  1       GGCCAGTTGCAGCTTGTTC  19
Template        488621  ...................  488603
```

>CP075848.1 Pseudomonas aeruginosa strain PaLo2 chromosome, complete genome

```
product length = 306
Forward primer  1       TGGGTGATCGCCTTGGTGA  19
Template        469434  ...................  469452

Reverse primer  1       GGCCAGTTGCAGCTTGTTC  19
Template        469739  ...................  469721
```

>CP075846.1 Pseudomonas aeruginosa strain PaLo4 chromosome

```
product length = 306
Forward primer  1       TGGGTGATCGCCTTGGTGA  19
Template        367728  ...................  367746

Reverse primer  1       GGCCAGTTGCAGCTTGTTC  19
Template        368033  ...................  368015
```

>CP075844.1 Pseudomonas aeruginosa strain PaLo6 chromosome, complete genome

```
product length = 306
Forward primer  1       TGGGTGATCGCCTTGGTGA  19
Template        473720  ...................  473738

Reverse primer  1       GGCCAGTTGCAGCTTGTTC  19
Template        474025  ...................  474007
```

>CP075843.1 Pseudomonas aeruginosa strain PaLo7 chromosome

```
product length = 306
Forward primer  1       TGGGTGATCGCCTTGGTGA  19
Template        507167  ...................  507185

Reverse primer  1       GGCCAGTTGCAGCTTGTTC  19
Template        507472  ...................  507454
```

>CP075841.1 Pseudomonas aeruginosa strain PaLo9 chromosome, complete genome

```
product length = 306
Forward primer  1       TGGGTGATCGCCTTGGTGA  19
Template        467427  ...................  467445

Reverse primer  1       GGCCAGTTGCAGCTTGTTC  19
Template        467732  ...................  467714
```

>CP075840.1 Pseudomonas aeruginosa strain PaLo10 chromosome, complete genome

```
product length = 306
Forward primer  1       TGGGTGATCGCCTTGGTGA  19
Template        466563  ...................  466581

Reverse primer  1       GGCCAGTTGCAGCTTGTTC  19
Template        466868  ...................  466850
```

>CP075838.1 Pseudomonas aeruginosa strain PaLo11 chromosome, complete genome

```
product length = 306
Forward primer  1       TGGGTGATCGCCTTGGTGA  19
Template        467766  ...................  467784

Reverse primer  1       GGCCAGTTGCAGCTTGTTC  19
Template        468071  ...................  468053
```

>CP075836.1 Pseudomonas aeruginosa strain PaLo12 chromosome, complete genome

```
product length = 306
Forward primer  1       TGGGTGATCGCCTTGGTGA  19
Template        469875  ...................  469893

Reverse primer  1       GGCCAGTTGCAGCTTGTTC  19
Template        470180  ...................  470162
```

>CP075835.1 Pseudomonas aeruginosa strain PaLo14 chromosome, complete genome

```
product length = 306
Forward primer  1       TGGGTGATCGCCTTGGTGA  19
Template        518393  ...................  518411

Reverse primer  1       GGCCAGTTGCAGCTTGTTC  19
Template        518698  ...................  518680
```

>CP075834.1 Pseudomonas aeruginosa strain PaLo15 chromosome, complete genome

```
product length = 306
Forward primer  1       TGGGTGATCGCCTTGGTGA  19
Template        515054  ...................  515072

Reverse primer  1       GGCCAGTTGCAGCTTGTTC  19
Template        515359  ...................  515341
```

>CP075833.1 Pseudomonas aeruginosa strain PaLo17 chromosome, complete genome

```
product length = 306
Forward primer  1       TGGGTGATCGCCTTGGTGA  19
Template        469392  ...................  469410

Reverse primer  1       GGCCAGTTGCAGCTTGTTC  19
Template        469697  ...................  469679
```

>CP075832.1 Pseudomonas aeruginosa strain PaLo20 chromosome, complete genome

```
product length = 306
Forward primer  1       TGGGTGATCGCCTTGGTGA  19
Template        471157  ...................  471175

Reverse primer  1       GGCCAGTTGCAGCTTGTTC  19
Template        471462  ...................  471444
```

>CP075831.1 Pseudomonas aeruginosa strain PaLo21 chromosome, complete genome

```
product length = 306
Forward primer  1       TGGGTGATCGCCTTGGTGA  19
Template        467572  ...................  467590

Reverse primer  1       GGCCAGTTGCAGCTTGTTC  19
Template        467877  ...................  467859
```

>CP075830.1 Pseudomonas aeruginosa strain PaLo22 chromosome, complete genome

```
product length = 306
Forward primer  1       TGGGTGATCGCCTTGGTGA  19
Template        465390  ...................  465408

Reverse primer  1       GGCCAGTTGCAGCTTGTTC  19
Template        465695  ...................  465677
```

>CP075829.1 Pseudomonas aeruginosa strain PaLo25 chromosome, complete genome

```
product length = 306
Forward primer  1       TGGGTGATCGCCTTGGTGA  19
Template        476752  ...................  476770

Reverse primer  1       GGCCAGTTGCAGCTTGTTC  19
Template        477057  ...................  477039
```

>CP075828.1 Pseudomonas aeruginosa strain PaLo26 chromosome, complete genome

```
product length = 306
Forward primer  1       TGGGTGATCGCCTTGGTGA  19
Template        470852  ...................  470870

Reverse primer  1       GGCCAGTTGCAGCTTGTTC  19
Template        471157  ...................  471139
```

>CP075827.1 Pseudomonas aeruginosa strain PaLo27 chromosome, complete genome

```
product length = 306
Forward primer  1       TGGGTGATCGCCTTGGTGA  19
Template        533789  ...................  533807

Reverse primer  1       GGCCAGTTGCAGCTTGTTC  19
Template        534094  ...................  534076
```

>CP075826.1 Pseudomonas aeruginosa strain PaLo29 chromosome, complete genome

```
product length = 306
Forward primer  1       TGGGTGATCGCCTTGGTGA  19
Template        422064  ...................  422082

Reverse primer  1       GGCCAGTTGCAGCTTGTTC  19
Template        422369  ...................  422351
```

>CP075825.1 Pseudomonas aeruginosa strain PaLo30 chromosome, complete genome

```
product length = 306
Forward primer  1       TGGGTGATCGCCTTGGTGA  19
Template        469424  ...................  469442

Reverse primer  1       GGCCAGTTGCAGCTTGTTC  19
Template        469729  ...................  469711
```

>CP075824.1 Pseudomonas aeruginosa strain PaLo31 chromosome, complete genome

```
product length = 306
Forward primer  1       TGGGTGATCGCCTTGGTGA  19
Template        508107  ...................  508125

Reverse primer  1       GGCCAGTTGCAGCTTGTTC  19
Template        508412  ...................  508394
```

>CP075823.1 Pseudomonas aeruginosa strain PaLo32 chromosome, complete genome

```
product length = 306
Forward primer  1       TGGGTGATCGCCTTGGTGA  19
Template        518496  ...................  518514

Reverse primer  1       GGCCAGTTGCAGCTTGTTC  19
Template        518801  ...................  518783
```

>CP075822.1 Pseudomonas aeruginosa strain PaLo34 chromosome, complete genome

```
product length = 306
Forward primer  1       TGGGTGATCGCCTTGGTGA  19
Template        469424  ...................  469442

Reverse primer  1       GGCCAGTTGCAGCTTGTTC  19
Template        469729  ...................  469711
```

>CP075821.1 Pseudomonas aeruginosa strain PaLo35 chromosome, complete genome

```
product length = 306
Forward primer  1       TGGGTGATCGCCTTGGTGA  19
Template        464881  ...................  464899

Reverse primer  1       GGCCAGTTGCAGCTTGTTC  19
Template        465186  ...................  465168
```

>CP075820.1 Pseudomonas aeruginosa strain PaLo36 chromosome, complete genome

```
product length = 306
Forward primer  1       TGGGTGATCGCCTTGGTGA  19
Template        467844  ...................  467862

Reverse primer  1       GGCCAGTTGCAGCTTGTTC  19
Template        468149  ...................  468131
```

>CP075819.1 Pseudomonas aeruginosa strain PaLo37 chromosome, complete genome

```
product length = 306
Forward primer  1       TGGGTGATCGCCTTGGTGA  19
Template        469386  ...................  469404

Reverse primer  1       GGCCAGTTGCAGCTTGTTC  19
Template        469691  ...................  469673
```

>CP075818.1 Pseudomonas aeruginosa strain PaLo38 chromosome, complete genome

```
product length = 306
Forward primer  1       TGGGTGATCGCCTTGGTGA  19
Template        469417  ...................  469435

Reverse primer  1       GGCCAGTTGCAGCTTGTTC  19
Template        469722  ...................  469704
```

>CP075817.1 Pseudomonas aeruginosa strain PaLo39 chromosome, complete genome

```
product length = 306
Forward primer  1       TGGGTGATCGCCTTGGTGA  19
Template        477729  ...................  477747

Reverse primer  1       GGCCAGTTGCAGCTTGTTC  19
Template        478034  ...................  478016
```

>CP075816.1 Pseudomonas aeruginosa strain PaLo40 chromosome, complete genome

```
product length = 306
Forward primer  1       TGGGTGATCGCCTTGGTGA  19
Template        469426  ...................  469444

Reverse primer  1       GGCCAGTTGCAGCTTGTTC  19
Template        469731  ...................  469713
```

>CP075815.1 Pseudomonas aeruginosa strain PaLo43 chromosome, complete genome

```
product length = 306
Forward primer  1       TGGGTGATCGCCTTGGTGA  19
Template        469329  ...................  469347

Reverse primer  1       GGCCAGTTGCAGCTTGTTC  19
Template        469634  ...................  469616
```

>CP075814.1 Pseudomonas aeruginosa strain PaLo44 chromosome, complete genome

```
product length = 306
Forward primer  1       TGGGTGATCGCCTTGGTGA  19
Template        477736  ...................  477754

Reverse primer  1       GGCCAGTTGCAGCTTGTTC  19
Template        478041  ...................  478023
```

>CP075813.1 Pseudomonas aeruginosa strain PaLo45 chromosome, complete genome

```
product length = 306
Forward primer  1       TGGGTGATCGCCTTGGTGA  19
Template        466365  ...................  466383

Reverse primer  1       GGCCAGTTGCAGCTTGTTC  19
Template        466670  ...................  466652
```

>CP075812.1 Pseudomonas aeruginosa strain PaLo46 chromosome, complete genome

```
product length = 306
Forward primer  1       TGGGTGATCGCCTTGGTGA  19
Template        518443  ...................  518461

Reverse primer  1       GGCCAGTTGCAGCTTGTTC  19
Template        518748  ...................  518730
```

>CP075811.1 Pseudomonas aeruginosa strain PaLo152 chromosome, complete genome

```
product length = 306
Forward primer  1       TGGGTGATCGCCTTGGTGA  19
Template        470251  ...................  470269

Reverse primer  1       GGCCAGTTGCAGCTTGTTC  19
Template        470556  ...................  470538
```

>CP075810.1 Pseudomonas aeruginosa strain PaLo166 chromosome, complete genome

```
product length = 306
Forward primer  1       TGGGTGATCGCCTTGGTGA  19
Template        476872  ...................  476890

Reverse primer  1       GGCCAGTTGCAGCTTGTTC  19
Template        477177  ...................  477159
```

>CP075809.1 Pseudomonas aeruginosa strain PaLo170 chromosome, complete genome

```
product length = 306
Forward primer  1       TGGGTGATCGCCTTGGTGA  19
Template        467950  ...................  467968

Reverse primer  1       GGCCAGTTGCAGCTTGTTC  19
Template        468255  ...................  468237
```

>CP075808.1 Pseudomonas aeruginosa strain PaLo185 chromosome

```
product length = 306
Forward primer  1       TGGGTGATCGCCTTGGTGA  19
Template        468421  ...................  468439

Reverse primer  1       GGCCAGTTGCAGCTTGTTC  19
Template        468726  ...................  468708
```

>CP075807.1 Pseudomonas aeruginosa strain PaLo191 chromosome, complete genome

```
product length = 306
Forward primer  1       TGGGTGATCGCCTTGGTGA  19
Template        466672  ...................  466690

Reverse primer  1       GGCCAGTTGCAGCTTGTTC  19
Template        466977  ...................  466959
```

>CP075806.1 Pseudomonas aeruginosa strain PaLo226 chromosome, complete genome

```
product length = 306
Forward primer  1       TGGGTGATCGCCTTGGTGA  19
Template        465632  ...................  465650

Reverse primer  1       GGCCAGTTGCAGCTTGTTC  19
Template        465937  ...................  465919
```

>CP075805.1 Pseudomonas aeruginosa strain PaLo227 chromosome, complete genome

```
product length = 306
Forward primer  1       TGGGTGATCGCCTTGGTGA  19
Template        465643  ...................  465661

Reverse primer  1       GGCCAGTTGCAGCTTGTTC  19
Template        465948  ...................  465930
```

>CP075804.1 Pseudomonas aeruginosa strain PaLo228 chromosome, complete genome

```
product length = 306
Forward primer  1       TGGGTGATCGCCTTGGTGA  19
Template        465632  ...................  465650

Reverse primer  1       GGCCAGTTGCAGCTTGTTC  19
Template        465937  ...................  465919
```

>CP075803.1 Pseudomonas aeruginosa strain PaLo229 chromosome, complete genome

```
product length = 306
Forward primer  1       TGGGTGATCGCCTTGGTGA  19
Template        465640  ...................  465658

Reverse primer  1       GGCCAGTTGCAGCTTGTTC  19
Template        465945  ...................  465927
```

>CP075802.1 Pseudomonas aeruginosa strain PaLo240 chromosome, complete genome

```
product length = 306
Forward primer  1       TGGGTGATCGCCTTGGTGA  19
Template        518514  ...................  518532

Reverse primer  1       GGCCAGTTGCAGCTTGTTC  19
Template        518819  ...................  518801
```

>CP075801.1 Pseudomonas aeruginosa strain PaLo249 chromosome, complete genome

```
product length = 306
Forward primer  1       TGGGTGATCGCCTTGGTGA  19
Template        465637  ...................  465655

Reverse primer  1       GGCCAGTTGCAGCTTGTTC  19
Template        465942  ...................  465924
```

>CP075800.1 Pseudomonas aeruginosa strain PaLo297 chromosome, complete genome

```
product length = 306
Forward primer  1       TGGGTGATCGCCTTGGTGA  19
Template        467206  ...................  467224

Reverse primer  1       GGCCAGTTGCAGCTTGTTC  19
Template        467511  ...................  467493
```

>CP075799.1 Pseudomonas aeruginosa strain PaLo310 chromosome, complete genome

```
product length = 306
Forward primer  1       TGGGTGATCGCCTTGGTGA  19
Template        469428  ...................  469446

Reverse primer  1       GGCCAGTTGCAGCTTGTTC  19
Template        469733  ...................  469715
```

>CP075798.1 Pseudomonas aeruginosa strain PaLo323 chromosome, complete genome

```
product length = 306
Forward primer  1       TGGGTGATCGCCTTGGTGA  19
Template        466717  ...................  466735

Reverse primer  1       GGCCAGTTGCAGCTTGTTC  19
Template        467022  ...................  467004
```

>CP075797.1 Pseudomonas aeruginosa strain PaLo326 chromosome, complete genome

```
product length = 306
Forward primer  1       TGGGTGATCGCCTTGGTGA  19
Template        474313  ...................  474331

Reverse primer  1       GGCCAGTTGCAGCTTGTTC  19
Template        474618  ...................  474600
```

>CP075796.1 Pseudomonas aeruginosa strain PaLo402 chromosome, complete genome

```
product length = 306
Forward primer  1       TGGGTGATCGCCTTGGTGA  19
Template        455425  ...................  455443

Reverse primer  1       GGCCAGTTGCAGCTTGTTC  19
Template        455730  ...................  455712
```

>CP075794.1 Pseudomonas aeruginosa strain PaLo418 chromosome, complete genome

```
product length = 306
Forward primer  1       TGGGTGATCGCCTTGGTGA  19
Template        466758  ...................  466776

Reverse primer  1       GGCCAGTTGCAGCTTGTTC  19
Template        467063  ...................  467045
```

>CP075793.1 Pseudomonas aeruginosa strain PaLo419 chromosome, complete genome

```
product length = 306
Forward primer  1       TGGGTGATCGCCTTGGTGA  19
Template        466758  ...................  466776

Reverse primer  1       GGCCAGTTGCAGCTTGTTC  19
Template        467063  ...................  467045
```

>CP075792.1 Pseudomonas aeruginosa strain PaLo422 chromosome, complete genome

```
product length = 306
Forward primer  1       TGGGTGATCGCCTTGGTGA  19
Template        532871  ...................  532889

Reverse primer  1       GGCCAGTTGCAGCTTGTTC  19
Template        533176  ...................  533158
```

>CP075788.1 Pseudomonas aeruginosa strain PaLo502 chromosome, complete genome

```
product length = 306
Forward primer  1       TGGGTGATCGCCTTGGTGA  19
Template        450150  ...................  450168

Reverse primer  1       GGCCAGTTGCAGCTTGTTC  19
Template        450455  ...................  450437
```

>CP075787.1 Pseudomonas aeruginosa strain PaLo504 chromosome, complete genome

```
product length = 306
Forward primer  1       TGGGTGATCGCCTTGGTGA  19
Template        480082  ...................  480100

Reverse primer  1       GGCCAGTTGCAGCTTGTTC  19
Template        480387  ...................  480369
```

>CP075785.1 Pseudomonas aeruginosa strain PaLo505 chromosome, complete genome

```
product length = 306
Forward primer  1       TGGGTGATCGCCTTGGTGA  19
Template        472409  ...................  472427

Reverse primer  1       GGCCAGTTGCAGCTTGTTC  19
Template        472714  ...................  472696
```

>CP075784.1 Pseudomonas aeruginosa strain PaLo507 chromosome, complete genome

```
product length = 306
Forward primer  1       TGGGTGATCGCCTTGGTGA  19
Template        470647  ...................  470665

Reverse primer  1       GGCCAGTTGCAGCTTGTTC  19
Template        470952  ...................  470934
```

>CP075783.1 Pseudomonas aeruginosa strain PaLo508 chromosome, complete genome

```
product length = 306
Forward primer  1       TGGGTGATCGCCTTGGTGA  19
Template        474321  ...................  474339

Reverse primer  1       GGCCAGTTGCAGCTTGTTC  19
Template        474626  ...................  474608
```

>CP075782.1 Pseudomonas aeruginosa strain PaLo509 chromosome, complete genome

```
product length = 306
Forward primer  1       TGGGTGATCGCCTTGGTGA  19
Template        474285  ...................  474303

Reverse primer  1       GGCCAGTTGCAGCTTGTTC  19
Template        474590  ...................  474572
```

>CP075781.1 Pseudomonas aeruginosa strain PaLo512 chromosome, complete genome

```
product length = 306
Forward primer  1       TGGGTGATCGCCTTGGTGA  19
Template        467866  ...................  467884

Reverse primer  1       GGCCAGTTGCAGCTTGTTC  19
Template        468171  ...................  468153
```

>CP075780.1 Pseudomonas aeruginosa strain PaLo524 chromosome, complete genome

```
product length = 306
Forward primer  1       TGGGTGATCGCCTTGGTGA  19
Template        465833  ...................  465851

Reverse primer  1       GGCCAGTTGCAGCTTGTTC  19
Template        466138  ...................  466120
```

>CP075779.1 Pseudomonas aeruginosa strain PaLo526 chromosome, complete genome

```
product length = 306
Forward primer  1       TGGGTGATCGCCTTGGTGA  19
Template        470318  ...................  470336

Reverse primer  1       GGCCAGTTGCAGCTTGTTC  19
Template        470623  ...................  470605
```

>CP075778.1 Pseudomonas aeruginosa strain PaLo527 chromosome, complete genome

```
product length = 306
Forward primer  1       TGGGTGATCGCCTTGGTGA  19
Template        466935  ...................  466953

Reverse primer  1       GGCCAGTTGCAGCTTGTTC  19
Template        467240  ...................  467222
```

>CP075777.1 Pseudomonas aeruginosa strain PaLo528 chromosome, complete genome

```
product length = 306
Forward primer  1       TGGGTGATCGCCTTGGTGA  19
Template        472287  ...................  472305

Reverse primer  1       GGCCAGTTGCAGCTTGTTC  19
Template        472592  ...................  472574
```

>CP075776.1 Pseudomonas aeruginosa strain PaLo529 chromosome, complete genome

```
product length = 306
Forward primer  1       TGGGTGATCGCCTTGGTGA  19
Template        479037  ...................  479055

Reverse primer  1       GGCCAGTTGCAGCTTGTTC  19
Template        479342  ...................  479324
```

>CP075773.1 Pseudomonas aeruginosa strain PaLo530 chromosome, complete genome

```
product length = 306
Forward primer  1       TGGGTGATCGCCTTGGTGA  19
Template        521168  ...................  521186

Reverse primer  1       GGCCAGTTGCAGCTTGTTC  19
Template        521473  ...................  521455
```

>CP075771.1 Pseudomonas aeruginosa strain PaLo532 chromosome, complete genome

```
product length = 306
Forward primer  1       TGGGTGATCGCCTTGGTGA  19
Template        467874  ...................  467892

Reverse primer  1       GGCCAGTTGCAGCTTGTTC  19
Template        468179  ...................  468161
```

>CP075769.1 Pseudomonas aeruginosa strain PaLo533 chromosome, complete genome

```
product length = 306
Forward primer  1       TGGGTGATCGCCTTGGTGA  19
Template        748034  ...................  748052

Reverse primer  1       GGCCAGTTGCAGCTTGTTC  19
Template        748339  ...................  748321
```

>CP075768.1 Pseudomonas aeruginosa strain PaLo535 chromosome

```
product length = 306
Forward primer  1       TGGGTGATCGCCTTGGTGA  19
Template        372177  ...................  372195

Reverse primer  1       GGCCAGTTGCAGCTTGTTC  19
Template        372482  ...................  372464
```

>CP075767.1 Pseudomonas aeruginosa strain PaLo536 chromosome, complete genome

```
product length = 306
Forward primer  1       TGGGTGATCGCCTTGGTGA  19
Template        466703  ...................  466721

Reverse primer  1       GGCCAGTTGCAGCTTGTTC  19
Template        467008  ...................  466990
```

>CP075766.1 Pseudomonas aeruginosa strain PaLo538 chromosome, complete genome

```
product length = 306
Forward primer  1       TGGGTGATCGCCTTGGTGA  19
Template        509938  ...................  509956

Reverse primer  1       GGCCAGTTGCAGCTTGTTC  19
Template        510243  ...................  510225
```

>CP075765.1 Pseudomonas aeruginosa strain PaLo539 chromosome, complete genome

```
product length = 306
Forward primer  1       TGGGTGATCGCCTTGGTGA  19
Template        472211  ...................  472229

Reverse primer  1       GGCCAGTTGCAGCTTGTTC  19
Template        472516  ...................  472498
```

>CP075764.1 Pseudomonas aeruginosa strain PaLo541 chromosome, complete genome

```
product length = 306
Forward primer  1       TGGGTGATCGCCTTGGTGA  19
Template        464273  ...................  464291

Reverse primer  1       GGCCAGTTGCAGCTTGTTC  19
Template        464578  ...................  464560
```

>CP075763.1 Pseudomonas aeruginosa strain PaLo543 chromosome, complete genome

```
product length = 306
Forward primer  1       TGGGTGATCGCCTTGGTGA  19
Template        559663  ...................  559681

Reverse primer  1       GGCCAGTTGCAGCTTGTTC  19
Template        559968  ...................  559950
```

>CP075762.1 Pseudomonas aeruginosa strain PaLo544 chromosome, complete genome

```
product length = 306
Forward primer  1       TGGGTGATCGCCTTGGTGA  19
Template        469258  ...................  469276

Reverse primer  1       GGCCAGTTGCAGCTTGTTC  19
Template        469563  ...................  469545
```

>CP075761.1 Pseudomonas aeruginosa strain PaLo545 chromosome

```
product length = 306
Forward primer  1       TGGGTGATCGCCTTGGTGA  19
Template        470482  ...................  470500

Reverse primer  1       GGCCAGTTGCAGCTTGTTC  19
Template        470787  ...................  470769
```

>CP075760.1 Pseudomonas aeruginosa strain PaLo550 chromosome, complete genome

```
product length = 306
Forward primer  1       TGGGTGATCGCCTTGGTGA  19
Template        468147  ...................  468165

Reverse primer  1       GGCCAGTTGCAGCTTGTTC  19
Template        468452  ...................  468434
```

>CP075757.1 Pseudomonas aeruginosa strain PaLo552 chromosome, complete genome

```
product length = 306
Forward primer  1       TGGGTGATCGCCTTGGTGA  19
Template        477684  ...................  477702

Reverse primer  1       GGCCAGTTGCAGCTTGTTC  19
Template        477989  ...................  477971
```

>CP075755.1 Pseudomonas aeruginosa strain PaLo553 chromosome, complete genome

```
product length = 306
Forward primer  1       TGGGTGATCGCCTTGGTGA  19
Template        487491  ...................  487509

Reverse primer  1       GGCCAGTTGCAGCTTGTTC  19
Template        487796  ...................  487778
```

>CP075754.1 Pseudomonas aeruginosa strain PaLo555 chromosome, complete genome

```
product length = 306
Forward primer  1       TGGGTGATCGCCTTGGTGA  19
Template        474034  ...................  474052

Reverse primer  1       GGCCAGTTGCAGCTTGTTC  19
Template        474339  ...................  474321
```

>CP075753.1 Pseudomonas aeruginosa strain PaLo556 chromosome, complete genome

```
product length = 306
Forward primer  1       TGGGTGATCGCCTTGGTGA  19
Template        469363  ...................  469381

Reverse primer  1       GGCCAGTTGCAGCTTGTTC  19
Template        469668  ...................  469650
```

>CP075752.1 Pseudomonas aeruginosa strain PaLo557 chromosome, complete genome

```
product length = 306
Forward primer  1       TGGGTGATCGCCTTGGTGA  19
Template        556567  ...................  556585

Reverse primer  1       GGCCAGTTGCAGCTTGTTC  19
Template        556872  ...................  556854
```

>CP075751.1 Pseudomonas aeruginosa strain PaLo561 chromosome, complete genome

```
product length = 306
Forward primer  1       TGGGTGATCGCCTTGGTGA  19
Template        475197  ...................  475215

Reverse primer  1       GGCCAGTTGCAGCTTGTTC  19
Template        475502  ...................  475484
```

>CP075750.1 Pseudomonas aeruginosa strain PaLo563 chromosome, complete genome

```
product length = 306
Forward primer  1       TGGGTGATCGCCTTGGTGA  19
Template        473645  ...................  473663

Reverse primer  1       GGCCAGTTGCAGCTTGTTC  19
Template        473950  ...................  473932
```

>CP075749.1 Pseudomonas aeruginosa strain PaLo564 chromosome, complete genome

```
product length = 306
Forward primer  1       TGGGTGATCGCCTTGGTGA  19
Template        507955  ...................  507973

Reverse primer  1       GGCCAGTTGCAGCTTGTTC  19
Template        508260  ...................  508242
```

>CP075748.1 Pseudomonas aeruginosa strain PaLo565 chromosome, complete genome

```
product length = 306
Forward primer  1       TGGGTGATCGCCTTGGTGA  19
Template        473646  ...................  473664

Reverse primer  1       GGCCAGTTGCAGCTTGTTC  19
Template        473951  ...................  473933
```

>CP116723.1 Pseudomonas aeruginosa strain 2872 chromosome

```
product length = 306
Forward primer  1        TGGGTGATCGCCTTGGTGA  19
Template        6654875  ...................  6654857

Reverse primer  1        GGCCAGTTGCAGCTTGTTC  19
Template        6654570  ...................  6654588
```

>CP116725.1 Pseudomonas aeruginosa strain 2881 chromosome, complete genome

```
product length = 306
Forward primer  1       TGGGTGATCGCCTTGGTGA  19
Template        530581  ...................  530599

Reverse primer  1       GGCCAGTTGCAGCTTGTTC  19
Template        530886  ...................  530868
```

>CP116722.1 Pseudomonas aeruginosa strain 2868 chromosome, complete genome

```
product length = 306
Forward primer  1       TGGGTGATCGCCTTGGTGA  19
Template        535956  ...................  535974

Reverse primer  1       GGCCAGTTGCAGCTTGTTC  19
Template        536261  ...................  536243
```

>CP116717.1 Pseudomonas aeruginosa strain 2857 chromosome, complete genome

```
product length = 306
Forward primer  1       TGGGTGATCGCCTTGGTGA  19
Template        550194  ...................  550212

Reverse primer  1       GGCCAGTTGCAGCTTGTTC  19
Template        550499  ...................  550481
```

>CP116727.1 Pseudomonas aeruginosa strain 2875 chromosome, complete genome

```
product length = 306
Forward primer  1       TGGGTGATCGCCTTGGTGA  19
Template        533138  ...................  533156

Reverse primer  1       GGCCAGTTGCAGCTTGTTC  19
Template        533443  ...................  533425
```

>CP116718.1 Pseudomonas aeruginosa strain 2858 chromosome, complete genome

```
product length = 306
Forward primer  1       TGGGTGATCGCCTTGGTGA  19
Template        509791  ...................  509809

Reverse primer  1       GGCCAGTTGCAGCTTGTTC  19
Template        510096  ...................  510078
```

>CP116720.1 Pseudomonas aeruginosa strain 2866 chromosome, complete genome

```
product length = 306
Forward primer  1       TGGGTGATCGCCTTGGTGA  19
Template        529966  ...................  529984

Reverse primer  1       GGCCAGTTGCAGCTTGTTC  19
Template        530271  ...................  530253
```

>CP116724.1 Pseudomonas aeruginosa strain 2880 chromosome, complete genome

```
product length = 306
Forward primer  1       TGGGTGATCGCCTTGGTGA  19
Template        545666  ...................  545684

Reverse primer  1       GGCCAGTTGCAGCTTGTTC  19
Template        545971  ...................  545953
```

>CP116721.1 Pseudomonas aeruginosa strain 2867 chromosome, complete genome

```
product length = 306
Forward primer  1       TGGGTGATCGCCTTGGTGA  19
Template        552835  ...................  552853

Reverse primer  1       GGCCAGTTGCAGCTTGTTC  19
Template        553140  ...................  553122
```

>CP116715.1 Pseudomonas aeruginosa strain 2856 chromosome, complete genome

```
product length = 306
Forward primer  1       TGGGTGATCGCCTTGGTGA  19
Template        532946  ...................  532964

Reverse primer  1       GGCCAGTTGCAGCTTGTTC  19
Template        533251  ...................  533233
```

>CP106784.1 Pseudomonas aeruginosa strain NY5085 chromosome, complete genome

```
product length = 306
Forward primer  1       TGGGTGATCGCCTTGGTGA  19
Template        478779  ...................  478797

Reverse primer  1       GGCCAGTTGCAGCTTGTTC  19
Template        479084  ...................  479066
```

>CP096913.1 Pseudomonas aeruginosa strain NY7610 chromosome, complete genome

```
product length = 306
Forward primer  1       TGGGTGATCGCCTTGGTGA  19
Template        490399  ...................  490417

Reverse primer  1       GGCCAGTTGCAGCTTGTTC  19
Template        490704  ...................  490686
```

>CP096912.1 Pseudomonas aeruginosa strain NY7770 chromosome, complete genome

```
product length = 306
Forward primer  1       TGGGTGATCGCCTTGGTGA  19
Template        456256  ...................  456274

Reverse primer  1       GGCCAGTTGCAGCTTGTTC  19
Template        456561  ...................  456543
```

>CP096909.1 Pseudomonas aeruginosa strain NY8688 chromosome, complete genome

```
product length = 306
Forward primer  1       TGGGTGATCGCCTTGGTGA  19
Template        496754  ...................  496772

Reverse primer  1       GGCCAGTTGCAGCTTGTTC  19
Template        497059  ...................  497041
```

>CP096822.1 Pseudomonas aeruginosa strain NY8709 chromosome, complete genome

```
product length = 306
Forward primer  1       TGGGTGATCGCCTTGGTGA  19
Template        478775  ...................  478793

Reverse primer  1       GGCCAGTTGCAGCTTGTTC  19
Template        479080  ...................  479062
```

>CP111030.1 Pseudomonas aeruginosa strain PALA38 chromosome, complete genome

```
product length = 306
Forward primer  1       TGGGTGATCGCCTTGGTGA  19
Template        488678  ...................  488696

Reverse primer  1       GGCCAGTTGCAGCTTGTTC  19
Template        488983  ...................  488965
```

>CP111032.1 Pseudomonas aeruginosa strain PALA54 chromosome, complete genome

```
product length = 306
Forward primer  1       TGGGTGATCGCCTTGGTGA  19
Template        489793  ...................  489811

Reverse primer  1       GGCCAGTTGCAGCTTGTTC  19
Template        490098  ...................  490080
```

>CP111034.1 Pseudomonas aeruginosa strain PALA50 chromosome, complete genome

```
product length = 306
Forward primer  1       TGGGTGATCGCCTTGGTGA  19
Template        502850  ...................  502868

Reverse primer  1       GGCCAGTTGCAGCTTGTTC  19
Template        503155  ...................  503137
```

>CP110353.1 Pseudomonas aeruginosa strain PALA48 chromosome, complete genome

```
product length = 306
Forward primer  1       TGGGTGATCGCCTTGGTGA  19
Template        466534  ...................  466552

Reverse primer  1       GGCCAGTTGCAGCTTGTTC  19
Template        466839  ...................  466821
```

>CP110352.1 Pseudomonas aeruginosa strain PALA47 chromosome, complete genome

```
product length = 306
Forward primer  1       TGGGTGATCGCCTTGGTGA  19
Template        478164  ...................  478182

Reverse primer  1       GGCCAGTTGCAGCTTGTTC  19
Template        478469  ...................  478451
```

>CP110351.1 Pseudomonas aeruginosa strain PALA45 chromosome, complete genome

```
product length = 306
Forward primer  1       TGGGTGATCGCCTTGGTGA  19
Template        466446  ...................  466464

Reverse primer  1       GGCCAGTTGCAGCTTGTTC  19
Template        466751  ...................  466733
```

>CP110350.1 Pseudomonas aeruginosa strain PALA44 chromosome, complete genome

```
product length = 306
Forward primer  1        TGGGTGATCGCCTTGGTGA  19
Template        1584296  ...................  1584314

Reverse primer  1        GGCCAGTTGCAGCTTGTTC  19
Template        1584601  ...................  1584583
```

>CP109932.1 Pseudomonas aeruginosa strain PALA43 chromosome, complete genome

```
product length = 306
Forward primer  1        TGGGTGATCGCCTTGGTGA  19
Template        1548737  ...................  1548755

Reverse primer  1        GGCCAGTTGCAGCTTGTTC  19
Template        1549042  ...................  1549024
```

>CP109931.1 Pseudomonas aeruginosa strain PALA42 chromosome, complete genome

```
product length = 306
Forward primer  1       TGGGTGATCGCCTTGGTGA  19
Template        465829  ...................  465847

Reverse primer  1       GGCCAGTTGCAGCTTGTTC  19
Template        466134  ...................  466116
```

>CP110349.1 Pseudomonas aeruginosa strain PALA40 chromosome, complete genome

```
product length = 306
Forward primer  1       TGGGTGATCGCCTTGGTGA  19
Template        580027  ...................  580045

Reverse primer  1       GGCCAGTTGCAGCTTGTTC  19
Template        580332  ...................  580314
```

>CP109920.1 Pseudomonas aeruginosa strain PALA39 chromosome, complete genome

```
product length = 306
Forward primer  1       TGGGTGATCGCCTTGGTGA  19
Template        465540  ...................  465558

Reverse primer  1       GGCCAGTTGCAGCTTGTTC  19
Template        465845  ...................  465827
```

>CP110348.1 Pseudomonas aeruginosa strain PALA36 chromosome, complete genome

```
product length = 306
Forward primer  1       TGGGTGATCGCCTTGGTGA  19
Template        487921  ...................  487939

Reverse primer  1       GGCCAGTTGCAGCTTGTTC  19
Template        488226  ...................  488208
```

>CP109919.1 Pseudomonas aeruginosa strain PALA56 chromosome, complete genome

```
product length = 306
Forward primer  1       TGGGTGATCGCCTTGGTGA  19
Template        466535  ...................  466553

Reverse primer  1       GGCCAGTTGCAGCTTGTTC  19
Template        466840  ...................  466822
```

>CP109918.1 Pseudomonas aeruginosa strain PALA55 chromosome, complete genome

```
product length = 306
Forward primer  1       TGGGTGATCGCCTTGGTGA  19
Template        470574  ...................  470592

Reverse primer  1       GGCCAGTTGCAGCTTGTTC  19
Template        470879  ...................  470861
```

>CP109856.1 Pseudomonas aeruginosa strain PALA53 chromosome, complete genome

```
product length = 306
Forward primer  1       TGGGTGATCGCCTTGGTGA  19
Template        474027  ...................  474045

Reverse primer  1       GGCCAGTTGCAGCTTGTTC  19
Template        474332  ...................  474314
```

>CP110347.1 Pseudomonas aeruginosa strain PALA52 chromosome, complete genome

```
product length = 306
Forward primer  1       TGGGTGATCGCCTTGGTGA  19
Template        477515  ...................  477533

Reverse primer  1       GGCCAGTTGCAGCTTGTTC  19
Template        477820  ...................  477802
```

>CP109851.1 Pseudomonas aeruginosa strain PALA51 chromosome, complete genome

```
product length = 306
Forward primer  1       TGGGTGATCGCCTTGGTGA  19
Template        503371  ...................  503389

Reverse primer  1       GGCCAGTTGCAGCTTGTTC  19
Template        503676  ...................  503658
```

>CP109850.1 Pseudomonas aeruginosa strain PALA37 chromosome, complete genome

```
product length = 306
Forward primer  1        TGGGTGATCGCCTTGGTGA  19
Template        1915158  ...................  1915140

Reverse primer  1        GGCCAGTTGCAGCTTGTTC  19
Template        1914853  ...................  1914871
```

>CP110346.1 Pseudomonas aeruginosa strain PALA35 chromosome, complete genome

```
product length = 306
Forward primer  1       TGGGTGATCGCCTTGGTGA  19
Template        465893  ...................  465911

Reverse primer  1       GGCCAGTTGCAGCTTGTTC  19
Template        466198  ...................  466180
```

>CP109849.1 Pseudomonas aeruginosa strain PALA34 chromosome, complete genome

```
product length = 306
Forward primer  1       TGGGTGATCGCCTTGGTGA  19
Template        482913  ...................  482931

Reverse primer  1       GGCCAGTTGCAGCTTGTTC  19
Template        483218  ...................  483200
```

>CP109845.1 Pseudomonas aeruginosa strain PALA33 chromosome, complete genome

```
product length = 306
Forward primer  1       TGGGTGATCGCCTTGGTGA  19
Template        568381  ...................  568399

Reverse primer  1       GGCCAGTTGCAGCTTGTTC  19
Template        568686  ...................  568668
```

>CP109844.1 Pseudomonas aeruginosa strain PALA32 chromosome, complete genome

```
product length = 306
Forward primer  1       TGGGTGATCGCCTTGGTGA  19
Template        475129  ...................  475147

Reverse primer  1       GGCCAGTTGCAGCTTGTTC  19
Template        475434  ...................  475416
```

>CP110345.1 Pseudomonas aeruginosa strain PALA30 chromosome, complete genome

```
product length = 306
Forward primer  1       TGGGTGATCGCCTTGGTGA  19
Template        548313  ...................  548331

Reverse primer  1       GGCCAGTTGCAGCTTGTTC  19
Template        548618  ...................  548600
```

>CP109843.1 Pseudomonas aeruginosa strain PALA29 chromosome, complete genome

```
product length = 306
Forward primer  1       TGGGTGATCGCCTTGGTGA  19
Template        471956  ...................  471974

Reverse primer  1       GGCCAGTTGCAGCTTGTTC  19
Template        472261  ...................  472243
```

>CP109835.1 Pseudomonas aeruginosa strain PALA26 chromosome, complete genome

```
product length = 306
Forward primer  1       TGGGTGATCGCCTTGGTGA  19
Template        487496  ...................  487514

Reverse primer  1       GGCCAGTTGCAGCTTGTTC  19
Template        487801  ...................  487783
```

>CP109834.1 Pseudomonas aeruginosa strain PALA25 chromosome, complete genome

```
product length = 306
Forward primer  1       TGGGTGATCGCCTTGGTGA  19
Template        474059  ...................  474077

Reverse primer  1       GGCCAGTTGCAGCTTGTTC  19
Template        474364  ...................  474346
```

>CP110344.1 Pseudomonas aeruginosa strain PALA24 chromosome, complete genome

```
product length = 306
Forward primer  1       TGGGTGATCGCCTTGGTGA  19
Template        474559  ...................  474577

Reverse primer  1       GGCCAGTTGCAGCTTGTTC  19
Template        474864  ...................  474846
```

>CP109833.1 Pseudomonas aeruginosa strain PALA23 chromosome, complete genome

```
product length = 306
Forward primer  1       TGGGTGATCGCCTTGGTGA  19
Template        482243  ...................  482261

Reverse primer  1       GGCCAGTTGCAGCTTGTTC  19
Template        482548  ...................  482530
```

>CP107064.1 Pseudomonas aeruginosa strain PALA20 chromosome, complete genome

```
product length = 306
Forward primer  1        TGGGTGATCGCCTTGGTGA  19
Template        1089128  ...................  1089110

Reverse primer  1        GGCCAGTTGCAGCTTGTTC  19
Template        1088823  ...................  1088841
```

>CP107029.1 Pseudomonas aeruginosa strain PALA19 chromosome, complete genome

```
product length = 306
Forward primer  1       TGGGTGATCGCCTTGGTGA  19
Template        465255  ...................  465273

Reverse primer  1       GGCCAGTTGCAGCTTGTTC  19
Template        465560  ...................  465542
```

>CP106745.1 Pseudomonas aeruginosa strain PALA17 chromosome, complete genome

```
product length = 306
Forward primer  1        TGGGTGATCGCCTTGGTGA  19
Template        6186990  ...................  6186972

Reverse primer  1        GGCCAGTTGCAGCTTGTTC  19
Template        6186685  ...................  6186703
```

>CP106744.1 Pseudomonas aeruginosa strain PALA16 chromosome, complete genome

```
product length = 306
Forward primer  1       TGGGTGATCGCCTTGGTGA  19
Template        482887  ...................  482905

Reverse primer  1       GGCCAGTTGCAGCTTGTTC  19
Template        483192  ...................  483174
```

>CP106743.1 Pseudomonas aeruginosa strain PALA15 chromosome, complete genome

```
product length = 306
Forward primer  1       TGGGTGATCGCCTTGGTGA  19
Template        473925  ...................  473943

Reverse primer  1       GGCCAGTTGCAGCTTGTTC  19
Template        474230  ...................  474212
```

>CP106742.1 Pseudomonas aeruginosa strain PALA14 chromosome, complete genome

```
product length = 306
Forward primer  1       TGGGTGATCGCCTTGGTGA  19
Template        508365  ...................  508383

Reverse primer  1       GGCCAGTTGCAGCTTGTTC  19
Template        508670  ...................  508652
```

>CP106682.1 Pseudomonas aeruginosa strain PALA13 chromosome, complete genome

```
product length = 306
Forward primer  1       TGGGTGATCGCCTTGGTGA  19
Template        518514  ...................  518532

Reverse primer  1       GGCCAGTTGCAGCTTGTTC  19
Template        518819  ...................  518801
```

>CP106681.1 Pseudomonas aeruginosa strain PALA12 chromosome, complete genome

```
product length = 306
Forward primer  1        TGGGTGATCGCCTTGGTGA  19
Template        5066140  ...................  5066122

Reverse primer  1        GGCCAGTTGCAGCTTGTTC  19
Template        5065835  ...................  5065853
```

>CP106680.1 Pseudomonas aeruginosa strain PALA11 chromosome, complete genome

```
product length = 306
Forward primer  1       TGGGTGATCGCCTTGGTGA  19
Template        467097  ...................  467115

Reverse primer  1       GGCCAGTTGCAGCTTGTTC  19
Template        467402  ...................  467384
```

>CP104870.1 Pseudomonas aeruginosa strain PALA9 chromosome, complete genome

```
product length = 306
Forward primer  1       TGGGTGATCGCCTTGGTGA  19
Template        545290  ...................  545308

Reverse primer  1       GGCCAGTTGCAGCTTGTTC  19
Template        545595  ...................  545577
```

>CP104869.1 Pseudomonas aeruginosa strain PALA8 chromosome, complete genome

```
product length = 306
Forward primer  1       TGGGTGATCGCCTTGGTGA  19
Template        478425  ...................  478443

Reverse primer  1       GGCCAGTTGCAGCTTGTTC  19
Template        478730  ...................  478712
```

>CP104868.1 Pseudomonas aeruginosa strain PALA7 chromosome, complete genome

```
product length = 306
Forward primer  1       TGGGTGATCGCCTTGGTGA  19
Template        478164  ...................  478182

Reverse primer  1       GGCCAGTTGCAGCTTGTTC  19
Template        478469  ...................  478451
```

>CP104866.1 Pseudomonas aeruginosa strain PALA4 chromosome, complete genome

```
product length = 306
Forward primer  1        TGGGTGATCGCCTTGGTGA  19
Template        4107974  ...................  4107956

Reverse primer  1        GGCCAGTTGCAGCTTGTTC  19
Template        4107669  ...................  4107687
```

>CP104254.1 Pseudomonas aeruginosa strain PALA1 chromosome, complete genome

```
product length = 306
Forward primer  1       TGGGTGATCGCCTTGGTGA  19
Template        464293  ...................  464311

Reverse primer  1       GGCCAGTTGCAGCTTGTTC  19
Template        464598  ...................  464580
```

>CP114761.1 Pseudomonas aeruginosa strain NF143349 chromosome, complete genome

```
product length = 306
Forward primer  1       TGGGTGATCGCCTTGGTGA  19
Template        538053  ...................  538071

Reverse primer  1       GGCCAGTTGCAGCTTGTTC  19
Template        538358  ...................  538340
```

>CP114374.1 Pseudomonas aeruginosa strain Jade-X chromosome, complete genome

```
product length = 306
Forward primer  1       TGGGTGATCGCCTTGGTGA  19
Template        468766  ...................  468784

Reverse primer  1       GGCCAGTTGCAGCTTGTTC  19
Template        469071  ...................  469053
```

>CP113974.1 Pseudomonas aeruginosa strain M6A146 chromosome, complete genome

```
product length = 306
Forward primer  1        TGGGTGATCGCCTTGGTGA  19
Template        5562545  ...................  5562563

Reverse primer  1        GGCCAGTTGCAGCTTGTTC  19
Template        5562850  ...................  5562832
```

>CP097555.1 Pseudomonas aeruginosa strain B1.2 chromosome, complete genome

```
product length = 306
Forward primer  1       TGGGTGATCGCCTTGGTGA  19
Template        481095  ...................  481113

Reverse primer  1       GGCCAGTTGCAGCTTGTTC  19
Template        481400  ...................  481382
```

>CP097556.1 Pseudomonas aeruginosa strain B2.1 chromosome, complete genome

```
product length = 306
Forward primer  1       TGGGTGATCGCCTTGGTGA  19
Template        481095  ...................  481113

Reverse primer  1       GGCCAGTTGCAGCTTGTTC  19
Template        481400  ...................  481382
```

>CP097557.1 Pseudomonas aeruginosa strain C1.3 chromosome, complete genome

```
product length = 306
Forward primer  1       TGGGTGATCGCCTTGGTGA  19
Template        463052  ...................  463070

Reverse primer  1       GGCCAGTTGCAGCTTGTTC  19
Template        463357  ...................  463339
```

>CP097560.1 Pseudomonas aeruginosa strain C4.2 chromosome, complete genome

```
product length = 306
Forward primer  1       TGGGTGATCGCCTTGGTGA  19
Template        530872  ...................  530890

Reverse primer  1       GGCCAGTTGCAGCTTGTTC  19
Template        531177  ...................  531159
```

>CP113230.1 Pseudomonas aeruginosa strain BIAI 160 chromosome, complete genome

```
product length = 306
Forward primer  1        TGGGTGATCGCCTTGGTGA  19
Template        1219550  ...................  1219532

Reverse primer  1        GGCCAGTTGCAGCTTGTTC  19
Template        1219245  ...................  1219263
```

>CP113246.1 Pseudomonas aeruginosa strain SMC4386 chromosome, complete genome

```
product length = 306
Forward primer  1        TGGGTGATCGCCTTGGTGA  19
Template        5786157  ...................  5786139

Reverse primer  1        GGCCAGTTGCAGCTTGTTC  19
Template        5785852  ...................  5785870
```

>CP113106.1 Pseudomonas aeruginosa strain BIAI 157 chromosome, complete genome

```
product length = 306
Forward primer  1        TGGGTGATCGCCTTGGTGA  19
Template        5273999  ...................  5273981

Reverse primer  1        GGCCAGTTGCAGCTTGTTC  19
Template        5273694  ...................  5273712
```

>CP097857.1 Pseudomonas sp. B111 chromosome, complete genome

```
product length = 306
Forward primer  1        TGGGTGATCGCCTTGGTGA  19
Template        5877684  ...................  5877702

Reverse primer  1        GGCCAGTTGCAGCTTGTTC  19
Template        5877989  ...................  5877971
```

>CP102441.2 Pseudomonas aeruginosa strain PA30 chromosome, complete genome

```
product length = 306
Forward primer  1       TGGGTGATCGCCTTGGTGA  19
Template        504954  ...................  504972

Reverse primer  1       GGCCAGTTGCAGCTTGTTC  19
Template        505259  ...................  505241
```

>CP036492.1 Pseudomonas aeruginosa strain Paer4 chromosome, complete genome

```
product length = 306
Forward primer  1        TGGGTGATCGCCTTGGTGA  19
Template        5804291  ...................  5804273

Reverse primer  1        GGCCAGTTGCAGCTTGTTC  19
Template        5803986  ...................  5804004
```

>CP083357.1 Pseudomonas aeruginosa strain KPA143 chromosome, complete genome

```
product length = 306
Forward primer  1        TGGGTGATCGCCTTGGTGA  19
Template        3471662  ...................  3471680

Reverse primer  1        GGCCAGTTGCAGCTTGTTC  19
Template        3471967  ...................  3471949
```

>CP083359.1 Pseudomonas aeruginosa strain KPA159 chromosome, complete genome

```
product length = 306
Forward primer  1        TGGGTGATCGCCTTGGTGA  19
Template        3294227  ...................  3294245

Reverse primer  1        GGCCAGTTGCAGCTTGTTC  19
Template        3294532  ...................  3294514
```

>CP083358.1 Pseudomonas aeruginosa strain KPA151 chromosome, complete genome

```
product length = 306
Forward primer  1       TGGGTGATCGCCTTGGTGA  19
Template        247463  ...................  247445

Reverse primer  1       GGCCAGTTGCAGCTTGTTC  19
Template        247158  ...................  247176
```

>CP083360.1 Pseudomonas aeruginosa strain KPA83 chromosome, complete genome

```
product length = 307
Forward primer  1        TGGGTGATCGCCTTGGTGA  19
Template        4923599  ...................  4923581

Reverse primer  1        GGCCAGTTGCAGCTTGTTC  19
Template        4923293  ...................  4923311
```

>CP083355.1 Pseudomonas aeruginosa strain KPA134 chromosome, complete genome

```
product length = 306
Forward primer  1        TGGGTGATCGCCTTGGTGA  19
Template        1462618  ...................  1462636

Reverse primer  1        GGCCAGTTGCAGCTTGTTC  19
Template        1462923  ...................  1462905
```

>CP083356.1 Pseudomonas aeruginosa strain KPA140 chromosome, complete genome

```
product length = 306
Forward primer  1       TGGGTGATCGCCTTGGTGA  19
Template        244048  ...................  244030

Reverse primer  1       GGCCAGTTGCAGCTTGTTC  19
Template        243743  ...................  243761
```

>CP083353.1 Pseudomonas aeruginosa strain KPA120 chromosome, complete genome

```
product length = 306
Forward primer  1        TGGGTGATCGCCTTGGTGA  19
Template        3315567  ...................  3315585

Reverse primer  1        GGCCAGTTGCAGCTTGTTC  19
Template        3315872  ...................  3315854
```

>CP083354.1 Pseudomonas aeruginosa strain KPA124 chromosome, complete genome

```
product length = 305
Forward primer  1        TGGGTGATCGCCTTGGTGA  19
Template        1511492  ...................  1511510

Reverse primer  1        GGCCAGTTGCAGCTTGTTC  19
Template        1511796  ...................  1511778
```

>CP083352.1 Pseudomonas aeruginosa strain KPA119 chromosome, complete genome

```
product length = 305
Forward primer  1        TGGGTGATCGCCTTGGTGA  19
Template        5670859  ...................  5670841

Reverse primer  1        GGCCAGTTGCAGCTTGTTC  19
Template        5670555  ...................  5670573
```

>CP107257.1 Pseudomonas aeruginosa strain 2019CK-00034 chromosome, complete genome

```
product length = 306
Forward primer  1       TGGGTGATCGCCTTGGTGA  19
Template        402107  ...................  402089

Reverse primer  1       GGCCAGTTGCAGCTTGTTC  19
Template        401802  ...................  401820
```

>CP104565.1 Pseudomonas aeruginosa strain HS_121 chromosome, complete genome

```
product length = 306
Forward primer  1       TGGGTGATCGCCTTGGTGA  19
Template        461218  ...................  461236

Reverse primer  1       GGCCAGTTGCAGCTTGTTC  19
Template        461523  ...................  461505
```

>CP104567.1 Pseudomonas aeruginosa strain HS_13 chromosome, complete genome

```
product length = 306
Forward primer  1       TGGGTGATCGCCTTGGTGA  19
Template        461218  ...................  461236

Reverse primer  1       GGCCAGTTGCAGCTTGTTC  19
Template        461523  ...................  461505
```

>CP107042.1 Pseudomonas aeruginosa strain GIMC5035:PA21/2013 chromosome

```
product length = 306
Forward primer  1        TGGGTGATCGCCTTGGTGA  19
Template        6068522  ...................  6068540

Reverse primer  1        GGCCAGTTGCAGCTTGTTC  19
Template        6068827  ...................  6068809
```

>CP086213.1 Pseudomonas aeruginosa strain Pa3 chromosome, complete genome

```
product length = 306
Forward primer  1       TGGGTGATCGCCTTGGTGA  19
Template        592231  ...................  592249

Reverse primer  1       GGCCAGTTGCAGCTTGTTC  19
Template        592536  ...................  592518
```

>CP104982.1 Pseudomonas aeruginosa PA14 isolate Gamma chromosome

```
product length = 306
Forward primer  1       TGGGTGATCGCCTTGGTGA  19
Template        487978  ...................  487996

Reverse primer  1       GGCCAGTTGCAGCTTGTTC  19
Template        488283  ...................  488265
```

>CP104984.1 Pseudomonas aeruginosa PA14 isolate Alpha chromosome

```
product length = 306
Forward primer  1       TGGGTGATCGCCTTGGTGA  19
Template        487978  ...................  487996

Reverse primer  1       GGCCAGTTGCAGCTTGTTC  19
Template        488283  ...................  488265
```

>CP104980.1 Pseudomonas aeruginosa PA14 isolate Epsilon chromosome

```
product length = 306
Forward primer  1       TGGGTGATCGCCTTGGTGA  19
Template        487978  ...................  487996

Reverse primer  1       GGCCAGTTGCAGCTTGTTC  19
Template        488283  ...................  488265
```

>CP104985.1 Pseudomonas aeruginosa PA14 chromosome

```
product length = 306
Forward primer  1       TGGGTGATCGCCTTGGTGA  19
Template        487978  ...................  487996

Reverse primer  1       GGCCAGTTGCAGCTTGTTC  19
Template        488283  ...................  488265
```

>CP104981.1 Pseudomonas aeruginosa PA14 isolate Delta chromosome

```
product length = 306
Forward primer  1       TGGGTGATCGCCTTGGTGA  19
Template        487978  ...................  487996

Reverse primer  1       GGCCAGTTGCAGCTTGTTC  19
Template        488283  ...................  488265
```

>CP104983.1 Pseudomonas aeruginosa PA14 isolate Beta chromosome

```
product length = 306
Forward primer  1       TGGGTGATCGCCTTGGTGA  19
Template        487978  ...................  487996

Reverse primer  1       GGCCAGTTGCAGCTTGTTC  19
Template        488283  ...................  488265
```

>CP104913.1 Pseudomonas aeruginosa strain PA-AUTBAM chromosome, complete genome

```
product length = 306
Forward primer  1       TGGGTGATCGCCTTGGTGA  19
Template        473227  ...................  473245

Reverse primer  1       GGCCAGTTGCAGCTTGTTC  19
Template        473532  ...................  473514
```

>CP104695.1 Pseudomonas aeruginosa strain 2021CK-01281 chromosome

```
product length = 306
Forward primer  1        TGGGTGATCGCCTTGGTGA  19
Template        5169341  ...................  5169359

Reverse primer  1        GGCCAGTTGCAGCTTGTTC  19
Template        5169646  ...................  5169628
```

>CP104720.1 Pseudomonas aeruginosa strain NY4593 chromosome, complete genome

```
product length = 306
Forward primer  1       TGGGTGATCGCCTTGGTGA  19
Template        467404  ...................  467422

Reverse primer  1       GGCCAGTTGCAGCTTGTTC  19
Template        467709  ...................  467691
```

>CP104590.1 Pseudomonas aeruginosa strain WTJH36 chromosome, complete genome

```
product length = 306
Forward primer  1        TGGGTGATCGCCTTGGTGA  19
Template        4990589  ...................  4990607

Reverse primer  1        GGCCAGTTGCAGCTTGTTC  19
Template        4990894  ...................  4990876
```

>CP104588.1 Pseudomonas aeruginosa strain WTJH32 chromosome, complete genome

```
product length = 306
Forward primer  1       TGGGTGATCGCCTTGGTGA  19
Template        524240  ...................  524258

Reverse primer  1       GGCCAGTTGCAGCTTGTTC  19
Template        524545  ...................  524527
```

>CP104586.1 Pseudomonas aeruginosa strain WTJH6 chromosome, complete genome

```
product length = 306
Forward primer  1        TGGGTGATCGCCTTGGTGA  19
Template        2693449  ...................  2693431

Reverse primer  1        GGCCAGTTGCAGCTTGTTC  19
Template        2693144  ...................  2693162
```

>CP104584.1 Pseudomonas aeruginosa strain WTJH2 chromosome, complete genome

```
product length = 306
Forward primer  1       TGGGTGATCGCCTTGGTGA  19
Template        524246  ...................  524264

Reverse primer  1       GGCCAGTTGCAGCTTGTTC  19
Template        524551  ...................  524533
```

>CP104301.1 Pseudomonas aeruginosa strain PLL01 chromosome, complete genome

```
product length = 306
Forward primer  1       TGGGTGATCGCCTTGGTGA  19
Template        473227  ...................  473245

Reverse primer  1       GGCCAGTTGCAGCTTGTTC  19
Template        473532  ...................  473514
```

>CP104170.1 Pseudomonas aeruginosa strain HW001G chromosome, complete genome

```
product length = 306
Forward primer  1        TGGGTGATCGCCTTGGTGA  19
Template        6435491  ...................  6435509

Reverse primer  1        GGCCAGTTGCAGCTTGTTC  19
Template        6435796  ...................  6435778
```

>CP096207.1 Pseudomonas aeruginosa TBCF10839 chromosome, complete genome

```
product length = 306
Forward primer  1        TGGGTGATCGCCTTGGTGA  19
Template        5046375  ...................  5046357

Reverse primer  1        GGCCAGTTGCAGCTTGTTC  19
Template        5046070  ...................  5046088
```

>CP079712.1 Pseudomonas aeruginosa strain PAO1-UW chromosome, complete genome

```
product length = 306
Forward primer  1       TGGGTGATCGCCTTGGTGA  19
Template        472605  ...................  472623

Reverse primer  1       GGCCAGTTGCAGCTTGTTC  19
Template        472910  ...................  472892
```

>CP085082.1 Pseudomonas aeruginosa strain PAO1-Holloway chromosome, complete genome

```
product length = 306
Forward primer  1       TGGGTGATCGCCTTGGTGA  19
Template        473006  ...................  473024

Reverse primer  1       GGCCAGTTGCAGCTTGTTC  19
Template        473311  ...................  473293
```

>CP101885.1 Pseudomonas aeruginosa strain M27432 chromosome, complete genome

```
product length = 306
Forward primer  1       TGGGTGATCGCCTTGGTGA  19
Template        473714  ...................  473732

Reverse primer  1       GGCCAGTTGCAGCTTGTTC  19
Template        474019  ...................  474001
```

>CP094677.1 Pseudomonas aeruginosa strain Pa150 chromosome, complete genome

```
product length = 306
Forward primer  1        TGGGTGATCGCCTTGGTGA  19
Template        2593929  ...................  2593911

Reverse primer  1        GGCCAGTTGCAGCTTGTTC  19
Template        2593624  ...................  2593642
```

>CP103307.1 Pseudomonas aeruginosa strain PLL01 chromosome, complete genome

```
product length = 306
Forward primer  1       TGGGTGATCGCCTTGGTGA  19
Template        473227  ...................  473245

Reverse primer  1       GGCCAGTTGCAGCTTGTTC  19
Template        473532  ...................  473514
```

>CP102946.1 Pseudomonas aeruginosa strain SCAID WND1-2022 (148) chromosome, complete genome

```
product length = 306
Forward primer  1       TGGGTGATCGCCTTGGTGA  19
Template        466252  ...................  466270

Reverse primer  1       GGCCAGTTGCAGCTTGTTC  19
Template        466557  ...................  466539
```

>CP102944.1 Pseudomonas aeruginosa strain SCAID TCT1-2022 (325) chromosome, complete genome

```
product length = 306
Forward primer  1       TGGGTGATCGCCTTGGTGA  19
Template        628254  ...................  628272

Reverse primer  1       GGCCAGTTGCAGCTTGTTC  19
Template        628559  ...................  628541
```

>CP102174.1 Pseudomonas aeruginosa strain PA5083 chromosome, complete genome

```
product length = 306
Forward primer  1       TGGGTGATCGCCTTGGTGA  19
Template        509974  ...................  509992
```

```
Reverse primer  1       GGCCAGTTGCAGCTTGTTC  19
Template        510279  ...................  510261
```

>CP101656.1 Pseudomonas aeruginosa strain L1a chromosome, complete genome

```
product length = 306
Forward primer  1        TGGGTGATCGCCTTGGTGA  19
Template        1473541  ...................  1473559

Reverse primer  1        GGCCAGTTGCAGCTTGTTC  19
Template        1473846  ...................  1473828
```

>CP101912.1 Pseudomonas aeruginosa strain ATCC 27853 chromosome, complete genome

```
product length = 306
Forward primer  1       TGGGTGATCGCCTTGGTGA  19
Template        482895  ...................  482913

Reverse primer  1       GGCCAGTTGCAGCTTGTTC  19
Template        483200  ...................  483182
```

>CP101911.1 Pseudomonas aeruginosa strain NWRC-1223 chromosome, complete genome

```
product length = 306
Forward primer  1       TGGGTGATCGCCTTGGTGA  19
Template        466127  ...................  466145

Reverse primer  1       GGCCAGTTGCAGCTTGTTC  19
Template        466432  ...................  466414
```

>CP101540.1 Pseudomonas aeruginosa strain D-2 chromosome, complete genome

```
product length = 306
Forward primer  1       TGGGTGATCGCCTTGGTGA  19
Template        456471  ...................  456489

Reverse primer  1       GGCCAGTTGCAGCTTGTTC  19
Template        456776  ...................  456758
```

>CP094851.1 Pseudomonas aeruginosa strain R20-14 chromosome, complete genome

```
product length = 306
Forward primer  1       TGGGTGATCGCCTTGGTGA  19
Template        472357  ...................  472375

Reverse primer  1       GGCCAGTTGCAGCTTGTTC  19
Template        472662  ...................  472644
```

>CP100760.1 Pseudomonas aeruginosa strain AX0001 chromosome

```
product length = 306
Forward primer  1        TGGGTGATCGCCTTGGTGA  19
Template        1484203  ...................  1484221
```

```
Reverse primer  1        GGCCAGTTGCAGCTTGTTC  19
Template        1484508  ...................  1484490
```

>CP100761.1 Pseudomonas aeruginosa strain PA0011 chromosome

```
product length = 306
Forward primer  1        TGGGTGATCGCCTTGGTGA  19
Template        1484203  ...................  1484221

Reverse primer  1        GGCCAGTTGCAGCTTGTTC  19
Template        1484508  ...................  1484490
```

>CP100759.1 Pseudomonas aeruginosa strain PA0009 chromosome

```
product length = 306
Forward primer  1       TGGGTGATCGCCTTGGTGA  19
Template        319823  ...................  319805

Reverse primer  1       GGCCAGTTGCAGCTTGTTC  19
Template        319518  ...................  319536
```

>CP097710.1 Pseudomonas aeruginosa strain PA-2 chromosome, complete genome

```
product length = 306
Forward primer  1       TGGGTGATCGCCTTGGTGA  19
Template        488992  ...................  489010

Reverse primer  1       GGCCAGTTGCAGCTTGTTC  19
Template        489297  ...................  489279
```

>CP097709.1 Pseudomonas aeruginosa strain PA-1 chromosome, complete genome

```
product length = 306
Forward primer  1       TGGGTGATCGCCTTGGTGA  19
Template        488992  ...................  489010

Reverse primer  1       GGCCAGTTGCAGCTTGTTC  19
Template        489297  ...................  489279
```

>CP100653.1 Pseudomonas aeruginosa strain F13 chromosome, complete genome

```
product length = 306
Forward primer  1       TGGGTGATCGCCTTGGTGA  19
Template        467123  ...................  467141

Reverse primer  1       GGCCAGTTGCAGCTTGTTC  19
Template        467428  ...................  467410
```

>CP099798.1 Pseudomonas aeruginosa strain PAO1-L chromosome, complete genome

```
product length = 306
Forward primer  1       TGGGTGATCGCCTTGGTGA  19
```

```
Template        473225  ...................  473243

Reverse primer  1       GGCCAGTTGCAGCTTGTTC  19
Template        473530  ...................  473512
```

>CP099797.1 Pseudomonas aeruginosa strain PAO1-N chromosome, complete genome

```
product length = 306
Forward primer  1       TGGGTGATCGCCTTGGTGA  19
Template        473231  ...................  473249

Reverse primer  1       GGCCAGTTGCAGCTTGTTC  19
Template        473536  ...................  473518
```

>CP096665.1 Pseudomonas aeruginosa strain PAO1_Mat-X-1 chromosome, complete genome

```
product length = 306
Forward primer  1       TGGGTGATCGCCTTGGTGA  19
Template        473225  ...................  473243

Reverse primer  1       GGCCAGTTGCAGCTTGTTC  19
Template        473530  ...................  473512
```

>CP096664.1 Pseudomonas aeruginosa strain PAO1_Kat-X-2 chromosome, complete genome

```
product length = 306
Forward primer  1       TGGGTGATCGCCTTGGTGA  19
Template        473185  ...................  473203

Reverse primer  1       GGCCAGTTGCAGCTTGTTC  19
Template        473490  ...................  473472
```

>CP091880.1 Pseudomonas aeruginosa strain US449 chromosome, complete genome

```
product length = 306
Forward primer  1       TGGGTGATCGCCTTGGTGA  19
Template        470337  ...................  470355

Reverse primer  1       GGCCAGTTGCAGCTTGTTC  19
Template        470642  ...................  470624
```

>CP069177.1 Pseudomonas aeruginosa strain Z154 chromosome, complete genome

```
product length = 306
Forward primer  1       TGGGTGATCGCCTTGGTGA  19
Template        469955  ...................  469973

Reverse primer  1       GGCCAGTTGCAGCTTGTTC  19
Template        470260  ...................  470242
```

>CP097575.1 Pseudomonas aeruginosa strain UNC_PaerCF25 chromosome, complete genome

```
product length = 306
```

```
Forward primer  1        TGGGTGATCGCCTTGGTGA  19
Template        2606607  ...................  2606589

Reverse primer  1        GGCCAGTTGCAGCTTGTTC  19
Template        2606302  ...................  2606320
```

>CP097383.1 Pseudomonas aeruginosa strain L00-a chromosome, complete genome

```
product length = 306
Forward primer  1       TGGGTGATCGCCTTGGTGA  19
Template        480895  ...................  480913

Reverse primer  1       GGCCAGTTGCAGCTTGTTC  19
Template        481200  ...................  481182
```

>CP097256.1 Pseudomonas aeruginosa strain D5 chromosome, complete genome

```
product length = 306
Forward primer  1       TGGGTGATCGCCTTGGTGA  19
Template        465260  ...................  465278

Reverse primer  1       GGCCAGTTGCAGCTTGTTC  19
Template        465565  ...................  465547
```

>CP096813.1 Pseudomonas aeruginosa strain 8D chromosome, complete genome

```
product length = 306
Forward primer  1        TGGGTGATCGCCTTGGTGA  19
Template        1848837  ...................  1848855

Reverse primer  1        GGCCAGTTGCAGCTTGTTC  19
Template        1849142  ...................  1849124
```

>CP095772.2 Pseudomonas aeruginosa strain 34Pae23 chromosome

```
product length = 306
Forward primer  1       TGGGTGATCGCCTTGGTGA  19
Template        510220  ...................  510238

Reverse primer  1       GGCCAGTTGCAGCTTGTTC  19
Template        510525  ...................  510507
```

>CP095923.1 Pseudomonas aeruginosa strain AR19438 chromosome, complete genome

```
product length = 306
Forward primer  1       TGGGTGATCGCCTTGGTGA  19
Template        511251  ...................  511269

Reverse primer  1       GGCCAGTTGCAGCTTGTTC  19
Template        511556  ...................  511538
```

>CP095920.1 Pseudomonas aeruginosa strain AR19640 chromosome, complete genome

```
product length = 306
Forward primer  1       TGGGTGATCGCCTTGGTGA  19
Template        479290  ...................  479308

Reverse primer  1       GGCCAGTTGCAGCTTGTTC  19
Template        479595  ...................  479577
```

>CP095922.1 Pseudomonas aeruginosa strain AR19583 chromosome, complete genome

```
product length = 306
Forward primer  1       TGGGTGATCGCCTTGGTGA  19
Template        476662  ...................  476680

Reverse primer  1       GGCCAGTTGCAGCTTGTTC  19
Template        476967  ...................  476949
```

>CP095770.1 Pseudomonas aeruginosa strain 34Pae36 chromosome, complete genome

```
product length = 306
Forward primer  1        TGGGTGATCGCCTTGGTGA  19
Template        1661045  ...................  1661027

Reverse primer  1        GGCCAGTTGCAGCTTGTTC  19
Template        1660740  ...................  1660758
```

>CP095774.1 Pseudomonas aeruginosa strain 34Pae8 chromosome, complete genome

```
product length = 306
Forward primer  1       TGGGTGATCGCCTTGGTGA  19
Template        510228  ...................  510246

Reverse primer  1       GGCCAGTTGCAGCTTGTTC  19
Template        510533  ...................  510515
```

>CP090649.1 Pseudomonas aeruginosa strain PA1609 chromosome, complete genome

```
product length = 306
Forward primer  1       TGGGTGATCGCCTTGGTGA  19
Template        484059  ...................  484077

Reverse primer  1       GGCCAGTTGCAGCTTGTTC  19
Template        484364  ...................  484346
```

>CP090648.1 Pseudomonas aeruginosa strain PA1616 chromosome, complete genome

```
product length = 306
Forward primer  1        TGGGTGATCGCCTTGGTGA  19
Template        3010481  ...................  3010499

Reverse primer  1        GGCCAGTTGCAGCTTGTTC  19
Template        3010786  ...................  3010768
```

>CP090647.1 Pseudomonas aeruginosa strain PA1681 chromosome, complete genome

```
product length = 306
Forward primer  1       TGGGTGATCGCCTTGGTGA  19
Template        472687  ...................  472705

Reverse primer  1       GGCCAGTTGCAGCTTGTTC  19
Template        472992  ...................  472974
```

>CP050149.1 Pseudomonas aeruginosa strain CHA chromosome

```
product length = 306
Forward primer  1       TGGGTGATCGCCTTGGTGA  19
Template        466804  ...................  466822

Reverse primer  1       GGCCAGTTGCAGCTTGTTC  19
Template        467109  ...................  467091
```

>CP050148.1 Pseudomonas aeruginosa strain AA43 chromosome, complete genome

```
product length = 306
Forward primer  1       TGGGTGATCGCCTTGGTGA  19
Template        476718  ...................  476736

Reverse primer  1       GGCCAGTTGCAGCTTGTTC  19
Template        477023  ...................  477005
```

>CP050147.1 Pseudomonas aeruginosa strain A5803 chromosome, complete genome

```
product length = 306
Forward primer  1       TGGGTGATCGCCTTGGTGA  19
Template        468794  ...................  468812

Reverse primer  1       GGCCAGTTGCAGCTTGTTC  19
Template        469099  ...................  469081
```

>CP064391.1 Pseudomonas aeruginosa strain PartH-Paeruginosa-RM8376 chromosome, complete genome

```
product length = 306
Forward primer  1        TGGGTGATCGCCTTGGTGA  19
Template        5697854  ...................  5697872

Reverse primer  1        GGCCAGTTGCAGCTTGTTC  19
Template        5698159  ...................  5698141
```

>CP063387.1 Pseudomonas aeruginosa strain ST1076_d100blood2 chromosome, complete genome

```
product length = 306
Forward primer  1       TGGGTGATCGCCTTGGTGA  19
Template        473107  ...................  473125

Reverse primer  1       GGCCAGTTGCAGCTTGTTC  19
Template        473412  ...................  473394
```

>CP047643.1 Pseudomonas aeruginosa CI27 chromosome, complete genome

```
product length = 306
Forward primer  1       TGGGTGATCGCCTTGGTGA  19
Template        487490  ...................  487508

Reverse primer  1       GGCCAGTTGCAGCTTGTTC  19
Template        487795  ...................  487777
```

>CP063396.1 Pseudomonas aeruginosa strain ST167_d26burn chromosome, complete genome

```
product length = 306
Forward primer  1       TGGGTGATCGCCTTGGTGA  19
Template        516965  ...................  516983

Reverse primer  1       GGCCAGTTGCAGCTTGTTC  19
Template        517270  ...................  517252
```

>CP063395.1 Pseudomonas aeruginosa strain ST167_d57blood chromosome, complete genome

```
product length = 306
Forward primer  1       TGGGTGATCGCCTTGGTGA  19
Template        516965  ...................  516983

Reverse primer  1       GGCCAGTTGCAGCTTGTTC  19
Template        517270  ...................  517252
```

>CP063394.1 Pseudomonas aeruginosa strain ST167_d67burn1 chromosome, complete genome

```
product length = 306
Forward primer  1       TGGGTGATCGCCTTGGTGA  19
Template        517436  ...................  517454

Reverse primer  1       GGCCAGTTGCAGCTTGTTC  19
Template        517741  ...................  517723
```

>CP063393.1 Pseudomonas aeruginosa strain ST167_d67burn2 chromosome, complete genome

```
product length = 306
Forward primer  1       TGGGTGATCGCCTTGGTGA  19
Template        516965  ...................  516983

Reverse primer  1       GGCCAGTTGCAGCTTGTTC  19
Template        517270  ...................  517252
```

>CP063392.1 Pseudomonas aeruginosa strain ST167_d68blood1 chromosome, complete genome

```
product length = 306
Forward primer  1       TGGGTGATCGCCTTGGTGA  19
Template        516965  ...................  516983

Reverse primer  1       GGCCAGTTGCAGCTTGTTC  19
Template        517270  ...................  517252
```

>CP063391.1 Pseudomonas aeruginosa strain ST167_d68blood2 chromosome, complete genome

```
product length = 306
Forward primer  1       TGGGTGATCGCCTTGGTGA  19
Template        516965  ...................  516983

Reverse primer  1       GGCCAGTTGCAGCTTGTTC  19
Template        517270  ...................  517252
```

>CP063390.1 Pseudomonas aeruginosa strain ST1076_d97burn1 chromosome, complete genome

```
product length = 306
Forward primer  1       TGGGTGATCGCCTTGGTGA  19
Template        473107  ...................  473125

Reverse primer  1       GGCCAGTTGCAGCTTGTTC  19
Template        473412  ...................  473394
```

>CP063389.1 Pseudomonas aeruginosa strain ST1076_d97burn2 chromosome, complete genome

```
product length = 306
Forward primer  1       TGGGTGATCGCCTTGGTGA  19
Template        473107  ...................  473125

Reverse primer  1       GGCCAGTTGCAGCTTGTTC  19
Template        473412  ...................  473394
```

>CP063388.1 Pseudomonas aeruginosa strain ST1076_d100blood1 chromosome, complete genome

```
product length = 306
Forward primer  1       TGGGTGATCGCCTTGGTGA  19
Template        473107  ...................  473125

Reverse primer  1       GGCCAGTTGCAGCTTGTTC  19
Template        473412  ...................  473394
```

>CP063386.1 Pseudomonas aeruginosa strain ST1076_d118limb1 chromosome, complete genome

```
product length = 306
Forward primer  1       TGGGTGATCGCCTTGGTGA  19
Template        473108  ...................  473126

Reverse primer  1       GGCCAGTTGCAGCTTGTTC  19
Template        473413  ...................  473395
```

>CP063385.1 Pseudomonas aeruginosa strain St1076_d123blood chromosome, complete genome

```
product length = 306
Forward primer  1       TGGGTGATCGCCTTGGTGA  19
Template        473107  ...................  473125

Reverse primer  1       GGCCAGTTGCAGCTTGTTC  19
Template        473412  ...................  473394
```

>CP093967.1 Pseudomonas aeruginosa strain NY4605 chromosome, complete genome

```
product length = 306
Forward primer  1       TGGGTGATCGCCTTGGTGA  19
Template        459737  ...................  459755

Reverse primer  1       GGCCAGTTGCAGCTTGTTC  19
Template        460042  ...................  460024
```

>CP093965.1 Pseudomonas aeruginosa strain ATCC BAA-2108 chromosome, complete genome

```
product length = 306
Forward primer  1       TGGGTGATCGCCTTGGTGA  19
Template        479216  ...................  479234

Reverse primer  1       GGCCAGTTGCAGCTTGTTC  19
Template        479521  ...................  479503
```

>CP093395.1 Pseudomonas aeruginosa strain PA1_NCHU chromosome, complete genome

```
product length = 306
Forward primer  1        TGGGTGATCGCCTTGGTGA  19
Template        4139286  ...................  4139268

Reverse primer  1        GGCCAGTTGCAGCTTGTTC  19
Template        4138981  ...................  4138999
```

>CP093358.1 Pseudomonas aeruginosa strain E167 chromosome, complete genome

```
product length = 306
Forward primer  1       TGGGTGATCGCCTTGGTGA  19
Template        471937  ...................  471955

Reverse primer  1       GGCCAGTTGCAGCTTGTTC  19
Template        472242  ...................  472224
```

>CP093356.1 Pseudomonas aeruginosa strain E125 chromosome, complete genome

```
product length = 306
Forward primer  1       TGGGTGATCGCCTTGGTGA  19
Template        518501  ...................  518519

Reverse primer  1       GGCCAGTTGCAGCTTGTTC  19
Template        518806  ...................  518788
```

>CP093357.1 Pseudomonas aeruginosa strain E131 chromosome, complete genome

```
product length = 306
Forward primer  1       TGGGTGATCGCCTTGGTGA  19
Template        471966  ...................  471984

Reverse primer  1       GGCCAGTTGCAGCTTGTTC  19
Template        472271  ...................  472253
```

>CP093355.1 Pseudomonas aeruginosa strain E104 chromosome, complete genome

```
product length = 306
Forward primer  1       TGGGTGATCGCCTTGGTGA  19
Template        471986  ...................  472004

Reverse primer  1       GGCCAGTTGCAGCTTGTTC  19
Template        472291  ...................  472273
```

>CP093354.1 Pseudomonas aeruginosa strain E113 chromosome, complete genome

```
product length = 306
Forward primer  1       TGGGTGATCGCCTTGGTGA  19
Template        505065  ...................  505083

Reverse primer  1       GGCCAGTTGCAGCTTGTTC  19
Template        505370  ...................  505352
```

>CP093028.1 Pseudomonas aeruginosa strain H05 chromosome, complete genome

```
product length = 306
Forward primer  1       TGGGTGATCGCCTTGGTGA  19
Template        463892  ...................  463910

Reverse primer  1       GGCCAGTTGCAGCTTGTTC  19
Template        464197  ...................  464179
```

>CP093030.1 Pseudomonas aeruginosa strain H04 chromosome, complete genome

```
product length = 306
Forward primer  1       TGGGTGATCGCCTTGGTGA  19
Template        465516  ...................  465534

Reverse primer  1       GGCCAGTTGCAGCTTGTTC  19
Template        465821  ...................  465803
```

>CP093032.1 Pseudomonas aeruginosa strain H02 chromosome, complete genome

```
product length = 306
Forward primer  1       TGGGTGATCGCCTTGGTGA  19
Template        487520  ...................  487538

Reverse primer  1       GGCCAGTTGCAGCTTGTTC  19
Template        487825  ...................  487807
```

>CP093031.1 Pseudomonas aeruginosa strain H03 chromosome, complete genome

```
product length = 306
Forward primer  1       TGGGTGATCGCCTTGGTGA  19
Template        517992  ...................  518010

Reverse primer  1       GGCCAGTTGCAGCTTGTTC  19
Template        518297  ...................  518279
```

>CP093024.1 Pseudomonas aeruginosa strain H06 chromosome

```
product length = 306
Forward primer  1        TGGGTGATCGCCTTGGTGA  19
Template        3020499  ...................  3020481

Reverse primer  1        GGCCAGTTGCAGCTTGTTC  19
Template        3020194  ...................  3020212
```

>CP093013.1 Pseudomonas aeruginosa strain H19 chromosome

```
product length = 306
Forward primer  1        TGGGTGATCGCCTTGGTGA  19
Template        2302500  ...................  2302482

Reverse primer  1        GGCCAGTTGCAGCTTGTTC  19
Template        2302195  ...................  2302213
```

>CP093022.1 Pseudomonas aeruginosa strain H08 chromosome, complete genome

```
product length = 306
Forward primer  1       TGGGTGATCGCCTTGGTGA  19
Template        468796  ...................  468814

Reverse primer  1       GGCCAGTTGCAGCTTGTTC  19
Template        469101  ...................  469083
```

>CP093023.1 Pseudomonas aeruginosa strain H07 chromosome, complete genome

```
product length = 306
Forward primer  1       TGGGTGATCGCCTTGGTGA  19
Template        469463  ...................  469481

Reverse primer  1       GGCCAGTTGCAGCTTGTTC  19
Template        469768  ...................  469750
```

>CP093018.1 Pseudomonas aeruginosa strain H11 chromosome

```
product length = 306
Forward primer  1        TGGGTGATCGCCTTGGTGA  19
Template        3331463  ...................  3331481

Reverse primer  1        GGCCAGTTGCAGCTTGTTC  19
Template        3331768  ...................  3331750
```

>CP093021.1 Pseudomonas aeruginosa strain H09 chromosome, complete genome

```
product length = 306
Forward primer  1       TGGGTGATCGCCTTGGTGA  19
Template        475047  ...................  475065

Reverse primer  1       GGCCAGTTGCAGCTTGTTC  19
Template        475352  ...................  475334
```

>CP093016.1 Pseudomonas aeruginosa strain H15 chromosome, complete genome

```
product length = 306
Forward primer  1        TGGGTGATCGCCTTGGTGA  19
Template        1095073  ...................  1095055

Reverse primer  1        GGCCAGTTGCAGCTTGTTC  19
Template        1094768  ...................  1094786
```

>CP093015.1 Pseudomonas aeruginosa strain H16 chromosome, complete genome

```
product length = 306
Forward primer  1       TGGGTGATCGCCTTGGTGA  19
Template        495288  ...................  495306

Reverse primer  1       GGCCAGTTGCAGCTTGTTC  19
Template        495593  ...................  495575
```

>CP093020.1 Pseudomonas aeruginosa strain H10 chromosome, complete genome

```
product length = 306
Forward primer  1       TGGGTGATCGCCTTGGTGA  19
Template        471956  ...................  471974

Reverse primer  1       GGCCAGTTGCAGCTTGTTC  19
Template        472261  ...................  472243
```

>CP093012.1 Pseudomonas aeruginosa strain H20 chromosome, complete genome

```
product length = 306
Forward primer  1       TGGGTGATCGCCTTGGTGA  19
Template        461651  ...................  461669

Reverse primer  1       GGCCAGTTGCAGCTTGTTC  19
Template        461956  ...................  461938
```

>CP093014.1 Pseudomonas aeruginosa strain H17 chromosome, complete genome

```
product length = 306
Forward primer  1       TGGGTGATCGCCTTGGTGA  19
Template        485572  ...................  485590

Reverse primer  1       GGCCAGTTGCAGCTTGTTC  19
Template        485877  ...................  485859
```

>CP080405.1 Pseudomonas aeruginosa strain PES_P749 chromosome, complete genome

```
product length = 306
Forward primer  1       TGGGTGATCGCCTTGGTGA  19
Template        471729  ...................  471747

Reverse primer  1       GGCCAGTTGCAGCTTGTTC  19
Template        472034  ...................  472016
```

>CP081148.1 Pseudomonas aeruginosa strain NDM1_2 chromosome

```
product length = 306
Forward primer  1       TGGGTGATCGCCTTGGTGA  19
Template        480679  ...................  480697

Reverse primer  1       GGCCAGTTGCAGCTTGTTC  19
Template        480984  ...................  480966
```

>CP092634.1 Pseudomonas aeruginosa strain LS.2c chromosome, complete genome

```
product length = 306
Forward primer  1       TGGGTGATCGCCTTGGTGA  19
Template        464996  ...................  465014

Reverse primer  1       GGCCAGTTGCAGCTTGTTC  19
Template        465301  ...................  465283
```

>CP092629.1 Pseudomonas aeruginosa strain HU20 chromosome, complete genome

```
product length = 306
Forward primer  1        TGGGTGATCGCCTTGGTGA  19
Template        1021097  ...................  1021079

Reverse primer  1        GGCCAGTTGCAGCTTGTTC  19
Template        1020792  ...................  1020810
```

>CP092032.1 Pseudomonas aeruginosa strain ZS-PA-05 chromosome, complete genome

```
product length = 306
Forward primer  1       TGGGTGATCGCCTTGGTGA  19
Template        473256  ...................  473274

Reverse primer  1       GGCCAGTTGCAGCTTGTTC  19
Template        473561  ...................  473543
```

>CP054794.1 Pseudomonas aeruginosa strain A0002 chromosome, complete genome

```
product length = 306
Forward primer  1       TGGGTGATCGCCTTGGTGA  19
Template        458881  ...................  458899

Reverse primer  1       GGCCAGTTGCAGCTTGTTC  19
Template        459186  ...................  459168
```

>CP054793.1 Pseudomonas aeruginosa strain SE5452 chromosome, complete genome

```
product length = 306
Forward primer  1       TGGGTGATCGCCTTGGTGA  19
Template        476958  ...................  476976

Reverse primer  1       GGCCAGTTGCAGCTTGTTC  19
Template        477263  ...................  477245
```

>CP054792.1 Pseudomonas aeruginosa strain SE5431 chromosome, complete genome

```
product length = 306
Forward primer  1       TGGGTGATCGCCTTGGTGA  19
Template        471455  ...................  471473

Reverse primer  1       GGCCAGTTGCAGCTTGTTC  19
Template        471760  ...................  471742
```

>CP054791.1 Pseudomonas aeruginosa strain SE5430 chromosome, complete genome

```
product length = 306
Forward primer  1       TGGGTGATCGCCTTGGTGA  19
Template        508821  ...................  508839

Reverse primer  1       GGCCAGTTGCAGCTTGTTC  19
Template        509126  ...................  509108
```

>CP054790.1 Pseudomonas aeruginosa strain SE5418 chromosome, complete genome

```
product length = 306
Forward primer  1       TGGGTGATCGCCTTGGTGA  19
Template        504635  ...................  504653

Reverse primer  1       GGCCAGTTGCAGCTTGTTC  19
Template        504940  ...................  504922
```

>CP054789.1 Pseudomonas aeruginosa strain SE5381 chromosome, complete genome

```
product length = 306
Forward primer  1       TGGGTGATCGCCTTGGTGA  19
Template        504630  ...................  504648

Reverse primer  1       GGCCAGTTGCAGCTTGTTC  19
Template        504935  ...................  504917
```

>CP054788.1 Pseudomonas aeruginosa strain YTSY4 chromosome, complete genome

```
product length = 306
Forward primer  1        TGGGTGATCGCCTTGGTGA  19
Template        3430914  ...................  3430896

Reverse primer  1        GGCCAGTTGCAGCTTGTTC  19
Template        3430609  ...................  3430627
```

>CP054787.1 Pseudomonas aeruginosa strain HB2011305RE chromosome, complete genome

```
product length = 306
Forward primer  1       TGGGTGATCGCCTTGGTGA  19
Template        520312  ...................  520330

Reverse primer  1       GGCCAGTTGCAGCTTGTTC  19
Template        520617  ...................  520599
```

>CP054786.1 Pseudomonas aeruginosa strain DL201330 chromosome, complete genome

```
product length = 306
Forward primer  1        TGGGTGATCGCCTTGGTGA  19
Template        4952087  ...................  4952105

Reverse primer  1        GGCCAGTTGCAGCTTGTTC  19
Template        4952392  ...................  4952374
```

>CP086122.1 Pseudomonas aeruginosa strain MIN-155 chromosome, complete genome

```
product length = 306
Forward primer  1       TGGGTGATCGCCTTGGTGA  19
Template        472690  ...................  472708

Reverse primer  1       GGCCAGTTGCAGCTTGTTC  19
Template        472995  ...................  472977
```

>CP081477.2 Pseudomonas aeruginosa strain P8W chromosome, complete genome

```
product length = 306
Forward primer  1        TGGGTGATCGCCTTGGTGA  19
Template        6734021  ...................  6734039

Reverse primer  1        GGCCAGTTGCAGCTTGTTC  19
Template        6734326  ...................  6734308
```

>CP090348.1 Pseudomonas aeruginosa strain PA8329 chromosome, complete genome

```
product length = 306
Forward primer  1       TGGGTGATCGCCTTGGTGA  19
Template        466549  ...................  466567

Reverse primer  1       GGCCAGTTGCAGCTTGTTC  19
Template        466854  ...................  466836
```

>CP053747.1 Pseudomonas aeruginosa strain Pae1255-NDM1 chromosome, complete genome

```
product length = 306
Forward primer  1       TGGGTGATCGCCTTGGTGA  19
Template        562682  ...................  562700

Reverse primer  1       GGCCAGTTGCAGCTTGTTC  19
Template        562987  ...................  562969
```

>CP089063.2 Pseudomonas aeruginosa strain UNC_PaerCF37 chromosome, complete genome

```
product length = 306
Forward primer  1        TGGGTGATCGCCTTGGTGA  19
Template        6260260  ...................  6260242

Reverse primer  1        GGCCAGTTGCAGCTTGTTC  19
Template        6259955  ...................  6259973
```

>CP089064.2 Pseudomonas aeruginosa strain UNC_PaerCF35 chromosome, complete genome

```
product length = 306
Forward primer  1        TGGGTGATCGCCTTGGTGA  19
Template        3293731  ...................  3293749

Reverse primer  1        GGCCAGTTGCAGCTTGTTC  19
Template        3294036  ...................  3294018
```

>CP089062.2 Pseudomonas aeruginosa strain UNC_PaerCF38 chromosome, complete genome

```
product length = 306
Forward primer  1        TGGGTGATCGCCTTGGTGA  19
Template        1697503  ...................  1697521

Reverse primer  1        GGCCAGTTGCAGCTTGTTC  19
Template        1697808  ...................  1697790
```

>CP089061.2 Pseudomonas aeruginosa strain UNC_PaerCF41 chromosome, complete genome

```
product length = 306
Forward primer  1        TGGGTGATCGCCTTGGTGA  19
Template        3368822  ...................  3368840

Reverse primer  1        GGCCAGTTGCAGCTTGTTC  19
Template        3369127  ...................  3369109
```

>CP089849.1 Pseudomonas aeruginosa strain PA0523 chromosome, complete genome

```
product length = 306
Forward primer  1       TGGGTGATCGCCTTGGTGA  19
Template        512005  ...................  512023

Reverse primer  1       GGCCAGTTGCAGCTTGTTC  19
Template        512310  ...................  512292
```

>CP089745.1 Pseudomonas aeruginosa strain Pa608 chromosome, complete genome

```
product length = 306
Forward primer  1       TGGGTGATCGCCTTGGTGA  19
Template        462196  ...................  462214

Reverse primer  1       GGCCAGTTGCAGCTTGTTC  19
Template        462501  ...................  462483
```

>CP089238.1 Pseudomonas aeruginosa strain JNQH-PA033 chromosome, complete genome

```
product length = 306
Forward primer  1       TGGGTGATCGCCTTGGTGA  19
Template        487316  ...................  487334

Reverse primer  1       GGCCAGTTGCAGCTTGTTC  19
Template        487621  ...................  487603
```

>CP089236.1 Pseudomonas aeruginosa strain JNQH-PA027 chromosome, complete genome

```
product length = 306
Forward primer  1       TGGGTGATCGCCTTGGTGA  19
Template        525409  ...................  525427

Reverse primer  1       GGCCAGTTGCAGCTTGTTC  19
Template        525714  ...................  525696
```

>CP087675.1 Pseudomonas aeruginosa strain P93127 chromosome, complete genome

```
product length = 306
Forward primer  1       TGGGTGATCGCCTTGGTGA  19
Template        488997  ...................  489015

Reverse primer  1       GGCCAGTTGCAGCTTGTTC  19
Template        489302  ...................  489284
```

>CP087674.1 Pseudomonas aeruginosa strain P4970C chromosome, complete genome

```
product length = 306
Forward primer  1       TGGGTGATCGCCTTGGTGA  19
Template        488991  ...................  489009

Reverse primer  1       GGCCAGTTGCAGCTTGTTC  19
Template        489296  ...................  489278
```

>CP087673.1 Pseudomonas aeruginosa strain P96131 chromosome, complete genome

```
product length = 306
Forward primer  1       TGGGTGATCGCCTTGGTGA  19
Template        475517  ...................  475535

Reverse primer  1       GGCCAGTTGCAGCTTGTTC  19
Template        475822  ...................  475804
```

>CP080369.1 Pseudomonas aeruginosa SG17M chromosome, complete genome

```
product length = 306
Forward primer  1       TGGGTGATCGCCTTGGTGA  19
Template        743691  ...................  743709

Reverse primer  1       GGCCAGTTGCAGCTTGTTC  19
Template        743996  ...................  743978
```

>CP086064.1 Pseudomonas aeruginosa strain CCBH28525 chromosome, complete genome

```
product length = 306
Forward primer  1       TGGGTGATCGCCTTGGTGA  19
Template        471812  ...................  471830

Reverse primer  1       GGCCAGTTGCAGCTTGTTC  19
Template        472117  ...................  472099
```

>CP086016.1 Pseudomonas aeruginosa isolate KB-PA_3 chromosome, complete genome

```
product length = 306
Forward primer  1       TGGGTGATCGCCTTGGTGA  19
Template        500277  ...................  500295

Reverse primer  1       GGCCAGTTGCAGCTTGTTC  19
Template        500582  ...................  500564
```

>CP086010.1 Pseudomonas aeruginosa isolate KB-PA_F19 chromosome, complete genome

```
product length = 306
Forward primer  1       TGGGTGATCGCCTTGGTGA  19
Template        470980  ...................  470998

Reverse primer  1       GGCCAGTTGCAGCTTGTTC  19
Template        471285  ...................  471267
```

>CP084484.1 Pseudomonas sp. PS1(2021) chromosome, complete genome

```
product length = 306
Forward primer  1        TGGGTGATCGCCTTGGTGA  19
Template        6592137  ...................  6592155

Reverse primer  1        GGCCAGTTGCAGCTTGTTC  19
Template        6592442  ...................  6592424
```

>CP083366.1 Pseudomonas aeruginosa strain PS1793 chromosome, complete genome

```
product length = 306
Forward primer  1       TGGGTGATCGCCTTGGTGA  19
Template        465117  ...................  465135

Reverse primer  1       GGCCAGTTGCAGCTTGTTC  19
Template        465422  ...................  465404
```

>CP082821.1 Pseudomonas aeruginosa strain SCAID PLC1-2021 (16/222) chromosome, complete genome

```
product length = 306
Forward primer  1       TGGGTGATCGCCTTGGTGA  19
Template        479793  ...................  479811

Reverse primer  1       GGCCAGTTGCAGCTTGTTC  19
Template        480098  ...................  480080
```

>CP082822.1 Pseudomonas aeruginosa strain SCAID WND1-2021 (9/195) chromosome, complete genome

```
product length = 306
Forward primer  1       TGGGTGATCGCCTTGGTGA  19
Template        497905  ...................  497923
```

```
Reverse primer  1       GGCCAGTTGCAGCTTGTTC  19
Template        498210  ...................  498192
```

>CP082823.1 Pseudomonas aeruginosa strain SCAID TST-2021 (7/157) chromosome, complete genome

```
product length = 306
Forward primer  1       TGGGTGATCGCCTTGGTGA  19
Template        479790  ...................  479808

Reverse primer  1       GGCCAGTTGCAGCTTGTTC  19
Template        480095  ...................  480077
```

>CP078009.1 Pseudomonas aeruginosa strain FAHZU31 chromosome, complete genome

```
product length = 306
Forward primer  1       TGGGTGATCGCCTTGGTGA  19
Template        468148  ...................  468166

Reverse primer  1       GGCCAGTTGCAGCTTGTTC  19
Template        468453  ...................  468435
```

>CP078007.1 Pseudomonas aeruginosa strain FAHZU40 chromosome, complete genome

```
product length = 306
Forward primer  1       TGGGTGATCGCCTTGGTGA  19
Template        480694  ...................  480712

Reverse primer  1       GGCCAGTTGCAGCTTGTTC  19
Template        480999  ...................  480981
```

>CP078006.1 Pseudomonas aeruginosa strain NDTH7329 chromosome, complete genome

```
product length = 306
Forward primer  1       TGGGTGATCGCCTTGGTGA  19
Template        505041  ...................  505059

Reverse primer  1       GGCCAGTTGCAGCTTGTTC  19
Template        505346  ...................  505328
```

>CP078004.1 Pseudomonas aeruginosa strain QZPH16 chromosome, complete genome

```
product length = 306
Forward primer  1       TGGGTGATCGCCTTGGTGA  19
Template        504960  ...................  504978

Reverse primer  1       GGCCAGTTGCAGCTTGTTC  19
Template        505265  ...................  505247
```

>CP078002.1 Pseudomonas aeruginosa strain QZPH21 chromosome, complete genome

```
product length = 306
Forward primer  1       TGGGTGATCGCCTTGGTGA  19
```

```
Template         504960  ...................  504978

Reverse primer   1       GGCCAGTTGCAGCTTGTTC  19
Template         505265  ...................  505247
```

>CP077999.1 Pseudomonas aeruginosa strain SRRSH1120 chromosome, complete genome

```
product length = 306
Forward primer   1       TGGGTGATCGCCTTGGTGA  19
Template         505041  ...................  505059

Reverse primer   1       GGCCAGTTGCAGCTTGTTC  19
Template         505346  ...................  505328
```

>CP077997.1 Pseudomonas aeruginosa strain SRRSH1521 chromosome, complete genome

```
product length = 306
Forward primer   1       TGGGTGATCGCCTTGGTGA  19
Template         468151  ...................  468169

Reverse primer   1       GGCCAGTTGCAGCTTGTTC  19
Template         468456  ...................  468438
```

>CP077994.1 Pseudomonas aeruginosa strain SRRSH2790 chromosome, complete genome

```
product length = 306
Forward primer   1       TGGGTGATCGCCTTGGTGA  19
Template         504966  ...................  504984

Reverse primer   1       GGCCAGTTGCAGCTTGTTC  19
Template         505271  ...................  505253
```

>CP077988.1 Pseudomonas aeruginosa strain ZPPH1 chromosome, complete genome

```
product length = 306
Forward primer   1       TGGGTGATCGCCTTGGTGA  19
Template         515160  ...................  515178

Reverse primer   1       GGCCAGTTGCAGCTTGTTC  19
Template         515465  ...................  515447
```

>CP077985.1 Pseudomonas aeruginosa strain ZPPH2 chromosome, complete genome

```
product length = 306
Forward primer   1       TGGGTGATCGCCTTGGTGA  19
Template         515166  ...................  515184

Reverse primer   1       GGCCAGTTGCAGCTTGTTC  19
Template         515471  ...................  515453
```

>CP077981.1 Pseudomonas aeruginosa strain ZPPH14 chromosome, complete genome

```
product length = 306
```

```
Forward primer  1       TGGGTGATCGCCTTGGTGA  19
Template        473993  ...................  474011

Reverse primer  1       GGCCAGTTGCAGCTTGTTC  19
Template        474298  ...................  474280
```

>CP077977.1 Pseudomonas aeruginosa strain ZPPH29 chromosome, complete genome

```
product length = 306
Forward primer  1       TGGGTGATCGCCTTGGTGA  19
Template        469395  ...................  469413

Reverse primer  1       GGCCAGTTGCAGCTTGTTC  19
Template        469700  ...................  469682
```

>CP077971.1 Pseudomonas aeruginosa strain ZPPH33 chromosome, complete genome

```
product length = 306
Forward primer  1       TGGGTGATCGCCTTGGTGA  19
Template        515160  ...................  515178

Reverse primer  1       GGCCAGTTGCAGCTTGTTC  19
Template        515465  ...................  515447
```

>CP064403.1 Pseudomonas aeruginosa strain WTJH12 chromosome, complete genome

```
product length = 306
Forward primer  1       TGGGTGATCGCCTTGGTGA  19
Template        472795  ...................  472813

Reverse primer  1       GGCCAGTTGCAGCTTGTTC  19
Template        473100  ...................  473082
```

>CP064401.1 Pseudomonas aeruginosa strain NDTH10366 chromosome, complete genome

```
product length = 306
Forward primer  1       TGGGTGATCGCCTTGGTGA  19
Template        508771  ...................  508789

Reverse primer  1       GGCCAGTTGCAGCTTGTTC  19
Template        509076  ...................  509058
```

>CP064399.1 Pseudomonas aeruginosa strain QZPH41 chromosome, complete genome

```
product length = 306
Forward primer  1       TGGGTGATCGCCTTGGTGA  19
Template        457100  ...................  457118

Reverse primer  1       GGCCAGTTGCAGCTTGTTC  19
Template        457405  ...................  457387
```

>CP064397.1 Pseudomonas aeruginosa strain SRRSH1002 chromosome, complete genome

```
product length = 306
Forward primer  1       TGGGTGATCGCCTTGGTGA  19
Template        505782  ...................  505800

Reverse primer  1       GGCCAGTTGCAGCTTGTTC  19
Template        506087  ...................  506069
```

>CP064395.1 Pseudomonas aeruginosa strain SRRSH1408 chromosome, complete genome

```
product length = 306
Forward primer  1       TGGGTGATCGCCTTGGTGA  19
Template        504943  ...................  504961

Reverse primer  1       GGCCAGTTGCAGCTTGTTC  19
Template        505248  ...................  505230
```

>CP064393.1 Pseudomonas aeruginosa strain SRRSH1101 chromosome, complete genome

```
product length = 306
Forward primer  1       TGGGTGATCGCCTTGGTGA  19
Template        504954  ...................  504972

Reverse primer  1       GGCCAGTTGCAGCTTGTTC  19
Template        505259  ...................  505241
```

>CP064392.1 Pseudomonas aeruginosa strain SRRSH15 chromosome, complete genome

```
product length = 306
Forward primer  1       TGGGTGATCGCCTTGGTGA  19
Template        468157  ...................  468175

Reverse primer  1       GGCCAGTTGCAGCTTGTTC  19
Template        468462  ...................  468444
```

>CP080518.1 Pseudomonas aeruginosa strain YY322 chromosome, complete genome

```
product length = 306
Forward primer  1       TGGGTGATCGCCTTGGTGA  19
Template        462082  ...................  462100

Reverse primer  1       GGCCAGTTGCAGCTTGTTC  19
Template        462387  ...................  462369
```

>CP080511.1 Pseudomonas aeruginosa strain DJ06 chromosome, complete genome

```
product length = 306
Forward primer  1       TGGGTGATCGCCTTGGTGA  19
Template        146234  ...................  146216

Reverse primer  1       GGCCAGTTGCAGCTTGTTC  19
Template        145929  ...................  145947
```

>CP071947.1 Pseudomonas aeruginosa strain 2020HL-00861 chromosome, complete genome

```
product length = 306
Forward primer  1        TGGGTGATCGCCTTGGTGA  19
Template        1976059  ...................  1976077

Reverse primer  1        GGCCAGTTGCAGCTTGTTC  19
Template        1976364  ...................  1976346
```

>CP080289.1 Pseudomonas aeruginosa strain PA2207 chromosome, complete genome

```
product length = 306
Forward primer  1       TGGGTGATCGCCTTGGTGA  19
Template        504954  ...................  504972

Reverse primer  1       GGCCAGTTGCAGCTTGTTC  19
Template        505259  ...................  505241
```

>CP080287.1 Pseudomonas aeruginosa strain UNC_PaerCF11 chromosome, complete genome

```
product length = 306
Forward primer  1        TGGGTGATCGCCTTGGTGA  19
Template        5069913  ...................  5069931

Reverse primer  1        GGCCAGTTGCAGCTTGTTC  19
Template        5070218  ...................  5070200
```

>CP080288.1 Pseudomonas aeruginosa strain UNC_PaerCF05 chromosome, complete genome

```
product length = 306
Forward primer  1       TGGGTGATCGCCTTGGTGA  19
Template        237454  ...................  237436

Reverse primer  1       GGCCAGTTGCAGCTTGTTC  19
Template        237149  ...................  237167
```

>CP080282.1 Pseudomonas aeruginosa strain UNC_PaerCF16 chromosome, complete genome

```
product length = 306
Forward primer  1        TGGGTGATCGCCTTGGTGA  19
Template        1967732  ...................  1967750

Reverse primer  1        GGCCAGTTGCAGCTTGTTC  19
Template        1968037  ...................  1968019
```

>CP080286.1 Pseudomonas aeruginosa strain UNC_PaerCF12 chromosome, complete genome

```
product length = 306
Forward primer  1        TGGGTGATCGCCTTGGTGA  19
Template        1730660  ...................  1730678

Reverse primer  1        GGCCAGTTGCAGCTTGTTC  19
Template        1730965  ...................  1730947
```

>CP080285.1 Pseudomonas aeruginosa strain UNC_PaerCF14 chromosome, complete genome

```
product length = 306
Forward primer  1       TGGGTGATCGCCTTGGTGA  19
Template        270922  ...................  270904

Reverse primer  1       GGCCAGTTGCAGCTTGTTC  19
Template        270617  ...................  270635
```

>CP080280.1 Pseudomonas aeruginosa strain UNC_PaerCF20 chromosome, complete genome

```
product length = 306
Forward primer  1        TGGGTGATCGCCTTGGTGA  19
Template        6041734  ...................  6041716

Reverse primer  1        GGCCAGTTGCAGCTTGTTC  19
Template        6041429  ...................  6041447
```

>CP080281.1 Pseudomonas aeruginosa strain UNC_PaerCF17 chromosome, complete genome

```
product length = 306
Forward primer  1        TGGGTGATCGCCTTGGTGA  19
Template        1947018  ...................  1947036

Reverse primer  1        GGCCAGTTGCAGCTTGTTC  19
Template        1947323  ...................  1947305
```

>CP080011.1 Pseudomonas aeruginosa strain TL3773 chromosome, complete genome

```
product length = 306
Forward primer  1        TGGGTGATCGCCTTGGTGA  19
Template        4249594  ...................  4249612

Reverse primer  1        GGCCAGTTGCAGCTTGTTC  19
Template        4249899  ...................  4249881
```

>CP080007.1 Pseudomonas aeruginosa strain S-1 chromosome, complete genome

```
product length = 306
Forward primer  1       TGGGTGATCGCCTTGGTGA  19
Template        482378  ...................  482396

Reverse primer  1       GGCCAGTTGCAGCTTGTTC  19
Template        482683  ...................  482665
```

>CP061376.1 Pseudomonas aeruginosa strain HS17-127 chromosome, complete genome

```
product length = 306
Forward primer  1       TGGGTGATCGCCTTGGTGA  19
Template        495078  ...................  495096

Reverse primer  1       GGCCAGTTGCAGCTTGTTC  19
Template        495383  ...................  495365
```

>CP078564.1 Pseudomonas aeruginosa strain Colony464 chromosome

```
product length = 306
Forward primer  1        TGGGTGATCGCCTTGGTGA  19
Template        4844728  ...................  4844746

Reverse primer  1        GGCCAGTTGCAGCTTGTTC  19
Template        4845033  ...................  4845015
```

>CP053390.1 Pseudomonas aeruginosa strain TL1285 chromosome, complete genome

```
product length = 306
Forward primer  1        TGGGTGATCGCCTTGGTGA  19
Template        3342045  ...................  3342063

Reverse primer  1        GGCCAGTTGCAGCTTGTTC  19
Template        3342350  ...................  3342332
```

>CP075176.1 Pseudomonas aeruginosa strain PA790 chromosome, complete genome

```
product length = 306
Forward primer  1       TGGGTGATCGCCTTGGTGA  19
Template        487224  ...................  487242

Reverse primer  1       GGCCAGTTGCAGCTTGTTC  19
Template        487529  ...................  487511
```

>CP065948.1 Pseudomonas aeruginosa strain PAM68 chromosome, complete genome

```
product length = 306
Forward primer  1        TGGGTGATCGCCTTGGTGA  19
Template        5784392  ...................  5784410

Reverse primer  1        GGCCAGTTGCAGCTTGTTC  19
Template        5784697  ...................  5784679
```

>CP065947.1 Pseudomonas aeruginosa strain PAS6 chromosome, complete genome

```
product length = 306
Forward primer  1        TGGGTGATCGCCTTGGTGA  19
Template        1579613  ...................  1579595

Reverse primer  1        GGCCAGTTGCAGCTTGTTC  19
Template        1579308  ...................  1579326
```

>CP065374.1 Pseudomonas aeruginosa strain PAG7 chromosome, complete genome

```
product length = 306
Forward primer  1        TGGGTGATCGCCTTGGTGA  19
Template        4049448  ...................  4049466

Reverse primer  1        GGCCAGTTGCAGCTTGTTC  19
Template        4049753  ...................  4049735
```

>CP073080.1 Pseudomonas aeruginosa strain NDTH9845 chromosome, complete genome

```
product length = 306
Forward primer  1       TGGGTGATCGCCTTGGTGA  19
Template        506144  ...................  506162

Reverse primer  1       GGCCAGTTGCAGCTTGTTC  19
Template        506449  ...................  506431
```

>CP073082.1 Pseudomonas aeruginosa strain WTJH17 chromosome, complete genome

```
product length = 306
Forward primer  1       TGGGTGATCGCCTTGGTGA  19
Template        466441  ...................  466459

Reverse primer  1       GGCCAGTTGCAGCTTGTTC  19
Template        466746  ...................  466728
```

>CP072783.1 Pseudomonas aeruginosa strain LICME WGH-6 chromosome, complete genome

```
product length = 306
Forward primer  1       TGGGTGATCGCCTTGGTGA  19
Template        470312  ...................  470330

Reverse primer  1       GGCCAGTTGCAGCTTGTTC  19
Template        470617  ...................  470599
```

>CP071731.1 Pseudomonas aeruginosa strain LYSZa2 chromosome, complete genome

```
product length = 306
Forward primer  1        TGGGTGATCGCCTTGGTGA  19
Template        3087987  ...................  3088005

Reverse primer  1        GGCCAGTTGCAGCTTGTTC  19
Template        3088292  ...................  3088274
```

>CP071730.1 Pseudomonas aeruginosa strain LYSZa5 chromosome, complete genome

```
product length = 306
Forward primer  1        TGGGTGATCGCCTTGGTGA  19
Template        3087998  ...................  3088016

Reverse primer  1        GGCCAGTTGCAGCTTGTTC  19
Template        3088303  ...................  3088285
```

>AP024513.1 Pseudomonas aeruginosa Pa12 DNA, complete genome

```
product length = 306
Forward primer  1       TGGGTGATCGCCTTGGTGA  19
Template        472015  ...................  472033

Reverse primer  1       GGCCAGTTGCAGCTTGTTC  19
Template        472320  ...................  472302
```

>CP024024.1 Pseudomonas aeruginosa strain PARM801 chromosome, complete genome

```
product length = 306
Forward primer  1        TGGGTGATCGCCTTGGTGA  19
Template        5388701  ...................  5388719

Reverse primer  1        GGCCAGTTGCAGCTTGTTC  19
Template        5389006  ...................  5388988
```

>CP069198.1 Pseudomonas aeruginosa strain 152962 chromosome, complete genome

```
product length = 306
Forward primer  1        TGGGTGATCGCCTTGGTGA  19
Template        4867598  ...................  4867580

Reverse primer  1        GGCCAGTTGCAGCTTGTTC  19
Template        4867293  ...................  4867311
```

>CP054623.1 Pseudomonas aeruginosa strain DL201330 chromosome, complete genome

```
product length = 306
Forward primer  1       TGGGTGATCGCCTTGGTGA  19
Template        483745  ...................  483763

Reverse primer  1       GGCCAGTTGCAGCTTGTTC  19
Template        484050  ...................  484032
```

>CP065966.1 Pseudomonas aeruginosa strain FDAARGOS_1041 chromosome, complete genome

```
product length = 306
Forward primer  1        TGGGTGATCGCCTTGGTGA  19
Template        4071420  ...................  4071438

Reverse primer  1        GGCCAGTTGCAGCTTGTTC  19
Template        4071725  ...................  4071707
```

>CP027857.1 Pseudomonas aeruginosa strain MPAO1 chromosome, complete genome

```
product length = 306
Forward primer  1       TGGGTGATCGCCTTGGTGA  19
Template        472743  ...................  472761

Reverse primer  1       GGCCAGTTGCAGCTTGTTC  19
Template        473048  ...................  473030
```

>CP065867.1 Pseudomonas aeruginosa strain TJ2014-049 chromosome, complete genome

```
product length = 306
Forward primer  1       TGGGTGATCGCCTTGGTGA  19
Template        469313  ...................  469331

Reverse primer  1       GGCCAGTTGCAGCTTGTTC  19
Template        469618  ...................  469600
```

>CP065865.1 Pseudomonas aeruginosa strain TJ2019-022 chromosome, complete genome

```
product length = 306
Forward primer  1       TGGGTGATCGCCTTGGTGA  19
Template        474777  ...................  474795

Reverse primer  1       GGCCAGTTGCAGCTTGTTC  19
Template        475082  ...................  475064
```

>CP065866.1 Pseudomonas aeruginosa strain TJ2019-017 chromosome, complete genome

```
product length = 306
Forward primer  1       TGGGTGATCGCCTTGGTGA  19
Template        472153  ...................  472171

Reverse primer  1       GGCCAGTTGCAGCTTGTTC  19
Template        472458  ...................  472440
```

>CP065848.1 Pseudomonas aeruginosa strain CMC-097 chromosome, complete genome

```
product length = 306
Forward primer  1       TGGGTGATCGCCTTGGTGA  19
Template        487970  ...................  487988

Reverse primer  1       GGCCAGTTGCAGCTTGTTC  19
Template        488275  ...................  488257
```

>CP065417.1 Pseudomonas aeruginosa isolate P23 chromosome, complete genome

```
product length = 306
Forward primer  1       TGGGTGATCGCCTTGGTGA  19
Template        504954  ...................  504972

Reverse primer  1       GGCCAGTTGCAGCTTGTTC  19
Template        505259  ...................  505241
```

>CP065412.1 Pseudomonas aeruginosa isolate P33 chromosome, complete genome

```
product length = 306
Forward primer  1       TGGGTGATCGCCTTGGTGA  19
Template        504992  ...................  505010

Reverse primer  1       GGCCAGTTGCAGCTTGTTC  19
Template        505297  ...................  505279
```

>CP046402.2 Pseudomonas aeruginosa strain SE5331 chromosome, complete genome

```
product length = 306
Forward primer  1        TGGGTGATCGCCTTGGTGA  19
Template        2635750  ...................  2635732

Reverse primer  1        GGCCAGTTGCAGCTTGTTC  19
Template        2635445  ...................  2635463
```

>CP046406.2 Pseudomonas aeruginosa strain SE5458 chromosome, complete genome

```
product length = 306
Forward primer  1       TGGGTGATCGCCTTGGTGA  19
Template        473558  ...................  473576

Reverse primer  1       GGCCAGTTGCAGCTTGTTC  19
Template        473863  ...................  473845
```

>CP045552.2 Pseudomonas aeruginosa strain YT12746 chromosome, complete genome

```
product length = 306
Forward primer  1       TGGGTGATCGCCTTGGTGA  19
Template        483308  ...................  483326

Reverse primer  1       GGCCAGTTGCAGCTTGTTC  19
Template        483613  ...................  483595
```

>CP060243.1 Pseudomonas aeruginosa strain A-I-1 chromosome, complete genome

```
product length = 306
Forward primer  1       TGGGTGATCGCCTTGGTGA  19
Template        570847  ...................  570865

Reverse primer  1       GGCCAGTTGCAGCTTGTTC  19
Template        571152  ...................  571134
```

>CP060242.1 Pseudomonas aeruginosa strain B-I-1 chromosome, complete genome

```
product length = 306
Forward primer  1       TGGGTGATCGCCTTGGTGA  19
Template        944924  ...................  944942

Reverse primer  1       GGCCAGTTGCAGCTTGTTC  19
Template        945229  ...................  945211
```

>CP060241.1 Pseudomonas aeruginosa strain C-I-1 chromosome, complete genome

```
product length = 306
Forward primer  1        TGGGTGATCGCCTTGGTGA  19
Template        5837923  ...................  5837905

Reverse primer  1        GGCCAGTTGCAGCTTGTTC  19
Template        5837618  ...................  5837636
```

>CP060240.1 Pseudomonas aeruginosa strain G-I-1 chromosome, complete genome

```
product length = 306
Forward primer  1       TGGGTGATCGCCTTGGTGA  19
Template        591622  ...................  591640

Reverse primer  1       GGCCAGTTGCAGCTTGTTC  19
Template        591927  ...................  591909
```

>LR898867.1 Pseudomonas aeruginosa isolate MINF_3A-sc-2280432 genome assembly, chromosome: 1

```
product length = 306
Forward primer  1       TGGGTGATCGCCTTGGTGA  19
Template        478950  ...................  478968

Reverse primer  1       GGCCAGTTGCAGCTTGTTC  19
Template        479255  ...................  479237
```

>LR890619.1 Pseudomonas aeruginosa isolate MINF_7A-sc-2280434 genome assembly, chromosome: 1

```
product length = 306
Forward primer  1       TGGGTGATCGCCTTGGTGA  19
Template        471922  ...................  471940

Reverse primer  1       GGCCAGTTGCAGCTTGTTC  19
Template        472227  ...................  472209
```

>CP061034.1 Pseudomonas aeruginosa strain PA3 chromosome, complete genome

```
product length = 306
Forward primer  1       TGGGTGATCGCCTTGGTGA  19
Template        478185  ...................  478203

Reverse primer  1       GGCCAGTTGCAGCTTGTTC  19
Template        478490  ...................  478472
```

>CP060703.1 Pseudomonas aeruginosa strain NRD619 chromosome, complete genome

```
product length = 306
Forward primer  1       TGGGTGATCGCCTTGGTGA  19
Template        465456  ...................  465474

Reverse primer  1       GGCCAGTTGCAGCTTGTTC  19
Template        465761  ...................  465743
```

>CP060086.1 Pseudomonas aeruginosa strain JNQH-PA57 chromosome, complete genome

```
product length = 306
Forward primer  1       TGGGTGATCGCCTTGGTGA  19
Template        496575  ...................  496593

Reverse primer  1       GGCCAGTTGCAGCTTGTTC  19
Template        496880  ...................  496862
```

>CP059995.1 Pseudomonas aeruginosa strain NY3045 chromosome, complete genome

```
product length = 306
Forward primer  1       TGGGTGATCGCCTTGGTGA  19
Template        499918  ...................  499936
```

```
Reverse primer  1       GGCCAGTTGCAGCTTGTTC  19
Template        500223  ...................  500205
```

>CP059063.1 Pseudomonas aeruginosa strain GIMC5034:PA52Ts32 chromosome

```
product length = 306
Forward primer  1        TGGGTGATCGCCTTGGTGA  19
Template        6319697  ...................  6319679

Reverse primer  1        GGCCAGTTGCAGCTTGTTC  19
Template        6319392  ...................  6319410
```

>CP058331.1 Pseudomonas aeruginosa strain ACR22 chromosome, complete genome

```
product length = 306
Forward primer  1        TGGGTGATCGCCTTGGTGA  19
Template        1032210  ...................  1032228

Reverse primer  1        GGCCAGTTGCAGCTTGTTC  19
Template        1032515  ...................  1032497
```

>CP058333.1 Pseudomonas aeruginosa strain ACR20 chromosome, complete genome

```
product length = 306
Forward primer  1        TGGGTGATCGCCTTGGTGA  19
Template        5177199  ...................  5177181

Reverse primer  1        GGCCAGTTGCAGCTTGTTC  19
Template        5176894  ...................  5176912
```

>CP053119.1 Pseudomonas aeruginosa strain A17CT chromosome

```
product length = 306
Forward primer  1       TGGGTGATCGCCTTGGTGA  19
Template        473339  ...................  473357

Reverse primer  1       GGCCAGTTGCAGCTTGTTC  19
Template        473644  ...................  473626
```

>CP053118.1 Pseudomonas aeruginosa strain A17PBS chromosome

```
product length = 306
Forward primer  1       TGGGTGATCGCCTTGGTGA  19
Template        473231  ...................  473249

Reverse primer  1       GGCCAGTTGCAGCTTGTTC  19
Template        473536  ...................  473518
```

>CP053117.1 Pseudomonas aeruginosa strain P16CT chromosome

```
product length = 306
Forward primer  1       TGGGTGATCGCCTTGGTGA  19
Template        473233  ...................  473251
```

```
Reverse primer  1       GGCCAGTTGCAGCTTGTTC  19
Template        473538  ...................  473520
```

>CP053116.1 Pseudomonas aeruginosa strain P16PBS chromosome

```
product length = 306
Forward primer  1       TGGGTGATCGCCTTGGTGA  19
Template        473231  ...................  473249

Reverse primer  1       GGCCAGTTGCAGCTTGTTC  19
Template        473536  ...................  473518
```

>CP053115.1 Pseudomonas aeruginosa strain P4CT chromosome

```
product length = 306
Forward primer  1       TGGGTGATCGCCTTGGTGA  19
Template        473231  ...................  473249

Reverse primer  1       GGCCAGTTGCAGCTTGTTC  19
Template        473536  ...................  473518
```

>CP053114.1 Pseudomonas aeruginosa strain P4PBS chromosome

```
product length = 306
Forward primer  1       TGGGTGATCGCCTTGGTGA  19
Template        473231  ...................  473249

Reverse primer  1       GGCCAGTTGCAGCTTGTTC  19
Template        473536  ...................  473518
```

>CP053113.1 Pseudomonas aeruginosa strain PAO1CT chromosome

```
product length = 306
Forward primer  1       TGGGTGATCGCCTTGGTGA  19
Template        473285  ...................  473303

Reverse primer  1       GGCCAGTTGCAGCTTGTTC  19
Template        473590  ...................  473572
```

>CP053112.1 Pseudomonas aeruginosa strain PAO1PBS chromosome

```
product length = 306
Forward primer  1       TGGGTGATCGCCTTGGTGA  19
Template        473231  ...................  473249

Reverse primer  1       GGCCAGTTGCAGCTTGTTC  19
Template        473536  ...................  473518
```

>CP053111.1 Pseudomonas aeruginosa strain UAB2CT chromosome

```
product length = 306
Forward primer  1       TGGGTGATCGCCTTGGTGA  19
```

```
Template        473236  ...................  473254

Reverse primer  1       GGCCAGTTGCAGCTTGTTC  19
Template        473541  ...................  473523
```

>CP053110.1 Pseudomonas aeruginosa strain UAB2PBS chromosome

```
product length = 306
Forward primer  1       TGGGTGATCGCCTTGGTGA  19
Template        473231  ...................  473249

Reverse primer  1       GGCCAGTTGCAGCTTGTTC  19
Template        473536  ...................  473518
```

>CP058332.1 Pseudomonas aeruginosa strain B18 chromosome, complete genome

```
product length = 306
Forward primer  1        TGGGTGATCGCCTTGGTGA  19
Template        1241865  ...................  1241847

Reverse primer  1        GGCCAGTTGCAGCTTGTTC  19
Template        1241560  ...................  1241578
```

>CP058323.1 Pseudomonas aeruginosa strain LV chromosome

```
product length = 306
Forward primer  1        TGGGTGATCGCCTTGGTGA  19
Template        1115394  ...................  1115412

Reverse primer  1        GGCCAGTTGCAGCTTGTTC  19
Template        1115699  ...................  1115681
```

>CP046405.1 Pseudomonas aeruginosa strain SE5443 chromosome, complete genome

```
product length = 306
Forward primer  1       TGGGTGATCGCCTTGGTGA  19
Template        481619  ...................  481637

Reverse primer  1       GGCCAGTTGCAGCTTGTTC  19
Template        481924  ...................  481906
```

>CP046404.1 Pseudomonas aeruginosa strain SE5416 chromosome, complete genome

```
product length = 306
Forward primer  1       TGGGTGATCGCCTTGGTGA  19
Template        481769  ...................  481787

Reverse primer  1       GGCCAGTTGCAGCTTGTTC  19
Template        482074  ...................  482056
```

>CP046403.1 Pseudomonas aeruginosa strain SE5369 chromosome, complete genome

```
product length = 306
```

```
Forward primer  1       TGGGTGATCGCCTTGGTGA  19
Template        531174  ...................  531192

Reverse primer  1       GGCCAGTTGCAGCTTGTTC  19
Template        531479  ...................  531461
```

>CP056774.1 Pseudomonas aeruginosa strain CDN129 chromosome, complete genome

```
product length = 306
Forward primer  1        TGGGTGATCGCCTTGGTGA  19
Template        5131265  ...................  5131247

Reverse primer  1        GGCCAGTTGCAGCTTGTTC  19
Template        5130960  ...................  5130978
```

>CP056100.1 Pseudomonas aeruginosa strain PABCH01 chromosome

```
product length = 306
Forward primer  1       TGGGTGATCGCCTTGGTGA  19
Template        484634  ...................  484652

Reverse primer  1       GGCCAGTTGCAGCTTGTTC  19
Template        484939  ...................  484921
```

>CP056090.1 Pseudomonas aeruginosa strain PABCH42 chromosome

```
product length = 306
Forward primer  1       TGGGTGATCGCCTTGGTGA  19
Template        734669  ...................  734651

Reverse primer  1       GGCCAGTTGCAGCTTGTTC  19
Template        734364  ...................  734382
```

>CP056089.1 Pseudomonas aeruginosa strain PABCH46 chromosome

```
product length = 306
Forward primer  1       TGGGTGATCGCCTTGGTGA  19
Template        523014  ...................  523032

Reverse primer  1       GGCCAGTTGCAGCTTGTTC  19
Template        523319  ...................  523301
```

>CP056095.1 Pseudomonas aeruginosa strain PABCH09 chromosome

```
product length = 306
Forward primer  1       TGGGTGATCGCCTTGGTGA  19
Template        526934  ...................  526952

Reverse primer  1       GGCCAGTTGCAGCTTGTTC  19
Template        527239  ...................  527221
```

>CP056092.1 Pseudomonas aeruginosa strain PABCH14 chromosome

```
product length = 306
Forward primer  1       TGGGTGATCGCCTTGGTGA  19
Template        625320  ...................  625338

Reverse primer  1       GGCCAGTTGCAGCTTGTTC  19
Template        625625  ...................  625607
```

>CP056101.1 Pseudomonas aeruginosa strain PABCH45 chromosome

```
product length = 306
Forward primer  1       TGGGTGATCGCCTTGGTGA  19
Template        482760  ...................  482778

Reverse primer  1       GGCCAGTTGCAGCTTGTTC  19
Template        483065  ...................  483047
```

>CP056098.1 Pseudomonas aeruginosa strain PABCH05 chromosome

```
product length = 306
Forward primer  1       TGGGTGATCGCCTTGGTGA  19
Template        462429  ...................  462447

Reverse primer  1       GGCCAGTTGCAGCTTGTTC  19
Template        462734  ...................  462716
```

>CP056094.1 Pseudomonas aeruginosa strain PABCH10 chromosome

```
product length = 306
Forward primer  1       TGGGTGATCGCCTTGGTGA  19
Template        474913  ...................  474931

Reverse primer  1       GGCCAGTTGCAGCTTGTTC  19
Template        475218  ...................  475200
```

>CP056093.1 Pseudomonas aeruginosa strain PABCH13 chromosome

```
product length = 306
Forward primer  1       TGGGTGATCGCCTTGGTGA  19
Template        481293  ...................  481311

Reverse primer  1       GGCCAGTTGCAGCTTGTTC  19
Template        481598  ...................  481580
```

>CP054591.1 Pseudomonas aeruginosa strain CDN118 chromosome, complete genome

```
product length = 306
Forward primer  1        TGGGTGATCGCCTTGGTGA  19
Template        6046582  ...................  6046564

Reverse primer  1        GGCCAGTTGCAGCTTGTTC  19
Template        6046277  ...................  6046295
```

>CP050335.1 Pseudomonas aeruginosa strain DVT401 chromosome, complete genome

```
product length = 306
Forward primer  1       TGGGTGATCGCCTTGGTGA  19
Template        466752  ...................  466770

Reverse primer  1       GGCCAGTTGCAGCTTGTTC  19
Template        467057  ...................  467039
```

>CP050334.1 Pseudomonas aeruginosa strain DVT410 chromosome, complete genome

```
product length = 306
Forward primer  1       TGGGTGATCGCCTTGGTGA  19
Template        468131  ...................  468149

Reverse primer  1       GGCCAGTTGCAGCTTGTTC  19
Template        468436  ...................  468418
```

>CP050333.1 Pseudomonas aeruginosa strain DVT412 chromosome, complete genome

```
product length = 306
Forward primer  1       TGGGTGATCGCCTTGGTGA  19
Template        483006  ...................  483024

Reverse primer  1       GGCCAGTTGCAGCTTGTTC  19
Template        483311  ...................  483293
```

>CP050332.1 Pseudomonas aeruginosa strain DVT413 chromosome, complete genome

```
product length = 306
Forward primer  1       TGGGTGATCGCCTTGGTGA  19
Template        541419  ...................  541437

Reverse primer  1       GGCCAGTTGCAGCTTGTTC  19
Template        541724  ...................  541706
```

>CP050331.1 Pseudomonas aeruginosa strain DVT414 chromosome, complete genome

```
product length = 306
Forward primer  1       TGGGTGATCGCCTTGGTGA  19
Template        467022  ...................  467040

Reverse primer  1       GGCCAGTTGCAGCTTGTTC  19
Template        467327  ...................  467309
```

>CP050330.1 Pseudomonas aeruginosa strain DVT779 chromosome, complete genome

```
product length = 306
Forward primer  1       TGGGTGATCGCCTTGGTGA  19
Template        476935  ...................  476953

Reverse primer  1       GGCCAGTTGCAGCTTGTTC  19
Template        477240  ...................  477222
```

>CP050329.1 Pseudomonas aeruginosa strain DVT417 chromosome, complete genome

```
product length = 306
Forward primer  1       TGGGTGATCGCCTTGGTGA  19
Template        472476  ...................  472494

Reverse primer  1       GGCCAGTTGCAGCTTGTTC  19
Template        472781  ...................  472763
```

>CP050328.1 Pseudomonas aeruginosa strain DVT419 chromosome, complete genome

```
product length = 321
Forward primer  1       TGGGTGATCGCCTTGGTGA  19
Template        462306  ...................  462324

Reverse primer  1       GGCCAGTTGCAGCTTGTTC  19
Template        462626  ...................  462608
```

>CP050327.1 Pseudomonas aeruginosa strain DVT421 chromosome, complete genome

```
product length = 306
Forward primer  1       TGGGTGATCGCCTTGGTGA  19
Template        466312  ...................  466330

Reverse primer  1       GGCCAGTTGCAGCTTGTTC  19
Template        466617  ...................  466599
```

>CP050326.1 Pseudomonas aeruginosa strain DVT423 chromosome, complete genome

```
product length = 306
Forward primer  1       TGGGTGATCGCCTTGGTGA  19
Template        518514  ...................  518532

Reverse primer  1       GGCCAGTTGCAGCTTGTTC  19
Template        518819  ...................  518801
```

>CP050325.1 Pseudomonas aeruginosa strain DVT425 chromosome, complete genome

```
product length = 306
Forward primer  1        TGGGTGATCGCCTTGGTGA  19
Template        3891276  ...................  3891258

Reverse primer  1        GGCCAGTTGCAGCTTGTTC  19
Template        3890971  ...................  3890989
```

>CP050324.1 Pseudomonas aeruginosa strain DVT427 chromosome, complete genome

```
product length = 306
Forward primer  1       TGGGTGATCGCCTTGGTGA  19
Template        465905  ...................  465923

Reverse primer  1       GGCCAGTTGCAGCTTGTTC  19
Template        466210  ...................  466192
```

>CP050323.1 Pseudomonas aeruginosa strain DVT429 chromosome, complete genome

```
product length = 306
Forward primer  1       TGGGTGATCGCCTTGGTGA  19
Template        459740  ...................  459758

Reverse primer  1       GGCCAGTTGCAGCTTGTTC  19
Template        460045  ...................  460027
```

>CP050322.1 Pseudomonas aeruginosa strain DVT729 chromosome, complete genome

```
product length = 306
Forward primer  1        TGGGTGATCGCCTTGGTGA  19
Template        5849899  ...................  5849881

Reverse primer  1        GGCCAGTTGCAGCTTGTTC  19
Template        5849594  ...................  5849612
```

>CP054572.1 Pseudomonas sp. FDAARGOS_761 chromosome, complete genome

```
product length = 306
Forward primer  1        TGGGTGATCGCCTTGGTGA  19
Template        5113646  ...................  5113628

Reverse primer  1        GGCCAGTTGCAGCTTGTTC  19
Template        5113341  ...................  5113359
```

>CP054473.1 Pseudomonas aeruginosa strain PAAK095 chromosome, complete genome

```
product length = 306
Forward primer  1       TGGGTGATCGCCTTGGTGA  19
Template        348764  ...................  348782

Reverse primer  1       GGCCAGTTGCAGCTTGTTC  19
Template        349069  ...................  349051
```

>CP054472.1 Pseudomonas aeruginosa strain PAAK088 chromosome, complete genome

```
product length = 306
Forward primer  1       TGGGTGATCGCCTTGGTGA  19
Template        469871  ...................  469889

Reverse primer  1       GGCCAGTTGCAGCTTGTTC  19
Template        470176  ...................  470158
```

>CP050052.1 Pseudomonas aeruginosa strain LIUYANG-E chromosome, complete genome

```
product length = 306
Forward primer  1       TGGGTGATCGCCTTGGTGA  19
Template        472743  ...................  472761

Reverse primer  1       GGCCAGTTGCAGCTTGTTC  19
Template        473048  ...................  473030
```

>CP050054.1 Pseudomonas aeruginosa strain LIUYANG-A chromosome, complete genome

```
product length = 306
Forward primer  1       TGGGTGATCGCCTTGGTGA  19
Template        472743  ...................  472761

Reverse primer  1       GGCCAGTTGCAGCTTGTTC  19
Template        473048  ...................  473030
```

>CP050053.1 Pseudomonas aeruginosa strain LIUYANG-C chromosome, complete genome

```
product length = 306
Forward primer  1       TGGGTGATCGCCTTGGTGA  19
Template        472743  ...................  472761

Reverse primer  1       GGCCAGTTGCAGCTTGTTC  19
Template        473048  ...................  473030
```

>CP053922.1 Pseudomonas aeruginosa strain YD001 chromosome, complete genome

```
product length = 306
Forward primer  1       TGGGTGATCGCCTTGGTGA  19
Template        483730  ...................  483748

Reverse primer  1       GGCCAGTTGCAGCTTGTTC  19
Template        484035  ...................  484017
```

>CP053917.1 Pseudomonas aeruginosa strain PSE6684 chromosome, complete genome

```
product length = 306
Forward primer  1        TGGGTGATCGCCTTGGTGA  19
Template        2375482  ...................  2375464

Reverse primer  1        GGCCAGTTGCAGCTTGTTC  19
Template        2375177  ...................  2375195
```

>CP053706.1 Pseudomonas aeruginosa strain PAC1 chromosome, complete genome

```
product length = 306
Forward primer  1        TGGGTGATCGCCTTGGTGA  19
Template        5986715  ...................  5986733

Reverse primer  1        GGCCAGTTGCAGCTTGTTC  19
Template        5987020  ...................  5987002
```

>CP053705.1 Pseudomonas aeruginosa strain PAC6 chromosome, complete genome

```
product length = 306
Forward primer  1       TGGGTGATCGCCTTGGTGA  19
Template        481346  ...................  481364

Reverse primer  1       GGCCAGTTGCAGCTTGTTC  19
Template        481651  ...................  481633
```

>CP053687.1 Pseudomonas aeruginosa strain K19PSE24 chromosome

```
product length = 306
Forward primer  1        TGGGTGATCGCCTTGGTGA  19
Template        6417065  ...................  6417083

Reverse primer  1        GGCCAGTTGCAGCTTGTTC  19
Template        6417370  ...................  6417352
```

>CP053686.1 Pseudomonas aeruginosa strain SCAID PHRX1-2019 chromosome

```
product length = 306
Forward primer  1        TGGGTGATCGCCTTGGTGA  19
Template        3753481  ...................  3753499

Reverse primer  1        GGCCAGTTGCAGCTTGTTC  19
Template        3753786  ...................  3753768
```

>CP044533.1 Pseudomonas aeruginosa strain Ps33 chromosome

```
product length = 306
Forward primer  1        TGGGTGATCGCCTTGGTGA  19
Template        5060988  ...................  5061006

Reverse primer  1        GGCCAGTTGCAGCTTGTTC  19
Template        5061293  ...................  5061275
```

>CP051770.1 Pseudomonas aeruginosa strain GIMC5021:PA52Ts17, complete sequence

```
product length = 306
Forward primer  1        TGGGTGATCGCCTTGGTGA  19
Template        6346957  ...................  6346939

Reverse primer  1        GGCCAGTTGCAGCTTGTTC  19
Template        6346652  ...................  6346670
```

>CP051768.1 Pseudomonas aeruginosa strain GIMC5020:PA52Ts2, complete sequence

```
product length = 306
Forward primer  1        TGGGTGATCGCCTTGGTGA  19
Template        6310286  ...................  6310268

Reverse primer  1        GGCCAGTTGCAGCTTGTTC  19
Template        6309981  ...................  6309999
```

>CP051766.1 Pseudomonas aeruginosa strain GIMC5019:PA52Ts1, complete sequence

```
product length = 306
Forward primer  1        TGGGTGATCGCCTTGGTGA  19
Template        6355859  ...................  6355841

Reverse primer  1        GGCCAGTTGCAGCTTGTTC  19
Template        6355554  ...................  6355572
```

>CP053028.1 Pseudomonas aeruginosa PAO1 chromosome, complete genome

```
product length = 306
Forward primer  1       TGGGTGATCGCCTTGGTGA  19
Template        473227  ...................  473245

Reverse primer  1       GGCCAGTTGCAGCTTGTTC  19
Template        473532  ...................  473514
```

>CP052759.1 Pseudomonas aeruginosa strain LYT4 chromosome, complete genome

```
product length = 306
Forward primer  1       TGGGTGATCGCCTTGGTGA  19
Template        476542  ...................  476560

Reverse primer  1       GGCCAGTTGCAGCTTGTTC  19
Template        476847  ...................  476829
```

>CP051547.1 Pseudomonas aeruginosa strain AA2 chromosome, complete genome

```
product length = 306
Forward primer  1       TGGGTGATCGCCTTGGTGA  19
Template        476718  ...................  476736

Reverse primer  1       GGCCAGTTGCAGCTTGTTC  19
Template        477023  ...................  477005
```

>CP046602.1 Pseudomonas aeruginosa strain CMC-115 chromosome, complete genome

```
product length = 306
Forward primer  1       TGGGTGATCGCCTTGGTGA  19
Template        482444  ...................  482462

Reverse primer  1       GGCCAGTTGCAGCTTGTTC  19
Template        482749  ...................  482731
```

>CP045916.1 Pseudomonas aeruginosa strain CF39S chromosome, complete genome

```
product length = 306
Forward primer  1       TGGGTGATCGCCTTGGTGA  19
Template        478688  ...................  478706

Reverse primer  1       GGCCAGTTGCAGCTTGTTC  19
Template        478993  ...................  478975
```

>CP045002.1 Pseudomonas aeruginosa strain PAG5 chromosome, complete genome

```
product length = 306
Forward primer  1       TGGGTGATCGCCTTGGTGA  19
Template        481012  ...................  481030

Reverse primer  1       GGCCAGTTGCAGCTTGTTC  19
Template        481317  ...................  481299
```

>CP021380.2 Pseudomonas aeruginosa strain CCBH4851 genome

```
product length = 306
Forward primer  1       TGGGTGATCGCCTTGGTGA  19
Template        472231  ...................  472249

Reverse primer  1       GGCCAGTTGCAGCTTGTTC  19
Template        472536  ...................  472518
```

>CP049161.1 Pseudomonas aeruginosa strain MS14403 chromosome, complete genome

```
product length = 306
Forward primer  1       TGGGTGATCGCCTTGGTGA  19
Template        472541  ...................  472559

Reverse primer  1       GGCCAGTTGCAGCTTGTTC  19
Template        472846  ...................  472828
```

>CP048791.1 Pseudomonas aeruginosa strain VIT PC9 chromosome, complete genome

```
product length = 306
Forward primer  1       TGGGTGATCGCCTTGGTGA  19
Template        257548  ...................  257530

Reverse primer  1       GGCCAGTTGCAGCTTGTTC  19
Template        257243  ...................  257261
```

>LR739071.1 Pseudomonas aeruginosa strain C7-25 genome assembly, chromosome: C7-25

```
product length = 306
Forward primer  1       TGGGTGATCGCCTTGGTGA  19
Template        469724  ...................  469742

Reverse primer  1       GGCCAGTTGCAGCTTGTTC  19
Template        470029  ...................  470011
```

>LR739069.1 Pseudomonas aeruginosa strain PcyII-40 genome assembly, chromosome: PcyII-40

```
product length = 306
Forward primer  1       TGGGTGATCGCCTTGGTGA  19
Template        549805  ...................  549823

Reverse primer  1       GGCCAGTTGCAGCTTGTTC  19
Template        550110  ...................  550092
```

>LR739068.1 Pseudomonas aeruginosa strain PcyII-29 genome assembly, chromosome: PcyII-29

```
product length = 306
Forward primer  1       TGGGTGATCGCCTTGGTGA  19
Template        478660  ...................  478678

Reverse primer  1       GGCCAGTTGCAGCTTGTTC  19
Template        478965  ...................  478947
```

>CP047697.1 Pseudomonas aeruginosa strain RD1-3 chromosome, complete genome

```
product length = 306
Forward primer  1       TGGGTGATCGCCTTGGTGA  19
Template        467424  ...................  467442

Reverse primer  1       GGCCAGTTGCAGCTTGTTC  19
Template        467729  ...................  467711
```

>CP047592.1 Pseudomonas aeruginosa strain INP-43 chromosome, complete genome

```
product length = 306
Forward primer  1        TGGGTGATCGCCTTGGTGA  19
Template        1660264  ...................  1660282

Reverse primer  1        GGCCAGTTGCAGCTTGTTC  19
Template        1660569  ...................  1660551
```

>CP028132.1 Pseudomonas aeruginosa strain YB01 chromosome, complete genome

```
product length = 306
Forward primer  1       TGGGTGATCGCCTTGGTGA  19
Template        469656  ...................  469674

Reverse primer  1       GGCCAGTTGCAGCTTGTTC  19
Template        469961  ...................  469943
```

>CP025056.3 Pseudomonas aeruginosa strain PB367 chromosome, complete genome

```
product length = 306
Forward primer  1       TGGGTGATCGCCTTGGTGA  19
Template        474185  ...................  474203

Reverse primer  1       GGCCAGTTGCAGCTTGTTC  19
Template        474490  ...................  474472
```

>CP025055.2 Pseudomonas aeruginosa strain PB350 chromosome, complete genome

```
product length = 306
Forward primer  1       TGGGTGATCGCCTTGGTGA  19
Template        474185  ...................  474203

Reverse primer  1       GGCCAGTTGCAGCTTGTTC  19
Template        474490  ...................  474472
```

>CP047069.1 Pseudomonas aeruginosa strain Environ_1 chromosome

```
product length = 306
Forward primer  1       TGGGTGATCGCCTTGGTGA  19
Template        469620  ...................  469638

Reverse primer  1       GGCCAGTTGCAGCTTGTTC  19
Template        469925  ...................  469907
```

>CP047063.1 Pseudomonas aeruginosa strain delta6_4 chromosome

```
product length = 306
Forward primer  1       TGGGTGATCGCCTTGGTGA  19
Template        473229  ...................  473247

Reverse primer  1       GGCCAGTTGCAGCTTGTTC  19
Template        473534  ...................  473516
```

>CP047070.1 Pseudomonas aeruginosa strain Environ_2 chromosome

```
product length = 306
Forward primer  1       TGGGTGATCGCCTTGGTGA  19
Template        473225  ...................  473243

Reverse primer  1       GGCCAGTTGCAGCTTGTTC  19
Template        473530  ...................  473512
```

>CP047064.1 Pseudomonas aeruginosa strain delta6_5 chromosome

```
product length = 306
Forward primer  1       TGGGTGATCGCCTTGGTGA  19
Template        473229  ...................  473247

Reverse primer  1       GGCCAGTTGCAGCTTGTTC  19
Template        473534  ...................  473516
```

>CP047061.1 Pseudomonas aeruginosa strain delta6_2 chromosome

```
product length = 306
Forward primer  1       TGGGTGATCGCCTTGGTGA  19
Template        473227  ...................  473245

Reverse primer  1       GGCCAGTTGCAGCTTGTTC  19
Template        473532  ...................  473514
```

>CP047062.1 Pseudomonas aeruginosa strain delta6_3 chromosome

```
product length = 306
Forward primer  1       TGGGTGATCGCCTTGGTGA  19
Template        473228  ...................  473246

Reverse primer  1       GGCCAGTTGCAGCTTGTTC  19
Template        473533  ...................  473515
```

>CP047067.1 Pseudomonas aeruginosa strain Cas9_1 chromosome

```
product length = 306
Forward primer  1       TGGGTGATCGCCTTGGTGA  19
Template        473225  ...................  473243

Reverse primer  1       GGCCAGTTGCAGCTTGTTC  19
Template        473530  ...................  473512
```

>CP047066.1 Pseudomonas aeruginosa strain delta10 chromosome

```
product length = 306
Forward primer  1       TGGGTGATCGCCTTGGTGA  19
Template        473225  ...................  473243

Reverse primer  1       GGCCAGTTGCAGCTTGTTC  19
Template        473530  ...................  473512
```

>CP047068.1 Pseudomonas aeruginosa strain Cas9_2 chromosome

```
product length = 306
Forward primer  1       TGGGTGATCGCCTTGGTGA  19
Template        473225  ...................  473243

Reverse primer  1       GGCCAGTTGCAGCTTGTTC  19
Template        473530  ...................  473512
```

>CP047065.1 Pseudomonas aeruginosa strain delta6_6 chromosome

```
product length = 306
Forward primer  1       TGGGTGATCGCCTTGGTGA  19
Template        473226  ...................  473244

Reverse primer  1       GGCCAGTTGCAGCTTGTTC  19
Template        473531  ...................  473513
```

>CP039990.1 Pseudomonas aeruginosa strain T2101 chromosome, complete genome

```
product length = 306
Forward primer  1       TGGGTGATCGCCTTGGTGA  19
Template        488522  ...................  488540

Reverse primer  1       GGCCAGTTGCAGCTTGTTC  19
Template        488827  ...................  488809
```

>CP039988.1 Pseudomonas aeruginosa strain T2436 chromosome, complete genome

```
product length = 306
Forward primer  1       TGGGTGATCGCCTTGGTGA  19
Template        485636  ...................  485654

Reverse primer  1       GGCCAGTTGCAGCTTGTTC  19
Template        485941  ...................  485923
```

>CP046069.1 Pseudomonas aeruginosa strain KRP1 chromosome, complete genome

```
product length = 306
Forward primer  1       TGGGTGATCGCCTTGGTGA  19
Template        471973  ...................  471991

Reverse primer  1       GGCCAGTTGCAGCTTGTTC  19
Template        472278  ...................  472260
```

>CP046060.1 Pseudomonas aeruginosa strain 1811-18R001 chromosome, complete genome

```
product length = 306
Forward primer  1       TGGGTGATCGCCTTGGTGA  19
Template        465517  ...................  465535

Reverse primer  1       GGCCAGTTGCAGCTTGTTC  19
Template        465822  ...................  465804
```

>CP046061.1 Pseudomonas aeruginosa strain 1811-13R031 chromosome, complete genome

```
product length = 306
Forward primer  1       TGGGTGATCGCCTTGGTGA  19
Template        465517  ...................  465535

Reverse primer  1       GGCCAGTTGCAGCTTGTTC  19
Template        465822  ...................  465804
```

>CP041945.1 Pseudomonas aeruginosa strain ST773 chromosome, complete genome

```
product length = 306
Forward primer  1       TGGGTGATCGCCTTGGTGA  19
Template        486582  ...................  486600

Reverse primer  1       GGCCAGTTGCAGCTTGTTC  19
Template        486887  ...................  486869
```

>CP045739.1 Pseudomonas aeruginosa strain AG1 chromosome, complete genome

```
product length = 306
Forward primer  1       TGGGTGATCGCCTTGGTGA  19
Template        557295  ...................  557313

Reverse primer  1       GGCCAGTTGCAGCTTGTTC  19
Template        557600  ...................  557582
```

>CP045768.1 Pseudomonas aeruginosa strain CFSAN084950 chromosome, complete genome

```
product length = 306
Forward primer  1        TGGGTGATCGCCTTGGTGA  19
Template        5893489  ...................  5893507

Reverse primer  1        GGCCAGTTGCAGCTTGTTC  19
Template        5893794  ...................  5893776
```

>CP042967.1 Pseudomonas aeruginosa PA99 chromosome, complete genome

```
product length = 306
Forward primer  1        TGGGTGATCGCCTTGGTGA  19
Template        3563852  ...................  3563870

Reverse primer  1        GGCCAGTTGCAGCTTGTTC  19
Template        3564157  ...................  3564139
```

>CP024630.1 Pseudomonas aeruginosa strain PA59 chromosome, complete genome

```
product length = 306
Forward primer  1       TGGGTGATCGCCTTGGTGA  19
Template        466495  ...................  466513

Reverse primer  1       GGCCAGTTGCAGCTTGTTC  19
Template        466800  ...................  466782
```

>CP044006.1 Pseudomonas aeruginosa strain E90 chromosome, complete genome

```
product length = 306
Forward primer  1       TGGGTGATCGCCTTGGTGA  19
Template        482230  ...................  482248

Reverse primer  1       GGCCAGTTGCAGCTTGTTC  19
Template        482535  ...................  482517
```

>CP043549.1 Pseudomonas aeruginosa strain GIMC5002:PAT-169 chromosome

```
product length = 306
Forward primer  1        TGGGTGATCGCCTTGGTGA  19
Template        5833795  ...................  5833777

Reverse primer  1        GGCCAGTTGCAGCTTGTTC  19
Template        5833490  ...................  5833508
```

>CP043483.1 Pseudomonas aeruginosa strain GIMC5001:PAT-23 chromosome

```
product length = 306
Forward primer  1       TGGGTGATCGCCTTGGTGA  19
Template        508743  ...................  508761

Reverse primer  1       GGCCAGTTGCAGCTTGTTC  19
Template        509048  ...................  509030
```

>LR700248.1 Pseudomonas aeruginosa isolate ID40 genome assembly, chromosome: ID40_omosome

```
product length = 306
Forward primer  1        TGGGTGATCGCCTTGGTGA  19
Template        4385631  ...................  4385649

Reverse primer  1        GGCCAGTTGCAGCTTGTTC  19
Template        4385936  ...................  4385918
```

>CP042269.1 Pseudomonas aeruginosa strain HOU1 chromosome, complete genome

```
product length = 306
Forward primer  1       TGGGTGATCGCCTTGGTGA  19
Template        481132  ...................  481150

Reverse primer  1       GGCCAGTTGCAGCTTGTTC  19
Template        481437  ...................  481419
```

>CP043328.1 Pseudomonas aeruginosa strain CCUG 51971 chromosome, complete genome

```
product length = 306
Forward primer  1       TGGGTGATCGCCTTGGTGA  19
Template        473647  ...................  473665

Reverse primer  1       GGCCAGTTGCAGCTTGTTC  19
Template        473952  ...................  473934
```

>CP028959.1 Pseudomonas aeruginosa strain IMP66 chromosome, complete genome

```
product length = 306
Forward primer  1       TGGGTGATCGCCTTGGTGA  19
Template        477329  ...................  477347

Reverse primer  1       GGCCAGTTGCAGCTTGTTC  19
Template        477634  ...................  477616
```

>CP028848.1 Pseudomonas aeruginosa strain IMP67 chromosome, complete genome

```
product length = 306
Forward primer  1       TGGGTGATCGCCTTGGTGA  19
Template        477329  ...................  477347

Reverse primer  1       GGCCAGTTGCAGCTTGTTC  19
Template        477634  ...................  477616
```

>CP028849.1 Pseudomonas aeruginosa strain IMP68 chromosome, complete genome

```
product length = 306
Forward primer  1       TGGGTGATCGCCTTGGTGA  19
Template        477327  ...................  477345

Reverse primer  1       GGCCAGTTGCAGCTTGTTC  19
Template        477632  ...................  477614
```

>CP040684.1 Pseudomonas aeruginosa strain C79 chromosome, complete genome

```
product length = 306
Forward primer  1        TGGGTGATCGCCTTGGTGA  19
Template        4246984  ...................  4246966

Reverse primer  1        GGCCAGTTGCAGCTTGTTC  19
Template        4246679  ...................  4246697
```

>CP041785.1 Pseudomonas aeruginosa strain SCAID WND3-2019 chromosome

```
product length = 306
Forward primer  1        TGGGTGATCGCCTTGGTGA  19
Template        5904971  ...................  5904989

Reverse primer  1        GGCCAGTTGCAGCTTGTTC  19
Template        5905276  ...................  5905258
```

>CP041787.1 Pseudomonas aeruginosa strain SCAID WND1-2019 chromosome

```
product length = 306
Forward primer  1        TGGGTGATCGCCTTGGTGA  19
Template        4755025  ...................  4755043

Reverse primer  1        GGCCAGTTGCAGCTTGTTC  19
Template        4755330  ...................  4755312
```

>CP041786.1 Pseudomonas aeruginosa strain SCAID WND2-2019 chromosome

```
product length = 306
Forward primer  1       TGGGTGATCGCCTTGGTGA  19
Template        470283  ...................  470301

Reverse primer  1       GGCCAGTTGCAGCTTGTTC  19
Template        470588  ...................  470570
```

>CP041773.1 Pseudomonas aeruginosa strain 519119 chromosome, complete genome

```
product length = 306
Forward primer  1        TGGGTGATCGCCTTGGTGA  19
Template        2973654  ...................  2973672

Reverse primer  1        GGCCAGTTGCAGCTTGTTC  19
Template        2973959  ...................  2973941
```

>CP041772.1 Pseudomonas aeruginosa strain 243931 chromosome, complete genome

```
product length = 306
Forward primer  1        TGGGTGATCGCCTTGGTGA  19
Template        2884501  ...................  2884519

Reverse primer  1        GGCCAGTTGCAGCTTGTTC  19
Template        2884806  ...................  2884788
```

>CP041771.1 Pseudomonas aeruginosa strain A681 chromosome, complete genome

```
product length = 306
Forward primer  1       TGGGTGATCGCCTTGGTGA  19
Template        470110  ...................  470128

Reverse primer  1       GGCCAGTTGCAGCTTGTTC  19
Template        470415  ...................  470397
```

>CP041774.1 Pseudomonas aeruginosa strain 60503 chromosome, complete genome

```
product length = 306
Forward primer  1       TGGGTGATCGCCTTGGTGA  19
Template        487364  ...................  487382

Reverse primer  1       GGCCAGTTGCAGCTTGTTC  19
Template        487669  ...................  487651
```

>LR657304.1 Pseudomonas aeruginosa strain PAK genome assembly, chromosome: 1

```
product length = 306
Forward primer  1       TGGGTGATCGCCTTGGTGA  19
Template        461734  ...................  461752

Reverse primer  1       GGCCAGTTGCAGCTTGTTC  19
Template        462039  ...................  462021
```

>CP034244.1 Pseudomonas aeruginosa UCBPP-PA14 chromosome

```
product length = 306
Forward primer  1       TGGGTGATCGCCTTGGTGA  19
Template        487916  ...................  487934

Reverse primer  1       GGCCAGTTGCAGCTTGTTC  19
Template        488221  ...................  488203
```

>CP041013.1 Pseudomonas aeruginosa strain FDAARGOS_610 chromosome, complete genome

```
product length = 306
Forward primer  1        TGGGTGATCGCCTTGGTGA  19
Template        6372477  ...................  6372459

Reverse primer  1        GGCCAGTTGCAGCTTGTTC  19
Template        6372172  ...................  6372190
```

>CP041008.1 Pseudomonas aeruginosa strain FDAARGOS_767 chromosome, complete genome

```
product length = 306
Forward primer  1        TGGGTGATCGCCTTGGTGA  19
Template        5250669  ...................  5250651

Reverse primer  1        GGCCAGTTGCAGCTTGTTC  19
Template        5250364  ...................  5250382
```

>CP032569.2 Pseudomonas aeruginosa strain BA7823 chromosome, complete genome

```
product length = 306
Forward primer  1       TGGGTGATCGCCTTGGTGA  19
Template        351155  ...................  351173

Reverse primer  1       GGCCAGTTGCAGCTTGTTC  19
Template        351460  ...................  351442
```

>CP040127.1 Pseudomonas aeruginosa strain PA298 chromosome, complete genome

```
product length = 306
Forward primer  1       TGGGTGATCGCCTTGGTGA  19
Template        472792  ...................  472810

Reverse primer  1       GGCCAGTTGCAGCTTGTTC  19
Template        473097  ...................  473079
```

>LR590474.1 Pseudomonas aeruginosa strain NCTC13618 genome assembly, chromosome: 1

```
product length = 306
Forward primer  1       TGGGTGATCGCCTTGGTGA  19
Template        472031  ...................  472049

Reverse primer  1       GGCCAGTTGCAGCTTGTTC  19
Template        472336  ...................  472318
```

>LR590473.1 Pseudomonas aeruginosa strain NCTC13359 genome assembly, chromosome: 1

```
product length = 306
Forward primer  1       TGGGTGATCGCCTTGGTGA  19
Template        856128  ...................  856146

Reverse primer  1       GGCCAGTTGCAGCTTGTTC  19
Template        856433  ...................  856415
```

>CP039749.1 Pseudomonas aeruginosa strain PRD-10 chromosome

```
product length = 306
Forward primer  1        TGGGTGATCGCCTTGGTGA  19
Template        6537445  ...................  6537463

Reverse primer  1        GGCCAGTTGCAGCTTGTTC  19
Template        6537750  ...................  6537732
```

>CP039293.1 Pseudomonas aeruginosa strain PABL048 chromosome, complete genome

```
product length = 306
Forward primer  1       TGGGTGATCGCCTTGGTGA  19
Template        464979  ...................  464997

Reverse primer  1       GGCCAGTTGCAGCTTGTTC  19
Template        465284  ...................  465266
```

>CP038661.1 Pseudomonas aeruginosa strain AJ D 2 chromosome

```
product length = 306
Forward primer  1       TGGGTGATCGCCTTGGTGA  19
Template        476006  ...................  476024

Reverse primer  1       GGCCAGTTGCAGCTTGTTC  19
Template        476311  ...................  476293
```

>CP037925.1 Pseudomonas aeruginosa strain AES1M chromosome, complete genome

```
product length = 306
Forward primer  1       TGGGTGATCGCCTTGGTGA  19
Template        467052  ...................  467070

Reverse primer  1       GGCCAGTTGCAGCTTGTTC  19
Template        467357  ...................  467339
```

>CP037926.1 Pseudomonas aeruginosa strain AES1R chromosome, complete genome

```
product length = 306
Forward primer  1       TGGGTGATCGCCTTGGTGA  19
Template        466988  ...................  467006

Reverse primer  1       GGCCAGTTGCAGCTTGTTC  19
Template        467293  ...................  467275
```

>CP028332.1 Pseudomonas aeruginosa strain PA-VAP-1 chromosome

```
product length = 306
Forward primer  1       TGGGTGATCGCCTTGGTGA  19
Template        463703  ...................  463721

Reverse primer  1       GGCCAGTTGCAGCTTGTTC  19
Template        464008  ...................  463990
```

>CP028331.1 Pseudomonas aeruginosa strain PA-VAP-2 chromosome

```
product length = 306
Forward primer  1        TGGGTGATCGCCTTGGTGA  19
Template        5808958  ...................  5808940

Reverse primer  1        GGCCAGTTGCAGCTTGTTC  19
Template        5808653  ...................  5808671
```

>CP028330.1 Pseudomonas aeruginosa strain PA-VAP-3 chromosome

```
product length = 306
Forward primer  1        TGGGTGATCGCCTTGGTGA  19
Template        4878217  ...................  4878199

Reverse primer  1        GGCCAGTTGCAGCTTGTTC  19
Template        4877912  ...................  4877930
```

>CP028368.1 Pseudomonas aeruginosa strain PA-VAP-4 chromosome

```
product length = 306
Forward primer  1        TGGGTGATCGCCTTGGTGA  19
Template        3155106  ...................  3155124

Reverse primer  1        GGCCAGTTGCAGCTTGTTC  19
Template        3155411  ...................  3155393
```

>CP031677.1 Pseudomonas aeruginosa strain E80 chromosome, complete genome

```
product length = 306
Forward primer  1       TGGGTGATCGCCTTGGTGA  19
Template        580366  ...................  580384

Reverse primer  1       GGCCAGTTGCAGCTTGTTC  19
Template        580671  ...................  580653
```

>CP022478.1 Pseudomonas aeruginosa strain LW chromosome, complete genome

```
product length = 306
Forward primer  1        TGGGTGATCGCCTTGGTGA  19
Template        5995702  ...................  5995720

Reverse primer  1        GGCCAGTTGCAGCTTGTTC  19
Template        5996007  ...................  5995989
```

>CP034430.1 Pseudomonas aeruginosa strain GIMC5016:PA1840 chromosome

```
product length = 306
Forward primer  1       TGGGTGATCGCCTTGGTGA  19
Template        616157  ...................  616175

Reverse primer  1       GGCCAGTTGCAGCTTGTTC  19
Template        616462  ...................  616444
```

>CP034429.1 Pseudomonas aeruginosa strain GIMC5015:PAKB6, complete sequence

```
product length = 306
Forward primer  1       TGGGTGATCGCCTTGGTGA  19
Template        472743  ...................  472761

Reverse primer  1       GGCCAGTTGCAGCTTGTTC  19
Template        473048  ...................  473030
```

>LR134342.1 Pseudomonas aeruginosa strain NCTC10728 genome assembly, chromosome: 1

```
product length = 306
Forward primer  1        TGGGTGATCGCCTTGGTGA  19
Template        3451932  ...................  3451950

Reverse primer  1        GGCCAGTTGCAGCTTGTTC  19
Template        3452237  ...................  3452219
```

>LR134330.1 Pseudomonas aeruginosa strain NCTC13715 genome assembly, chromosome: 1

```
product length = 306
Forward primer  1        TGGGTGATCGCCTTGGTGA  19
Template        2331452  ...................  2331470

Reverse primer  1        GGCCAGTTGCAGCTTGTTC  19
Template        2331757  ...................  2331739
```

>LR134309.1 Pseudomonas aeruginosa strain NCTC12903 genome assembly, chromosome: 1

```
product length = 306
Forward primer  1       TGGGTGATCGCCTTGGTGA  19
Template        482889  ...................  482907

Reverse primer  1       GGCCAGTTGCAGCTTGTTC  19
Template        483194  ...................  483176
```

>LR134308.1 Pseudomonas aeruginosa strain NCTC11445 genome assembly, chromosome: 1

```
product length = 306
Forward primer  1        TGGGTGATCGCCTTGGTGA  19
Template        5053053  ...................  5053035

Reverse primer  1        GGCCAGTTGCAGCTTGTTC  19
Template        5052748  ...................  5052766
```

>LR134300.1 Pseudomonas fluorescens strain NCTC10783 genome assembly, chromosome: 1

```
product length = 306
Forward primer  1        TGGGTGATCGCCTTGGTGA  19
Template        6130670  ...................  6130688

Reverse primer  1        GGCCAGTTGCAGCTTGTTC  19
Template        6130975  ...................  6130957
```

>CP032541.1 Pseudomonas aeruginosa strain PGN5 chromosome

```
product length = 306
Forward primer  1       TGGGTGATCGCCTTGGTGA  19
Template        473225  ...................  473243

Reverse primer  1       GGCCAGTTGCAGCTTGTTC  19
Template        473530  ...................  473512
```

>CP032540.1 Pseudomonas aeruginosa strain PGN4 chromosome

```
product length = 306
Forward primer  1       TGGGTGATCGCCTTGGTGA  19
Template        473220  ...................  473238

Reverse primer  1       GGCCAGTTGCAGCTTGTTC  19
Template        473525  ...................  473507
```

>CP034434.1 Pseudomonas aeruginosa strain SP2230 chromosome, complete genome

```
product length = 306
Forward primer  1        TGGGTGATCGCCTTGGTGA  19
Template        6532699  ...................  6532717

Reverse primer  1        GGCCAGTTGCAGCTTGTTC  19
Template        6533004  ...................  6532986
```

>CP034435.1 Pseudomonas aeruginosa strain B14130 chromosome, complete genome

```
product length = 306
Forward primer  1        TGGGTGATCGCCTTGGTGA  19
Template        5889240  ...................  5889222

Reverse primer  1        GGCCAGTTGCAGCTTGTTC  19
Template        5888935  ...................  5888953
```

>CP034436.1 Pseudomonas aeruginosa strain B17932 chromosome, complete genome

```
product length = 306
Forward primer  1        TGGGTGATCGCCTTGGTGA  19
Template        5755991  ...................  5755973

Reverse primer  1        GGCCAGTTGCAGCTTGTTC  19
Template        5755686  ...................  5755704
```

>CP034409.1 Pseudomonas aeruginosa strain SP4527 chromosome, complete genome

```
product length = 306
Forward primer  1        TGGGTGATCGCCTTGGTGA  19
Template        5825163  ...................  5825181

Reverse primer  1        GGCCAGTTGCAGCTTGTTC  19
Template        5825468  ...................  5825450
```

>CP034369.1 Pseudomonas aeruginosa strain SP4371 chromosome, complete genome

```
product length = 306
Forward primer  1        TGGGTGATCGCCTTGGTGA  19
Template        6719184  ...................  6719202

Reverse primer  1        GGCCAGTTGCAGCTTGTTC  19
Template        6719489  ...................  6719471
```

>CP034368.1 Pseudomonas aeruginosa strain B41226 chromosome, complete genome

```
product length = 306
Forward primer  1       TGGGTGATCGCCTTGGTGA  19
Template        180157  ...................  180175

Reverse primer  1       GGCCAGTTGCAGCTTGTTC  19
Template        180462  ...................  180444
```

>CP034354.1 Pseudomonas aeruginosa strain IMP-13 chromosome, complete genome

```
product length = 306
Forward primer  1        TGGGTGATCGCCTTGGTGA  19
Template        3570969  ...................  3570987

Reverse primer  1        GGCCAGTTGCAGCTTGTTC  19
Template        3571274  ...................  3571256
```

>LR130537.1 Pseudomonas aeruginosa isolate paerg012 genome assembly, chromosome: 0

```
product length = 306
Forward primer  1       TGGGTGATCGCCTTGGTGA  19
Template        476685  ...................  476703

Reverse primer  1       GGCCAGTTGCAGCTTGTTC  19
Template        476990  ...................  476972
```

>LR130536.1 Pseudomonas aeruginosa isolate paerg010 genome assembly, chromosome: 0

```
product length = 306
Forward primer  1       TGGGTGATCGCCTTGGTGA  19
Template        476705  ...................  476723

Reverse primer  1       GGCCAGTTGCAGCTTGTTC  19
Template        477010  ...................  476992
```

>LR130535.1 Pseudomonas aeruginosa isolate paerg011 genome assembly, chromosome: 0

```
product length = 306
Forward primer  1       TGGGTGATCGCCTTGGTGA  19
Template        476710  ...................  476728

Reverse primer  1       GGCCAGTTGCAGCTTGTTC  19
Template        477015  ...................  476997
```

>LR130534.1 Pseudomonas aeruginosa isolate paerg005 genome assembly, chromosome: 0

```
product length = 306
Forward primer  1       TGGGTGATCGCCTTGGTGA  19
Template        549300  ...................  549318

Reverse primer  1       GGCCAGTTGCAGCTTGTTC  19
Template        549605  ...................  549587
```

>LR130533.1 Pseudomonas aeruginosa isolate paerg009 genome assembly, chromosome: 0

```
product length = 306
Forward primer  1        TGGGTGATCGCCTTGGTGA  19
Template        3868309  ...................  3868327

Reverse primer  1        GGCCAGTTGCAGCTTGTTC  19
Template        3868614  ...................  3868596
```

>LR130531.1 Pseudomonas aeruginosa isolate paerg004 genome assembly, chromosome: 0

```
product length = 306
Forward primer  1        TGGGTGATCGCCTTGGTGA  19
Template        4228317  ...................  4228335

Reverse primer  1        GGCCAGTTGCAGCTTGTTC  19
Template        4228622  ...................  4228604
```

>LR130530.1 Pseudomonas aeruginosa isolate paerg003 genome assembly, chromosome: 0

```
product length = 306
Forward primer  1       TGGGTGATCGCCTTGGTGA  19
Template        476668  ...................  476686

Reverse primer  1       GGCCAGTTGCAGCTTGTTC  19
Template        476973  ...................  476955
```

>LR130528.1 Pseudomonas aeruginosa isolate paerg000 genome assembly, chromosome: 0

```
product length = 306
Forward primer  1       TGGGTGATCGCCTTGGTGA  19
Template        466414  ...................  466432

Reverse primer  1       GGCCAGTTGCAGCTTGTTC  19
Template        466719  ...................  466701
```

>LR130527.1 Pseudomonas aeruginosa isolate paerg002 genome assembly, chromosome: 0

```
product length = 306
Forward primer  1        TGGGTGATCGCCTTGGTGA  19
Template        3591203  ...................  3591221

Reverse primer  1        GGCCAGTTGCAGCTTGTTC  19
Template        3591508  ...................  3591490
```

>CP033832.1 Pseudomonas aeruginosa strain FDAARGOS_505 chromosome, complete genome

```
product length = 306
Forward primer  1        TGGGTGATCGCCTTGGTGA  19
Template        1972476  ...................  1972494

Reverse primer  1        GGCCAGTTGCAGCTTGTTC  19
Template        1972781  ...................  1972763
```

>CP033835.1 Pseudomonas aeruginosa strain FDAARGOS_570 chromosome, complete genome

```
product length = 306
Forward primer  1        TGGGTGATCGCCTTGGTGA  19
Template        6691944  ...................  6691926

Reverse primer  1        GGCCAGTTGCAGCTTGTTC  19
Template        6691639  ...................  6691657
```

>CP033833.1 Pseudomonas aeruginosa strain FDAARGOS_571 chromosome, complete genome

```
product length = 306
Forward primer  1        TGGGTGATCGCCTTGGTGA  19
Template        3741217  ...................  3741235

Reverse primer  1        GGCCAGTTGCAGCTTGTTC  19
Template        3741522  ...................  3741504
```

>CP033843.1 Pseudomonas aeruginosa strain FDAARGOS_501 chromosome, complete genome

```
product length = 306
Forward primer  1        TGGGTGATCGCCTTGGTGA  19
Template        2206170  ...................  2206152

Reverse primer  1        GGCCAGTTGCAGCTTGTTC  19
Template        2205865  ...................  2205883
```

>CP033771.1 Pseudomonas aeruginosa strain FDAARGOS_532 chromosome, complete genome

```
product length = 306
Forward primer  1        TGGGTGATCGCCTTGGTGA  19
Template        5304206  ...................  5304224

Reverse primer  1        GGCCAGTTGCAGCTTGTTC  19
Template        5304511  ...................  5304493
```

>CP033684.1 Pseudomonas aeruginosa strain H26027 chromosome, complete genome

```
product length = 306
Forward primer  1       TGGGTGATCGCCTTGGTGA  19
Template        518502  ...................  518520

Reverse primer  1       GGCCAGTTGCAGCTTGTTC  19
Template        518807  ...................  518789
```

>CP033686.1 Pseudomonas aeruginosa strain H25883 chromosome, complete genome

```
product length = 306
Forward primer  1       TGGGTGATCGCCTTGGTGA  19
Template        473082  ...................  473100

Reverse primer  1       GGCCAGTTGCAGCTTGTTC  19
Template        473387  ...................  473369
```

>CP033685.1 Pseudomonas aeruginosa strain H26023 chromosome, complete genome

```
product length = 306
Forward primer  1       TGGGTGATCGCCTTGGTGA  19
Template        487490  ...................  487508

Reverse primer  1       GGCCAGTTGCAGCTTGTTC  19
Template        487795  ...................  487777
```

>CP029713.1 Pseudomonas aeruginosa strain BH9 chromosome

```
product length = 306
Forward primer  1       TGGGTGATCGCCTTGGTGA  19
Template        466464  ...................  466482

Reverse primer  1       GGCCAGTTGCAGCTTGTTC  19
Template        466769  ...................  466751
```

>CP033439.1 Pseudomonas aeruginosa strain SP4528 chromosome, complete genome

```
product length = 306
Forward primer  1        TGGGTGATCGCCTTGGTGA  19
Template        6032317  ...................  6032299

Reverse primer  1        GGCCAGTTGCAGCTTGTTC  19
Template        6032012  ...................  6032030
```

>CP033432.1 Pseudomonas aeruginosa strain BA15561 chromosome, complete genome

```
product length = 306
Forward primer  1        TGGGTGATCGCCTTGGTGA  19
Template        4176085  ...................  4176067

Reverse primer  1        GGCCAGTTGCAGCTTGTTC  19
Template        4175780  ...................  4175798
```

>CP033084.1 Pseudomonas aeruginosa strain PA-3 chromosome, complete genome

```
product length = 306
Forward primer  1        TGGGTGATCGCCTTGGTGA  19
Template        4066879  ...................  4066861

Reverse primer  1        GGCCAGTTGCAGCTTGTTC  19
Template        4066574  ...................  4066592
```

>CP030075.1 Pseudomonas aeruginosa strain 6762 chromosome

```
product length = 306
Forward primer  1        TGGGTGATCGCCTTGGTGA  19
Template        2583143  ...................  2583161

Reverse primer  1        GGCCAGTTGCAGCTTGTTC  19
Template        2583448  ...................  2583430
```

>CP032552.1 Pseudomonas aeruginosa strain PA34 chromosome, complete genome

```
product length = 306
Forward primer  1       TGGGTGATCGCCTTGGTGA  19
Template        477930  ...................  477948

Reverse primer  1       GGCCAGTTGCAGCTTGTTC  19
Template        478235  ...................  478217
```

>CP032761.1 Pseudomonas aeruginosa strain 268 chromosome, complete genome

```
product length = 306
Forward primer  1       TGGGTGATCGCCTTGGTGA  19
Template        558451  ...................  558469

Reverse primer  1       GGCCAGTTGCAGCTTGTTC  19
Template        558756  ...................  558738
```

>CP028584.2 Pseudomonas aeruginosa strain WCHPA075019 chromosome, complete genome

```
product length = 306
Forward primer  1       TGGGTGATCGCCTTGGTGA  19
Template        471753  ...................  471771

Reverse primer  1       GGCCAGTTGCAGCTTGTTC  19
Template        472058  ...................  472040
```

>CP032257.1 Pseudomonas aeruginosa strain AR_0111 chromosome, complete genome

```
product length = 306
Forward primer  1        TGGGTGATCGCCTTGGTGA  19
Template        3818297  ...................  3818315

Reverse primer  1        GGCCAGTTGCAGCTTGTTC  19
Template        3818602  ...................  3818584
```

>CP031877.1 Pseudomonas aeruginosa strain WPB100 chromosome

```
product length = 306
Forward primer  1       TGGGTGATCGCCTTGGTGA  19
Template        273867  ...................  273849

Reverse primer  1       GGCCAGTTGCAGCTTGTTC  19
Template        273562  ...................  273580
```

>CP031876.1 Pseudomonas aeruginosa strain WPB101 chromosome

```
product length = 306
Forward primer  1       TGGGTGATCGCCTTGGTGA  19
Template        241717  ...................  241699

Reverse primer  1       GGCCAGTTGCAGCTTGTTC  19
Template        241412  ...................  241430
```

>CP031878.1 Pseudomonas aeruginosa strain WPB099 chromosome

```
product length = 306
Forward primer  1       TGGGTGATCGCCTTGGTGA  19
Template        241717  ...................  241699

Reverse primer  1       GGCCAGTTGCAGCTTGTTC  19
Template        241412  ...................  241430
```

>CP031879.1 Pseudomonas aeruginosa strain WPB098 chromosome

```
product length = 306
Forward primer  1        TGGGTGATCGCCTTGGTGA  19
Template        4722333  ...................  4722315

Reverse primer  1        GGCCAGTTGCAGCTTGTTC  19
Template        4722028  ...................  4722046
```

>CP029605.1 Pseudomonas aeruginosa strain 24Pae112 chromosome, complete genome

```
product length = 306
Forward primer  1       TGGGTGATCGCCTTGGTGA  19
Template        511260  ...................  511278

Reverse primer  1       GGCCAGTTGCAGCTTGTTC  19
Template        511565  ...................  511547
```

>CP031660.1 Pseudomonas aeruginosa strain PABL017 chromosome, complete genome

```
product length = 306
Forward primer  1       TGGGTGATCGCCTTGGTGA  19
Template        471464  ...................  471482

Reverse primer  1       GGCCAGTTGCAGCTTGTTC  19
Template        471769  ...................  471751
```

>CP031659.1 Pseudomonas aeruginosa strain PABL012 chromosome, complete genome

```
product length = 306
Forward primer  1       TGGGTGATCGCCTTGGTGA  19
Template        475965  ...................  475983

Reverse primer  1       GGCCAGTTGCAGCTTGTTC  19
Template        476270  ...................  476252
```

>CP030911.1 Pseudomonas aeruginosa strain Y71 chromosome, complete genome

```
product length = 306
Forward primer  1       TGGGTGATCGCCTTGGTGA  19
Template        490856  ...................  490874

Reverse primer  1       GGCCAGTTGCAGCTTGTTC  19
Template        491161  ...................  491143
```

>CP035739.1 Pseudomonas aeruginosa strain 1334/14 chromosome, complete genome

```
product length = 306
Forward primer  1        TGGGTGATCGCCTTGGTGA  19
Template        3611535  ...................  3611517

Reverse primer  1        GGCCAGTTGCAGCTTGTTC  19
Template        3611230  ...................  3611248
```

>CP031449.2 Pseudomonas aeruginosa strain 97 chromosome, complete genome

```
product length = 306
Forward primer  1       TGGGTGATCGCCTTGGTGA  19
Template        488340  ...................  488358

Reverse primer  1       GGCCAGTTGCAGCTTGTTC  19
Template        488645  ...................  488627
```

>LS998783.1 Pseudomonas aeruginosa isolate 1 genome assembly, chromosome: 1

```
product length = 306
Forward primer  1       TGGGTGATCGCCTTGGTGA  19
Template        531327  ...................  531345

Reverse primer  1       GGCCAGTTGCAGCTTGTTC  19
Template        531632  ...................  531614
```

>CP032126.1 Pseudomonas aeruginosa strain PAO1161 chromosome, complete genome

```
product length = 306
Forward primer  1       TGGGTGATCGCCTTGGTGA  19
Template        473225  ...................  473243

Reverse primer  1       GGCCAGTTGCAGCTTGTTC  19
Template        473530  ...................  473512
```

>CP030913.1 Pseudomonas aeruginosa strain Y89 chromosome, complete genome

```
product length = 306
Forward primer  1       TGGGTGATCGCCTTGGTGA  19
Template        491509  ...................  491527

Reverse primer  1       GGCCAGTTGCAGCTTGTTC  19
Template        491814  ...................  491796
```

>CP030912.1 Pseudomonas aeruginosa strain Y82 chromosome, complete genome

```
product length = 306
Forward primer  1       TGGGTGATCGCCTTGGTGA  19
Template        534822  ...................  534840

Reverse primer  1       GGCCAGTTGCAGCTTGTTC  19
Template        535127  ...................  535109
```

>CP030910.1 Pseudomonas aeruginosa strain Y31 chromosome, complete genome

```
product length = 306
Forward primer  1       TGGGTGATCGCCTTGGTGA  19
Template        470835  ...................  470853

Reverse primer  1       GGCCAGTTGCAGCTTGTTC  19
Template        471140  ...................  471122
```

>CP030861.1 Pseudomonas aeruginosa strain HS9 chromosome, complete genome

```
product length = 306
Forward primer  1        TGGGTGATCGCCTTGGTGA  19
Template        2064964  ...................  2064946

Reverse primer  1        GGCCAGTTGCAGCTTGTTC  19
Template        2064659  ...................  2064677
```

>CP030327.1 Pseudomonas aeruginosa strain AR_458 chromosome, complete genome

```
product length = 306
Forward primer  1        TGGGTGATCGCCTTGGTGA  19
Template        2568728  ...................  2568710

Reverse primer  1        GGCCAGTTGCAGCTTGTTC  19
Template        2568423  ...................  2568441
```

>CP030351.1 Pseudomonas aeruginosa strain AR_460 chromosome, complete genome

```
product length = 306
Forward primer  1        TGGGTGATCGCCTTGGTGA  19
Template        4172505  ...................  4172487

Reverse primer  1        GGCCAGTTGCAGCTTGTTC  19
Template        4172200  ...................  4172218
```

>CP030328.1 Pseudomonas aeruginosa strain AR_455 chromosome, complete genome

```
product length = 306
Forward primer  1        TGGGTGATCGCCTTGGTGA  19
Template        1838312  ...................  1838330

Reverse primer  1        GGCCAGTTGCAGCTTGTTC  19
Template        1838617  ...................  1838599
```

>LS483497.1 Pseudomonas aeruginosa strain NCTC9433 genome assembly, chromosome: 1

```
product length = 306
Forward primer  1       TGGGTGATCGCCTTGGTGA  19
Template        454565  ...................  454583

Reverse primer  1       GGCCAGTTGCAGCTTGTTC  19
Template        454870  ...................  454852
```

>CP029707.1 Pseudomonas aeruginosa strain K34-7 chromosome, complete genome

```
product length = 306
Forward primer  1        TGGGTGATCGCCTTGGTGA  19
Template        6055993  ...................  6056011

Reverse primer  1        GGCCAGTTGCAGCTTGTTC  19
Template        6056298  ...................  6056280
```

>CP029745.1 Pseudomonas aeruginosa strain AR_0110 chromosome, complete genome

```
product length = 306
Forward primer  1       TGGGTGATCGCCTTGGTGA  19
Template        215182  ...................  215164

Reverse primer  1       GGCCAGTTGCAGCTTGTTC  19
Template        214877  ...................  214895
```

>CP023255.1 Pseudomonas aeruginosa strain CCUG 70744 chromosome, complete genome

```
product length = 306
Forward primer  1        TGGGTGATCGCCTTGGTGA  19
Template        5255194  ...................  5255212

Reverse primer  1        GGCCAGTTGCAGCTTGTTC  19
Template        5255499  ...................  5255481
```

>CP029660.1 Pseudomonas aeruginosa strain AR_0446 chromosome, complete genome

```
product length = 306
Forward primer  1        TGGGTGATCGCCTTGGTGA  19
Template        5618727  ...................  5618745

Reverse primer  1        GGCCAGTTGCAGCTTGTTC  19
Template        5619032  ...................  5619014
```

>CP029148.1 Pseudomonas aeruginosa strain AR_0440 chromosome

```
product length = 306
Forward primer  1        TGGGTGATCGCCTTGGTGA  19
Template        6704442  ...................  6704460

Reverse primer  1        GGCCAGTTGCAGCTTGTTC  19
Template        6704747  ...................  6704729
```

>CP029147.1 Pseudomonas aeruginosa strain AR_0443 chromosome

```
product length = 306
Forward primer  1        TGGGTGATCGCCTTGGTGA  19
Template        4135326  ...................  4135344

Reverse primer  1        GGCCAGTTGCAGCTTGTTC  19
Template        4135631  ...................  4135613
```

>CP029097.1 Pseudomonas aeruginosa strain AR439 chromosome, complete genome

```
product length = 306
Forward primer  1        TGGGTGATCGCCTTGGTGA  19
Template        6816131  ...................  6816113

Reverse primer  1        GGCCAGTTGCAGCTTGTTC  19
Template        6815826  ...................  6815844
```

>CP029090.1 Pseudomonas aeruginosa strain AR442 chromosome, complete genome

```
product length = 306
Forward primer  1        TGGGTGATCGCCTTGGTGA  19
Template        6705674  ...................  6705692

Reverse primer  1        GGCCAGTTGCAGCTTGTTC  19
Template        6705979  ...................  6705961
```

>CP029089.1 Pseudomonas aeruginosa strain AR444 chromosome, complete genome

```
product length = 306
Forward primer  1        TGGGTGATCGCCTTGGTGA  19
Template        6212575  ...................  6212593

Reverse primer  1        GGCCAGTTGCAGCTTGTTC  19
Template        6212880  ...................  6212862
```

>CP029088.1 Pseudomonas aeruginosa strain AR445 chromosome, complete genome

```
product length = 306
Forward primer  1       TGGGTGATCGCCTTGGTGA  19
Template        527416  ...................  527434

Reverse primer  1       GGCCAGTTGCAGCTTGTTC  19
Template        527721  ...................  527703
```

>CP028917.1 Pseudomonas aeruginosa strain JB2 chromosome, complete genome

```
product length = 306
Forward primer  1       TGGGTGATCGCCTTGGTGA  19
Template        459961  ...................  459979

Reverse primer  1       GGCCAGTTGCAGCTTGTTC  19
Template        460266  ...................  460248
```

>CP023316.1 Pseudomonas aeruginosa strain PPF-1 chromosome, complete genome

```
product length = 306
Forward primer  1       TGGGTGATCGCCTTGGTGA  19
Template        536728  ...................  536746

Reverse primer  1       GGCCAGTTGCAGCTTGTTC  19
Template        537033  ...................  537015
```

>CP028162.1 Pseudomonas aeruginosa strain MRSN12280 chromosome, complete genome

```
product length = 306
Forward primer  1       TGGGTGATCGCCTTGGTGA  19
Template        513213  ...................  513231

Reverse primer  1       GGCCAGTTGCAGCTTGTTC  19
Template        513518  ...................  513500
```

>CP027538.1 Pseudomonas aeruginosa strain AR_0095 chromosome, complete genome

```
product length = 306
Forward primer  1        TGGGTGATCGCCTTGGTGA  19
Template        2566691  ...................  2566673

Reverse primer  1        GGCCAGTTGCAGCTTGTTC  19
Template        2566386  ...................  2566404
```

>CP027166.1 Pseudomonas aeruginosa strain AR_0357 chromosome, complete genome

```
product length = 306
Forward primer  1        TGGGTGATCGCCTTGGTGA  19
Template        1196176  ...................  1196158

Reverse primer  1        GGCCAGTTGCAGCTTGTTC  19
Template        1195871  ...................  1195889
```

>CP027174.1 Pseudomonas aeruginosa strain AR_0230 chromosome, complete genome

```
product length = 306
Forward primer  1        TGGGTGATCGCCTTGGTGA  19
Template        6066891  ...................  6066909

Reverse primer  1        GGCCAGTTGCAGCTTGTTC  19
Template        6067196  ...................  6067178
```

>CP027172.1 Pseudomonas aeruginosa strain AR_0353 chromosome, complete genome

```
product length = 306
Forward primer  1        TGGGTGATCGCCTTGGTGA  19
Template        6806420  ...................  6806402

Reverse primer  1        GGCCAGTTGCAGCTTGTTC  19
Template        6806115  ...................  6806133
```

>CP027171.1 Pseudomonas aeruginosa strain AR_0354 chromosome, complete genome

```
product length = 306
Forward primer  1        TGGGTGATCGCCTTGGTGA  19
Template        5136021  ...................  5136039

Reverse primer  1        GGCCAGTTGCAGCTTGTTC  19
Template        5136326  ...................  5136308
```

>CP027165.1 Pseudomonas aeruginosa strain AR_0360 chromosome, complete genome

```
product length = 306
Forward primer  1        TGGGTGATCGCCTTGGTGA  19
Template        5702886  ...................  5702904

Reverse primer  1        GGCCAGTTGCAGCTTGTTC  19
Template        5703191  ...................  5703173
```

>CP026680.1 Pseudomonas aeruginosa strain F5677 chromosome, complete genome

```
product length = 306
Forward primer  1       TGGGTGATCGCCTTGGTGA  19
Template        476780  ...................  476798

Reverse primer  1       GGCCAGTTGCAGCTTGTTC  19
Template        477085  ...................  477067
```

>LT969520.1 Pseudomonas aeruginosa isolate RW109 genome assembly, chromosome: Main_chromosome

```
product length = 306
Forward primer  1       TGGGTGATCGCCTTGGTGA  19
Template        486189  ...................  486207

Reverse primer  1       GGCCAGTTGCAGCTTGTTC  19
```

```
Template        486494  ...................  486476
```

>CP025229.1 Pseudomonas sp. AK6U chromosome, complete genome

```
product length = 306
Forward primer  1        TGGGTGATCGCCTTGGTGA  19
Template        4537227  ...................  4537209

Reverse primer  1        GGCCAGTTGCAGCTTGTTC  19
Template        4536922  ...................  4536940
```

>CP025051.1 Pseudomonas aeruginosa strain PB353 chromosome, complete genome

```
product length = 306
Forward primer  1       TGGGTGATCGCCTTGGTGA  19
Template        469379  ...................  469397

Reverse primer  1       GGCCAGTTGCAGCTTGTTC  19
Template        469684  ...................  469666
```

>CP025050.1 Pseudomonas aeruginosa strain PB368 chromosome, complete genome

```
product length = 306
Forward primer  1       TGGGTGATCGCCTTGGTGA  19
Template        355164  ...................  355146

Reverse primer  1       GGCCAGTTGCAGCTTGTTC  19
Template        354859  ...................  354877
```

>CP025049.1 Pseudomonas aeruginosa strain PB369 chromosome, complete genome

```
product length = 306
Forward primer  1       TGGGTGATCGCCTTGGTGA  19
Template        457852  ...................  457870

Reverse primer  1       GGCCAGTTGCAGCTTGTTC  19
Template        458157  ...................  458139
```

>CP025053.1 Pseudomonas aeruginosa strain PB354 chromosome, complete genome

```
product length = 306
Forward primer  1       TGGGTGATCGCCTTGGTGA  19
Template        469379  ...................  469397

Reverse primer  1       GGCCAGTTGCAGCTTGTTC  19
Template        469684  ...................  469666
```

>CP024477.1 Pseudomonas aeruginosa strain 12939 chromosome, complete genome

```
product length = 306
Forward primer  1       TGGGTGATCGCCTTGGTGA  19
Template        477631  ...................  477649
```

```
Reverse primer  1       GGCCAGTTGCAGCTTGTTC  19
Template        477936  ...................  477918
```

>CP017306.1 Pseudomonas aeruginosa strain PA_150577 chromosome, complete genome

```
product length = 306
Forward primer  1       TGGGTGATCGCCTTGGTGA  19
Template        467031  ...................  467049

Reverse primer  1       GGCCAGTTGCAGCTTGTTC  19
Template        467336  ...................  467318
```

>CP022526.1 Pseudomonas aeruginosa strain Ocean-1155, complete genome

```
product length = 306
Forward primer  1        TGGGTGATCGCCTTGGTGA  19
Template        4227547  ...................  4227565

Reverse primer  1        GGCCAGTTGCAGCTTGTTC  19
Template        4227852  ...................  4227834
```

>CP022525.1 Pseudomonas aeruginosa strain Ocean-1175, complete genome

```
product length = 306
Forward primer  1        TGGGTGATCGCCTTGGTGA  19
Template        1387044  ...................  1387026

Reverse primer  1        GGCCAGTTGCAGCTTGTTC  19
Template        1386739  ...................  1386757
```

>CP019338.1 Pseudomonas aeruginosa strain L10, complete genome

```
product length = 306
Forward primer  1       TGGGTGATCGCCTTGGTGA  19
Template        487453  ...................  487471

Reverse primer  1       GGCCAGTTGCAGCTTGTTC  19
Template        487758  ...................  487740
```

>CP017293.1 Pseudomonas aeruginosa strain PA83, complete genome

```
product length = 306
Forward primer  1       TGGGTGATCGCCTTGGTGA  19
Template        515093  ...................  515111

Reverse primer  1       GGCCAGTTGCAGCTTGTTC  19
Template        515398  ...................  515380
```

>CP022002.1 Pseudomonas aeruginosa strain Pa1242, complete genome

```
product length = 306
Forward primer  1       TGGGTGATCGCCTTGGTGA  19
Template        472221  ...................  472239
```

```
Reverse primer  1       GGCCAGTTGCAGCTTGTTC  19
Template        472526  ...................  472508
```

>CP022001.1 Pseudomonas aeruginosa strain Pa1207, complete genome

```
product length = 306
Forward primer  1       TGGGTGATCGCCTTGGTGA  19
Template        485810  ...................  485828

Reverse primer  1       GGCCAGTTGCAGCTTGTTC  19
Template        486115  ...................  486097
```

>CP022000.1 Pseudomonas aeruginosa strain Pa127, complete genome

```
product length = 306
Forward primer  1       TGGGTGATCGCCTTGGTGA  19
Template        607254  ...................  607272

Reverse primer  1       GGCCAGTTGCAGCTTGTTC  19
Template        607559  ...................  607541
```

>CP021999.1 Pseudomonas aeruginosa strain Pa84, complete genome

```
product length = 306
Forward primer  1       TGGGTGATCGCCTTGGTGA  19
Template        491768  ...................  491786

Reverse primer  1       GGCCAGTTGCAGCTTGTTC  19
Template        492073  ...................  492055
```

>LT883143.1 Pseudomonas aeruginosa C-NN2 isolate early isolate NN2 (clone C) genome assembly, chromosome: I

```
product length = 306
Forward primer  1       TGGGTGATCGCCTTGGTGA  19
Template        518991  ...................  519009

Reverse primer  1       GGCCAGTTGCAGCTTGTTC  19
Template        519296  ...................  519278
```

>CP021774.1 Pseudomonas aeruginosa strain Pa124, complete genome

```
product length = 306
Forward primer  1       TGGGTGATCGCCTTGGTGA  19
Template        607511  ...................  607529

Reverse primer  1       GGCCAGTTGCAGCTTGTTC  19
Template        607816  ...................  607798
```

>CP021775.1 Pseudomonas aeruginosa strain Pa58, complete genome

```
product length = 306
```

```
Forward primer  1       TGGGTGATCGCCTTGGTGA  19
Template        549737  ...................  549755

Reverse primer  1       GGCCAGTTGCAGCTTGTTC  19
Template        550042  ...................  550024
```

>CP015650.1 Pseudomonas aeruginosa strain Pb18 genome

```
product length = 306
Forward primer  1        TGGGTGATCGCCTTGGTGA  19
Template        4896466  ...................  4896484

Reverse primer  1        GGCCAGTTGCAGCTTGTTC  19
Template        4896771  ...................  4896753
```

>CP015649.1 Pseudomonas aeruginosa strain M28A1 genome

```
product length = 306
Forward primer  1        TGGGTGATCGCCTTGGTGA  19
Template        3774636  ...................  3774654

Reverse primer  1        GGCCAGTTGCAGCTTGTTC  19
Template        3774941  ...................  3774923
```

>CP015648.1 Pseudomonas aeruginosa strain M8A4 genome

```
product length = 306
Forward primer  1        TGGGTGATCGCCTTGGTGA  19
Template        5925784  ...................  5925766

Reverse primer  1        GGCCAGTTGCAGCTTGTTC  19
Template        5925479  ...................  5925497
```

>CP015647.1 Pseudomonas aeruginosa strain M8A1 genome

```
product length = 306
Forward primer  1       TGGGTGATCGCCTTGGTGA  19
Template        413379  ...................  413397

Reverse primer  1       GGCCAGTTGCAGCTTGTTC  19
Template        413684  ...................  413666
```

>CP020704.1 Pseudomonas aeruginosa strain PASGNDM699, complete genome

```
product length = 306
Forward primer  1       TGGGTGATCGCCTTGGTGA  19
Template        478906  ...................  478924

Reverse primer  1       GGCCAGTTGCAGCTTGTTC  19
Template        479211  ...................  479193
```

>CP020703.1 Pseudomonas aeruginosa strain PASGNDM345, complete genome

```
product length = 306
Forward primer  1       TGGGTGATCGCCTTGGTGA  19
Template        478904  ...................  478922

Reverse primer  1       GGCCAGTTGCAGCTTGTTC  19
Template        479209  ...................  479191
```

>CP008858.2 Pseudomonas aeruginosa strain F63912 chromosome, complete genome

```
product length = 306
Forward primer  1       TGGGTGATCGCCTTGGTGA  19
Template        467848  ...................  467866

Reverse primer  1       GGCCAGTTGCAGCTTGTTC  19
Template        468153  ...................  468135
```

>CP020659.1 Pseudomonas aeruginosa PAK chromosome, complete genome

```
product length = 306
Forward primer  1        TGGGTGATCGCCTTGGTGA  19
Template        6038949  ...................  6038967

Reverse primer  1        GGCCAGTTGCAGCTTGTTC  19
Template        6039254  ...................  6039236
```

>CP008872.2 Pseudomonas aeruginosa strain X78812 chromosome, complete genome

```
product length = 306
Forward primer  1       TGGGTGATCGCCTTGGTGA  19
Template        474894  ...................  474912

Reverse primer  1       GGCCAGTTGCAGCTTGTTC  19
Template        475199  ...................  475181
```

>CP008871.2 Pseudomonas aeruginosa strain W45909 chromosome, complete genome

```
product length = 306
Forward primer  1       TGGGTGATCGCCTTGGTGA  19
Template        472455  ...................  472473

Reverse primer  1       GGCCAGTTGCAGCTTGTTC  19
Template        472760  ...................  472742
```

>CP008870.2 Pseudomonas aeruginosa strain W36662 chromosome, complete genome

```
product length = 306
Forward primer  1       TGGGTGATCGCCTTGGTGA  19
Template        580358  ...................  580376

Reverse primer  1       GGCCAGTTGCAGCTTGTTC  19
Template        580663  ...................  580645
```

>CP008869.2 Pseudomonas aeruginosa strain W16407 chromosome, complete genome

```
product length = 306
Forward primer  1       TGGGTGATCGCCTTGGTGA  19
Template        468539  ...................  468557

Reverse primer  1       GGCCAGTTGCAGCTTGTTC  19
Template        468844  ...................  468826
```

>CP008866.2 Pseudomonas aeruginosa strain T38079 chromosome, complete genome

```
product length = 306
Forward primer  1       TGGGTGATCGCCTTGGTGA  19
Template        485823  ...................  485841

Reverse primer  1       GGCCAGTTGCAGCTTGTTC  19
Template        486128  ...................  486110
```

>CP008865.2 Pseudomonas aeruginosa strain S86968 chromosome, complete genome

```
product length = 306
Forward primer  1       TGGGTGATCGCCTTGGTGA  19
Template        484596  ...................  484614

Reverse primer  1       GGCCAGTTGCAGCTTGTTC  19
Template        484901  ...................  484883
```

>CP008864.2 Pseudomonas aeruginosa strain W60856 chromosome, complete genome

```
product length = 306
Forward primer  1       TGGGTGATCGCCTTGGTGA  19
Template        482753  ...................  482771

Reverse primer  1       GGCCAGTTGCAGCTTGTTC  19
Template        483058  ...................  483040
```

>CP008862.2 Pseudomonas aeruginosa strain M1608 chromosome, complete genome

```
product length = 306
Forward primer  1       TGGGTGATCGCCTTGGTGA  19
Template        526111  ...................  526129

Reverse primer  1       GGCCAGTTGCAGCTTGTTC  19
Template        526416  ...................  526398
```

>CP008860.2 Pseudomonas aeruginosa strain H27930 chromosome, complete genome

```
product length = 306
Forward primer  1       TGGGTGATCGCCTTGGTGA  19
Template        483687  ...................  483705

Reverse primer  1       GGCCAGTTGCAGCTTGTTC  19
Template        483992  ...................  483974
```

>CP008859.2 Pseudomonas aeruginosa strain H5708 chromosome, complete genome

```
product length = 306
Forward primer  1       TGGGTGATCGCCTTGGTGA  19
Template        507775  ...................  507793

Reverse primer  1       GGCCAGTTGCAGCTTGTTC  19
Template        508080  ...................  508062
```

>CP008856.2 Pseudomonas aeruginosa strain F23197 chromosome, complete genome

```
product length = 306
Forward primer  1       TGGGTGATCGCCTTGGTGA  19
Template        477014  ...................  477032

Reverse primer  1       GGCCAGTTGCAGCTTGTTC  19
Template        477319  ...................  477301
```

>CP020603.1 Pseudomonas aeruginosa strain E6130952, complete genome

```
product length = 306
Forward primer  1       TGGGTGATCGCCTTGGTGA  19
Template        474119  ...................  474137

Reverse primer  1       GGCCAGTTGCAGCTTGTTC  19
Template        474424  ...................  474406
```

>CP016955.1 Pseudomonas aeruginosa strain RIVM-EMC2982, complete genome

```
product length = 306
Forward primer  1        TGGGTGATCGCCTTGGTGA  19
Template        6824516  ...................  6824498

Reverse primer  1        GGCCAGTTGCAGCTTGTTC  19
Template        6824211  ...................  6824229
```

>CP014866.1 Pseudomonas aeruginosa strain PA_154197 chromosome, complete genome

```
product length = 306
Forward primer  1       TGGGTGATCGCCTTGGTGA  19
Template        461339  ...................  461357

Reverse primer  1       GGCCAGTTGCAGCTTGTTC  19
Template        461644  ...................  461626
```

>LT673656.1 Pseudomonas aeruginosa isolate PcyII-10 genome assembly, chromosome: PcyII-10

```
product length = 306
Forward primer  1       TGGGTGATCGCCTTGGTGA  19
Template        465626  ...................  465644

Reverse primer  1       GGCCAGTTGCAGCTTGTTC  19
Template        465931  ...................  465913
```

>CP013479.1 Pseudomonas aeruginosa strain NHmuc chromosome, complete genome

```
product length = 306
Forward primer  1       TGGGTGATCGCCTTGGTGA  19
Template        478895  ...................  478913

Reverse primer  1       GGCCAGTTGCAGCTTGTTC  19
Template        479200  ...................  479182
```

>CP013478.1 Pseudomonas aeruginosa strain SCVJan chromosome, complete genome

```
product length = 306
Forward primer  1       TGGGTGATCGCCTTGGTGA  19
Template        478895  ...................  478913

Reverse primer  1       GGCCAGTTGCAGCTTGTTC  19
Template        479200  ...................  479182
```

>CP013477.1 Pseudomonas aeruginosa strain SCVFeb chromosome, complete genome

```
product length = 306
Forward primer  1       TGGGTGATCGCCTTGGTGA  19
Template        478895  ...................  478913

Reverse primer  1       GGCCAGTTGCAGCTTGTTC  19
Template        479200  ...................  479182
```

>CP013113.1 Pseudomonas aeruginosa strain PAER4_119 chromosome, complete genome

```
product length = 306
Forward primer  1       TGGGTGATCGCCTTGGTGA  19
Template        474794  ...................  474812

Reverse primer  1       GGCCAGTTGCAGCTTGTTC  19
Template        475099  ...................  475081
```

>CP017969.1 Pseudomonas aeruginosa isolate B10W chromosome, complete genome

```
product length = 306
Forward primer  1       TGGGTGATCGCCTTGGTGA  19
Template        222537  ...................  222555

Reverse primer  1       GGCCAGTTGCAGCTTGTTC  19
Template        222842  ...................  222824
```

>CP014999.1 Pseudomonas aeruginosa strain PA7790, complete genome

```
product length = 306
Forward primer  1       TGGGTGATCGCCTTGGTGA  19
Template        472276  ...................  472294

Reverse primer  1       GGCCAGTTGCAGCTTGTTC  19
Template        472581  ...................  472563
```

>CP015003.1 Pseudomonas aeruginosa strain PA11803 chromosome, complete genome

```
product length = 306
Forward primer  1       TGGGTGATCGCCTTGGTGA  19
Template        472263  ...................  472281

Reverse primer  1       GGCCAGTTGCAGCTTGTTC  19
Template        472568  ...................  472550
```

>CP015002.1 Pseudomonas aeruginosa strain PA8281 chromosome, complete genome

```
product length = 306
Forward primer  1       TGGGTGATCGCCTTGGTGA  19
Template        472270  ...................  472288

Reverse primer  1       GGCCAGTTGCAGCTTGTTC  19
Template        472575  ...................  472557
```

>CP015001.1 Pseudomonas aeruginosa strain PA1088 chromosome, complete genome

```
product length = 306
Forward primer  1       TGGGTGATCGCCTTGGTGA  19
Template        472270  ...................  472288

Reverse primer  1       GGCCAGTTGCAGCTTGTTC  19
Template        472575  ...................  472557
```

>CP017353.1 Pseudomonas aeruginosa strain FA-HZ1 chromosome, complete genome

```
product length = 306
Forward primer  1        TGGGTGATCGCCTTGGTGA  19
Template        3169880  ...................  3169898

Reverse primer  1        GGCCAGTTGCAGCTTGTTC  19
Template        3170185  ...................  3170167
```

>CP017149.1 Pseudomonas aeruginosa strain ATCC 15692, complete genome

```
product length = 306
Forward primer  1       TGGGTGATCGCCTTGGTGA  19
Template        472748  ...................  472766

Reverse primer  1       GGCCAGTTGCAGCTTGTTC  19
Template        473053  ...................  473035
```

>CP012582.1 Pseudomonas aeruginosa strain PA_D21, complete genome

```
product length = 306
Forward primer  1       TGGGTGATCGCCTTGGTGA  19
Template        473298  ...................  473316

Reverse primer  1       GGCCAGTTGCAGCTTGTTC  19
Template        473603  ...................  473585
```

>CP012579.1 Pseudomonas aeruginosa strain PA_D5, complete genome

```
product length = 306
Forward primer  1       TGGGTGATCGCCTTGGTGA  19
Template        473298  ...................  473316

Reverse primer  1       GGCCAGTTGCAGCTTGTTC  19
Template        473603  ...................  473585
```

>CP017099.1 Pseudomonas aeruginosa strain DN1, complete genome

```
product length = 306
Forward primer  1       TGGGTGATCGCCTTGGTGA  19
Template        478144  ...................  478162

Reverse primer  1       GGCCAGTTGCAGCTTGTTC  19
Template        478449  ...................  478431
```

>CP012584.1 Pseudomonas aeruginosa strain PA_D25, complete genome

```
product length = 306
Forward primer  1       TGGGTGATCGCCTTGGTGA  19
Template        473296  ...................  473314

Reverse primer  1       GGCCAGTTGCAGCTTGTTC  19
Template        473601  ...................  473583
```

>CP012583.1 Pseudomonas aeruginosa strain PA_D22, complete genome

```
product length = 306
Forward primer  1       TGGGTGATCGCCTTGGTGA  19
Template        473298  ...................  473316

Reverse primer  1       GGCCAGTTGCAGCTTGTTC  19
Template        473603  ...................  473585
```

>CP012581.1 Pseudomonas aeruginosa strain PA_D16, complete genome

```
product length = 306
Forward primer  1       TGGGTGATCGCCTTGGTGA  19
Template        473298  ...................  473316

Reverse primer  1       GGCCAGTTGCAGCTTGTTC  19
Template        473603  ...................  473585
```

>CP012580.1 Pseudomonas aeruginosa strain PA_D9, complete genome

```
product length = 306
Forward primer  1       TGGGTGATCGCCTTGGTGA  19
Template        473298  ...................  473316

Reverse primer  1       GGCCAGTTGCAGCTTGTTC  19
Template        473603  ...................  473585
```

>CP012578.1 Pseudomonas aeruginosa strain PA_D2, complete genome

```
product length = 306
Forward primer  1       TGGGTGATCGCCTTGGTGA  19
Template        473298  ...................  473316

Reverse primer  1       GGCCAGTTGCAGCTTGTTC  19
Template        473603  ...................  473585
```

>CP012585.1 Pseudomonas aeruginosa strain PA_D1, complete genome

```
product length = 306
Forward primer  1       TGGGTGATCGCCTTGGTGA  19
Template        473298  ...................  473316

Reverse primer  1       GGCCAGTTGCAGCTTGTTC  19
Template        473603  ...................  473585
```

>LT608330.1 Pseudomonas aeruginosa isolate PA14Or_reads genome assembly, chromosome: PA14OR

```
product length = 306
Forward primer  1       TGGGTGATCGCCTTGGTGA  19
Template        487978  ...................  487996

Reverse primer  1       GGCCAGTTGCAGCTTGTTC  19
Template        488283  ...................  488265
```

>CP011857.1 Pseudomonas aeruginosa strain ATCC 27853, complete genome

```
product length = 306
Forward primer  1       TGGGTGATCGCCTTGGTGA  19
Template        482887  ...................  482905

Reverse primer  1       GGCCAGTTGCAGCTTGTTC  19
Template        483192  ...................  483174
```

>CP016214.1 Pseudomonas aeruginosa strain PA121617, complete genome

```
product length = 306
Forward primer  1        TGGGTGATCGCCTTGGTGA  19
Template        3687400  ...................  3687418

Reverse primer  1        GGCCAGTTGCAGCTTGTTC  19
Template        3687705  ...................  3687687
```

>CP015877.1 Pseudomonas aeruginosa SJTD-1 chromosome, complete genome

```
product length = 306
Forward primer  1       TGGGTGATCGCCTTGGTGA  19
Template        550200  ...................  550182

Reverse primer  1       GGCCAGTTGCAGCTTGTTC  19
Template        549895  ...................  549913
```

>CP015377.1 Pseudomonas aeruginosa strain BAMCPA07-48 chromosome, complete genome

```
product length = 306
Forward primer  1        TGGGTGATCGCCTTGGTGA  19
Template        2557605  ...................  2557623

Reverse primer  1        GGCCAGTTGCAGCTTGTTC  19
Template        2557910  ...................  2557892
```

>CP015117.1 Pseudomonas aeruginosa strain ATCC 27853 chromosome, complete genome

```
product length = 306
Forward primer  1        TGGGTGATCGCCTTGGTGA  19
Template        4074435  ...................  4074453

Reverse primer  1        GGCCAGTTGCAGCTTGTTC  19
Template        4074740  ...................  4074722
```

>CP014948.1 Pseudomonas aeruginosa strain N17-1, complete genome

```
product length = 306
Forward primer  1       TGGGTGATCGCCTTGGTGA  19
Template        466627  ...................  466645

Reverse primer  1       GGCCAGTTGCAGCTTGTTC  19
Template        466932  ...................  466914
```

>OX638701.1 Pseudomonas aeruginosa strain 4782MK genome assembly, chromosome: 4782

```
product length = 306
Forward primer  1        TGGGTGATCGCCTTGGTGA  19
Template        1748733  ...................  1748751

Reverse primer  1        GGCCAGTTGCAGCTTGTTC  19
Template        1749038  ...................  1749020
```

>OX638610.1 Pseudomonas aeruginosa strain 3541 genome assembly, chromosome: 3541

```
product length = 306
Forward primer  1       TGGGTGATCGCCTTGGTGA  19
Template        230691  ...................  230673

Reverse primer  1       GGCCAGTTGCAGCTTGTTC  19
Template        230386  ...................  230404
```

>OX638564.1 Pseudomonas aeruginosa strain 3796A genome assembly, chromosome: 3796A

```
product length = 306
Forward primer  1       TGGGTGATCGCCTTGGTGA  19
Template        478681  ...................  478699

Reverse primer  1       GGCCAGTTGCAGCTTGTTC  19
Template        478986  ...................  478968
```

>CP008873.1 Pseudomonas aeruginosa strain F9670 chromosome, complete genome

```
product length = 306
Forward primer  1       TGGGTGATCGCCTTGGTGA  19
Template        897907  ...................  897889

Reverse primer  1       GGCCAGTTGCAGCTTGTTC  19
Template        897602  ...................  897620
```

>CP013993.1 Pseudomonas aeruginosa DHS01 chromosome, complete genome

```
product length = 306
Forward primer  1       TGGGTGATCGCCTTGGTGA  19
Template        466029  ...................  466047

Reverse primer  1       GGCCAGTTGCAGCTTGTTC  19
Template        466334  ...................  466316
```

>CP013989.1 Pseudomonas aeruginosa strain USDA-ARS-USMARC-41639 chromosome, complete genome

```
product length = 306
Forward primer  1       TGGGTGATCGCCTTGGTGA  19
Template        495906  ...................  495924

Reverse primer  1       GGCCAGTTGCAGCTTGTTC  19
Template        496211  ...................  496193
```

>CP008868.1 Pseudomonas aeruginosa strain T63266 chromosome, complete genome

```
product length = 306
Forward primer  1        TGGGTGATCGCCTTGGTGA  19
Template        2130646  ...................  2130664

Reverse primer  1        GGCCAGTTGCAGCTTGTTC  19
Template        2130951  ...................  2130933
```

>CP008867.1 Pseudomonas aeruginosa strain T52373 chromosome, complete genome

```
product length = 306
Forward primer  1        TGGGTGATCGCCTTGGTGA  19
Template        2962204  ...................  2962222

Reverse primer  1        GGCCAGTTGCAGCTTGTTC  19
Template        2962509  ...................  2962491
```

>CP008863.1 Pseudomonas aeruginosa strain M37351 chromosome, complete genome

```
product length = 306
Forward primer  1        TGGGTGATCGCCTTGGTGA  19
Template        4078047  ...................  4078065

Reverse primer  1        GGCCAGTTGCAGCTTGTTC  19
```

```
Template        4078352  ...................  4078334
```

>CP008861.1 Pseudomonas aeruginosa strain H47921 chromosome, complete genome

```
product length = 306
Forward primer  1        TGGGTGATCGCCTTGGTGA  19
Template        3255527  ...................  3255509

Reverse primer  1        GGCCAGTTGCAGCTTGTTC  19
Template        3255222  ...................  3255240
```

>CP008857.1 Pseudomonas aeruginosa strain F30658 chromosome, complete genome

```
product length = 306
Forward primer  1        TGGGTGATCGCCTTGGTGA  19
Template        5151493  ...................  5151511

Reverse primer  1        GGCCAGTTGCAGCTTGTTC  19
Template        5151798  ...................  5151780
```

>CP012901.1 Pseudomonas aeruginosa strain N15-01092 chromosome, complete genome

```
product length = 306
Forward primer  1        TGGGTGATCGCCTTGGTGA  19
Template        6431490  ...................  6431472

Reverse primer  1        GGCCAGTTGCAGCTTGTTC  19
Template        6431185  ...................  6431203
```

>CP013696.1 Pseudomonas aeruginosa strain 12-4-4(59) chromosome, complete genome

```
product length = 306
Forward primer  1        TGGGTGATCGCCTTGGTGA  19
Template        1979635  ...................  1979653

Reverse primer  1        GGCCAGTTGCAGCTTGTTC  19
Template        1979940  ...................  1979922
```

>CP124672.1 Pseudomonas aeruginosa strain 2022CK-00451 chromosome, complete genome

```
product length = 306
Forward primer  1        TGGGTGATCGCCTTGGTGA  19
Template        2156113  ...................  2156131

Reverse primer  1        GGCCAGTTGCAGCTTGTTC  19
Template        2156418  ...................  2156400
```

>CP125367.1 Pseudomonas aeruginosa strain ZY1710 chromosome, complete genome

```
product length = 306
Forward primer  1       TGGGTGATCGCCTTGGTGA  19
Template        504959  ...................  504977
```

```
Reverse primer  1       GGCCAGTTGCAGCTTGTTC  19
Template        505264  ...................  505246
```

>CP125365.1 Pseudomonas aeruginosa strain ZY36 chromosome, complete genome

```
product length = 306
Forward primer  1       TGGGTGATCGCCTTGGTGA  19
Template        504960  ...................  504978

Reverse primer  1       GGCCAGTTGCAGCTTGTTC  19
Template        505265  ...................  505247
```

>CP125363.1 Pseudomonas aeruginosa strain ZY156 chromosome, complete genome

```
product length = 306
Forward primer  1       TGGGTGATCGCCTTGGTGA  19
Template        504960  ...................  504978

Reverse primer  1       GGCCAGTTGCAGCTTGTTC  19
Template        505265  ...................  505247
```

>CP125361.1 Pseudomonas aeruginosa strain ZY94 chromosome, complete genome

```
product length = 306
Forward primer  1       TGGGTGATCGCCTTGGTGA  19
Template        504954  ...................  504972

Reverse primer  1       GGCCAGTTGCAGCTTGTTC  19
Template        505259  ...................  505241
```

>CP125288.1 Pseudomonas aeruginosa strain SF416 chromosome, complete genome

```
product length = 306
Forward primer  1       TGGGTGATCGCCTTGGTGA  19
Template        491101  ...................  491119

Reverse primer  1       GGCCAGTTGCAGCTTGTTC  19
Template        491406  ...................  491388
```

>CP013144.1 Pseudomonas aeruginosa strain Cu1510 chromosome, complete genome

```
product length = 306
Forward primer  1        TGGGTGATCGCCTTGGTGA  19
Template        4306771  ...................  4306789

Reverse primer  1        GGCCAGTTGCAGCTTGTTC  19
Template        4307076  ...................  4307058
```

>AP017302.1 Pseudomonas aeruginosa DNA, complete genome, strain: IOMTU 133

```
product length = 306
Forward primer  1       TGGGTGATCGCCTTGGTGA  19
Template        608388  ...................  608406
```

```
Reverse primer  1        GGCCAGTTGCAGCTTGTTC  19
Template        608693   ...................  608675
```

>CP013245.1 Pseudomonas aeruginosa strain VA-134 chromosome, complete genome

```
product length = 306
Forward primer  1         TGGGTGATCGCCTTGGTGA  19
Template        1746234   ...................  1746216

Reverse primer  1         GGCCAGTTGCAGCTTGTTC  19
Template        1745929   ...................  1745947
```

>LN870292.1 Pseudomonas aeruginosa DK1 genome assembly Pseudomonas aeruginosa DK1 substr. NH57388A, chromosome : I

```
product length = 306
Forward primer  1        TGGGTGATCGCCTTGGTGA  19
Template        479377   ...................  479395

Reverse primer  1        GGCCAGTTGCAGCTTGTTC  19
Template        479682   ...................  479664
```

>CP012679.1 Pseudomonas aeruginosa strain PA1RG chromosome, complete genome

```
product length = 306
Forward primer  1        TGGGTGATCGCCTTGGTGA  19
Template        470684   ...................  470702

Reverse primer  1        GGCCAGTTGCAGCTTGTTC  19
Template        470989   ...................  470971
```

>CP004054.2 Pseudomonas aeruginosa PA1, complete genome

```
product length = 306
Forward primer  1        TGGGTGATCGCCTTGGTGA  19
Template        470684   ...................  470702

Reverse primer  1        GGCCAGTTGCAGCTTGTTC  19
Template        470989   ...................  470971
```

>LN871187.1 Pseudomonas aeruginosa genome assembly PAO1OR, chromosome : I

```
product length = 306
Forward primer  1        TGGGTGATCGCCTTGGTGA  19
Template        473231   ...................  473249

Reverse primer  1        GGCCAGTTGCAGCTTGTTC  19
Template        473536   ...................  473518
```

>AP014839.2 Pseudomonas aeruginosa DNA, complete genome, strain: 8380

```
product length = 306
```

```
Forward primer  1       TGGGTGATCGCCTTGGTGA  19
Template        468537  ...................  468555

Reverse primer  1       GGCCAGTTGCAGCTTGTTC  19
Template        468842  ...................  468824
```

>CP012066.1 Pseudomonas aeruginosa strain F9676, complete genome

```
product length = 306
Forward primer  1        TGGGTGATCGCCTTGGTGA  19
Template        5464370  ...................  5464352

Reverse primer  1        GGCCAGTTGCAGCTTGTTC  19
Template        5464065  ...................  5464083
```

>CP012001.1 Pseudomonas aeruginosa DSM 50071, complete genome

```
product length = 306
Forward primer  1       TGGGTGATCGCCTTGGTGA  19
Template        464892  ...................  464910

Reverse primer  1       GGCCAGTTGCAGCTTGTTC  19
Template        465197  ...................  465179
```

>CP011369.1 Pseudomonas aeruginosa strain S04 90 chromosome

```
product length = 306
Forward primer  1       TGGGTGATCGCCTTGGTGA  19
Template        479121  ...................  479139

Reverse primer  1       GGCCAGTTGCAGCTTGTTC  19
Template        479426  ...................  479408
```

>CP011317.1 Pseudomonas aeruginosa strain Carb01 63, complete genome

```
product length = 306
Forward primer  1       TGGGTGATCGCCTTGGTGA  19
Template        556412  ...................  556430

Reverse primer  1       GGCCAGTTGCAGCTTGTTC  19
Template        556717  ...................  556699
```

>LN831024.1 Pseudomonas aeruginosa genome assembly NCTC10332, chromosome : 1

```
product length = 306
Forward primer  1       TGGGTGATCGCCTTGGTGA  19
Template        464405  ...................  464423

Reverse primer  1       GGCCAGTTGCAGCTTGTTC  19
Template        464710  ...................  464692
```

>AP014651.1 Pseudomonas aeruginosa DNA, complete genome, strain: NCGM257

```
product length = 306
Forward primer  1       TGGGTGATCGCCTTGGTGA  19
Template        560089  ...................  560107

Reverse primer  1       GGCCAGTTGCAGCTTGTTC  19
Template        560394  ...................  560376
```

>CP010555.1 Pseudomonas aeruginosa strain FRD1, complete genome

```
product length = 306
Forward primer  1        TGGGTGATCGCCTTGGTGA  19
Template        4282877  ...................  4282895

Reverse primer  1        GGCCAGTTGCAGCTTGTTC  19
Template        4283182  ...................  4283164
```

>CP007399.1 Pseudomonas aeruginosa strain F22031, complete genome

```
product length = 306
Forward primer  1        TGGGTGATCGCCTTGGTGA  19
Template        3017988  ...................  3018006

Reverse primer  1        GGCCAGTTGCAGCTTGTTC  19
Template        3018293  ...................  3018275
```

>AP014646.1 Pseudomonas aeruginosa DNA, complete genome, strain: NCGM 1984

```
product length = 306
Forward primer  1       TGGGTGATCGCCTTGGTGA  19
Template        474161  ...................  474179

Reverse primer  1       GGCCAGTTGCAGCTTGTTC  19
Template        474466  ...................  474448
```

>HG974234.1 Pseudomonas aeruginosa strain PSE305, genome

```
product length = 306
Forward primer  1        TGGGTGATCGCCTTGGTGA  19
Template        6380822  ...................  6380840

Reverse primer  1        GGCCAGTTGCAGCTTGTTC  19
Template        6381127  ...................  6381109
```

>CP089067.2 Pseudomonas aeruginosa strain UNC_PaerCF19 chromosome, complete genome

```
product length = 306
Forward primer  1       TGGGTGATCGCCTTGGTGA  19
Template        495399  ...................  495381

Reverse primer  1       GGCCAGTTGCAGCTTGTTC  19
Template        495094  ...................  495112
```

>CP089068.2 Pseudomonas aeruginosa strain UNC_PaerCF13 chromosome, complete genome

```
product length = 306
Forward primer  1        TGGGTGATCGCCTTGGTGA  19
Template        1989766  ...................  1989784

Reverse primer  1        GGCCAGTTGCAGCTTGTTC  19
Template        1990071  ...................  1990053
```

>CP089065.2 Pseudomonas aeruginosa strain UNC_PaerCF34 chromosome, complete genome

```
product length = 306
Forward primer  1       TGGGTGATCGCCTTGGTGA  19
Template        253419  ...................  253401

Reverse primer  1       GGCCAGTTGCAGCTTGTTC  19
Template        253114  ...................  253132
```

>CP069331.1 Pseudomonas aeruginosa strain R09 chromosome, complete genome

```
product length = 306
Forward primer  1       TGGGTGATCGCCTTGGTGA  19
Template        453831  ...................  453849

Reverse primer  1       GGCCAGTTGCAGCTTGTTC  19
Template        454136  ...................  454118
```

>CP069337.1 Pseudomonas aeruginosa strain E04 chromosome, complete genome

```
product length = 306
Forward primer  1       TGGGTGATCGCCTTGGTGA  19
Template        504604  ...................  504622

Reverse primer  1       GGCCAGTTGCAGCTTGTTC  19
Template        504909  ...................  504891
```

>CP069336.1 Pseudomonas aeruginosa strain E01 chromosome, complete genome

```
product length = 306
Forward primer  1       TGGGTGATCGCCTTGGTGA  19
Template        453879  ...................  453897

Reverse primer  1       GGCCAGTTGCAGCTTGTTC  19
Template        454184  ...................  454166
```

>CP069335.1 Pseudomonas aeruginosa strain E02 chromosome, complete genome

```
product length = 306
Forward primer  1       TGGGTGATCGCCTTGGTGA  19
Template        453880  ...................  453898

Reverse primer  1       GGCCAGTTGCAGCTTGTTC  19
Template        454185  ...................  454167
```

>CP069334.1 Pseudomonas aeruginosa strain E03 chromosome, complete genome

```
product length = 306
Forward primer  1       TGGGTGATCGCCTTGGTGA  19
Template        453880  ...................  453898

Reverse primer  1       GGCCAGTTGCAGCTTGTTC  19
Template        454185  ...................  454167
```

>CP069333.1 Pseudomonas aeruginosa strain R01 chromosome, complete genome

```
product length = 306
Forward primer  1       TGGGTGATCGCCTTGGTGA  19
Template        453876  ...................  453894

Reverse primer  1       GGCCAGTTGCAGCTTGTTC  19
Template        454181  ...................  454163
```

>CP069332.1 Pseudomonas aeruginosa strain R08 chromosome, complete genome

```
product length = 306
Forward primer  1       TGGGTGATCGCCTTGGTGA  19
Template        453831  ...................  453849

Reverse primer  1       GGCCAGTTGCAGCTTGTTC  19
Template        454136  ...................  454118
```

>CP069330.1 Pseudomonas aeruginosa strain R07 chromosome, complete genome

```
product length = 306
Forward primer  1       TGGGTGATCGCCTTGGTGA  19
Template        454537  ...................  454555

Reverse primer  1       GGCCAGTTGCAGCTTGTTC  19
Template        454842  ...................  454824
```

>CP069329.1 Pseudomonas aeruginosa strain R10 chromosome, complete genome

```
product length = 306
Forward primer  1       TGGGTGATCGCCTTGGTGA  19
Template        453831  ...................  453849

Reverse primer  1       GGCCAGTTGCAGCTTGTTC  19
Template        454136  ...................  454118
```

>CP069328.1 Pseudomonas aeruginosa strain R04 chromosome, complete genome

```
product length = 306
Forward primer  1       TGGGTGATCGCCTTGGTGA  19
Template        453858  ...................  453876

Reverse primer  1       GGCCAGTTGCAGCTTGTTC  19
Template        454163  ...................  454145
```

>CP069327.1 Pseudomonas aeruginosa strain R03 chromosome, complete genome

```
product length = 306
Forward primer  1       TGGGTGATCGCCTTGGTGA  19
Template        453849  ...................  453867

Reverse primer  1       GGCCAGTTGCAGCTTGTTC  19
Template        454154  ...................  454136
```

>CP069326.1 Pseudomonas aeruginosa strain R11 chromosome, complete genome

```
product length = 306
Forward primer  1       TGGGTGATCGCCTTGGTGA  19
Template        453840  ...................  453858

Reverse primer  1       GGCCAGTTGCAGCTTGTTC  19
Template        454145  ...................  454127
```

>CP069325.1 Pseudomonas aeruginosa strain R02 chromosome, complete genome

```
product length = 306
Forward primer  1       TGGGTGATCGCCTTGGTGA  19
Template        453873  ...................  453891

Reverse primer  1       GGCCAGTTGCAGCTTGTTC  19
Template        454178  ...................  454160
```

>CP069324.1 Pseudomonas aeruginosa strain R05 chromosome, complete genome

```
product length = 306
Forward primer  1       TGGGTGATCGCCTTGGTGA  19
Template        453852  ...................  453870

Reverse primer  1       GGCCAGTTGCAGCTTGTTC  19
Template        454157  ...................  454139
```

>CP069323.1 Pseudomonas aeruginosa strain R06 chromosome, complete genome

```
product length = 306
Forward primer  1       TGGGTGATCGCCTTGGTGA  19
Template        453852  ...................  453870

Reverse primer  1       GGCCAGTTGCAGCTTGTTC  19
Template        454157  ...................  454139
```

>CP068678.1 Pseudomonas aeruginosa strain NCCP15783 chromosome, complete genome

```
product length = 306
Forward primer  1        TGGGTGATCGCCTTGGTGA  19
Template        1980335  ...................  1980353

Reverse primer  1        GGCCAGTTGCAGCTTGTTC  19
Template        1980640  ...................  1980622
```

>CP008739.2 Pseudomonas aeruginosa VRFPA04, complete genome

```
product length = 306
Forward primer  1        TGGGTGATCGCCTTGGTGA  19
Template        6230235  ...................  6230217

Reverse primer  1        GGCCAGTTGCAGCTTGTTC  19
Template        6229930  ...................  6229948
```

>AP014622.1 Pseudomonas aeruginosa DNA, complete genome, strain: NCGM 1900

```
product length = 306
Forward primer  1       TGGGTGATCGCCTTGGTGA  19
Template        474159  ...................  474177

Reverse primer  1       GGCCAGTTGCAGCTTGTTC  19
Template        474464  ...................  474446
```

>CP008749.1 Pseudomonas aeruginosa PAO1H2O genome

```
product length = 306
Forward primer  1       TGGGTGATCGCCTTGGTGA  19
Template        473227  ...................  473245

Reverse primer  1       GGCCAGTTGCAGCTTGTTC  19
Template        473532  ...................  473514
```

>CP007224.1 Pseudomonas aeruginosa PA96 genome

```
product length = 306
Forward primer  1       TGGGTGATCGCCTTGGTGA  19
Template        467980  ...................  467998

Reverse primer  1       GGCCAGTTGCAGCTTGTTC  19
Template        468285  ...................  468267
```

>CP006985.1 Pseudomonas aeruginosa LESlike4 sequence

```
product length = 306
Forward primer  1       TGGGTGATCGCCTTGGTGA  19
Template        466517  ...................  466535

Reverse primer  1       GGCCAGTTGCAGCTTGTTC  19
Template        466822  ...................  466804
```

>CP006984.1 Pseudomonas aeruginosa LESlike1 chromosome

```
product length = 306
Forward primer  1       TGGGTGATCGCCTTGGTGA  19
Template        467073  ...................  467091

Reverse primer  1       GGCCAGTTGCAGCTTGTTC  19
Template        467378  ...................  467360
```

>CP006983.1 Pseudomonas aeruginosa LESB65 sequence

```
product length = 306
Forward primer  1       TGGGTGATCGCCTTGGTGA  19
Template        466580  ...................  466598

Reverse primer  1       GGCCAGTTGCAGCTTGTTC  19
Template        466885  ...................  466867
```

>CP006982.1 Pseudomonas aeruginosa LES400 sequence

```
product length = 306
Forward primer  1       TGGGTGATCGCCTTGGTGA  19
Template        466742  ...................  466760

Reverse primer  1       GGCCAGTTGCAGCTTGTTC  19
Template        467047  ...................  467029
```

>CP006981.1 Pseudomonas aeruginosa LESlike7 sequence

```
product length = 306
Forward primer  1       TGGGTGATCGCCTTGGTGA  19
Template        466950  ...................  466968

Reverse primer  1       GGCCAGTTGCAGCTTGTTC  19
Template        467255  ...................  467237
```

>CP006980.1 Pseudomonas aeruginosa LESlike5 sequence

```
product length = 306
Forward primer  1       TGGGTGATCGCCTTGGTGA  19
Template        467036  ...................  467054

Reverse primer  1       GGCCAGTTGCAGCTTGTTC  19
Template        467341  ...................  467323
```

>CP007147.1 Pseudomonas aeruginosa YL84, complete genome

```
product length = 306
Forward primer  1       TGGGTGATCGCCTTGGTGA  19
Template        358116  ...................  358098

Reverse primer  1       GGCCAGTTGCAGCTTGTTC  19
Template        357811  ...................  357829
```

>HG530068.1 Pseudomonas aeruginosa PA38182, complete genome

```
product length = 306
Forward primer  1       TGGGTGATCGCCTTGGTGA  19
Template        500196  ...................  500214

Reverse primer  1       GGCCAGTTGCAGCTTGTTC  19
Template        500501  ...................  500483
```

>CP006931.1 Pseudomonas aeruginosa SCV20265, complete genome

```
product length = 306
Forward primer  1       TGGGTGATCGCCTTGGTGA  19
Template        473076  ...................  473094

Reverse primer  1       GGCCAGTTGCAGCTTGTTC  19
Template        473381  ...................  473363
```

>CP006853.1 Pseudomonas aeruginosa MTB-1, complete genome

```
product length = 306
Forward primer  1       TGGGTGATCGCCTTGGTGA  19
Template        473962  ...................  473980

Reverse primer  1       GGCCAGTTGCAGCTTGTTC  19
Template        474267  ...................  474249
```

>CP004055.1 Pseudomonas aeruginosa PA1R, complete genome

```
product length = 306
Forward primer  1        TGGGTGATCGCCTTGGTGA  19
Template        4389728  ...................  4389746

Reverse primer  1        GGCCAGTTGCAGCTTGTTC  19
Template        4390033  ...................  4390015
```

>CP081345.1 Pseudomonas aeruginosa strain F291007 chromosome, complete genome

```
product length = 306
Forward primer  1       TGGGTGATCGCCTTGGTGA  19
Template        510243  ...................  510261

Reverse primer  1       GGCCAGTTGCAGCTTGTTC  19
Template        510548  ...................  510530
```

>CP081346.1 Pseudomonas aeruginosa strain SE5419 chromosome, complete genome

```
product length = 306
Forward primer  1       TGGGTGATCGCCTTGGTGA  19
Template        481769  ...................  481787

Reverse primer  1       GGCCAGTTGCAGCTTGTTC  19
Template        482074  ...................  482056
```

>CP081287.1 Pseudomonas aeruginosa strain F092021 chromosome, complete genome

```
product length = 306
Forward primer  1       TGGGTGATCGCCTTGGTGA  19
Template        513697  ...................  513715

Reverse primer  1       GGCCAGTTGCAGCTTGTTC  19
Template        514002  ...................  513984
```

>CP081202.1 Pseudomonas aeruginosa strain P9W chromosome, complete genome

```
product length = 306
Forward primer  1        TGGGTGATCGCCTTGGTGA  19
Template        5159794  ...................  5159776

Reverse primer  1        GGCCAGTTGCAGCTTGTTC  19
Template        5159489  ...................  5159507
```

>CP006832.1 Pseudomonas aeruginosa PAO1-VE13 genome

```
product length = 306
Forward primer  1       TGGGTGATCGCCTTGGTGA  19
Template        473227  ...................  473245

Reverse primer  1       GGCCAGTTGCAGCTTGTTC  19
Template        473532  ...................  473514
```

>CP006831.1 Pseudomonas aeruginosa PAO1-VE2 genome

```
product length = 306
Forward primer  1       TGGGTGATCGCCTTGGTGA  19
Template        473227  ...................  473245

Reverse primer  1       GGCCAGTTGCAGCTTGTTC  19
Template        473532  ...................  473514
```

>CP006705.1 Pseudomonas aeruginosa PAO581 genome

```
product length = 306
Forward primer  1       TGGGTGATCGCCTTGGTGA  19
Template        473103  ...................  473121

Reverse primer  1       GGCCAGTTGCAGCTTGTTC  19
Template        473408  ...................  473390
```

>CP006728.1 Pseudomonas aeruginosa c7447m genome

```
product length = 306
Forward primer  1       TGGGTGATCGCCTTGGTGA  19
Template        473204  ...................  473222

Reverse primer  1       GGCCAGTTGCAGCTTGTTC  19
Template        473509  ...................  473491
```

>CP006245.1 Pseudomonas aeruginosa RP73, complete genome

```
product length = 306
Forward primer  1       TGGGTGATCGCCTTGGTGA  19
Template        467845  ...................  467863

Reverse primer  1       GGCCAGTTGCAGCTTGTTC  19
Template        468150  ...................  468132
```

>CP074424.1 Pseudomonas aeruginosa strain 88A chromosome

```
product length = 306
Forward primer  1        TGGGTGATCGCCTTGGTGA  19
Template        4371607  ...................  4371625

Reverse primer  1        GGCCAGTTGCAGCTTGTTC  19
Template        4371912  ...................  4371894
```

>CP061780.1 Pseudomonas aeruginosa strain ZBX-P11 chromosome, complete genome

```
product length = 306
Forward primer  1        TGGGTGATCGCCTTGGTGA  19
Template        3078798  ...................  3078816

Reverse primer  1        GGCCAGTTGCAGCTTGTTC  19
Template        3079103  ...................  3079085
```

>CP061779.1 Pseudomonas aeruginosa strain ZBX-P12 chromosome, complete genome

```
product length = 306
Forward primer  1        TGGGTGATCGCCTTGGTGA  19
Template        6509902  ...................  6509920

Reverse primer  1        GGCCAGTTGCAGCTTGTTC  19
Template        6510207  ...................  6510189
```

>CP061778.1 Pseudomonas aeruginosa strain ZBX-P13 chromosome, complete genome

```
product length = 306
Forward primer  1        TGGGTGATCGCCTTGGTGA  19
Template        2220156  ...................  2220174

Reverse primer  1        GGCCAGTTGCAGCTTGTTC  19
Template        2220461  ...................  2220443
```

>CP061777.1 Pseudomonas aeruginosa strain ZBX-P23 chromosome, complete genome

```
product length = 306
Forward primer  1       TGGGTGATCGCCTTGGTGA  19
Template        184232  ...................  184250

Reverse primer  1       GGCCAGTTGCAGCTTGTTC  19
Template        184537  ...................  184519
```

>CP004061.1 Pseudomonas aeruginosa B136-33, complete genome

```
product length = 306
Forward primer  1       TGGGTGATCGCCTTGGTGA  19
Template        465090  ...................  465108

Reverse primer  1       GGCCAGTTGCAGCTTGTTC  19
Template        465395  ...................  465377
```

>CP070471.1 Pseudomonas aeruginosa strain B17932 chromosome, complete genome

```
product length = 306
Forward primer  1        TGGGTGATCGCCTTGGTGA  19
Template        6334160  ...................  6334142

Reverse primer  1        GGCCAGTTGCAGCTTGTTC  19
Template        6333855  ...................  6333873
```

>CP070467.1 Pseudomonas aeruginosa strain B17416 chromosome, complete genome

```
product length = 306
Forward primer  1        TGGGTGATCGCCTTGGTGA  19
Template        6572800  ...................  6572782

Reverse primer  1        GGCCAGTTGCAGCTTGTTC  19
Template        6572495  ...................  6572513
```

>CP070355.1 Pseudomonas aeruginosa strain PDNC003 chromosome

```
product length = 306
Forward primer  1        TGGGTGATCGCCTTGGTGA  19
Template        5621221  ...................  5621239

Reverse primer  1        GGCCAGTTGCAGCTTGTTC  19
Template        5621526  ...................  5621508
```

>CP060392.1 Pseudomonas aeruginosa strain 1903031130 chromosome, complete genome

```
product length = 306
Forward primer  1       TGGGTGATCGCCTTGGTGA  19
Template        509980  ...................  509998

Reverse primer  1       GGCCAGTTGCAGCTTGTTC  19
Template        510285  ...................  510267
```

>CP054845.1 Pseudomonas aeruginosa strain SE5429 chromosome, complete genome

```
product length = 306
Forward primer  1       TGGGTGATCGCCTTGGTGA  19
Template        473554  ...................  473572

Reverse primer  1       GGCCAGTTGCAGCTTGTTC  19
Template        473859  ...................  473841
```

>CP054844.1 Pseudomonas aeruginosa strain SE5357 chromosome, complete genome

```
product length = 306
Forward primer  1       TGGGTGATCGCCTTGGTGA  19
Template        485660  ...................  485678

Reverse primer  1       GGCCAGTTGCAGCTTGTTC  19
Template        485965  ...................  485947
```

>CP054843.1 Pseudomonas aeruginosa strain SE5352 chromosome, complete genome

```
product length = 306
Forward primer  1       TGGGTGATCGCCTTGGTGA  19
Template        509965  ...................  509983

Reverse primer  1       GGCCAGTTGCAGCTTGTTC  19
Template        510270  ...................  510252
```

>CP054581.1 Pseudomonas aeruginosa strain YTSEY8 chromosome, complete genome

```
product length = 306
Forward primer  1       TGGGTGATCGCCTTGGTGA  19
Template        483318  ...................  483336

Reverse primer  1       GGCCAGTTGCAGCTTGTTC  19
Template        483623  ...................  483605
```

>CP068239.1 Pseudomonas aeruginosa strain PA19-3047 chromosome, complete genome

```
product length = 306
Forward primer  1        TGGGTGATCGCCTTGGTGA  19
Template        6531118  ...................  6531100

Reverse primer  1        GGCCAGTTGCAGCTTGTTC  19
Template        6530813  ...................  6530831
```

>CP061699.1 Pseudomonas aeruginosa strain LYSZa7 chromosome, complete genome

```
product length = 306
Forward primer  1        TGGGTGATCGCCTTGGTGA  19
Template        6272843  ...................  6272825

Reverse primer  1        GGCCAGTTGCAGCTTGTTC  19
Template        6272538  ...................  6272556
```

>CP063237.1 Pseudomonas aeruginosa strain mPA08-31 chromosome

```
product length = 306
Forward primer  1       TGGGTGATCGCCTTGGTGA  19
Template        415242  ...................  415260

Reverse primer  1       GGCCAGTTGCAGCTTGTTC  19
Template        415547  ...................  415529
```

>CP063047.1 Pseudomonas aeruginosa strain KC-Tt-1 chromosome, complete genome

```
product length = 306
Forward primer  1        TGGGTGATCGCCTTGGTGA  19
Template        5957968  ...................  5957986

Reverse primer  1        GGCCAGTTGCAGCTTGTTC  19
Template        5958273  ...................  5958255
```

>CP062219.1 Pseudomonas aeruginosa strain JT86 chromosome, complete genome

```
product length = 306
Forward primer  1        TGGGTGATCGCCTTGGTGA  19
Template        5246043  ...................  5246061

Reverse primer  1        GGCCAGTTGCAGCTTGTTC  19
Template        5246348  ...................  5246330
```

>CP061850.1 Pseudomonas aeruginosa strain R31 chromosome, complete genome

```
product length = 306
Forward primer  1        TGGGTGATCGCCTTGGTGA  19
Template        6780051  ...................  6780069

Reverse primer  1        GGCCAGTTGCAGCTTGTTC  19
Template        6780356  ...................  6780338
```

>CP058257.1 Pseudomonas aeruginosa strain PA179 chromosome

```
product length = 306
Forward primer  1       TGGGTGATCGCCTTGGTGA  19
Template        578202  ...................  578220

Reverse primer  1       GGCCAGTTGCAGCTTGTTC  19
Template        578507  ...................  578489
```

>CP059852.1 Pseudomonas aeruginosa strain ZM03 chromosome, complete genome

```
product length = 306
Forward primer  1        TGGGTGATCGCCTTGGTGA  19
Template        3777019  ...................  3777001

Reverse primer  1        GGCCAGTTGCAGCTTGTTC  19
Template        3776714  ...................  3776732
```

>AP012280.1 Pseudomonas aeruginosa NCGM2.S1 DNA, complete genome

```
product length = 306
Forward primer  1        TGGGTGATCGCCTTGGTGA  19
Template        6157425  ...................  6157407

Reverse primer  1        GGCCAGTTGCAGCTTGTTC  19
Template        6157120  ...................  6157138
```

>CP002496.1 Pseudomonas aeruginosa M18, complete genome

```
product length = 306
Forward primer  1       TGGGTGATCGCCTTGGTGA  19
Template        466751  ...................  466769

Reverse primer  1       GGCCAGTTGCAGCTTGTTC  19
Template        467056  ...................  467038
```

>CP002727.1 Pseudomonas fulva 12-X, complete genome

```
product length = 306
Forward primer  1       TGGGTGATCGCCTTGGTGA  19
Template        972437  ...................  972455

Reverse primer  1       GGCCAGTTGCAGCTTGTTC  19
Template        972742  ...................  972724
```

>FM209186.1 Pseudomonas aeruginosa LESB58 complete genome sequence

```
product length = 306
Forward primer  1       TGGGTGATCGCCTTGGTGA  19
Template        466968  ...................  466986

Reverse primer  1       GGCCAGTTGCAGCTTGTTC  19
Template        467273  ...................  467255
```

>CP000438.1 Pseudomonas aeruginosa UCBPP-PA14, complete genome

```
product length = 306
Forward primer  1       TGGGTGATCGCCTTGGTGA  19
Template        487978  ...................  487996

Reverse primer  1       GGCCAGTTGCAGCTTGTTC  19
Template        488283  ...................  488265
```

>AE004091.2 Pseudomonas aeruginosa PAO1, complete genome

```
product length = 306
Forward primer  1       TGGGTGATCGCCTTGGTGA  19
Template        473227  ...................  473245

Reverse primer  1       GGCCAGTTGCAGCTTGTTC  19
Template        473532  ...................  473514
```

>AF092566.1 Pseudomonas aeruginosa multidrug resistance protein MexA precursor (mexA) and multidrug resistance protein MexB (mexB) genes, partial cds

```
product length = 306
Forward primer  1    TGGGTGATCGCCTTGGTGA  19
Template        661  ...................  679

Reverse primer  1    GGCCAGTTGCAGCTTGTTC  19
Template        966  ...................  948
```

>L11616.1 Pseudomonas aeruginosa mexA and mexB genes, complete cds and outer membrane protein (oprM) gene, partial cds

```
product length = 306
Forward primer  1     TGGGTGATCGCCTTGGTGA  19
Template        1606  ...................  1624

Reverse primer  1     GGCCAGTTGCAGCTTGTTC  19
```

```
Template        1911  ...................  1893
```

>CP104579.1 Pseudomonas oleovorans strain GD04132 chromosome, complete genome

```
product length = 306
Forward primer  1        TGGGTGATCGCCTTGGTGA  19
Template        3738489  ...................  3738507

Reverse primer  1        GGCCAGTTGCAGCTTGTTC  19
Template        3738794  C..................  3738776
```

>CP104582.1 Pseudomonas sp. GD03919 chromosome, complete genome

```
product length = 306
Forward primer  1       TGGGTGATCGCCTTGGTGA  19
Template        586739  ...................  586721

Reverse primer  1       GGCCAGTTGCAGCTTGTTC  19
Template        586434  C..................  586452
```

>CP104583.1 Pseudomonas sp. GD03721 chromosome, complete genome

```
product length = 306
Forward primer  1       TGGGTGATCGCCTTGGTGA  19
Template        586738  ...................  586720

Reverse primer  1       GGCCAGTTGCAGCTTGTTC  19
Template        586433  C..................  586451
```

>CP107275.1 Pseudomonas aeruginosa strain PALA22 chromosome, complete genome

```
product length = 306
Forward primer  1       TGGGTGATCGCCTTGGTGA  19
Template        469344  .............C.....  469362

Reverse primer  1       GGCCAGTTGCAGCTTGTTC  19
Template        469649  ...................  469631
```

>CP104867.1 Pseudomonas aeruginosa strain PALA6 chromosome, complete genome

```
product length = 306
Forward primer  1       TGGGTGATCGCCTTGGTGA  19
Template        472074  ...................  472092

Reverse primer  1       GGCCAGTTGCAGCTTGTTC  19
Template        472379  ............G......  472361
```

>CP104865.1 Pseudomonas aeruginosa strain PALA2 chromosome, complete genome

```
product length = 306
Forward primer  1       TGGGTGATCGCCTTGGTGA  19
Template        474529  ...................  474547
```

```
Reverse primer  1       GGCCAGTTGCAGCTTGTTC  19
Template        474834  ............G......  474816
```

>CP111110.1 Pseudomonas chengduensis strain BC1815 chromosome, complete genome

```
product length = 306
Forward primer  1        TGGGTGATCGCCTTGGTGA  19
Template        4588110  ...................  4588128

Reverse primer  1        GGCCAGTTGCAGCTTGTTC  19
Template        4588415  C..................  4588397
```

>CP109657.1 Pseudomonas aeruginosa strain Zw26 chromosome, complete genome

```
product length = 306
Forward primer  1       TGGGTGATCGCCTTGGTGA  19
Template        440476  ...................  440494

Reverse primer  1       GGCCAGTTGCAGCTTGTTC  19
Template        440781  C..................  440763
```

>CP082928.1 Pseudomonas fulva strain ZJU1 chromosome, complete genome

```
product length = 306
Forward primer  1        TGGGTGATCGCCTTGGTGA  19
Template        3406594  ...................  3406612

Reverse primer  1        GGCCAGTTGCAGCTTGTTC  19
Template        3406899  ......C............  3406881
```

>CP093966.1 Pseudomonas aeruginosa strain ATCC BAA-2114 chromosome, complete genome

```
product length = 306
Forward primer  1       TGGGTGATCGCCTTGGTGA  19
Template        466876  ...................  466894

Reverse primer  1       GGCCAGTTGCAGCTTGTTC  19
Template        467181  .............C.....  467163
```

>CP059139.1 Pseudomonas sp. MSPm1 chromosome

```
product length = 306
Forward primer  1        TGGGTGATCGCCTTGGTGA  19
Template        4516599  ...................  4516617

Reverse primer  1        GGCCAGTTGCAGCTTGTTC  19
Template        4516904  C..................  4516886
```

>CP045302.1 Azotobacter salinestris strain KACC 13899 chromosome, complete genome

```
product length = 306
Forward primer  1        TGGGTGATCGCCTTGGTGA  19
Template        2516859  ...................  2516841
```

```
Reverse primer  1        GGCCAGTTGCAGCTTGTTC  19
Template        2516554  ......C............  2516572
```

>CP041354.1 Pseudomonas aeruginosa strain AZPAE15042 chromosome, complete genome

```
product length = 306
Forward primer  1       TGGGTGATCGCCTTGGTGA  19
Template        442171  ...................  442189

Reverse primer  1       GGCCAGTTGCAGCTTGTTC  19
Template        442476  C..................  442458
```

>LR134290.1 Pseudomonas mendocina strain NCTC10897 genome assembly, chromosome: 1

```
product length = 306
Forward primer  1        TGGGTGATCGCCTTGGTGA  19
Template        4159613  ...................  4159631

Reverse primer  1        GGCCAGTTGCAGCTTGTTC  19
Template        4159918  ............T......  4159900
```

>CP031606.1 Pseudomonas sp. phDV1 chromosome, complete genome

```
product length = 306
Forward primer  1       TGGGTGATCGCCTTGGTGA  19
Template        932084  ...................  932066

Reverse primer  1       GGCCAGTTGCAGCTTGTTC  19
Template        931779  C..................  931797
```

>CP029093.1 Pseudomonas paraeruginosa strain AR441 chromosome, complete genome

```
product length = 306
Forward primer  1       TGGGTGATCGCCTTGGTGA  19
Template        502270  ...................  502288

Reverse primer  1       GGCCAGTTGCAGCTTGTTC  19
Template        502575  C..................  502557
```

>CP020560.1 Pseudomonas paraeruginosa strain Cr1 chromosome, complete genome

```
product length = 306
Forward primer  1       TGGGTGATCGCCTTGGTGA  19
Template        447427  ...................  447445

Reverse primer  1       GGCCAGTTGCAGCTTGTTC  19
Template        447732  C..................  447714
```

>CP027657.1 Pseudomonas mendocina strain NEB698 chromosome, complete genome

```
product length = 306
Forward primer  1        TGGGTGATCGCCTTGGTGA  19
```

```
Template        1147863  ...................  1147881

Reverse primer  1        GGCCAGTTGCAGCTTGTTC  19
Template        1148168  C..................  1148150
```

>CP027169.1 Pseudomonas paraeruginosa strain AR_0356 chromosome, complete genome

```
product length = 306
Forward primer  1        TGGGTGATCGCCTTGGTGA  19
Template        1762588  ...................  1762606

Reverse primer  1        GGCCAGTTGCAGCTTGTTC  19
Template        1762893  C..................  1762875
```

>CP020100.1 Pseudomonas phragmitis strain S-6-2 chromosome, complete genome

```
product length = 306
Forward primer  1       TGGGTGATCGCCTTGGTGA  19
Template        413328  ...................  413310

Reverse primer  1       GGCCAGTTGCAGCTTGTTC  19
Template        413023  C..................  413041
```

>CP016162.1 Pseudomonas alcaliphila JAB1, complete genome

```
product length = 306
Forward primer  1        TGGGTGATCGCCTTGGTGA  19
Template        4248545  ............C......  4248563

Reverse primer  1        GGCCAGTTGCAGCTTGTTC  19
Template        4248850  ...................  4248832
```

>LK391695.1 Pseudomonas pseudoalcaligenes genome assembly Ppseudo_Pac, chromosome : I

```
product length = 306
Forward primer  1       TGGGTGATCGCCTTGGTGA  19
Template        882556  ...................  882538

Reverse primer  1       GGCCAGTTGCAGCTTGTTC  19
Template        882251  C..................  882269
```

>HG916826.1 Pseudomonas pseudoalcaligenes CECT 5344 complete genome

```
product length = 306
Forward primer  1       TGGGTGATCGCCTTGGTGA  19
Template        880337  ...................  880319

Reverse primer  1       GGCCAGTTGCAGCTTGTTC  19
Template        880032  C..................  880050
```

>CP068238.1 Pseudomonas aeruginosa strain A39-1 chromosome, complete genome

```
product length = 306
```

```
Forward primer  1       TGGGTGATCGCCTTGGTGA  19
Template        431322  ...................  431340

Reverse primer  1       GGCCAGTTGCAGCTTGTTC  19
Template        431627  C..................  431609
```

>CP000744.1 Pseudomonas aeruginosa PA7, complete genome

```
product length = 306
Forward primer  1       TGGGTGATCGCCTTGGTGA  19
Template        539600  ...................  539618

Reverse primer  1       GGCCAGTTGCAGCTTGTTC  19
Template        539905  C..................  539887
```

>CP005095.1 Azotobacter vinelandii CA6, complete genome

```
product length = 306
Forward primer  1       TGGGTGATCGCCTTGGTGA  19
Template        184082  ........T..........  184100

Reverse primer  1       GGCCAGTTGCAGCTTGTTC  19
Template        184387  ............T......  184369
```

>CP005094.1 Azotobacter vinelandii CA, complete genome

```
product length = 306
Forward primer  1       TGGGTGATCGCCTTGGTGA  19
Template        184082  ........T..........  184100

Reverse primer  1       GGCCAGTTGCAGCTTGTTC  19
Template        184387  ............T......  184369
```

>CP001157.1 Azotobacter vinelandii DJ, complete genome

```
product length = 306
Forward primer  1       TGGGTGATCGCCTTGGTGA  19
Template        184082  ........T..........  184100

Reverse primer  1       GGCCAGTTGCAGCTTGTTC  19
Template        184387  ............T......  184369
```

>CP095766.1 Pseudomonas chengduensis strain T1624 chromosome, complete genome

```
product length = 306
Forward primer  1        TGGGTGATCGCCTTGGTGA  19
Template        4491919  ............C......  4491937

Reverse primer  1        GGCCAGTTGCAGCTTGTTC  19
Template        4492224  C..................  4492206
```

>CP072610.1 Pseudomonas wenzhouensis strain A20 chromosome, complete genome

```
product length = 306
Forward primer  1        TGGGTGATCGCCTTGGTGA  19
Template        4089620  ............C......  4089638

Reverse primer  1        GGCCAGTTGCAGCTTGTTC  19
Template        4089925  C..................  4089907
```

>CP066310.1 Azotobacter chroococcum strain HR1 chromosome, complete genome

```
product length = 306
Forward primer  1        TGGGTGATCGCCTTGGTGA  19
Template        4517252  ............C......  4517270

Reverse primer  1        GGCCAGTTGCAGCTTGTTC  19
Template        4517557  ......C............  4517539
```

>CP060008.1 Pseudomonas sp. B11D7D chromosome, complete genome

```
product length = 306
Forward primer  1        TGGGTGATCGCCTTGGTGA  19
Template        2007837  ............C......  2007819

Reverse primer  1        GGCCAGTTGCAGCTTGTTC  19
Template        2007532  C..................  2007550
```

>CP043626.1 Pseudomonas denitrificans (nom. rej.) strain BG1 chromosome, complete genome

```
product length = 306
Forward primer  1        TGGGTGATCGCCTTGGTGA  19
Template        5791360  ............C......  5791378

Reverse primer  1        GGCCAGTTGCAGCTTGTTC  19
Template        5791665  ......C............  5791647
```

>CP011835.1 Azotobacter chroococcum strain B3, complete genome

```
product length = 306
Forward primer  1        TGGGTGATCGCCTTGGTGA  19
Template        4460100  ............C......  4460118

Reverse primer  1        GGCCAGTTGCAGCTTGTTC  19
Template        4460405  ......C............  4460387
```

>CP123623.1 Pseudomonas sp. CW003PS chromosome, complete genome

```
product length = 306
Forward primer  1        TGGGTGATCGCCTTGGTGA  19
Template        1437215  ............C......  1437233

Reverse primer  1        GGCCAGTTGCAGCTTGTTC  19
Template        1437520  C..................  1437502
```

>CP010415.1 Azotobacter chroococcum NCIMB 8003, complete genome

```
product length = 306
Forward primer  1        TGGGTGATCGCCTTGGTGA  19
Template        4462149  ...........C......  4462167

Reverse primer  1        GGCCAGTTGCAGCTTGTTC  19
Template        4462454  ......C............  4462436
```

>CP104580.1 Stutzerimonas stutzeri strain GD04120 chromosome, complete genome

```
product length = 306
Forward primer  1        TGGGTGATCGCCTTGGTGA  19
Template        4441055  ...........C......  4441037

Reverse primer  1        GGCCAGTTGCAGCTTGTTC  19
Template        4440750  ......C............  4440768
```

>CP115817.1 Pseudomonas mendocina strain GD22SC3150TT chromosome, complete genome

```
product length = 306
Forward primer  1        TGGGTGATCGCCTTGGTGA  19
Template        3632894  ...........C......  3632912

Reverse primer  1        GGCCAGTTGCAGCTTGTTC  19
Template        3633199  ......C............  3633181
```

>CP115669.1 Pseudomonas sp. PSE14 chromosome, complete genome

```
product length = 306
Forward primer  1       TGGGTGATCGCCTTGGTGA  19
Template        436302  ...........C......  436320

Reverse primer  1       GGCCAGTTGCAGCTTGTTC  19
Template        436607  ......C............  436589
```

>CP067126.1 Paracoccus alcaliphilus strain TK 1015 = DSM 8512 plasmid p402633, complete sequence

```
product length = 303
Forward primer  1       TGGGTGATCGCCTTGGTGA  19
Template        394547  .............C....  394529

Reverse primer  1       GGCCAGTTGCAGCTTGTTC  19
Template        394245  .A.................  394263
```

>CP088004.1 Stutzerimonas stutzeri strain SB-1 chromosome, complete genome

```
product length = 306
Forward primer  1       TGGGTGATCGCCTTGGTGA  19
Template        319501  ...........C......  319519

Reverse primer  1       GGCCAGTTGCAGCTTGTTC  19
Template        319806  ......C............  319788
```

>CP098606.1 Pseudomonas mendocina strain MET-2 chromosome, complete genome

```
product length = 306
Forward primer  1        TGGGTGATCGCCTTGGTGA  19
Template        4707410  ............C......  4707428

Reverse primer  1        GGCCAGTTGCAGCTTGTTC  19
Template        4707715  ......C............  4707697
```

>CP107549.1 Pseudomonas sp. Z8(2022) chromosome, complete genome

```
product length = 306
Forward primer  1       TGGGTGATCGCCTTGGTGA  19
Template        822515  ............C......  822497

Reverse primer  1       GGCCAGTTGCAGCTTGTTC  19
Template        822210  ......C............  822228
```

>CP104727.1 Pseudomonas citronellolis strain G5.80 chromosome, complete genome

```
product length = 306
Forward primer  1        TGGGTGATCGCCTTGGTGA  19
Template        5184947  ............C......  5184929

Reverse primer  1        GGCCAGTTGCAGCTTGTTC  19
Template        5184642  ......C............  5184660
```

>CP104011.1 Pseudomonas sp. GCEP-101 chromosome, complete genome

```
product length = 306
Forward primer  1        TGGGTGATCGCCTTGGTGA  19
Template        2320117  ............C......  2320135

Reverse primer  1        GGCCAGTTGCAGCTTGTTC  19
Template        2320422  ......C............  2320404
```

>CP098731.1 Stutzerimonas stutzeri strain NRCB010 chromosome, complete genome

```
product length = 306
Forward primer  1        TGGGTGATCGCCTTGGTGA  19
Template        4275748  ............C......  4275730

Reverse primer  1        GGCCAGTTGCAGCTTGTTC  19
Template        4275443  ......C............  4275461
```

>CP102830.1 Pseudomonas sp. LS44 chromosome, complete genome

```
product length = 306
Forward primer  1        TGGGTGATCGCCTTGGTGA  19
Template        1932836  ............C......  1932818

Reverse primer  1        GGCCAGTTGCAGCTTGTTC  19
Template        1932531  ......C............  1932549
```

>CP101752.1 Pseudomonas citronellolis strain C12 chromosome, complete genome

```
product length = 306
Forward primer  1        TGGGTGATCGCCTTGGTGA  19
Template        2404357  ............C......  2404375

Reverse primer  1        GGCCAGTTGCAGCTTGTTC  19
Template        2404662  ......C............  2404644
```

>CP100553.1 Pseudomonas hydrolytica strain KHPS2 chromosome, complete genome

```
product length = 306
Forward primer  1        TGGGTGATCGCCTTGGTGA  19
Template        4481987  ............C......  4482005

Reverse primer  1        GGCCAGTTGCAGCTTGTTC  19
Template        4482292  ......C............  4482274
```

>CP062162.1 Stutzerimonas stutzeri strain 2020WEIHUA_G chromosome, complete genome

```
product length = 306
Forward primer  1        TGGGTGATCGCCTTGGTGA  19
Template        4257073  ............C......  4257055

Reverse primer  1        GGCCAGTTGCAGCTTGTTC  19
Template        4256768  ......C............  4256786
```

>AP025273.1 Pseudomonas alcaligenes MRCP2 DNA, complete genome

```
product length = 306
Forward primer  1        TGGGTGATCGCCTTGGTGA  19
Template        2034523  ............C......  2034541

Reverse primer  1        GGCCAGTTGCAGCTTGTTC  19
Template        2034828  ......C............  2034810
```

>CP078087.1 Stutzerimonas stutzeri strain YWX-1 chromosome, complete genome

```
product length = 306
Forward primer  1        TGGGTGATCGCCTTGGTGA  19
Template        4186726  ............C......  4186708

Reverse primer  1        GGCCAGTTGCAGCTTGTTC  19
Template        4186421  ......C............  4186439
```

>CP068551.1 Pseudomonas khazarica strain ODT-83 chromosome, complete genome

```
product length = 306
Forward primer  1        TGGGTGATCGCCTTGGTGA  19
Template        4380630  ............C......  4380648

Reverse primer  1        GGCCAGTTGCAGCTTGTTC  19
Template        4380935  ......C............  4380917
```

>CP060009.1 Pseudomonas sediminis strain B10D7D chromosome, complete genome

```
product length = 306
Forward primer  1        TGGGTGATCGCCTTGGTGA  19
Template        1261834  ............C......  1261816

Reverse primer  1        GGCCAGTTGCAGCTTGTTC  19
Template        1261529  ......C............  1261547
```

>CP047698.1 Pseudomonas knackmussii strain N1-2 chromosome, complete genome

```
product length = 306
Forward primer  1       TGGGTGATCGCCTTGGTGA  19
Template        524782  ............C......  524800

Reverse primer  1       GGCCAGTTGCAGCTTGTTC  19
Template        525087  ......C............  525069
```

>CP045359.1 Pseudomonas sp. THAF42 chromosome, complete genome

```
product length = 306
Forward primer  1        TGGGTGATCGCCTTGGTGA  19
Template        4283674  ............C......  4283692

Reverse primer  1        GGCCAGTTGCAGCTTGTTC  19
Template        4283979  ......C............  4283961
```

>CP045349.1 Pseudomonas sp. THAF187a chromosome, complete genome

```
product length = 306
Forward primer  1        TGGGTGATCGCCTTGGTGA  19
Template        4284216  ............C......  4284234

Reverse primer  1        GGCCAGTTGCAGCTTGTTC  19
Template        4284521  ......C............  4284503
```

>CP045118.1 Pseudomonas sp. SCB32 chromosome, complete genome

```
product length = 306
Forward primer  1       TGGGTGATCGCCTTGGTGA  19
Template        460717  ............C......  460735

Reverse primer  1       GGCCAGTTGCAGCTTGTTC  19
Template        461022  ......C............  461004
```

>LR134319.1 Pseudomonas stutzeri strain NCTC10450 genome assembly, chromosome: 1

```
product length = 306
Forward primer  1       TGGGTGATCGCCTTGGTGA  19
Template        318309  ............C......  318327

Reverse primer  1       GGCCAGTTGCAGCTTGTTC  19
Template        318614  ......C............  318596
```

>CP029829.1 Azospirillum ramasamyi strain M2T2B2 chromosome, complete genome

```
product length = 2526
Forward primer  1        TGGGTGATCGCCTTGGTGA  19
Template        1405171  .............C.....  1405189

Forward primer  1        TGGGTGATCGCCTTGGTGA  19
Template        1407696  .............C.....  1407678
```

>CP027664.1 Stutzerimonas stutzeri strain 1W1-1A chromosome, complete genome

```
product length = 306
Forward primer  1        TGGGTGATCGCCTTGGTGA  19
Template        1121545  ............C......  1121527

Reverse primer  1        GGCCAGTTGCAGCTTGTTC  19
Template        1121240  ......C............  1121258
```

>LT629751.1 Pseudomonas oryzae strain KCTC 32247 genome assembly, chromosome: I

```
product length = 306
Forward primer  1        TGGGTGATCGCCTTGGTGA  19
Template        4260866  ............C......  4260884

Reverse primer  1        GGCCAGTTGCAGCTTGTTC  19
Template        4261171  ......C............  4261153
```

>CP015285.1 Azospirillum humicireducens strain SgZ-5 chromosome, complete genome

```
product length = 2525
Forward primer  1        TGGGTGATCGCCTTGGTGA  19
Template        1550464  .............C.....  1550482

Forward primer  1        TGGGTGATCGCCTTGGTGA  19
Template        1552988  .............C.....  1552970
```

>CP014784.1 Pseudomonas alcaligenes strain NEB 585 chromosome, complete genome

```
product length = 306
Forward primer  1        TGGGTGATCGCCTTGGTGA  19
Template        4139000  ............C......  4138982

Reverse primer  1        GGCCAGTTGCAGCTTGTTC  19
Template        4138695  ......C............  4138713
```

>CP035677.1 Pseudomonas sp. REST10 chromosome

```
product length = 306
Forward primer  1        TGGGTGATCGCCTTGGTGA  19
Template        4340103  ............C......  4340121

Reverse primer  1        GGCCAGTTGCAGCTTGTTC  19
Template        4340408  ......C............  4340390
```

>CP091174.1 Stutzerimonas stutzeri strain XX1 chromosome, complete genome

```
product length = 306
Forward primer  1       TGGGTGATCGCCTTGGTGA  19
Template        694741  ............C......  694759

Reverse primer  1       GGCCAGTTGCAGCTTGTTC  19
Template        695046  ......C............  695028
```

>HG322950.1 Pseudomonas knackmussii B13 complete genome

```
product length = 306
Forward primer  1       TGGGTGATCGCCTTGGTGA  19
Template        480530  ............C......  480548

Reverse primer  1       GGCCAGTTGCAGCTTGTTC  19
Template        480835  ......C............  480817
```

>CP006579.1 Aeromonas hydrophila 4AK4, complete genome

```
product length = 306
Forward primer  1        TGGGTGATCGCCTTGGTGA  19
Template        2977401  ............C......  2977383

Reverse primer  1        GGCCAGTTGCAGCTTGTTC  19
Template        2977096  ....T..............  2977114
```

>CP004143.1 Pseudomonas denitrificans ATCC 13867, complete genome

```
product length = 306
Forward primer  1       TGGGTGATCGCCTTGGTGA  19
Template        425821  ............C......  425839

Reverse primer  1       GGCCAGTTGCAGCTTGTTC  19
Template        426126  ......C............  426108
```

>AP024354.1 Pseudomonas alcaligenes KAM426 DNA, complete genome

```
product length = 306
Forward primer  1       TGGGTGATCGCCTTGGTGA  19
Template        861756  ............C......  861774

Reverse primer  1       GGCCAGTTGCAGCTTGTTC  19
Template        862061  ......C............  862043
```

>AP010946.1 Azospirillum sp. B510 DNA, complete genome

```
product length = 2528
Forward primer  1        TGGGTGATCGCCTTGGTGA  19
Template        1903358  .............C.....  1903376

Forward primer  1        TGGGTGATCGCCTTGGTGA  19
Template        1905885  .............C.....  1905867
```

>CP000304.1 Pseudomonas stutzeri A1501, complete genome

```
product length = 306
Forward primer  1        TGGGTGATCGCCTTGGTGA  19
Template        4273633  ............C......  4273615

Reverse primer  1        GGCCAGTTGCAGCTTGTTC  19
Template        4273328  ......C............  4273346
```

>HE663493.1 Rhodospirillum photometricum DSM 122 draft genome sequence

```
product length = 306
Forward primer  1        TGGGTGATCGCCTTGGTGA  19
Template        1411227  ...............C...  1411209

Reverse primer  1        GGCCAGTTGCAGCTTGTTC  19
Template        1410922  C.................T  1410940
```

If you want to allow any of the unintended targets, check the box(es) next to the ones you accept and try again to re-search for specific primers Submit

Help

FOLLOW NCBI

Connect with NLM

National Library of Medicine
8600 Rockville Pike
Bethesda, MD 20894

Web Policies
FOIA
HHS Vulnerability Disclosure

Help
Accessibility
Careers

NLM NIH HHS USA.gov
