# Supplementary material for: Phylogenetic Aspects of Antibiotic Resistance and Biofilm Formation of P. aeruginosa Isolated from Clinical Samples
Source: Can J Infect Dis Med Microbiol. 2024 Jan 13;2024:6213873. doi: 10.1155/2024/6213873 (PMC10799695; doi:10.1155/2024/6213873)
Supplement: Supplementary Materials — Original pictures and primer-blast results. [file 6213873.f1.zip › Pel Primer-Blast results.pdf]

An official website of the United States government

Here's how you know

The .gov means it’s official.
Federal government websites often end in .gov or .mil. Before sharing sensitive information, make sure you’re on a federal government site.

The site is secure.
The https:// ensures that you are connecting to the official website and that any information you provide is encrypted and transmitted securely.

Skip to main page content

Access keys NCBI Homepage MyNCBI Homepage Main Content Main Navigation

Log in

Primer-BLAST

» JOB ID:lJ5KnzxvMccW-TT8OZwQzkOHAfxulBrhbw

PrimerBLAST users!
We want to hear from you about how PrimerBLAST can be improved.
Contact us

Primer-BLAST Results
Help

•

Input PCR template
none

Specificity of primers
Target templates were found in selected database: Nucleotide collection (nt)

Other reports
Search Summary

Detailed primer reports
You can re-search for specific primers by accepting some of the unintended targets, check the box(es) next to the ones you accept and try again to re-search for specific primers Submit
Help

Primer pair 1

	Sequence (5'->3')	Length	Tm	GC%	Self complementarity	Self 3' complementarity
Forward primer	GACTGGGTGGTGCTCGAAG	19	60.37	63.16	4.00	2.00
Reverse primer	GCGCTCCTCGGCCTGTAG	18	62.58	72.22	4.00	2.00

Products on intended targets

Products on allowed targets

Products on allowed transcript variants

Products on potentially unintended templates

Products on target templates

>CP127126.1 Pseudomonas aeruginosa PA14 strain MA3 isolate DTU_MIE chromosome, complete genome

```
product length = 312
Forward primer  1        GACTGGGTGGTGCTCGAAG  19
Template        2135181  ...................  2135199

Reverse primer  1        GCGCTCCTCGGCCTGTAG  18
Template        2135492  ..................  2135475
```

>CP121766.1 Pseudomonas aeruginosa strain 22112 chromosome, complete genome

```
product length = 312
Forward primer  1        GACTGGGTGGTGCTCGAAG  19
Template        1423952  ...................  1423970

Reverse primer  1        GCGCTCCTCGGCCTGTAG  18
Template        1424263  ..................  1424246
```

>CP127016.1 Pseudomonas aeruginosa strain TBCF10839 chromosome

```
product length = 312
Forward primer  1        GACTGGGTGGTGCTCGAAG  19
Template        2205564  ...................  2205546

Reverse primer  1        GCGCTCCTCGGCCTGTAG  18
Template        2205253  ..................  2205270
```

>CP123792.1 Pseudomonas aeruginosa strain 2021CK-01658 chromosome, complete genome

```
product length = 312
Forward primer  1        GACTGGGTGGTGCTCGAAG  19
Template        4923285  ...................  4923267

Reverse primer  1        GCGCTCCTCGGCCTGTAG  18
Template        4922974  ..................  4922991
```

>CP109685.1 Pseudomonas aeruginosa strain 2017-45-137A chromosome, complete genome

```
product length = 312
Forward primer  1       GACTGGGTGGTGCTCGAAG  19
Template        147986  ...................  148004

Reverse primer  1       GCGCTCCTCGGCCTGTAG  18
Template        148297  ..................  148280
```

>CP061073.2 Pseudomonas aeruginosa strain PAD8 chromosome, complete genome

```
product length = 312
Forward primer  1        GACTGGGTGGTGCTCGAAG  19
Template        5369692  ...................  5369710

Reverse primer  1        GCGCTCCTCGGCCTGTAG  18
Template        5370003  ..................  5369986
```

>CP123786.1 Pseudomonas aeruginosa strain 2021CK-01424 chromosome, complete genome

```
product length = 312
Forward primer  1        GACTGGGTGGTGCTCGAAG  19
Template        2324538  ...................  2324556

Reverse primer  1        GCGCTCCTCGGCCTGTAG  18
Template        2324849  ..................  2324832
```

>CP123785.1 Pseudomonas aeruginosa strain 2021CK-01267 chromosome, complete genome

```
product length = 312
Forward primer  1        GACTGGGTGGTGCTCGAAG  19
Template        2324769  ...................  2324787

Reverse primer  1        GCGCTCCTCGGCCTGTAG  18
Template        2325080  ..................  2325063
```

>CP123787.1 Pseudomonas aeruginosa strain 2020CK-00194 chromosome, complete genome

```
product length = 312
Forward primer  1        GACTGGGTGGTGCTCGAAG  19
Template        2203908  ...................  2203926

Reverse primer  1        GCGCTCCTCGGCCTGTAG  18
Template        2204219  ..................  2204202
```

>CP123789.1 Pseudomonas aeruginosa strain 2021CK-01381 chromosome, complete genome

```
product length = 312
Forward primer  1        GACTGGGTGGTGCTCGAAG  19
Template        4853975  ...................  4853957

Reverse primer  1        GCGCTCCTCGGCCTGTAG  18
Template        4853664  ..................  4853681
```

>CP123791.1 Pseudomonas aeruginosa strain 2021CK-01305 chromosome, complete genome

```
product length = 312
Forward primer  1        GACTGGGTGGTGCTCGAAG  19
Template        2304624  ...................  2304642

Reverse primer  1        GCGCTCCTCGGCCTGTAG  18
Template        2304935  ..................  2304918
```

>CP123793.1 Pseudomonas aeruginosa strain 2021CK-01107 chromosome, complete genome

```
product length = 312
Forward primer  1        GACTGGGTGGTGCTCGAAG  19
Template        2214346  ...................  2214364

Reverse primer  1        GCGCTCCTCGGCCTGTAG  18
Template        2214657  ..................  2214640
```

>CP096964.1 Pseudomonas aeruginosa strain NY13936 chromosome, complete genome

```
product length = 312
Forward primer  1        GACTGGGTGGTGCTCGAAG  19
Template        4460981  ...................  4460963

Reverse primer  1        GCGCTCCTCGGCCTGTAG  18
Template        4460670  ..................  4460687
```

>CP096961.1 Pseudomonas aeruginosa strain NY13932 chromosome, complete genome

```
product length = 312
Forward primer  1        GACTGGGTGGTGCTCGAAG  19
Template        2078197  ...................  2078215

Reverse primer  1        GCGCTCCTCGGCCTGTAG  18
Template        2078508  ..................  2078491
```

>CP096960.1 Pseudomonas aeruginosa strain NY11254 chromosome, complete genome

```
product length = 312
Forward primer  1        GACTGGGTGGTGCTCGAAG  19
Template        2138095  ...................  2138113

Reverse primer  1        GCGCTCCTCGGCCTGTAG  18
Template        2138406  ..................  2138389
```

>CP096958.1 Pseudomonas aeruginosa strain NY11210 chromosome, complete genome

```
product length = 312
Forward primer  1        GACTGGGTGGTGCTCGAAG  19
Template        2202209  ...................  2202227

Reverse primer  1        GCGCTCCTCGGCCTGTAG  18
Template        2202520  ..................  2202503
```

>CP096956.1 Pseudomonas aeruginosa strain NY11173 chromosome, complete genome

```
product length = 312
Forward primer  1        GACTGGGTGGTGCTCGAAG  19
Template        2093181  ...................  2093199

Reverse primer  1        GCGCTCCTCGGCCTGTAG  18
Template        2093492  ..................  2093475
```

>CP096953.1 Pseudomonas aeruginosa strain NY5535 chromosome, complete genome

```
product length = 312
Forward primer  1        GACTGGGTGGTGCTCGAAG  19
Template        2203849  ...................  2203867

Reverse primer  1        GCGCTCCTCGGCCTGTAG  18
Template        2204160  ..................  2204143
```

>CP096950.1 Pseudomonas aeruginosa strain NY5532 chromosome, complete genome

```
product length = 312
Forward primer  1        GACTGGGTGGTGCTCGAAG  19
Template        2150343  ...................  2150361

Reverse primer  1        GCGCTCCTCGGCCTGTAG  18
Template        2150654  ..................  2150637
```

>CP096946.1 Pseudomonas aeruginosa strain NY5530 chromosome, complete genome

```
product length = 312
Forward primer  1        GACTGGGTGGTGCTCGAAG  19
Template        2241827  ...................  2241845

Reverse primer  1        GCGCTCCTCGGCCTGTAG  18
Template        2242138  ..................  2242121
```

>CP096945.1 Pseudomonas aeruginosa strain NY5525 chromosome, complete genome

```
product length = 312
Forward primer  1        GACTGGGTGGTGCTCGAAG  19
Template        2221573  ...................  2221591

Reverse primer  1        GCGCTCCTCGGCCTGTAG  18
Template        2221884  ..................  2221867
```

>CP096942.1 Pseudomonas aeruginosa strain NY5524 chromosome, complete genome

```
product length = 312
Forward primer  1        GACTGGGTGGTGCTCGAAG  19
Template        4044673  ...................  4044655

Reverse primer  1        GCGCTCCTCGGCCTGTAG  18
Template        4044362  ..................  4044379
```

>CP096941.1 Pseudomonas aeruginosa strain NY5523 chromosome, complete genome

```
product length = 312
Forward primer  1        GACTGGGTGGTGCTCGAAG  19
Template        2138124  ...................  2138142

Reverse primer  1        GCGCTCCTCGGCCTGTAG  18
Template        2138435  ..................  2138418
```

>CP096937.1 Pseudomonas aeruginosa strain NY5520 chromosome, complete genome

```
product length = 312
Forward primer  1        GACTGGGTGGTGCTCGAAG  19
Template        2203551  ...................  2203569

Reverse primer  1        GCGCTCCTCGGCCTGTAG  18
Template        2203862  ..................  2203845
```

>CP096934.1 Pseudomonas aeruginosa strain NY5511 chromosome, complete genome

```
product length = 312
Forward primer  1        GACTGGGTGGTGCTCGAAG  19
Template        5386325  ...................  5386307

Reverse primer  1        GCGCTCCTCGGCCTGTAG  18
Template        5386014  ..................  5386031
```

>CP096932.1 Pseudomonas aeruginosa strain NY5510 chromosome, complete genome

```
product length = 312
Forward primer  1        GACTGGGTGGTGCTCGAAG  19
Template        2203837  ...................  2203855

Reverse primer  1        GCGCTCCTCGGCCTGTAG  18
Template        2204148  ..................  2204131
```

>CP096929.1 Pseudomonas aeruginosa strain NY5507 chromosome, complete genome

```
product length = 312
Forward primer  1        GACTGGGTGGTGCTCGAAG  19
Template        2242082  ...................  2242100

Reverse primer  1        GCGCTCCTCGGCCTGTAG  18
Template        2242393  ..................  2242376
```

>CP096927.1 Pseudomonas aeruginosa strain NY5506 chromosome, complete genome

```
product length = 312
Forward primer  1        GACTGGGTGGTGCTCGAAG  19
Template        2057426  ...................  2057444

Reverse primer  1        GCGCTCCTCGGCCTGTAG  18
Template        2057737  ..................  2057720
```

>CP124673.1 Pseudomonas aeruginosa strain 2022CK-00491 chromosome, complete genome

```
product length = 312
Forward primer  1        GACTGGGTGGTGCTCGAAG  19
Template        2202663  ...................  2202681

Reverse primer  1        GCGCTCCTCGGCCTGTAG  18
Template        2202974  ..................  2202957
```

>CP124674.1 Pseudomonas aeruginosa strain 2022CK-00339 chromosome, complete genome

```
product length = 312
Forward primer  1        GACTGGGTGGTGCTCGAAG  19
Template        5036121  ...................  5036103

Reverse primer  1        GCGCTCCTCGGCCTGTAG  18
Template        5035810  ..................  5035827
```

>CP124662.1 Pseudomonas aeruginosa strain 2021CK-01633 chromosome, complete genome

```
product length = 312
Forward primer  1        GACTGGGTGGTGCTCGAAG  19
Template        2203910  ...................  2203928

Reverse primer  1        GCGCTCCTCGGCCTGTAG  18
Template        2204221  ..................  2204204
```

>CP124660.1 Pseudomonas aeruginosa strain 2022CK-00160 chromosome, complete genome

```
product length = 312
Forward primer  1        GACTGGGTGGTGCTCGAAG  19
Template        2315304  ...................  2315322

Reverse primer  1        GCGCTCCTCGGCCTGTAG  18
Template        2315615  ..................  2315598
```

>CP124652.1 Pseudomonas aeruginosa strain 2020CK-00443 chromosome, complete genome

```
product length = 312
Forward primer  1        GACTGGGTGGTGCTCGAAG  19
Template        4716083  ...................  4716065

Reverse primer  1        GCGCTCCTCGGCCTGTAG  18
Template        4715772  ..................  4715789
```

>CP124649.1 Pseudomonas aeruginosa strain 2020CK-00218 chromosome, complete genome

```
product length = 312
Forward primer  1        GACTGGGTGGTGCTCGAAG  19
Template        2228507  ...................  2228525

Reverse primer  1        GCGCTCCTCGGCCTGTAG  18
Template        2228818  ..................  2228801
```

>CP124664.1 Pseudomonas aeruginosa strain 2021CK-01256 chromosome, complete genome

```
product length = 312
Forward primer  1        GACTGGGTGGTGCTCGAAG  19
Template        2132017  ...................  2132035

Reverse primer  1        GCGCTCCTCGGCCTGTAG  18
Template        2132328  ..................  2132311
```

>CP124655.1 Pseudomonas aeruginosa strain 2022CK-00096 chromosome, complete genome

```
product length = 312
Forward primer  1        GACTGGGTGGTGCTCGAAG  19
Template        4784222  ...................  4784204

Reverse primer  1        GCGCTCCTCGGCCTGTAG  18
Template        4783911  ..................  4783928
```

>CP124654.1 Pseudomonas aeruginosa strain 2021CK-01851 chromosome, complete genome

```
product length = 312
Forward primer  1        GACTGGGTGGTGCTCGAAG  19
Template        2032253  ...................  2032271

Reverse primer  1        GCGCTCCTCGGCCTGTAG  18
Template        2032564  ..................  2032547
```

>CP124657.1 Pseudomonas aeruginosa strain 2022CK-00069 chromosome, complete genome

```
product length = 312
Forward primer  1        GACTGGGTGGTGCTCGAAG  19
Template        2139296  ...................  2139314

Reverse primer  1        GCGCTCCTCGGCCTGTAG  18
Template        2139607  ..................  2139590
```

>CP124651.1 Pseudomonas aeruginosa strain 2020CK-00217 chromosome, complete genome

```
product length = 312
Forward primer  1        GACTGGGTGGTGCTCGAAG  19
Template        2204138  ...................  2204156

Reverse primer  1        GCGCTCCTCGGCCTGTAG  18
Template        2204449  ..................  2204432
```

>CP124669.1 Pseudomonas aeruginosa strain 2021CK-01494 chromosome, complete genome

```
product length = 312
Forward primer  1        GACTGGGTGGTGCTCGAAG  19
Template        2078588  ...................  2078606

Reverse primer  1        GCGCTCCTCGGCCTGTAG  18
Template        2078899  ..................  2078882
```

>CP124668.1 Pseudomonas aeruginosa strain 2021CK-01445 chromosome, complete genome

```
product length = 312
Forward primer  1        GACTGGGTGGTGCTCGAAG  19
Template        2078548  ...................  2078566

Reverse primer  1        GCGCTCCTCGGCCTGTAG  18
Template        2078859  ..................  2078842
```

>CP124666.1 Pseudomonas aeruginosa strain 2021CK-01283 chromosome, complete genome

```
product length = 312
Forward primer  1        GACTGGGTGGTGCTCGAAG  19
Template        2133122  ...................  2133140

Reverse primer  1        GCGCTCCTCGGCCTGTAG  18
Template        2133433  ..................  2133416
```

>CP124665.1 Pseudomonas aeruginosa strain 2021CK-01229 chromosome, complete genome

```
product length = 312
Forward primer  1        GACTGGGTGGTGCTCGAAG  19
Template        2133125  ...................  2133143

Reverse primer  1        GCGCTCCTCGGCCTGTAG  18
Template        2133436  ..................  2133419
```

>CP124667.1 Pseudomonas aeruginosa strain 2021CK-01315 chromosome, complete genome

```
product length = 312
Forward primer  1        GACTGGGTGGTGCTCGAAG  19
Template        4491876  ...................  4491858

Reverse primer  1        GCGCTCCTCGGCCTGTAG  18
Template        4491565  ..................  4491582
```

>CP124670.1 Pseudomonas aeruginosa strain 2021CK-01536 chromosome, complete genome

```
product length = 312
Forward primer  1        GACTGGGTGGTGCTCGAAG  19
Template        2036403  ...................  2036421

Reverse primer  1        GCGCTCCTCGGCCTGTAG  18
Template        2036714  ..................  2036697
```

>CP124646.1 Pseudomonas aeruginosa strain 2020CK-00185 chromosome, complete genome

```
product length = 312
Forward primer  1        GACTGGGTGGTGCTCGAAG  19
Template        4764250  ...................  4764232

Reverse primer  1        GCGCTCCTCGGCCTGTAG  18
Template        4763939  ..................  4763956
```

>CP124648.1 Pseudomonas aeruginosa strain 2020CK-00220 chromosome, complete genome

```
product length = 312
Forward primer  1        GACTGGGTGGTGCTCGAAG  19
Template        4619273  ...................  4619291

Reverse primer  1        GCGCTCCTCGGCCTGTAG  18
Template        4619584  ..................  4619567
```

>CP124643.1 Pseudomonas aeruginosa strain 2021CK-01197 chromosome, complete genome

```
product length = 312
Forward primer  1        GACTGGGTGGTGCTCGAAG  19
Template        2544971  ...................  2544953

Reverse primer  1        GCGCTCCTCGGCCTGTAG  18
Template        2544660  ..................  2544677
```

>CP124626.1 Pseudomonas aeruginosa strain 2021CK-01161 chromosome, complete genome

```
product length = 312
Forward primer  1        GACTGGGTGGTGCTCGAAG  19
Template        5978010  ...................  5977992

Reverse primer  1        GCGCTCCTCGGCCTGTAG  18
Template        5977699  ..................  5977716
```

>CP124632.1 Pseudomonas aeruginosa strain 2021CK-01162 chromosome, complete genome

```
product length = 312
Forward primer  1        GACTGGGTGGTGCTCGAAG  19
Template        2564241  ...................  2564259

Reverse primer  1        GCGCTCCTCGGCCTGTAG  18
Template        2564552  ..................  2564535
```

>CP124663.1 Pseudomonas aeruginosa strain 2021CK-01227 chromosome, complete genome

```
product length = 312
Forward primer  1        GACTGGGTGGTGCTCGAAG  19
Template        2133125  ...................  2133143

Reverse primer  1        GCGCTCCTCGGCCTGTAG  18
Template        2133436  ..................  2133419
```

>CP124622.1 Pseudomonas aeruginosa strain 2021CK-01159 chromosome, complete genome

```
product length = 312
Forward primer  1        GACTGGGTGGTGCTCGAAG  19
Template        4491836  ...................  4491818

Reverse primer  1        GCGCTCCTCGGCCTGTAG  18
Template        4491525  ..................  4491542
```

>CP124600.1 Pseudomonas aeruginosa strain Li010 chromosome, complete genome

```
product length = 312
Forward primer  1        GACTGGGTGGTGCTCGAAG  19
Template        4402505  ...................  4402487

Reverse primer  1        GCGCTCCTCGGCCTGTAG  18
Template        4402194  ..................  4402211
```

>CP123953.1 Pseudomonas aeruginosa strain 59 chromosome, complete genome

```
product length = 312
Forward primer  1        GACTGGGTGGTGCTCGAAG  19
Template        2116487  ...................  2116505

Reverse primer  1        GCGCTCCTCGGCCTGTAG  18
Template        2116798  ..................  2116781
```

>CP116682.1 Pseudomonas aeruginosa strain HS337 chromosome, complete genome

```
product length = 312
Forward primer  1        GACTGGGTGGTGCTCGAAG  19
Template        2184831  ...................  2184849

Reverse primer  1        GCGCTCCTCGGCCTGTAG  18
Template        2185142  ..................  2185125
```

>CP110190.1 Pseudomonas aeruginosa strain HS204 chromosome, complete genome

```
product length = 312
Forward primer  1        GACTGGGTGGTGCTCGAAG  19
Template        2043236  ...................  2043254

Reverse primer  1        GCGCTCCTCGGCCTGTAG  18
Template        2043547  ..................  2043530
```

>CP118638.1 Pseudomonas aeruginosa strain P9 chromosome, complete genome

```
product length = 312
Forward primer  1        GACTGGGTGGTGCTCGAAG  19
Template        2098369  ...................  2098387

Reverse primer  1        GCGCTCCTCGGCCTGTAG  18
Template        2098680  ..................  2098663
```

>CP118641.1 Pseudomonas aeruginosa strain P23 chromosome, complete genome

```
product length = 312
Forward primer  1        GACTGGGTGGTGCTCGAAG  19
Template        2126924  ...................  2126942

Reverse primer  1        GCGCTCCTCGGCCTGTAG  18
Template        2127235  ..................  2127218
```

>CP119298.1 Pseudomonas aeruginosa strain SNDPR-01 chromosome, complete genome

```
product length = 312
Forward primer  1        GACTGGGTGGTGCTCGAAG  19
Template        2101236  ...................  2101254

Reverse primer  1        GCGCTCCTCGGCCTGTAG  18
Template        2101547  ..................  2101530
```

>CP117300.1 Pseudomonas aeruginosa strain 0201761-1 chromosome, complete genome

```
product length = 312
Forward primer  1        GACTGGGTGGTGCTCGAAG  19
Template        2205960  ...................  2205978

Reverse primer  1        GCGCTCCTCGGCCTGTAG  18
Template        2206271  ..................  2206254
```

>CP084321.1 Pseudomonas aeruginosa strain HS18-89 chromosome, complete genome

```
product length = 312
Forward primer  1        GACTGGGTGGTGCTCGAAG  19
Template        2116873  ...................  2116891

Reverse primer  1        GCGCTCCTCGGCCTGTAG  18
Template        2117184  ..................  2117167
```

>CP117974.1 Pseudomonas aeruginosa strain B-3509 chromosome, complete genome

```
product length = 312
Forward primer  1        GACTGGGTGGTGCTCGAAG  19
Template        3359812  ...................  3359794

Reverse primer  1        GCGCTCCTCGGCCTGTAG  18
Template        3359501  ..................  3359518
```

>CP117527.1 Pseudomonas aeruginosa strain MF1 chromosome, complete genome

```
product length = 312
Forward primer  1        GACTGGGTGGTGCTCGAAG  19
Template        2148013  ...................  2148031

Reverse primer  1        GCGCTCCTCGGCCTGTAG  18
Template        2148324  ..................  2148307
```

>CP075851.1 Pseudomonas aeruginosa strain PaLo33 chromosome, complete genome

```
product length = 312
Forward primer  1        GACTGGGTGGTGCTCGAAG  19
Template        2152600  ...................  2152618

Reverse primer  1        GCGCTCCTCGGCCTGTAG  18
Template        2152911  ..................  2152894
```

>CP075849.1 Pseudomonas aeruginosa strain PaLo1 chromosome, complete genome

```
product length = 312
Forward primer  1        GACTGGGTGGTGCTCGAAG  19
Template        4596259  ...................  4596241

Reverse primer  1        GCGCTCCTCGGCCTGTAG  18
Template        4595948  ..................  4595965
```

>CP075848.1 Pseudomonas aeruginosa strain PaLo2 chromosome, complete genome

```
product length = 312
Forward primer  1        GACTGGGTGGTGCTCGAAG  19
Template        2056802  ...................  2056820

Reverse primer  1        GCGCTCCTCGGCCTGTAG  18
Template        2057113  ..................  2057096
```

>CP075847.1 Pseudomonas aeruginosa strain PaLo3 chromosome, complete genome

```
product length = 312
Forward primer  1        GACTGGGTGGTGCTCGAAG  19
Template        4960259  ...................  4960241

Reverse primer  1        GCGCTCCTCGGCCTGTAG  18
Template        4959948  ..................  4959965
```

>CP075846.1 Pseudomonas aeruginosa strain PaLo4 chromosome

```
product length = 312
Forward primer  1        GACTGGGTGGTGCTCGAAG  19
Template        4861765  ...................  4861747

Reverse primer  1        GCGCTCCTCGGCCTGTAG  18
Template        4861454  ..................  4861471
```

>CP075844.1 Pseudomonas aeruginosa strain PaLo6 chromosome, complete genome

```
product length = 312
Forward primer  1        GACTGGGTGGTGCTCGAAG  19
Template        2094098  ...................  2094116

Reverse primer  1        GCGCTCCTCGGCCTGTAG  18
Template        2094409  ..................  2094392
```

>CP075843.1 Pseudomonas aeruginosa strain PaLo7 chromosome

```
product length = 312
Forward primer  1        GACTGGGTGGTGCTCGAAG  19
Template        2156748  ...................  2156766

Reverse primer  1        GCGCTCCTCGGCCTGTAG  18
Template        2157059  ..................  2157042
```

>CP075841.1 Pseudomonas aeruginosa strain PaLo9 chromosome, complete genome

```
product length = 312
Forward primer  1        GACTGGGTGGTGCTCGAAG  19
Template        2234311  ...................  2234329

Reverse primer  1        GCGCTCCTCGGCCTGTAG  18
Template        2234622  ..................  2234605
```

>CP075840.1 Pseudomonas aeruginosa strain PaLo10 chromosome, complete genome

```
product length = 312
Forward primer  1        GACTGGGTGGTGCTCGAAG  19
Template        2057951  ...................  2057969

Reverse primer  1        GCGCTCCTCGGCCTGTAG  18
Template        2058262  ..................  2058245
```

>CP075838.1 Pseudomonas aeruginosa strain PaLo11 chromosome, complete genome

```
product length = 312
Forward primer  1        GACTGGGTGGTGCTCGAAG  19
Template        1912065  ...................  1912083

Reverse primer  1        GCGCTCCTCGGCCTGTAG  18
Template        1912376  ..................  1912359
```

>CP075836.1 Pseudomonas aeruginosa strain PaLo12 chromosome, complete genome

```
product length = 312
Forward primer  1        GACTGGGTGGTGCTCGAAG  19
Template        2075482  ...................  2075500

Reverse primer  1        GCGCTCCTCGGCCTGTAG  18
Template        2075793  ..................  2075776
```

>CP075835.1 Pseudomonas aeruginosa strain PaLo14 chromosome, complete genome

```
product length = 312
Forward primer  1        GACTGGGTGGTGCTCGAAG  19
Template        4398158  ...................  4398140

Reverse primer  1        GCGCTCCTCGGCCTGTAG  18
Template        4397847  ..................  4397864
```

>CP075833.1 Pseudomonas aeruginosa strain PaLo17 chromosome, complete genome

```
product length = 312
Forward primer  1        GACTGGGTGGTGCTCGAAG  19
Template        2056797  ...................  2056815

Reverse primer  1        GCGCTCCTCGGCCTGTAG  18
Template        2057108  ..................  2057091
```

>CP075832.1 Pseudomonas aeruginosa strain PaLo20 chromosome, complete genome

```
product length = 312
Forward primer  1        GACTGGGTGGTGCTCGAAG  19
Template        4730281  ...................  4730263

Reverse primer  1        GCGCTCCTCGGCCTGTAG  18
Template        4729970  ..................  4729987
```

>CP075831.1 Pseudomonas aeruginosa strain PaLo21 chromosome, complete genome

```
product length = 312
Forward primer  1        GACTGGGTGGTGCTCGAAG  19
Template        2081678  ...................  2081696

Reverse primer  1        GCGCTCCTCGGCCTGTAG  18
Template        2081989  ..................  2081972
```

>CP075830.1 Pseudomonas aeruginosa strain PaLo22 chromosome, complete genome

```
product length = 312
Forward primer  1        GACTGGGTGGTGCTCGAAG  19
Template        2551453  ...................  2551471

Reverse primer  1        GCGCTCCTCGGCCTGTAG  18
Template        2551764  ..................  2551747
```

>CP075828.1 Pseudomonas aeruginosa strain PaLo26 chromosome, complete genome

```
product length = 312
Forward primer  1        GACTGGGTGGTGCTCGAAG  19
Template        2096017  ...................  2096035

Reverse primer  1        GCGCTCCTCGGCCTGTAG  18
Template        2096328  ..................  2096311
```

>CP075827.1 Pseudomonas aeruginosa strain PaLo27 chromosome, complete genome

```
product length = 312
Forward primer  1        GACTGGGTGGTGCTCGAAG  19
Template        2169162  ...................  2169180

Reverse primer  1        GCGCTCCTCGGCCTGTAG  18
Template        2169473  ..................  2169456
```

>CP075826.1 Pseudomonas aeruginosa strain PaLo29 chromosome, complete genome

```
product length = 312
Forward primer  1        GACTGGGTGGTGCTCGAAG  19
Template        2038732  ...................  2038750

Reverse primer  1        GCGCTCCTCGGCCTGTAG  18
Template        2039043  ..................  2039026
```

>CP075825.1 Pseudomonas aeruginosa strain PaLo30 chromosome, complete genome

```
product length = 312
Forward primer  1        GACTGGGTGGTGCTCGAAG  19
Template        2056830  ...................  2056848

Reverse primer  1        GCGCTCCTCGGCCTGTAG  18
Template        2057141  ..................  2057124
```

>CP075824.1 Pseudomonas aeruginosa strain PaLo31 chromosome, complete genome

```
product length = 312
Forward primer  1        GACTGGGTGGTGCTCGAAG  19
Template        2157679  ...................  2157697

Reverse primer  1        GCGCTCCTCGGCCTGTAG  18
Template        2157990  ..................  2157973
```

>CP075823.1 Pseudomonas aeruginosa strain PaLo32 chromosome, complete genome

```
product length = 312
Forward primer  1        GACTGGGTGGTGCTCGAAG  19
Template        2152543  ...................  2152561

Reverse primer  1        GCGCTCCTCGGCCTGTAG  18
Template        2152854  ..................  2152837
```

>CP075822.1 Pseudomonas aeruginosa strain PaLo34 chromosome, complete genome

```
product length = 312
Forward primer  1        GACTGGGTGGTGCTCGAAG  19
Template        2056829  ...................  2056847

Reverse primer  1        GCGCTCCTCGGCCTGTAG  18
Template        2057140  ..................  2057123
```

>CP075821.1 Pseudomonas aeruginosa strain PaLo35 chromosome, complete genome

```
product length = 312
Forward primer  1        GACTGGGTGGTGCTCGAAG  19
Template        4334090  ...................  4334072

Reverse primer  1        GCGCTCCTCGGCCTGTAG  18
Template        4333779  ..................  4333796
```

>CP075820.1 Pseudomonas aeruginosa strain PaLo36 chromosome, complete genome

```
product length = 312
Forward primer  1        GACTGGGTGGTGCTCGAAG  19
Template        2126474  ...................  2126492

Reverse primer  1        GCGCTCCTCGGCCTGTAG  18
Template        2126785  ..................  2126768
```

>CP075819.1 Pseudomonas aeruginosa strain PaLo37 chromosome, complete genome

```
product length = 312
Forward primer  1        GACTGGGTGGTGCTCGAAG  19
Template        3721633  ...................  3721615

Reverse primer  1        GCGCTCCTCGGCCTGTAG  18
Template        3721322  ..................  3721339
```

>CP075818.1 Pseudomonas aeruginosa strain PaLo38 chromosome, complete genome

```
product length = 312
Forward primer  1        GACTGGGTGGTGCTCGAAG  19
Template        2056813  ...................  2056831

Reverse primer  1        GCGCTCCTCGGCCTGTAG  18
Template        2057124  ..................  2057107
```

>CP075817.1 Pseudomonas aeruginosa strain PaLo39 chromosome, complete genome

```
product length = 312
Forward primer  1        GACTGGGTGGTGCTCGAAG  19
Template        4718557  ...................  4718539

Reverse primer  1        GCGCTCCTCGGCCTGTAG  18
Template        4718246  ..................  4718263
```

>CP075816.1 Pseudomonas aeruginosa strain PaLo40 chromosome, complete genome

```
product length = 312
Forward primer  1        GACTGGGTGGTGCTCGAAG  19
Template        3993123  ...................  3993105

Reverse primer  1        GCGCTCCTCGGCCTGTAG  18
Template        3992812  ..................  3992829
```

>CP075815.1 Pseudomonas aeruginosa strain PaLo43 chromosome, complete genome

```
product length = 312
Forward primer  1        GACTGGGTGGTGCTCGAAG  19
Template        3939679  ...................  3939661

Reverse primer  1        GCGCTCCTCGGCCTGTAG  18
Template        3939368  ..................  3939385
```

>CP075814.1 Pseudomonas aeruginosa strain PaLo44 chromosome, complete genome

```
product length = 312
Forward primer  1        GACTGGGTGGTGCTCGAAG  19
Template        2038549  ...................  2038567

Reverse primer  1        GCGCTCCTCGGCCTGTAG  18
Template        2038860  ..................  2038843
```

>CP075813.1 Pseudomonas aeruginosa strain PaLo45 chromosome, complete genome

```
product length = 312
Forward primer  1        GACTGGGTGGTGCTCGAAG  19
Template        2053768  ...................  2053786

Reverse primer  1        GCGCTCCTCGGCCTGTAG  18
Template        2054079  ..................  2054062
```

>CP075812.1 Pseudomonas aeruginosa strain PaLo46 chromosome, complete genome

```
product length = 312
Forward primer  1        GACTGGGTGGTGCTCGAAG  19
Template        2150993  ...................  2151011

Reverse primer  1        GCGCTCCTCGGCCTGTAG  18
Template        2151304  ..................  2151287
```

>CP075810.1 Pseudomonas aeruginosa strain PaLo166 chromosome, complete genome

```
product length = 312
Forward primer  1        GACTGGGTGGTGCTCGAAG  19
Template        2126810  ...................  2126828

Reverse primer  1        GCGCTCCTCGGCCTGTAG  18
Template        2127121  ..................  2127104
```

>CP075809.1 Pseudomonas aeruginosa strain PaLo170 chromosome, complete genome

```
product length = 312
Forward primer  1        GACTGGGTGGTGCTCGAAG  19
Template        2081981  ...................  2081999

Reverse primer  1        GCGCTCCTCGGCCTGTAG  18
Template        2082292  ..................  2082275
```

>CP075808.1 Pseudomonas aeruginosa strain PaLo185 chromosome

```
product length = 312
Forward primer  1        GACTGGGTGGTGCTCGAAG  19
Template        2086746  ...................  2086764

Reverse primer  1        GCGCTCCTCGGCCTGTAG  18
Template        2087057  ..................  2087040
```

>CP075807.1 Pseudomonas aeruginosa strain PaLo191 chromosome, complete genome

```
product length = 312
Forward primer  1        GACTGGGTGGTGCTCGAAG  19
Template        4142559  ...................  4142541

Reverse primer  1        GCGCTCCTCGGCCTGTAG  18
Template        4142248  ..................  4142265
```

>CP075806.1 Pseudomonas aeruginosa strain PaLo226 chromosome, complete genome

```
product length = 312
Forward primer  1        GACTGGGTGGTGCTCGAAG  19
Template        2034558  ...................  2034576

Reverse primer  1        GCGCTCCTCGGCCTGTAG  18
Template        2034869  ..................  2034852
```

>CP075805.1 Pseudomonas aeruginosa strain PaLo227 chromosome, complete genome

```
product length = 312
Forward primer  1        GACTGGGTGGTGCTCGAAG  19
Template        2034581  ...................  2034599

Reverse primer  1        GCGCTCCTCGGCCTGTAG  18
Template        2034892  ..................  2034875
```

>CP075804.1 Pseudomonas aeruginosa strain PaLo228 chromosome, complete genome

```
product length = 312
Forward primer  1        GACTGGGTGGTGCTCGAAG  19
Template        2034558  ...................  2034576

Reverse primer  1        GCGCTCCTCGGCCTGTAG  18
Template        2034869  ..................  2034852
```

>CP075803.1 Pseudomonas aeruginosa strain PaLo229 chromosome, complete genome

```
product length = 312
Forward primer  1        GACTGGGTGGTGCTCGAAG  19
Template        2102319  ...................  2102337

Reverse primer  1        GCGCTCCTCGGCCTGTAG  18
Template        2102630  ..................  2102613
```

>CP075802.1 Pseudomonas aeruginosa strain PaLo240 chromosome, complete genome

```
product length = 312
Forward primer  1        GACTGGGTGGTGCTCGAAG  19
Template        2152660  ...................  2152678

Reverse primer  1        GCGCTCCTCGGCCTGTAG  18
Template        2152971  ..................  2152954
```

>CP075801.1 Pseudomonas aeruginosa strain PaLo249 chromosome, complete genome

```
product length = 312
Forward primer  1        GACTGGGTGGTGCTCGAAG  19
Template        2034574  ...................  2034592

Reverse primer  1        GCGCTCCTCGGCCTGTAG  18
Template        2034885  ..................  2034868
```

>CP075800.1 Pseudomonas aeruginosa strain PaLo297 chromosome, complete genome

```
product length = 312
Forward primer  1        GACTGGGTGGTGCTCGAAG  19
Template        2069484  ...................  2069502

Reverse primer  1        GCGCTCCTCGGCCTGTAG  18
Template        2069795  ..................  2069778
```

>CP075799.1 Pseudomonas aeruginosa strain PaLo310 chromosome, complete genome

```
product length = 312
Forward primer  1        GACTGGGTGGTGCTCGAAG  19
Template        2084348  ...................  2084366

Reverse primer  1        GCGCTCCTCGGCCTGTAG  18
Template        2084659  ..................  2084642
```

>CP075798.1 Pseudomonas aeruginosa strain PaLo323 chromosome, complete genome

```
product length = 312
Forward primer  1        GACTGGGTGGTGCTCGAAG  19
Template        2068785  ...................  2068803

Reverse primer  1        GCGCTCCTCGGCCTGTAG  18
Template        2069096  ..................  2069079
```

>CP075796.1 Pseudomonas aeruginosa strain PaLo402 chromosome, complete genome

```
product length = 312
Forward primer  1        GACTGGGTGGTGCTCGAAG  19
Template        2053163  ...................  2053181

Reverse primer  1        GCGCTCCTCGGCCTGTAG  18
Template        2053474  ..................  2053457
```

>CP075787.1 Pseudomonas aeruginosa strain PaLo504 chromosome, complete genome

```
product length = 312
Forward primer  1        GACTGGGTGGTGCTCGAAG  19
Template        2065649  ...................  2065667

Reverse primer  1        GCGCTCCTCGGCCTGTAG  18
Template        2065960  ..................  2065943
```

>CP075785.1 Pseudomonas aeruginosa strain PaLo505 chromosome, complete genome

```
product length = 312
Forward primer  1        GACTGGGTGGTGCTCGAAG  19
Template        2097142  ...................  2097160

Reverse primer  1        GCGCTCCTCGGCCTGTAG  18
Template        2097453  ..................  2097436
```

>CP075784.1 Pseudomonas aeruginosa strain PaLo507 chromosome, complete genome

```
product length = 312
Forward primer  1        GACTGGGTGGTGCTCGAAG  19
Template        2032819  ...................  2032837

Reverse primer  1        GCGCTCCTCGGCCTGTAG  18
Template        2033130  ..................  2033113
```

>CP075783.1 Pseudomonas aeruginosa strain PaLo508 chromosome, complete genome

```
product length = 312
Forward primer  1        GACTGGGTGGTGCTCGAAG  19
Template        2100543  ...................  2100561

Reverse primer  1        GCGCTCCTCGGCCTGTAG  18
Template        2100854  ..................  2100837
```

>CP075782.1 Pseudomonas aeruginosa strain PaLo509 chromosome, complete genome

```
product length = 312
Forward primer  1        GACTGGGTGGTGCTCGAAG  19
Template        2100512  ...................  2100530

Reverse primer  1        GCGCTCCTCGGCCTGTAG  18
Template        2100823  ..................  2100806
```

>CP075781.1 Pseudomonas aeruginosa strain PaLo512 chromosome, complete genome

```
product length = 312
Forward primer  1        GACTGGGTGGTGCTCGAAG  19
Template        2159387  ...................  2159405

Reverse primer  1        GCGCTCCTCGGCCTGTAG  18
Template        2159698  ..................  2159681
```

>CP075780.1 Pseudomonas aeruginosa strain PaLo524 chromosome, complete genome

```
product length = 312
Forward primer  1        GACTGGGTGGTGCTCGAAG  19
Template        2074935  ...................  2074953

Reverse primer  1        GCGCTCCTCGGCCTGTAG  18
Template        2075246  ..................  2075229
```

>CP075779.1 Pseudomonas aeruginosa strain PaLo526 chromosome, complete genome

```
product length = 312
Forward primer  1        GACTGGGTGGTGCTCGAAG  19
Template        2088475  ...................  2088493

Reverse primer  1        GCGCTCCTCGGCCTGTAG  18
Template        2088786  ..................  2088769
```

>CP075778.1 Pseudomonas aeruginosa strain PaLo527 chromosome, complete genome

```
product length = 312
Forward primer  1        GACTGGGTGGTGCTCGAAG  19
Template        2022217  ...................  2022235

Reverse primer  1        GCGCTCCTCGGCCTGTAG  18
Template        2022528  ..................  2022511
```

>CP075777.1 Pseudomonas aeruginosa strain PaLo528 chromosome, complete genome

```
product length = 312
Forward primer  1        GACTGGGTGGTGCTCGAAG  19
Template        2047801  ...................  2047819

Reverse primer  1        GCGCTCCTCGGCCTGTAG  18
Template        2048112  ..................  2048095
```

>CP075776.1 Pseudomonas aeruginosa strain PaLo529 chromosome, complete genome

```
product length = 312
Forward primer  1        GACTGGGTGGTGCTCGAAG  19
Template        2088648  ...................  2088666

Reverse primer  1        GCGCTCCTCGGCCTGTAG  18
Template        2088959  ..................  2088942
```

>CP075773.1 Pseudomonas aeruginosa strain PaLo530 chromosome, complete genome

```
product length = 312
Forward primer  1        GACTGGGTGGTGCTCGAAG  19
Template        2151887  ...................  2151905

Reverse primer  1        GCGCTCCTCGGCCTGTAG  18
Template        2152198  ..................  2152181
```

>CP075771.1 Pseudomonas aeruginosa strain PaLo532 chromosome, complete genome

```
product length = 312
Forward primer  1        GACTGGGTGGTGCTCGAAG  19
Template        2171351  ...................  2171369

Reverse primer  1        GCGCTCCTCGGCCTGTAG  18
Template        2171662  ..................  2171645
```

>CP075769.1 Pseudomonas aeruginosa strain PaLo533 chromosome, complete genome

```
product length = 312
Forward primer  1        GACTGGGTGGTGCTCGAAG  19
Template        2301927  ...................  2301945

Reverse primer  1        GCGCTCCTCGGCCTGTAG  18
Template        2302238  ..................  2302221
```

>CP075768.1 Pseudomonas aeruginosa strain PaLo535 chromosome

```
product length = 312
Forward primer  1        GACTGGGTGGTGCTCGAAG  19
Template        1951751  ...................  1951769

Reverse primer  1        GCGCTCCTCGGCCTGTAG  18
Template        1952062  ..................  1952045
```

>CP075767.1 Pseudomonas aeruginosa strain PaLo536 chromosome, complete genome

```
product length = 312
Forward primer  1        GACTGGGTGGTGCTCGAAG  19
Template        2100277  ...................  2100295

Reverse primer  1        GCGCTCCTCGGCCTGTAG  18
Template        2100588  ..................  2100571
```

>CP075766.1 Pseudomonas aeruginosa strain PaLo538 chromosome, complete genome

```
product length = 312
Forward primer  1        GACTGGGTGGTGCTCGAAG  19
Template        2139528  ...................  2139546

Reverse primer  1        GCGCTCCTCGGCCTGTAG  18
Template        2139839  ..................  2139822
```

>CP075765.1 Pseudomonas aeruginosa strain PaLo539 chromosome, complete genome

```
product length = 312
Forward primer  1        GACTGGGTGGTGCTCGAAG  19
Template        2131521  ...................  2131539

Reverse primer  1        GCGCTCCTCGGCCTGTAG  18
Template        2131832  ..................  2131815
```

>CP075764.1 Pseudomonas aeruginosa strain PaLo541 chromosome, complete genome

```
product length = 312
Forward primer  1        GACTGGGTGGTGCTCGAAG  19
Template        2022906  ...................  2022924

Reverse primer  1        GCGCTCCTCGGCCTGTAG  18
Template        2023217  ..................  2023200
```

>CP075763.1 Pseudomonas aeruginosa strain PaLo543 chromosome, complete genome

```
product length = 312
Forward primer  1        GACTGGGTGGTGCTCGAAG  19
Template        2349254  ...................  2349272

Reverse primer  1        GCGCTCCTCGGCCTGTAG  18
Template        2349565  ..................  2349548
```

>CP075762.1 Pseudomonas aeruginosa strain PaLo544 chromosome, complete genome

```
product length = 312
Forward primer  1        GACTGGGTGGTGCTCGAAG  19
Template        2090627  ...................  2090645

Reverse primer  1        GCGCTCCTCGGCCTGTAG  18
Template        2090938  ..................  2090921
```

>CP075761.1 Pseudomonas aeruginosa strain PaLo545 chromosome

```
product length = 312
Forward primer  1        GACTGGGTGGTGCTCGAAG  19
Template        2195980  ...................  2195998

Reverse primer  1        GCGCTCCTCGGCCTGTAG  18
Template        2196291  ..................  2196274
```

>CP075760.1 Pseudomonas aeruginosa strain PaLo550 chromosome, complete genome

```
product length = 312
Forward primer  1        GACTGGGTGGTGCTCGAAG  19
Template        2125908  ...................  2125926

Reverse primer  1        GCGCTCCTCGGCCTGTAG  18
Template        2126219  ..................  2126202
```

>CP075757.1 Pseudomonas aeruginosa strain PaLo552 chromosome, complete genome

```
product length = 312
Forward primer  1        GACTGGGTGGTGCTCGAAG  19
Template        2078950  ...................  2078968

Reverse primer  1        GCGCTCCTCGGCCTGTAG  18
Template        2079261  ..................  2079244
```

>CP075755.1 Pseudomonas aeruginosa strain PaLo553 chromosome, complete genome

```
product length = 312
Forward primer  1        GACTGGGTGGTGCTCGAAG  19
Template        4514454  ...................  4514436

Reverse primer  1        GCGCTCCTCGGCCTGTAG  18
Template        4514143  ..................  4514160
```

>CP075754.1 Pseudomonas aeruginosa strain PaLo555 chromosome, complete genome

```
product length = 312
Forward primer  1        GACTGGGTGGTGCTCGAAG  19
Template        2050022  ...................  2050040

Reverse primer  1        GCGCTCCTCGGCCTGTAG  18
Template        2050333  ..................  2050316
```

>CP075753.1 Pseudomonas aeruginosa strain PaLo556 chromosome, complete genome

```
product length = 312
Forward primer  1        GACTGGGTGGTGCTCGAAG  19
Template        2077798  ...................  2077816

Reverse primer  1        GCGCTCCTCGGCCTGTAG  18
Template        2078109  ..................  2078092
```

>CP075752.1 Pseudomonas aeruginosa strain PaLo557 chromosome, complete genome

```
product length = 312
Forward primer  1        GACTGGGTGGTGCTCGAAG  19
Template        2189830  ...................  2189848

Reverse primer  1        GCGCTCCTCGGCCTGTAG  18
Template        2190141  ..................  2190124
```

>CP075751.1 Pseudomonas aeruginosa strain PaLo561 chromosome, complete genome

```
product length = 312
Forward primer  1        GACTGGGTGGTGCTCGAAG  19
Template        2145473  ...................  2145491

Reverse primer  1        GCGCTCCTCGGCCTGTAG  18
Template        2145784  ..................  2145767
```

>CP075750.1 Pseudomonas aeruginosa strain PaLo563 chromosome, complete genome

```
product length = 312
Forward primer  1        GACTGGGTGGTGCTCGAAG  19
Template        4779738  ...................  4779720

Reverse primer  1        GCGCTCCTCGGCCTGTAG  18
Template        4779427  ..................  4779444
```

>CP075749.1 Pseudomonas aeruginosa strain PaLo564 chromosome, complete genome

```
product length = 312
Forward primer  1        GACTGGGTGGTGCTCGAAG  19
Template        2184708  ...................  2184726

Reverse primer  1        GCGCTCCTCGGCCTGTAG  18
Template        2185019  ..................  2185002
```

>CP075748.1 Pseudomonas aeruginosa strain PaLo565 chromosome, complete genome

```
product length = 312
Forward primer  1        GACTGGGTGGTGCTCGAAG  19
Template        2995494  ...................  2995512

Reverse primer  1        GCGCTCCTCGGCCTGTAG  18
Template        2995805  ..................  2995788
```

>CP116723.1 Pseudomonas aeruginosa strain 2872 chromosome

```
product length = 312
Forward primer  1        GACTGGGTGGTGCTCGAAG  19
Template        4975293  ...................  4975275

Reverse primer  1        GCGCTCCTCGGCCTGTAG  18
Template        4974982  ..................  4974999
```

>CP116725.1 Pseudomonas aeruginosa strain 2881 chromosome, complete genome

```
product length = 312
Forward primer  1        GACTGGGTGGTGCTCGAAG  19
Template        2279703  ...................  2279721

Reverse primer  1        GCGCTCCTCGGCCTGTAG  18
Template        2280014  ..................  2279997
```

>CP116722.1 Pseudomonas aeruginosa strain 2868 chromosome, complete genome

```
product length = 312
Forward primer  1        GACTGGGTGGTGCTCGAAG  19
Template        2257500  ...................  2257518

Reverse primer  1        GCGCTCCTCGGCCTGTAG  18
Template        2257811  ..................  2257794
```

>CP116717.1 Pseudomonas aeruginosa strain 2857 chromosome, complete genome

```
product length = 312
Forward primer  1        GACTGGGTGGTGCTCGAAG  19
Template        2340711  ...................  2340729

Reverse primer  1        GCGCTCCTCGGCCTGTAG  18
Template        2341022  ..................  2341005
```

>CP116727.1 Pseudomonas aeruginosa strain 2875 chromosome, complete genome

```
product length = 312
Forward primer  1        GACTGGGTGGTGCTCGAAG  19
Template        2220609  ...................  2220627

Reverse primer  1        GCGCTCCTCGGCCTGTAG  18
Template        2220920  ..................  2220903
```

>CP116718.1 Pseudomonas aeruginosa strain 2858 chromosome, complete genome

```
product length = 312
Forward primer  1        GACTGGGTGGTGCTCGAAG  19
Template        5108286  ...................  5108268

Reverse primer  1        GCGCTCCTCGGCCTGTAG  18
Template        5107975  ..................  5107992
```

>CP116720.1 Pseudomonas aeruginosa strain 2866 chromosome, complete genome

```
product length = 312
Forward primer  1        GACTGGGTGGTGCTCGAAG  19
Template        2239557  ...................  2239575

Reverse primer  1        GCGCTCCTCGGCCTGTAG  18
Template        2239868  ..................  2239851
```

>CP116724.1 Pseudomonas aeruginosa strain 2880 chromosome, complete genome

```
product length = 312
Forward primer  1        GACTGGGTGGTGCTCGAAG  19
Template        2252832  ...................  2252850

Reverse primer  1        GCGCTCCTCGGCCTGTAG  18
Template        2253143  ..................  2253126
```

>CP116721.1 Pseudomonas aeruginosa strain 2867 chromosome, complete genome

```
product length = 312
Forward primer  1        GACTGGGTGGTGCTCGAAG  19
Template        2283915  ...................  2283933

Reverse primer  1        GCGCTCCTCGGCCTGTAG  18
Template        2284226  ..................  2284209
```

>CP116715.1 Pseudomonas aeruginosa strain 2856 chromosome, complete genome

```
product length = 312
Forward primer  1        GACTGGGTGGTGCTCGAAG  19
Template        2307611  ...................  2307629

Reverse primer  1        GCGCTCCTCGGCCTGTAG  18
Template        2307922  ..................  2307905
```

>CP106784.1 Pseudomonas aeruginosa strain NY5085 chromosome, complete genome

```
product length = 312
Forward primer  1        GACTGGGTGGTGCTCGAAG  19
Template        2149000  ...................  2149018

Reverse primer  1        GCGCTCCTCGGCCTGTAG  18
Template        2149311  ..................  2149294
```

>CP096913.1 Pseudomonas aeruginosa strain NY7610 chromosome, complete genome

```
product length = 312
Forward primer  1        GACTGGGTGGTGCTCGAAG  19
Template        2023631  ...................  2023649

Reverse primer  1        GCGCTCCTCGGCCTGTAG  18
Template        2023942  ..................  2023925
```

>CP096912.1 Pseudomonas aeruginosa strain NY7770 chromosome, complete genome

```
product length = 312
Forward primer  1        GACTGGGTGGTGCTCGAAG  19
Template        2058591  ...................  2058609

Reverse primer  1        GCGCTCCTCGGCCTGTAG  18
Template        2058902  ..................  2058885
```

>CP096909.1 Pseudomonas aeruginosa strain NY8688 chromosome, complete genome

```
product length = 312
Forward primer  1        GACTGGGTGGTGCTCGAAG  19
Template        4816093  ...................  4816075

Reverse primer  1        GCGCTCCTCGGCCTGTAG  18
Template        4815782  ..................  4815799
```

>CP096822.1 Pseudomonas aeruginosa strain NY8709 chromosome, complete genome

```
product length = 312
Forward primer  1        GACTGGGTGGTGCTCGAAG  19
Template        2095285  ...................  2095303

Reverse primer  1        GCGCTCCTCGGCCTGTAG  18
Template        2095596  ..................  2095579
```

>CP111030.1 Pseudomonas aeruginosa strain PALA38 chromosome, complete genome

```
product length = 312
Forward primer  1        GACTGGGTGGTGCTCGAAG  19
Template        2053569  ...................  2053587

Reverse primer  1        GCGCTCCTCGGCCTGTAG  18
Template        2053880  ..................  2053863
```

>CP111032.1 Pseudomonas aeruginosa strain PALA54 chromosome, complete genome

```
product length = 312
Forward primer  1        GACTGGGTGGTGCTCGAAG  19
Template        2054366  ...................  2054384

Reverse primer  1        GCGCTCCTCGGCCTGTAG  18
Template        2054677  ..................  2054660
```

>CP111034.1 Pseudomonas aeruginosa strain PALA50 chromosome, complete genome

```
product length = 312
Forward primer  1        GACTGGGTGGTGCTCGAAG  19
Template        2132170  ...................  2132188

Reverse primer  1        GCGCTCCTCGGCCTGTAG  18
Template        2132481  ..................  2132464
```

>CP110353.1 Pseudomonas aeruginosa strain PALA48 chromosome, complete genome

```
product length = 312
Forward primer  1        GACTGGGTGGTGCTCGAAG  19
Template        2161040  ...................  2161058

Reverse primer  1        GCGCTCCTCGGCCTGTAG  18
Template        2161351  ..................  2161334
```

>CP110352.1 Pseudomonas aeruginosa strain PALA47 chromosome, complete genome

```
product length = 312
Forward primer  1        GACTGGGTGGTGCTCGAAG  19
Template        2032602  ...................  2032620

Reverse primer  1        GCGCTCCTCGGCCTGTAG  18
Template        2032913  ..................  2032896
```

>CP110351.1 Pseudomonas aeruginosa strain PALA45 chromosome, complete genome

```
product length = 312
Forward primer  1        GACTGGGTGGTGCTCGAAG  19
Template        4075019  ...................  4075001

Reverse primer  1        GCGCTCCTCGGCCTGTAG  18
Template        4074708  ..................  4074725
```

>CP110350.1 Pseudomonas aeruginosa strain PALA44 chromosome, complete genome

```
product length = 312
Forward primer  1        GACTGGGTGGTGCTCGAAG  19
Template        3365084  ...................  3365102

Reverse primer  1        GCGCTCCTCGGCCTGTAG  18
Template        3365395  ..................  3365378
```

>CP109932.1 Pseudomonas aeruginosa strain PALA43 chromosome, complete genome

```
product length = 312
Forward primer  1        GACTGGGTGGTGCTCGAAG  19
Template        3162692  ...................  3162710

Reverse primer  1        GCGCTCCTCGGCCTGTAG  18
Template        3163003  ..................  3162986
```

>CP109931.1 Pseudomonas aeruginosa strain PALA42 chromosome, complete genome

```
product length = 312
Forward primer  1        GACTGGGTGGTGCTCGAAG  19
Template        4832916  ...................  4832898

Reverse primer  1        GCGCTCCTCGGCCTGTAG  18
Template        4832605  ..................  4832622
```

>CP110349.1 Pseudomonas aeruginosa strain PALA40 chromosome, complete genome

```
product length = 312
Forward primer  1        GACTGGGTGGTGCTCGAAG  19
Template        2269881  ...................  2269899

Reverse primer  1        GCGCTCCTCGGCCTGTAG  18
Template        2270192  ..................  2270175
```

>CP109920.1 Pseudomonas aeruginosa strain PALA39 chromosome, complete genome

```
product length = 312
Forward primer  1        GACTGGGTGGTGCTCGAAG  19
Template        2117961  ...................  2117979

Reverse primer  1        GCGCTCCTCGGCCTGTAG  18
Template        2118272  ..................  2118255
```

>CP110348.1 Pseudomonas aeruginosa strain PALA36 chromosome, complete genome

```
product length = 312
Forward primer  1        GACTGGGTGGTGCTCGAAG  19
Template        2268709  ...................  2268727

Reverse primer  1        GCGCTCCTCGGCCTGTAG  18
Template        2269020  ..................  2269003
```

>CP109919.1 Pseudomonas aeruginosa strain PALA56 chromosome, complete genome

```
product length = 312
Forward primer  1        GACTGGGTGGTGCTCGAAG  19
Template        2102061  ...................  2102079

Reverse primer  1        GCGCTCCTCGGCCTGTAG  18
Template        2102372  ..................  2102355
```

>CP109918.1 Pseudomonas aeruginosa strain PALA55 chromosome, complete genome

```
product length = 312
Forward primer  1        GACTGGGTGGTGCTCGAAG  19
Template        2081337  ...................  2081355

Reverse primer  1        GCGCTCCTCGGCCTGTAG  18
Template        2081648  ..................  2081631
```

>CP109856.1 Pseudomonas aeruginosa strain PALA53 chromosome, complete genome

```
product length = 312
Forward primer  1        GACTGGGTGGTGCTCGAAG  19
Template        2093991  ...................  2094009

Reverse primer  1        GCGCTCCTCGGCCTGTAG  18
Template        2094302  ..................  2094285
```

>CP109851.1 Pseudomonas aeruginosa strain PALA51 chromosome, complete genome

```
product length = 312
Forward primer  1        GACTGGGTGGTGCTCGAAG  19
Template        2075859  ...................  2075877

Reverse primer  1        GCGCTCCTCGGCCTGTAG  18
Template        2076170  ..................  2076153
```

>CP109850.1 Pseudomonas aeruginosa strain PALA37 chromosome, complete genome

```
product length = 312
Forward primer  1       GACTGGGTGGTGCTCGAAG  19
Template        266643  ...................  266625

Reverse primer  1       GCGCTCCTCGGCCTGTAG  18
Template        266332  ..................  266349
```

>CP110346.1 Pseudomonas aeruginosa strain PALA35 chromosome, complete genome

```
product length = 312
Forward primer  1        GACTGGGTGGTGCTCGAAG  19
Template        2180328  ...................  2180346

Reverse primer  1        GCGCTCCTCGGCCTGTAG  18
Template        2180639  ..................  2180622
```

>CP109849.1 Pseudomonas aeruginosa strain PALA34 chromosome, complete genome

```
product length = 312
Forward primer  1        GACTGGGTGGTGCTCGAAG  19
Template        2195042  ...................  2195060

Reverse primer  1        GCGCTCCTCGGCCTGTAG  18
Template        2195353  ..................  2195336
```

>CP109845.1 Pseudomonas aeruginosa strain PALA33 chromosome, complete genome

```
product length = 312
Forward primer  1        GACTGGGTGGTGCTCGAAG  19
Template        2202523  ...................  2202541

Reverse primer  1        GCGCTCCTCGGCCTGTAG  18
Template        2202834  ..................  2202817
```

>CP109844.1 Pseudomonas aeruginosa strain PALA32 chromosome, complete genome

```
product length = 312
Forward primer  1        GACTGGGTGGTGCTCGAAG  19
Template        2160868  ...................  2160886

Reverse primer  1        GCGCTCCTCGGCCTGTAG  18
Template        2161179  ..................  2161162
```

>CP110345.1 Pseudomonas aeruginosa strain PALA30 chromosome, complete genome

```
product length = 312
Forward primer  1        GACTGGGTGGTGCTCGAAG  19
Template        2133106  ...................  2133124

Reverse primer  1        GCGCTCCTCGGCCTGTAG  18
Template        2133417  ..................  2133400
```

>CP109843.1 Pseudomonas aeruginosa strain PALA29 chromosome, complete genome

```
product length = 312
Forward primer  1        GACTGGGTGGTGCTCGAAG  19
Template        2164927  ...................  2164945

Reverse primer  1        GCGCTCCTCGGCCTGTAG  18
Template        2165238  ..................  2165221
```

>CP109835.1 Pseudomonas aeruginosa strain PALA26 chromosome, complete genome

```
product length = 312
Forward primer  1        GACTGGGTGGTGCTCGAAG  19
Template        2159342  ...................  2159360

Reverse primer  1        GCGCTCCTCGGCCTGTAG  18
Template        2159653  ..................  2159636
```

>CP109834.1 Pseudomonas aeruginosa strain PALA25 chromosome, complete genome

```
product length = 312
Forward primer  1        GACTGGGTGGTGCTCGAAG  19
Template        2094330  ...................  2094348

Reverse primer  1        GCGCTCCTCGGCCTGTAG  18
Template        2094641  ..................  2094624
```

>CP110344.1 Pseudomonas aeruginosa strain PALA24 chromosome, complete genome

```
product length = 312
Forward primer  1        GACTGGGTGGTGCTCGAAG  19
Template        2081181  ...................  2081199

Reverse primer  1        GCGCTCCTCGGCCTGTAG  18
Template        2081492  ..................  2081475
```

>CP109833.1 Pseudomonas aeruginosa strain PALA23 chromosome, complete genome

```
product length = 312
Forward primer  1        GACTGGGTGGTGCTCGAAG  19
Template        2089404  ...................  2089422

Reverse primer  1        GCGCTCCTCGGCCTGTAG  18
Template        2089715  ..................  2089698
```

>CP107275.1 Pseudomonas aeruginosa strain PALA22 chromosome, complete genome

```
product length = 312
Forward primer  1        GACTGGGTGGTGCTCGAAG  19
Template        5304222  ...................  5304204

Reverse primer  1        GCGCTCCTCGGCCTGTAG  18
Template        5303911  ..................  5303928
```

>CP107064.1 Pseudomonas aeruginosa strain PALA20 chromosome, complete genome

```
product length = 312
Forward primer  1        GACTGGGTGGTGCTCGAAG  19
Template        6425745  ...................  6425727

Reverse primer  1        GCGCTCCTCGGCCTGTAG  18
Template        6425434  ..................  6425451
```

>CP107029.1 Pseudomonas aeruginosa strain PALA19 chromosome, complete genome

```
product length = 312
Forward primer  1        GACTGGGTGGTGCTCGAAG  19
Template        4931952  ...................  4931934

Reverse primer  1        GCGCTCCTCGGCCTGTAG  18
Template        4931641  ..................  4931658
```

>CP106745.1 Pseudomonas aeruginosa strain PALA17 chromosome, complete genome

```
product length = 312
Forward primer  1        GACTGGGTGGTGCTCGAAG  19
Template        1877074  ...................  1877092

Reverse primer  1        GCGCTCCTCGGCCTGTAG  18
Template        1877385  ..................  1877368
```

>CP106744.1 Pseudomonas aeruginosa strain PALA16 chromosome, complete genome

```
product length = 312
Forward primer  1        GACTGGGTGGTGCTCGAAG  19
Template        2070674  ...................  2070692

Reverse primer  1        GCGCTCCTCGGCCTGTAG  18
Template        2070985  ..................  2070968
```

>CP106743.1 Pseudomonas aeruginosa strain PALA15 chromosome, complete genome

```
product length = 312
Forward primer  1        GACTGGGTGGTGCTCGAAG  19
Template        2054163  ...................  2054181

Reverse primer  1        GCGCTCCTCGGCCTGTAG  18
Template        2054474  ..................  2054457
```

>CP106742.1 Pseudomonas aeruginosa strain PALA14 chromosome, complete genome

```
product length = 312
Forward primer  1        GACTGGGTGGTGCTCGAAG  19
Template        5336498  ...................  5336480

Reverse primer  1        GCGCTCCTCGGCCTGTAG  18
Template        5336187  ..................  5336204
```

>CP106682.1 Pseudomonas aeruginosa strain PALA13 chromosome, complete genome

```
product length = 312
Forward primer  1        GACTGGGTGGTGCTCGAAG  19
Template        4402383  ...................  4402365

Reverse primer  1        GCGCTCCTCGGCCTGTAG  18
Template        4402072  ..................  4402089
```

>CP106681.1 Pseudomonas aeruginosa strain PALA12 chromosome, complete genome

```
product length = 312
Forward primer  1        GACTGGGTGGTGCTCGAAG  19
Template        3465047  ...................  3465029

Reverse primer  1        GCGCTCCTCGGCCTGTAG  18
Template        3464736  ..................  3464753
```

>CP106680.1 Pseudomonas aeruginosa strain PALA11 chromosome, complete genome

```
product length = 312
Forward primer  1        GACTGGGTGGTGCTCGAAG  19
Template        2062156  ...................  2062174

Reverse primer  1        GCGCTCCTCGGCCTGTAG  18
Template        2062467  ..................  2062450
```

>CP104870.1 Pseudomonas aeruginosa strain PALA9 chromosome, complete genome

```
product length = 312
Forward primer  1        GACTGGGTGGTGCTCGAAG  19
Template        2198780  ...................  2198798

Reverse primer  1        GCGCTCCTCGGCCTGTAG  18
Template        2199091  ..................  2199074
```

>CP104869.1 Pseudomonas aeruginosa strain PALA8 chromosome, complete genome

```
product length = 312
Forward primer  1        GACTGGGTGGTGCTCGAAG  19
Template        2143304  ...................  2143322

Reverse primer  1        GCGCTCCTCGGCCTGTAG  18
Template        2143615  ..................  2143598
```

>CP104868.1 Pseudomonas aeruginosa strain PALA7 chromosome, complete genome

```
product length = 312
Forward primer  1        GACTGGGTGGTGCTCGAAG  19
Template        2032602  ...................  2032620

Reverse primer  1        GCGCTCCTCGGCCTGTAG  18
Template        2032913  ..................  2032896
```

>CP104867.1 Pseudomonas aeruginosa strain PALA6 chromosome, complete genome

```
product length = 312
Forward primer  1        GACTGGGTGGTGCTCGAAG  19
Template        3935814  ...................  3935796

Reverse primer  1        GCGCTCCTCGGCCTGTAG  18
Template        3935503  ..................  3935520
```

>CP104866.1 Pseudomonas aeruginosa strain PALA4 chromosome, complete genome

```
product length = 312
Forward primer  1        GACTGGGTGGTGCTCGAAG  19
Template        1989142  ...................  1989160

Reverse primer  1        GCGCTCCTCGGCCTGTAG  18
Template        1989453  ..................  1989436
```

>CP104865.1 Pseudomonas aeruginosa strain PALA2 chromosome, complete genome

```
product length = 312
Forward primer  1        GACTGGGTGGTGCTCGAAG  19
Template        2028937  ...................  2028955

Reverse primer  1        GCGCTCCTCGGCCTGTAG  18
Template        2029248  ..................  2029231
```

>CP104254.1 Pseudomonas aeruginosa strain PALA1 chromosome, complete genome

```
product length = 312
Forward primer  1        GACTGGGTGGTGCTCGAAG  19
Template        2116070  ...................  2116088

Reverse primer  1        GCGCTCCTCGGCCTGTAG  18
Template        2116381  ..................  2116364
```

>CP114761.1 Pseudomonas aeruginosa strain NF143349 chromosome, complete genome

```
product length = 312
Forward primer  1        GACTGGGTGGTGCTCGAAG  19
Template        2232370  ...................  2232388

Reverse primer  1        GCGCTCCTCGGCCTGTAG  18
Template        2232681  ..................  2232664
```

>CP114374.1 Pseudomonas aeruginosa strain Jade-X chromosome, complete genome

```
product length = 312
Forward primer  1        GACTGGGTGGTGCTCGAAG  19
Template        2040209  ...................  2040227

Reverse primer  1        GCGCTCCTCGGCCTGTAG  18
Template        2040520  ..................  2040503
```

>CP113974.1 Pseudomonas aeruginosa strain M6A146 chromosome, complete genome

```
product length = 312
Forward primer  1       GACTGGGTGGTGCTCGAAG  19
Template        441002  ...................  441020

Reverse primer  1       GCGCTCCTCGGCCTGTAG  18
Template        441313  ..................  441296
```

>CP097555.1 Pseudomonas aeruginosa strain B1.2 chromosome, complete genome

```
product length = 312
Forward primer  1        GACTGGGTGGTGCTCGAAG  19
Template        2088288  ...................  2088306

Reverse primer  1        GCGCTCCTCGGCCTGTAG  18
Template        2088599  ..................  2088582
```

>CP097556.1 Pseudomonas aeruginosa strain B2.1 chromosome, complete genome

```
product length = 312
Forward primer  1        GACTGGGTGGTGCTCGAAG  19
Template        2088288  ...................  2088306

Reverse primer  1        GCGCTCCTCGGCCTGTAG  18
Template        2088599  ..................  2088582
```

>CP113230.1 Pseudomonas aeruginosa strain BIAI 160 chromosome, complete genome

```
product length = 312
Forward primer  1        GACTGGGTGGTGCTCGAAG  19
Template        3871882  ...................  3871900

Reverse primer  1        GCGCTCCTCGGCCTGTAG  18
Template        3872193  ..................  3872176
```

>CP113246.1 Pseudomonas aeruginosa strain SMC4386 chromosome, complete genome

```
product length = 312
Forward primer  1        GACTGGGTGGTGCTCGAAG  19
Template        4208359  ...................  4208341

Reverse primer  1        GCGCTCCTCGGCCTGTAG  18
Template        4208048  ..................  4208065
```

>CP113106.1 Pseudomonas aeruginosa strain BIAI 157 chromosome, complete genome

```
product length = 312
Forward primer  1        GACTGGGTGGTGCTCGAAG  19
Template        1095783  ...................  1095801

Reverse primer  1        GCGCTCCTCGGCCTGTAG  18
Template        1096094  ..................  1096077
```

>CP097857.1 Pseudomonas sp. B111 chromosome, complete genome

```
product length = 312
Forward primer  1        GACTGGGTGGTGCTCGAAG  19
Template        1784302  ...................  1784284

Reverse primer  1        GCGCTCCTCGGCCTGTAG  18
Template        1783991  ..................  1784008
```

>CP102441.2 Pseudomonas aeruginosa strain PA30 chromosome, complete genome

```
product length = 312
Forward primer  1        GACTGGGTGGTGCTCGAAG  19
Template        4549614  ...................  4549596

Reverse primer  1        GCGCTCCTCGGCCTGTAG  18
Template        4549303  ..................  4549320
```

>CP036492.1 Pseudomonas aeruginosa strain Paer4 chromosome, complete genome

```
product length = 312
Forward primer  1        GACTGGGTGGTGCTCGAAG  19
Template        4195373  ...................  4195355

Reverse primer  1        GCGCTCCTCGGCCTGTAG  18
Template        4195062  ..................  4195079
```

>CP083357.1 Pseudomonas aeruginosa strain KPA143 chromosome, complete genome

```
product length = 312
Forward primer  1        GACTGGGTGGTGCTCGAAG  19
Template        5070857  ...................  5070875

Reverse primer  1        GCGCTCCTCGGCCTGTAG  18
Template        5071168  ..................  5071151
```

>CP083359.1 Pseudomonas aeruginosa strain KPA159 chromosome, complete genome

```
product length = 311
Forward primer  1        GACTGGGTGGTGCTCGAAG  19
Template        4907336  ...................  4907354

Reverse primer  1        GCGCTCCTCGGCCTGTAG  18
Template        4907646  ..................  4907629
```

>CP083358.1 Pseudomonas aeruginosa strain KPA151 chromosome, complete genome

```
product length = 312
Forward primer  1        GACTGGGTGGTGCTCGAAG  19
Template        5048910  ...................  5048892

Reverse primer  1        GCGCTCCTCGGCCTGTAG  18
Template        5048599  ..................  5048616
```

>CP083360.1 Pseudomonas aeruginosa strain KPA83 chromosome, complete genome

```
product length = 312
Forward primer  1        GACTGGGTGGTGCTCGAAG  19
Template        3346729  ...................  3346711

Reverse primer  1        GCGCTCCTCGGCCTGTAG  18
Template        3346418  ..................  3346435
```

>CP083355.1 Pseudomonas aeruginosa strain KPA134 chromosome, complete genome

```
product length = 310
Forward primer  1        GACTGGGTGGTGCTCGAAG  19
Template        3040551  ...................  3040569

Reverse primer  1        GCGCTCCTCGGCCTGTAG  18
Template        3040860  ..................  3040843
```

>CP083356.1 Pseudomonas aeruginosa strain KPA140 chromosome, complete genome

```
product length = 312
Forward primer  1        GACTGGGTGGTGCTCGAAG  19
Template        4948103  ...................  4948085

Reverse primer  1        GCGCTCCTCGGCCTGTAG  18
Template        4947792  ..................  4947809
```

>CP083353.1 Pseudomonas aeruginosa strain KPA120 chromosome, complete genome

```
product length = 314
Forward primer  1        GACTGGGTGGTGCTCGAAG  19
Template        4926513  ...................  4926531

Reverse primer  1        GCGCTCCTCGGCCTGTAG  18
Template        4926826  ..................  4926809
```

>CP083354.1 Pseudomonas aeruginosa strain KPA124 chromosome, complete genome

```
product length = 311
Forward primer  1        GACTGGGTGGTGCTCGAAG  19
Template        3099470  ...................  3099488

Reverse primer  1        GCGCTCCTCGGCCTGTAG  18
Template        3099780  ..................  3099763
```

>CP083352.1 Pseudomonas aeruginosa strain KPA119 chromosome, complete genome

```
product length = 312
Forward primer  1        GACTGGGTGGTGCTCGAAG  19
Template        4072307  ...................  4072289

Reverse primer  1        GCGCTCCTCGGCCTGTAG  18
Template        4071996  ..................  4072013
```

>CP104565.1 Pseudomonas aeruginosa strain HS_121 chromosome, complete genome

```
product length = 312
Forward primer  1        GACTGGGTGGTGCTCGAAG  19
Template        2057540  ...................  2057558

Reverse primer  1        GCGCTCCTCGGCCTGTAG  18
Template        2057851  ..................  2057834
```

>CP104567.1 Pseudomonas aeruginosa strain HS_13 chromosome, complete genome

```
product length = 312
Forward primer  1        GACTGGGTGGTGCTCGAAG  19
Template        2063666  ...................  2063684

Reverse primer  1        GCGCTCCTCGGCCTGTAG  18
Template        2063977  ..................  2063960
```

>CP107042.1 Pseudomonas aeruginosa strain GIMC5035:PA21/2013 chromosome

```
product length = 312
Forward primer  1       GACTGGGTGGTGCTCGAAG  19
Template        691564  ...................  691582

Reverse primer  1       GCGCTCCTCGGCCTGTAG  18
Template        691875  ..................  691858
```

>CP086213.1 Pseudomonas aeruginosa strain Pa3 chromosome, complete genome

```
product length = 312
Forward primer  1        GACTGGGTGGTGCTCGAAG  19
Template        2299405  ...................  2299423

Reverse primer  1        GCGCTCCTCGGCCTGTAG  18
Template        2299716  ..................  2299699
```

>CP104982.1 Pseudomonas aeruginosa PA14 isolate Gamma chromosome

```
product length = 312
Forward primer  1        GACTGGGTGGTGCTCGAAG  19
Template        2135663  ...................  2135681

Reverse primer  1        GCGCTCCTCGGCCTGTAG  18
Template        2135974  ..................  2135957
```

>CP104984.1 Pseudomonas aeruginosa PA14 isolate Alpha chromosome

```
product length = 312
Forward primer  1        GACTGGGTGGTGCTCGAAG  19
Template        2135663  ...................  2135681

Reverse primer  1        GCGCTCCTCGGCCTGTAG  18
Template        2135974  ..................  2135957
```

>CP104980.1 Pseudomonas aeruginosa PA14 isolate Epsilon chromosome

```
product length = 312
Forward primer  1        GACTGGGTGGTGCTCGAAG  19
Template        2135663  ...................  2135681

Reverse primer  1        GCGCTCCTCGGCCTGTAG  18
Template        2135974  ..................  2135957
```

>CP104985.1 Pseudomonas aeruginosa PA14 chromosome

```
product length = 312
Forward primer  1        GACTGGGTGGTGCTCGAAG  19
Template        2135663  ...................  2135681

Reverse primer  1        GCGCTCCTCGGCCTGTAG  18
Template        2135974  ..................  2135957
```

>CP104981.1 Pseudomonas aeruginosa PA14 isolate Delta chromosome

```
product length = 312
Forward primer  1        GACTGGGTGGTGCTCGAAG  19
Template        2135663  ...................  2135681

Reverse primer  1        GCGCTCCTCGGCCTGTAG  18
Template        2135974  ..................  2135957
```

>CP104983.1 Pseudomonas aeruginosa PA14 isolate Beta chromosome

```
product length = 312
Forward primer  1        GACTGGGTGGTGCTCGAAG  19
Template        2135663  ...................  2135681

Reverse primer  1        GCGCTCCTCGGCCTGTAG  18
Template        2135974  ..................  2135957
```

>CP104913.1 Pseudomonas aeruginosa strain PA-AUTBAM chromosome, complete genome

```
product length = 312
Forward primer  1        GACTGGGTGGTGCTCGAAG  19
Template        3433687  ...................  3433669

Reverse primer  1        GCGCTCCTCGGCCTGTAG  18
Template        3433376  ..................  3433393
```

>CP104696.1 Pseudomonas aeruginosa strain 2021CK-01281 plasmid unnamed1

```
product length = 312
Forward primer  1       GACTGGGTGGTGCTCGAAG  19
Template        572971  ...................  572989

Reverse primer  1       GCGCTCCTCGGCCTGTAG  18
Template        573282  ..................  573265
```

>CP104720.1 Pseudomonas aeruginosa strain NY4593 chromosome, complete genome

```
product length = 312
Forward primer  1        GACTGGGTGGTGCTCGAAG  19
Template        2043328  ...................  2043346

Reverse primer  1        GCGCTCCTCGGCCTGTAG  18
Template        2043639  ..................  2043622
```

>CP104590.1 Pseudomonas aeruginosa strain WTJH36 chromosome, complete genome

```
product length = 312
Forward primer  1        GACTGGGTGGTGCTCGAAG  19
Template        6591718  ...................  6591736

Reverse primer  1        GCGCTCCTCGGCCTGTAG  18
Template        6592029  ..................  6592012
```

>CP104588.1 Pseudomonas aeruginosa strain WTJH32 chromosome, complete genome

```
product length = 312
Forward primer  1        GACTGGGTGGTGCTCGAAG  19
Template        2129828  ...................  2129846

Reverse primer  1        GCGCTCCTCGGCCTGTAG  18
Template        2130139  ..................  2130122
```

>CP104586.1 Pseudomonas aeruginosa strain WTJH6 chromosome, complete genome

```
product length = 312
Forward primer  1        GACTGGGTGGTGCTCGAAG  19
Template        1036272  ...................  1036254

Reverse primer  1        GCGCTCCTCGGCCTGTAG  18
Template        1035961  ..................  1035978
```

>CP104584.1 Pseudomonas aeruginosa strain WTJH2 chromosome, complete genome

```
product length = 312
Forward primer  1        GACTGGGTGGTGCTCGAAG  19
Template        2129823  ...................  2129841

Reverse primer  1        GCGCTCCTCGGCCTGTAG  18
Template        2130134  ..................  2130117
```

>CP092972.1 Pseudomonas aeruginosa strain PA01135 chromosome, complete genome

```
product length = 312
Forward primer  1        GACTGGGTGGTGCTCGAAG  19
Template        4080473  ...................  4080455

Reverse primer  1        GCGCTCCTCGGCCTGTAG  18
Template        4080162  ..................  4080179
```

>CP079758.1 Pseudomonas aeruginosa strain PA0386 chromosome, complete genome

```
product length = 312
Forward primer  1        GACTGGGTGGTGCTCGAAG  19
Template        4074891  ...................  4074873

Reverse primer  1        GCGCTCCTCGGCCTGTAG  18
Template        4074580  ..................  4074597
```

>CP079757.1 Pseudomonas aeruginosa strain PA0200 chromosome, complete genome

```
product length = 312
Forward primer  1        GACTGGGTGGTGCTCGAAG  19
Template        3429375  ...................  3429357

Reverse primer  1        GCGCTCCTCGGCCTGTAG  18
Template        3429064  ..................  3429081
```

>CP104301.1 Pseudomonas aeruginosa strain PLL01 chromosome, complete genome

```
product length = 312
Forward primer  1        GACTGGGTGGTGCTCGAAG  19
Template        3434247  ...................  3434229

Reverse primer  1        GCGCTCCTCGGCCTGTAG  18
Template        3433936  ..................  3433953
```

>CP104170.1 Pseudomonas aeruginosa strain HW001G chromosome, complete genome

```
product length = 312
Forward primer  1        GACTGGGTGGTGCTCGAAG  19
Template        1402978  ...................  1402996

Reverse primer  1        GCGCTCCTCGGCCTGTAG  18
Template        1403289  ..................  1403272
```

>CP096207.1 Pseudomonas aeruginosa TBCF10839 chromosome, complete genome

```
product length = 312
Forward primer  1        GACTGGGTGGTGCTCGAAG  19
Template        3398676  ...................  3398658

Reverse primer  1        GCGCTCCTCGGCCTGTAG  18
Template        3398365  ..................  3398382
```

>CP079712.1 Pseudomonas aeruginosa strain PAO1-UW chromosome, complete genome

```
product length = 312
Forward primer  1        GACTGGGTGGTGCTCGAAG  19
Template        2081524  ...................  2081542

Reverse primer  1        GCGCTCCTCGGCCTGTAG  18
Template        2081835  ..................  2081818
```

>CP085082.1 Pseudomonas aeruginosa strain PAO1-Holloway chromosome, complete genome

```
product length = 312
Forward primer  1        GACTGGGTGGTGCTCGAAG  19
Template        3433499  ...................  3433481

Reverse primer  1        GCGCTCCTCGGCCTGTAG  18
Template        3433188  ..................  3433205
```

>CP101885.1 Pseudomonas aeruginosa strain M27432 chromosome, complete genome

```
product length = 312
Forward primer  1        GACTGGGTGGTGCTCGAAG  19
Template        4908487  ...................  4908469

Reverse primer  1        GCGCTCCTCGGCCTGTAG  18
Template        4908176  ..................  4908193
```

>CP094677.1 Pseudomonas aeruginosa strain Pa150 chromosome, complete genome

```
product length = 312
Forward primer  1       GACTGGGTGGTGCTCGAAG  19
Template        947552  ...................  947534

Reverse primer  1       GCGCTCCTCGGCCTGTAG  18
Template        947241  ..................  947258
```

>CP103307.1 Pseudomonas aeruginosa strain PLL01 chromosome, complete genome

```
product length = 312
Forward primer  1        GACTGGGTGGTGCTCGAAG  19
Template        3429894  ...................  3429876

Reverse primer  1        GCGCTCCTCGGCCTGTAG  18
Template        3429583  ..................  3429600
```

>CP102946.1 Pseudomonas aeruginosa strain SCAID WND1-2022 (148) chromosome, complete genome

```
product length = 312
Forward primer  1        GACTGGGTGGTGCTCGAAG  19
Template        2086766  ...................  2086784

Reverse primer  1        GCGCTCCTCGGCCTGTAG  18
Template        2087077  ..................  2087060
```

>CP102944.1 Pseudomonas aeruginosa strain SCAID TCT1-2022 (325) chromosome, complete genome

```
product length = 312
Forward primer  1        GACTGGGTGGTGCTCGAAG  19
Template        2185024  ...................  2185042

Reverse primer  1        GCGCTCCTCGGCCTGTAG  18
Template        2185335  ..................  2185318
```

>CP102174.1 Pseudomonas aeruginosa strain PA5083 chromosome, complete genome

```
product length = 312
Forward primer  1        GACTGGGTGGTGCTCGAAG  19
Template        2131576  ...................  2131594

Reverse primer  1        GCGCTCCTCGGCCTGTAG  18
Template        2131887  ..................  2131870
```

>CP101656.1 Pseudomonas aeruginosa strain L1a chromosome, complete genome

```
product length = 312
Forward primer  1        GACTGGGTGGTGCTCGAAG  19
Template        3082458  ...................  3082476
```

```
Reverse primer  1        GCGCTCCTCGGCCTGTAG  18
Template        3082769  ..................  3082752
```

>CP101912.1 Pseudomonas aeruginosa strain ATCC 27853 chromosome, complete genome

```
product length = 312
Forward primer  1        GACTGGGTGGTGCTCGAAG  19
Template        2027484  ...................  2027502

Reverse primer  1        GCGCTCCTCGGCCTGTAG  18
Template        2027795  ..................  2027778
```

>CP101911.1 Pseudomonas aeruginosa strain NWRC-1223 chromosome, complete genome

```
product length = 312
Forward primer  1        GACTGGGTGGTGCTCGAAG  19
Template        2069353  ...................  2069371

Reverse primer  1        GCGCTCCTCGGCCTGTAG  18
Template        2069664  ..................  2069647
```

>CP101540.1 Pseudomonas aeruginosa strain D-2 chromosome, complete genome

```
product length = 312
Forward primer  1        GACTGGGTGGTGCTCGAAG  19
Template        2045242  ...................  2045260

Reverse primer  1        GCGCTCCTCGGCCTGTAG  18
Template        2045553  ..................  2045536
```

>CP094851.1 Pseudomonas aeruginosa strain R20-14 chromosome, complete genome

```
product length = 312
Forward primer  1        GACTGGGTGGTGCTCGAAG  19
Template        2071110  ...................  2071128

Reverse primer  1        GCGCTCCTCGGCCTGTAG  18
Template        2071421  ..................  2071404
```

>CP100760.1 Pseudomonas aeruginosa strain AX0001 chromosome

```
product length = 312
Forward primer  1        GACTGGGTGGTGCTCGAAG  19
Template        3120373  ...................  3120391

Reverse primer  1        GCGCTCCTCGGCCTGTAG  18
Template        3120684  ..................  3120667
```

>CP100761.1 Pseudomonas aeruginosa strain PA0011 chromosome

```
product length = 312
Forward primer  1        GACTGGGTGGTGCTCGAAG  19
Template        3120373  ...................  3120391
```

```
Reverse primer  1        GCGCTCCTCGGCCTGTAG  18
Template        3120684  ..................  3120667
```

>CP097710.1 Pseudomonas aeruginosa strain PA-2 chromosome, complete genome

```
product length = 312
Forward primer  1        GACTGGGTGGTGCTCGAAG  19
Template        2155241  ...................  2155259

Reverse primer  1        GCGCTCCTCGGCCTGTAG  18
Template        2155552  ..................  2155535
```

>CP097709.1 Pseudomonas aeruginosa strain PA-1 chromosome, complete genome

```
product length = 312
Forward primer  1        GACTGGGTGGTGCTCGAAG  19
Template        2154138  ...................  2154156

Reverse primer  1        GCGCTCCTCGGCCTGTAG  18
Template        2154449  ..................  2154432
```

>CP100653.1 Pseudomonas aeruginosa strain F13 chromosome, complete genome

```
product length = 312
Forward primer  1        GACTGGGTGGTGCTCGAAG  19
Template        2139930  ...................  2139948

Reverse primer  1        GCGCTCCTCGGCCTGTAG  18
Template        2140241  ..................  2140224
```

>CP099798.1 Pseudomonas aeruginosa strain PAO1-L chromosome, complete genome

```
product length = 312
Forward primer  1        GACTGGGTGGTGCTCGAAG  19
Template        2082144  ...................  2082162

Reverse primer  1        GCGCTCCTCGGCCTGTAG  18
Template        2082455  ..................  2082438
```

>CP099797.1 Pseudomonas aeruginosa strain PAO1-N chromosome, complete genome

```
product length = 312
Forward primer  1        GACTGGGTGGTGCTCGAAG  19
Template        2082150  ...................  2082168

Reverse primer  1        GCGCTCCTCGGCCTGTAG  18
Template        2082461  ..................  2082444
```

>CP096665.1 Pseudomonas aeruginosa strain PAO1_Mat-X-1 chromosome, complete genome

```
product length = 312
Forward primer  1        GACTGGGTGGTGCTCGAAG  19
```

```
Template        3185070  ...................  3185052

Reverse primer  1        GCGCTCCTCGGCCTGTAG  18
Template        3184759  ..................  3184776
```

>CP096664.1 Pseudomonas aeruginosa strain PAO1_Kat-X-2 chromosome, complete genome

```
product length = 312
Forward primer  1        GACTGGGTGGTGCTCGAAG  19
Template        3086144  ...................  3086126

Reverse primer  1        GCGCTCCTCGGCCTGTAG  18
Template        3085833  ..................  3085850
```

>CP091880.1 Pseudomonas aeruginosa strain US449 chromosome, complete genome

```
product length = 312
Forward primer  1        GACTGGGTGGTGCTCGAAG  19
Template        2070331  ...................  2070349

Reverse primer  1        GCGCTCCTCGGCCTGTAG  18
Template        2070642  ..................  2070625
```

>CP069177.1 Pseudomonas aeruginosa strain Z154 chromosome, complete genome

```
product length = 312
Forward primer  1        GACTGGGTGGTGCTCGAAG  19
Template        5246509  ...................  5246491

Reverse primer  1        GCGCTCCTCGGCCTGTAG  18
Template        5246198  ..................  5246215
```

>CP097575.1 Pseudomonas aeruginosa strain UNC_PaerCF25 chromosome, complete genome

```
product length = 312
Forward primer  1        GACTGGGTGGTGCTCGAAG  19
Template        5392407  ...................  5392425

Reverse primer  1        GCGCTCCTCGGCCTGTAG  18
Template        5392718  ..................  5392701
```

>CP097383.1 Pseudomonas aeruginosa strain L00-a chromosome, complete genome

```
product length = 312
Forward primer  1        GACTGGGTGGTGCTCGAAG  19
Template        2190381  ...................  2190399

Reverse primer  1        GCGCTCCTCGGCCTGTAG  18
Template        2190692  ..................  2190675
```

>CP097256.1 Pseudomonas aeruginosa strain D5 chromosome, complete genome

```
product length = 312
```

```
Forward primer  1        GACTGGGTGGTGCTCGAAG  19
Template        2049077  ...................  2049095

Reverse primer  1        GCGCTCCTCGGCCTGTAG  18
Template        2049388  ..................  2049371
```

>CP096813.1 Pseudomonas aeruginosa strain 8D chromosome, complete genome

```
product length = 312
Forward primer  1        GACTGGGTGGTGCTCGAAG  19
Template        3501344  ...................  3501362

Reverse primer  1        GCGCTCCTCGGCCTGTAG  18
Template        3501655  ..................  3501638
```

>CP095772.2 Pseudomonas aeruginosa strain 34Pae23 chromosome

```
product length = 312
Forward primer  1        GACTGGGTGGTGCTCGAAG  19
Template        2146895  ...................  2146913

Reverse primer  1        GCGCTCCTCGGCCTGTAG  18
Template        2147206  ..................  2147189
```

>CP095923.1 Pseudomonas aeruginosa strain AR19438 chromosome, complete genome

```
product length = 312
Forward primer  1        GACTGGGTGGTGCTCGAAG  19
Template        2144012  ...................  2144030

Reverse primer  1        GCGCTCCTCGGCCTGTAG  18
Template        2144323  ..................  2144306
```

>CP095920.1 Pseudomonas aeruginosa strain AR19640 chromosome, complete genome

```
product length = 312
Forward primer  1        GACTGGGTGGTGCTCGAAG  19
Template        2041612  ...................  2041630

Reverse primer  1        GCGCTCCTCGGCCTGTAG  18
Template        2041923  ..................  2041906
```

>CP095922.1 Pseudomonas aeruginosa strain AR19583 chromosome, complete genome

```
product length = 312
Forward primer  1        GACTGGGTGGTGCTCGAAG  19
Template        2036779  ...................  2036797

Reverse primer  1        GCGCTCCTCGGCCTGTAG  18
Template        2037090  ..................  2037073
```

>CP095770.1 Pseudomonas aeruginosa strain 34Pae36 chromosome, complete genome

```
product length = 312
Forward primer  1        GACTGGGTGGTGCTCGAAG  19
Template        2399834  ...................  2399852

Reverse primer  1        GCGCTCCTCGGCCTGTAG  18
Template        2400145  ..................  2400128
```

>CP095774.1 Pseudomonas aeruginosa strain 34Pae8 chromosome, complete genome

```
product length = 312
Forward primer  1        GACTGGGTGGTGCTCGAAG  19
Template        4397421  ...................  4397403

Reverse primer  1        GCGCTCCTCGGCCTGTAG  18
Template        4397110  ..................  4397127
```

>CP090649.1 Pseudomonas aeruginosa strain PA1609 chromosome, complete genome

```
product length = 312
Forward primer  1        GACTGGGTGGTGCTCGAAG  19
Template        2185453  ...................  2185471

Reverse primer  1        GCGCTCCTCGGCCTGTAG  18
Template        2185764  ..................  2185747
```

>CP090648.1 Pseudomonas aeruginosa strain PA1616 chromosome, complete genome

```
product length = 312
Forward primer  1        GACTGGGTGGTGCTCGAAG  19
Template        4657258  ...................  4657276

Reverse primer  1        GCGCTCCTCGGCCTGTAG  18
Template        4657569  ..................  4657552
```

>CP090647.1 Pseudomonas aeruginosa strain PA1681 chromosome, complete genome

```
product length = 312
Forward primer  1        GACTGGGTGGTGCTCGAAG  19
Template        4112962  ...................  4112944

Reverse primer  1        GCGCTCCTCGGCCTGTAG  18
Template        4112651  ..................  4112668
```

>CP050149.1 Pseudomonas aeruginosa strain CHA chromosome

```
product length = 312
Forward primer  1        GACTGGGTGGTGCTCGAAG  19
Template        2066975  ...................  2066993

Reverse primer  1        GCGCTCCTCGGCCTGTAG  18
Template        2067286  ..................  2067269
```

>CP050148.1 Pseudomonas aeruginosa strain AA43 chromosome, complete genome

```
product length = 312
Forward primer  1        GACTGGGTGGTGCTCGAAG  19
Template        2051612  ...................  2051630

Reverse primer  1        GCGCTCCTCGGCCTGTAG  18
Template        2051923  ..................  2051906
```

>CP064391.1 Pseudomonas aeruginosa strain PartH-Paeruginosa-RM8376 chromosome, complete genome

```
product length = 312
Forward primer  1        GACTGGGTGGTGCTCGAAG  19
Template        1043104  ...................  1043122

Reverse primer  1        GCGCTCCTCGGCCTGTAG  18
Template        1043415  ..................  1043398
```

>CP063387.1 Pseudomonas aeruginosa strain ST1076_d100blood2 chromosome, complete genome

```
product length = 312
Forward primer  1        GACTGGGTGGTGCTCGAAG  19
Template        2112145  ...................  2112163

Reverse primer  1        GCGCTCCTCGGCCTGTAG  18
Template        2112456  ..................  2112439
```

>CP047643.1 Pseudomonas aeruginosa CI27 chromosome, complete genome

```
product length = 312
Forward primer  1        GACTGGGTGGTGCTCGAAG  19
Template        2236068  ...................  2236086

Reverse primer  1        GCGCTCCTCGGCCTGTAG  18
Template        2236379  ..................  2236362
```

>CP063396.1 Pseudomonas aeruginosa strain ST167_d26burn chromosome, complete genome

```
product length = 312
Forward primer  1        GACTGGGTGGTGCTCGAAG  19
Template        2115755  ...................  2115773

Reverse primer  1        GCGCTCCTCGGCCTGTAG  18
Template        2116066  ..................  2116049
```

>CP063395.1 Pseudomonas aeruginosa strain ST167_d57blood chromosome, complete genome

```
product length = 312
Forward primer  1        GACTGGGTGGTGCTCGAAG  19
Template        2115755  ...................  2115773

Reverse primer  1        GCGCTCCTCGGCCTGTAG  18
Template        2116066  ..................  2116049
```

>CP063394.1 Pseudomonas aeruginosa strain ST167_d67burn1 chromosome, complete genome

```
product length = 312
Forward primer  1        GACTGGGTGGTGCTCGAAG  19
Template        2116226  ...................  2116244

Reverse primer  1        GCGCTCCTCGGCCTGTAG  18
Template        2116537  ..................  2116520
```

>CP063393.1 Pseudomonas aeruginosa strain ST167_d67burn2 chromosome, complete genome

```
product length = 312
Forward primer  1        GACTGGGTGGTGCTCGAAG  19
Template        2115743  ...................  2115761

Reverse primer  1        GCGCTCCTCGGCCTGTAG  18
Template        2116054  ..................  2116037
```

>CP063392.1 Pseudomonas aeruginosa strain ST167_d68blood1 chromosome, complete genome

```
product length = 312
Forward primer  1        GACTGGGTGGTGCTCGAAG  19
Template        2115760  ...................  2115778

Reverse primer  1        GCGCTCCTCGGCCTGTAG  18
Template        2116071  ..................  2116054
```

>CP063391.1 Pseudomonas aeruginosa strain ST167_d68blood2 chromosome, complete genome

```
product length = 312
Forward primer  1        GACTGGGTGGTGCTCGAAG  19
Template        2115756  ...................  2115774

Reverse primer  1        GCGCTCCTCGGCCTGTAG  18
Template        2116067  ..................  2116050
```

>CP063390.1 Pseudomonas aeruginosa strain ST1076_d97burn1 chromosome, complete genome

```
product length = 312
Forward primer  1        GACTGGGTGGTGCTCGAAG  19
Template        2112145  ...................  2112163

Reverse primer  1        GCGCTCCTCGGCCTGTAG  18
Template        2112456  ..................  2112439
```

>CP063389.1 Pseudomonas aeruginosa strain ST1076_d97burn2 chromosome, complete genome

```
product length = 312
Forward primer  1        GACTGGGTGGTGCTCGAAG  19
Template        2112145  ...................  2112163

Reverse primer  1        GCGCTCCTCGGCCTGTAG  18
Template        2112456  ..................  2112439
```

>CP063388.1 Pseudomonas aeruginosa strain ST1076_d100blood1 chromosome, complete genome

```
product length = 312
Forward primer  1        GACTGGGTGGTGCTCGAAG  19
Template        2112145  ...................  2112163

Reverse primer  1        GCGCTCCTCGGCCTGTAG  18
Template        2112456  ..................  2112439
```

>CP063386.1 Pseudomonas aeruginosa strain ST1076_d118limb1 chromosome, complete genome

```
product length = 312
Forward primer  1        GACTGGGTGGTGCTCGAAG  19
Template        2104508  ...................  2104526

Reverse primer  1        GCGCTCCTCGGCCTGTAG  18
Template        2104819  ..................  2104802
```

>CP063385.1 Pseudomonas aeruginosa strain St1076_d123blood chromosome, complete genome

```
product length = 312
Forward primer  1        GACTGGGTGGTGCTCGAAG  19
Template        2112145  ...................  2112163

Reverse primer  1        GCGCTCCTCGGCCTGTAG  18
Template        2112456  ..................  2112439
```

>CP093967.1 Pseudomonas aeruginosa strain NY4605 chromosome, complete genome

```
product length = 312
Forward primer  1        GACTGGGTGGTGCTCGAAG  19
Template        2080398  ...................  2080416

Reverse primer  1        GCGCTCCTCGGCCTGTAG  18
Template        2080709  ..................  2080692
```

>CP093965.1 Pseudomonas aeruginosa strain ATCC BAA-2108 chromosome, complete genome

```
product length = 312
Forward primer  1        GACTGGGTGGTGCTCGAAG  19
Template        2121270  ...................  2121288

Reverse primer  1        GCGCTCCTCGGCCTGTAG  18
Template        2121581  ..................  2121564
```

>CP093966.1 Pseudomonas aeruginosa strain ATCC BAA-2114 chromosome, complete genome

```
product length = 312
Forward primer  1        GACTGGGTGGTGCTCGAAG  19
Template        2030560  ...................  2030578

Reverse primer  1        GCGCTCCTCGGCCTGTAG  18
Template        2030871  ..................  2030854
```

>CP093395.1 Pseudomonas aeruginosa strain PA1_NCHU chromosome, complete genome

```
product length = 312
Forward primer  1        GACTGGGTGGTGCTCGAAG  19
Template        2529663  ...................  2529645

Reverse primer  1        GCGCTCCTCGGCCTGTAG  18
Template        2529352  ..................  2529369
```

>CP093358.1 Pseudomonas aeruginosa strain E167 chromosome, complete genome

```
product length = 312
Forward primer  1        GACTGGGTGGTGCTCGAAG  19
Template        2082376  ...................  2082394

Reverse primer  1        GCGCTCCTCGGCCTGTAG  18
Template        2082687  ..................  2082670
```

>CP093356.1 Pseudomonas aeruginosa strain E125 chromosome, complete genome

```
product length = 312
Forward primer  1        GACTGGGTGGTGCTCGAAG  19
Template        2194483  ...................  2194501

Reverse primer  1        GCGCTCCTCGGCCTGTAG  18
Template        2194794  ..................  2194777
```

>CP093357.1 Pseudomonas aeruginosa strain E131 chromosome, complete genome

```
product length = 312
Forward primer  1        GACTGGGTGGTGCTCGAAG  19
Template        2082494  ...................  2082512

Reverse primer  1        GCGCTCCTCGGCCTGTAG  18
Template        2082805  ..................  2082788
```

>CP093355.1 Pseudomonas aeruginosa strain E104 chromosome, complete genome

```
product length = 312
Forward primer  1        GACTGGGTGGTGCTCGAAG  19
Template        2123020  ...................  2123038

Reverse primer  1        GCGCTCCTCGGCCTGTAG  18
Template        2123331  ..................  2123314
```

>CP093354.1 Pseudomonas aeruginosa strain E113 chromosome, complete genome

```
product length = 312
Forward primer  1        GACTGGGTGGTGCTCGAAG  19
Template        2231776  ...................  2231794

Reverse primer  1        GCGCTCCTCGGCCTGTAG  18
Template        2232087  ..................  2232070
```

>CP093030.1 Pseudomonas aeruginosa strain H04 chromosome, complete genome

```
product length = 312
Forward primer  1        GACTGGGTGGTGCTCGAAG  19
Template        2159803  ...................  2159821

Reverse primer  1        GCGCTCCTCGGCCTGTAG  18
Template        2160114  ..................  2160097
```

>CP093032.1 Pseudomonas aeruginosa strain H02 chromosome, complete genome

```
product length = 312
Forward primer  1        GACTGGGTGGTGCTCGAAG  19
Template        2135157  ...................  2135175

Reverse primer  1        GCGCTCCTCGGCCTGTAG  18
Template        2135468  ..................  2135451
```

>CP093031.1 Pseudomonas aeruginosa strain H03 chromosome, complete genome

```
product length = 312
Forward primer  1        GACTGGGTGGTGCTCGAAG  19
Template        2185689  ...................  2185707

Reverse primer  1        GCGCTCCTCGGCCTGTAG  18
Template        2186000  ..................  2185983
```

>CP093024.1 Pseudomonas aeruginosa strain H06 chromosome

```
product length = 312
Forward primer  1        GACTGGGTGGTGCTCGAAG  19
Template        1236487  ...................  1236469

Reverse primer  1        GCGCTCCTCGGCCTGTAG  18
Template        1236176  ..................  1236193
```

>CP093013.1 Pseudomonas aeruginosa strain H19 chromosome

```
product length = 312
Forward primer  1       GACTGGGTGGTGCTCGAAG  19
Template        633414  ...................  633396

Reverse primer  1       GCGCTCCTCGGCCTGTAG  18
Template        633103  ..................  633120
```

>CP093023.1 Pseudomonas aeruginosa strain H07 chromosome, complete genome

```
product length = 312
Forward primer  1        GACTGGGTGGTGCTCGAAG  19
Template        2055967  ...................  2055985

Reverse primer  1        GCGCTCCTCGGCCTGTAG  18
Template        2056278  ..................  2056261
```

>CP093018.1 Pseudomonas aeruginosa strain H11 chromosome

```
product length = 312
Forward primer  1        GACTGGGTGGTGCTCGAAG  19
Template        4948346  ...................  4948364

Reverse primer  1        GCGCTCCTCGGCCTGTAG  18
Template        4948657  ..................  4948640
```

>CP093016.1 Pseudomonas aeruginosa strain H15 chromosome, complete genome

```
product length = 312
Forward primer  1        GACTGGGTGGTGCTCGAAG  19
Template        6329881  ...................  6329863

Reverse primer  1        GCGCTCCTCGGCCTGTAG  18
Template        6329570  ..................  6329587
```

>CP093020.1 Pseudomonas aeruginosa strain H10 chromosome, complete genome

```
product length = 312
Forward primer  1        GACTGGGTGGTGCTCGAAG  19
Template        2122801  ...................  2122819

Reverse primer  1        GCGCTCCTCGGCCTGTAG  18
Template        2123112  ..................  2123095
```

>CP093012.1 Pseudomonas aeruginosa strain H20 chromosome, complete genome

```
product length = 312
Forward primer  1        GACTGGGTGGTGCTCGAAG  19
Template        2084697  ...................  2084715

Reverse primer  1        GCGCTCCTCGGCCTGTAG  18
Template        2085008  ..................  2084991
```

>CP093014.1 Pseudomonas aeruginosa strain H17 chromosome, complete genome

```
product length = 312
Forward primer  1        GACTGGGTGGTGCTCGAAG  19
Template        2088179  ...................  2088197

Reverse primer  1        GCGCTCCTCGGCCTGTAG  18
Template        2088490  ..................  2088473
```

>CP080405.1 Pseudomonas aeruginosa strain PES_P749 chromosome, complete genome

```
product length = 312
Forward primer  1        GACTGGGTGGTGCTCGAAG  19
Template        2054292  ...................  2054310

Reverse primer  1        GCGCTCCTCGGCCTGTAG  18
Template        2054603  ..................  2054586
```

>CP081148.1 Pseudomonas aeruginosa strain NDM1_2 chromosome

```
product length = 312
Forward primer  1        GACTGGGTGGTGCTCGAAG  19
Template        2142550  ...................  2142568

Reverse primer  1        GCGCTCCTCGGCCTGTAG  18
Template        2142861  ..................  2142844
```

>CP092634.1 Pseudomonas aeruginosa strain LS.2c chromosome, complete genome

```
product length = 312
Forward primer  1        GACTGGGTGGTGCTCGAAG  19
Template        2069926  ...................  2069944

Reverse primer  1        GCGCTCCTCGGCCTGTAG  18
Template        2070237  ..................  2070220
```

>CP092629.1 Pseudomonas aeruginosa strain HU20 chromosome, complete genome

```
product length = 312
Forward primer  1        GACTGGGTGGTGCTCGAAG  19
Template        5730695  ...................  5730677

Reverse primer  1        GCGCTCCTCGGCCTGTAG  18
Template        5730384  ..................  5730401
```

>CP092032.1 Pseudomonas aeruginosa strain ZS-PA-05 chromosome, complete genome

```
product length = 312
Forward primer  1        GACTGGGTGGTGCTCGAAG  19
Template        2394460  ...................  2394478

Reverse primer  1        GCGCTCCTCGGCCTGTAG  18
Template        2394771  ..................  2394754
```

>CP054794.1 Pseudomonas aeruginosa strain A0002 chromosome, complete genome

```
product length = 312
Forward primer  1        GACTGGGTGGTGCTCGAAG  19
Template        2187177  ...................  2187195

Reverse primer  1        GCGCTCCTCGGCCTGTAG  18
Template        2187488  ..................  2187471
```

>CP054793.1 Pseudomonas aeruginosa strain SE5452 chromosome, complete genome

```
product length = 312
Forward primer  1        GACTGGGTGGTGCTCGAAG  19
Template        2037071  ...................  2037089

Reverse primer  1        GCGCTCCTCGGCCTGTAG  18
Template        2037382  ..................  2037365
```

>CP054792.1 Pseudomonas aeruginosa strain SE5431 chromosome, complete genome

```
product length = 312
Forward primer  1        GACTGGGTGGTGCTCGAAG  19
Template        2057314  ...................  2057332

Reverse primer  1        GCGCTCCTCGGCCTGTAG  18
Template        2057625  ..................  2057608
```

>CP054791.1 Pseudomonas aeruginosa strain SE5430 chromosome, complete genome

```
product length = 312
Forward primer  1        GACTGGGTGGTGCTCGAAG  19
Template        2170239  ...................  2170257

Reverse primer  1        GCGCTCCTCGGCCTGTAG  18
Template        2170550  ..................  2170533
```

>CP054790.1 Pseudomonas aeruginosa strain SE5418 chromosome, complete genome

```
product length = 312
Forward primer  1        GACTGGGTGGTGCTCGAAG  19
Template        2193170  ...................  2193188

Reverse primer  1        GCGCTCCTCGGCCTGTAG  18
Template        2193481  ..................  2193464
```

>CP054789.1 Pseudomonas aeruginosa strain SE5381 chromosome, complete genome

```
product length = 312
Forward primer  1        GACTGGGTGGTGCTCGAAG  19
Template        2193155  ...................  2193173

Reverse primer  1        GCGCTCCTCGGCCTGTAG  18
Template        2193466  ..................  2193449
```

>CP054788.1 Pseudomonas aeruginosa strain YTSY4 chromosome, complete genome

```
product length = 312
Forward primer  1        GACTGGGTGGTGCTCGAAG  19
Template        1790528  ...................  1790510

Reverse primer  1        GCGCTCCTCGGCCTGTAG  18
Template        1790217  ..................  1790234
```

>CP054787.1 Pseudomonas aeruginosa strain HB2011305RE chromosome, complete genome

```
product length = 312
Forward primer  1        GACTGGGTGGTGCTCGAAG  19
Template        2178951  ...................  2178969

Reverse primer  1        GCGCTCCTCGGCCTGTAG  18
Template        2179262  ..................  2179245
```

>CP054786.1 Pseudomonas aeruginosa strain DL201330 chromosome, complete genome

```
product length = 312
Forward primer  1      GACTGGGTGGTGCTCGAAG  19
Template        58843  ...................  58861

Reverse primer  1      GCGCTCCTCGGCCTGTAG  18
Template        59154  ..................  59137
```

>CP086122.1 Pseudomonas aeruginosa strain MIN-155 chromosome, complete genome

```
product length = 312
Forward primer  1        GACTGGGTGGTGCTCGAAG  19
Template        2081582  ...................  2081600

Reverse primer  1        GCGCTCCTCGGCCTGTAG  18
Template        2081893  ..................  2081876
```

>CP081477.2 Pseudomonas aeruginosa strain P8W chromosome, complete genome

```
product length = 312
Forward primer  1        GACTGGGTGGTGCTCGAAG  19
Template        3210217  ...................  3210199

Reverse primer  1        GCGCTCCTCGGCCTGTAG  18
Template        3209906  ..................  3209923
```

>CP090348.1 Pseudomonas aeruginosa strain PA8329 chromosome, complete genome

```
product length = 312
Forward primer  1        GACTGGGTGGTGCTCGAAG  19
Template        2043732  ...................  2043750

Reverse primer  1        GCGCTCCTCGGCCTGTAG  18
Template        2044043  ..................  2044026
```

>CP053747.1 Pseudomonas aeruginosa strain Pae1255-NDM1 chromosome, complete genome

```
product length = 312
Forward primer  1        GACTGGGTGGTGCTCGAAG  19
Template        2188074  ...................  2188092

Reverse primer  1        GCGCTCCTCGGCCTGTAG  18
Template        2188385  ..................  2188368
```

>CP089063.2 Pseudomonas aeruginosa strain UNC_PaerCF37 chromosome, complete genome

```
product length = 312
Forward primer  1        GACTGGGTGGTGCTCGAAG  19
Template        1621745  ...................  1621763

Reverse primer  1        GCGCTCCTCGGCCTGTAG  18
Template        1622056  ..................  1622039
```

>CP089064.2 Pseudomonas aeruginosa strain UNC_PaerCF35 chromosome, complete genome

```
product length = 312
Forward primer  1        GACTGGGTGGTGCTCGAAG  19
Template        4912703  ...................  4912721

Reverse primer  1        GCGCTCCTCGGCCTGTAG  18
Template        4913014  ..................  4912997
```

>CP089062.2 Pseudomonas aeruginosa strain UNC_PaerCF38 chromosome, complete genome

```
product length = 312
Forward primer  1        GACTGGGTGGTGCTCGAAG  19
Template        3317122  ...................  3317140

Reverse primer  1        GCGCTCCTCGGCCTGTAG  18
Template        3317433  ..................  3317416
```

>CP089061.2 Pseudomonas aeruginosa strain UNC_PaerCF41 chromosome, complete genome

```
product length = 312
Forward primer  1        GACTGGGTGGTGCTCGAAG  19
Template        4983473  ...................  4983491

Reverse primer  1        GCGCTCCTCGGCCTGTAG  18
Template        4983784  ..................  4983767
```

>CP089849.1 Pseudomonas aeruginosa strain PA0523 chromosome, complete genome

```
product length = 312
Forward primer  1        GACTGGGTGGTGCTCGAAG  19
Template        4229532  ...................  4229514

Reverse primer  1        GCGCTCCTCGGCCTGTAG  18
Template        4229221  ..................  4229238
```

>CP089745.1 Pseudomonas aeruginosa strain Pa608 chromosome, complete genome

```
product length = 312
Forward primer  1        GACTGGGTGGTGCTCGAAG  19
Template        2040725  ...................  2040743

Reverse primer  1        GCGCTCCTCGGCCTGTAG  18
Template        2041036  ..................  2041019
```

>CP089236.1 Pseudomonas aeruginosa strain JNQH-PA027 chromosome, complete genome

```
product length = 312
Forward primer  1        GACTGGGTGGTGCTCGAAG  19
Template        2263509  ...................  2263527

Reverse primer  1        GCGCTCCTCGGCCTGTAG  18
Template        2263820  ..................  2263803
```

>CP087675.1 Pseudomonas aeruginosa strain P93127 chromosome, complete genome

```
product length = 312
Forward primer  1        GACTGGGTGGTGCTCGAAG  19
Template        2243321  ...................  2243339

Reverse primer  1        GCGCTCCTCGGCCTGTAG  18
Template        2243632  ..................  2243615
```

>CP087674.1 Pseudomonas aeruginosa strain P4970C chromosome, complete genome

```
product length = 312
Forward primer  1        GACTGGGTGGTGCTCGAAG  19
Template        2243315  ...................  2243333

Reverse primer  1        GCGCTCCTCGGCCTGTAG  18
Template        2243626  ..................  2243609
```

>CP087673.1 Pseudomonas aeruginosa strain P96131 chromosome, complete genome

```
product length = 312
Forward primer  1        GACTGGGTGGTGCTCGAAG  19
Template        2160912  ...................  2160930

Reverse primer  1        GCGCTCCTCGGCCTGTAG  18
Template        2161223  ..................  2161206
```

>CP080369.1 Pseudomonas aeruginosa SG17M chromosome, complete genome

```
product length = 312
Forward primer  1        GACTGGGTGGTGCTCGAAG  19
Template        2377843  ...................  2377861

Reverse primer  1        GCGCTCCTCGGCCTGTAG  18
Template        2378154  ..................  2378137
```

>CP086064.1 Pseudomonas aeruginosa strain CCBH28525 chromosome, complete genome

```
product length = 312
Forward primer  1        GACTGGGTGGTGCTCGAAG  19
Template        4609687  ...................  4609669

Reverse primer  1        GCGCTCCTCGGCCTGTAG  18
Template        4609376  ..................  4609393
```

>CP086010.1 Pseudomonas aeruginosa isolate KB-PA_F19 chromosome, complete genome

```
product length = 312
Forward primer  1        GACTGGGTGGTGCTCGAAG  19
Template        2162315  ...................  2162333

Reverse primer  1        GCGCTCCTCGGCCTGTAG  18
Template        2162626  ..................  2162609
```

>CP082821.1 Pseudomonas aeruginosa strain SCAID PLC1-2021 (16/222) chromosome, complete genome

```
product length = 312
Forward primer  1        GACTGGGTGGTGCTCGAAG  19
Template        2154148  ...................  2154166

Reverse primer  1        GCGCTCCTCGGCCTGTAG  18
Template        2154459  ..................  2154442
```

>CP082822.1 Pseudomonas aeruginosa strain SCAID WND1-2021 (9/195) chromosome, complete genome

```
product length = 312
Forward primer  1        GACTGGGTGGTGCTCGAAG  19
Template        2206784  ...................  2206802

Reverse primer  1        GCGCTCCTCGGCCTGTAG  18
Template        2207095  ..................  2207078
```

>CP082823.1 Pseudomonas aeruginosa strain SCAID TST-2021 (7/157) chromosome, complete genome

```
product length = 312
Forward primer  1        GACTGGGTGGTGCTCGAAG  19
Template        2150751  ...................  2150769

Reverse primer  1        GCGCTCCTCGGCCTGTAG  18
Template        2151062  ..................  2151045
```

>CP078009.1 Pseudomonas aeruginosa strain FAHZU31 chromosome, complete genome

```
product length = 312
Forward primer  1        GACTGGGTGGTGCTCGAAG  19
Template        2082075  ...................  2082093

Reverse primer  1        GCGCTCCTCGGCCTGTAG  18
Template        2082386  ..................  2082369
```

>CP078007.1 Pseudomonas aeruginosa strain FAHZU40 chromosome, complete genome

```
product length = 312
Forward primer  1        GACTGGGTGGTGCTCGAAG  19
Template        2114154  ...................  2114172

Reverse primer  1        GCGCTCCTCGGCCTGTAG  18
Template        2114465  ..................  2114448
```

>CP078006.1 Pseudomonas aeruginosa strain NDTH7329 chromosome, complete genome

```
product length = 312
Forward primer  1        GACTGGGTGGTGCTCGAAG  19
Template        4187107  ...................  4187089
```

```
Reverse primer  1        GCGCTCCTCGGCCTGTAG  18
Template        4186796  ..................  4186813
```

>CP078004.1 Pseudomonas aeruginosa strain QZPH16 chromosome, complete genome

```
product length = 312
Forward primer  1        GACTGGGTGGTGCTCGAAG  19
Template        2162378  ...................  2162396

Reverse primer  1        GCGCTCCTCGGCCTGTAG  18
Template        2162689  ..................  2162672
```

>CP078002.1 Pseudomonas aeruginosa strain QZPH21 chromosome, complete genome

```
product length = 312
Forward primer  1        GACTGGGTGGTGCTCGAAG  19
Template        2162377  ...................  2162395

Reverse primer  1        GCGCTCCTCGGCCTGTAG  18
Template        2162688  ..................  2162671
```

>CP077999.1 Pseudomonas aeruginosa strain SRRSH1120 chromosome, complete genome

```
product length = 312
Forward primer  1        GACTGGGTGGTGCTCGAAG  19
Template        2122796  ...................  2122814

Reverse primer  1        GCGCTCCTCGGCCTGTAG  18
Template        2123107  ..................  2123090
```

>CP077997.1 Pseudomonas aeruginosa strain SRRSH1521 chromosome, complete genome

```
product length = 312
Forward primer  1        GACTGGGTGGTGCTCGAAG  19
Template        2082067  ...................  2082085

Reverse primer  1        GCGCTCCTCGGCCTGTAG  18
Template        2082378  ..................  2082361
```

>CP077994.1 Pseudomonas aeruginosa strain SRRSH2790 chromosome, complete genome

```
product length = 312
Forward primer  1        GACTGGGTGGTGCTCGAAG  19
Template        2162384  ...................  2162402

Reverse primer  1        GCGCTCCTCGGCCTGTAG  18
Template        2162695  ..................  2162678
```

>CP077988.1 Pseudomonas aeruginosa strain ZPPH1 chromosome, complete genome

```
product length = 312
Forward primer  1        GACTGGGTGGTGCTCGAAG  19
```

```
Template        2167846  ...................  2167864

Reverse primer  1        GCGCTCCTCGGCCTGTAG  18
Template        2168157  ..................  2168140
```

>CP077985.1 Pseudomonas aeruginosa strain ZPPH2 chromosome, complete genome

```
product length = 312
Forward primer  1        GACTGGGTGGTGCTCGAAG  19
Template        2116949  ...................  2116967

Reverse primer  1        GCGCTCCTCGGCCTGTAG  18
Template        2117260  ..................  2117243
```

>CP077981.1 Pseudomonas aeruginosa strain ZPPH14 chromosome, complete genome

```
product length = 312
Forward primer  1        GACTGGGTGGTGCTCGAAG  19
Template        2080491  ...................  2080509

Reverse primer  1        GCGCTCCTCGGCCTGTAG  18
Template        2080802  ..................  2080785
```

>CP077977.1 Pseudomonas aeruginosa strain ZPPH29 chromosome, complete genome

```
product length = 312
Forward primer  1        GACTGGGTGGTGCTCGAAG  19
Template        2185794  ...................  2185812

Reverse primer  1        GCGCTCCTCGGCCTGTAG  18
Template        2186105  ..................  2186088
```

>CP077971.1 Pseudomonas aeruginosa strain ZPPH33 chromosome, complete genome

```
product length = 312
Forward primer  1        GACTGGGTGGTGCTCGAAG  19
Template        2094187  ...................  2094205

Reverse primer  1        GCGCTCCTCGGCCTGTAG  18
Template        2094498  ..................  2094481
```

>CP064403.1 Pseudomonas aeruginosa strain WTJH12 chromosome, complete genome

```
product length = 312
Forward primer  1        GACTGGGTGGTGCTCGAAG  19
Template        2095384  ...................  2095402

Reverse primer  1        GCGCTCCTCGGCCTGTAG  18
Template        2095695  ..................  2095678
```

>CP064401.1 Pseudomonas aeruginosa strain NDTH10366 chromosome, complete genome

```
product length = 312
```

```
Forward primer  1        GACTGGGTGGTGCTCGAAG  19
Template        2136338  ...................  2136356

Reverse primer  1        GCGCTCCTCGGCCTGTAG  18
Template        2136649  ..................  2136632
```

>CP064399.1 Pseudomonas aeruginosa strain QZPH41 chromosome, complete genome

```
product length = 312
Forward primer  1        GACTGGGTGGTGCTCGAAG  19
Template        2109774  ...................  2109792

Reverse primer  1        GCGCTCCTCGGCCTGTAG  18
Template        2110085  ..................  2110068
```

>CP064397.1 Pseudomonas aeruginosa strain SRRSH1002 chromosome, complete genome

```
product length = 312
Forward primer  1        GACTGGGTGGTGCTCGAAG  19
Template        2127182  ...................  2127200

Reverse primer  1        GCGCTCCTCGGCCTGTAG  18
Template        2127493  ..................  2127476
```

>CP064395.1 Pseudomonas aeruginosa strain SRRSH1408 chromosome, complete genome

```
product length = 312
Forward primer  1        GACTGGGTGGTGCTCGAAG  19
Template        2125774  ...................  2125792

Reverse primer  1        GCGCTCCTCGGCCTGTAG  18
Template        2126085  ..................  2126068
```

>CP064393.1 Pseudomonas aeruginosa strain SRRSH1101 chromosome, complete genome

```
product length = 312
Forward primer  1        GACTGGGTGGTGCTCGAAG  19
Template        2125786  ...................  2125804

Reverse primer  1        GCGCTCCTCGGCCTGTAG  18
Template        2126097  ..................  2126080
```

>CP064392.1 Pseudomonas aeruginosa strain SRRSH15 chromosome, complete genome

```
product length = 312
Forward primer  1        GACTGGGTGGTGCTCGAAG  19
Template        2082073  ...................  2082091

Reverse primer  1        GCGCTCCTCGGCCTGTAG  18
Template        2082384  ..................  2082367
```

>CP080518.1 Pseudomonas aeruginosa strain YY322 chromosome, complete genome

```
product length = 312
Forward primer  1        GACTGGGTGGTGCTCGAAG  19
Template        2069373  ...................  2069391

Reverse primer  1        GCGCTCCTCGGCCTGTAG  18
Template        2069684  ..................  2069667
```

>CP080511.1 Pseudomonas aeruginosa strain DJ06 chromosome, complete genome

```
product length = 312
Forward primer  1        GACTGGGTGGTGCTCGAAG  19
Template        5036715  ...................  5036697

Reverse primer  1        GCGCTCCTCGGCCTGTAG  18
Template        5036404  ..................  5036421
```

>CP071947.1 Pseudomonas aeruginosa strain 2020HL-00861 chromosome, complete genome

```
product length = 312
Forward primer  1        GACTGGGTGGTGCTCGAAG  19
Template        3554531  ...................  3554549

Reverse primer  1        GCGCTCCTCGGCCTGTAG  18
Template        3554842  ..................  3554825
```

>CP080289.1 Pseudomonas aeruginosa strain PA2207 chromosome, complete genome

```
product length = 312
Forward primer  1        GACTGGGTGGTGCTCGAAG  19
Template        4544266  ...................  4544248

Reverse primer  1        GCGCTCCTCGGCCTGTAG  18
Template        4543955  ..................  4543972
```

>CP080287.1 Pseudomonas aeruginosa strain UNC_PaerCF11 chromosome, complete genome

```
product length = 312
Forward primer  1        GACTGGGTGGTGCTCGAAG  19
Template        2273692  ...................  2273674

Reverse primer  1        GCGCTCCTCGGCCTGTAG  18
Template        2273381  ..................  2273398
```

>CP080288.1 Pseudomonas aeruginosa strain UNC_PaerCF05 chromosome, complete genome

```
product length = 312
Forward primer  1        GACTGGGTGGTGCTCGAAG  19
Template        4853268  ...................  4853250

Reverse primer  1        GCGCTCCTCGGCCTGTAG  18
Template        4852957  ..................  4852974
```

>CP080282.1 Pseudomonas aeruginosa strain UNC_PaerCF16 chromosome, complete genome

```
product length = 312
Forward primer  1        GACTGGGTGGTGCTCGAAG  19
Template        3602631  ...................  3602649

Reverse primer  1        GCGCTCCTCGGCCTGTAG  18
Template        3602942  ..................  3602925
```

>CP080285.1 Pseudomonas aeruginosa strain UNC_PaerCF14 chromosome, complete genome

```
product length = 312
Forward primer  1        GACTGGGTGGTGCTCGAAG  19
Template        2999523  ...................  2999541

Reverse primer  1        GCGCTCCTCGGCCTGTAG  18
Template        2999834  ..................  2999817
```

>CP080280.1 Pseudomonas aeruginosa strain UNC_PaerCF20 chromosome, complete genome

```
product length = 312
Forward primer  1        GACTGGGTGGTGCTCGAAG  19
Template        4440580  ...................  4440562

Reverse primer  1        GCGCTCCTCGGCCTGTAG  18
Template        4440269  ..................  4440286
```

>CP080281.1 Pseudomonas aeruginosa strain UNC_PaerCF17 chromosome, complete genome

```
product length = 312
Forward primer  1        GACTGGGTGGTGCTCGAAG  19
Template        3524363  ...................  3524381

Reverse primer  1        GCGCTCCTCGGCCTGTAG  18
Template        3524674  ..................  3524657
```

>CP080011.1 Pseudomonas aeruginosa strain TL3773 chromosome, complete genome

```
product length = 312
Forward primer  1        GACTGGGTGGTGCTCGAAG  19
Template        1360722  ...................  1360704

Reverse primer  1        GCGCTCCTCGGCCTGTAG  18
Template        1360411  ..................  1360428
```

>CP080007.1 Pseudomonas aeruginosa strain S-1 chromosome, complete genome

```
product length = 312
Forward primer  1        GACTGGGTGGTGCTCGAAG  19
Template        2092618  ...................  2092636

Reverse primer  1        GCGCTCCTCGGCCTGTAG  18
Template        2092929  ..................  2092912
```

>CP061376.1 Pseudomonas aeruginosa strain HS17-127 chromosome, complete genome

```
product length = 312
Forward primer  1        GACTGGGTGGTGCTCGAAG  19
Template        2143314  ...................  2143332

Reverse primer  1        GCGCTCCTCGGCCTGTAG  18
Template        2143625  ..................  2143608
```

>CP078564.1 Pseudomonas aeruginosa strain Colony464 chromosome

```
product length = 312
Forward primer  1        GACTGGGTGGTGCTCGAAG  19
Template        2775425  ...................  2775407

Reverse primer  1        GCGCTCCTCGGCCTGTAG  18
Template        2775114  ..................  2775131
```

>CP053390.1 Pseudomonas aeruginosa strain TL1285 chromosome, complete genome

```
product length = 312
Forward primer  1       GACTGGGTGGTGCTCGAAG  19
Template        448694  ...................  448676

Reverse primer  1       GCGCTCCTCGGCCTGTAG  18
Template        448383  ..................  448400
```

>CP075176.1 Pseudomonas aeruginosa strain PA790 chromosome, complete genome

```
product length = 312
Forward primer  1        GACTGGGTGGTGCTCGAAG  19
Template        2089458  ...................  2089476

Reverse primer  1        GCGCTCCTCGGCCTGTAG  18
Template        2089769  ..................  2089752
```

>CP065948.1 Pseudomonas aeruginosa strain PAM68 chromosome, complete genome

```
product length = 312
Forward primer  1       GACTGGGTGGTGCTCGAAG  19
Template        842500  ...................  842518

Reverse primer  1       GCGCTCCTCGGCCTGTAG  18
Template        842811  ..................  842794
```

>CP065947.1 Pseudomonas aeruginosa strain PAS6 chromosome, complete genome

```
product length = 312
Forward primer  1        GACTGGGTGGTGCTCGAAG  19
Template        6703598  ...................  6703580

Reverse primer  1        GCGCTCCTCGGCCTGTAG  18
Template        6703287  ..................  6703304
```

>CP065374.1 Pseudomonas aeruginosa strain PAG7 chromosome, complete genome

```
product length = 312
Forward primer  1        GACTGGGTGGTGCTCGAAG  19
Template        5667800  ...................  5667818

Reverse primer  1        GCGCTCCTCGGCCTGTAG  18
Template        5668111  ..................  5668094
```

>CP073080.1 Pseudomonas aeruginosa strain NDTH9845 chromosome, complete genome

```
product length = 312
Forward primer  1        GACTGGGTGGTGCTCGAAG  19
Template        2130258  ...................  2130276

Reverse primer  1        GCGCTCCTCGGCCTGTAG  18
Template        2130569  ..................  2130552
```

>CP073082.1 Pseudomonas aeruginosa strain WTJH17 chromosome, complete genome

```
product length = 312
Forward primer  1        GACTGGGTGGTGCTCGAAG  19
Template        2066785  ...................  2066803

Reverse primer  1        GCGCTCCTCGGCCTGTAG  18
Template        2067096  ..................  2067079
```

>CP071731.1 Pseudomonas aeruginosa strain LYSZa2 chromosome, complete genome

```
product length = 312
Forward primer  1        GACTGGGTGGTGCTCGAAG  19
Template        4722786  ...................  4722804

Reverse primer  1        GCGCTCCTCGGCCTGTAG  18
Template        4723097  ..................  4723080
```

>CP071730.1 Pseudomonas aeruginosa strain LYSZa5 chromosome, complete genome

```
product length = 312
Forward primer  1        GACTGGGTGGTGCTCGAAG  19
Template        4722796  ...................  4722814

Reverse primer  1        GCGCTCCTCGGCCTGTAG  18
Template        4723107  ..................  4723090
```

>AP024513.1 Pseudomonas aeruginosa Pa12 DNA, complete genome

```
product length = 312
Forward primer  1        GACTGGGTGGTGCTCGAAG  19
Template        2116292  ...................  2116310

Reverse primer  1        GCGCTCCTCGGCCTGTAG  18
Template        2116603  ..................  2116586
```

>CP024024.1 Pseudomonas aeruginosa strain PARM801 chromosome, complete genome

```
product length = 312
Forward primer  1        GACTGGGTGGTGCTCGAAG  19
Template        7033128  ...................  7033146

Reverse primer  1        GCGCTCCTCGGCCTGTAG  18
Template        7033439  ..................  7033422
```

>CP069198.1 Pseudomonas aeruginosa strain 152962 chromosome, complete genome

```
product length = 312
Forward primer  1        GACTGGGTGGTGCTCGAAG  19
Template        3188867  ...................  3188849

Reverse primer  1        GCGCTCCTCGGCCTGTAG  18
Template        3188556  ..................  3188573
```

>CP060392.1 Pseudomonas aeruginosa strain 1903031130 chromosome, complete genome

```
product length = 312
Forward primer  1        GACTGGGTGGTGCTCGAAG  19
Template        2137845  ...................  2137863

Reverse primer  1        GCGCTCCTCGGCCTGTAG  18
Template        2138156  ..................  2138139
```

>CP054845.1 Pseudomonas aeruginosa strain SE5429 chromosome, complete genome

```
product length = 312
Forward primer  1        GACTGGGTGGTGCTCGAAG  19
Template        2546487  ...................  2546505

Reverse primer  1        GCGCTCCTCGGCCTGTAG  18
Template        2546798  ..................  2546781
```

>CP054844.1 Pseudomonas aeruginosa strain SE5357 chromosome, complete genome

```
product length = 312
Forward primer  1        GACTGGGTGGTGCTCGAAG  19
Template        2185091  ...................  2185109

Reverse primer  1        GCGCTCCTCGGCCTGTAG  18
Template        2185402  ..................  2185385
```

>CP054843.1 Pseudomonas aeruginosa strain SE5352 chromosome, complete genome

```
product length = 312
Forward primer  1        GACTGGGTGGTGCTCGAAG  19
Template        2137884  ...................  2137902

Reverse primer  1        GCGCTCCTCGGCCTGTAG  18
Template        2138195  ..................  2138178
```

>CP054581.1 Pseudomonas aeruginosa strain YTSEY8 chromosome, complete genome

```
product length = 312
Forward primer  1        GACTGGGTGGTGCTCGAAG  19
Template        2249287  ...................  2249305

Reverse primer  1        GCGCTCCTCGGCCTGTAG  18
Template        2249598  ..................  2249581
```

>CP068239.1 Pseudomonas aeruginosa strain PA19-3047 chromosome, complete genome

```
product length = 312
Forward primer  1        GACTGGGTGGTGCTCGAAG  19
Template        4255072  ...................  4255054

Reverse primer  1        GCGCTCCTCGGCCTGTAG  18
Template        4254761  ..................  4254778
```

>CP061699.1 Pseudomonas aeruginosa strain LYSZa7 chromosome, complete genome

```
product length = 312
Forward primer  1        GACTGGGTGGTGCTCGAAG  19
Template        4655585  ...................  4655567

Reverse primer  1        GCGCTCCTCGGCCTGTAG  18
Template        4655274  ..................  4655291
```

>CP054623.1 Pseudomonas aeruginosa strain DL201330 chromosome, complete genome

```
product length = 312
Forward primer  1        GACTGGGTGGTGCTCGAAG  19
Template        2143184  ...................  2143202

Reverse primer  1        GCGCTCCTCGGCCTGTAG  18
Template        2143495  ..................  2143478
```

>CP065966.1 Pseudomonas aeruginosa strain FDAARGOS_1041 chromosome, complete genome

```
product length = 312
Forward primer  1        GACTGGGTGGTGCTCGAAG  19
Template        5616009  ...................  5616027

Reverse primer  1        GCGCTCCTCGGCCTGTAG  18
Template        5616320  ..................  5616303
```

>CP027857.1 Pseudomonas aeruginosa strain MPAO1 chromosome, complete genome

```
product length = 312
Forward primer  1        GACTGGGTGGTGCTCGAAG  19
Template        2081662  ...................  2081680

Reverse primer  1        GCGCTCCTCGGCCTGTAG  18
Template        2081973  ..................  2081956
```

>CP065867.1 Pseudomonas aeruginosa strain TJ2014-049 chromosome, complete genome

```
product length = 312
Forward primer  1        GACTGGGTGGTGCTCGAAG  19
Template        2126252  ...................  2126270

Reverse primer  1        GCGCTCCTCGGCCTGTAG  18
Template        2126563  ..................  2126546
```

>CP065865.1 Pseudomonas aeruginosa strain TJ2019-022 chromosome, complete genome

```
product length = 312
Forward primer  1        GACTGGGTGGTGCTCGAAG  19
Template        2192557  ...................  2192575

Reverse primer  1        GCGCTCCTCGGCCTGTAG  18
Template        2192868  ..................  2192851
```

>CP065866.1 Pseudomonas aeruginosa strain TJ2019-017 chromosome, complete genome

```
product length = 312
Forward primer  1        GACTGGGTGGTGCTCGAAG  19
Template        2067583  ...................  2067601

Reverse primer  1        GCGCTCCTCGGCCTGTAG  18
Template        2067894  ..................  2067877
```

>CP065848.1 Pseudomonas aeruginosa strain CMC-097 chromosome, complete genome

```
product length = 312
Forward primer  1        GACTGGGTGGTGCTCGAAG  19
Template        5512145  ...................  5512127

Reverse primer  1        GCGCTCCTCGGCCTGTAG  18
Template        5511834  ..................  5511851
```

>CP065417.1 Pseudomonas aeruginosa isolate P23 chromosome, complete genome

```
product length = 312
Forward primer  1        GACTGGGTGGTGCTCGAAG  19
Template        2125785  ...................  2125803

Reverse primer  1        GCGCTCCTCGGCCTGTAG  18
Template        2126096  ..................  2126079
```

>CP065412.1 Pseudomonas aeruginosa isolate P33 chromosome, complete genome

```
product length = 312
Forward primer  1        GACTGGGTGGTGCTCGAAG  19
Template        2131415  ...................  2131433

Reverse primer  1        GCGCTCCTCGGCCTGTAG  18
Template        2131726  ..................  2131709
```

>CP046402.2 Pseudomonas aeruginosa strain SE5331 chromosome, complete genome

```
product length = 312
Forward primer  1       GACTGGGTGGTGCTCGAAG  19
Template        999066  ...................  999048

Reverse primer  1       GCGCTCCTCGGCCTGTAG  18
Template        998755  ..................  998772
```

>CP046406.2 Pseudomonas aeruginosa strain SE5458 chromosome, complete genome

```
product length = 312
Forward primer  1        GACTGGGTGGTGCTCGAAG  19
Template        4556009  ...................  4555991

Reverse primer  1        GCGCTCCTCGGCCTGTAG  18
Template        4555698  ..................  4555715
```

>CP045552.2 Pseudomonas aeruginosa strain YT12746 chromosome, complete genome

```
product length = 312
Forward primer  1        GACTGGGTGGTGCTCGAAG  19
Template        2262666  ...................  2262684

Reverse primer  1        GCGCTCCTCGGCCTGTAG  18
Template        2262977  ..................  2262960
```

>CP060243.1 Pseudomonas aeruginosa strain A-I-1 chromosome, complete genome

```
product length = 312
Forward primer  1        GACTGGGTGGTGCTCGAAG  19
Template        2315106  ...................  2315124

Reverse primer  1        GCGCTCCTCGGCCTGTAG  18
Template        2315417  ..................  2315400
```

>CP060242.1 Pseudomonas aeruginosa strain B-I-1 chromosome, complete genome

```
product length = 312
Forward primer  1        GACTGGGTGGTGCTCGAAG  19
Template        2526851  ...................  2526869

Reverse primer  1        GCGCTCCTCGGCCTGTAG  18
Template        2527162  ..................  2527145
```

>CP060241.1 Pseudomonas aeruginosa strain C-I-1 chromosome, complete genome

```
product length = 312
Forward primer  1        GACTGGGTGGTGCTCGAAG  19
Template        4277763  ...................  4277745

Reverse primer  1        GCGCTCCTCGGCCTGTAG  18
Template        4277452  ..................  4277469
```

>CP060240.1 Pseudomonas aeruginosa strain G-I-1 chromosome, complete genome

```
product length = 312
Forward primer  1        GACTGGGTGGTGCTCGAAG  19
Template        2153970  ...................  2153988

Reverse primer  1        GCGCTCCTCGGCCTGTAG  18
Template        2154281  ..................  2154264
```

>LR898867.1 Pseudomonas aeruginosa isolate MINF_3A-sc-2280432 genome assembly, chromosome: 1

```
product length = 312
Forward primer  1        GACTGGGTGGTGCTCGAAG  19
Template        2081344  ...................  2081362

Reverse primer  1        GCGCTCCTCGGCCTGTAG  18
Template        2081655  ..................  2081638
```

>LR890619.1 Pseudomonas aeruginosa isolate MINF_7A-sc-2280434 genome assembly, chromosome: 1

```
product length = 312
Forward primer  1        GACTGGGTGGTGCTCGAAG  19
Template        2034022  ...................  2034040

Reverse primer  1        GCGCTCCTCGGCCTGTAG  18
Template        2034333  ..................  2034316
```

>CP061034.1 Pseudomonas aeruginosa strain PA3 chromosome, complete genome

```
product length = 312
Forward primer  1        GACTGGGTGGTGCTCGAAG  19
Template        2050263  ...................  2050281

Reverse primer  1        GCGCTCCTCGGCCTGTAG  18
Template        2050574  ..................  2050557
```

>CP060703.1 Pseudomonas aeruginosa strain NRD619 chromosome, complete genome

```
product length = 312
Forward primer  1        GACTGGGTGGTGCTCGAAG  19
Template        2027800  ...................  2027818

Reverse primer  1        GCGCTCCTCGGCCTGTAG  18
Template        2028111  ..................  2028094
```

>CP060086.1 Pseudomonas aeruginosa strain JNQH-PA57 chromosome, complete genome

```
product length = 312
Forward primer  1        GACTGGGTGGTGCTCGAAG  19
Template        2194327  ...................  2194345

Reverse primer  1        GCGCTCCTCGGCCTGTAG  18
```

```
Template        2194638  ..................  2194621
```

>CP059063.1 Pseudomonas aeruginosa strain GIMC5034:PA52Ts32 chromosome

```
product length = 312
Forward primer  1        GACTGGGTGGTGCTCGAAG  19
Template        4763108  ...................  4763090

Reverse primer  1        GCGCTCCTCGGCCTGTAG  18
Template        4762797  ..................  4762814
```

>CP058331.1 Pseudomonas aeruginosa strain ACR22 chromosome, complete genome

```
product length = 312
Forward primer  1        GACTGGGTGGTGCTCGAAG  19
Template        2631762  ...................  2631780

Reverse primer  1        GCGCTCCTCGGCCTGTAG  18
Template        2632073  ..................  2632056
```

>CP058333.1 Pseudomonas aeruginosa strain ACR20 chromosome, complete genome

```
product length = 312
Forward primer  1        GACTGGGTGGTGCTCGAAG  19
Template        3577647  ...................  3577629

Reverse primer  1        GCGCTCCTCGGCCTGTAG  18
Template        3577336  ..................  3577353
```

>CP053119.1 Pseudomonas aeruginosa strain A17CT chromosome

```
product length = 312
Forward primer  1        GACTGGGTGGTGCTCGAAG  19
Template        3434002  ...................  3433984

Reverse primer  1        GCGCTCCTCGGCCTGTAG  18
Template        3433691  ..................  3433708
```

>CP053118.1 Pseudomonas aeruginosa strain A17PBS chromosome

```
product length = 312
Forward primer  1        GACTGGGTGGTGCTCGAAG  19
Template        3433677  ...................  3433659

Reverse primer  1        GCGCTCCTCGGCCTGTAG  18
Template        3433366  ..................  3433383
```

>CP053117.1 Pseudomonas aeruginosa strain P16CT chromosome

```
product length = 312
Forward primer  1        GACTGGGTGGTGCTCGAAG  19
Template        3433772  ...................  3433754
```

```
Reverse primer  1        GCGCTCCTCGGCCTGTAG  18
Template        3433461  ..................  3433478
```

>CP053116.1 Pseudomonas aeruginosa strain P16PBS chromosome

```
product length = 312
Forward primer  1        GACTGGGTGGTGCTCGAAG  19
Template        3433697  ...................  3433679

Reverse primer  1        GCGCTCCTCGGCCTGTAG  18
Template        3433386  ..................  3433403
```

>CP053115.1 Pseudomonas aeruginosa strain P4CT chromosome

```
product length = 312
Forward primer  1        GACTGGGTGGTGCTCGAAG  19
Template        3433707  ...................  3433689

Reverse primer  1        GCGCTCCTCGGCCTGTAG  18
Template        3433396  ..................  3433413
```

>CP053114.1 Pseudomonas aeruginosa strain P4PBS chromosome

```
product length = 312
Forward primer  1        GACTGGGTGGTGCTCGAAG  19
Template        3433749  ...................  3433731

Reverse primer  1        GCGCTCCTCGGCCTGTAG  18
Template        3433438  ..................  3433455
```

>CP053113.1 Pseudomonas aeruginosa strain PAO1CT chromosome

```
product length = 312
Forward primer  1        GACTGGGTGGTGCTCGAAG  19
Template        3433754  ...................  3433736

Reverse primer  1        GCGCTCCTCGGCCTGTAG  18
Template        3433443  ..................  3433460
```

>CP053112.1 Pseudomonas aeruginosa strain PAO1PBS chromosome

```
product length = 312
Forward primer  1        GACTGGGTGGTGCTCGAAG  19
Template        3433705  ...................  3433687

Reverse primer  1        GCGCTCCTCGGCCTGTAG  18
Template        3433394  ..................  3433411
```

>CP053111.1 Pseudomonas aeruginosa strain UAB2CT chromosome

```
product length = 312
Forward primer  1        GACTGGGTGGTGCTCGAAG  19
Template        3433667  ...................  3433649
```

```
Reverse primer  1        GCGCTCCTCGGCCTGTAG  18
Template        3433356  ..................  3433373
```

>CP053110.1 Pseudomonas aeruginosa strain UAB2PBS chromosome

```
product length = 312
Forward primer  1        GACTGGGTGGTGCTCGAAG  19
Template        3433694  ...................  3433676

Reverse primer  1        GCGCTCCTCGGCCTGTAG  18
Template        3433383  ..................  3433400
```

>CP058332.1 Pseudomonas aeruginosa strain B18 chromosome, complete genome

```
product length = 312
Forward primer  1        GACTGGGTGGTGCTCGAAG  19
Template        6132324  ...................  6132306

Reverse primer  1        GCGCTCCTCGGCCTGTAG  18
Template        6132013  ..................  6132030
```

>CP058323.1 Pseudomonas aeruginosa strain LV chromosome

```
product length = 312
Forward primer  1        GACTGGGTGGTGCTCGAAG  19
Template        4933375  ...................  4933357

Reverse primer  1        GCGCTCCTCGGCCTGTAG  18
Template        4933064  ..................  4933081
```

>CP046405.1 Pseudomonas aeruginosa strain SE5443 chromosome, complete genome

```
product length = 312
Forward primer  1        GACTGGGTGGTGCTCGAAG  19
Template        2051392  ...................  2051410

Reverse primer  1        GCGCTCCTCGGCCTGTAG  18
Template        2051703  ..................  2051686
```

>CP046404.1 Pseudomonas aeruginosa strain SE5416 chromosome, complete genome

```
product length = 312
Forward primer  1        GACTGGGTGGTGCTCGAAG  19
Template        2100979  ...................  2100997

Reverse primer  1        GCGCTCCTCGGCCTGTAG  18
Template        2101290  ..................  2101273
```

>CP046403.1 Pseudomonas aeruginosa strain SE5369 chromosome, complete genome

```
product length = 312
Forward primer  1        GACTGGGTGGTGCTCGAAG  19
```

```
Template        2232852  ...................  2232870

Reverse primer  1        GCGCTCCTCGGCCTGTAG  18
Template        2233163  ..................  2233146
```

>CP056774.1 Pseudomonas aeruginosa strain CDN129 chromosome, complete genome

```
product length = 312
Forward primer  1        GACTGGGTGGTGCTCGAAG  19
Template        3526050  ...................  3526032

Reverse primer  1        GCGCTCCTCGGCCTGTAG  18
Template        3525739  ..................  3525756
```

>CP056100.1 Pseudomonas aeruginosa strain PABCH01 chromosome

```
product length = 312
Forward primer  1        GACTGGGTGGTGCTCGAAG  19
Template        2114349  ...................  2114367

Reverse primer  1        GCGCTCCTCGGCCTGTAG  18
Template        2114660  ..................  2114643
```

>CP056090.1 Pseudomonas aeruginosa strain PABCH42 chromosome

```
product length = 312
Forward primer  1        GACTGGGTGGTGCTCGAAG  19
Template        6232245  ...................  6232227

Reverse primer  1        GCGCTCCTCGGCCTGTAG  18
Template        6231934  ..................  6231951
```

>CP056095.1 Pseudomonas aeruginosa strain PABCH09 chromosome

```
product length = 312
Forward primer  1        GACTGGGTGGTGCTCGAAG  19
Template        2087091  ...................  2087109

Reverse primer  1        GCGCTCCTCGGCCTGTAG  18
Template        2087402  ..................  2087385
```

>CP056092.1 Pseudomonas aeruginosa strain PABCH14 chromosome

```
product length = 312
Forward primer  1        GACTGGGTGGTGCTCGAAG  19
Template        2249444  ...................  2249462

Reverse primer  1        GCGCTCCTCGGCCTGTAG  18
Template        2249755  ..................  2249738
```

>CP056101.1 Pseudomonas aeruginosa strain PABCH45 chromosome

```
product length = 312
```

```
Forward primer  1        GACTGGGTGGTGCTCGAAG  19
Template        3943596  ...................  3943578

Reverse primer  1        GCGCTCCTCGGCCTGTAG  18
Template        3943285  ..................  3943302
```

>CP056098.1 Pseudomonas aeruginosa strain PABCH05 chromosome

```
product length = 312
Forward primer  1        GACTGGGTGGTGCTCGAAG  19
Template        2052902  ...................  2052920

Reverse primer  1        GCGCTCCTCGGCCTGTAG  18
Template        2053213  ..................  2053196
```

>CP056094.1 Pseudomonas aeruginosa strain PABCH10 chromosome

```
product length = 312
Forward primer  1        GACTGGGTGGTGCTCGAAG  19
Template        2312261  ...................  2312279

Reverse primer  1        GCGCTCCTCGGCCTGTAG  18
Template        2312572  ..................  2312555
```

>CP056093.1 Pseudomonas aeruginosa strain PABCH13 chromosome

```
product length = 312
Forward primer  1        GACTGGGTGGTGCTCGAAG  19
Template        2042992  ...................  2043010

Reverse primer  1        GCGCTCCTCGGCCTGTAG  18
Template        2043303  ..................  2043286
```

>CP034908.2 Pseudomonas aeruginosa strain PA0750 chromosome, complete genome

```
product length = 312
Forward primer  1        GACTGGGTGGTGCTCGAAG  19
Template        3419324  ...................  3419306

Reverse primer  1        GCGCTCCTCGGCCTGTAG  18
Template        3419013  ..................  3419030
```

>CP054591.1 Pseudomonas aeruginosa strain CDN118 chromosome, complete genome

```
product length = 312
Forward primer  1        GACTGGGTGGTGCTCGAAG  19
Template        4441349  ...................  4441331

Reverse primer  1        GCGCTCCTCGGCCTGTAG  18
Template        4441038  ..................  4441055
```

>CP050335.1 Pseudomonas aeruginosa strain DVT401 chromosome, complete genome

```
product length = 312
Forward primer  1        GACTGGGTGGTGCTCGAAG  19
Template        2074365  ...................  2074383

Reverse primer  1        GCGCTCCTCGGCCTGTAG  18
Template        2074676  ..................  2074659
```

>CP050334.1 Pseudomonas aeruginosa strain DVT410 chromosome, complete genome

```
product length = 312
Forward primer  1        GACTGGGTGGTGCTCGAAG  19
Template        1999028  ...................  1999046

Reverse primer  1        GCGCTCCTCGGCCTGTAG  18
Template        1999339  ..................  1999322
```

>CP050333.1 Pseudomonas aeruginosa strain DVT412 chromosome, complete genome

```
product length = 312
Forward primer  1        GACTGGGTGGTGCTCGAAG  19
Template        3553949  ...................  3553931

Reverse primer  1        GCGCTCCTCGGCCTGTAG  18
Template        3553638  ..................  3553655
```

>CP050332.1 Pseudomonas aeruginosa strain DVT413 chromosome, complete genome

```
product length = 312
Forward primer  1        GACTGGGTGGTGCTCGAAG  19
Template        2126529  ...................  2126547

Reverse primer  1        GCGCTCCTCGGCCTGTAG  18
Template        2126840  ..................  2126823
```

>CP050331.1 Pseudomonas aeruginosa strain DVT414 chromosome, complete genome

```
product length = 312
Forward primer  1        GACTGGGTGGTGCTCGAAG  19
Template        2035908  ...................  2035926

Reverse primer  1        GCGCTCCTCGGCCTGTAG  18
Template        2036219  ..................  2036202
```

>CP050330.1 Pseudomonas aeruginosa strain DVT779 chromosome, complete genome

```
product length = 312
Forward primer  1        GACTGGGTGGTGCTCGAAG  19
Template        2094264  ...................  2094282

Reverse primer  1        GCGCTCCTCGGCCTGTAG  18
Template        2094575  ..................  2094558
```

>CP050329.1 Pseudomonas aeruginosa strain DVT417 chromosome, complete genome

```
product length = 312
Forward primer  1        GACTGGGTGGTGCTCGAAG  19
Template        2041722  ...................  2041740

Reverse primer  1        GCGCTCCTCGGCCTGTAG  18
Template        2042033  ..................  2042016
```

>CP050328.1 Pseudomonas aeruginosa strain DVT419 chromosome, complete genome

```
product length = 312
Forward primer  1        GACTGGGTGGTGCTCGAAG  19
Template        3688999  ...................  3688981

Reverse primer  1        GCGCTCCTCGGCCTGTAG  18
Template        3688688  ..................  3688705
```

>CP050327.1 Pseudomonas aeruginosa strain DVT421 chromosome, complete genome

```
product length = 312
Forward primer  1        GACTGGGTGGTGCTCGAAG  19
Template        3727647  ...................  3727629

Reverse primer  1        GCGCTCCTCGGCCTGTAG  18
Template        3727336  ..................  3727353
```

>CP050326.1 Pseudomonas aeruginosa strain DVT423 chromosome, complete genome

```
product length = 312
Forward primer  1        GACTGGGTGGTGCTCGAAG  19
Template        5100167  ...................  5100149

Reverse primer  1        GCGCTCCTCGGCCTGTAG  18
Template        5099856  ..................  5099873
```

>CP050325.1 Pseudomonas aeruginosa strain DVT425 chromosome, complete genome

```
product length = 312
Forward primer  1        GACTGGGTGGTGCTCGAAG  19
Template        1617607  ...................  1617625

Reverse primer  1        GCGCTCCTCGGCCTGTAG  18
Template        1617918  ..................  1617901
```

>CP050324.1 Pseudomonas aeruginosa strain DVT427 chromosome, complete genome

```
product length = 312
Forward primer  1        GACTGGGTGGTGCTCGAAG  19
Template        2073005  ...................  2073023

Reverse primer  1        GCGCTCCTCGGCCTGTAG  18
Template        2073316  ..................  2073299
```

>CP050323.1 Pseudomonas aeruginosa strain DVT429 chromosome, complete genome

```
product length = 312
Forward primer  1        GACTGGGTGGTGCTCGAAG  19
Template        5167056  ...................  5167038

Reverse primer  1        GCGCTCCTCGGCCTGTAG  18
Template        5166745  ..................  5166762
```

>CP050322.1 Pseudomonas aeruginosa strain DVT729 chromosome, complete genome

```
product length = 312
Forward primer  1        GACTGGGTGGTGCTCGAAG  19
Template        2178329  ...................  2178347

Reverse primer  1        GCGCTCCTCGGCCTGTAG  18
Template        2178640  ..................  2178623
```

>CP054572.1 Pseudomonas sp. FDAARGOS_761 chromosome, complete genome

```
product length = 312
Forward primer  1        GACTGGGTGGTGCTCGAAG  19
Template        3528189  ...................  3528171

Reverse primer  1        GCGCTCCTCGGCCTGTAG  18
Template        3527878  ..................  3527895
```

>CP054473.1 Pseudomonas aeruginosa strain PAAK095 chromosome, complete genome

```
product length = 312
Forward primer  1        GACTGGGTGGTGCTCGAAG  19
Template        5213439  ...................  5213421

Reverse primer  1        GCGCTCCTCGGCCTGTAG  18
Template        5213128  ..................  5213145
```

>CP054472.1 Pseudomonas aeruginosa strain PAAK088 chromosome, complete genome

```
product length = 312
Forward primer  1        GACTGGGTGGTGCTCGAAG  19
Template        2120473  ...................  2120491

Reverse primer  1        GCGCTCCTCGGCCTGTAG  18
Template        2120784  ..................  2120767
```

>CP050052.1 Pseudomonas aeruginosa strain LIUYANG-E chromosome, complete genome

```
product length = 312
Forward primer  1        GACTGGGTGGTGCTCGAAG  19
Template        2081317  ...................  2081335

Reverse primer  1        GCGCTCCTCGGCCTGTAG  18
Template        2081628  ..................  2081611
```

>CP050054.1 Pseudomonas aeruginosa strain LIUYANG-A chromosome, complete genome

```
product length = 312
Forward primer  1        GACTGGGTGGTGCTCGAAG  19
Template        2081317  ...................  2081335

Reverse primer  1        GCGCTCCTCGGCCTGTAG  18
Template        2081628  ..................  2081611
```

>CP050053.1 Pseudomonas aeruginosa strain LIUYANG-C chromosome, complete genome

```
product length = 312
Forward primer  1        GACTGGGTGGTGCTCGAAG  19
Template        3911403  ...................  3911385

Reverse primer  1        GCGCTCCTCGGCCTGTAG  18
Template        3911092  ..................  3911109
```

>CP053922.1 Pseudomonas aeruginosa strain YD001 chromosome, complete genome

```
product length = 312
Forward primer  1        GACTGGGTGGTGCTCGAAG  19
Template        2126262  ...................  2126280

Reverse primer  1        GCGCTCCTCGGCCTGTAG  18
Template        2126573  ..................  2126556
```

>CP053917.1 Pseudomonas aeruginosa strain PSE6684 chromosome, complete genome

```
product length = 312
Forward primer  1       GACTGGGTGGTGCTCGAAG  19
Template        773203  ...................  773185

Reverse primer  1       GCGCTCCTCGGCCTGTAG  18
Template        772892  ..................  772909
```

>CP053706.1 Pseudomonas aeruginosa strain PAC1 chromosome, complete genome

```
product length = 312
Forward primer  1        GACTGGGTGGTGCTCGAAG  19
Template        2903023  ...................  2903005

Reverse primer  1        GCGCTCCTCGGCCTGTAG  18
Template        2902712  ..................  2902729
```

>CP053705.1 Pseudomonas aeruginosa strain PAC6 chromosome, complete genome

```
product length = 312
Forward primer  1        GACTGGGTGGTGCTCGAAG  19
Template        2132451  ...................  2132469

Reverse primer  1        GCGCTCCTCGGCCTGTAG  18
Template        2132762  ..................  2132745
```

>CP053686.1 Pseudomonas aeruginosa strain SCAID PHRX1-2019 chromosome

```
product length = 312
Forward primer  1        GACTGGGTGGTGCTCGAAG  19
Template        5332166  ...................  5332184

Reverse primer  1        GCGCTCCTCGGCCTGTAG  18
Template        5332477  ..................  5332460
```

>CP044533.1 Pseudomonas aeruginosa strain Ps33 chromosome

```
product length = 312
Forward primer  1        GACTGGGTGGTGCTCGAAG  19
Template        1919658  ...................  1919640

Reverse primer  1        GCGCTCCTCGGCCTGTAG  18
Template        1919347  ..................  1919364
```

>CP051770.1 Pseudomonas aeruginosa strain GIMC5021:PA52Ts17, complete sequence

```
product length = 312
Forward primer  1        GACTGGGTGGTGCTCGAAG  19
Template        4790478  ...................  4790460

Reverse primer  1        GCGCTCCTCGGCCTGTAG  18
Template        4790167  ..................  4790184
```

>CP051768.1 Pseudomonas aeruginosa strain GIMC5020:PA52Ts2, complete sequence

```
product length = 312
Forward primer  1        GACTGGGTGGTGCTCGAAG  19
Template        4753729  ...................  4753711

Reverse primer  1        GCGCTCCTCGGCCTGTAG  18
Template        4753418  ..................  4753435
```

>CP051766.1 Pseudomonas aeruginosa strain GIMC5019:PA52Ts1, complete sequence

```
product length = 312
Forward primer  1        GACTGGGTGGTGCTCGAAG  19
Template        4799302  ...................  4799284

Reverse primer  1        GCGCTCCTCGGCCTGTAG  18
Template        4798991  ..................  4799008
```

>CP053028.1 Pseudomonas aeruginosa PAO1 chromosome, complete genome

```
product length = 312
Forward primer  1        GACTGGGTGGTGCTCGAAG  19
Template        2082141  ...................  2082159

Reverse primer  1        GCGCTCCTCGGCCTGTAG  18
Template        2082452  ..................  2082435
```

>CP052759.1 Pseudomonas aeruginosa strain LYT4 chromosome, complete genome

```
product length = 312
Forward primer  1        GACTGGGTGGTGCTCGAAG  19
Template        2140208  ...................  2140226

Reverse primer  1        GCGCTCCTCGGCCTGTAG  18
Template        2140519  ..................  2140502
```

>CP051547.1 Pseudomonas aeruginosa strain AA2 chromosome, complete genome

```
product length = 312
Forward primer  1        GACTGGGTGGTGCTCGAAG  19
Template        2051626  ...................  2051644

Reverse primer  1        GCGCTCCTCGGCCTGTAG  18
Template        2051937  ..................  2051920
```

>CP045916.1 Pseudomonas aeruginosa strain CF39S chromosome, complete genome

```
product length = 312
Forward primer  1        GACTGGGTGGTGCTCGAAG  19
Template        2074687  ...................  2074705

Reverse primer  1        GCGCTCCTCGGCCTGTAG  18
Template        2074998  ..................  2074981
```

>CP045002.1 Pseudomonas aeruginosa strain PAG5 chromosome, complete genome

```
product length = 312
Forward primer  1        GACTGGGTGGTGCTCGAAG  19
Template        2051043  ...................  2051061

Reverse primer  1        GCGCTCCTCGGCCTGTAG  18
Template        2051354  ..................  2051337
```

>CP021380.2 Pseudomonas aeruginosa strain CCBH4851 genome

```
product length = 312
Forward primer  1        GACTGGGTGGTGCTCGAAG  19
Template        2060945  ...................  2060963

Reverse primer  1        GCGCTCCTCGGCCTGTAG  18
Template        2061256  ..................  2061239
```

>CP049161.1 Pseudomonas aeruginosa strain MS14403 chromosome, complete genome

```
product length = 312
Forward primer  1        GACTGGGTGGTGCTCGAAG  19
Template        2031852  ...................  2031870

Reverse primer  1        GCGCTCCTCGGCCTGTAG  18
Template        2032163  ..................  2032146
```

>CP048791.1 Pseudomonas aeruginosa strain VIT PC9 chromosome, complete genome

```
product length = 312
Forward primer  1        GACTGGGTGGTGCTCGAAG  19
Template        5036030  ...................  5036012

Reverse primer  1        GCGCTCCTCGGCCTGTAG  18
Template        5035719  ..................  5035736
```

>LR739071.1 Pseudomonas aeruginosa strain C7-25 genome assembly, chromosome: C7-25

```
product length = 312
Forward primer  1        GACTGGGTGGTGCTCGAAG  19
Template        2114801  ...................  2114819

Reverse primer  1        GCGCTCCTCGGCCTGTAG  18
Template        2115112  ..................  2115095
```

>LR739069.1 Pseudomonas aeruginosa strain PcyII-40 genome assembly, chromosome: PcyII-40

```
product length = 312
Forward primer  1        GACTGGGTGGTGCTCGAAG  19
Template        2223914  ...................  2223932

Reverse primer  1        GCGCTCCTCGGCCTGTAG  18
Template        2224225  ..................  2224208
```

>LR739068.1 Pseudomonas aeruginosa strain PcyII-29 genome assembly, chromosome: PcyII-29

```
product length = 312
Forward primer  1        GACTGGGTGGTGCTCGAAG  19
Template        2075907  ...................  2075925

Reverse primer  1        GCGCTCCTCGGCCTGTAG  18
Template        2076218  ..................  2076201
```

>CP047697.1 Pseudomonas aeruginosa strain RD1-3 chromosome, complete genome

```
product length = 312
Forward primer  1        GACTGGGTGGTGCTCGAAG  19
Template        4820265  ...................  4820247

Reverse primer  1        GCGCTCCTCGGCCTGTAG  18
Template        4819954  ..................  4819971
```

>CP047592.1 Pseudomonas aeruginosa strain INP-43 chromosome, complete genome

```
product length = 312
Forward primer  1        GACTGGGTGGTGCTCGAAG  19
Template        3224951  ...................  3224969

Reverse primer  1        GCGCTCCTCGGCCTGTAG  18
Template        3225262  ..................  3225245
```

>CP028132.1 Pseudomonas aeruginosa strain YB01 chromosome, complete genome

```
product length = 312
Forward primer  1        GACTGGGTGGTGCTCGAAG  19
Template        2028867  ...................  2028885

Reverse primer  1        GCGCTCCTCGGCCTGTAG  18
Template        2029178  ..................  2029161
```

>CP025056.3 Pseudomonas aeruginosa strain PB367 chromosome, complete genome

```
product length = 312
Forward primer  1        GACTGGGTGGTGCTCGAAG  19
Template        2154633  ...................  2154651

Reverse primer  1        GCGCTCCTCGGCCTGTAG  18
Template        2154944  ..................  2154927
```

>CP025055.2 Pseudomonas aeruginosa strain PB350 chromosome, complete genome

```
product length = 312
Forward primer  1        GACTGGGTGGTGCTCGAAG  19
Template        2154633  ...................  2154651

Reverse primer  1        GCGCTCCTCGGCCTGTAG  18
Template        2154944  ..................  2154927
```

>CP047069.1 Pseudomonas aeruginosa strain Environ_1 chromosome

```
product length = 312
Forward primer  1        GACTGGGTGGTGCTCGAAG  19
Template        3339796  ...................  3339778

Reverse primer  1        GCGCTCCTCGGCCTGTAG  18
Template        3339485  ..................  3339502
```

>CP047063.1 Pseudomonas aeruginosa strain delta6_4 chromosome

```
product length = 312
Forward primer  1        GACTGGGTGGTGCTCGAAG  19
Template        3433675  ...................  3433657

Reverse primer  1        GCGCTCCTCGGCCTGTAG  18
Template        3433364  ..................  3433381
```

>CP047070.1 Pseudomonas aeruginosa strain Environ_2 chromosome

```
product length = 312
Forward primer  1        GACTGGGTGGTGCTCGAAG  19
Template        3433595  ...................  3433577

Reverse primer  1        GCGCTCCTCGGCCTGTAG  18
Template        3433284  ..................  3433301
```

>CP047064.1 Pseudomonas aeruginosa strain delta6_5 chromosome

```
product length = 312
Forward primer  1        GACTGGGTGGTGCTCGAAG  19
Template        3433693  ...................  3433675

Reverse primer  1        GCGCTCCTCGGCCTGTAG  18
Template        3433382  ..................  3433399
```

>CP047061.1 Pseudomonas aeruginosa strain delta6_2 chromosome

```
product length = 312
Forward primer  1        GACTGGGTGGTGCTCGAAG  19
Template        3433675  ...................  3433657

Reverse primer  1        GCGCTCCTCGGCCTGTAG  18
Template        3433364  ..................  3433381
```

>CP047062.1 Pseudomonas aeruginosa strain delta6_3 chromosome

```
product length = 312
Forward primer  1        GACTGGGTGGTGCTCGAAG  19
Template        3433679  ...................  3433661

Reverse primer  1        GCGCTCCTCGGCCTGTAG  18
Template        3433368  ..................  3433385
```

>CP047067.1 Pseudomonas aeruginosa strain Cas9_1 chromosome

```
product length = 312
Forward primer  1        GACTGGGTGGTGCTCGAAG  19
Template        3433693  ...................  3433675

Reverse primer  1        GCGCTCCTCGGCCTGTAG  18
Template        3433382  ..................  3433399
```

>CP047066.1 Pseudomonas aeruginosa strain delta10 chromosome

```
product length = 312
Forward primer  1        GACTGGGTGGTGCTCGAAG  19
Template        3433682  ...................  3433664

Reverse primer  1        GCGCTCCTCGGCCTGTAG  18
Template        3433371  ..................  3433388
```

>CP047068.1 Pseudomonas aeruginosa strain Cas9_2 chromosome

```
product length = 312
Forward primer  1        GACTGGGTGGTGCTCGAAG  19
Template        3433694  ...................  3433676

Reverse primer  1        GCGCTCCTCGGCCTGTAG  18
Template        3433383  ..................  3433400
```

>CP047065.1 Pseudomonas aeruginosa strain delta6_6 chromosome

```
product length = 312
Forward primer  1        GACTGGGTGGTGCTCGAAG  19
Template        3433674  ...................  3433656

Reverse primer  1        GCGCTCCTCGGCCTGTAG  18
Template        3433363  ..................  3433380
```

>CP039990.1 Pseudomonas aeruginosa strain T2101 chromosome, complete genome

```
product length = 312
Forward primer  1        GACTGGGTGGTGCTCGAAG  19
Template        2475063  ...................  2475081

Reverse primer  1        GCGCTCCTCGGCCTGTAG  18
Template        2475374  ..................  2475357
```

>CP039988.1 Pseudomonas aeruginosa strain T2436 chromosome, complete genome

```
product length = 312
Forward primer  1        GACTGGGTGGTGCTCGAAG  19
Template        2051909  ...................  2051927

Reverse primer  1        GCGCTCCTCGGCCTGTAG  18
Template        2052220  ..................  2052203
```

>CP046069.1 Pseudomonas aeruginosa strain KRP1 chromosome, complete genome

```
product length = 312
Forward primer  1        GACTGGGTGGTGCTCGAAG  19
Template        2082469  ...................  2082487

Reverse primer  1        GCGCTCCTCGGCCTGTAG  18
Template        2082780  ..................  2082763
```

>CP041945.1 Pseudomonas aeruginosa strain ST773 chromosome, complete genome

```
product length = 312
Forward primer  1        GACTGGGTGGTGCTCGAAG  19
Template        2089904  ...................  2089922

Reverse primer  1        GCGCTCCTCGGCCTGTAG  18
Template        2090215  ..................  2090198
```

>CP045739.1 Pseudomonas aeruginosa strain AG1 chromosome, complete genome

```
product length = 312
Forward primer  1        GACTGGGTGGTGCTCGAAG  19
Template        2304972  ...................  2304990

Reverse primer  1        GCGCTCCTCGGCCTGTAG  18
Template        2305283  ..................  2305266
```

>CP045768.1 Pseudomonas aeruginosa strain CFSAN084950 chromosome, complete genome

```
product length = 312
Forward primer  1        GACTGGGTGGTGCTCGAAG  19
Template        1065608  ...................  1065626

Reverse primer  1        GCGCTCCTCGGCCTGTAG  18
Template        1065919  ..................  1065902
```

>CP042967.1 Pseudomonas aeruginosa PA99 chromosome, complete genome

```
product length = 311
Forward primer  1        GACTGGGTGGTGCTCGAAG  19
Template        5254859  ...................  5254877

Reverse primer  1        GCGCTCCTCGGCCTGTAG  18
Template        5255169  ..................  5255152
```

>CP024630.1 Pseudomonas aeruginosa strain PA59 chromosome, complete genome

```
product length = 312
Forward primer  1        GACTGGGTGGTGCTCGAAG  19
Template        2109366  ...................  2109384

Reverse primer  1        GCGCTCCTCGGCCTGTAG  18
Template        2109677  ..................  2109660
```

>CP044006.1 Pseudomonas aeruginosa strain E90 chromosome, complete genome

```
product length = 312
Forward primer  1        GACTGGGTGGTGCTCGAAG  19
Template        2104081  ...................  2104099

Reverse primer  1        GCGCTCCTCGGCCTGTAG  18
Template        2104392  ..................  2104375
```

>CP043549.1 Pseudomonas aeruginosa strain GIMC5002:PAT-169 chromosome

```
product length = 312
Forward primer  1        GACTGGGTGGTGCTCGAAG  19
Template        4238074  ...................  4238056

Reverse primer  1        GCGCTCCTCGGCCTGTAG  18
Template        4237763  ..................  4237780
```

>CP043483.1 Pseudomonas aeruginosa strain GIMC5001:PAT-23 chromosome

```
product length = 312
Forward primer  1        GACTGGGTGGTGCTCGAAG  19
Template        2099187  ...................  2099205

Reverse primer  1        GCGCTCCTCGGCCTGTAG  18
Template        2099498  ..................  2099481
```

>LR700248.1 Pseudomonas aeruginosa isolate ID40 genome assembly, chromosome: ID40_omosome

```
product length = 312
Forward primer  1        GACTGGGTGGTGCTCGAAG  19
Template        6021341  ...................  6021359

Reverse primer  1        GCGCTCCTCGGCCTGTAG  18
Template        6021652  ..................  6021635
```

>CP042269.1 Pseudomonas aeruginosa strain HOU1 chromosome, complete genome

```
product length = 312
Forward primer  1        GACTGGGTGGTGCTCGAAG  19
Template        1951167  ...................  1951185

Reverse primer  1        GCGCTCCTCGGCCTGTAG  18
Template        1951478  ..................  1951461
```

>CP043328.1 Pseudomonas aeruginosa strain CCUG 51971 chromosome, complete genome

```
product length = 312
Forward primer  1        GACTGGGTGGTGCTCGAAG  19
Template        2260969  ...................  2260987

Reverse primer  1        GCGCTCCTCGGCCTGTAG  18
Template        2261280  ..................  2261263
```

>CP028959.1 Pseudomonas aeruginosa strain IMP66 chromosome, complete genome

```
product length = 312
Forward primer  1        GACTGGGTGGTGCTCGAAG  19
Template        2079968  ...................  2079986

Reverse primer  1        GCGCTCCTCGGCCTGTAG  18
Template        2080279  ..................  2080262
```

>CP028848.1 Pseudomonas aeruginosa strain IMP67 chromosome, complete genome

```
product length = 312
Forward primer  1        GACTGGGTGGTGCTCGAAG  19
Template        2065135  ...................  2065153

Reverse primer  1        GCGCTCCTCGGCCTGTAG  18
Template        2065446  ..................  2065429
```

>CP028849.1 Pseudomonas aeruginosa strain IMP68 chromosome, complete genome

```
product length = 312
Forward primer  1        GACTGGGTGGTGCTCGAAG  19
Template        2065053  ...................  2065071

Reverse primer  1        GCGCTCCTCGGCCTGTAG  18
```

```
Template         2065364  ..................  2065347
```

>CP040684.1 Pseudomonas aeruginosa strain C79 chromosome, complete genome

```
product length = 312
Forward primer  1        GACTGGGTGGTGCTCGAAG  19
Template        2615431  ...................  2615413

Reverse primer  1        GCGCTCCTCGGCCTGTAG  18
Template        2615120  ..................  2615137
```

>CP041785.1 Pseudomonas aeruginosa strain SCAID WND3-2019 chromosome

```
product length = 312
Forward primer  1        GACTGGGTGGTGCTCGAAG  19
Template        1139749  ...................  1139767

Reverse primer  1        GCGCTCCTCGGCCTGTAG  18
Template        1140060  ..................  1140043
```

>CP041787.1 Pseudomonas aeruginosa strain SCAID WND1-2019 chromosome

```
product length = 312
Forward primer  1      GACTGGGTGGTGCTCGAAG  19
Template        56160  ...................  56178

Reverse primer  1      GCGCTCCTCGGCCTGTAG  18
Template        56471  ..................  56454
```

>CP041786.1 Pseudomonas aeruginosa strain SCAID WND2-2019 chromosome

```
product length = 312
Forward primer  1        GACTGGGTGGTGCTCGAAG  19
Template        3360366  ...................  3360348

Reverse primer  1        GCGCTCCTCGGCCTGTAG  18
Template        3360055  ..................  3360072
```

>CP041773.1 Pseudomonas aeruginosa strain 519119 chromosome, complete genome

```
product length = 312
Forward primer  1        GACTGGGTGGTGCTCGAAG  19
Template        4587185  ...................  4587203

Reverse primer  1        GCGCTCCTCGGCCTGTAG  18
Template        4587496  ..................  4587479
```

>CP041772.1 Pseudomonas aeruginosa strain 243931 chromosome, complete genome

```
product length = 312
Forward primer  1        GACTGGGTGGTGCTCGAAG  19
Template        4468194  ...................  4468212
```

```
Reverse primer  1        GCGCTCCTCGGCCTGTAG  18
Template        4468505  ..................  4468488
```

>CP041771.1 Pseudomonas aeruginosa strain A681 chromosome, complete genome

```
product length = 312
Forward primer  1        GACTGGGTGGTGCTCGAAG  19
Template        2204872  ...................  2204890

Reverse primer  1        GCGCTCCTCGGCCTGTAG  18
Template        2205183  ..................  2205166
```

>CP041774.1 Pseudomonas aeruginosa strain 60503 chromosome, complete genome

```
product length = 312
Forward primer  1        GACTGGGTGGTGCTCGAAG  19
Template        2084692  ...................  2084710

Reverse primer  1        GCGCTCCTCGGCCTGTAG  18
Template        2085003  ..................  2084986
```

>LR657304.1 Pseudomonas aeruginosa strain PAK genome assembly, chromosome: 1

```
product length = 312
Forward primer  1        GACTGGGTGGTGCTCGAAG  19
Template        2058501  ...................  2058519

Reverse primer  1        GCGCTCCTCGGCCTGTAG  18
Template        2058812  ..................  2058795
```

>CP034244.1 Pseudomonas aeruginosa UCBPP-PA14 chromosome

```
product length = 312
Forward primer  1        GACTGGGTGGTGCTCGAAG  19
Template        2135352  ...................  2135370

Reverse primer  1        GCGCTCCTCGGCCTGTAG  18
Template        2135663  ..................  2135646
```

>CP041013.1 Pseudomonas aeruginosa strain FDAARGOS_610 chromosome, complete genome

```
product length = 312
Forward primer  1        GACTGGGTGGTGCTCGAAG  19
Template        4702245  ...................  4702227

Reverse primer  1        GCGCTCCTCGGCCTGTAG  18
Template        4701934  ..................  4701951
```

>CP041008.1 Pseudomonas aeruginosa strain FDAARGOS_767 chromosome, complete genome

```
product length = 312
Forward primer  1        GACTGGGTGGTGCTCGAAG  19
Template        3641750  ...................  3641732
```

```
Reverse primer  1        GCGCTCCTCGGCCTGTAG  18
Template        3641439  ..................  3641456
```

>CP032569.2 Pseudomonas aeruginosa strain BA7823 chromosome, complete genome

```
product length = 312
Forward primer  1        GACTGGGTGGTGCTCGAAG  19
Template        1901922  ...................  1901940

Reverse primer  1        GCGCTCCTCGGCCTGTAG  18
Template        1902233  ..................  1902216
```

>CP040127.1 Pseudomonas aeruginosa strain PA298 chromosome, complete genome

```
product length = 312
Forward primer  1        GACTGGGTGGTGCTCGAAG  19
Template        2164476  ...................  2164494

Reverse primer  1        GCGCTCCTCGGCCTGTAG  18
Template        2164787  ..................  2164770
```

>LR590474.1 Pseudomonas aeruginosa strain NCTC13618 genome assembly, chromosome: 1

```
product length = 312
Forward primer  1        GACTGGGTGGTGCTCGAAG  19
Template        2125484  ...................  2125502

Reverse primer  1        GCGCTCCTCGGCCTGTAG  18
Template        2125795  ..................  2125778
```

>LR590473.1 Pseudomonas aeruginosa strain NCTC13359 genome assembly, chromosome: 1

```
product length = 312
Forward primer  1        GACTGGGTGGTGCTCGAAG  19
Template        2526401  ...................  2526419

Reverse primer  1        GCGCTCCTCGGCCTGTAG  18
Template        2526712  ..................  2526695
```

>LR590472.1 Pseudomonas aeruginosa strain NCTC13620 genome assembly, chromosome: 1

```
product length = 312
Forward primer  1        GACTGGGTGGTGCTCGAAG  19
Template        2151952  ...................  2151970

Reverse primer  1        GCGCTCCTCGGCCTGTAG  18
Template        2152263  ..................  2152246
```

>CP039749.1 Pseudomonas aeruginosa strain PRD-10 chromosome

```
product length = 312
Forward primer  1        GACTGGGTGGTGCTCGAAG  19
```

```
Template        1415502  ...................  1415520

Reverse primer  1        GCGCTCCTCGGCCTGTAG  18
Template        1415813  ..................  1415796
```

>CP038661.1 Pseudomonas aeruginosa strain AJ D 2 chromosome

```
product length = 312
Forward primer  1        GACTGGGTGGTGCTCGAAG  19
Template        3444023  ...................  3444005

Reverse primer  1        GCGCTCCTCGGCCTGTAG  18
Template        3443712  ..................  3443729
```

>CP037925.1 Pseudomonas aeruginosa strain AES1M chromosome, complete genome

```
product length = 312
Forward primer  1        GACTGGGTGGTGCTCGAAG  19
Template        4041136  ...................  4041118

Reverse primer  1        GCGCTCCTCGGCCTGTAG  18
Template        4040825  ..................  4040842
```

>CP037926.1 Pseudomonas aeruginosa strain AES1R chromosome, complete genome

```
product length = 312
Forward primer  1        GACTGGGTGGTGCTCGAAG  19
Template        2036537  ...................  2036555

Reverse primer  1        GCGCTCCTCGGCCTGTAG  18
Template        2036848  ..................  2036831
```

>CP028332.1 Pseudomonas aeruginosa strain PA-VAP-1 chromosome

```
product length = 312
Forward primer  1        GACTGGGTGGTGCTCGAAG  19
Template        4379771  ...................  4379789

Reverse primer  1        GCGCTCCTCGGCCTGTAG  18
Template        4380082  ..................  4380065
```

>CP028331.1 Pseudomonas aeruginosa strain PA-VAP-2 chromosome

```
product length = 312
Forward primer  1        GACTGGGTGGTGCTCGAAG  19
Template        3730418  ...................  3730400

Reverse primer  1        GCGCTCCTCGGCCTGTAG  18
Template        3730107  ..................  3730124
```

>CP028330.1 Pseudomonas aeruginosa strain PA-VAP-3 chromosome

```
product length = 312
```

```
Forward primer  1        GACTGGGTGGTGCTCGAAG  19
Template        4211433  ...................  4211451

Reverse primer  1        GCGCTCCTCGGCCTGTAG  18
Template        4211744  ..................  4211727
```

>CP022478.1 Pseudomonas aeruginosa strain LW chromosome, complete genome

```
product length = 312
Forward primer  1       GACTGGGTGGTGCTCGAAG  19
Template        916314  ...................  916332

Reverse primer  1       GCGCTCCTCGGCCTGTAG  18
Template        916625  ..................  916608
```

>CP034430.1 Pseudomonas aeruginosa strain GIMC5016:PA1840 chromosome

```
product length = 312
Forward primer  1        GACTGGGTGGTGCTCGAAG  19
Template        2209536  ...................  2209554

Reverse primer  1        GCGCTCCTCGGCCTGTAG  18
Template        2209847  ..................  2209830
```

>CP034429.1 Pseudomonas aeruginosa strain GIMC5015:PAKB6, complete sequence

```
product length = 312
Forward primer  1        GACTGGGTGGTGCTCGAAG  19
Template        2081662  ...................  2081680

Reverse primer  1        GCGCTCCTCGGCCTGTAG  18
Template        2081973  ..................  2081956
```

>LR134342.1 Pseudomonas aeruginosa strain NCTC10728 genome assembly, chromosome: 1

```
product length = 312
Forward primer  1        GACTGGGTGGTGCTCGAAG  19
Template        5027016  ...................  5027034

Reverse primer  1        GCGCTCCTCGGCCTGTAG  18
Template        5027327  ..................  5027310
```

>LR134330.1 Pseudomonas aeruginosa strain NCTC13715 genome assembly, chromosome: 1

```
product length = 312
Forward primer  1        GACTGGGTGGTGCTCGAAG  19
Template        3933733  ...................  3933751

Reverse primer  1        GCGCTCCTCGGCCTGTAG  18
Template        3934044  ..................  3934027
```

>LR134309.1 Pseudomonas aeruginosa strain NCTC12903 genome assembly, chromosome: 1

```
product length = 312
Forward primer  1        GACTGGGTGGTGCTCGAAG  19
Template        2027478  ...................  2027496

Reverse primer  1        GCGCTCCTCGGCCTGTAG  18
Template        2027789  ..................  2027772
```

>LR134308.1 Pseudomonas aeruginosa strain NCTC11445 genome assembly, chromosome: 1

```
product length = 312
Forward primer  1        GACTGGGTGGTGCTCGAAG  19
Template        3390497  ...................  3390479

Reverse primer  1        GCGCTCCTCGGCCTGTAG  18
Template        3390186  ..................  3390203
```

>LR134300.1 Pseudomonas fluorescens strain NCTC10783 genome assembly, chromosome: 1

```
product length = 312
Forward primer  1       GACTGGGTGGTGCTCGAAG  19
Template        915928  ...................  915946

Reverse primer  1       GCGCTCCTCGGCCTGTAG  18
Template        916239  ..................  916222
```

>CP032541.1 Pseudomonas aeruginosa strain PGN5 chromosome

```
product length = 312
Forward primer  1        GACTGGGTGGTGCTCGAAG  19
Template        3433702  ...................  3433684

Reverse primer  1        GCGCTCCTCGGCCTGTAG  18
Template        3433391  ..................  3433408
```

>CP032540.1 Pseudomonas aeruginosa strain PGN4 chromosome

```
product length = 312
Forward primer  1        GACTGGGTGGTGCTCGAAG  19
Template        3433702  ...................  3433684

Reverse primer  1        GCGCTCCTCGGCCTGTAG  18
Template        3433391  ..................  3433408
```

>CP034434.1 Pseudomonas aeruginosa strain SP2230 chromosome, complete genome

```
product length = 312
Forward primer  1        GACTGGGTGGTGCTCGAAG  19
Template        1204032  ...................  1204050

Reverse primer  1        GCGCTCCTCGGCCTGTAG  18
Template        1204343  ..................  1204326
```

>CP034435.1 Pseudomonas aeruginosa strain B14130 chromosome, complete genome

```
product length = 312
Forward primer  1        GACTGGGTGGTGCTCGAAG  19
Template        4332891  ...................  4332873

Reverse primer  1        GCGCTCCTCGGCCTGTAG  18
Template        4332580  ..................  4332597
```

>CP034436.1 Pseudomonas aeruginosa strain B17932 chromosome, complete genome

```
product length = 312
Forward primer  1        GACTGGGTGGTGCTCGAAG  19
Template        4159333  ...................  4159315

Reverse primer  1        GCGCTCCTCGGCCTGTAG  18
Template        4159022  ..................  4159039
```

>CP034409.1 Pseudomonas aeruginosa strain SP4527 chromosome, complete genome

```
product length = 312
Forward primer  1       GACTGGGTGGTGCTCGAAG  19
Template        482843  ...................  482861

Reverse primer  1       GCGCTCCTCGGCCTGTAG  18
Template        483154  ..................  483137
```

>CP034369.1 Pseudomonas aeruginosa strain SP4371 chromosome, complete genome

```
product length = 312
Forward primer  1        GACTGGGTGGTGCTCGAAG  19
Template        1389328  ...................  1389346

Reverse primer  1        GCGCTCCTCGGCCTGTAG  18
Template        1389639  ..................  1389622
```

>CP034368.1 Pseudomonas aeruginosa strain B41226 chromosome, complete genome

```
product length = 312
Forward primer  1        GACTGGGTGGTGCTCGAAG  19
Template        3883044  ...................  3883026

Reverse primer  1        GCGCTCCTCGGCCTGTAG  18
Template        3882733  ..................  3882750
```

>CP034354.1 Pseudomonas aeruginosa strain IMP-13 chromosome, complete genome

```
product length = 312
Forward primer  1        GACTGGGTGGTGCTCGAAG  19
Template        5219294  ...................  5219312

Reverse primer  1        GCGCTCCTCGGCCTGTAG  18
Template        5219605  ..................  5219588
```

>LR130537.1 Pseudomonas aeruginosa isolate paerg012 genome assembly, chromosome: 0

```
product length = 312
Forward primer  1        GACTGGGTGGTGCTCGAAG  19
Template        2036744  ...................  2036762

Reverse primer  1        GCGCTCCTCGGCCTGTAG  18
Template        2037055  ..................  2037038
```

>LR130536.1 Pseudomonas aeruginosa isolate paerg010 genome assembly, chromosome: 0

```
product length = 312
Forward primer  1        GACTGGGTGGTGCTCGAAG  19
Template        2036759  ...................  2036777

Reverse primer  1        GCGCTCCTCGGCCTGTAG  18
Template        2037070  ..................  2037053
```

>LR130535.1 Pseudomonas aeruginosa isolate paerg011 genome assembly, chromosome: 0

```
product length = 312
Forward primer  1        GACTGGGTGGTGCTCGAAG  19
Template        2036822  ...................  2036840

Reverse primer  1        GCGCTCCTCGGCCTGTAG  18
Template        2037133  ..................  2037116
```

>LR130534.1 Pseudomonas aeruginosa isolate paerg005 genome assembly, chromosome: 0

```
product length = 312
Forward primer  1        GACTGGGTGGTGCTCGAAG  19
Template        2182272  ...................  2182290

Reverse primer  1        GCGCTCCTCGGCCTGTAG  18
Template        2182583  ..................  2182566
```

>LR130533.1 Pseudomonas aeruginosa isolate paerg009 genome assembly, chromosome: 0

```
product length = 312
Forward primer  1        GACTGGGTGGTGCTCGAAG  19
Template        5498189  ...................  5498207

Reverse primer  1        GCGCTCCTCGGCCTGTAG  18
Template        5498500  ..................  5498483
```

>LR130531.1 Pseudomonas aeruginosa isolate paerg004 genome assembly, chromosome: 0

```
product length = 312
Forward primer  1        GACTGGGTGGTGCTCGAAG  19
Template        5788403  ...................  5788421

Reverse primer  1        GCGCTCCTCGGCCTGTAG  18
Template        5788714  ..................  5788697
```

>LR130530.1 Pseudomonas aeruginosa isolate paerg003 genome assembly, chromosome: 0

```
product length = 312
Forward primer  1        GACTGGGTGGTGCTCGAAG  19
Template        2036772  ...................  2036790

Reverse primer  1        GCGCTCCTCGGCCTGTAG  18
Template        2037083  ..................  2037066
```

>LR130528.1 Pseudomonas aeruginosa isolate paerg000 genome assembly, chromosome: 0

```
product length = 312
Forward primer  1        GACTGGGTGGTGCTCGAAG  19
Template        4902868  ...................  4902850

Reverse primer  1        GCGCTCCTCGGCCTGTAG  18
Template        4902557  ..................  4902574
```

>LR130527.1 Pseudomonas aeruginosa isolate paerg002 genome assembly, chromosome: 0

```
product length = 312
Forward primer  1        GACTGGGTGGTGCTCGAAG  19
Template        5151210  ...................  5151228

Reverse primer  1        GCGCTCCTCGGCCTGTAG  18
Template        5151521  ..................  5151504
```

>CP033832.1 Pseudomonas aeruginosa strain FDAARGOS_505 chromosome, complete genome

```
product length = 312
Forward primer  1        GACTGGGTGGTGCTCGAAG  19
Template        3622784  ...................  3622802

Reverse primer  1        GCGCTCCTCGGCCTGTAG  18
Template        3623095  ..................  3623078
```

>CP033833.1 Pseudomonas aeruginosa strain FDAARGOS_571 chromosome, complete genome

```
product length = 312
Forward primer  1        GACTGGGTGGTGCTCGAAG  19
Template        5351254  ...................  5351272

Reverse primer  1        GCGCTCCTCGGCCTGTAG  18
Template        5351565  ..................  5351548
```

>CP033843.1 Pseudomonas aeruginosa strain FDAARGOS_501 chromosome, complete genome

```
product length = 312
Forward primer  1        GACTGGGTGGTGCTCGAAG  19
Template        4636666  ...................  4636684

Reverse primer  1        GCGCTCCTCGGCCTGTAG  18
Template        4636977  ..................  4636960
```

>CP033771.1 Pseudomonas aeruginosa strain FDAARGOS_532 chromosome, complete genome

```
product length = 312
Forward primer  1        GACTGGGTGGTGCTCGAAG  19
Template        1161756  ...................  1161738

Reverse primer  1        GCGCTCCTCGGCCTGTAG  18
Template        1161445  ..................  1161462
```

>CP033684.1 Pseudomonas aeruginosa strain H26027 chromosome, complete genome

```
product length = 312
Forward primer  1        GACTGGGTGGTGCTCGAAG  19
Template        2236420  ...................  2236438

Reverse primer  1        GCGCTCCTCGGCCTGTAG  18
Template        2236731  ..................  2236714
```

>CP033686.1 Pseudomonas aeruginosa strain H25883 chromosome, complete genome

```
product length = 312
Forward primer  1        GACTGGGTGGTGCTCGAAG  19
Template        2112100  ...................  2112118

Reverse primer  1        GCGCTCCTCGGCCTGTAG  18
Template        2112411  ..................  2112394
```

>CP033685.1 Pseudomonas aeruginosa strain H26023 chromosome, complete genome

```
product length = 312
Forward primer  1        GACTGGGTGGTGCTCGAAG  19
Template        2128232  ...................  2128250

Reverse primer  1        GCGCTCCTCGGCCTGTAG  18
Template        2128543  ..................  2128526
```

>CP029713.1 Pseudomonas aeruginosa strain BH9 chromosome

```
product length = 312
Forward primer  1        GACTGGGTGGTGCTCGAAG  19
Template        2185560  ...................  2185578

Reverse primer  1        GCGCTCCTCGGCCTGTAG  18
Template        2185871  ..................  2185854
```

>CP033439.1 Pseudomonas aeruginosa strain SP4528 chromosome, complete genome

```
product length = 312
Forward primer  1        GACTGGGTGGTGCTCGAAG  19
Template        4475759  ...................  4475741

Reverse primer  1        GCGCTCCTCGGCCTGTAG  18
Template        4475448  ..................  4475465
```

>CP033432.1 Pseudomonas aeruginosa strain BA15561 chromosome, complete genome

```
product length = 312
Forward primer  1        GACTGGGTGGTGCTCGAAG  19
Template        2618482  ...................  2618464

Reverse primer  1        GCGCTCCTCGGCCTGTAG  18
Template        2618171  ..................  2618188
```

>CP033084.1 Pseudomonas aeruginosa strain PA-3 chromosome, complete genome

```
product length = 312
Forward primer  1        GACTGGGTGGTGCTCGAAG  19
Template        2415474  ...................  2415456

Reverse primer  1        GCGCTCCTCGGCCTGTAG  18
Template        2415163  ..................  2415180
```

>CP030075.1 Pseudomonas aeruginosa strain 6762 chromosome

```
product length = 312
Forward primer  1        GACTGGGTGGTGCTCGAAG  19
Template        4152068  ...................  4152086

Reverse primer  1        GCGCTCCTCGGCCTGTAG  18
Template        4152379  ..................  4152362
```

>CP032552.1 Pseudomonas aeruginosa strain PA34 chromosome, complete genome

```
product length = 312
Forward primer  1        GACTGGGTGGTGCTCGAAG  19
Template        2063796  ...................  2063814

Reverse primer  1        GCGCTCCTCGGCCTGTAG  18
Template        2064107  ..................  2064090
```

>CP032761.1 Pseudomonas aeruginosa strain 268 chromosome, complete genome

```
product length = 312
Forward primer  1        GACTGGGTGGTGCTCGAAG  19
Template        2269369  ...................  2269387

Reverse primer  1        GCGCTCCTCGGCCTGTAG  18
Template        2269680  ..................  2269663
```

>CP028584.2 Pseudomonas aeruginosa strain WCHPA075019 chromosome, complete genome

```
product length = 312
Forward primer  1        GACTGGGTGGTGCTCGAAG  19
Template        2273948  ...................  2273966

Reverse primer  1        GCGCTCCTCGGCCTGTAG  18
Template        2274259  ..................  2274242
```

>CP032257.1 Pseudomonas aeruginosa strain AR_0111 chromosome, complete genome

```
product length = 312
Forward primer  1        GACTGGGTGGTGCTCGAAG  19
Template        5506264  ...................  5506282

Reverse primer  1        GCGCTCCTCGGCCTGTAG  18
Template        5506575  ..................  5506558
```

>CP031877.1 Pseudomonas aeruginosa strain WPB100 chromosome

```
product length = 312
Forward primer  1        GACTGGGTGGTGCTCGAAG  19
Template        4990014  ...................  4989996

Reverse primer  1        GCGCTCCTCGGCCTGTAG  18
Template        4989703  ..................  4989720
```

>CP031876.1 Pseudomonas aeruginosa strain WPB101 chromosome

```
product length = 312
Forward primer  1        GACTGGGTGGTGCTCGAAG  19
Template        5311363  ...................  5311345

Reverse primer  1        GCGCTCCTCGGCCTGTAG  18
Template        5311052  ..................  5311069
```

>CP031878.1 Pseudomonas aeruginosa strain WPB099 chromosome

```
product length = 312
Forward primer  1        GACTGGGTGGTGCTCGAAG  19
Template        4890399  ...................  4890381

Reverse primer  1        GCGCTCCTCGGCCTGTAG  18
Template        4890088  ..................  4890105
```

>CP031879.1 Pseudomonas aeruginosa strain WPB098 chromosome

```
product length = 312
Forward primer  1        GACTGGGTGGTGCTCGAAG  19
Template        4022726  ...................  4022708

Reverse primer  1        GCGCTCCTCGGCCTGTAG  18
Template        4022415  ..................  4022432
```

>CP029605.1 Pseudomonas aeruginosa strain 24Pae112 chromosome, complete genome

```
product length = 312
Forward primer  1        GACTGGGTGGTGCTCGAAG  19
Template        2077218  ...................  2077236

Reverse primer  1        GCGCTCCTCGGCCTGTAG  18
Template        2077529  ..................  2077512
```

>CP031660.1 Pseudomonas aeruginosa strain PABL017 chromosome, complete genome

```
product length = 312
Forward primer  1        GACTGGGTGGTGCTCGAAG  19
Template        2094305  ...................  2094323

Reverse primer  1        GCGCTCCTCGGCCTGTAG  18
Template        2094616  ..................  2094599
```

>CP031659.1 Pseudomonas aeruginosa strain PABL012 chromosome, complete genome

```
product length = 312
Forward primer  1        GACTGGGTGGTGCTCGAAG  19
Template        2179639  ...................  2179657

Reverse primer  1        GCGCTCCTCGGCCTGTAG  18
Template        2179950  ..................  2179933
```

>CP030911.1 Pseudomonas aeruginosa strain Y71 chromosome, complete genome

```
product length = 312
Forward primer  1        GACTGGGTGGTGCTCGAAG  19
Template        2114829  ...................  2114847

Reverse primer  1        GCGCTCCTCGGCCTGTAG  18
Template        2115140  ..................  2115123
```

>CP035739.1 Pseudomonas aeruginosa strain 1334/14 chromosome, complete genome

```
product length = 312
Forward primer  1        GACTGGGTGGTGCTCGAAG  19
Template        1878423  ...................  1878405

Reverse primer  1        GCGCTCCTCGGCCTGTAG  18
Template        1878112  ..................  1878129
```

>CP031449.2 Pseudomonas aeruginosa strain 97 chromosome, complete genome

```
product length = 312
Forward primer  1        GACTGGGTGGTGCTCGAAG  19
Template        2221103  ...................  2221121

Reverse primer  1        GCGCTCCTCGGCCTGTAG  18
Template        2221414  ..................  2221397
```

>LS998783.1 Pseudomonas aeruginosa isolate 1 genome assembly, chromosome: 1

```
product length = 312
Forward primer  1        GACTGGGTGGTGCTCGAAG  19
Template        2326890  ...................  2326908

Reverse primer  1        GCGCTCCTCGGCCTGTAG  18
Template        2327201  ..................  2327184
```

>CP032126.1 Pseudomonas aeruginosa strain PAO1161 chromosome, complete genome

```
product length = 312
Forward primer  1        GACTGGGTGGTGCTCGAAG  19
Template        2082132  ...................  2082150

Reverse primer  1        GCGCTCCTCGGCCTGTAG  18
Template        2082443  ..................  2082426
```

>CP030913.1 Pseudomonas aeruginosa strain Y89 chromosome, complete genome

```
product length = 312
Forward primer  1        GACTGGGTGGTGCTCGAAG  19
Template        2115482  ...................  2115500

Reverse primer  1        GCGCTCCTCGGCCTGTAG  18
Template        2115793  ..................  2115776
```

>CP030912.1 Pseudomonas aeruginosa strain Y82 chromosome, complete genome

```
product length = 312
Forward primer  1        GACTGGGTGGTGCTCGAAG  19
Template        2284372  ...................  2284390

Reverse primer  1        GCGCTCCTCGGCCTGTAG  18
Template        2284683  ..................  2284666
```

>CP030910.1 Pseudomonas aeruginosa strain Y31 chromosome, complete genome

```
product length = 312
Forward primer  1        GACTGGGTGGTGCTCGAAG  19
Template        2914883  ...................  2914901

Reverse primer  1        GCGCTCCTCGGCCTGTAG  18
Template        2915194  ..................  2915177
```

>CP030861.1 Pseudomonas aeruginosa strain HS9 chromosome, complete genome

```
product length = 312
Forward primer  1       GACTGGGTGGTGCTCGAAG  19
Template        372103  ...................  372085

Reverse primer  1       GCGCTCCTCGGCCTGTAG  18
Template        371792  ..................  371809
```

>CP030327.1 Pseudomonas aeruginosa strain AR_458 chromosome, complete genome

```
product length = 312
Forward primer  1        GACTGGGTGGTGCTCGAAG  19
Template        5272967  ...................  5272985

Reverse primer  1        GCGCTCCTCGGCCTGTAG  18
Template        5273278  ..................  5273261
```

>CP030351.1 Pseudomonas aeruginosa strain AR_460 chromosome, complete genome

```
product length = 312
Forward primer  1       GACTGGGTGGTGCTCGAAG  19
Template        406807  ...................  406825

Reverse primer  1       GCGCTCCTCGGCCTGTAG  18
Template        407118  ..................  407101
```

>CP029707.1 Pseudomonas aeruginosa strain K34-7 chromosome, complete genome

```
product length = 312
Forward primer  1       GACTGGGTGGTGCTCGAAG  19
Template        662042  ...................  662060

Reverse primer  1       GCGCTCCTCGGCCTGTAG  18
Template        662353  ..................  662336
```

>CP029745.1 Pseudomonas aeruginosa strain AR_0110 chromosome, complete genome

```
product length = 312
Forward primer  1        GACTGGGTGGTGCTCGAAG  19
Template        2080563  ...................  2080581

Reverse primer  1        GCGCTCCTCGGCCTGTAG  18
Template        2080874  ..................  2080857
```

>CP023255.1 Pseudomonas aeruginosa strain CCUG 70744 chromosome, complete genome

```
product length = 312
Forward primer  1      GACTGGGTGGTGCTCGAAG  19
Template        42167  ...................  42185

Reverse primer  1      GCGCTCCTCGGCCTGTAG  18
Template        42478  ..................  42461
```

>CP029148.1 Pseudomonas aeruginosa strain AR_0440 chromosome

```
product length = 312
Forward primer  1        GACTGGGTGGTGCTCGAAG  19
Template        1200486  ...................  1200504

Reverse primer  1        GCGCTCCTCGGCCTGTAG  18
Template        1200797  ..................  1200780
```

>CP029147.1 Pseudomonas aeruginosa strain AR_0443 chromosome

```
product length = 312
Forward primer  1        GACTGGGTGGTGCTCGAAG  19
Template        5691909  ...................  5691927

Reverse primer  1        GCGCTCCTCGGCCTGTAG  18
Template        5692220  ..................  5692203
```

>CP029097.1 Pseudomonas aeruginosa strain AR439 chromosome, complete genome

```
product length = 312
Forward primer  1        GACTGGGTGGTGCTCGAAG  19
Template        5123030  ...................  5123012

Reverse primer  1        GCGCTCCTCGGCCTGTAG  18
Template        5122719  ..................  5122736
```

>CP029090.1 Pseudomonas aeruginosa strain AR442 chromosome, complete genome

```
product length = 312
Forward primer  1        GACTGGGTGGTGCTCGAAG  19
Template        1196968  ...................  1196986

Reverse primer  1        GCGCTCCTCGGCCTGTAG  18
Template        1197279  ..................  1197262
```

>CP029089.1 Pseudomonas aeruginosa strain AR444 chromosome, complete genome

```
product length = 312
Forward primer  1        GACTGGGTGGTGCTCGAAG  19
Template        3239341  ...................  3239323

Reverse primer  1        GCGCTCCTCGGCCTGTAG  18
Template        3239030  ..................  3239047
```

>CP029088.1 Pseudomonas aeruginosa strain AR445 chromosome, complete genome

```
product length = 312
Forward primer  1        GACTGGGTGGTGCTCGAAG  19
Template        2311483  ...................  2311501

Reverse primer  1        GCGCTCCTCGGCCTGTAG  18
Template        2311794  ..................  2311777
```

>CP028917.1 Pseudomonas aeruginosa strain JB2 chromosome, complete genome

```
product length = 312
Forward primer  1        GACTGGGTGGTGCTCGAAG  19
Template        5243716  ...................  5243698

Reverse primer  1        GCGCTCCTCGGCCTGTAG  18
Template        5243405  ..................  5243422
```

>CP023316.1 Pseudomonas aeruginosa strain PPF-1 chromosome, complete genome

```
product length = 312
Forward primer  1        GACTGGGTGGTGCTCGAAG  19
Template        2197047  ...................  2197065

Reverse primer  1        GCGCTCCTCGGCCTGTAG  18
Template        2197358  ..................  2197341
```

>CP028162.1 Pseudomonas aeruginosa strain MRSN12280 chromosome, complete genome

```
product length = 312
Forward primer  1        GACTGGGTGGTGCTCGAAG  19
Template        2161291  ...................  2161309

Reverse primer  1        GCGCTCCTCGGCCTGTAG  18
Template        2161602  ..................  2161585
```

>CP027538.1 Pseudomonas aeruginosa strain AR_0095 chromosome, complete genome

```
product length = 312
Forward primer  1       GACTGGGTGGTGCTCGAAG  19
Template        892896  ...................  892878

Reverse primer  1       GCGCTCCTCGGCCTGTAG  18
Template        892585  ..................  892602
```

>CP027166.1 Pseudomonas aeruginosa strain AR_0357 chromosome, complete genome

```
product length = 312
Forward primer  1        GACTGGGTGGTGCTCGAAG  19
Template        6660750  ...................  6660732

Reverse primer  1        GCGCTCCTCGGCCTGTAG  18
Template        6660439  ..................  6660456
```

>CP027174.1 Pseudomonas aeruginosa strain AR_0230 chromosome, complete genome

```
product length = 312
Forward primer  1       GACTGGGTGGTGCTCGAAG  19
Template        661982  ...................  662000

Reverse primer  1       GCGCTCCTCGGCCTGTAG  18
Template        662293  ..................  662276
```

>CP027172.1 Pseudomonas aeruginosa strain AR_0353 chromosome, complete genome

```
product length = 312
Forward primer  1        GACTGGGTGGTGCTCGAAG  19
Template        5096036  ...................  5096018

Reverse primer  1        GCGCTCCTCGGCCTGTAG  18
Template        5095725  ..................  5095742
```

>CP027171.1 Pseudomonas aeruginosa strain AR_0354 chromosome, complete genome

```
product length = 312
Forward primer  1        GACTGGGTGGTGCTCGAAG  19
Template        6716670  ...................  6716688

Reverse primer  1        GCGCTCCTCGGCCTGTAG  18
Template        6716981  ..................  6716964
```

>CP027165.1 Pseudomonas aeruginosa strain AR_0360 chromosome, complete genome

```
product length = 312
Forward primer  1        GACTGGGTGGTGCTCGAAG  19
Template        844322   ...................  844340

Reverse primer  1        GCGCTCCTCGGCCTGTAG  18
Template        844633   ..................  844616
```

>CP026680.1 Pseudomonas aeruginosa strain F5677 chromosome, complete genome

```
product length = 312
Forward primer  1        GACTGGGTGGTGCTCGAAG  19
Template        2174690  ...................  2174708

Reverse primer  1        GCGCTCCTCGGCCTGTAG  18
Template        2175001  ..................  2174984
```

>LT969520.1 Pseudomonas aeruginosa isolate RW109 genome assembly, chromosome: Main_chromosome

```
product length = 312
Forward primer  1        GACTGGGTGGTGCTCGAAG  19
Template        2277901  ...................  2277919

Reverse primer  1        GCGCTCCTCGGCCTGTAG  18
Template        2278212  ..................  2278195
```

>CP025229.1 Pseudomonas sp. AK6U chromosome, complete genome

```
product length = 312
Forward primer  1        GACTGGGTGGTGCTCGAAG  19
Template        2883633  ...................  2883615

Reverse primer  1        GCGCTCCTCGGCCTGTAG  18
Template        2883322  ..................  2883339
```

>CP025051.1 Pseudomonas aeruginosa strain PB353 chromosome, complete genome

```
product length = 312
Forward primer  1        GACTGGGTGGTGCTCGAAG  19
Template        2123495  ...................  2123513

Reverse primer  1        GCGCTCCTCGGCCTGTAG  18
Template        2123806  ..................  2123789
```

>CP025050.1 Pseudomonas aeruginosa strain PB368 chromosome, complete genome

```
product length = 312
Forward primer  1        GACTGGGTGGTGCTCGAAG  19
Template        2103304  ...................  2103322

Reverse primer  1        GCGCTCCTCGGCCTGTAG  18
Template        2103615  ..................  2103598
```

>CP025049.1 Pseudomonas aeruginosa strain PB369 chromosome, complete genome

```
product length = 312
Forward primer  1        GACTGGGTGGTGCTCGAAG  19
Template        2066495  ...................  2066513

Reverse primer  1        GCGCTCCTCGGCCTGTAG  18
Template        2066806  ..................  2066789
```

>CP025053.1 Pseudomonas aeruginosa strain PB354 chromosome, complete genome

```
product length = 312
Forward primer  1        GACTGGGTGGTGCTCGAAG  19
Template        2123495  ...................  2123513

Reverse primer  1        GCGCTCCTCGGCCTGTAG  18
Template        2123806  ..................  2123789
```

>CP024477.1 Pseudomonas aeruginosa strain 12939 chromosome, complete genome

```
product length = 312
Forward primer  1        GACTGGGTGGTGCTCGAAG  19
Template        2085809  ...................  2085827

Reverse primer  1        GCGCTCCTCGGCCTGTAG  18
Template        2086120  ..................  2086103
```

>CP017306.1 Pseudomonas aeruginosa strain PA_150577 chromosome, complete genome

```
product length = 312
Forward primer  1        GACTGGGTGGTGCTCGAAG  19
Template        2040268  ...................  2040286

Reverse primer  1        GCGCTCCTCGGCCTGTAG  18
Template        2040579  ..................  2040562
```

>CP022526.1 Pseudomonas aeruginosa strain Ocean-1155, complete genome

```
product length = 312
Forward primer  1        GACTGGGTGGTGCTCGAAG  19
Template        1215236  ...................  1215218

Reverse primer  1        GCGCTCCTCGGCCTGTAG  18
Template        1214925  ..................  1214942
```

>CP022525.1 Pseudomonas aeruginosa strain Ocean-1175, complete genome

```
product length = 312
Forward primer  1        GACTGGGTGGTGCTCGAAG  19
Template        6672445  ...................  6672427

Reverse primer  1        GCGCTCCTCGGCCTGTAG  18
Template        6672134  ..................  6672151
```

>CP019338.1 Pseudomonas aeruginosa strain L10, complete genome

```
product length = 312
Forward primer  1        GACTGGGTGGTGCTCGAAG  19
Template        2144596  ...................  2144614

Reverse primer  1        GCGCTCCTCGGCCTGTAG  18
Template        2144907  ..................  2144890
```

>CP017293.1 Pseudomonas aeruginosa strain PA83, complete genome

```
product length = 312
Forward primer  1        GACTGGGTGGTGCTCGAAG  19
Template        2132065  ...................  2132083

Reverse primer  1        GCGCTCCTCGGCCTGTAG  18
Template        2132376  ..................  2132359
```

>CP022002.1 Pseudomonas aeruginosa strain Pa1242, complete genome

```
product length = 312
Forward primer  1        GACTGGGTGGTGCTCGAAG  19
Template        5300988  ...................  5300970

Reverse primer  1        GCGCTCCTCGGCCTGTAG  18
Template        5300677  ..................  5300694
```

>CP022001.1 Pseudomonas aeruginosa strain Pa1207, complete genome

```
product length = 312
Forward primer  1        GACTGGGTGGTGCTCGAAG  19
Template        5664319  ...................  5664301

Reverse primer  1        GCGCTCCTCGGCCTGTAG  18
Template        5664008  ..................  5664025
```

>CP022000.1 Pseudomonas aeruginosa strain Pa127, complete genome

```
product length = 312
Forward primer  1        GACTGGGTGGTGCTCGAAG  19
Template        2263309  ...................  2263327

Reverse primer  1        GCGCTCCTCGGCCTGTAG  18
Template        2263620  ..................  2263603
```

>CP021999.1 Pseudomonas aeruginosa strain Pa84, complete genome

```
product length = 312
Forward primer  1        GACTGGGTGGTGCTCGAAG  19
Template        2167183  ...................  2167201

Reverse primer  1        GCGCTCCTCGGCCTGTAG  18
Template        2167494  ..................  2167477
```

>LT883143.1 Pseudomonas aeruginosa C-NN2 isolate early isolate NN2 (clone C) genome assembly, chromosome: I

```
product length = 312
Forward primer  1        GACTGGGTGGTGCTCGAAG  19
Template        2152663  ...................  2152681

Reverse primer  1        GCGCTCCTCGGCCTGTAG  18
Template        2152974  ..................  2152957
```

>CP021774.1 Pseudomonas aeruginosa strain Pa124, complete genome

```
product length = 312
Forward primer  1        GACTGGGTGGTGCTCGAAG  19
Template        2215253  ...................  2215271

Reverse primer  1        GCGCTCCTCGGCCTGTAG  18
Template        2215564  ..................  2215547
```

>CP021775.1 Pseudomonas aeruginosa strain Pa58, complete genome

```
product length = 312
Forward primer  1        GACTGGGTGGTGCTCGAAG  19
Template        2262609  ...................  2262627

Reverse primer  1        GCGCTCCTCGGCCTGTAG  18
Template        2262920  ..................  2262903
```

>CP015650.1 Pseudomonas aeruginosa strain Pb18 genome

```
product length = 312
Forward primer  1      GACTGGGTGGTGCTCGAAG  19
Template        55079  ...................  55097

Reverse primer  1      GCGCTCCTCGGCCTGTAG  18
Template        55390  ..................  55373
```

>CP015649.1 Pseudomonas aeruginosa strain M28A1 genome

```
product length = 312
Forward primer  1        GACTGGGTGGTGCTCGAAG  19
Template        5382401  ...................  5382419

Reverse primer  1        GCGCTCCTCGGCCTGTAG  18
Template        5382712  ..................  5382695
```

>CP015648.1 Pseudomonas aeruginosa strain M8A4 genome

```
product length = 312
Forward primer  1        GACTGGGTGGTGCTCGAAG  19
Template        4328431  ...................  4328413

Reverse primer  1        GCGCTCCTCGGCCTGTAG  18
```

```
Template        4328120  ..................  4328137
```

>CP015647.1 Pseudomonas aeruginosa strain M8A1 genome

```
product length = 312
Forward primer  1        GACTGGGTGGTGCTCGAAG  19
Template        2005357  ...................  2005375

Reverse primer  1        GCGCTCCTCGGCCTGTAG  18
Template        2005668  ..................  2005651
```

>CP020704.1 Pseudomonas aeruginosa strain PASGNDM699, complete genome

```
product length = 312
Forward primer  1        GACTGGGTGGTGCTCGAAG  19
Template        2170474  ...................  2170492

Reverse primer  1        GCGCTCCTCGGCCTGTAG  18
Template        2170785  ..................  2170768
```

>CP020703.1 Pseudomonas aeruginosa strain PASGNDM345, complete genome

```
product length = 312
Forward primer  1        GACTGGGTGGTGCTCGAAG  19
Template        2170468  ...................  2170486

Reverse primer  1        GCGCTCCTCGGCCTGTAG  18
Template        2170779  ..................  2170762
```

>CP008858.2 Pseudomonas aeruginosa strain F63912 chromosome, complete genome

```
product length = 312
Forward primer  1        GACTGGGTGGTGCTCGAAG  19
Template        2104644  ...................  2104662

Reverse primer  1        GCGCTCCTCGGCCTGTAG  18
Template        2104955  ..................  2104938
```

>CP020659.1 Pseudomonas aeruginosa PAK chromosome, complete genome

```
product length = 312
Forward primer  1        GACTGGGTGGTGCTCGAAG  19
Template        2785940  ...................  2785922

Reverse primer  1        GCGCTCCTCGGCCTGTAG  18
Template        2785629  ..................  2785646
```

>CP008872.2 Pseudomonas aeruginosa strain X78812 chromosome, complete genome

```
product length = 312
Forward primer  1        GACTGGGTGGTGCTCGAAG  19
Template        2063965  ...................  2063983
```

```
Reverse primer  1        GCGCTCCTCGGCCTGTAG  18
Template        2064276  ..................  2064259
```

>CP008871.2 Pseudomonas aeruginosa strain W45909 chromosome, complete genome

```
product length = 312
Forward primer  1        GACTGGGTGGTGCTCGAAG  19
Template        2123489  ...................  2123507

Reverse primer  1        GCGCTCCTCGGCCTGTAG  18
Template        2123800  ..................  2123783
```

>CP008870.2 Pseudomonas aeruginosa strain W36662 chromosome, complete genome

```
product length = 312
Forward primer  1        GACTGGGTGGTGCTCGAAG  19
Template        4152825  ...................  4152807

Reverse primer  1        GCGCTCCTCGGCCTGTAG  18
Template        4152514  ..................  4152531
```

>CP008869.2 Pseudomonas aeruginosa strain W16407 chromosome, complete genome

```
product length = 312
Forward primer  1        GACTGGGTGGTGCTCGAAG  19
Template        2166478  ...................  2166496

Reverse primer  1        GCGCTCCTCGGCCTGTAG  18
Template        2166789  ..................  2166772
```

>CP008866.2 Pseudomonas aeruginosa strain T38079 chromosome, complete genome

```
product length = 312
Forward primer  1        GACTGGGTGGTGCTCGAAG  19
Template        2054031  ...................  2054049

Reverse primer  1        GCGCTCCTCGGCCTGTAG  18
Template        2054342  ..................  2054325
```

>CP008865.2 Pseudomonas aeruginosa strain S86968 chromosome, complete genome

```
product length = 312
Forward primer  1        GACTGGGTGGTGCTCGAAG  19
Template        2139343  ...................  2139361

Reverse primer  1        GCGCTCCTCGGCCTGTAG  18
Template        2139654  ..................  2139637
```

>CP008864.2 Pseudomonas aeruginosa strain W60856 chromosome, complete genome

```
product length = 312
Forward primer  1        GACTGGGTGGTGCTCGAAG  19
Template        2661619  ...................  2661637
```

```
Reverse primer  1        GCGCTCCTCGGCCTGTAG  18
Template        2661930  ..................  2661913
```

>CP008862.2 Pseudomonas aeruginosa strain M1608 chromosome, complete genome

```
product length = 312
Forward primer  1        GACTGGGTGGTGCTCGAAG  19
Template        4238342  ...................  4238324

Reverse primer  1        GCGCTCCTCGGCCTGTAG  18
Template        4238031  ..................  4238048
```

>CP008860.2 Pseudomonas aeruginosa strain H27930 chromosome, complete genome

```
product length = 312
Forward primer  1        GACTGGGTGGTGCTCGAAG  19
Template        2096152  ...................  2096170

Reverse primer  1        GCGCTCCTCGGCCTGTAG  18
Template        2096463  ..................  2096446
```

>CP008859.2 Pseudomonas aeruginosa strain H5708 chromosome, complete genome

```
product length = 312
Forward primer  1        GACTGGGTGGTGCTCGAAG  19
Template        2057358  ...................  2057376

Reverse primer  1        GCGCTCCTCGGCCTGTAG  18
Template        2057669  ..................  2057652
```

>CP008856.2 Pseudomonas aeruginosa strain F23197 chromosome, complete genome

```
product length = 312
Forward primer  1        GACTGGGTGGTGCTCGAAG  19
Template        2055282  ...................  2055300

Reverse primer  1        GCGCTCCTCGGCCTGTAG  18
Template        2055593  ..................  2055576
```

>CP020603.1 Pseudomonas aeruginosa strain E6130952, complete genome

```
product length = 312
Forward primer  1        GACTGGGTGGTGCTCGAAG  19
Template        2098714  ...................  2098732

Reverse primer  1        GCGCTCCTCGGCCTGTAG  18
Template        2099025  ..................  2099008
```

>CP016955.1 Pseudomonas aeruginosa strain RIVM-EMC2982, complete genome

```
product length = 312
Forward primer  1        GACTGGGTGGTGCTCGAAG  19
```

```
Template        5004556  ...................  5004538

Reverse primer  1        GCGCTCCTCGGCCTGTAG  18
Template        5004245  ..................  5004262
```

>CP014866.1 Pseudomonas aeruginosa strain PA_154197 chromosome, complete genome

```
product length = 312
Forward primer  1        GACTGGGTGGTGCTCGAAG  19
Template        2095376  ...................  2095394

Reverse primer  1        GCGCTCCTCGGCCTGTAG  18
Template        2095687  ..................  2095670
```

>LT673656.1 Pseudomonas aeruginosa isolate PcyII-10 genome assembly, chromosome: PcyII-10

```
product length = 312
Forward primer  1        GACTGGGTGGTGCTCGAAG  19
Template        2038290  ...................  2038308

Reverse primer  1        GCGCTCCTCGGCCTGTAG  18
Template        2038601  ..................  2038584
```

>CP013479.1 Pseudomonas aeruginosa strain NHmuc chromosome, complete genome

```
product length = 312
Forward primer  1        GACTGGGTGGTGCTCGAAG  19
Template        3867219  ...................  3867201

Reverse primer  1        GCGCTCCTCGGCCTGTAG  18
Template        3866908  ..................  3866925
```

>CP013478.1 Pseudomonas aeruginosa strain SCVJan chromosome, complete genome

```
product length = 312
Forward primer  1        GACTGGGTGGTGCTCGAAG  19
Template        2068300  ...................  2068318

Reverse primer  1        GCGCTCCTCGGCCTGTAG  18
Template        2068611  ..................  2068594
```

>CP013477.1 Pseudomonas aeruginosa strain SCVFeb chromosome, complete genome

```
product length = 312
Forward primer  1        GACTGGGTGGTGCTCGAAG  19
Template        2068300  ...................  2068318

Reverse primer  1        GCGCTCCTCGGCCTGTAG  18
Template        2068611  ..................  2068594
```

>CP013113.1 Pseudomonas aeruginosa strain PAER4_119 chromosome, complete genome

```
product length = 312
```

```
Forward primer  1        GACTGGGTGGTGCTCGAAG  19
Template        2075113  ...................  2075131

Reverse primer  1        GCGCTCCTCGGCCTGTAG  18
Template        2075424  ..................  2075407
```

>CP017969.1 Pseudomonas aeruginosa isolate B10W chromosome, complete genome

```
product length = 312
Forward primer  1        GACTGGGTGGTGCTCGAAG  19
Template        1885727  ...................  1885745

Reverse primer  1        GCGCTCCTCGGCCTGTAG  18
Template        1886038  ..................  1886021
```

>CP014999.1 Pseudomonas aeruginosa strain PA7790, complete genome

```
product length = 312
Forward primer  1        GACTGGGTGGTGCTCGAAG  19
Template        2091972  ...................  2091990

Reverse primer  1        GCGCTCCTCGGCCTGTAG  18
Template        2092283  ..................  2092266
```

>CP015003.1 Pseudomonas aeruginosa strain PA11803 chromosome, complete genome

```
product length = 312
Forward primer  1        GACTGGGTGGTGCTCGAAG  19
Template        2228220  ...................  2228238

Reverse primer  1        GCGCTCCTCGGCCTGTAG  18
Template        2228531  ..................  2228514
```

>CP015002.1 Pseudomonas aeruginosa strain PA8281 chromosome, complete genome

```
product length = 312
Forward primer  1        GACTGGGTGGTGCTCGAAG  19
Template        2091967  ...................  2091985

Reverse primer  1        GCGCTCCTCGGCCTGTAG  18
Template        2092278  ..................  2092261
```

>CP015001.1 Pseudomonas aeruginosa strain PA1088 chromosome, complete genome

```
product length = 312
Forward primer  1        GACTGGGTGGTGCTCGAAG  19
Template        2061243  ...................  2061261

Reverse primer  1        GCGCTCCTCGGCCTGTAG  18
Template        2061554  ..................  2061537
```

>CP017353.1 Pseudomonas aeruginosa strain FA-HZ1 chromosome, complete genome

```
product length = 312
Forward primer  1        GACTGGGTGGTGCTCGAAG  19
Template        4862697  ...................  4862715

Reverse primer  1        GCGCTCCTCGGCCTGTAG  18
Template        4863008  ..................  4862991
```

>CP017149.1 Pseudomonas aeruginosa strain ATCC 15692, complete genome

```
product length = 312
Forward primer  1        GACTGGGTGGTGCTCGAAG  19
Template        2081666  ...................  2081684

Reverse primer  1        GCGCTCCTCGGCCTGTAG  18
Template        2081977  ..................  2081960
```

>CP012582.1 Pseudomonas aeruginosa strain PA_D21, complete genome

```
product length = 312
Forward primer  1        GACTGGGTGGTGCTCGAAG  19
Template        2139649  ...................  2139667

Reverse primer  1        GCGCTCCTCGGCCTGTAG  18
Template        2139960  ..................  2139943
```

>CP012579.1 Pseudomonas aeruginosa strain PA_D5, complete genome

```
product length = 312
Forward primer  1        GACTGGGTGGTGCTCGAAG  19
Template        2139650  ...................  2139668

Reverse primer  1        GCGCTCCTCGGCCTGTAG  18
Template        2139961  ..................  2139944
```

>CP017099.1 Pseudomonas aeruginosa strain DN1, complete genome

```
product length = 312
Forward primer  1        GACTGGGTGGTGCTCGAAG  19
Template        2344055  ...................  2344037

Reverse primer  1        GCGCTCCTCGGCCTGTAG  18
Template        2343744  ..................  2343761
```

>CP012584.1 Pseudomonas aeruginosa strain PA_D25, complete genome

```
product length = 312
Forward primer  1        GACTGGGTGGTGCTCGAAG  19
Template        2140887  ...................  2140905

Reverse primer  1        GCGCTCCTCGGCCTGTAG  18
Template        2141198  ..................  2141181
```

>CP012583.1 Pseudomonas aeruginosa strain PA_D22, complete genome

```
product length = 312
Forward primer  1        GACTGGGTGGTGCTCGAAG  19
Template        2139651  ...................  2139669

Reverse primer  1        GCGCTCCTCGGCCTGTAG  18
Template        2139962  ..................  2139945
```

>CP012581.1 Pseudomonas aeruginosa strain PA_D16, complete genome

```
product length = 312
Forward primer  1        GACTGGGTGGTGCTCGAAG  19
Template        2139650  ...................  2139668

Reverse primer  1        GCGCTCCTCGGCCTGTAG  18
Template        2139961  ..................  2139944
```

>CP012580.1 Pseudomonas aeruginosa strain PA_D9, complete genome

```
product length = 312
Forward primer  1        GACTGGGTGGTGCTCGAAG  19
Template        2723346  ...................  2723364

Reverse primer  1        GCGCTCCTCGGCCTGTAG  18
Template        2723657  ..................  2723640
```

>CP012578.1 Pseudomonas aeruginosa strain PA_D2, complete genome

```
product length = 312
Forward primer  1        GACTGGGTGGTGCTCGAAG  19
Template        2139650  ...................  2139668

Reverse primer  1        GCGCTCCTCGGCCTGTAG  18
Template        2139961  ..................  2139944
```

>CP012585.1 Pseudomonas aeruginosa strain PA_D1, complete genome

```
product length = 312
Forward primer  1        GACTGGGTGGTGCTCGAAG  19
Template        2139650  ...................  2139668

Reverse primer  1        GCGCTCCTCGGCCTGTAG  18
Template        2139961  ..................  2139944
```

>LT608330.1 Pseudomonas aeruginosa isolate PA14Or_reads genome assembly, chromosome: PA14OR

```
product length = 312
Forward primer  1        GACTGGGTGGTGCTCGAAG  19
Template        2135663  ...................  2135681

Reverse primer  1        GCGCTCCTCGGCCTGTAG  18
Template        2135974  ..................  2135957
```

>CP011857.1 Pseudomonas aeruginosa strain ATCC 27853, complete genome

```
product length = 312
Forward primer  1        GACTGGGTGGTGCTCGAAG  19
Template        2027472  ...................  2027490

Reverse primer  1        GCGCTCCTCGGCCTGTAG  18
Template        2027783  ..................  2027766
```

>CP016214.1 Pseudomonas aeruginosa strain PA121617, complete genome

```
product length = 312
Forward primer  1        GACTGGGTGGTGCTCGAAG  19
Template        5296989  ...................  5297007

Reverse primer  1        GCGCTCCTCGGCCTGTAG  18
Template        5297300  ..................  5297283
```

>CP015877.1 Pseudomonas aeruginosa SJTD-1 chromosome, complete genome

```
product length = 312
Forward primer  1        GACTGGGTGGTGCTCGAAG  19
Template        5225537  ...................  5225519

Reverse primer  1        GCGCTCCTCGGCCTGTAG  18
Template        5225226  ..................  5225243
```

>CP015117.1 Pseudomonas aeruginosa strain ATCC 27853 chromosome, complete genome

```
product length = 312
Forward primer  1        GACTGGGTGGTGCTCGAAG  19
Template        5618334  ...................  5618352

Reverse primer  1        GCGCTCCTCGGCCTGTAG  18
Template        5618645  ..................  5618628
```

>CP014948.1 Pseudomonas aeruginosa strain N17-1, complete genome

```
product length = 312
Forward primer  1        GACTGGGTGGTGCTCGAAG  19
Template        2058618  ...................  2058636

Reverse primer  1        GCGCTCCTCGGCCTGTAG  18
Template        2058929  ..................  2058912
```

>CP014210.1 Pseudomonas aeruginosa strain KU, partial genome

```
product length = 312
Forward primer  1        GACTGGGTGGTGCTCGAAG  19
Template        2135672  ...................  2135690

Reverse primer  1        GCGCTCCTCGGCCTGTAG  18
Template        2135983  ..................  2135966
```

>OX638701.1 Pseudomonas aeruginosa strain 4782MK genome assembly, chromosome: 4782

```
product length = 312
Forward primer  1        GACTGGGTGGTGCTCGAAG  19
Template        3504278  ...................  3504296

Reverse primer  1        GCGCTCCTCGGCCTGTAG  18
Template        3504589  ..................  3504572
```

>OX638610.1 Pseudomonas aeruginosa strain 3541 genome assembly, chromosome: 3541

```
product length = 312
Forward primer  1        GACTGGGTGGTGCTCGAAG  19
Template        5444770  ...................  5444752

Reverse primer  1        GCGCTCCTCGGCCTGTAG  18
Template        5444459  ..................  5444476
```

>OX638564.1 Pseudomonas aeruginosa strain 3796A genome assembly, chromosome: 3796A

```
product length = 312
Forward primer  1        GACTGGGTGGTGCTCGAAG  19
Template        2130966  ...................  2130984

Reverse primer  1        GCGCTCCTCGGCCTGTAG  18
Template        2131277  ..................  2131260
```

>CP008873.1 Pseudomonas aeruginosa strain F9670 chromosome, complete genome

```
product length = 312
Forward primer  1        GACTGGGTGGTGCTCGAAG  19
Template        6125272  ...................  6125254

Reverse primer  1        GCGCTCCTCGGCCTGTAG  18
Template        6124961  ..................  6124978
```

>CP013993.1 Pseudomonas aeruginosa DHS01 chromosome, complete genome

```
product length = 312
Forward primer  1        GACTGGGTGGTGCTCGAAG  19
Template        2264127  ...................  2264145

Reverse primer  1        GCGCTCCTCGGCCTGTAG  18
Template        2264438  ..................  2264421
```

>CP013989.1 Pseudomonas aeruginosa strain USDA-ARS-USMARC-41639 chromosome, complete genome

```
product length = 312
Forward primer  1        GACTGGGTGGTGCTCGAAG  19
Template        2090027  ...................  2090045

Reverse primer  1        GCGCTCCTCGGCCTGTAG  18
```

```
Template        2090338  ..................  2090321
```

>CP008868.1 Pseudomonas aeruginosa strain T63266 chromosome, complete genome

```
product length = 312
Forward primer  1        GACTGGGTGGTGCTCGAAG  19
Template        3697747  ...................  3697765

Reverse primer  1        GCGCTCCTCGGCCTGTAG  18
Template        3698058  ..................  3698041
```

>CP008867.1 Pseudomonas aeruginosa strain T52373 chromosome, complete genome

```
product length = 312
Forward primer  1        GACTGGGTGGTGCTCGAAG  19
Template        4538709  ...................  4538727

Reverse primer  1        GCGCTCCTCGGCCTGTAG  18
Template        4539020  ..................  4539003
```

>CP008863.1 Pseudomonas aeruginosa strain M37351 chromosome, complete genome

```
product length = 312
Forward primer  1        GACTGGGTGGTGCTCGAAG  19
Template        1330248  ...................  1330230

Reverse primer  1        GCGCTCCTCGGCCTGTAG  18
Template        1329937  ..................  1329954
```

>CP008857.1 Pseudomonas aeruginosa strain F30658 chromosome, complete genome

```
product length = 312
Forward primer  1        GACTGGGTGGTGCTCGAAG  19
Template        6886570  ...................  6886588

Reverse primer  1        GCGCTCCTCGGCCTGTAG  18
Template        6886881  ..................  6886864
```

>CP012901.1 Pseudomonas aeruginosa strain N15-01092 chromosome, complete genome

```
product length = 312
Forward primer  1        GACTGGGTGGTGCTCGAAG  19
Template        4761761  ...................  4761743

Reverse primer  1        GCGCTCCTCGGCCTGTAG  18
Template        4761450  ..................  4761467
```

>CP013696.1 Pseudomonas aeruginosa strain 12-4-4(59) chromosome, complete genome

```
product length = 312
Forward primer  1        GACTGGGTGGTGCTCGAAG  19
Template        3625518  ...................  3625536
```

```
Reverse primer  1        GCGCTCCTCGGCCTGTAG  18
Template        3625829  ..................  3625812
```

>CP124672.1 Pseudomonas aeruginosa strain 2022CK-00451 chromosome, complete genome

```
product length = 312
Forward primer  1        GACTGGGTGGTGCTCGAAG  19
Template        3779290  ...................  3779308

Reverse primer  1        GCGCTCCTCGGCCTGTAG  18
Template        3779601  ..................  3779584
```

>CP125367.1 Pseudomonas aeruginosa strain ZY1710 chromosome, complete genome

```
product length = 312
Forward primer  1        GACTGGGTGGTGCTCGAAG  19
Template        2125532  ...................  2125550

Reverse primer  1        GCGCTCCTCGGCCTGTAG  18
Template        2125843  ..................  2125826
```

>CP125365.1 Pseudomonas aeruginosa strain ZY36 chromosome, complete genome

```
product length = 312
Forward primer  1        GACTGGGTGGTGCTCGAAG  19
Template        2125792  ...................  2125810

Reverse primer  1        GCGCTCCTCGGCCTGTAG  18
Template        2126103  ..................  2126086
```

>CP125363.1 Pseudomonas aeruginosa strain ZY156 chromosome, complete genome

```
product length = 312
Forward primer  1        GACTGGGTGGTGCTCGAAG  19
Template        2125533  ...................  2125551

Reverse primer  1        GCGCTCCTCGGCCTGTAG  18
Template        2125844  ..................  2125827
```

>CP125361.1 Pseudomonas aeruginosa strain ZY94 chromosome, complete genome

```
product length = 312
Forward primer  1        GACTGGGTGGTGCTCGAAG  19
Template        2125527  ...................  2125545

Reverse primer  1        GCGCTCCTCGGCCTGTAG  18
Template        2125838  ..................  2125821
```

>CP125288.1 Pseudomonas aeruginosa strain SF416 chromosome, complete genome

```
product length = 312
Forward primer  1        GACTGGGTGGTGCTCGAAG  19
Template        2066921  ...................  2066939
```

```
Reverse primer  1        GCGCTCCTCGGCCTGTAG  18
Template        2067232  ..................  2067215
```

>CP013144.1 Pseudomonas aeruginosa strain Cu1510 chromosome, complete genome

```
product length = 312
Forward primer  1        GACTGGGTGGTGCTCGAAG  19
Template        2288566  ...................  2288548

Reverse primer  1        GCGCTCCTCGGCCTGTAG  18
Template        2288255  ..................  2288272
```

>AP017302.1 Pseudomonas aeruginosa DNA, complete genome, strain: IOMTU 133

```
product length = 312
Forward primer  1        GACTGGGTGGTGCTCGAAG  19
Template        2267137  ...................  2267155

Reverse primer  1        GCGCTCCTCGGCCTGTAG  18
Template        2267448  ..................  2267431
```

>CP013245.1 Pseudomonas aeruginosa strain VA-134 chromosome, complete genome

```
product length = 312
Forward primer  1       GACTGGGTGGTGCTCGAAG  19
Template        176047  ...................  176029

Reverse primer  1       GCGCTCCTCGGCCTGTAG  18
Template        175736  ..................  175753
```

>LN870292.1 Pseudomonas aeruginosa DK1 genome assembly Pseudomonas aeruginosa DK1 substr. NH57388A, chromosome : I

```
product length = 312
Forward primer  1        GACTGGGTGGTGCTCGAAG  19
Template        2069762  ...................  2069780

Reverse primer  1        GCGCTCCTCGGCCTGTAG  18
Template        2070073  ..................  2070056
```

>CP012679.1 Pseudomonas aeruginosa strain PA1RG chromosome, complete genome

```
product length = 312
Forward primer  1        GACTGGGTGGTGCTCGAAG  19
Template        2135654  ...................  2135672

Reverse primer  1        GCGCTCCTCGGCCTGTAG  18
Template        2135965  ..................  2135948
```

>CP004054.2 Pseudomonas aeruginosa PA1, complete genome

```
product length = 312
```

```
Forward primer  1        GACTGGGTGGTGCTCGAAG  19
Template        2135654  ...................  2135672

Reverse primer  1        GCGCTCCTCGGCCTGTAG  18
Template        2135965  ..................  2135948
```

>LN871187.1 Pseudomonas aeruginosa genome assembly PAO1OR, chromosome : I

```
product length = 312
Forward primer  1        GACTGGGTGGTGCTCGAAG  19
Template        2082151  ...................  2082169

Reverse primer  1        GCGCTCCTCGGCCTGTAG  18
Template        2082462  ..................  2082445
```

>AP014839.2 Pseudomonas aeruginosa DNA, complete genome, strain: 8380

```
product length = 312
Forward primer  1        GACTGGGTGGTGCTCGAAG  19
Template        2156892  ...................  2156910

Reverse primer  1        GCGCTCCTCGGCCTGTAG  18
Template        2157203  ..................  2157186
```

>CP012066.1 Pseudomonas aeruginosa strain F9676, complete genome

```
product length = 312
Forward primer  1        GACTGGGTGGTGCTCGAAG  19
Template        3835600  ...................  3835582

Reverse primer  1        GCGCTCCTCGGCCTGTAG  18
Template        3835289  ..................  3835306
```

>CP012001.1 Pseudomonas aeruginosa DSM 50071, complete genome

```
product length = 312
Forward primer  1        GACTGGGTGGTGCTCGAAG  19
Template        2050548  ...................  2050566

Reverse primer  1        GCGCTCCTCGGCCTGTAG  18
Template        2050859  ..................  2050842
```

>CP011317.1 Pseudomonas aeruginosa strain Carb01 63, complete genome

```
product length = 312
Forward primer  1        GACTGGGTGGTGCTCGAAG  19
Template        2415187  ...................  2415205

Reverse primer  1        GCGCTCCTCGGCCTGTAG  18
Template        2415498  ..................  2415481
```

>LN831024.1 Pseudomonas aeruginosa genome assembly NCTC10332, chromosome : 1

```
product length = 312
Forward primer  1        GACTGGGTGGTGCTCGAAG  19
Template        2050028  ...................  2050046

Reverse primer  1        GCGCTCCTCGGCCTGTAG  18
Template        2050339  ..................  2050322
```

>AP014651.1 Pseudomonas aeruginosa DNA, complete genome, strain: NCGM257

```
product length = 312
Forward primer  1        GACTGGGTGGTGCTCGAAG  19
Template        2236874  ...................  2236892

Reverse primer  1        GCGCTCCTCGGCCTGTAG  18
Template        2237185  ..................  2237168
```

>CP010555.1 Pseudomonas aeruginosa strain FRD1, complete genome

```
product length = 312
Forward primer  1        GACTGGGTGGTGCTCGAAG  19
Template        2079406  ...................  2079388

Reverse primer  1        GCGCTCCTCGGCCTGTAG  18
Template        2079095  ..................  2079112
```

>CP007399.1 Pseudomonas aeruginosa strain F22031, complete genome

```
product length = 312
Forward primer  1        GACTGGGTGGTGCTCGAAG  19
Template        4669029  ...................  4669047

Reverse primer  1        GCGCTCCTCGGCCTGTAG  18
Template        4669340  ..................  4669323
```

>AP014646.1 Pseudomonas aeruginosa DNA, complete genome, strain: NCGM 1984

```
product length = 312
Forward primer  1        GACTGGGTGGTGCTCGAAG  19
Template        2092834  ...................  2092852

Reverse primer  1        GCGCTCCTCGGCCTGTAG  18
Template        2093145  ..................  2093128
```

>HG974234.1 Pseudomonas aeruginosa strain PSE305, genome

```
product length = 312
Forward primer  1        GACTGGGTGGTGCTCGAAG  19
Template        3494129  ...................  3494111

Reverse primer  1        GCGCTCCTCGGCCTGTAG  18
Template        3493818  ..................  3493835
```

>CP089067.2 Pseudomonas aeruginosa strain UNC_PaerCF19 chromosome, complete genome

```
product length = 312
Forward primer  1        GACTGGGTGGTGCTCGAAG  19
Template        5602897  ...................  5602879

Reverse primer  1        GCGCTCCTCGGCCTGTAG  18
Template        5602586  ..................  5602603
```

>CP089068.2 Pseudomonas aeruginosa strain UNC_PaerCF13 chromosome, complete genome

```
product length = 312
Forward primer  1        GACTGGGTGGTGCTCGAAG  19
Template        3576103  ...................  3576121

Reverse primer  1        GCGCTCCTCGGCCTGTAG  18
Template        3576414  ..................  3576397
```

>CP089065.2 Pseudomonas aeruginosa strain UNC_PaerCF34 chromosome, complete genome

```
product length = 312
Forward primer  1        GACTGGGTGGTGCTCGAAG  19
Template        5239992  ...................  5239974

Reverse primer  1        GCGCTCCTCGGCCTGTAG  18
Template        5239681  ..................  5239698
```

>CP069331.1 Pseudomonas aeruginosa strain R09 chromosome, complete genome

```
product length = 312
Forward primer  1        GACTGGGTGGTGCTCGAAG  19
Template        4682003  ...................  4681985

Reverse primer  1        GCGCTCCTCGGCCTGTAG  18
Template        4681692  ..................  4681709
```

>CP069337.1 Pseudomonas aeruginosa strain E04 chromosome, complete genome

```
product length = 312
Forward primer  1        GACTGGGTGGTGCTCGAAG  19
Template        2085442  ...................  2085460

Reverse primer  1        GCGCTCCTCGGCCTGTAG  18
Template        2085753  ..................  2085736
```

>CP069336.1 Pseudomonas aeruginosa strain E01 chromosome, complete genome

```
product length = 312
Forward primer  1        GACTGGGTGGTGCTCGAAG  19
Template        2067892  ...................  2067910

Reverse primer  1        GCGCTCCTCGGCCTGTAG  18
Template        2068203  ..................  2068186
```

>CP069335.1 Pseudomonas aeruginosa strain E02 chromosome, complete genome

```
product length = 312
Forward primer  1        GACTGGGTGGTGCTCGAAG  19
Template        2067898  ...................  2067916

Reverse primer  1        GCGCTCCTCGGCCTGTAG  18
Template        2068209  ..................  2068192
```

>CP069334.1 Pseudomonas aeruginosa strain E03 chromosome, complete genome

```
product length = 312
Forward primer  1        GACTGGGTGGTGCTCGAAG  19
Template        2067893  ...................  2067911

Reverse primer  1        GCGCTCCTCGGCCTGTAG  18
Template        2068204  ..................  2068187
```

>CP069333.1 Pseudomonas aeruginosa strain R01 chromosome, complete genome

```
product length = 312
Forward primer  1        GACTGGGTGGTGCTCGAAG  19
Template        2067823  ...................  2067841

Reverse primer  1        GCGCTCCTCGGCCTGTAG  18
Template        2068134  ..................  2068117
```

>CP069332.1 Pseudomonas aeruginosa strain R08 chromosome, complete genome

```
product length = 312
Forward primer  1        GACTGGGTGGTGCTCGAAG  19
Template        4681698  ...................  4681680

Reverse primer  1        GCGCTCCTCGGCCTGTAG  18
Template        4681387  ..................  4681404
```

>CP069330.1 Pseudomonas aeruginosa strain R07 chromosome, complete genome

```
product length = 312
Forward primer  1        GACTGGGTGGTGCTCGAAG  19
Template        2068533  ...................  2068551

Reverse primer  1        GCGCTCCTCGGCCTGTAG  18
Template        2068844  ..................  2068827
```

>CP069329.1 Pseudomonas aeruginosa strain R10 chromosome, complete genome

```
product length = 312
Forward primer  1        GACTGGGTGGTGCTCGAAG  19
Template        4680287  ...................  4680269

Reverse primer  1        GCGCTCCTCGGCCTGTAG  18
Template        4679976  ..................  4679993
```

>CP069328.1 Pseudomonas aeruginosa strain R04 chromosome, complete genome

```
product length = 312
Forward primer  1        GACTGGGTGGTGCTCGAAG  19
Template        2067866  ...................  2067884

Reverse primer  1        GCGCTCCTCGGCCTGTAG  18
Template        2068177  ..................  2068160
```

>CP069327.1 Pseudomonas aeruginosa strain R03 chromosome, complete genome

```
product length = 312
Forward primer  1        GACTGGGTGGTGCTCGAAG  19
Template        2067843  ...................  2067861

Reverse primer  1        GCGCTCCTCGGCCTGTAG  18
Template        2068154  ..................  2068137
```

>CP069326.1 Pseudomonas aeruginosa strain R11 chromosome, complete genome

```
product length = 312
Forward primer  1        GACTGGGTGGTGCTCGAAG  19
Template        2067829  ...................  2067847

Reverse primer  1        GCGCTCCTCGGCCTGTAG  18
Template        2068140  ..................  2068123
```

>CP069325.1 Pseudomonas aeruginosa strain R02 chromosome, complete genome

```
product length = 312
Forward primer  1        GACTGGGTGGTGCTCGAAG  19
Template        4681802  ...................  4681784

Reverse primer  1        GCGCTCCTCGGCCTGTAG  18
Template        4681491  ..................  4681508
```

>CP069324.1 Pseudomonas aeruginosa strain R05 chromosome, complete genome

```
product length = 312
Forward primer  1        GACTGGGTGGTGCTCGAAG  19
Template        2067859  ...................  2067877

Reverse primer  1        GCGCTCCTCGGCCTGTAG  18
Template        2068170  ..................  2068153
```

>CP069323.1 Pseudomonas aeruginosa strain R06 chromosome, complete genome

```
product length = 312
Forward primer  1        GACTGGGTGGTGCTCGAAG  19
Template        2067427  ...................  2067445

Reverse primer  1        GCGCTCCTCGGCCTGTAG  18
Template        2067738  ..................  2067721
```

>CP068678.1 Pseudomonas aeruginosa strain NCCP15783 chromosome, complete genome

```
product length = 312
Forward primer  1        GACTGGGTGGTGCTCGAAG  19
Template        6097305  ...................  6097287

Reverse primer  1        GCGCTCCTCGGCCTGTAG  18
Template        6096994  ..................  6097011
```

>CP008739.2 Pseudomonas aeruginosa VRFPA04, complete genome

```
product length = 312
Forward primer  1        GACTGGGTGGTGCTCGAAG  19
Template        4608872  ...................  4608890

Reverse primer  1        GCGCTCCTCGGCCTGTAG  18
Template        4609183  ..................  4609166
```

>AP014622.1 Pseudomonas aeruginosa DNA, complete genome, strain: NCGM 1900

```
product length = 312
Forward primer  1        GACTGGGTGGTGCTCGAAG  19
Template        3377827  ...................  3377845

Reverse primer  1        GCGCTCCTCGGCCTGTAG  18
Template        3378138  ..................  3378121
```

>CP008749.1 Pseudomonas aeruginosa PAO1H2O genome

```
product length = 312
Forward primer  1        GACTGGGTGGTGCTCGAAG  19
Template        3433687  ...................  3433669

Reverse primer  1        GCGCTCCTCGGCCTGTAG  18
Template        3433376  ..................  3433393
```

>CP007224.1 Pseudomonas aeruginosa PA96 genome

```
product length = 312
Forward primer  1        GACTGGGTGGTGCTCGAAG  19
Template        2031750  ...................  2031768

Reverse primer  1        GCGCTCCTCGGCCTGTAG  18
Template        2032061  ..................  2032044
```

>CP006985.1 Pseudomonas aeruginosa LESlike4 sequence

```
product length = 312
Forward primer  1        GACTGGGTGGTGCTCGAAG  19
Template        2145597  ...................  2145615

Reverse primer  1        GCGCTCCTCGGCCTGTAG  18
Template        2145908  ..................  2145891
```

>CP006984.1 Pseudomonas aeruginosa LESlike1 chromosome

```
product length = 312
Forward primer  1        GACTGGGTGGTGCTCGAAG  19
Template        2161121  ...................  2161139

Reverse primer  1        GCGCTCCTCGGCCTGTAG  18
Template        2161432  ..................  2161415
```

>CP006983.1 Pseudomonas aeruginosa LESB65 sequence

```
product length = 312
Forward primer  1        GACTGGGTGGTGCTCGAAG  19
Template        2160952  ...................  2160970

Reverse primer  1        GCGCTCCTCGGCCTGTAG  18
Template        2161263  ..................  2161246
```

>CP006982.1 Pseudomonas aeruginosa LES400 sequence

```
product length = 312
Forward primer  1        GACTGGGTGGTGCTCGAAG  19
Template        2161196  ...................  2161214

Reverse primer  1        GCGCTCCTCGGCCTGTAG  18
Template        2161507  ..................  2161490
```

>CP006981.1 Pseudomonas aeruginosa LESlike7 sequence

```
product length = 312
Forward primer  1        GACTGGGTGGTGCTCGAAG  19
Template        2119311  ...................  2119329

Reverse primer  1        GCGCTCCTCGGCCTGTAG  18
Template        2119622  ..................  2119605
```

>CP006980.1 Pseudomonas aeruginosa LESlike5 sequence

```
product length = 312
Forward primer  1        GACTGGGTGGTGCTCGAAG  19
Template        2161555  ...................  2161573

Reverse primer  1        GCGCTCCTCGGCCTGTAG  18
Template        2161866  ..................  2161849
```

>CP007147.1 Pseudomonas aeruginosa YL84, complete genome

```
product length = 312
Forward primer  1        GACTGGGTGGTGCTCGAAG  19
Template        5152744  ...................  5152726

Reverse primer  1        GCGCTCCTCGGCCTGTAG  18
Template        5152433  ..................  5152450
```

>HG530068.1 Pseudomonas aeruginosa PA38182, complete genome

```
product length = 312
Forward primer  1        GACTGGGTGGTGCTCGAAG  19
Template        3564063  ...................  3564045

Reverse primer  1        GCGCTCCTCGGCCTGTAG  18
Template        3563752  ..................  3563769
```

>CP006931.1 Pseudomonas aeruginosa SCV20265, complete genome

```
product length = 312
Forward primer  1        GACTGGGTGGTGCTCGAAG  19
Template        2099295  ...................  2099313

Reverse primer  1        GCGCTCCTCGGCCTGTAG  18
Template        2099606  ..................  2099589
```

>CP006937.1 Pseudomonas aeruginosa LES431, complete genome

```
product length = 312
Forward primer  1        GACTGGGTGGTGCTCGAAG  19
Template        2119319  ...................  2119337

Reverse primer  1        GCGCTCCTCGGCCTGTAG  18
Template        2119630  ..................  2119613
```

>CP006853.1 Pseudomonas aeruginosa MTB-1, complete genome

```
product length = 312
Forward primer  1        GACTGGGTGGTGCTCGAAG  19
Template        2059218  ...................  2059236

Reverse primer  1        GCGCTCCTCGGCCTGTAG  18
Template        2059529  ..................  2059512
```

>CP004055.1 Pseudomonas aeruginosa PA1R, complete genome

```
product length = 312
Forward primer  1        GACTGGGTGGTGCTCGAAG  19
Template        1022787  ...................  1022769

Reverse primer  1        GCGCTCCTCGGCCTGTAG  18
Template        1022476  ..................  1022493
```

>CP081345.1 Pseudomonas aeruginosa strain F291007 chromosome, complete genome

```
product length = 312
Forward primer  1        GACTGGGTGGTGCTCGAAG  19
Template        2138113  ...................  2138131

Reverse primer  1        GCGCTCCTCGGCCTGTAG  18
Template        2138424  ..................  2138407
```

>CP081346.1 Pseudomonas aeruginosa strain SE5419 chromosome, complete genome

```
product length = 312
Forward primer  1        GACTGGGTGGTGCTCGAAG  19
Template        2136695  ...................  2136713

Reverse primer  1        GCGCTCCTCGGCCTGTAG  18
Template        2137006  ..................  2136989
```

>CP081287.1 Pseudomonas aeruginosa strain F092021 chromosome, complete genome

```
product length = 312
Forward primer  1        GACTGGGTGGTGCTCGAAG  19
Template        2202231  ...................  2202249

Reverse primer  1        GCGCTCCTCGGCCTGTAG  18
Template        2202542  ..................  2202525
```

>CP081202.1 Pseudomonas aeruginosa strain P9W chromosome, complete genome

```
product length = 312
Forward primer  1        GACTGGGTGGTGCTCGAAG  19
Template        3250645  ...................  3250627

Reverse primer  1        GCGCTCCTCGGCCTGTAG  18
Template        3250334  ..................  3250351
```

>CP006832.1 Pseudomonas aeruginosa PAO1-VE13 genome

```
product length = 312
Forward primer  1        GACTGGGTGGTGCTCGAAG  19
Template        3433682  ...................  3433664

Reverse primer  1        GCGCTCCTCGGCCTGTAG  18
Template        3433371  ..................  3433388
```

>CP006831.1 Pseudomonas aeruginosa PAO1-VE2 genome

```
product length = 312
Forward primer  1        GACTGGGTGGTGCTCGAAG  19
Template        3433682  ...................  3433664

Reverse primer  1        GCGCTCCTCGGCCTGTAG  18
Template        3433371  ..................  3433388
```

>CP006705.1 Pseudomonas aeruginosa PAO581 genome

```
product length = 312
Forward primer  1        GACTGGGTGGTGCTCGAAG  19
Template        3213268  ...................  3213250

Reverse primer  1        GCGCTCCTCGGCCTGTAG  18
Template        3212957  ..................  3212974
```

>CP006728.1 Pseudomonas aeruginosa c7447m genome

```
product length = 312
Forward primer  1        GACTGGGTGGTGCTCGAAG  19
Template        3432115  ...................  3432097

Reverse primer  1        GCGCTCCTCGGCCTGTAG  18
Template        3431804  ..................  3431821
```

>CP006245.1 Pseudomonas aeruginosa RP73, complete genome

```
product length = 312
Forward primer  1        GACTGGGTGGTGCTCGAAG  19
Template        3519302  ...................  3519284

Reverse primer  1        GCGCTCCTCGGCCTGTAG  18
Template        3518991  ..................  3519008
```

>CP074424.1 Pseudomonas aeruginosa strain 88A chromosome

```
product length = 312
Forward primer  1        GACTGGGTGGTGCTCGAAG  19
Template        5964203  ...................  5964221

Reverse primer  1        GCGCTCCTCGGCCTGTAG  18
Template        5964514  ..................  5964497
```

>CP061780.1 Pseudomonas aeruginosa strain ZBX-P11 chromosome, complete genome

```
product length = 312
Forward primer  1        GACTGGGTGGTGCTCGAAG  19
Template        4716810  ...................  4716828

Reverse primer  1        GCGCTCCTCGGCCTGTAG  18
Template        4717121  ..................  4717104
```

>CP061779.1 Pseudomonas aeruginosa strain ZBX-P12 chromosome, complete genome

```
product length = 312
Forward primer  1        GACTGGGTGGTGCTCGAAG  19
Template        1130364  ...................  1130382

Reverse primer  1        GCGCTCCTCGGCCTGTAG  18
Template        1130675  ..................  1130658
```

>CP061778.1 Pseudomonas aeruginosa strain ZBX-P13 chromosome, complete genome

```
product length = 312
Forward primer  1        GACTGGGTGGTGCTCGAAG  19
Template        3776739  ...................  3776757

Reverse primer  1        GCGCTCCTCGGCCTGTAG  18
Template        3777050  ..................  3777033
```

>CP061777.1 Pseudomonas aeruginosa strain ZBX-P23 chromosome, complete genome

```
product length = 312
Forward primer  1        GACTGGGTGGTGCTCGAAG  19
Template        1885905  ...................  1885923

Reverse primer  1        GCGCTCCTCGGCCTGTAG  18
Template        1886216  ..................  1886199
```

>CP004061.1 Pseudomonas aeruginosa B136-33, complete genome

```
product length = 312
Forward primer  1        GACTGGGTGGTGCTCGAAG  19
Template        2047466  ...................  2047484

Reverse primer  1        GCGCTCCTCGGCCTGTAG  18
Template        2047777  ..................  2047760
```

>CP070471.1 Pseudomonas aeruginosa strain B17932 chromosome, complete genome

```
product length = 312
Forward primer  1        GACTGGGTGGTGCTCGAAG  19
Template        4738774  ...................  4738756

Reverse primer  1        GCGCTCCTCGGCCTGTAG  18
Template        4738463  ..................  4738480
```

>CP070467.1 Pseudomonas aeruginosa strain B17416 chromosome, complete genome

```
product length = 312
Forward primer  1        GACTGGGTGGTGCTCGAAG  19
Template        4977414  ...................  4977396

Reverse primer  1        GCGCTCCTCGGCCTGTAG  18
Template        4977103  ..................  4977120
```

>CP070355.1 Pseudomonas aeruginosa strain PDNC003 chromosome

```
product length = 312
Forward primer  1       GACTGGGTGGTGCTCGAAG  19
Template        825765  ...................  825783

Reverse primer  1       GCGCTCCTCGGCCTGTAG  18
Template        826076  ..................  826059
```

>CP003149.1 Pseudomonas aeruginosa DK2, complete genome

```
product length = 312
Forward primer  1        GACTGGGTGGTGCTCGAAG  19
Template        1955562  ...................  1955580

Reverse primer  1        GCGCTCCTCGGCCTGTAG  18
Template        1955873  ..................  1955856
```

>CP063237.1 Pseudomonas aeruginosa strain mPA08-31 chromosome

```
product length = 312
Forward primer  1        GACTGGGTGGTGCTCGAAG  19
Template        3492115  ...................  3492097

Reverse primer  1        GCGCTCCTCGGCCTGTAG  18
Template        3491804  ..................  3491821
```

>CP063047.1 Pseudomonas aeruginosa strain KC-Tt-1 chromosome, complete genome

```
product length = 312
Forward primer  1        GACTGGGTGGTGCTCGAAG  19
Template        1328178  ...................  1328196

Reverse primer  1        GCGCTCCTCGGCCTGTAG  18
Template        1328489  ..................  1328472
```

>CP062219.1 Pseudomonas aeruginosa strain JT86 chromosome, complete genome

```
product length = 312
Forward primer  1       GACTGGGTGGTGCTCGAAG  19
Template        333064  ...................  333082

Reverse primer  1       GCGCTCCTCGGCCTGTAG  18
Template        333375  ..................  333358
```

>CP061850.1 Pseudomonas aeruginosa strain R31 chromosome, complete genome

```
product length = 312
Forward primer  1        GACTGGGTGGTGCTCGAAG  19
Template        1381435  ...................  1381453

Reverse primer  1        GCGCTCCTCGGCCTGTAG  18
Template        1381746  ..................  1381729
```

>CP058257.1 Pseudomonas aeruginosa strain PA179 chromosome

```
product length = 312
Forward primer  1        GACTGGGTGGTGCTCGAAG  19
Template        3557680  ...................  3557662

Reverse primer  1        GCGCTCCTCGGCCTGTAG  18
Template        3557369  ..................  3557386
```

>CP059852.1 Pseudomonas aeruginosa strain ZM03 chromosome, complete genome

```
product length = 312
Forward primer  1        GACTGGGTGGTGCTCGAAG  19
Template        2053856  ...................  2053838

Reverse primer  1        GCGCTCCTCGGCCTGTAG  18
Template        2053545  ..................  2053562
```

>AP012280.1 Pseudomonas aeruginosa NCGM2.S1 DNA, complete genome

```
product length = 312
Forward primer  1        GACTGGGTGGTGCTCGAAG  19
Template        4511981  ...................  4511963

Reverse primer  1        GCGCTCCTCGGCCTGTAG  18
Template        4511670  ..................  4511687
```

>CP002496.1 Pseudomonas aeruginosa M18, complete genome

```
product length = 312
Forward primer  1        GACTGGGTGGTGCTCGAAG  19
Template        2084487  ...................  2084505

Reverse primer  1        GCGCTCCTCGGCCTGTAG  18
Template        2084798  ..................  2084781
```

>FM209186.1 Pseudomonas aeruginosa LESB58 complete genome sequence

```
product length = 312
Forward primer  1        GACTGGGTGGTGCTCGAAG  19
Template        2161496  ...................  2161514

Reverse primer  1        GCGCTCCTCGGCCTGTAG  18
Template        2161807  ..................  2161790
```

>CP000438.1 Pseudomonas aeruginosa UCBPP-PA14, complete genome

```
product length = 312
Forward primer  1        GACTGGGTGGTGCTCGAAG  19
Template        2135672  ...................  2135690

Reverse primer  1        GCGCTCCTCGGCCTGTAG  18
Template        2135983  ..................  2135966
```

>AE004091.2 Pseudomonas aeruginosa PAO1, complete genome

```
product length = 312
Forward primer  1        GACTGGGTGGTGCTCGAAG  19
Template        3433687  ...................  3433669

Reverse primer  1        GCGCTCCTCGGCCTGTAG  18
Template        3433376  ..................  3433393
```

>CP109757.1 Pseudomonas aeruginosa strain 2017-45-85 chromosome, complete genome

```
product length = 312
Forward primer  1        GACTGGGTGGTGCTCGAAG  19
Template        2010302  .................G.  2010320

Reverse primer  1        GCGCTCCTCGGCCTGTAG  18
Template        2010613  ..................  2010596
```

>CP109683.1 Pseudomonas aeruginosa strain 2017-45-169 chromosome, complete genome

```
product length = 312
Forward primer  1       GACTGGGTGGTGCTCGAAG  19
Template        898805  .................G.  898787

Reverse primer  1       GCGCTCCTCGGCCTGTAG  18
Template        898494  ..................  898511
```

>CP124638.1 Pseudomonas aeruginosa strain 2021CK-01158 chromosome, complete genome

```
product length = 312
Forward primer  1        GACTGGGTGGTGCTCGAAG  19
Template        4156638  .................G.  4156620

Reverse primer  1        GCGCTCCTCGGCCTGTAG  18
Template        4156327  ..................  4156344
```

>CP124624.1 Pseudomonas aeruginosa strain 2021CK-01157 chromosome, complete genome

```
product length = 312
Forward primer  1        GACTGGGTGGTGCTCGAAG  19
Template        2134777  .................G.  2134795

Reverse primer  1        GCGCTCCTCGGCCTGTAG  18
Template        2135088  ..................  2135071
```

>CP124641.1 Pseudomonas aeruginosa strain 2021CK-01198 chromosome, complete genome

```
product length = 312
Forward primer  1        GACTGGGTGGTGCTCGAAG  19
Template        2134793  .................G.  2134811

Reverse primer  1        GCGCTCCTCGGCCTGTAG  18
Template        2135104  ..................  2135087
```

>CP117749.1 Pseudomonas aeruginosa strain 2022CK-00828 chromosome, complete genome

```
product length = 312
Forward primer  1        GACTGGGTGGTGCTCGAAG  19
Template        2116257  .................G.  2116275

Reverse primer  1        GCGCTCCTCGGCCTGTAG  18
Template        2116568  ..................  2116551
```

>CP075834.1 Pseudomonas aeruginosa strain PaLo15 chromosome, complete genome

```
product length = 312
Forward primer  1        GACTGGGTGGTGCTCGAAG  19
Template        4724522  .................G.  4724504

Reverse primer  1        GCGCTCCTCGGCCTGTAG  18
Template        4724211  ..................  4724228
```

>CP075829.1 Pseudomonas aeruginosa strain PaLo25 chromosome, complete genome

```
product length = 312
Forward primer  1        GACTGGGTGGTGCTCGAAG  19
Template        2038587  .................G.  2038605

Reverse primer  1        GCGCTCCTCGGCCTGTAG  18
Template        2038898  ..................  2038881
```

>CP075811.1 Pseudomonas aeruginosa strain PaLo152 chromosome, complete genome

```
product length = 312
Forward primer  1        GACTGGGTGGTGCTCGAAG  19
Template        2108023  .................G.  2108041

Reverse primer  1        GCGCTCCTCGGCCTGTAG  18
Template        2108334  ..................  2108317
```

>CP075797.1 Pseudomonas aeruginosa strain PaLo326 chromosome, complete genome

```
product length = 312
Forward primer  1        GACTGGGTGGTGCTCGAAG  19
Template        2067183  .................G.  2067201

Reverse primer  1        GCGCTCCTCGGCCTGTAG  18
Template        2067494  ..................  2067477
```

>CP075794.1 Pseudomonas aeruginosa strain PaLo418 chromosome, complete genome

```
product length = 312
Forward primer  1        GACTGGGTGGTGCTCGAAG  19
Template        2071634  .................G.  2071652

Reverse primer  1        GCGCTCCTCGGCCTGTAG  18
Template        2071945  ..................  2071928
```

>CP075793.1 Pseudomonas aeruginosa strain PaLo419 chromosome, complete genome

```
product length = 312
Forward primer  1        GACTGGGTGGTGCTCGAAG  19
Template        2071515  .................G.  2071533

Reverse primer  1        GCGCTCCTCGGCCTGTAG  18
Template        2071826  ..................  2071809
```

>CP075792.1 Pseudomonas aeruginosa strain PaLo422 chromosome, complete genome

```
product length = 312
Forward primer  1        GACTGGGTGGTGCTCGAAG  19
Template        2161789  .................G.  2161807

Reverse primer  1        GCGCTCCTCGGCCTGTAG  18
Template        2162100  ..................  2162083
```

>CP075788.1 Pseudomonas aeruginosa strain PaLo502 chromosome, complete genome

```
product length = 312
Forward primer  1        GACTGGGTGGTGCTCGAAG  19
Template        4121750  .................G.  4121732

Reverse primer  1        GCGCTCCTCGGCCTGTAG  18
Template        4121439  ..................  4121456
```

>CP110347.1 Pseudomonas aeruginosa strain PALA52 chromosome, complete genome

```
product length = 312
Forward primer  1        GACTGGGTGGTGCTCGAAG  19
Template        2049940  .................G.  2049958

Reverse primer  1        GCGCTCCTCGGCCTGTAG  18
Template        2050251  ..................  2050234
```

>CP097560.1 Pseudomonas aeruginosa strain C4.2 chromosome, complete genome

```
product length = 312
Forward primer  1        GACTGGGTGGTGCTCGAAG  19
Template        2196994  .................G.  2197012

Reverse primer  1        GCGCTCCTCGGCCTGTAG  18
Template        2197305  ..................  2197288
```

>CP109657.1 Pseudomonas aeruginosa strain Zw26 chromosome, complete genome

```
product length = 312
Forward primer  1        GACTGGGTGGTGCTCGAAG  19
Template        2025697  .................G.  2025715

Reverse primer  1        GCGCTCCTCGGCCTGTAG  18
Template        2026008  ..................  2025991
```

>CP100759.1 Pseudomonas aeruginosa strain PA0009 chromosome

```
product length = 312
Forward primer  1        GACTGGGTGGTGCTCGAAG  19
Template        5192513  .................G.  5192495

Reverse primer  1        GCGCTCCTCGGCCTGTAG  18
Template        5192202  ..................  5192219
```

>CP093028.1 Pseudomonas aeruginosa strain H05 chromosome, complete genome

```
product length = 312
Forward primer  1        GACTGGGTGGTGCTCGAAG  19
Template        2105779  .................G.  2105797

Reverse primer  1        GCGCTCCTCGGCCTGTAG  18
Template        2106090  ..................  2106073
```

>CP093021.1 Pseudomonas aeruginosa strain H09 chromosome, complete genome

```
product length = 312
Forward primer  1        GACTGGGTGGTGCTCGAAG  19
Template        2062934  ..................G.  2062952

Reverse primer  1        GCGCTCCTCGGCCTGTAG  18
Template        2063245  ..................  2063228
```

>CP093015.1 Pseudomonas aeruginosa strain H16 chromosome, complete genome

```
product length = 312
Forward primer  1        GACTGGGTGGTGCTCGAAG  19
Template        2124214  ..................G.  2124232

Reverse primer  1        GCGCTCCTCGGCCTGTAG  18
Template        2124525  ..................  2124508
```

>CP089238.1 Pseudomonas aeruginosa strain JNQH-PA033 chromosome, complete genome

```
product length = 312
Forward primer  1        GACTGGGTGGTGCTCGAAG  19
Template        2103218  ..................G.  2103236

Reverse primer  1        GCGCTCCTCGGCCTGTAG  18
Template        2103529  ..................  2103512
```

>CP086016.1 Pseudomonas aeruginosa isolate KB-PA_3 chromosome, complete genome

```
product length = 312
Forward primer  1        GACTGGGTGGTGCTCGAAG  19
Template        4827753  ..................G.  4827735

Reverse primer  1        GCGCTCCTCGGCCTGTAG  18
Template        4827442  ..................  4827459
```

>CP084484.1 Pseudomonas sp. PS1(2021) chromosome, complete genome

```
product length = 312
Forward primer  1       GACTGGGTGGTGCTCGAAG  19
Template        681669  ..................G.  681687

Reverse primer  1       GCGCTCCTCGGCCTGTAG  18
Template        681980  ..................  681963
```

>CP083366.1 Pseudomonas aeruginosa strain PS1793 chromosome, complete genome

```
product length = 312
Forward primer  1        GACTGGGTGGTGCTCGAAG  19
Template        2157511  ..................G.  2157529

Reverse primer  1        GCGCTCCTCGGCCTGTAG  18
Template        2157822  ..................  2157805
```

>CP080286.1 Pseudomonas aeruginosa strain UNC_PaerCF12 chromosome, complete genome

```
product length = 312
Forward primer  1        GACTGGGTGGTGCTCGAAG  19
Template        3373485  .................G.  3373503

Reverse primer  1        GCGCTCCTCGGCCTGTAG  18
Template        3373796  ..................  3373779
```

>CP072783.1 Pseudomonas aeruginosa strain LICME WGH-6 chromosome, complete genome

```
product length = 312
Forward primer  1        GACTGGGTGGTGCTCGAAG  19
Template        4282034  .................G.  4282016

Reverse primer  1        GCGCTCCTCGGCCTGTAG  18
Template        4281723  ..................  4281740
```

>CP059995.1 Pseudomonas aeruginosa strain NY3045 chromosome, complete genome

```
product length = 312
Forward primer  1        GACTGGGTGGTGCTCGAAG  19
Template        2224727  .................G.  2224745

Reverse primer  1        GCGCTCCTCGGCCTGTAG  18
Template        2225038  ..................  2225021
```

>CP056089.1 Pseudomonas aeruginosa strain PABCH46 chromosome

```
product length = 312
Forward primer  1        GACTGGGTGGTGCTCGAAG  19
Template        4803415  .................G.  4803397

Reverse primer  1        GCGCTCCTCGGCCTGTAG  18
Template        4803104  ..................  4803121
```

>CP046602.1 Pseudomonas aeruginosa strain CMC-115 chromosome, complete genome

```
product length = 312
Forward primer  1        GACTGGGTGGTGCTCGAAG  19
Template        3492049  .................G.  3492031

Reverse primer  1        GCGCTCCTCGGCCTGTAG  18
Template        3491738  ..................  3491755
```

>CP041354.1 Pseudomonas aeruginosa strain AZPAE15042 chromosome, complete genome

```
product length = 312
Forward primer  1        GACTGGGTGGTGCTCGAAG  19
Template        2024174  .................G.  2024192

Reverse primer  1        GCGCTCCTCGGCCTGTAG  18
Template        2024485  ..................  2024468
```

>CP039293.1 Pseudomonas aeruginosa strain PABL048 chromosome, complete genome

```
product length = 312
Forward primer  1        GACTGGGTGGTGCTCGAAG  19
Template        2157374  .................G.  2157392

Reverse primer  1        GCGCTCCTCGGCCTGTAG  18
Template        2157685  ..................  2157668
```

>CP028368.1 Pseudomonas aeruginosa strain PA-VAP-4 chromosome

```
product length = 312
Forward primer  1        GACTGGGTGGTGCTCGAAG  19
Template        5706400  .................G.  5706382

Reverse primer  1        GCGCTCCTCGGCCTGTAG  18
Template        5706089  ..................  5706106
```

>CP033835.1 Pseudomonas aeruginosa strain FDAARGOS_570 chromosome, complete genome

```
product length = 312
Forward primer  1        GACTGGGTGGTGCTCGAAG  19
Template        4990775  .................G.  4990757

Reverse primer  1        GCGCTCCTCGGCCTGTAG  18
Template        4990464  ..................  4990481
```

>CP030328.1 Pseudomonas aeruginosa strain AR_455 chromosome, complete genome

```
product length = 312
Forward primer  1        GACTGGGTGGTGCTCGAAG  19
Template        3611369  .................G.  3611387

Reverse primer  1        GCGCTCCTCGGCCTGTAG  18
Template        3611680  ..................  3611663
```

>LS483497.1 Pseudomonas aeruginosa strain NCTC9433 genome assembly, chromosome: 1

```
product length = 312
Forward primer  1        GACTGGGTGGTGCTCGAAG  19
Template        2073240  .................G.  2073258

Reverse primer  1        GCGCTCCTCGGCCTGTAG  18
Template        2073551  ..................  2073534
```

>CP020560.1 Pseudomonas paraeruginosa strain Cr1 chromosome, complete genome

```
product length = 312
Forward primer  1        GACTGGGTGGTGCTCGAAG  19
Template        2029462  .................G.  2029480

Reverse primer  1        GCGCTCCTCGGCCTGTAG  18
Template        2029773  ..................  2029756
```

>CP008861.1 Pseudomonas aeruginosa strain H47921 chromosome, complete genome

```
product length = 312
Forward primer  1        GACTGGGTGGTGCTCGAAG  19
Template        1599802  .................G.  1599784

Reverse primer  1        GCGCTCCTCGGCCTGTAG  18
Template        1599491  ..................  1599508
```

>CP011369.1 Pseudomonas aeruginosa strain S04 90 chromosome

```
product length = 312
Forward primer  1        GACTGGGTGGTGCTCGAAG  19
Template        2151115  .................G.  2151133

Reverse primer  1        GCGCTCCTCGGCCTGTAG  18
Template        2151426  ..................  2151409
```

>CP068238.1 Pseudomonas aeruginosa strain A39-1 chromosome, complete genome

```
product length = 312
Forward primer  1        GACTGGGTGGTGCTCGAAG  19
Template        1999668  .................G.  1999686

Reverse primer  1        GCGCTCCTCGGCCTGTAG  18
Template        1999979  ..................  1999962
```

>CP000744.1 Pseudomonas aeruginosa PA7, complete genome

```
product length = 312
Forward primer  1        GACTGGGTGGTGCTCGAAG  19
Template        2121203  .................G.  2121221

Reverse primer  1        GCGCTCCTCGGCCTGTAG  18
Template        2121514  ..................  2121497
```

If you want to allow any of the unintended targets, check the box(es) next to the ones you accept and try again to re-search for specific primers Submit

Help

FOLLOW NCBI

Connect with NLM

National Library of Medicine
8600 Rockville Pike
Bethesda, MD 20894

Web Policies
FOIA
HHS Vulnerability Disclosure

Help
Accessibility
Careers

NLM NIH HHS USA.gov
