# Supplementary material for: Phylogenetic Aspects of Antibiotic Resistance and Biofilm Formation of P. aeruginosa Isolated from Clinical Samples
Source: Can J Infect Dis Med Microbiol. 2024 Jan 13;2024:6213873. doi: 10.1155/2024/6213873 (PMC10799695; doi:10.1155/2024/6213873)
Supplement: Supplementary Materials — Original pictures and primer-blast results. [file 6213873.f1.zip › pslA Primer-Blast results.pdf]

An official website of the United States government

Here's how you know

The .gov means it’s official.
Federal government websites often end in .gov or .mil. Before sharing sensitive information, make sure you’re on a federal government site.

The site is secure.
The https:// ensures that you are connecting to the official website and that any information you provide is encrypted and transmitted securely.

Skip to main page content

Access keys NCBI Homepage MyNCBI Homepage Main Content Main Navigation

Log in

Primer-BLAST

» JOB ID:AgjdnXWveAdfOWI8b1xGDhVHVzw4VEwhOQ

PrimerBLAST users!
We want to hear from you about how PrimerBLAST can be improved.
Contact us

Primer-BLAST Results
Help

•

Input PCR template
none

Specificity of primers
Target templates were found in selected database: Nucleotide collection (nt)

Other reports
Search Summary

Detailed primer reports

You can re-search for specific primers by accepting some of the unintended targets, check the box(es) next to the ones you accept and try again to re-search for specific primers Submit
Help

Primer pair 1

	Sequence (5'->3')	Length	Tm	GC%	Self complementarity	Self 3' complementarity
Forward primer	TGCCTGGAACATAATCACCGT	21	59.72	47.62	3.00	3.00
Reverse primer	GTCGGTAGATAGCCTGTCGC	20	60.04	60.00	5.00	2.00

Products on intended targets

Products on allowed targets

Products on allowed transcript variants

Products on potentially unintended templates

Products on target templates

>CP075841.1 Pseudomonas aeruginosa strain PaLo9 chromosome, complete genome

```
product length = 202
Forward primer  1        TGCCTGGAACATAATCACCGT  21
Template        3492158  .....................  3492178

Reverse primer  1        GTCGGTAGATAGCCTGTCGC  20
Template        3492359  ....................  3492340
```

>CP075800.1 Pseudomonas aeruginosa strain PaLo297 chromosome, complete genome

```
product length = 202
Forward primer  1        TGCCTGGAACATAATCACCGT  21
Template        3121196  .....................  3121216

Reverse primer  1        GTCGGTAGATAGCCTGTCGC  20
Template        3121397  ....................  3121378
```

>CP075765.1 Pseudomonas aeruginosa strain PaLo539 chromosome, complete genome

```
product length = 202
Forward primer  1        TGCCTGGAACATAATCACCGT  21
Template        3204485  .....................  3204505

Reverse primer  1        GTCGGTAGATAGCCTGTCGC  20
Template        3204686  ....................  3204667
```

>CP075762.1 Pseudomonas aeruginosa strain PaLo544 chromosome, complete genome

```
product length = 202
Forward primer  1        TGCCTGGAACATAATCACCGT  21
Template        3158582  .....................  3158602

Reverse primer  1        GTCGGTAGATAGCCTGTCGC  20
Template        3158783  ....................  3158764
```

>CP109932.1 Pseudomonas aeruginosa strain PALA43 chromosome, complete genome

```
product length = 202
Forward primer  1        TGCCTGGAACATAATCACCGT  21
Template        4157199  .....................  4157219

Reverse primer  1        GTCGGTAGATAGCCTGTCGC  20
Template        4157400  ....................  4157381
```

>CP107275.1 Pseudomonas aeruginosa strain PALA22 chromosome, complete genome

```
product length = 202
Forward primer  1        TGCCTGGAACATAATCACCGT  21
Template        4109609  .....................  4109589

Reverse primer  1        GTCGGTAGATAGCCTGTCGC  20
Template        4109408  ....................  4109427
```

>CP036492.1 Pseudomonas aeruginosa strain Paer4 chromosome, complete genome

```
product length = 202
Forward primer  1        TGCCTGGAACATAATCACCGT  21
Template        3215561  .....................  3215541

Reverse primer  1        GTCGGTAGATAGCCTGTCGC  20
Template        3215360  ....................  3215379
```

>CP104913.1 Pseudomonas aeruginosa strain PA-AUTBAM chromosome, complete genome

```
product length = 202
Forward primer  1        TGCCTGGAACATAATCACCGT  21
Template        2453879  .....................  2453859

Reverse primer  1        GTCGGTAGATAGCCTGTCGC  20
Template        2453678  ....................  2453697
```

>CP104720.1 Pseudomonas aeruginosa strain NY4593 chromosome, complete genome

```
product length = 202
Forward primer  1        TGCCTGGAACATAATCACCGT  21
Template        2851058  .....................  2851078

Reverse primer  1        GTCGGTAGATAGCCTGTCGC  20
Template        2851259  ....................  2851240
```

>CP092972.1 Pseudomonas aeruginosa strain PA01135 chromosome, complete genome

```
product length = 202
Forward primer  1        TGCCTGGAACATAATCACCGT  21
Template        3098373  .....................  3098353

Reverse primer  1        GTCGGTAGATAGCCTGTCGC  20
Template        3098172  ....................  3098191
```

>CP079758.1 Pseudomonas aeruginosa strain PA0386 chromosome, complete genome

```
product length = 202
Forward primer  1        TGCCTGGAACATAATCACCGT  21
Template        3098422  .....................  3098402

Reverse primer  1        GTCGGTAGATAGCCTGTCGC  20
Template        3098221  ....................  3098240
```

>CP079757.1 Pseudomonas aeruginosa strain PA0200 chromosome, complete genome

```
product length = 202
Forward primer  1        TGCCTGGAACATAATCACCGT  21
Template        2449723  .....................  2449703

Reverse primer  1        GTCGGTAGATAGCCTGTCGC  20
Template        2449522  ....................  2449541
```

>CP104301.1 Pseudomonas aeruginosa strain PLL01 chromosome, complete genome

```
product length = 202
Forward primer  1        TGCCTGGAACATAATCACCGT  21
Template        2453311  .....................  2453291

Reverse primer  1        GTCGGTAGATAGCCTGTCGC  20
Template        2453110  ....................  2453129
```

>CP079712.1 Pseudomonas aeruginosa strain PAO1-UW chromosome, complete genome

```
product length = 202
Forward primer  1        TGCCTGGAACATAATCACCGT  21
Template        3061336  .....................  3061356

Reverse primer  1        GTCGGTAGATAGCCTGTCGC  20
Template        3061537  ....................  3061518
```

>CP085082.1 Pseudomonas aeruginosa strain PAO1-Holloway chromosome, complete genome

```
product length = 202
Forward primer  1        TGCCTGGAACATAATCACCGT  21
Template        2453673  .....................  2453653

Reverse primer  1        GTCGGTAGATAGCCTGTCGC  20
Template        2453472  ....................  2453491
```

>CP103307.1 Pseudomonas aeruginosa strain PLL01 chromosome, complete genome

```
product length = 202
Forward primer  1        TGCCTGGAACATAATCACCGT  21
Template        2450086  .....................  2450066

Reverse primer  1        GTCGGTAGATAGCCTGTCGC  20
Template        2449885  ....................  2449904
```

>CP101656.1 Pseudomonas aeruginosa strain L1a chromosome, complete genome

```
product length = 202
Forward primer  1        TGCCTGGAACATAATCACCGT  21
Template        4062287  .....................  4062307

Reverse primer  1        GTCGGTAGATAGCCTGTCGC  20
Template        4062488  ....................  4062469
```

>CP099798.1 Pseudomonas aeruginosa strain PAO1-L chromosome, complete genome

```
product length = 202
Forward primer  1        TGCCTGGAACATAATCACCGT  21
Template        3061956  .....................  3061976

Reverse primer  1        GTCGGTAGATAGCCTGTCGC  20
Template        3062157  ....................  3062138
```

>CP099797.1 Pseudomonas aeruginosa strain PAO1-N chromosome, complete genome

```
product length = 202
Forward primer  1        TGCCTGGAACATAATCACCGT  21
Template        3061981  .....................  3062001

Reverse primer  1        GTCGGTAGATAGCCTGTCGC  20
Template        3062182  ....................  3062163
```

>CP096665.1 Pseudomonas aeruginosa strain PAO1_Mat-X-1 chromosome, complete genome

```
product length = 202
Forward primer  1        TGCCTGGAACATAATCACCGT  21
Template        2205342  .....................  2205322

Reverse primer  1        GTCGGTAGATAGCCTGTCGC  20
Template        2205141  ....................  2205160
```

>CP096664.1 Pseudomonas aeruginosa strain PAO1_Kat-X-2 chromosome, complete genome

```
product length = 202
Forward primer  1        TGCCTGGAACATAATCACCGT  21
Template        2106353  .....................  2106333

Reverse primer  1        GTCGGTAGATAGCCTGTCGC  20
Template        2106152  ....................  2106171
```

>CP064391.1 Pseudomonas aeruginosa strain PartH-Paeruginosa-RM8376 chromosome, complete genome

```
product length = 202
Forward primer  1        TGCCTGGAACATAATCACCGT  21
Template        2011049  .....................  2011069

Reverse primer  1        GTCGGTAGATAGCCTGTCGC  20
Template        2011250  ....................  2011231
```

>CP086122.1 Pseudomonas aeruginosa strain MIN-155 chromosome, complete genome

```
product length = 202
Forward primer  1        TGCCTGGAACATAATCACCGT  21
Template        3061173  .....................  3061193

Reverse primer  1        GTCGGTAGATAGCCTGTCGC  20
Template        3061374  ....................  3061355
```

>CP089064.2 Pseudomonas aeruginosa strain UNC_PaerCF35 chromosome, complete genome

```
product length = 202
Forward primer  1        TGCCTGGAACATAATCACCGT  21
Template        5934681  .....................  5934701

Reverse primer  1        GTCGGTAGATAGCCTGTCGC  20
Template        5934882  ....................  5934863
```

>CP064399.1 Pseudomonas aeruginosa strain QZPH41 chromosome, complete genome

```
product length = 202
Forward primer  1        TGCCTGGAACATAATCACCGT  21
Template        3127491  .....................  3127511

Reverse primer  1        GTCGGTAGATAGCCTGTCGC  20
```

```
Template        3127692  ....................  3127673
```

>CP061779.1 Pseudomonas aeruginosa strain ZBX-P12 chromosome, complete genome

```
product length = 202
Forward primer  1        TGCCTGGAACATAATCACCGT  21
Template        2339488  .....................  2339508

Reverse primer  1        GTCGGTAGATAGCCTGTCGC  20
Template        2339689  ....................  2339670
```

>CP061777.1 Pseudomonas aeruginosa strain ZBX-P23 chromosome, complete genome

```
product length = 202
Forward primer  1        TGCCTGGAACATAATCACCGT  21
Template        3197810  .....................  3197830

Reverse primer  1        GTCGGTAGATAGCCTGTCGC  20
Template        3198011  ....................  3197992
```

>CP027857.1 Pseudomonas aeruginosa strain MPAO1 chromosome, complete genome

```
product length = 202
Forward primer  1        TGCCTGGAACATAATCACCGT  21
Template        3061466  .....................  3061486

Reverse primer  1        GTCGGTAGATAGCCTGTCGC  20
Template        3061667  ....................  3061648
```

>CP053119.1 Pseudomonas aeruginosa strain A17CT chromosome

```
product length = 202
Forward primer  1        TGCCTGGAACATAATCACCGT  21
Template        2454009  .....................  2453989

Reverse primer  1        GTCGGTAGATAGCCTGTCGC  20
Template        2453808  ....................  2453827
```

>CP053118.1 Pseudomonas aeruginosa strain A17PBS chromosome

```
product length = 202
Forward primer  1        TGCCTGGAACATAATCACCGT  21
Template        2453846  .....................  2453826

Reverse primer  1        GTCGGTAGATAGCCTGTCGC  20
Template        2453645  ....................  2453664
```

>CP053116.1 Pseudomonas aeruginosa strain P16PBS chromosome

```
product length = 202
Forward primer  1        TGCCTGGAACATAATCACCGT  21
Template        2453866  .....................  2453846
```

```
Reverse primer  1        GTCGGTAGATAGCCTGTCGC  20
Template        2453665  ....................  2453684
```

>CP053115.1 Pseudomonas aeruginosa strain P4CT chromosome

```
product length = 202
Forward primer  1        TGCCTGGAACATAATCACCGT  21
Template        2453876  .....................  2453856

Reverse primer  1        GTCGGTAGATAGCCTGTCGC  20
Template        2453675  ....................  2453694
```

>CP053114.1 Pseudomonas aeruginosa strain P4PBS chromosome

```
product length = 202
Forward primer  1        TGCCTGGAACATAATCACCGT  21
Template        2453926  .....................  2453906

Reverse primer  1        GTCGGTAGATAGCCTGTCGC  20
Template        2453725  ....................  2453744
```

>CP053113.1 Pseudomonas aeruginosa strain PAO1CT chromosome

```
product length = 202
Forward primer  1        TGCCTGGAACATAATCACCGT  21
Template        2453931  .....................  2453911

Reverse primer  1        GTCGGTAGATAGCCTGTCGC  20
Template        2453730  ....................  2453749
```

>CP053112.1 Pseudomonas aeruginosa strain PAO1PBS chromosome

```
product length = 202
Forward primer  1        TGCCTGGAACATAATCACCGT  21
Template        2453874  .....................  2453854

Reverse primer  1        GTCGGTAGATAGCCTGTCGC  20
Template        2453673  ....................  2453692
```

>CP053111.1 Pseudomonas aeruginosa strain UAB2CT chromosome

```
product length = 202
Forward primer  1        TGCCTGGAACATAATCACCGT  21
Template        2453836  .....................  2453816

Reverse primer  1        GTCGGTAGATAGCCTGTCGC  20
Template        2453635  ....................  2453654
```

>CP053110.1 Pseudomonas aeruginosa strain UAB2PBS chromosome

```
product length = 202
Forward primer  1        TGCCTGGAACATAATCACCGT  21
Template        2453871  .....................  2453851
```

```
Reverse primer  1        GTCGGTAGATAGCCTGTCGC  20
Template        2453670  ....................  2453689
```

>CP056774.1 Pseudomonas aeruginosa strain CDN129 chromosome, complete genome

```
product length = 202
Forward primer  1        TGCCTGGAACATAATCACCGT  21
Template        2370667  .....................  2370647

Reverse primer  1        GTCGGTAGATAGCCTGTCGC  20
Template        2370466  ....................  2370485
```

>CP034908.2 Pseudomonas aeruginosa strain PA0750 chromosome, complete genome

```
product length = 202
Forward primer  1        TGCCTGGAACATAATCACCGT  21
Template        2444998  .....................  2444978

Reverse primer  1        GTCGGTAGATAGCCTGTCGC  20
Template        2444797  ....................  2444816
```

>CP054591.1 Pseudomonas aeruginosa strain CDN118 chromosome, complete genome

```
product length = 202
Forward primer  1        TGCCTGGAACATAATCACCGT  21
Template        3221377  .....................  3221357

Reverse primer  1        GTCGGTAGATAGCCTGTCGC  20
Template        3221176  ....................  3221195
```

>CP050052.1 Pseudomonas aeruginosa strain LIUYANG-E chromosome, complete genome

```
product length = 202
Forward primer  1        TGCCTGGAACATAATCACCGT  21
Template        3061121  .....................  3061141

Reverse primer  1        GTCGGTAGATAGCCTGTCGC  20
Template        3061322  ....................  3061303
```

>CP050054.1 Pseudomonas aeruginosa strain LIUYANG-A chromosome, complete genome

```
product length = 202
Forward primer  1        TGCCTGGAACATAATCACCGT  21
Template        3061103  .....................  3061123

Reverse primer  1        GTCGGTAGATAGCCTGTCGC  20
Template        3061304  ....................  3061285
```

>CP050053.1 Pseudomonas aeruginosa strain LIUYANG-C chromosome, complete genome

```
product length = 202
Forward primer  1        TGCCTGGAACATAATCACCGT  21
```

```
Template        2939991  .....................  2939971

Reverse primer  1        GTCGGTAGATAGCCTGTCGC  20
Template        2939790  ....................  2939809
```

>CP053028.1 Pseudomonas aeruginosa PAO1 chromosome, complete genome

```
product length = 202
Forward primer  1        TGCCTGGAACATAATCACCGT  21
Template        3061949  .....................  3061969

Reverse primer  1        GTCGGTAGATAGCCTGTCGC  20
Template        3062150  ....................  3062131
```

>CP052759.1 Pseudomonas aeruginosa strain LYT4 chromosome, complete genome

```
product length = 202
Forward primer  1        TGCCTGGAACATAATCACCGT  21
Template        3208814  .....................  3208834

Reverse primer  1        GTCGGTAGATAGCCTGTCGC  20
Template        3209015  ....................  3208996
```

>CP047063.1 Pseudomonas aeruginosa strain delta6_4 chromosome

```
product length = 202
Forward primer  1        TGCCTGGAACATAATCACCGT  21
Template        2453882  .....................  2453862

Reverse primer  1        GTCGGTAGATAGCCTGTCGC  20
Template        2453681  ....................  2453700
```

>CP047061.1 Pseudomonas aeruginosa strain delta6_2 chromosome

```
product length = 202
Forward primer  1        TGCCTGGAACATAATCACCGT  21
Template        2453885  .....................  2453865

Reverse primer  1        GTCGGTAGATAGCCTGTCGC  20
Template        2453684  ....................  2453703
```

>CP047062.1 Pseudomonas aeruginosa strain delta6_3 chromosome

```
product length = 202
Forward primer  1        TGCCTGGAACATAATCACCGT  21
Template        2453888  .....................  2453868

Reverse primer  1        GTCGGTAGATAGCCTGTCGC  20
Template        2453687  ....................  2453706
```

>CP047067.1 Pseudomonas aeruginosa strain Cas9_1 chromosome

```
product length = 202
```

```
Forward primer  1        TGCCTGGAACATAATCACCGT  21
Template        2453889  .....................  2453869

Reverse primer  1        GTCGGTAGATAGCCTGTCGC  20
Template        2453688  ....................  2453707
```

>CP047068.1 Pseudomonas aeruginosa strain Cas9_2 chromosome

```
product length = 202
Forward primer  1        TGCCTGGAACATAATCACCGT  21
Template        2453890  .....................  2453870

Reverse primer  1        GTCGGTAGATAGCCTGTCGC  20
Template        2453689  ....................  2453708
```

>CP047065.1 Pseudomonas aeruginosa strain delta6_6 chromosome

```
product length = 202
Forward primer  1        TGCCTGGAACATAATCACCGT  21
Template        2453886  .....................  2453866

Reverse primer  1        GTCGGTAGATAGCCTGTCGC  20
Template        2453685  ....................  2453704
```

>CP041008.1 Pseudomonas aeruginosa strain FDAARGOS_767 chromosome, complete genome

```
product length = 202
Forward primer  1        TGCCTGGAACATAATCACCGT  21
Template        2673805  .....................  2673785

Reverse primer  1        GTCGGTAGATAGCCTGTCGC  20
Template        2673604  ....................  2673623
```

>CP034429.1 Pseudomonas aeruginosa strain GIMC5015:PAKB6, complete sequence

```
product length = 202
Forward primer  1        TGCCTGGAACATAATCACCGT  21
Template        3049607  .....................  3049627

Reverse primer  1        GTCGGTAGATAGCCTGTCGC  20
Template        3049808  ....................  3049789
```

>CP032541.1 Pseudomonas aeruginosa strain PGN5 chromosome

```
product length = 202
Forward primer  1        TGCCTGGAACATAATCACCGT  21
Template        2453891  .....................  2453871

Reverse primer  1        GTCGGTAGATAGCCTGTCGC  20
Template        2453690  ....................  2453709
```

>CP032540.1 Pseudomonas aeruginosa strain PGN4 chromosome

```
product length = 202
Forward primer  1        TGCCTGGAACATAATCACCGT  21
Template        2453890  .....................  2453870

Reverse primer  1        GTCGGTAGATAGCCTGTCGC  20
Template        2453689  ....................  2453708
```

>CP029713.1 Pseudomonas aeruginosa strain BH9 chromosome

```
product length = 202
Forward primer  1        TGCCTGGAACATAATCACCGT  21
Template        3518590  .....................  3518610

Reverse primer  1        GTCGGTAGATAGCCTGTCGC  20
Template        3518791  ....................  3518772
```

>CP032126.1 Pseudomonas aeruginosa strain PAO1161 chromosome, complete genome

```
product length = 202
Forward primer  1        TGCCTGGAACATAATCACCGT  21
Template        3061833  .....................  3061853

Reverse primer  1        GTCGGTAGATAGCCTGTCGC  20
Template        3062034  ....................  3062015
```

>CP029707.1 Pseudomonas aeruginosa strain K34-7 chromosome, complete genome

```
product length = 202
Forward primer  1        TGCCTGGAACATAATCACCGT  21
Template        1917553  .....................  1917573

Reverse primer  1        GTCGGTAGATAGCCTGTCGC  20
Template        1917754  ....................  1917735
```

>CP029089.1 Pseudomonas aeruginosa strain AR444 chromosome, complete genome

```
product length = 202
Forward primer  1        TGCCTGGAACATAATCACCGT  21
Template        2030898  .....................  2030878

Reverse primer  1        GTCGGTAGATAGCCTGTCGC  20
Template        2030697  ....................  2030716
```

>CP027174.1 Pseudomonas aeruginosa strain AR_0230 chromosome, complete genome

```
product length = 202
Forward primer  1        TGCCTGGAACATAATCACCGT  21
Template        1955244  .....................  1955264

Reverse primer  1        GTCGGTAGATAGCCTGTCGC  20
Template        1955445  ....................  1955426
```

>CP027165.1 Pseudomonas aeruginosa strain AR_0360 chromosome, complete genome

```
product length = 202
Forward primer  1        TGCCTGGAACATAATCACCGT  21
Template        1913516  .....................  1913536

Reverse primer  1        GTCGGTAGATAGCCTGTCGC  20
Template        1913717  ....................  1913698
```

>CP017293.1 Pseudomonas aeruginosa strain PA83, complete genome

```
product length = 202
Forward primer  1        TGCCTGGAACATAATCACCGT  21
Template        3350760  .....................  3350780

Reverse primer  1        GTCGGTAGATAGCCTGTCGC  20
Template        3350961  ....................  3350942
```

>CP017149.1 Pseudomonas aeruginosa strain ATCC 15692, complete genome

```
product length = 202
Forward primer  1        TGCCTGGAACATAATCACCGT  21
Template        3061468  .....................  3061488

Reverse primer  1        GTCGGTAGATAGCCTGTCGC  20
Template        3061669  ....................  3061650
```

>CP014948.1 Pseudomonas aeruginosa strain N17-1, complete genome

```
product length = 202
Forward primer  1        TGCCTGGAACATAATCACCGT  21
Template        3090199  .....................  3090219

Reverse primer  1        GTCGGTAGATAGCCTGTCGC  20
Template        3090400  ....................  3090381
```

>OX638701.1 Pseudomonas aeruginosa strain 4782MK genome assembly, chromosome: 4782

```
product length = 202
Forward primer  1        TGCCTGGAACATAATCACCGT  21
Template        4793532  .....................  4793552

Reverse primer  1        GTCGGTAGATAGCCTGTCGC  20
Template        4793733  ....................  4793714
```

>LN871187.1 Pseudomonas aeruginosa genome assembly PAO1OR, chromosome : I

```
product length = 202
Forward primer  1        TGCCTGGAACATAATCACCGT  21
Template        3061958  .....................  3061978

Reverse primer  1        GTCGGTAGATAGCCTGTCGC  20
Template        3062159  ....................  3062140
```

>CP007147.1 Pseudomonas aeruginosa YL84, complete genome

```
product length = 202
Forward primer  1        TGCCTGGAACATAATCACCGT  21
Template        4164636  .....................  4164616

Reverse primer  1        GTCGGTAGATAGCCTGTCGC  20
Template        4164435  ....................  4164454
```

>CP006832.1 Pseudomonas aeruginosa PAO1-VE13 genome

```
product length = 202
Forward primer  1        TGCCTGGAACATAATCACCGT  21
Template        2453874  .....................  2453854

Reverse primer  1        GTCGGTAGATAGCCTGTCGC  20
Template        2453673  ....................  2453692
```

>CP006831.1 Pseudomonas aeruginosa PAO1-VE2 genome

```
product length = 202
Forward primer  1        TGCCTGGAACATAATCACCGT  21
Template        2453874  .....................  2453854

Reverse primer  1        GTCGGTAGATAGCCTGTCGC  20
Template        2453673  ....................  2453692
```

>CP006705.1 Pseudomonas aeruginosa PAO581 genome

```
product length = 202
Forward primer  1        TGCCTGGAACATAATCACCGT  21
Template        2453764  .....................  2453744

Reverse primer  1        GTCGGTAGATAGCCTGTCGC  20
Template        2453563  ....................  2453582
```

>AE004091.2 Pseudomonas aeruginosa PAO1, complete genome

```
product length = 202
Forward primer  1        TGCCTGGAACATAATCACCGT  21
Template        2453879  .....................  2453859

Reverse primer  1        GTCGGTAGATAGCCTGTCGC  20
Template        2453678  ....................  2453697
```

>CP121766.1 Pseudomonas aeruginosa strain 22112 chromosome, complete genome

```
product length = 202
Forward primer  1        TGCCTGGAACATAATCACCGT  21
Template        2711583  ............G........  2711603

Reverse primer  1        GTCGGTAGATAGCCTGTCGC  20
Template        2711784  ....................  2711765
```

>CP127016.1 Pseudomonas aeruginosa strain TBCF10839 chromosome

```
product length = 202
Forward primer  1        TGCCTGGAACATAATCACCGT  21
Template        922429   ............G........  922409

Reverse primer  1        GTCGGTAGATAGCCTGTCGC  20
Template        922228   ....................  922247
```

>CP123792.1 Pseudomonas aeruginosa strain 2021CK-01658 chromosome, complete genome

```
product length = 202
Forward primer  1        TGCCTGGAACATAATCACCGT  21
Template        3749538  ............G........  3749518

Reverse primer  1        GTCGGTAGATAGCCTGTCGC  20
Template        3749337  ....................  3749356
```

>CP109757.1 Pseudomonas aeruginosa strain 2017-45-85 chromosome, complete genome

```
product length = 202
Forward primer  1        TGCCTGGAACATAATCACCGT  21
Template        3270283  ............G........  3270303

Reverse primer  1        GTCGGTAGATAGCCTGTCGC  20
Template        3270484  ....................  3270465
```

>CP109683.1 Pseudomonas aeruginosa strain 2017-45-169 chromosome, complete genome

```
product length = 202
Forward primer  1        TGCCTGGAACATAATCACCGT  21
Template        3838199  ............G........  3838219

Reverse primer  1        GTCGGTAGATAGCCTGTCGC  20
Template        3838400  ....................  3838381
```

>CP109685.1 Pseudomonas aeruginosa strain 2017-45-137A chromosome, complete genome

```
product length = 202
Forward primer  1        TGCCTGGAACATAATCACCGT  21
Template        1479244  ............G........  1479264

Reverse primer  1        GTCGGTAGATAGCCTGTCGC  20
Template        1479445  ....................  1479426
```

>CP061073.2 Pseudomonas aeruginosa strain PAD8 chromosome, complete genome

```
product length = 202
Forward primer  1        TGCCTGGAACATAATCACCGT  21
Template        6361263  ............G........  6361283

Reverse primer  1        GTCGGTAGATAGCCTGTCGC  20
Template        6361464  ....................  6361445
```

>CP123786.1 Pseudomonas aeruginosa strain 2021CK-01424 chromosome, complete genome

```
product length = 202
Forward primer  1        TGCCTGGAACATAATCACCGT  21
Template        3478443  ............G........  3478463

Reverse primer  1        GTCGGTAGATAGCCTGTCGC  20
Template        3478644  ....................  3478625
```

>CP123785.1 Pseudomonas aeruginosa strain 2021CK-01267 chromosome, complete genome

```
product length = 202
Forward primer  1        TGCCTGGAACATAATCACCGT  21
Template        3478812  ............G........  3478832

Reverse primer  1        GTCGGTAGATAGCCTGTCGC  20
Template        3479013  ....................  3478994
```

>CP123787.1 Pseudomonas aeruginosa strain 2020CK-00194 chromosome, complete genome

```
product length = 202
Forward primer  1        TGCCTGGAACATAATCACCGT  21
Template        3395927  ............G........  3395947

Reverse primer  1        GTCGGTAGATAGCCTGTCGC  20
Template        3396128  ....................  3396109
```

>CP123789.1 Pseudomonas aeruginosa strain 2021CK-01381 chromosome, complete genome

```
product length = 202
Forward primer  1        TGCCTGGAACATAATCACCGT  21
Template        3741212  ............G........  3741192

Reverse primer  1        GTCGGTAGATAGCCTGTCGC  20
Template        3741011  ....................  3741030
```

>CP123791.1 Pseudomonas aeruginosa strain 2021CK-01305 chromosome, complete genome

```
product length = 202
Forward primer  1        TGCCTGGAACATAATCACCGT  21
Template        3458665  ............G........  3458685

Reverse primer  1        GTCGGTAGATAGCCTGTCGC  20
Template        3458866  ....................  3458847
```

>CP123793.1 Pseudomonas aeruginosa strain 2021CK-01107 chromosome, complete genome

```
product length = 202
Forward primer  1        TGCCTGGAACATAATCACCGT  21
Template        3367027  ............G........  3367047

Reverse primer  1        GTCGGTAGATAGCCTGTCGC  20
Template        3367228  ....................  3367209
```

>CP096964.1 Pseudomonas aeruginosa strain NY13936 chromosome, complete genome

```
product length = 202
Forward primer  1        TGCCTGGAACATAATCACCGT  21
Template        3472423  ............G........  3472403

Reverse primer  1        GTCGGTAGATAGCCTGTCGC  20
Template        3472222  ....................  3472241
```

>CP096961.1 Pseudomonas aeruginosa strain NY13932 chromosome, complete genome

```
product length = 202
Forward primer  1        TGCCTGGAACATAATCACCGT  21
Template        3098059  ............G........  3098079

Reverse primer  1        GTCGGTAGATAGCCTGTCGC  20
Template        3098260  ....................  3098241
```

>CP096960.1 Pseudomonas aeruginosa strain NY11254 chromosome, complete genome

```
product length = 202
Forward primer  1        TGCCTGGAACATAATCACCGT  21
Template        3700464  ............G........  3700444

Reverse primer  1        GTCGGTAGATAGCCTGTCGC  20
Template        3700263  ....................  3700282
```

>CP096958.1 Pseudomonas aeruginosa strain NY11210 chromosome, complete genome

```
product length = 202
Forward primer  1        TGCCTGGAACATAATCACCGT  21
Template        3440854  ............G........  3440874

Reverse primer  1        GTCGGTAGATAGCCTGTCGC  20
Template        3441055  ....................  3441036
```

>CP096956.1 Pseudomonas aeruginosa strain NY11173 chromosome, complete genome

```
product length = 202
Forward primer  1        TGCCTGGAACATAATCACCGT  21
Template        3219054  ............G........  3219074

Reverse primer  1        GTCGGTAGATAGCCTGTCGC  20
Template        3219255  ....................  3219236
```

>CP096953.1 Pseudomonas aeruginosa strain NY5535 chromosome, complete genome

```
product length = 202
Forward primer  1        TGCCTGGAACATAATCACCGT  21
Template        3248845  ............G........  3248865

Reverse primer  1        GTCGGTAGATAGCCTGTCGC  20
Template        3249046  ....................  3249027
```

>CP096950.1 Pseudomonas aeruginosa strain NY5532 chromosome, complete genome

```
product length = 202
Forward primer  1        TGCCTGGAACATAATCACCGT  21
Template        3258332  ............G........  3258352

Reverse primer  1        GTCGGTAGATAGCCTGTCGC  20
Template        3258533  ....................  3258514
```

>CP096946.1 Pseudomonas aeruginosa strain NY5530 chromosome, complete genome

```
product length = 202
Forward primer  1        TGCCTGGAACATAATCACCGT  21
Template        3439223  ............G........  3439243

Reverse primer  1        GTCGGTAGATAGCCTGTCGC  20
Template        3439424  ....................  3439405
```

>CP096942.1 Pseudomonas aeruginosa strain NY5524 chromosome, complete genome

```
product length = 202
Forward primer  1        TGCCTGGAACATAATCACCGT  21
Template        3204779  ............G........  3204799

Reverse primer  1        GTCGGTAGATAGCCTGTCGC  20
Template        3204980  ....................  3204961
```

>CP096941.1 Pseudomonas aeruginosa strain NY5523 chromosome, complete genome

```
product length = 202
Forward primer  1        TGCCTGGAACATAATCACCGT  21
Template        3700494  ............G........  3700474

Reverse primer  1        GTCGGTAGATAGCCTGTCGC  20
Template        3700293  ....................  3700312
```

>CP096937.1 Pseudomonas aeruginosa strain NY5520 chromosome, complete genome

```
product length = 202
Forward primer  1        TGCCTGGAACATAATCACCGT  21
Template        3249145  ............G........  3249165

Reverse primer  1        GTCGGTAGATAGCCTGTCGC  20
Template        3249346  ....................  3249327
```

>CP096934.1 Pseudomonas aeruginosa strain NY5511 chromosome, complete genome

```
product length = 202
Forward primer  1        TGCCTGGAACATAATCACCGT  21
Template        4212066  ............G........  4212046

Reverse primer  1        GTCGGTAGATAGCCTGTCGC  20
Template        4211865  ....................  4211884
```

>CP096932.1 Pseudomonas aeruginosa strain NY5510 chromosome, complete genome

```
product length = 202
Forward primer  1        TGCCTGGAACATAATCACCGT  21
Template        3253473  ............G........  3253493

Reverse primer  1        GTCGGTAGATAGCCTGTCGC  20
Template        3253674  ....................  3253655
```

>CP096929.1 Pseudomonas aeruginosa strain NY5507 chromosome, complete genome

```
product length = 202
Forward primer  1        TGCCTGGAACATAATCACCGT  21
Template        3437861  ............G........  3437881

Reverse primer  1        GTCGGTAGATAGCCTGTCGC  20
Template        3438062  ....................  3438043
```

>CP096927.1 Pseudomonas aeruginosa strain NY5506 chromosome, complete genome

```
product length = 202
Forward primer  1        TGCCTGGAACATAATCACCGT  21
Template        3078912  ............G........  3078932

Reverse primer  1        GTCGGTAGATAGCCTGTCGC  20
Template        3079113  ....................  3079094
```

>CP124673.1 Pseudomonas aeruginosa strain 2022CK-00491 chromosome, complete genome

```
product length = 202
Forward primer  1        TGCCTGGAACATAATCACCGT  21
Template        3355257  ............G........  3355277

Reverse primer  1        GTCGGTAGATAGCCTGTCGC  20
Template        3355458  ....................  3355439
```

>CP124674.1 Pseudomonas aeruginosa strain 2022CK-00339 chromosome, complete genome

```
product length = 202
Forward primer  1        TGCCTGGAACATAATCACCGT  21
Template        3824889  ............G........  3824869

Reverse primer  1        GTCGGTAGATAGCCTGTCGC  20
Template        3824688  ....................  3824707
```

>CP084890.1 Pseudomonas aeruginosa strain CH1 chromosome

```
product length = 202
Forward primer  1        TGCCTGGAACATAATCACCGT  21
Template        6949879  ............G........  6949859

Reverse primer  1        GTCGGTAGATAGCCTGTCGC  20
Template        6949678  ....................  6949697
```

>CP124658.1 Pseudomonas aeruginosa strain 2022CK-00068 chromosome, complete genome

```
product length = 202
Forward primer  1        TGCCTGGAACATAATCACCGT  21
Template        3282982  ............G........  3283002

Reverse primer  1        GTCGGTAGATAGCCTGTCGC  20
Template        3283183  ....................  3283164
```

>CP124662.1 Pseudomonas aeruginosa strain 2021CK-01633 chromosome, complete genome

```
product length = 202
Forward primer  1        TGCCTGGAACATAATCACCGT  21
Template        3395928  ............G........  3395948

Reverse primer  1        GTCGGTAGATAGCCTGTCGC  20
Template        3396129  ....................  3396110
```

>CP124652.1 Pseudomonas aeruginosa strain 2020CK-00443 chromosome, complete genome

```
product length = 202
Forward primer  1        TGCCTGGAACATAATCACCGT  21
Template        3559382  ............G........  3559362

Reverse primer  1        GTCGGTAGATAGCCTGTCGC  20
Template        3559181  ....................  3559200
```

>CP124649.1 Pseudomonas aeruginosa strain 2020CK-00218 chromosome, complete genome

```
product length = 202
Forward primer  1        TGCCTGGAACATAATCACCGT  21
Template        3474979  ............G........  3474999

Reverse primer  1        GTCGGTAGATAGCCTGTCGC  20
Template        3475180  ....................  3475161
```

>CP124664.1 Pseudomonas aeruginosa strain 2021CK-01256 chromosome, complete genome

```
product length = 202
Forward primer  1        TGCCTGGAACATAATCACCGT  21
Template        3169284  ............G........  3169304

Reverse primer  1        GTCGGTAGATAGCCTGTCGC  20
Template        3169485  ....................  3169466
```

>CP124655.1 Pseudomonas aeruginosa strain 2022CK-00096 chromosome, complete genome

```
product length = 202
Forward primer  1        TGCCTGGAACATAATCACCGT  21
Template        3625291  ............G........  3625271

Reverse primer  1        GTCGGTAGATAGCCTGTCGC  20
Template        3625090  ....................  3625109
```

>CP124654.1 Pseudomonas aeruginosa strain 2021CK-01851 chromosome, complete genome

```
product length = 202
Forward primer  1        TGCCTGGAACATAATCACCGT  21
Template        3170539  ............G........  3170559

Reverse primer  1        GTCGGTAGATAGCCTGTCGC  20
Template        3170740  ....................  3170721
```

>CP124657.1 Pseudomonas aeruginosa strain 2022CK-00069 chromosome, complete genome

```
product length = 202
Forward primer  1        TGCCTGGAACATAATCACCGT  21
Template        3290897  ............G........  3290917

Reverse primer  1        GTCGGTAGATAGCCTGTCGC  20
Template        3291098  ....................  3291079
```

>CP124651.1 Pseudomonas aeruginosa strain 2020CK-00217 chromosome, complete genome

```
product length = 202
Forward primer  1        TGCCTGGAACATAATCACCGT  21
Template        3396024  ............G........  3396044

Reverse primer  1        GTCGGTAGATAGCCTGTCGC  20
Template        3396225  ....................  3396206
```

>CP124669.1 Pseudomonas aeruginosa strain 2021CK-01494 chromosome, complete genome

```
product length = 202
Forward primer  1        TGCCTGGAACATAATCACCGT  21
Template        3196133  ............G........  3196153

Reverse primer  1        GTCGGTAGATAGCCTGTCGC  20
Template        3196334  ....................  3196315
```

>CP124638.1 Pseudomonas aeruginosa strain 2021CK-01158 chromosome, complete genome

```
product length = 202
Forward primer  1        TGCCTGGAACATAATCACCGT  21
Template        3154323  ............G........  3154303

Reverse primer  1        GTCGGTAGATAGCCTGTCGC  20
Template        3154122  ....................  3154141
```

>CP124668.1 Pseudomonas aeruginosa strain 2021CK-01445 chromosome, complete genome

```
product length = 202
Forward primer  1        TGCCTGGAACATAATCACCGT  21
Template        3196093  ............G........  3196113

Reverse primer  1        GTCGGTAGATAGCCTGTCGC  20
Template        3196294  ....................  3196275
```

>CP124666.1 Pseudomonas aeruginosa strain 2021CK-01283 chromosome, complete genome

```
product length = 202
Forward primer  1        TGCCTGGAACATAATCACCGT  21
Template        3155526  ............G........  3155546

Reverse primer  1        GTCGGTAGATAGCCTGTCGC  20
Template        3155727  ....................  3155708
```

>CP124624.1 Pseudomonas aeruginosa strain 2021CK-01157 chromosome, complete genome

```
product length = 202
Forward primer  1        TGCCTGGAACATAATCACCGT  21
Template        3137226  ............G........  3137246

Reverse primer  1        GTCGGTAGATAGCCTGTCGC  20
Template        3137427  ....................  3137408
```

>CP124665.1 Pseudomonas aeruginosa strain 2021CK-01229 chromosome, complete genome

```
product length = 202
Forward primer  1        TGCCTGGAACATAATCACCGT  21
Template        3155538  ............G........  3155558

Reverse primer  1        GTCGGTAGATAGCCTGTCGC  20
Template        3155739  ....................  3155720
```

>CP124667.1 Pseudomonas aeruginosa strain 2021CK-01315 chromosome, complete genome

```
product length = 202
Forward primer  1        TGCCTGGAACATAATCACCGT  21
Template        3337306  ............G........  3337286

Reverse primer  1        GTCGGTAGATAGCCTGTCGC  20
Template        3337105  ....................  3337124
```

>CP124641.1 Pseudomonas aeruginosa strain 2021CK-01198 chromosome, complete genome

```
product length = 202
Forward primer  1        TGCCTGGAACATAATCACCGT  21
Template        3136977  ............G........  3136997

Reverse primer  1        GTCGGTAGATAGCCTGTCGC  20
Template        3137178  ....................  3137159
```

>CP124670.1 Pseudomonas aeruginosa strain 2021CK-01536 chromosome, complete genome

```
product length = 202
Forward primer  1        TGCCTGGAACATAATCACCGT  21
Template        3066914  ............G........  3066934

Reverse primer  1        GTCGGTAGATAGCCTGTCGC  20
Template        3067115  ....................  3067096
```

>CP124646.1 Pseudomonas aeruginosa strain 2020CK-00185 chromosome, complete genome

```
product length = 202
Forward primer  1        TGCCTGGAACATAATCACCGT  21
Template        3643124  ............G........  3643104

Reverse primer  1        GTCGGTAGATAGCCTGTCGC  20
Template        3642923  ....................  3642942
```

>CP124648.1 Pseudomonas aeruginosa strain 2020CK-00220 chromosome, complete genome

```
product length = 202
Forward primer  1        TGCCTGGAACATAATCACCGT  21
Template        5620093  ............G........  5620113

Reverse primer  1        GTCGGTAGATAGCCTGTCGC  20
Template        5620294  ....................  5620275
```

>CP124643.1 Pseudomonas aeruginosa strain 2021CK-01197 chromosome, complete genome

```
product length = 202
Forward primer  1        TGCCTGGAACATAATCACCGT  21
Template        1254348  ............G........  1254328

Reverse primer  1        GTCGGTAGATAGCCTGTCGC  20
Template        1254147  ....................  1254166
```

>CP124626.1 Pseudomonas aeruginosa strain 2021CK-01161 chromosome, complete genome

```
product length = 202
Forward primer  1        TGCCTGGAACATAATCACCGT  21
Template        4800093  ............G........  4800073

Reverse primer  1        GTCGGTAGATAGCCTGTCGC  20
Template        4799892  ....................  4799911
```

>CP124632.1 Pseudomonas aeruginosa strain 2021CK-01162 chromosome, complete genome

```
product length = 202
Forward primer  1        TGCCTGGAACATAATCACCGT  21
Template        3742144  ............G........  3742164

Reverse primer  1        GTCGGTAGATAGCCTGTCGC  20
Template        3742345  ....................  3742326
```

>CP124663.1 Pseudomonas aeruginosa strain 2021CK-01227 chromosome, complete genome

```
product length = 202
Forward primer  1        TGCCTGGAACATAATCACCGT  21
Template        3155683  ............G........  3155703

Reverse primer  1        GTCGGTAGATAGCCTGTCGC  20
Template        3155884  ....................  3155865
```

>CP124622.1 Pseudomonas aeruginosa strain 2021CK-01159 chromosome, complete genome

```
product length = 202
Forward primer  1        TGCCTGGAACATAATCACCGT  21
Template        3337266  ............G........  3337246

Reverse primer  1        GTCGGTAGATAGCCTGTCGC  20
Template        3337065  ....................  3337084
```

>CP124600.1 Pseudomonas aeruginosa strain Li010 chromosome, complete genome

```
product length = 202
Forward primer  1        TGCCTGGAACATAATCACCGT  21
Template        3206794  ............G........  3206774

Reverse primer  1        GTCGGTAGATAGCCTGTCGC  20
Template        3206593  ....................  3206612
```

>CP123953.1 Pseudomonas aeruginosa strain 59 chromosome, complete genome

```
product length = 202
Forward primer  1        TGCCTGGAACATAATCACCGT  21
Template        4358191  ............G........  4358171

Reverse primer  1        GTCGGTAGATAGCCTGTCGC  20
Template        4357990  ....................  4358009
```

>CP116682.1 Pseudomonas aeruginosa strain HS337 chromosome, complete genome

```
product length = 202
Forward primer  1        TGCCTGGAACATAATCACCGT  21
Template        3222165  ............G........  3222185

Reverse primer  1        GTCGGTAGATAGCCTGTCGC  20
Template        3222366  ....................  3222347
```

>CP110190.1 Pseudomonas aeruginosa strain HS204 chromosome, complete genome

```
product length = 202
Forward primer  1        TGCCTGGAACATAATCACCGT  21
Template        3324036  ............G........  3324016

Reverse primer  1        GTCGGTAGATAGCCTGTCGC  20
Template        3323835  ....................  3323854
```

>CP118638.1 Pseudomonas aeruginosa strain P9 chromosome, complete genome

```
product length = 202
Forward primer  1        TGCCTGGAACATAATCACCGT  21
Template        3183933  ............G........  3183953

Reverse primer  1        GTCGGTAGATAGCCTGTCGC  20
Template        3184134  ....................  3184115
```

>CP118641.1 Pseudomonas aeruginosa strain P23 chromosome, complete genome

```
product length = 202
Forward primer  1        TGCCTGGAACATAATCACCGT  21
Template        3348568  ............G........  3348588

Reverse primer  1        GTCGGTAGATAGCCTGTCGC  20
Template        3348769  ....................  3348750
```

>CP119298.1 Pseudomonas aeruginosa strain SNDPR-01 chromosome, complete genome

```
product length = 202
Forward primer  1        TGCCTGGAACATAATCACCGT  21
Template        3151545  ............G........  3151565

Reverse primer  1        GTCGGTAGATAGCCTGTCGC  20
Template        3151746  ....................  3151727
```

>CP117300.1 Pseudomonas aeruginosa strain 0201761-1 chromosome, complete genome

```
product length = 202
Forward primer  1        TGCCTGGAACATAATCACCGT  21
Template        3344257  ............G........  3344237

Reverse primer  1        GTCGGTAGATAGCCTGTCGC  20
Template        3344056  ....................  3344075
```

>CP084321.1 Pseudomonas aeruginosa strain HS18-89 chromosome, complete genome

```
product length = 202
Forward primer  1        TGCCTGGAACATAATCACCGT  21
Template        3619929  ............G........  3619949

Reverse primer  1        GTCGGTAGATAGCCTGTCGC  20
Template        3620130  ....................  3620111
```

>CP117974.1 Pseudomonas aeruginosa strain B-3509 chromosome, complete genome

```
product length = 202
Forward primer  1        TGCCTGGAACATAATCACCGT  21
Template        2110088  ............G........  2110068

Reverse primer  1        GTCGGTAGATAGCCTGTCGC  20
Template        2109887  ....................  2109906
```

>CP117749.1 Pseudomonas aeruginosa strain 2022CK-00828 chromosome, complete genome

```
product length = 202
Forward primer  1        TGCCTGGAACATAATCACCGT  21
Template        3268638  ............G........  3268658

Reverse primer  1        GTCGGTAGATAGCCTGTCGC  20
Template        3268839  ....................  3268820
```

>CP117527.1 Pseudomonas aeruginosa strain MF1 chromosome, complete genome

```
product length = 202
Forward primer  1        TGCCTGGAACATAATCACCGT  21
Template        3326824  ............G........  3326844

Reverse primer  1        GTCGGTAGATAGCCTGTCGC  20
Template        3327025  ....................  3327006
```

>CP075851.1 Pseudomonas aeruginosa strain PaLo33 chromosome, complete genome

```
product length = 202
Forward primer  1        TGCCTGGAACATAATCACCGT  21
Template        3324097  ............G........  3324117

Reverse primer  1        GTCGGTAGATAGCCTGTCGC  20
Template        3324298  ....................  3324279
```

>CP075849.1 Pseudomonas aeruginosa strain PaLo1 chromosome, complete genome

```
product length = 202
Forward primer  1        TGCCTGGAACATAATCACCGT  21
Template        3436102  ............G........  3436082

Reverse primer  1        GTCGGTAGATAGCCTGTCGC  20
Template        3435901  ....................  3435920
```

>CP075848.1 Pseudomonas aeruginosa strain PaLo2 chromosome, complete genome

```
product length = 202
Forward primer  1        TGCCTGGAACATAATCACCGT  21
Template        3071836  ............G........  3071856

Reverse primer  1        GTCGGTAGATAGCCTGTCGC  20
Template        3072037  ....................  3072018
```

>CP075847.1 Pseudomonas aeruginosa strain PaLo3 chromosome, complete genome

```
product length = 202
Forward primer  1        TGCCTGGAACATAATCACCGT  21
Template        3880816  ............G........  3880796

Reverse primer  1        GTCGGTAGATAGCCTGTCGC  20
Template        3880615  ....................  3880634
```

>CP075846.1 Pseudomonas aeruginosa strain PaLo4 chromosome

```
product length = 202
Forward primer  1        TGCCTGGAACATAATCACCGT  21
Template        3782325  ............G........  3782305

Reverse primer  1        GTCGGTAGATAGCCTGTCGC  20
Template        3782124  ....................  3782143
```

>CP075844.1 Pseudomonas aeruginosa strain PaLo6 chromosome, complete genome

```
product length = 202
Forward primer  1        TGCCTGGAACATAATCACCGT  21
Template        3257380  ............G........  3257400

Reverse primer  1        GTCGGTAGATAGCCTGTCGC  20
Template        3257581  ....................  3257562
```

>CP075843.1 Pseudomonas aeruginosa strain PaLo7 chromosome

```
product length = 202
Forward primer  1        TGCCTGGAACATAATCACCGT  21
Template        3278414  ............G........  3278434

Reverse primer  1        GTCGGTAGATAGCCTGTCGC  20
Template        3278615  ....................  3278596
```

>CP075840.1 Pseudomonas aeruginosa strain PaLo10 chromosome, complete genome

```
product length = 202
Forward primer  1        TGCCTGGAACATAATCACCGT  21
Template        3046167  ............G........  3046187

Reverse primer  1        GTCGGTAGATAGCCTGTCGC  20
Template        3046368  ....................  3046349
```

>CP075835.1 Pseudomonas aeruginosa strain PaLo14 chromosome, complete genome

```
product length = 202
Forward primer  1        TGCCTGGAACATAATCACCGT  21
Template        3226651  ............G........  3226631

Reverse primer  1        GTCGGTAGATAGCCTGTCGC  20
Template        3226450  ....................  3226469
```

>CP075833.1 Pseudomonas aeruginosa strain PaLo17 chromosome, complete genome

```
product length = 202
Forward primer  1        TGCCTGGAACATAATCACCGT  21
Template        3081168  ............G........  3081188

Reverse primer  1        GTCGGTAGATAGCCTGTCGC  20
Template        3081369  ....................  3081350
```

>CP075832.1 Pseudomonas aeruginosa strain PaLo20 chromosome, complete genome

```
product length = 202
Forward primer  1        TGCCTGGAACATAATCACCGT  21
Template        3701544  ............G........  3701524

Reverse primer  1        GTCGGTAGATAGCCTGTCGC  20
Template        3701343  ....................  3701362
```

>CP075831.1 Pseudomonas aeruginosa strain PaLo21 chromosome, complete genome

```
product length = 202
Forward primer  1        TGCCTGGAACATAATCACCGT  21
Template        3109406  ............G........  3109426

Reverse primer  1        GTCGGTAGATAGCCTGTCGC  20
Template        3109607  ....................  3109588
```

>CP075829.1 Pseudomonas aeruginosa strain PaLo25 chromosome, complete genome

```
product length = 202
Forward primer  1        TGCCTGGAACATAATCACCGT  21
Template        3021664  ............G........  3021684

Reverse primer  1        GTCGGTAGATAGCCTGTCGC  20
Template        3021865  ....................  3021846
```

>CP075828.1 Pseudomonas aeruginosa strain PaLo26 chromosome, complete genome

```
product length = 202
Forward primer  1        TGCCTGGAACATAATCACCGT  21
Template        3134054  ............G........  3134074

Reverse primer  1        GTCGGTAGATAGCCTGTCGC  20
Template        3134255  ....................  3134236
```

>CP075827.1 Pseudomonas aeruginosa strain PaLo27 chromosome, complete genome

```
product length = 202
Forward primer  1        TGCCTGGAACATAATCACCGT  21
Template        3343517  ............G........  3343537

Reverse primer  1        GTCGGTAGATAGCCTGTCGC  20
Template        3343718  ....................  3343699
```

>CP075826.1 Pseudomonas aeruginosa strain PaLo29 chromosome, complete genome

```
product length = 202
Forward primer  1        TGCCTGGAACATAATCACCGT  21
Template        3250760  ............G........  3250780

Reverse primer  1        GTCGGTAGATAGCCTGTCGC  20
Template        3250961  ....................  3250942
```

>CP075825.1 Pseudomonas aeruginosa strain PaLo30 chromosome, complete genome

```
product length = 202
Forward primer  1        TGCCTGGAACATAATCACCGT  21
Template        3124310  ............G........  3124330

Reverse primer  1        GTCGGTAGATAGCCTGTCGC  20
Template        3124511  ....................  3124492
```

>CP075824.1 Pseudomonas aeruginosa strain PaLo31 chromosome, complete genome

```
product length = 202
Forward primer  1        TGCCTGGAACATAATCACCGT  21
Template        3265928  ............G........  3265948

Reverse primer  1        GTCGGTAGATAGCCTGTCGC  20
Template        3266129  ....................  3266110
```

>CP075823.1 Pseudomonas aeruginosa strain PaLo32 chromosome, complete genome

```
product length = 202
Forward primer  1        TGCCTGGAACATAATCACCGT  21
Template        3324054  ............G........  3324074

Reverse primer  1        GTCGGTAGATAGCCTGTCGC  20
Template        3324255  ....................  3324236
```

>CP075822.1 Pseudomonas aeruginosa strain PaLo34 chromosome, complete genome

```
product length = 202
Forward primer  1        TGCCTGGAACATAATCACCGT  21
Template        3124307  ............G........  3124327

Reverse primer  1        GTCGGTAGATAGCCTGTCGC  20
Template        3124508  ....................  3124489
```

>CP075819.1 Pseudomonas aeruginosa strain PaLo37 chromosome, complete genome

```
product length = 202
Forward primer  1        TGCCTGGAACATAATCACCGT  21
Template        2844595  ............G........  2844575

Reverse primer  1        GTCGGTAGATAGCCTGTCGC  20
Template        2844394  ....................  2844413
```

>CP075818.1 Pseudomonas aeruginosa strain PaLo38 chromosome, complete genome

```
product length = 202
Forward primer  1        TGCCTGGAACATAATCACCGT  21
Template        3124290  ............G........  3124310

Reverse primer  1        GTCGGTAGATAGCCTGTCGC  20
Template        3124491  ....................  3124472
```

>CP075813.1 Pseudomonas aeruginosa strain PaLo45 chromosome, complete genome

```
product length = 202
Forward primer  1        TGCCTGGAACATAATCACCGT  21
Template        3121248  ............G........  3121268

Reverse primer  1        GTCGGTAGATAGCCTGTCGC  20
Template        3121449  ....................  3121430
```

>CP075812.1 Pseudomonas aeruginosa strain PaLo46 chromosome, complete genome

```
product length = 202
Forward primer  1        TGCCTGGAACATAATCACCGT  21
Template        3311816  ............G........  3311836

Reverse primer  1        GTCGGTAGATAGCCTGTCGC  20
Template        3312017  ....................  3311998
```

>CP075811.1 Pseudomonas aeruginosa strain PaLo152 chromosome, complete genome

```
product length = 202
Forward primer  1        TGCCTGGAACATAATCACCGT  21
Template        3076332  ............G........  3076352

Reverse primer  1        GTCGGTAGATAGCCTGTCGC  20
Template        3076533  ....................  3076514
```

>CP075810.1 Pseudomonas aeruginosa strain PaLo166 chromosome, complete genome

```
product length = 202
Forward primer  1        TGCCTGGAACATAATCACCGT  21
Template        3191751  ............G........  3191771

Reverse primer  1        GTCGGTAGATAGCCTGTCGC  20
Template        3191952  ....................  3191933
```

>CP075809.1 Pseudomonas aeruginosa strain PaLo170 chromosome, complete genome

```
product length = 202
Forward primer  1        TGCCTGGAACATAATCACCGT  21
Template        3150238  ............G........  3150258

Reverse primer  1        GTCGGTAGATAGCCTGTCGC  20
Template        3150439  ....................  3150420
```

>CP075808.1 Pseudomonas aeruginosa strain PaLo185 chromosome

```
product length = 202
Forward primer  1        TGCCTGGAACATAATCACCGT  21
Template        3486781  ............G........  3486801

Reverse primer  1        GTCGGTAGATAGCCTGTCGC  20
Template        3486982  ....................  3486963
```

>CP075807.1 Pseudomonas aeruginosa strain PaLo191 chromosome, complete genome

```
product length = 202
Forward primer  1        TGCCTGGAACATAATCACCGT  21
Template        3145831  ............G........  3145811

Reverse primer  1        GTCGGTAGATAGCCTGTCGC  20
Template        3145630  ....................  3145649
```

>CP075806.1 Pseudomonas aeruginosa strain PaLo226 chromosome, complete genome

```
product length = 202
Forward primer  1        TGCCTGGAACATAATCACCGT  21
Template        3080513  ............G........  3080533

Reverse primer  1        GTCGGTAGATAGCCTGTCGC  20
Template        3080714  ....................  3080695
```

>CP075805.1 Pseudomonas aeruginosa strain PaLo227 chromosome, complete genome

```
product length = 202
Forward primer  1        TGCCTGGAACATAATCACCGT  21
Template        3080546  ............G........  3080566

Reverse primer  1        GTCGGTAGATAGCCTGTCGC  20
Template        3080747  ....................  3080728
```

>CP075804.1 Pseudomonas aeruginosa strain PaLo228 chromosome, complete genome

```
product length = 202
Forward primer  1        TGCCTGGAACATAATCACCGT  21
Template        3080513  ............G........  3080533

Reverse primer  1        GTCGGTAGATAGCCTGTCGC  20
Template        3080714  ....................  3080695
```

>CP075803.1 Pseudomonas aeruginosa strain PaLo229 chromosome, complete genome

```
product length = 202
Forward primer  1        TGCCTGGAACATAATCACCGT  21
Template        3148271  ............G........  3148291

Reverse primer  1        GTCGGTAGATAGCCTGTCGC  20
Template        3148472  ....................  3148453
```

>CP075802.1 Pseudomonas aeruginosa strain PaLo240 chromosome, complete genome

```
product length = 202
Forward primer  1        TGCCTGGAACATAATCACCGT  21
Template        3513579  ............G........  3513599

Reverse primer  1        GTCGGTAGATAGCCTGTCGC  20
Template        3513780  ....................  3513761
```

>CP075801.1 Pseudomonas aeruginosa strain PaLo249 chromosome, complete genome

```
product length = 202
Forward primer  1        TGCCTGGAACATAATCACCGT  21
Template        3080536  ............G........  3080556

Reverse primer  1        GTCGGTAGATAGCCTGTCGC  20
Template        3080737  ....................  3080718
```

>CP075799.1 Pseudomonas aeruginosa strain PaLo310 chromosome, complete genome

```
product length = 202
Forward primer  1        TGCCTGGAACATAATCACCGT  21
Template        3107689  ............G........  3107709

Reverse primer  1        GTCGGTAGATAGCCTGTCGC  20
Template        3107890  ....................  3107871
```

>CP075798.1 Pseudomonas aeruginosa strain PaLo323 chromosome, complete genome

```
product length = 202
Forward primer  1        TGCCTGGAACATAATCACCGT  21
Template        3155614  ............G........  3155634

Reverse primer  1        GTCGGTAGATAGCCTGTCGC  20
Template        3155815  ....................  3155796
```

>CP075797.1 Pseudomonas aeruginosa strain PaLo326 chromosome, complete genome

```
product length = 202
Forward primer  1        TGCCTGGAACATAATCACCGT  21
Template        3069467  ............G........  3069487

Reverse primer  1        GTCGGTAGATAGCCTGTCGC  20
Template        3069668  ....................  3069649
```

>CP075796.1 Pseudomonas aeruginosa strain PaLo402 chromosome, complete genome

```
product length = 202
Forward primer  1        TGCCTGGAACATAATCACCGT  21
Template        3064302  ............G........  3064322

Reverse primer  1        GTCGGTAGATAGCCTGTCGC  20
Template        3064503  ....................  3064484
```

>CP075794.1 Pseudomonas aeruginosa strain PaLo418 chromosome, complete genome

```
product length = 202
Forward primer  1        TGCCTGGAACATAATCACCGT  21
Template        3026745  ............G........  3026765

Reverse primer  1        GTCGGTAGATAGCCTGTCGC  20
Template        3026946  ....................  3026927
```

>CP075793.1 Pseudomonas aeruginosa strain PaLo419 chromosome, complete genome

```
product length = 202
Forward primer  1        TGCCTGGAACATAATCACCGT  21
Template        2929283  ............G........  2929303

Reverse primer  1        GTCGGTAGATAGCCTGTCGC  20
Template        2929484  ....................  2929465
```

>CP075792.1 Pseudomonas aeruginosa strain PaLo422 chromosome, complete genome

```
product length = 202
Forward primer  1        TGCCTGGAACATAATCACCGT  21
Template        3151969  ............G........  3151989

Reverse primer  1        GTCGGTAGATAGCCTGTCGC  20
Template        3152170  ....................  3152151
```

>CP075788.1 Pseudomonas aeruginosa strain PaLo502 chromosome, complete genome

```
product length = 202
Forward primer  1        TGCCTGGAACATAATCACCGT  21
Template        2797001  ............G........  2797021

Reverse primer  1        GTCGGTAGATAGCCTGTCGC  20
Template        2797202  ....................  2797183
```

>CP075787.1 Pseudomonas aeruginosa strain PaLo504 chromosome, complete genome

```
product length = 202
Forward primer  1        TGCCTGGAACATAATCACCGT  21
Template        3150264  ............G........  3150284

Reverse primer  1        GTCGGTAGATAGCCTGTCGC  20
Template        3150465  ....................  3150446
```

>CP075785.1 Pseudomonas aeruginosa strain PaLo505 chromosome, complete genome

```
product length = 202
Forward primer  1        TGCCTGGAACATAATCACCGT  21
Template        3264300  ............G........  3264320

Reverse primer  1        GTCGGTAGATAGCCTGTCGC  20
Template        3264501  ....................  3264482
```

>CP075783.1 Pseudomonas aeruginosa strain PaLo508 chromosome, complete genome

```
product length = 202
Forward primer  1        TGCCTGGAACATAATCACCGT  21
Template        3293485  ............G........  3293505

Reverse primer  1        GTCGGTAGATAGCCTGTCGC  20
Template        3293686  ....................  3293667
```

>CP075782.1 Pseudomonas aeruginosa strain PaLo509 chromosome, complete genome

```
product length = 202
Forward primer  1        TGCCTGGAACATAATCACCGT  21
Template        3293480  ............G........  3293500

Reverse primer  1        GTCGGTAGATAGCCTGTCGC  20
Template        3293681  ....................  3293662
```

>CP075781.1 Pseudomonas aeruginosa strain PaLo512 chromosome, complete genome

```
product length = 202
Forward primer  1        TGCCTGGAACATAATCACCGT  21
Template        3459211  ............G........  3459231

Reverse primer  1        GTCGGTAGATAGCCTGTCGC  20
Template        3459412  ....................  3459393
```

>CP075780.1 Pseudomonas aeruginosa strain PaLo524 chromosome, complete genome

```
product length = 202
Forward primer  1        TGCCTGGAACATAATCACCGT  21
Template        3200604  ............G........  3200624

Reverse primer  1        GTCGGTAGATAGCCTGTCGC  20
Template        3200805  ....................  3200786
```

>CP075779.1 Pseudomonas aeruginosa strain PaLo526 chromosome, complete genome

```
product length = 202
Forward primer  1        TGCCTGGAACATAATCACCGT  21
Template        3095004  ............G........  3095024

Reverse primer  1        GTCGGTAGATAGCCTGTCGC  20
Template        3095205  ....................  3095186
```

>CP075778.1 Pseudomonas aeruginosa strain PaLo527 chromosome, complete genome

```
product length = 202
Forward primer  1        TGCCTGGAACATAATCACCGT  21
Template        3065949  ............G........  3065969

Reverse primer  1        GTCGGTAGATAGCCTGTCGC  20
Template        3066150  ....................  3066131
```

>CP075777.1 Pseudomonas aeruginosa strain PaLo528 chromosome, complete genome

```
product length = 202
Forward primer  1        TGCCTGGAACATAATCACCGT  21
Template        3148708  ............G........  3148728

Reverse primer  1        GTCGGTAGATAGCCTGTCGC  20
Template        3148909  ....................  3148890
```

>CP075776.1 Pseudomonas aeruginosa strain PaLo529 chromosome, complete genome

```
product length = 202
Forward primer  1        TGCCTGGAACATAATCACCGT  21
Template        3156989  ............G........  3157009

Reverse primer  1        GTCGGTAGATAGCCTGTCGC  20
Template        3157190  ....................  3157171
```

>CP075771.1 Pseudomonas aeruginosa strain PaLo532 chromosome, complete genome

```
product length = 202
Forward primer  1        TGCCTGGAACATAATCACCGT  21
Template        3434256  ............G........  3434276

Reverse primer  1        GTCGGTAGATAGCCTGTCGC  20
Template        3434457  ....................  3434438
```

>CP075768.1 Pseudomonas aeruginosa strain PaLo535 chromosome

```
product length = 202
Forward primer  1        TGCCTGGAACATAATCACCGT  21
Template        2985403  ............G........  2985423

Reverse primer  1        GTCGGTAGATAGCCTGTCGC  20
Template        2985604  ....................  2985585
```

>CP075767.1 Pseudomonas aeruginosa strain PaLo536 chromosome, complete genome

```
product length = 202
Forward primer  1        TGCCTGGAACATAATCACCGT  21
Template        3155955  ............G........  3155975

Reverse primer  1        GTCGGTAGATAGCCTGTCGC  20
Template        3156156  ....................  3156137
```

>CP075766.1 Pseudomonas aeruginosa strain PaLo538 chromosome, complete genome

```
product length = 202
Forward primer  1        TGCCTGGAACATAATCACCGT  21
Template        3371215  ............G........  3371235

Reverse primer  1        GTCGGTAGATAGCCTGTCGC  20
Template        3371416  ....................  3371397
```

>CP075763.1 Pseudomonas aeruginosa strain PaLo543 chromosome, complete genome

```
product length = 202
Forward primer  1        TGCCTGGAACATAATCACCGT  21
Template        3563180  ............G........  3563200

Reverse primer  1        GTCGGTAGATAGCCTGTCGC  20
Template        3563381  ....................  3563362
```

>CP075761.1 Pseudomonas aeruginosa strain PaLo545 chromosome

```
product length = 202
Forward primer  1        TGCCTGGAACATAATCACCGT  21
Template        3824596  ............G........  3824576

Reverse primer  1        GTCGGTAGATAGCCTGTCGC  20
Template        3824395  ....................  3824414
```

>CP075760.1 Pseudomonas aeruginosa strain PaLo550 chromosome, complete genome

```
product length = 202
Forward primer  1        TGCCTGGAACATAATCACCGT  21
Template        3217919  ............G........  3217939

Reverse primer  1        GTCGGTAGATAGCCTGTCGC  20
Template        3218120  ....................  3218101
```

>CP075755.1 Pseudomonas aeruginosa strain PaLo553 chromosome, complete genome

```
product length = 202
Forward primer  1        TGCCTGGAACATAATCACCGT  21
Template        3468106  ............G........  3468086

Reverse primer  1        GTCGGTAGATAGCCTGTCGC  20
Template        3467905  ....................  3467924
```

>CP075754.1 Pseudomonas aeruginosa strain PaLo555 chromosome, complete genome

```
product length = 202
Forward primer  1        TGCCTGGAACATAATCACCGT  21
Template        3068781  ............G........  3068801

Reverse primer  1        GTCGGTAGATAGCCTGTCGC  20
Template        3068982  ....................  3068963
```

>CP075753.1 Pseudomonas aeruginosa strain PaLo556 chromosome, complete genome

```
product length = 202
Forward primer  1        TGCCTGGAACATAATCACCGT  21
Template        3122134  ............G........  3122154

Reverse primer  1        GTCGGTAGATAGCCTGTCGC  20
Template        3122335  ....................  3122316
```

>CP075752.1 Pseudomonas aeruginosa strain PaLo557 chromosome, complete genome

```
product length = 202
Forward primer  1        TGCCTGGAACATAATCACCGT  21
Template        3223621  ............G........  3223641

Reverse primer  1        GTCGGTAGATAGCCTGTCGC  20
Template        3223822  ....................  3223803
```

>CP075751.1 Pseudomonas aeruginosa strain PaLo561 chromosome, complete genome

```
product length = 202
Forward primer  1        TGCCTGGAACATAATCACCGT  21
Template        3262490  ............G........  3262510

Reverse primer  1        GTCGGTAGATAGCCTGTCGC  20
Template        3262691  ....................  3262672
```

>CP075750.1 Pseudomonas aeruginosa strain PaLo563 chromosome, complete genome

```
product length = 202
Forward primer  1        TGCCTGGAACATAATCACCGT  21
Template        3582277  ............G........  3582257

Reverse primer  1        GTCGGTAGATAGCCTGTCGC  20
Template        3582076  ....................  3582095
```

>CP075749.1 Pseudomonas aeruginosa strain PaLo564 chromosome, complete genome

```
product length = 202
Forward primer  1        TGCCTGGAACATAATCACCGT  21
Template        3228979  ............G........  3228999

Reverse primer  1        GTCGGTAGATAGCCTGTCGC  20
Template        3229180  ....................  3229161
```

>CP075748.1 Pseudomonas aeruginosa strain PaLo565 chromosome, complete genome

```
product length = 202
Forward primer  1        TGCCTGGAACATAATCACCGT  21
Template        4192980  ............G........  4193000

Reverse primer  1        GTCGGTAGATAGCCTGTCGC  20
Template        4193181  ....................  4193162
```

>CP116723.1 Pseudomonas aeruginosa strain 2872 chromosome

```
product length = 202
Forward primer  1        TGCCTGGAACATAATCACCGT  21
Template        3762137  ............G........  3762117

Reverse primer  1        GTCGGTAGATAGCCTGTCGC  20
Template        3761936  ....................  3761955
```

>CP116725.1 Pseudomonas aeruginosa strain 2881 chromosome, complete genome

```
product length = 202
Forward primer  1        TGCCTGGAACATAATCACCGT  21
Template        3454682  ............G........  3454702

Reverse primer  1        GTCGGTAGATAGCCTGTCGC  20
Template        3454883  ....................  3454864
```

>CP116722.1 Pseudomonas aeruginosa strain 2868 chromosome, complete genome

```
product length = 202
Forward primer  1        TGCCTGGAACATAATCACCGT  21
Template        3547307  ............G........  3547327

Reverse primer  1        GTCGGTAGATAGCCTGTCGC  20
Template        3547508  ....................  3547489
```

>CP116717.1 Pseudomonas aeruginosa strain 2857 chromosome, complete genome

```
product length = 202
Forward primer  1        TGCCTGGAACATAATCACCGT  21
Template        3608320  ............G........  3608340

Reverse primer  1        GTCGGTAGATAGCCTGTCGC  20
Template        3608521  ....................  3608502
```

>CP116727.1 Pseudomonas aeruginosa strain 2875 chromosome, complete genome

```
product length = 202
Forward primer  1        TGCCTGGAACATAATCACCGT  21
Template        3442959  ............G........  3442979

Reverse primer  1        GTCGGTAGATAGCCTGTCGC  20
Template        3443160  ....................  3443141
```

>CP116718.1 Pseudomonas aeruginosa strain 2858 chromosome, complete genome

```
product length = 202
Forward primer  1        TGCCTGGAACATAATCACCGT  21
Template        4066877  ............G........  4066897

Reverse primer  1        GTCGGTAGATAGCCTGTCGC  20
Template        4067078  ....................  4067059
```

>CP116720.1 Pseudomonas aeruginosa strain 2866 chromosome, complete genome

```
product length = 202
Forward primer  1        TGCCTGGAACATAATCACCGT  21
Template        3410902  ............G........  3410922

Reverse primer  1        GTCGGTAGATAGCCTGTCGC  20
Template        3411103  ....................  3411084
```

>CP116724.1 Pseudomonas aeruginosa strain 2880 chromosome, complete genome

```
product length = 202
Forward primer  1        TGCCTGGAACATAATCACCGT  21
Template        3466771  ............G........  3466791

Reverse primer  1        GTCGGTAGATAGCCTGTCGC  20
Template        3466972  ....................  3466953
```

>CP116715.1 Pseudomonas aeruginosa strain 2856 chromosome, complete genome

```
product length = 202
Forward primer  1        TGCCTGGAACATAATCACCGT  21
Template        3392731  ............G........  3392751

Reverse primer  1        GTCGGTAGATAGCCTGTCGC  20
Template        3392932  ....................  3392913
```

>CP106784.1 Pseudomonas aeruginosa strain NY5085 chromosome, complete genome

```
product length = 202
Forward primer  1        TGCCTGGAACATAATCACCGT  21
Template        3278296  ............G........  3278316

Reverse primer  1        GTCGGTAGATAGCCTGTCGC  20
Template        3278497  ....................  3278478
```

>CP096913.1 Pseudomonas aeruginosa strain NY7610 chromosome, complete genome

```
product length = 202
Forward primer  1        TGCCTGGAACATAATCACCGT  21
Template        3330989  ............G........  3331009

Reverse primer  1        GTCGGTAGATAGCCTGTCGC  20
Template        3331190  ....................  3331171
```

>CP096912.1 Pseudomonas aeruginosa strain NY7770 chromosome, complete genome

```
product length = 202
Forward primer  1        TGCCTGGAACATAATCACCGT  21
Template        3341430  ............G........  3341410

Reverse primer  1        GTCGGTAGATAGCCTGTCGC  20
Template        3341229  ....................  3341248
```

>CP096909.1 Pseudomonas aeruginosa strain NY8688 chromosome, complete genome

```
product length = 202
Forward primer  1        TGCCTGGAACATAATCACCGT  21
Template        3558601  ............G........  3558581

Reverse primer  1        GTCGGTAGATAGCCTGTCGC  20
Template        3558400  ....................  3558419
```

>CP111030.1 Pseudomonas aeruginosa strain PALA38 chromosome, complete genome

```
product length = 202
Forward primer  1        TGCCTGGAACATAATCACCGT  21
Template        3287091  ............G........  3287111

Reverse primer  1        GTCGGTAGATAGCCTGTCGC  20
Template        3287292  ....................  3287273
```

>CP111032.1 Pseudomonas aeruginosa strain PALA54 chromosome, complete genome

```
product length = 202
Forward primer  1        TGCCTGGAACATAATCACCGT  21
Template        3288899  ............G........  3288919

Reverse primer  1        GTCGGTAGATAGCCTGTCGC  20
Template        3289100  ....................  3289081
```

>CP111034.1 Pseudomonas aeruginosa strain PALA50 chromosome, complete genome

```
product length = 202
Forward primer  1        TGCCTGGAACATAATCACCGT  21
Template        3149831  ............G........  3149851

Reverse primer  1        GTCGGTAGATAGCCTGTCGC  20
Template        3150032  ....................  3150013
```

>CP110353.1 Pseudomonas aeruginosa strain PALA48 chromosome, complete genome

```
product length = 202
Forward primer  1        TGCCTGGAACATAATCACCGT  21
Template        3328665  ............G........  3328685

Reverse primer  1        GTCGGTAGATAGCCTGTCGC  20
Template        3328866  ....................  3328847
```

>CP110352.1 Pseudomonas aeruginosa strain PALA47 chromosome, complete genome

```
product length = 202
Forward primer  1        TGCCTGGAACATAATCACCGT  21
Template        3122601  ............G........  3122621

Reverse primer  1        GTCGGTAGATAGCCTGTCGC  20
Template        3122802  ....................  3122783
```

>CP110350.1 Pseudomonas aeruginosa strain PALA44 chromosome, complete genome

```
product length = 202
Forward primer  1        TGCCTGGAACATAATCACCGT  21
Template        4488301  ............G........  4488321

Reverse primer  1        GTCGGTAGATAGCCTGTCGC  20
Template        4488502  ....................  4488483
```

>CP109931.1 Pseudomonas aeruginosa strain PALA42 chromosome, complete genome

```
product length = 202
Forward primer  1        TGCCTGGAACATAATCACCGT  21
Template        3791205  ............G........  3791185

Reverse primer  1        GTCGGTAGATAGCCTGTCGC  20
Template        3791004  ....................  3791023
```

>CP110349.1 Pseudomonas aeruginosa strain PALA40 chromosome, complete genome

```
product length = 202
Forward primer  1        TGCCTGGAACATAATCACCGT  21
Template        3610356  ............G........  3610376

Reverse primer  1        GTCGGTAGATAGCCTGTCGC  20
Template        3610557  ....................  3610538
```

>CP109920.1 Pseudomonas aeruginosa strain PALA39 chromosome, complete genome

```
product length = 202
Forward primer  1        TGCCTGGAACATAATCACCGT  21
Template        3346166  ............G........  3346186

Reverse primer  1        GTCGGTAGATAGCCTGTCGC  20
Template        3346367  ....................  3346348
```

>CP110348.1 Pseudomonas aeruginosa strain PALA36 chromosome, complete genome

```
product length = 202
Forward primer  1        TGCCTGGAACATAATCACCGT  21
Template        3391926  ............G........  3391946

Reverse primer  1        GTCGGTAGATAGCCTGTCGC  20
Template        3392127  ....................  3392108
```

>CP109918.1 Pseudomonas aeruginosa strain PALA55 chromosome, complete genome

```
product length = 202
Forward primer  1        TGCCTGGAACATAATCACCGT  21
Template        3078865  ............G........  3078885

Reverse primer  1        GTCGGTAGATAGCCTGTCGC  20
Template        3079066  ....................  3079047
```

>CP110347.1 Pseudomonas aeruginosa strain PALA52 chromosome, complete genome

```
product length = 202
Forward primer  1        TGCCTGGAACATAATCACCGT  21
Template        3061040  ............G........  3061060

Reverse primer  1        GTCGGTAGATAGCCTGTCGC  20
Template        3061241  ....................  3061222
```

>CP109851.1 Pseudomonas aeruginosa strain PALA51 chromosome, complete genome

```
product length = 202
Forward primer  1        TGCCTGGAACATAATCACCGT  21
Template        3127194  ............G........  3127214

Reverse primer  1        GTCGGTAGATAGCCTGTCGC  20
Template        3127395  ....................  3127376
```

>CP109850.1 Pseudomonas aeruginosa strain PALA37 chromosome, complete genome

```
product length = 202
Forward primer  1        TGCCTGGAACATAATCACCGT  21
Template        5674091  ............G........  5674071

Reverse primer  1        GTCGGTAGATAGCCTGTCGC  20
Template        5673890  ....................  5673909
```

>CP110346.1 Pseudomonas aeruginosa strain PALA35 chromosome, complete genome

```
product length = 202
Forward primer  1        TGCCTGGAACATAATCACCGT  21
Template        3210053  ............G........  3210073

Reverse primer  1        GTCGGTAGATAGCCTGTCGC  20
Template        3210254  ....................  3210235
```

>CP109849.1 Pseudomonas aeruginosa strain PALA34 chromosome, complete genome

```
product length = 202
Forward primer  1        TGCCTGGAACATAATCACCGT  21
Template        3464833  ............G........  3464853

Reverse primer  1        GTCGGTAGATAGCCTGTCGC  20
Template        3465034  ....................  3465015
```

>CP109845.1 Pseudomonas aeruginosa strain PALA33 chromosome, complete genome

```
product length = 202
Forward primer  1        TGCCTGGAACATAATCACCGT  21
Template        3421788  ............G........  3421808

Reverse primer  1        GTCGGTAGATAGCCTGTCGC  20
Template        3421989  ....................  3421970
```

>CP110345.1 Pseudomonas aeruginosa strain PALA30 chromosome, complete genome

```
product length = 202
Forward primer  1        TGCCTGGAACATAATCACCGT  21
Template        3379563  ............G........  3379583

Reverse primer  1        GTCGGTAGATAGCCTGTCGC  20
Template        3379764  ....................  3379745
```

>CP109843.1 Pseudomonas aeruginosa strain PALA29 chromosome, complete genome

```
product length = 202
Forward primer  1        TGCCTGGAACATAATCACCGT  21
Template        3223393  ............G........  3223413

Reverse primer  1        GTCGGTAGATAGCCTGTCGC  20
Template        3223594  ....................  3223575
```

>CP109835.1 Pseudomonas aeruginosa strain PALA26 chromosome, complete genome

```
product length = 202
Forward primer  1        TGCCTGGAACATAATCACCGT  21
Template        3203806  ............G........  3203826

Reverse primer  1        GTCGGTAGATAGCCTGTCGC  20
Template        3204007  ....................  3203988
```

>CP110344.1 Pseudomonas aeruginosa strain PALA24 chromosome, complete genome

```
product length = 202
Forward primer  1        TGCCTGGAACATAATCACCGT  21
Template        5203439  ............G........  5203419

Reverse primer  1        GTCGGTAGATAGCCTGTCGC  20
Template        5203238  ....................  5203257
```

>CP109833.1 Pseudomonas aeruginosa strain PALA23 chromosome, complete genome

```
product length = 202
Forward primer  1        TGCCTGGAACATAATCACCGT  21
Template        3170354  ............G........  3170374

Reverse primer  1        GTCGGTAGATAGCCTGTCGC  20
Template        3170555  ....................  3170536
```

>CP107064.1 Pseudomonas aeruginosa strain PALA20 chromosome, complete genome

```
product length = 202
Forward primer  1        TGCCTGGAACATAATCACCGT  21
Template        5123641  ............G........  5123621

Reverse primer  1        GTCGGTAGATAGCCTGTCGC  20
Template        5123440  ....................  5123459
```

>CP107029.1 Pseudomonas aeruginosa strain PALA19 chromosome, complete genome

```
product length = 202
Forward primer  1        TGCCTGGAACATAATCACCGT  21
Template        3820263  ............G........  3820243

Reverse primer  1        GTCGGTAGATAGCCTGTCGC  20
Template        3820062  ....................  3820081
```

>CP106745.1 Pseudomonas aeruginosa strain PALA17 chromosome, complete genome

```
product length = 202
Forward primer  1        TGCCTGGAACATAATCACCGT  21
Template        2995243  ............G........  2995263

Reverse primer  1        GTCGGTAGATAGCCTGTCGC  20
Template        2995444  ....................  2995425
```

>CP106744.1 Pseudomonas aeruginosa strain PALA16 chromosome, complete genome

```
product length = 202
Forward primer  1        TGCCTGGAACATAATCACCGT  21
Template        3386057  ............G........  3386077

Reverse primer  1        GTCGGTAGATAGCCTGTCGC  20
Template        3386258  ....................  3386239
```

>CP106742.1 Pseudomonas aeruginosa strain PALA14 chromosome, complete genome

```
product length = 202
Forward primer  1        TGCCTGGAACATAATCACCGT  21
Template        4241417  ............G........  4241397

Reverse primer  1        GTCGGTAGATAGCCTGTCGC  20
Template        4241216  ....................  4241235
```

>CP106682.1 Pseudomonas aeruginosa strain PALA13 chromosome, complete genome

```
product length = 202
Forward primer  1        TGCCTGGAACATAATCACCGT  21
Template        3178393  ............G........  3178373

Reverse primer  1        GTCGGTAGATAGCCTGTCGC  20
Template        3178192  ....................  3178211
```

>CP106681.1 Pseudomonas aeruginosa strain PALA12 chromosome, complete genome

```
product length = 202
Forward primer  1        TGCCTGGAACATAATCACCGT  21
Template        2292834  ............G........  2292814

Reverse primer  1        GTCGGTAGATAGCCTGTCGC  20
Template        2292633  ....................  2292652
```

>CP106680.1 Pseudomonas aeruginosa strain PALA11 chromosome, complete genome

```
product length = 202
Forward primer  1        TGCCTGGAACATAATCACCGT  21
Template        3098205  ............G........  3098225

Reverse primer  1        GTCGGTAGATAGCCTGTCGC  20
Template        3098406  ....................  3098387
```

>CP104870.1 Pseudomonas aeruginosa strain PALA9 chromosome, complete genome

```
product length = 202
Forward primer  1        TGCCTGGAACATAATCACCGT  21
Template        3361879  ............G........  3361899

Reverse primer  1        GTCGGTAGATAGCCTGTCGC  20
Template        3362080  ....................  3362061
```

>CP104869.1 Pseudomonas aeruginosa strain PALA8 chromosome, complete genome

```
product length = 202
Forward primer  1        TGCCTGGAACATAATCACCGT  21
Template        3267432  ............G........  3267452

Reverse primer  1        GTCGGTAGATAGCCTGTCGC  20
Template        3267633  ....................  3267614
```

>CP104868.1 Pseudomonas aeruginosa strain PALA7 chromosome, complete genome

```
product length = 202
Forward primer  1        TGCCTGGAACATAATCACCGT  21
Template        3122234  ............G........  3122254

Reverse primer  1        GTCGGTAGATAGCCTGTCGC  20
Template        3122435  ....................  3122416
```

>CP104867.1 Pseudomonas aeruginosa strain PALA6 chromosome, complete genome

```
product length = 202
Forward primer  1        TGCCTGGAACATAATCACCGT  21
Template        2951696  ............G........  2951676

Reverse primer  1        GTCGGTAGATAGCCTGTCGC  20
Template        2951495  ....................  2951514
```

>CP104866.1 Pseudomonas aeruginosa strain PALA4 chromosome, complete genome

```
product length = 202
Forward primer  1        TGCCTGGAACATAATCACCGT  21
Template        3235944  ............G........  3235964

Reverse primer  1        GTCGGTAGATAGCCTGTCGC  20
Template        3236145  ....................  3236126
```

>CP104865.1 Pseudomonas aeruginosa strain PALA2 chromosome, complete genome

```
product length = 202
Forward primer  1        TGCCTGGAACATAATCACCGT  21
Template        3165571  ............G........  3165551

Reverse primer  1        GTCGGTAGATAGCCTGTCGC  20
Template        3165370  ....................  3165389
```

>CP104254.1 Pseudomonas aeruginosa strain PALA1 chromosome, complete genome

```
product length = 202
Forward primer  1        TGCCTGGAACATAATCACCGT  21
Template        3355680  ............G........  3355700

Reverse primer  1        GTCGGTAGATAGCCTGTCGC  20
Template        3355881  ....................  3355862
```

>CP114761.1 Pseudomonas aeruginosa strain NF143349 chromosome, complete genome

```
product length = 202
Forward primer  1        TGCCTGGAACATAATCACCGT  21
Template        3364400  ............G........  3364420

Reverse primer  1        GTCGGTAGATAGCCTGTCGC  20
Template        3364601  ....................  3364582
```

>CP114374.1 Pseudomonas aeruginosa strain Jade-X chromosome, complete genome

```
product length = 202
Forward primer  1        TGCCTGGAACATAATCACCGT  21
Template        3138942  ............G........  3138962

Reverse primer  1        GTCGGTAGATAGCCTGTCGC  20
Template        3139143  ....................  3139124
```

>CP113974.1 Pseudomonas aeruginosa strain M6A146 chromosome, complete genome

```
product length = 202
Forward primer  1        TGCCTGGAACATAATCACCGT  21
Template        1612497  ............G........  1612517

Reverse primer  1        GTCGGTAGATAGCCTGTCGC  20
Template        1612698  ....................  1612679
```

>CP097555.1 Pseudomonas aeruginosa strain B1.2 chromosome, complete genome

```
product length = 202
Forward primer  1        TGCCTGGAACATAATCACCGT  21
Template        3130640  ............G........  3130660

Reverse primer  1        GTCGGTAGATAGCCTGTCGC  20
Template        3130841  ....................  3130822
```

>CP097556.1 Pseudomonas aeruginosa strain B2.1 chromosome, complete genome

```
product length = 202
Forward primer  1        TGCCTGGAACATAATCACCGT  21
Template        3130627  ............G........  3130647

Reverse primer  1        GTCGGTAGATAGCCTGTCGC  20
Template        3130828  ....................  3130809
```

>CP097557.1 Pseudomonas aeruginosa strain C1.3 chromosome, complete genome

```
product length = 202
Forward primer  1        TGCCTGGAACATAATCACCGT  21
Template        3296412  ............G........  3296392

Reverse primer  1        GTCGGTAGATAGCCTGTCGC  20
Template        3296211  ....................  3296230
```

>CP097560.1 Pseudomonas aeruginosa strain C4.2 chromosome, complete genome

```
product length = 202
Forward primer  1        TGCCTGGAACATAATCACCGT  21
Template        3406232  ............G........  3406252

Reverse primer  1        GTCGGTAGATAGCCTGTCGC  20
Template        3406433  ....................  3406414
```

>CP113230.1 Pseudomonas aeruginosa strain BIAI 160 chromosome, complete genome

```
product length = 202
Forward primer  1        TGCCTGGAACATAATCACCGT  21
Template        4950735  ............G........  4950755

Reverse primer  1        GTCGGTAGATAGCCTGTCGC  20
Template        4950936  ....................  4950917
```

>CP113246.1 Pseudomonas aeruginosa strain SMC4386 chromosome, complete genome

```
product length = 202
Forward primer  1        TGCCTGGAACATAATCACCGT  21
Template        3229498  ............G........  3229478

Reverse primer  1        GTCGGTAGATAGCCTGTCGC  20
Template        3229297  ....................  3229316
```

>CP113106.1 Pseudomonas aeruginosa strain BIAI 157 chromosome, complete genome

```
product length = 202
Forward primer  1        TGCCTGGAACATAATCACCGT  21
Template        2174631  ............G........  2174651

Reverse primer  1        GTCGGTAGATAGCCTGTCGC  20
Template        2174832  ....................  2174813
```

>CP097857.1 Pseudomonas sp. B111 chromosome, complete genome

```
product length = 202
Forward primer  1        TGCCTGGAACATAATCACCGT  21
Template        2427072  ............G........  2427092

Reverse primer  1        GTCGGTAGATAGCCTGTCGC  20
Template        2427273  ....................  2427254
```

>CP102441.2 Pseudomonas aeruginosa strain PA30 chromosome, complete genome

```
product length = 202
Forward primer  1        TGCCTGGAACATAATCACCGT  21
Template        3392800  ............G........  3392780

Reverse primer  1        GTCGGTAGATAGCCTGTCGC  20
Template        3392599  ....................  3392618
```

>CP083357.1 Pseudomonas aeruginosa strain KPA143 chromosome, complete genome

```
product length = 202
Forward primer  1        TGCCTGGAACATAATCACCGT  21
Template        6175635  ............G........  6175655

Reverse primer  1        GTCGGTAGATAGCCTGTCGC  20
Template        6175836  ....................  6175817
```

>CP083359.1 Pseudomonas aeruginosa strain KPA159 chromosome, complete genome

```
product length = 203
Forward primer  1        TGCCTGGAACATAATCACCGT  21
Template        6006603  ............G........  6006623

Reverse primer  1        GTCGGTAGATAGCCTGTCGC  20
Template        6006805  ....................  6006786
```

>CP083358.1 Pseudomonas aeruginosa strain KPA151 chromosome, complete genome

```
product length = 205
Forward primer  1        TGCCTGGAACATAATCACCGT  21
Template        3999023  ............G........  3999003

Reverse primer  1        GTCGGTAGATAGCCTGTCGC  20
Template        3998819  ....................  3998838
```

>CP083355.1 Pseudomonas aeruginosa strain KPA134 chromosome, complete genome

```
product length = 202
Forward primer  1        TGCCTGGAACATAATCACCGT  21
Template        4074515  ............G........  4074535

Reverse primer  1        GTCGGTAGATAGCCTGTCGC  20
Template        4074716  ....................  4074697
```

>CP083356.1 Pseudomonas aeruginosa strain KPA140 chromosome, complete genome

```
product length = 203
Forward primer  1        TGCCTGGAACATAATCACCGT  21
Template        3934407  ............G........  3934387

Reverse primer  1        GTCGGTAGATAGCCTGTCGC  20
Template        3934205  ....................  3934224
```

>CP083353.1 Pseudomonas aeruginosa strain KPA120 chromosome, complete genome

```
product length = 203
Forward primer  1        TGCCTGGAACATAATCACCGT  21
Template        5947600  ............G........  5947620

Reverse primer  1        GTCGGTAGATAGCCTGTCGC  20
Template        5947802  ....................  5947783
```

>CP083354.1 Pseudomonas aeruginosa strain KPA124 chromosome, complete genome

```
product length = 205
Forward primer  1        TGCCTGGAACATAATCACCGT  21
Template        4078351  ............G........  4078371

Reverse primer  1        GTCGGTAGATAGCCTGTCGC  20
Template        4078555  ....................  4078536
```

>CP107257.1 Pseudomonas aeruginosa strain 2019CK-00034 chromosome, complete genome

```
product length = 202
Forward primer  1        TGCCTGGAACATAATCACCGT  21
Template        4537759  ............G........  4537739

Reverse primer  1        GTCGGTAGATAGCCTGTCGC  20
Template        4537558  ....................  4537577
```

>CP104565.1 Pseudomonas aeruginosa strain HS_121 chromosome, complete genome

```
product length = 202
Forward primer  1        TGCCTGGAACATAATCACCGT  21
Template        3157930  ............G........  3157950

Reverse primer  1        GTCGGTAGATAGCCTGTCGC  20
Template        3158131  ....................  3158112
```

>CP104567.1 Pseudomonas aeruginosa strain HS_13 chromosome, complete genome

```
product length = 202
Forward primer  1        TGCCTGGAACATAATCACCGT  21
Template        3107965  ............G........  3107985

Reverse primer  1        GTCGGTAGATAGCCTGTCGC  20
Template        3108166  ....................  3108147
```

>CP107042.1 Pseudomonas aeruginosa strain GIMC5035:PA21/2013 chromosome

```
product length = 202
Forward primer  1        TGCCTGGAACATAATCACCGT  21
Template        1883552  ............G........  1883572

Reverse primer  1        GTCGGTAGATAGCCTGTCGC  20
Template        1883753  ....................  1883734
```

>CP086213.1 Pseudomonas aeruginosa strain Pa3 chromosome, complete genome

```
product length = 202
Forward primer  1        TGCCTGGAACATAATCACCGT  21
Template        3569069  ............G........  3569089

Reverse primer  1        GTCGGTAGATAGCCTGTCGC  20
Template        3569270  ....................  3569251
```

>CP104695.1 Pseudomonas aeruginosa strain 2021CK-01281 chromosome

```
product length = 202
Forward primer  1        TGCCTGGAACATAATCACCGT  21
Template        1783251  ............G........  1783271

Reverse primer  1        GTCGGTAGATAGCCTGTCGC  20
Template        1783452  ....................  1783433
```

>CP104590.1 Pseudomonas aeruginosa strain WTJH36 chromosome, complete genome

```
product length = 202
Forward primer  1       TGCCTGGAACATAATCACCGT  21
Template        855601  ............G........  855621

Reverse primer  1       GTCGGTAGATAGCCTGTCGC  20
Template        855802  ....................  855783
```

>CP104588.1 Pseudomonas aeruginosa strain WTJH32 chromosome, complete genome

```
product length = 202
Forward primer  1        TGCCTGGAACATAATCACCGT  21
Template        3477526  ............G........  3477546

Reverse primer  1        GTCGGTAGATAGCCTGTCGC  20
Template        3477727  ....................  3477708
```

>CP104584.1 Pseudomonas aeruginosa strain WTJH2 chromosome, complete genome

```
product length = 202
Forward primer  1        TGCCTGGAACATAATCACCGT  21
Template        3477521  ............G........  3477541

Reverse primer  1        GTCGGTAGATAGCCTGTCGC  20
Template        3477722  ....................  3477703
```

>CP104170.1 Pseudomonas aeruginosa strain HW001G chromosome, complete genome

```
product length = 202
Forward primer  1        TGCCTGGAACATAATCACCGT  21
Template        2508739  ............G........  2508759

Reverse primer  1        GTCGGTAGATAGCCTGTCGC  20
Template        2508940  ....................  2508921
```

>CP096207.1 Pseudomonas aeruginosa TBCF10839 chromosome, complete genome

```
product length = 202
Forward primer  1        TGCCTGGAACATAATCACCGT  21
Template        2115541  ............G........  2115521

Reverse primer  1        GTCGGTAGATAGCCTGTCGC  20
Template        2115340  ....................  2115359
```

>CP101885.1 Pseudomonas aeruginosa strain M27432 chromosome, complete genome

```
product length = 202
Forward primer  1        TGCCTGGAACATAATCACCGT  21
Template        3740167  ............G........  3740147

Reverse primer  1        GTCGGTAGATAGCCTGTCGC  20
Template        3739966  ....................  3739985
```

>CP102946.1 Pseudomonas aeruginosa strain SCAID WND1-2022 (148) chromosome, complete genome

```
product length = 202
Forward primer  1        TGCCTGGAACATAATCACCGT  21
Template        3175432  ............G........  3175452

Reverse primer  1        GTCGGTAGATAGCCTGTCGC  20
Template        3175633  ....................  3175614
```

>CP102174.1 Pseudomonas aeruginosa strain PA5083 chromosome, complete genome

```
product length = 202
Forward primer  1        TGCCTGGAACATAATCACCGT  21
Template        3694999  ............G........  3694979

Reverse primer  1        GTCGGTAGATAGCCTGTCGC  20
Template        3694798  ....................  3694817
```

>CP101912.1 Pseudomonas aeruginosa strain ATCC 27853 chromosome, complete genome

```
product length = 202
Forward primer  1        TGCCTGGAACATAATCACCGT  21
Template        3207544  ............G........  3207564

Reverse primer  1        GTCGGTAGATAGCCTGTCGC  20
Template        3207745  ....................  3207726
```

>CP101911.1 Pseudomonas aeruginosa strain NWRC-1223 chromosome, complete genome

```
product length = 202
Forward primer  1        TGCCTGGAACATAATCACCGT  21
Template        3135176  ............G........  3135196

Reverse primer  1        GTCGGTAGATAGCCTGTCGC  20
Template        3135377  ....................  3135358
```

>CP101540.1 Pseudomonas aeruginosa strain D-2 chromosome, complete genome

```
product length = 202
Forward primer  1        TGCCTGGAACATAATCACCGT  21
Template        3124017  ............G........  3124037

Reverse primer  1        GTCGGTAGATAGCCTGTCGC  20
Template        3124218  ....................  3124199
```

>CP094851.1 Pseudomonas aeruginosa strain R20-14 chromosome, complete genome

```
product length = 202
Forward primer  1        TGCCTGGAACATAATCACCGT  21
Template        3100396  ............G........  3100416

Reverse primer  1        GTCGGTAGATAGCCTGTCGC  20
```

```
Template        3100597  ....................  3100578
```

>CP100760.1 Pseudomonas aeruginosa strain AX0001 chromosome

```
product length = 202
Forward primer  1        TGCCTGGAACATAATCACCGT  21
Template        4177246  ............G........  4177266

Reverse primer  1        GTCGGTAGATAGCCTGTCGC  20
Template        4177447  ....................  4177428
```

>CP100761.1 Pseudomonas aeruginosa strain PA0011 chromosome

```
product length = 202
Forward primer  1        TGCCTGGAACATAATCACCGT  21
Template        4177246  ............G........  4177266

Reverse primer  1        GTCGGTAGATAGCCTGTCGC  20
Template        4177447  ....................  4177428
```

>CP100759.1 Pseudomonas aeruginosa strain PA0009 chromosome

```
product length = 202
Forward primer  1        TGCCTGGAACATAATCACCGT  21
Template        4133537  ............G........  4133517

Reverse primer  1        GTCGGTAGATAGCCTGTCGC  20
Template        4133336  ....................  4133355
```

>CP097710.1 Pseudomonas aeruginosa strain PA-2 chromosome, complete genome

```
product length = 202
Forward primer  1        TGCCTGGAACATAATCACCGT  21
Template        3858240  ............G........  3858220

Reverse primer  1        GTCGGTAGATAGCCTGTCGC  20
Template        3858039  ....................  3858058
```

>CP097709.1 Pseudomonas aeruginosa strain PA-1 chromosome, complete genome

```
product length = 202
Forward primer  1        TGCCTGGAACATAATCACCGT  21
Template        3857137  ............G........  3857117

Reverse primer  1        GTCGGTAGATAGCCTGTCGC  20
Template        3856936  ....................  3856955
```

>CP100653.1 Pseudomonas aeruginosa strain F13 chromosome, complete genome

```
product length = 202
Forward primer  1        TGCCTGGAACATAATCACCGT  21
Template        3210847  ............G........  3210867
```

```
Reverse primer  1        GTCGGTAGATAGCCTGTCGC  20
Template        3211048  ....................  3211029
```

>CP091880.1 Pseudomonas aeruginosa strain US449 chromosome, complete genome

```
product length = 202
Forward primer  1        TGCCTGGAACATAATCACCGT  21
Template        3155052  ............G........  3155072

Reverse primer  1        GTCGGTAGATAGCCTGTCGC  20
Template        3155253  ....................  3155234
```

>CP069177.1 Pseudomonas aeruginosa strain Z154 chromosome, complete genome

```
product length = 202
Forward primer  1        TGCCTGGAACATAATCACCGT  21
Template        4010642  ............G........  4010622

Reverse primer  1        GTCGGTAGATAGCCTGTCGC  20
Template        4010441  ....................  4010460
```

>CP097575.1 Pseudomonas aeruginosa strain UNC_PaerCF25 chromosome, complete genome

```
product length = 202
Forward primer  1      TGCCTGGAACATAATCACCGT  21
Template        90916  ............G........  90936

Reverse primer  1      GTCGGTAGATAGCCTGTCGC  20
Template        91117  ....................  91098
```

>CP097383.1 Pseudomonas aeruginosa strain L00-a chromosome, complete genome

```
product length = 202
Forward primer  1        TGCCTGGAACATAATCACCGT  21
Template        3271357  ............G........  3271377

Reverse primer  1        GTCGGTAGATAGCCTGTCGC  20
Template        3271558  ....................  3271539
```

>CP096813.1 Pseudomonas aeruginosa strain 8D chromosome, complete genome

```
product length = 202
Forward primer  1        TGCCTGGAACATAATCACCGT  21
Template        4596050  ............G........  4596070

Reverse primer  1        GTCGGTAGATAGCCTGTCGC  20
Template        4596251  ....................  4596232
```

>CP095772.2 Pseudomonas aeruginosa strain 34Pae23 chromosome

```
product length = 202
Forward primer  1        TGCCTGGAACATAATCACCGT  21
Template        3214775  ............G........  3214795
```

```
Reverse primer  1        GTCGGTAGATAGCCTGTCGC  20
Template        3214976  ....................  3214957
```

>CP095923.1 Pseudomonas aeruginosa strain AR19438 chromosome, complete genome

```
product length = 202
Forward primer  1        TGCCTGGAACATAATCACCGT  21
Template        2873508  ............G........  2873488

Reverse primer  1        GTCGGTAGATAGCCTGTCGC  20
Template        2873307  ....................  2873326
```

>CP095920.1 Pseudomonas aeruginosa strain AR19640 chromosome, complete genome

```
product length = 202
Forward primer  1        TGCCTGGAACATAATCACCGT  21
Template        3074948  ............G........  3074968

Reverse primer  1        GTCGGTAGATAGCCTGTCGC  20
Template        3075149  ....................  3075130
```

>CP095922.1 Pseudomonas aeruginosa strain AR19583 chromosome, complete genome

```
product length = 202
Forward primer  1        TGCCTGGAACATAATCACCGT  21
Template        3067490  ............G........  3067510

Reverse primer  1        GTCGGTAGATAGCCTGTCGC  20
Template        3067691  ....................  3067672
```

>CP095770.1 Pseudomonas aeruginosa strain 34Pae36 chromosome, complete genome

```
product length = 202
Forward primer  1        TGCCTGGAACATAATCACCGT  21
Template        3663837  ............G........  3663857

Reverse primer  1        GTCGGTAGATAGCCTGTCGC  20
Template        3664038  ....................  3664019
```

>CP095774.1 Pseudomonas aeruginosa strain 34Pae8 chromosome, complete genome

```
product length = 202
Forward primer  1        TGCCTGGAACATAATCACCGT  21
Template        3327643  ............G........  3327623

Reverse primer  1        GTCGGTAGATAGCCTGTCGC  20
Template        3327442  ....................  3327461
```

>CP090649.1 Pseudomonas aeruginosa strain PA1609 chromosome, complete genome

```
product length = 202
Forward primer  1        TGCCTGGAACATAATCACCGT  21
```

```
Template        3196428  ...........G........  3196448

Reverse primer  1        GTCGGTAGATAGCCTGTCGC  20
Template        3196629  ....................  3196610
```

>CP090648.1 Pseudomonas aeruginosa strain PA1616 chromosome, complete genome

```
product length = 202
Forward primer  1        TGCCTGGAACATAATCACCGT  21
Template        5812433  ...........G........  5812453

Reverse primer  1        GTCGGTAGATAGCCTGTCGC  20
Template        5812634  ....................  5812615
```

>CP090647.1 Pseudomonas aeruginosa strain PA1681 chromosome, complete genome

```
product length = 202
Forward primer  1        TGCCTGGAACATAATCACCGT  21
Template        3088716  ...........G........  3088696

Reverse primer  1        GTCGGTAGATAGCCTGTCGC  20
Template        3088515  ....................  3088534
```

>CP050149.1 Pseudomonas aeruginosa strain CHA chromosome

```
product length = 202
Forward primer  1        TGCCTGGAACATAATCACCGT  21
Template        3172818  ...........G........  3172838

Reverse primer  1        GTCGGTAGATAGCCTGTCGC  20
Template        3173019  ....................  3173000
```

>CP050148.1 Pseudomonas aeruginosa strain AA43 chromosome, complete genome

```
product length = 202
Forward primer  1        TGCCTGGAACATAATCACCGT  21
Template        3046907  ...........G........  3046927

Reverse primer  1        GTCGGTAGATAGCCTGTCGC  20
Template        3047108  ....................  3047089
```

>CP050147.1 Pseudomonas aeruginosa strain A5803 chromosome, complete genome

```
product length = 202
Forward primer  1        TGCCTGGAACATAATCACCGT  21
Template        3309322  ...........G........  3309342

Reverse primer  1        GTCGGTAGATAGCCTGTCGC  20
Template        3309523  ....................  3309504
```

>CP063387.1 Pseudomonas aeruginosa strain ST1076_d100blood2 chromosome, complete genome

```
product length = 202
```

```
Forward primer  1        TGCCTGGAACATAATCACCGT  21
Template        3191206  ............G........  3191226

Reverse primer  1        GTCGGTAGATAGCCTGTCGC  20
Template        3191407  ....................  3191388
```

>CP047643.1 Pseudomonas aeruginosa CI27 chromosome, complete genome

```
product length = 202
Forward primer  1        TGCCTGGAACATAATCACCGT  21
Template        3280336  ............G........  3280356

Reverse primer  1        GTCGGTAGATAGCCTGTCGC  20
Template        3280537  ....................  3280518
```

>CP063390.1 Pseudomonas aeruginosa strain ST1076_d97burn1 chromosome, complete genome

```
product length = 202
Forward primer  1        TGCCTGGAACATAATCACCGT  21
Template        3191206  ............G........  3191226

Reverse primer  1        GTCGGTAGATAGCCTGTCGC  20
Template        3191407  ....................  3191388
```

>CP063389.1 Pseudomonas aeruginosa strain ST1076_d97burn2 chromosome, complete genome

```
product length = 202
Forward primer  1        TGCCTGGAACATAATCACCGT  21
Template        3191206  ............G........  3191226

Reverse primer  1        GTCGGTAGATAGCCTGTCGC  20
Template        3191407  ....................  3191388
```

>CP063388.1 Pseudomonas aeruginosa strain ST1076_d100blood1 chromosome, complete genome

```
product length = 202
Forward primer  1        TGCCTGGAACATAATCACCGT  21
Template        3191206  ............G........  3191226

Reverse primer  1        GTCGGTAGATAGCCTGTCGC  20
Template        3191407  ....................  3191388
```

>CP063386.1 Pseudomonas aeruginosa strain ST1076_d118limb1 chromosome, complete genome

```
product length = 202
Forward primer  1        TGCCTGGAACATAATCACCGT  21
Template        3183403  ............G........  3183423

Reverse primer  1        GTCGGTAGATAGCCTGTCGC  20
Template        3183604  ....................  3183585
```

>CP063385.1 Pseudomonas aeruginosa strain St1076_d123blood chromosome, complete genome

```
product length = 202
Forward primer  1        TGCCTGGAACATAATCACCGT  21
Template        3191206  ............G........  3191226

Reverse primer  1        GTCGGTAGATAGCCTGTCGC  20
Template        3191407  ....................  3191388
```

>CP093967.1 Pseudomonas aeruginosa strain NY4605 chromosome, complete genome

```
product length = 202
Forward primer  1        TGCCTGGAACATAATCACCGT  21
Template        3087361  ............G........  3087381

Reverse primer  1        GTCGGTAGATAGCCTGTCGC  20
Template        3087562  ....................  3087543
```

>CP093966.1 Pseudomonas aeruginosa strain ATCC BAA-2114 chromosome, complete genome

```
product length = 202
Forward primer  1        TGCCTGGAACATAATCACCGT  21
Template        3057396  ............G........  3057416

Reverse primer  1        GTCGGTAGATAGCCTGTCGC  20
Template        3057597  ....................  3057578
```

>CP093395.1 Pseudomonas aeruginosa strain PA1_NCHU chromosome, complete genome

```
product length = 202
Forward primer  1        TGCCTGGAACATAATCACCGT  21
Template        1444161  ............G........  1444141

Reverse primer  1        GTCGGTAGATAGCCTGTCGC  20
Template        1443960  ....................  1443979
```

>CP093358.1 Pseudomonas aeruginosa strain E167 chromosome, complete genome

```
product length = 202
Forward primer  1        TGCCTGGAACATAATCACCGT  21
Template        3307178  ............G........  3307198

Reverse primer  1        GTCGGTAGATAGCCTGTCGC  20
Template        3307379  ....................  3307360
```

>CP093356.1 Pseudomonas aeruginosa strain E125 chromosome, complete genome

```
product length = 202
Forward primer  1        TGCCTGGAACATAATCACCGT  21
Template        3421068  ............G........  3421088

Reverse primer  1        GTCGGTAGATAGCCTGTCGC  20
Template        3421269  ....................  3421250
```

>CP093357.1 Pseudomonas aeruginosa strain E131 chromosome, complete genome

```
product length = 202
Forward primer  1        TGCCTGGAACATAATCACCGT  21
Template        3225803  ............G........  3225823

Reverse primer  1        GTCGGTAGATAGCCTGTCGC  20
Template        3226004  ....................  3225985
```

>CP093355.1 Pseudomonas aeruginosa strain E104 chromosome, complete genome

```
product length = 202
Forward primer  1        TGCCTGGAACATAATCACCGT  21
Template        3309300  ............G........  3309320

Reverse primer  1        GTCGGTAGATAGCCTGTCGC  20
Template        3309501  ....................  3309482
```

>CP093354.1 Pseudomonas aeruginosa strain E113 chromosome, complete genome

```
product length = 202
Forward primer  1        TGCCTGGAACATAATCACCGT  21
Template        3319738  ............G........  3319758

Reverse primer  1        GTCGGTAGATAGCCTGTCGC  20
Template        3319939  ....................  3319920
```

>CP093028.1 Pseudomonas aeruginosa strain H05 chromosome, complete genome

```
product length = 202
Forward primer  1        TGCCTGGAACATAATCACCGT  21
Template        3308587  ............G........  3308607

Reverse primer  1        GTCGGTAGATAGCCTGTCGC  20
Template        3308788  ....................  3308769
```

>CP093030.1 Pseudomonas aeruginosa strain H04 chromosome, complete genome

```
product length = 202
Forward primer  1        TGCCTGGAACATAATCACCGT  21
Template        3371763  ............G........  3371783

Reverse primer  1        GTCGGTAGATAGCCTGTCGC  20
Template        3371964  ....................  3371945
```

>CP093032.1 Pseudomonas aeruginosa strain H02 chromosome, complete genome

```
product length = 202
Forward primer  1        TGCCTGGAACATAATCACCGT  21
Template        3435639  ............G........  3435659

Reverse primer  1        GTCGGTAGATAGCCTGTCGC  20
Template        3435840  ....................  3435821
```

>CP093031.1 Pseudomonas aeruginosa strain H03 chromosome, complete genome

```
product length = 202
Forward primer  1        TGCCTGGAACATAATCACCGT  21
Template        3394291  ............G........  3394311

Reverse primer  1        GTCGGTAGATAGCCTGTCGC  20
Template        3394492  ....................  3394473
```

>CP093024.1 Pseudomonas aeruginosa strain H06 chromosome

```
product length = 202
Forward primer  1      TGCCTGGAACATAATCACCGT  21
Template        10821  ............G........  10801

Reverse primer  1      GTCGGTAGATAGCCTGTCGC  20
Template        10620  ....................  10639
```

>CP093013.1 Pseudomonas aeruginosa strain H19 chromosome

```
product length = 202
Forward primer  1        TGCCTGGAACATAATCACCGT  21
Template        6610340  ............G........  6610320

Reverse primer  1        GTCGGTAGATAGCCTGTCGC  20
Template        6610139  ....................  6610158
```

>CP093022.1 Pseudomonas aeruginosa strain H08 chromosome, complete genome

```
product length = 202
Forward primer  1        TGCCTGGAACATAATCACCGT  21
Template        3309323  ............G........  3309343

Reverse primer  1        GTCGGTAGATAGCCTGTCGC  20
Template        3309524  ....................  3309505
```

>CP093023.1 Pseudomonas aeruginosa strain H07 chromosome, complete genome

```
product length = 202
Forward primer  1        TGCCTGGAACATAATCACCGT  21
Template        3099385  ............G........  3099405

Reverse primer  1        GTCGGTAGATAGCCTGTCGC  20
Template        3099586  ....................  3099567
```

>CP093018.1 Pseudomonas aeruginosa strain H11 chromosome

```
product length = 202
Forward primer  1        TGCCTGGAACATAATCACCGT  21
Template        5990333  ............G........  5990353

Reverse primer  1        GTCGGTAGATAGCCTGTCGC  20
Template        5990534  ....................  5990515
```

>CP093021.1 Pseudomonas aeruginosa strain H09 chromosome, complete genome

```
product length = 202
Forward primer  1        TGCCTGGAACATAATCACCGT  21
Template        3077690  ............G........  3077710

Reverse primer  1        GTCGGTAGATAGCCTGTCGC  20
Template        3077891  ....................  3077872
```

>CP093016.1 Pseudomonas aeruginosa strain H15 chromosome, complete genome

```
product length = 202
Forward primer  1        TGCCTGGAACATAATCACCGT  21
Template        5036869  ............G........  5036849

Reverse primer  1        GTCGGTAGATAGCCTGTCGC  20
Template        5036668  ....................  5036687
```

>CP093015.1 Pseudomonas aeruginosa strain H16 chromosome, complete genome

```
product length = 202
Forward primer  1        TGCCTGGAACATAATCACCGT  21
Template        3265534  ............G........  3265554

Reverse primer  1        GTCGGTAGATAGCCTGTCGC  20
Template        3265735  ....................  3265716
```

>CP093020.1 Pseudomonas aeruginosa strain H10 chromosome, complete genome

```
product length = 202
Forward primer  1        TGCCTGGAACATAATCACCGT  21
Template        3144908  ............G........  3144928

Reverse primer  1        GTCGGTAGATAGCCTGTCGC  20
Template        3145109  ....................  3145090
```

>CP093014.1 Pseudomonas aeruginosa strain H17 chromosome, complete genome

```
product length = 202
Forward primer  1        TGCCTGGAACATAATCACCGT  21
Template        3102797  ............G........  3102817

Reverse primer  1        GTCGGTAGATAGCCTGTCGC  20
Template        3102998  ....................  3102979
```

>CP080405.1 Pseudomonas aeruginosa strain PES_P749 chromosome, complete genome

```
product length = 202
Forward primer  1        TGCCTGGAACATAATCACCGT  21
Template        3140956  ............G........  3140976

Reverse primer  1        GTCGGTAGATAGCCTGTCGC  20
Template        3141157  ....................  3141138
```

>CP081148.1 Pseudomonas aeruginosa strain NDM1_2 chromosome

```
product length = 202
Forward primer  1        TGCCTGGAACATAATCACCGT  21
Template        3678017  ............G........  3677997

Reverse primer  1        GTCGGTAGATAGCCTGTCGC  20
Template        3677816  ....................  3677835
```

>CP092634.1 Pseudomonas aeruginosa strain LS.2c chromosome, complete genome

```
product length = 202
Forward primer  1        TGCCTGGAACATAATCACCGT  21
Template        3035499  ............G........  3035519

Reverse primer  1        GTCGGTAGATAGCCTGTCGC  20
Template        3035700  ....................  3035681
```

>CP092629.1 Pseudomonas aeruginosa strain HU20 chromosome, complete genome

```
product length = 202
Forward primer  1        TGCCTGGAACATAATCACCGT  21
Template        4704024  ............G........  4704004

Reverse primer  1        GTCGGTAGATAGCCTGTCGC  20
Template        4703823  ....................  4703842
```

>CP092032.1 Pseudomonas aeruginosa strain ZS-PA-05 chromosome, complete genome

```
product length = 202
Forward primer  1        TGCCTGGAACATAATCACCGT  21
Template        3648204  ............G........  3648224

Reverse primer  1        GTCGGTAGATAGCCTGTCGC  20
Template        3648405  ....................  3648386
```

>CP054794.1 Pseudomonas aeruginosa strain A0002 chromosome, complete genome

```
product length = 202
Forward primer  1        TGCCTGGAACATAATCACCGT  21
Template        3176085  ............G........  3176105

Reverse primer  1        GTCGGTAGATAGCCTGTCGC  20
Template        3176286  ....................  3176267
```

>CP054793.1 Pseudomonas aeruginosa strain SE5452 chromosome, complete genome

```
product length = 202
Forward primer  1        TGCCTGGAACATAATCACCGT  21
Template        3067686  ............G........  3067706

Reverse primer  1        GTCGGTAGATAGCCTGTCGC  20
Template        3067887  ....................  3067868
```

>CP054792.1 Pseudomonas aeruginosa strain SE5431 chromosome, complete genome

```
product length = 202
Forward primer  1        TGCCTGGAACATAATCACCGT  21
Template        3374267  ............G........  3374247

Reverse primer  1        GTCGGTAGATAGCCTGTCGC  20
Template        3374066  ....................  3374085
```

>CP054791.1 Pseudomonas aeruginosa strain SE5430 chromosome, complete genome

```
product length = 202
Forward primer  1        TGCCTGGAACATAATCACCGT  21
Template        3496991  ............G........  3496971

Reverse primer  1        GTCGGTAGATAGCCTGTCGC  20
Template        3496790  ....................  3496809
```

>CP054790.1 Pseudomonas aeruginosa strain SE5418 chromosome, complete genome

```
product length = 202
Forward primer  1        TGCCTGGAACATAATCACCGT  21
Template        3323745  ............G........  3323765

Reverse primer  1        GTCGGTAGATAGCCTGTCGC  20
Template        3323946  ....................  3323927
```

>CP054789.1 Pseudomonas aeruginosa strain SE5381 chromosome, complete genome

```
product length = 202
Forward primer  1        TGCCTGGAACATAATCACCGT  21
Template        3315590  ............G........  3315610

Reverse primer  1        GTCGGTAGATAGCCTGTCGC  20
Template        3315791  ....................  3315772
```

>CP054787.1 Pseudomonas aeruginosa strain HB2011305RE chromosome, complete genome

```
product length = 202
Forward primer  1        TGCCTGGAACATAATCACCGT  21
Template        3168202  ............G........  3168222

Reverse primer  1        GTCGGTAGATAGCCTGTCGC  20
Template        3168403  ....................  3168384
```

>CP054786.1 Pseudomonas aeruginosa strain DL201330 chromosome, complete genome

```
product length = 202
Forward primer  1        TGCCTGGAACATAATCACCGT  21
Template        1048094  ............G........  1048114

Reverse primer  1        GTCGGTAGATAGCCTGTCGC  20
Template        1048295  ....................  1048276
```

>CP081477.2 Pseudomonas aeruginosa strain P8W chromosome, complete genome

```
product length = 202
Forward primer  1        TGCCTGGAACATAATCACCGT  21
Template        1970273  ............G........  1970253

Reverse primer  1        GTCGGTAGATAGCCTGTCGC  20
Template        1970072  ....................  1970091
```

>CP090348.1 Pseudomonas aeruginosa strain PA8329 chromosome, complete genome

```
product length = 202
Forward primer  1        TGCCTGGAACATAATCACCGT  21
Template        3103878  ............G........  3103898

Reverse primer  1        GTCGGTAGATAGCCTGTCGC  20
Template        3104079  ....................  3104060
```

>CP053747.1 Pseudomonas aeruginosa strain Pae1255-NDM1 chromosome, complete genome

```
product length = 202
Forward primer  1        TGCCTGGAACATAATCACCGT  21
Template        3648690  ............G........  3648670

Reverse primer  1        GTCGGTAGATAGCCTGTCGC  20
Template        3648489  ....................  3648508
```

>CP089063.2 Pseudomonas aeruginosa strain UNC_PaerCF37 chromosome, complete genome

```
product length = 202
Forward primer  1        TGCCTGGAACATAATCACCGT  21
Template        2832225  ............G........  2832245

Reverse primer  1        GTCGGTAGATAGCCTGTCGC  20
Template        2832426  ....................  2832407
```

>CP089062.2 Pseudomonas aeruginosa strain UNC_PaerCF38 chromosome, complete genome

```
product length = 202
Forward primer  1        TGCCTGGAACATAATCACCGT  21
Template        4376736  ............G........  4376756

Reverse primer  1        GTCGGTAGATAGCCTGTCGC  20
Template        4376937  ....................  4376918
```

>CP089061.2 Pseudomonas aeruginosa strain UNC_PaerCF41 chromosome, complete genome

```
product length = 202
Forward primer  1        TGCCTGGAACATAATCACCGT  21
Template        6044279  ............G........  6044299

Reverse primer  1        GTCGGTAGATAGCCTGTCGC  20
Template        6044480  ....................  6044461
```

>CP089236.1 Pseudomonas aeruginosa strain JNQH-PA027 chromosome, complete genome

```
product length = 202
Forward primer  1        TGCCTGGAACATAATCACCGT  21
Template        2960418  ............G........  2960398

Reverse primer  1        GTCGGTAGATAGCCTGTCGC  20
Template        2960217  ....................  2960236
```

>CP087675.1 Pseudomonas aeruginosa strain P93127 chromosome, complete genome

```
product length = 202
Forward primer  1        TGCCTGGAACATAATCACCGT  21
Template        3349786  ............G........  3349806

Reverse primer  1        GTCGGTAGATAGCCTGTCGC  20
Template        3349987  ....................  3349968
```

>CP087674.1 Pseudomonas aeruginosa strain P4970C chromosome, complete genome

```
product length = 202
Forward primer  1        TGCCTGGAACATAATCACCGT  21
Template        3349786  ............G........  3349806

Reverse primer  1        GTCGGTAGATAGCCTGTCGC  20
Template        3349987  ....................  3349968
```

>CP087673.1 Pseudomonas aeruginosa strain P96131 chromosome, complete genome

```
product length = 202
Forward primer  1        TGCCTGGAACATAATCACCGT  21
Template        3253710  ............G........  3253730

Reverse primer  1        GTCGGTAGATAGCCTGTCGC  20
Template        3253911  ....................  3253892
```

>CP080369.1 Pseudomonas aeruginosa SG17M chromosome, complete genome

```
product length = 202
Forward primer  1        TGCCTGGAACATAATCACCGT  21
Template        3738768  ............G........  3738788

Reverse primer  1        GTCGGTAGATAGCCTGTCGC  20
Template        3738969  ....................  3738950
```

>CP086064.1 Pseudomonas aeruginosa strain CCBH28525 chromosome, complete genome

```
product length = 202
Forward primer  1        TGCCTGGAACATAATCACCGT  21
Template        3363391  ............G........  3363371

Reverse primer  1        GTCGGTAGATAGCCTGTCGC  20
Template        3363190  ....................  3363209
```

>CP086016.1 Pseudomonas aeruginosa isolate KB-PA_3 chromosome, complete genome

```
product length = 202
Forward primer  1        TGCCTGGAACATAATCACCGT  21
Template        3712371  ............G........  3712351

Reverse primer  1        GTCGGTAGATAGCCTGTCGC  20
Template        3712170  ....................  3712189
```

>CP086010.1 Pseudomonas aeruginosa isolate KB-PA_F19 chromosome, complete genome

```
product length = 202
Forward primer  1        TGCCTGGAACATAATCACCGT  21
Template        3231467  ............G........  3231487

Reverse primer  1        GTCGGTAGATAGCCTGTCGC  20
Template        3231668  ....................  3231649
```

>CP084484.1 Pseudomonas sp. PS1(2021) chromosome, complete genome

```
product length = 202
Forward primer  1        TGCCTGGAACATAATCACCGT  21
Template        1982250  ............G........  1982270

Reverse primer  1        GTCGGTAGATAGCCTGTCGC  20
Template        1982451  ....................  1982432
```

>CP083366.1 Pseudomonas aeruginosa strain PS1793 chromosome, complete genome

```
product length = 202
Forward primer  1        TGCCTGGAACATAATCACCGT  21
Template        3319531  ............G........  3319551

Reverse primer  1        GTCGGTAGATAGCCTGTCGC  20
Template        3319732  ....................  3319713
```

>CP082821.1 Pseudomonas aeruginosa strain SCAID PLC1-2021 (16/222) chromosome, complete genome

```
product length = 202
Forward primer  1        TGCCTGGAACATAATCACCGT  21
Template        3339257  ............G........  3339277

Reverse primer  1        GTCGGTAGATAGCCTGTCGC  20
Template        3339458  ....................  3339439
```

>CP082822.1 Pseudomonas aeruginosa strain SCAID WND1-2021 (9/195) chromosome, complete genome

```
product length = 202
Forward primer  1        TGCCTGGAACATAATCACCGT  21
Template        3388691  ............G........  3388711

Reverse primer  1        GTCGGTAGATAGCCTGTCGC  20
```

```
Template        3388892  ....................  3388873
```

>CP082823.1 Pseudomonas aeruginosa strain SCAID TST-2021 (7/157) chromosome, complete genome

```
product length = 202
Forward primer  1        TGCCTGGAACATAATCACCGT  21
Template        3307438  ............G........  3307458

Reverse primer  1        GTCGGTAGATAGCCTGTCGC  20
Template        3307639  ....................  3307620
```

>CP078009.1 Pseudomonas aeruginosa strain FAHZU31 chromosome, complete genome

```
product length = 202
Forward primer  1        TGCCTGGAACATAATCACCGT  21
Template        3195077  ............G........  3195097

Reverse primer  1        GTCGGTAGATAGCCTGTCGC  20
Template        3195278  ....................  3195259
```

>CP078007.1 Pseudomonas aeruginosa strain FAHZU40 chromosome, complete genome

```
product length = 202
Forward primer  1        TGCCTGGAACATAATCACCGT  21
Template        3108751  ............G........  3108771

Reverse primer  1        GTCGGTAGATAGCCTGTCGC  20
Template        3108952  ....................  3108933
```

>CP078006.1 Pseudomonas aeruginosa strain NDTH7329 chromosome, complete genome

```
product length = 202
Forward primer  1        TGCCTGGAACATAATCACCGT  21
Template        2947938  ............G........  2947958

Reverse primer  1        GTCGGTAGATAGCCTGTCGC  20
Template        2948139  ....................  2948120
```

>CP078004.1 Pseudomonas aeruginosa strain QZPH16 chromosome, complete genome

```
product length = 202
Forward primer  1        TGCCTGGAACATAATCACCGT  21
Template        3319233  ............G........  3319253

Reverse primer  1        GTCGGTAGATAGCCTGTCGC  20
Template        3319434  ....................  3319415
```

>CP078002.1 Pseudomonas aeruginosa strain QZPH21 chromosome, complete genome

```
product length = 202
Forward primer  1        TGCCTGGAACATAATCACCGT  21
Template        3319232  ............G........  3319252
```

```
Reverse primer  1        GTCGGTAGATAGCCTGTCGC  20
Template        3319433  ....................  3319414
```

>CP077999.1 Pseudomonas aeruginosa strain SRRSH1120 chromosome, complete genome

```
product length = 202
Forward primer  1        TGCCTGGAACATAATCACCGT  21
Template        3318352  ............G........  3318372

Reverse primer  1        GTCGGTAGATAGCCTGTCGC  20
Template        3318553  ....................  3318534
```

>CP077997.1 Pseudomonas aeruginosa strain SRRSH1521 chromosome, complete genome

```
product length = 202
Forward primer  1        TGCCTGGAACATAATCACCGT  21
Template        3157821  ............G........  3157841

Reverse primer  1        GTCGGTAGATAGCCTGTCGC  20
Template        3158022  ....................  3158003
```

>CP077994.1 Pseudomonas aeruginosa strain SRRSH2790 chromosome, complete genome

```
product length = 202
Forward primer  1        TGCCTGGAACATAATCACCGT  21
Template        3318599  ............G........  3318619

Reverse primer  1        GTCGGTAGATAGCCTGTCGC  20
Template        3318800  ....................  3318781
```

>CP077988.1 Pseudomonas aeruginosa strain ZPPH1 chromosome, complete genome

```
product length = 202
Forward primer  1        TGCCTGGAACATAATCACCGT  21
Template        3212512  ............G........  3212532

Reverse primer  1        GTCGGTAGATAGCCTGTCGC  20
Template        3212713  ....................  3212694
```

>CP077985.1 Pseudomonas aeruginosa strain ZPPH2 chromosome, complete genome

```
product length = 202
Forward primer  1        TGCCTGGAACATAATCACCGT  21
Template        3164978  ............G........  3164998

Reverse primer  1        GTCGGTAGATAGCCTGTCGC  20
Template        3165179  ....................  3165160
```

>CP077981.1 Pseudomonas aeruginosa strain ZPPH14 chromosome, complete genome

```
product length = 202
Forward primer  1        TGCCTGGAACATAATCACCGT  21
```

```
Template         3141620  ...........G........  3141640

Reverse primer  1        GTCGGTAGATAGCCTGTCGC  20
Template         3141821  ....................  3141802
```

>CP077977.1 Pseudomonas aeruginosa strain ZPPH29 chromosome, complete genome

```
product length = 202
Forward primer  1        TGCCTGGAACATAATCACCGT  21
Template         3288718  ...........G........  3288738

Reverse primer  1        GTCGGTAGATAGCCTGTCGC  20
Template         3288919  ....................  3288900
```

>CP077971.1 Pseudomonas aeruginosa strain ZPPH33 chromosome, complete genome

```
product length = 202
Forward primer  1        TGCCTGGAACATAATCACCGT  21
Template         3178359  ...........G........  3178379

Reverse primer  1        GTCGGTAGATAGCCTGTCGC  20
Template         3178560  ....................  3178541
```

>CP064401.1 Pseudomonas aeruginosa strain NDTH10366 chromosome, complete genome

```
product length = 202
Forward primer  1        TGCCTGGAACATAATCACCGT  21
Template         3513694  ...........G........  3513714

Reverse primer  1        GTCGGTAGATAGCCTGTCGC  20
Template         3513895  ....................  3513876
```

>CP064397.1 Pseudomonas aeruginosa strain SRRSH1002 chromosome, complete genome

```
product length = 202
Forward primer  1        TGCCTGGAACATAATCACCGT  21
Template         3283485  ...........G........  3283505

Reverse primer  1        GTCGGTAGATAGCCTGTCGC  20
Template         3283686  ....................  3283667
```

>CP064395.1 Pseudomonas aeruginosa strain SRRSH1408 chromosome, complete genome

```
product length = 202
Forward primer  1        TGCCTGGAACATAATCACCGT  21
Template         3281936  ...........G........  3281956

Reverse primer  1        GTCGGTAGATAGCCTGTCGC  20
Template         3282137  ....................  3282118
```

>CP064393.1 Pseudomonas aeruginosa strain SRRSH1101 chromosome, complete genome

```
product length = 202
```

```
Forward primer  1        TGCCTGGAACATAATCACCGT  21
Template        3281948  ............G........  3281968

Reverse primer  1        GTCGGTAGATAGCCTGTCGC  20
Template        3282149  ....................  3282130
```

>CP064392.1 Pseudomonas aeruginosa strain SRRSH15 chromosome, complete genome

```
product length = 202
Forward primer  1        TGCCTGGAACATAATCACCGT  21
Template        3159572  ............G........  3159592

Reverse primer  1        GTCGGTAGATAGCCTGTCGC  20
Template        3159773  ....................  3159754
```

>CP080518.1 Pseudomonas aeruginosa strain YY322 chromosome, complete genome

```
product length = 202
Forward primer  1        TGCCTGGAACATAATCACCGT  21
Template        3132450  ............G........  3132470

Reverse primer  1        GTCGGTAGATAGCCTGTCGC  20
Template        3132651  ....................  3132632
```

>CP080511.1 Pseudomonas aeruginosa strain DJ06 chromosome, complete genome

```
product length = 202
Forward primer  1        TGCCTGGAACATAATCACCGT  21
Template        3921385  ............G........  3921365

Reverse primer  1        GTCGGTAGATAGCCTGTCGC  20
Template        3921184  ....................  3921203
```

>CP080289.1 Pseudomonas aeruginosa strain PA2207 chromosome, complete genome

```
product length = 202
Forward primer  1        TGCCTGGAACATAATCACCGT  21
Template        3387963  ............G........  3387943

Reverse primer  1        GTCGGTAGATAGCCTGTCGC  20
Template        3387762  ....................  3387781
```

>CP080288.1 Pseudomonas aeruginosa strain UNC_PaerCF05 chromosome, complete genome

```
product length = 202
Forward primer  1        TGCCTGGAACATAATCACCGT  21
Template        3856854  ............G........  3856834

Reverse primer  1        GTCGGTAGATAGCCTGTCGC  20
Template        3856653  ....................  3856672
```

>CP080282.1 Pseudomonas aeruginosa strain UNC_PaerCF16 chromosome, complete genome

```
product length = 202
Forward primer  1        TGCCTGGAACATAATCACCGT  21
Template        4620908  ............G........  4620928

Reverse primer  1        GTCGGTAGATAGCCTGTCGC  20
Template        4621109  ....................  4621090
```

>CP080285.1 Pseudomonas aeruginosa strain UNC_PaerCF14 chromosome, complete genome

```
product length = 203
Forward primer  1        TGCCTGGAACATAATCACCGT  21
Template        3995938  ............G........  3995958

Reverse primer  1        GTCGGTAGATAGCCTGTCGC  20
Template        3996140  ....................  3996121
```

>CP080280.1 Pseudomonas aeruginosa strain UNC_PaerCF20 chromosome, complete genome

```
product length = 202
Forward primer  1        TGCCTGGAACATAATCACCGT  21
Template        3281097  ............G........  3281077

Reverse primer  1        GTCGGTAGATAGCCTGTCGC  20
Template        3280896  ....................  3280915
```

>CP080281.1 Pseudomonas aeruginosa strain UNC_PaerCF17 chromosome, complete genome

```
product length = 202
Forward primer  1        TGCCTGGAACATAATCACCGT  21
Template        4598386  ............G........  4598406

Reverse primer  1        GTCGGTAGATAGCCTGTCGC  20
Template        4598587  ....................  4598568
```

>CP080007.1 Pseudomonas aeruginosa strain S-1 chromosome, complete genome

```
product length = 202
Forward primer  1        TGCCTGGAACATAATCACCGT  21
Template        3125461  ............G........  3125481

Reverse primer  1        GTCGGTAGATAGCCTGTCGC  20
Template        3125662  ....................  3125643
```

>CP061376.1 Pseudomonas aeruginosa strain HS17-127 chromosome, complete genome

```
product length = 202
Forward primer  1        TGCCTGGAACATAATCACCGT  21
Template        3136627  ............G........  3136647

Reverse primer  1        GTCGGTAGATAGCCTGTCGC  20
Template        3136828  ....................  3136809
```

>CP078564.1 Pseudomonas aeruginosa strain Colony464 chromosome

```
product length = 202
Forward primer  1        TGCCTGGAACATAATCACCGT  21
Template        1461953  ............G........  1461933

Reverse primer  1        GTCGGTAGATAGCCTGTCGC  20
Template        1461752  ....................  1461771
```

>CP053390.1 Pseudomonas aeruginosa strain TL1285 chromosome, complete genome

```
product length = 202
Forward primer  1        TGCCTGGAACATAATCACCGT  21
Template        5937550  ............G........  5937530

Reverse primer  1        GTCGGTAGATAGCCTGTCGC  20
Template        5937349  ....................  5937368
```

>CP075176.1 Pseudomonas aeruginosa strain PA790 chromosome, complete genome

```
product length = 202
Forward primer  1        TGCCTGGAACATAATCACCGT  21
Template        3235269  ............G........  3235289

Reverse primer  1        GTCGGTAGATAGCCTGTCGC  20
Template        3235470  ....................  3235451
```

>CP065948.1 Pseudomonas aeruginosa strain PAM68 chromosome, complete genome

```
product length = 202
Forward primer  1        TGCCTGGAACATAATCACCGT  21
Template        1807373  ............G........  1807393

Reverse primer  1        GTCGGTAGATAGCCTGTCGC  20
Template        1807574  ....................  1807555
```

>CP065947.1 Pseudomonas aeruginosa strain PAS6 chromosome, complete genome

```
product length = 202
Forward primer  1        TGCCTGGAACATAATCACCGT  21
Template        5591478  ............G........  5591458

Reverse primer  1        GTCGGTAGATAGCCTGTCGC  20
Template        5591277  ....................  5591296
```

>CP065374.1 Pseudomonas aeruginosa strain PAG7 chromosome, complete genome

```
product length = 202
Forward primer  1      TGCCTGGAACATAATCACCGT  21
Template        64679  ............G........  64699

Reverse primer  1      GTCGGTAGATAGCCTGTCGC  20
Template        64880  ....................  64861
```

>CP073080.1 Pseudomonas aeruginosa strain NDTH9845 chromosome, complete genome

```
product length = 202
Forward primer  1        TGCCTGGAACATAATCACCGT  21
Template        3640272  ............G........  3640292

Reverse primer  1        GTCGGTAGATAGCCTGTCGC  20
Template        3640473  ....................  3640454
```

>CP073082.1 Pseudomonas aeruginosa strain WTJH17 chromosome, complete genome

```
product length = 202
Forward primer  1        TGCCTGGAACATAATCACCGT  21
Template        3150633  ............G........  3150653

Reverse primer  1        GTCGGTAGATAGCCTGTCGC  20
Template        3150834  ....................  3150815
```

>CP072783.1 Pseudomonas aeruginosa strain LICME WGH-6 chromosome, complete genome

```
product length = 202
Forward primer  1        TGCCTGGAACATAATCACCGT  21
Template        3223166  ............G........  3223146

Reverse primer  1        GTCGGTAGATAGCCTGTCGC  20
Template        3222965  ....................  3222984
```

>CP071731.1 Pseudomonas aeruginosa strain LYSZa2 chromosome, complete genome

```
product length = 202
Forward primer  1        TGCCTGGAACATAATCACCGT  21
Template        5811473  ............G........  5811493

Reverse primer  1        GTCGGTAGATAGCCTGTCGC  20
Template        5811674  ....................  5811655
```

>CP071730.1 Pseudomonas aeruginosa strain LYSZa5 chromosome, complete genome

```
product length = 202
Forward primer  1        TGCCTGGAACATAATCACCGT  21
Template        5811483  ............G........  5811503

Reverse primer  1        GTCGGTAGATAGCCTGTCGC  20
Template        5811684  ....................  5811665
```

>AP024513.1 Pseudomonas aeruginosa Pa12 DNA, complete genome

```
product length = 202
Forward primer  1        TGCCTGGAACATAATCACCGT  21
Template        3149218  ............G........  3149238

Reverse primer  1        GTCGGTAGATAGCCTGTCGC  20
Template        3149419  ....................  3149400
```

>CP024024.1 Pseudomonas aeruginosa strain PARM801 chromosome, complete genome

```
product length = 202
Forward primer  1        TGCCTGGAACATAATCACCGT  21
Template        1228250  ............G........  1228270

Reverse primer  1        GTCGGTAGATAGCCTGTCGC  20
Template        1228451  ....................  1228432
```

>CP069198.1 Pseudomonas aeruginosa strain 152962 chromosome, complete genome

```
product length = 202
Forward primer  1        TGCCTGGAACATAATCACCGT  21
Template        2017853  ............G........  2017833

Reverse primer  1        GTCGGTAGATAGCCTGTCGC  20
Template        2017652  ....................  2017671
```

>CP060392.1 Pseudomonas aeruginosa strain 1903031130 chromosome, complete genome

```
product length = 202
Forward primer  1        TGCCTGGAACATAATCACCGT  21
Template        3700221  ............G........  3700201

Reverse primer  1        GTCGGTAGATAGCCTGTCGC  20
Template        3700020  ....................  3700039
```

>CP054845.1 Pseudomonas aeruginosa strain SE5429 chromosome, complete genome

```
product length = 202
Forward primer  1        TGCCTGGAACATAATCACCGT  21
Template        3557213  ............G........  3557233

Reverse primer  1        GTCGGTAGATAGCCTGTCGC  20
Template        3557414  ....................  3557395
```

>CP054843.1 Pseudomonas aeruginosa strain SE5352 chromosome, complete genome

```
product length = 202
Forward primer  1        TGCCTGGAACATAATCACCGT  21
Template        3700618  ............G........  3700598

Reverse primer  1        GTCGGTAGATAGCCTGTCGC  20
Template        3700417  ....................  3700436
```

>CP054581.1 Pseudomonas aeruginosa strain YTSEY8 chromosome, complete genome

```
product length = 202
Forward primer  1        TGCCTGGAACATAATCACCGT  21
Template        3483496  ............G........  3483516

Reverse primer  1        GTCGGTAGATAGCCTGTCGC  20
Template        3483697  ....................  3483678
```

>CP068239.1 Pseudomonas aeruginosa strain PA19-3047 chromosome, complete genome

```
product length = 202
Forward primer  1        TGCCTGGAACATAATCACCGT  21
Template        3297046  ............G........  3297066

Reverse primer  1        GTCGGTAGATAGCCTGTCGC  20
Template        3297247  ....................  3297228
```

>CP061699.1 Pseudomonas aeruginosa strain LYSZa7 chromosome, complete genome

```
product length = 202
Forward primer  1        TGCCTGGAACATAATCACCGT  21
Template        3681327  ............G........  3681307

Reverse primer  1        GTCGGTAGATAGCCTGTCGC  20
Template        3681126  ....................  3681145
```

>CP054623.1 Pseudomonas aeruginosa strain DL201330 chromosome, complete genome

```
product length = 202
Forward primer  1        TGCCTGGAACATAATCACCGT  21
Template        3132435  ............G........  3132455

Reverse primer  1        GTCGGTAGATAGCCTGTCGC  20
Template        3132636  ....................  3132617
```

>CP065966.1 Pseudomonas aeruginosa strain FDAARGOS_1041 chromosome, complete genome

```
product length = 202
Forward primer  1        TGCCTGGAACATAATCACCGT  21
Template        6796275  ............G........  6796295

Reverse primer  1        GTCGGTAGATAGCCTGTCGC  20
Template        6796476  ....................  6796457
```

>CP065867.1 Pseudomonas aeruginosa strain TJ2014-049 chromosome, complete genome

```
product length = 202
Forward primer  1        TGCCTGGAACATAATCACCGT  21
Template        3175388  ............G........  3175408

Reverse primer  1        GTCGGTAGATAGCCTGTCGC  20
Template        3175589  ....................  3175570
```

>CP065865.1 Pseudomonas aeruginosa strain TJ2019-022 chromosome, complete genome

```
product length = 202
Forward primer  1        TGCCTGGAACATAATCACCGT  21
Template        3575723  ............G........  3575703

Reverse primer  1        GTCGGTAGATAGCCTGTCGC  20
Template        3575522  ....................  3575541
```

>CP065866.1 Pseudomonas aeruginosa strain TJ2019-017 chromosome, complete genome

```
product length = 202
Forward primer  1        TGCCTGGAACATAATCACCGT  21
Template        3133276  ............G........  3133296

Reverse primer  1        GTCGGTAGATAGCCTGTCGC  20
Template        3133477  ....................  3133458
```

>CP065848.1 Pseudomonas aeruginosa strain CMC-097 chromosome, complete genome

```
product length = 202
Forward primer  1        TGCCTGGAACATAATCACCGT  21
Template        4212821  ............G........  4212801

Reverse primer  1        GTCGGTAGATAGCCTGTCGC  20
Template        4212620  ....................  4212639
```

>CP065417.1 Pseudomonas aeruginosa isolate P23 chromosome, complete genome

```
product length = 202
Forward primer  1        TGCCTGGAACATAATCACCGT  21
Template        3282614  ............G........  3282634

Reverse primer  1        GTCGGTAGATAGCCTGTCGC  20
Template        3282815  ....................  3282796
```

>CP046402.2 Pseudomonas aeruginosa strain SE5331 chromosome, complete genome

```
product length = 202
Forward primer  1        TGCCTGGAACATAATCACCGT  21
Template        3423844  ............G........  3423864

Reverse primer  1        GTCGGTAGATAGCCTGTCGC  20
Template        3424045  ....................  3424026
```

>CP046406.2 Pseudomonas aeruginosa strain SE5458 chromosome, complete genome

```
product length = 202
Forward primer  1        TGCCTGGAACATAATCACCGT  21
Template        3545283  ............G........  3545263

Reverse primer  1        GTCGGTAGATAGCCTGTCGC  20
Template        3545082  ....................  3545101
```

>CP045552.2 Pseudomonas aeruginosa strain YT12746 chromosome, complete genome

```
product length = 202
Forward primer  1        TGCCTGGAACATAATCACCGT  21
Template        3304211  ............G........  3304231

Reverse primer  1        GTCGGTAGATAGCCTGTCGC  20
Template        3304412  ....................  3304393
```

>CP060243.1 Pseudomonas aeruginosa strain A-I-1 chromosome, complete genome

```
product length = 202
Forward primer  1        TGCCTGGAACATAATCACCGT  21
Template        3436380  ............G........  3436400

Reverse primer  1        GTCGGTAGATAGCCTGTCGC  20
Template        3436581  ....................  3436562
```

>CP060242.1 Pseudomonas aeruginosa strain B-I-1 chromosome, complete genome

```
product length = 202
Forward primer  1        TGCCTGGAACATAATCACCGT  21
Template        3791146  ............G........  3791166

Reverse primer  1        GTCGGTAGATAGCCTGTCGC  20
Template        3791347  ....................  3791328
```

>CP060241.1 Pseudomonas aeruginosa strain C-I-1 chromosome, complete genome

```
product length = 202
Forward primer  1        TGCCTGGAACATAATCACCGT  21
Template        3233295  ............G........  3233275

Reverse primer  1        GTCGGTAGATAGCCTGTCGC  20
Template        3233094  ....................  3233113
```

>CP060240.1 Pseudomonas aeruginosa strain G-I-1 chromosome, complete genome

```
product length = 202
Forward primer  1        TGCCTGGAACATAATCACCGT  21
Template        3222579  ............G........  3222599

Reverse primer  1        GTCGGTAGATAGCCTGTCGC  20
Template        3222780  ....................  3222761
```

>LR898867.1 Pseudomonas aeruginosa isolate MINF_3A-sc-2280432 genome assembly, chromosome: 1

```
product length = 202
Forward primer  1        TGCCTGGAACATAATCACCGT  21
Template        3267763  ............G........  3267783

Reverse primer  1        GTCGGTAGATAGCCTGTCGC  20
Template        3267964  ....................  3267945
```

>LR890619.1 Pseudomonas aeruginosa isolate MINF_7A-sc-2280434 genome assembly, chromosome: 1

```
product length = 202
Forward primer  1        TGCCTGGAACATAATCACCGT  21
Template        3026862  ............G........  3026882

Reverse primer  1        GTCGGTAGATAGCCTGTCGC  20
```

```
Template        3027063  ....................  3027044
```

>CP061034.1 Pseudomonas aeruginosa strain PA3 chromosome, complete genome

```
product length = 202
Forward primer  1        TGCCTGGAACATAATCACCGT  21
Template        3217135  ............G........  3217155

Reverse primer  1        GTCGGTAGATAGCCTGTCGC  20
Template        3217336  ....................  3217317
```

>CP060703.1 Pseudomonas aeruginosa strain NRD619 chromosome, complete genome

```
product length = 202
Forward primer  1        TGCCTGGAACATAATCACCGT  21
Template        3070711  ............G........  3070731

Reverse primer  1        GTCGGTAGATAGCCTGTCGC  20
Template        3070912  ....................  3070893
```

>CP060086.1 Pseudomonas aeruginosa strain JNQH-PA57 chromosome, complete genome

```
product length = 202
Forward primer  1        TGCCTGGAACATAATCACCGT  21
Template        3192303  ............G........  3192323

Reverse primer  1        GTCGGTAGATAGCCTGTCGC  20
Template        3192504  ....................  3192485
```

>CP059995.1 Pseudomonas aeruginosa strain NY3045 chromosome, complete genome

```
product length = 202
Forward primer  1        TGCCTGGAACATAATCACCGT  21
Template        3340109  ............G........  3340129

Reverse primer  1        GTCGGTAGATAGCCTGTCGC  20
Template        3340310  ....................  3340291
```

>CP059063.1 Pseudomonas aeruginosa strain GIMC5034:PA52Ts32 chromosome

```
product length = 202
Forward primer  1        TGCCTGGAACATAATCACCGT  21
Template        3617216  ............G........  3617196

Reverse primer  1        GTCGGTAGATAGCCTGTCGC  20
Template        3617015  ....................  3617034
```

>CP058331.1 Pseudomonas aeruginosa strain ACR22 chromosome, complete genome

```
product length = 202
Forward primer  1        TGCCTGGAACATAATCACCGT  21
Template        3747092  ............G........  3747112
```

```
Reverse primer  1        GTCGGTAGATAGCCTGTCGC  20
Template        3747293  ....................  3747274
```

>CP058333.1 Pseudomonas aeruginosa strain ACR20 chromosome, complete genome

```
product length = 202
Forward primer  1        TGCCTGGAACATAATCACCGT  21
Template        2462313  ............G........  2462293

Reverse primer  1        GTCGGTAGATAGCCTGTCGC  20
Template        2462112  ....................  2462131
```

>CP053117.1 Pseudomonas aeruginosa strain P16CT chromosome

```
product length = 202
Forward primer  1        TGCCTGGAACATAATCACCGT  21
Template        2453970  ............G........  2453950

Reverse primer  1        GTCGGTAGATAGCCTGTCGC  20
Template        2453769  ....................  2453788
```

>CP058332.1 Pseudomonas aeruginosa strain B18 chromosome, complete genome

```
product length = 202
Forward primer  1        TGCCTGGAACATAATCACCGT  21
Template        5016996  ............G........  5016976

Reverse primer  1        GTCGGTAGATAGCCTGTCGC  20
Template        5016795  ....................  5016814
```

>CP058323.1 Pseudomonas aeruginosa strain LV chromosome

```
product length = 202
Forward primer  1        TGCCTGGAACATAATCACCGT  21
Template        4164099  ............G........  4164079

Reverse primer  1        GTCGGTAGATAGCCTGTCGC  20
Template        4163898  ....................  4163917
```

>CP046405.1 Pseudomonas aeruginosa strain SE5443 chromosome, complete genome

```
product length = 202
Forward primer  1        TGCCTGGAACATAATCACCGT  21
Template        3043207  ............G........  3043227

Reverse primer  1        GTCGGTAGATAGCCTGTCGC  20
Template        3043408  ....................  3043389
```

>CP046404.1 Pseudomonas aeruginosa strain SE5416 chromosome, complete genome

```
product length = 202
Forward primer  1        TGCCTGGAACATAATCACCGT  21
Template        3756514  ............G........  3756534
```

```
Reverse primer  1        GTCGGTAGATAGCCTGTCGC  20
Template        3756715  ....................  3756696
```

>CP046403.1 Pseudomonas aeruginosa strain SE5369 chromosome, complete genome

```
product length = 202
Forward primer  1        TGCCTGGAACATAATCACCGT  21
Template        3538782  ............G........  3538802

Reverse primer  1        GTCGGTAGATAGCCTGTCGC  20
Template        3538983  ....................  3538964
```

>CP056100.1 Pseudomonas aeruginosa strain PABCH01 chromosome

```
product length = 202
Forward primer  1        TGCCTGGAACATAATCACCGT  21
Template        3357002  ............G........  3357022

Reverse primer  1        GTCGGTAGATAGCCTGTCGC  20
Template        3357203  ....................  3357184
```

>CP056090.1 Pseudomonas aeruginosa strain PABCH42 chromosome

```
product length = 202
Forward primer  1        TGCCTGGAACATAATCACCGT  21
Template        4986280  ............G........  4986260

Reverse primer  1        GTCGGTAGATAGCCTGTCGC  20
Template        4986079  ....................  4986098
```

>CP056089.1 Pseudomonas aeruginosa strain PABCH46 chromosome

```
product length = 202
Forward primer  1        TGCCTGGAACATAATCACCGT  21
Template        3677679  ............G........  3677659

Reverse primer  1        GTCGGTAGATAGCCTGTCGC  20
Template        3677478  ....................  3677497
```

>CP056095.1 Pseudomonas aeruginosa strain PABCH09 chromosome

```
product length = 202
Forward primer  1        TGCCTGGAACATAATCACCGT  21
Template        3169078  ............G........  3169098

Reverse primer  1        GTCGGTAGATAGCCTGTCGC  20
Template        3169279  ....................  3169260
```

>CP056092.1 Pseudomonas aeruginosa strain PABCH14 chromosome

```
product length = 202
Forward primer  1        TGCCTGGAACATAATCACCGT  21
```

```
Template        3565066  ............G........  3565086

Reverse primer  1        GTCGGTAGATAGCCTGTCGC  20
Template        3565267  ....................  3565248
```

>CP056101.1 Pseudomonas aeruginosa strain PABCH45 chromosome

```
product length = 202
Forward primer  1        TGCCTGGAACATAATCACCGT  21
Template        2644963  ............G........  2644943

Reverse primer  1        GTCGGTAGATAGCCTGTCGC  20
Template        2644762  ....................  2644781
```

>CP056098.1 Pseudomonas aeruginosa strain PABCH05 chromosome

```
product length = 202
Forward primer  1        TGCCTGGAACATAATCACCGT  21
Template        3028204  ............G........  3028224

Reverse primer  1        GTCGGTAGATAGCCTGTCGC  20
Template        3028405  ....................  3028386
```

>CP056094.1 Pseudomonas aeruginosa strain PABCH10 chromosome

```
product length = 202
Forward primer  1        TGCCTGGAACATAATCACCGT  21
Template        3567653  ............G........  3567673

Reverse primer  1        GTCGGTAGATAGCCTGTCGC  20
Template        3567854  ....................  3567835
```

>CP056093.1 Pseudomonas aeruginosa strain PABCH13 chromosome

```
product length = 202
Forward primer  1        TGCCTGGAACATAATCACCGT  21
Template        3129230  ............G........  3129250

Reverse primer  1        GTCGGTAGATAGCCTGTCGC  20
Template        3129431  ....................  3129412
```

>CP050334.1 Pseudomonas aeruginosa strain DVT410 chromosome, complete genome

```
product length = 202
Forward primer  1        TGCCTGGAACATAATCACCGT  21
Template        3012269  ............G........  3012289

Reverse primer  1        GTCGGTAGATAGCCTGTCGC  20
Template        3012470  ....................  3012451
```

>CP050333.1 Pseudomonas aeruginosa strain DVT412 chromosome, complete genome

```
product length = 202
```

```
Forward primer  1        TGCCTGGAACATAATCACCGT  21
Template        2477389  ............G........  2477369

Reverse primer  1        GTCGGTAGATAGCCTGTCGC  20
Template        2477188  ....................  2477207
```

>CP050332.1 Pseudomonas aeruginosa strain DVT413 chromosome, complete genome

```
product length = 202
Forward primer  1        TGCCTGGAACATAATCACCGT  21
Template        3428165  ............G........  3428185

Reverse primer  1        GTCGGTAGATAGCCTGTCGC  20
Template        3428366  ....................  3428347
```

>CP050331.1 Pseudomonas aeruginosa strain DVT414 chromosome, complete genome

```
product length = 202
Forward primer  1        TGCCTGGAACATAATCACCGT  21
Template        3144140  ............G........  3144160

Reverse primer  1        GTCGGTAGATAGCCTGTCGC  20
Template        3144341  ....................  3144322
```

>CP050330.1 Pseudomonas aeruginosa strain DVT779 chromosome, complete genome

```
product length = 202
Forward primer  1        TGCCTGGAACATAATCACCGT  21
Template        3130567  ............G........  3130587

Reverse primer  1        GTCGGTAGATAGCCTGTCGC  20
Template        3130768  ....................  3130749
```

>CP050329.1 Pseudomonas aeruginosa strain DVT417 chromosome, complete genome

```
product length = 202
Forward primer  1        TGCCTGGAACATAATCACCGT  21
Template        3103065  ............G........  3103085

Reverse primer  1        GTCGGTAGATAGCCTGTCGC  20
Template        3103266  ....................  3103247
```

>CP050326.1 Pseudomonas aeruginosa strain DVT423 chromosome, complete genome

```
product length = 202
Forward primer  1        TGCCTGGAACATAATCACCGT  21
Template        3928651  ............G........  3928631

Reverse primer  1        GTCGGTAGATAGCCTGTCGC  20
Template        3928450  ....................  3928469
```

>CP050325.1 Pseudomonas aeruginosa strain DVT425 chromosome, complete genome

```
product length = 202
Forward primer  1        TGCCTGGAACATAATCACCGT  21
Template        2698376  ............G........  2698396

Reverse primer  1        GTCGGTAGATAGCCTGTCGC  20
Template        2698577  ....................  2698558
```

>CP050324.1 Pseudomonas aeruginosa strain DVT427 chromosome, complete genome

```
product length = 202
Forward primer  1        TGCCTGGAACATAATCACCGT  21
Template        3158398  ............G........  3158418

Reverse primer  1        GTCGGTAGATAGCCTGTCGC  20
Template        3158599  ....................  3158580
```

>CP050323.1 Pseudomonas aeruginosa strain DVT429 chromosome, complete genome

```
product length = 202
Forward primer  1        TGCCTGGAACATAATCACCGT  21
Template        3924816  ............G........  3924796

Reverse primer  1        GTCGGTAGATAGCCTGTCGC  20
Template        3924615  ....................  3924634
```

>CP050322.1 Pseudomonas aeruginosa strain DVT729 chromosome, complete genome

```
product length = 202
Forward primer  1        TGCCTGGAACATAATCACCGT  21
Template        3351263  ............G........  3351283

Reverse primer  1        GTCGGTAGATAGCCTGTCGC  20
Template        3351464  ....................  3351445
```

>CP054572.1 Pseudomonas sp. FDAARGOS_761 chromosome, complete genome

```
product length = 202
Forward primer  1        TGCCTGGAACATAATCACCGT  21
Template        2487671  ............G........  2487651

Reverse primer  1        GTCGGTAGATAGCCTGTCGC  20
Template        2487470  ....................  2487489
```

>CP054473.1 Pseudomonas aeruginosa strain PAAK095 chromosome, complete genome

```
product length = 202
Forward primer  1        TGCCTGGAACATAATCACCGT  21
Template        3938041  ............G........  3938021

Reverse primer  1        GTCGGTAGATAGCCTGTCGC  20
Template        3937840  ....................  3937859
```

>CP054472.1 Pseudomonas aeruginosa strain PAAK088 chromosome, complete genome

```
product length = 202
Forward primer  1        TGCCTGGAACATAATCACCGT  21
Template        3172599  ............G........  3172619

Reverse primer  1        GTCGGTAGATAGCCTGTCGC  20
Template        3172800  ....................  3172781
```

>CP053922.1 Pseudomonas aeruginosa strain YD001 chromosome, complete genome

```
product length = 202
Forward primer  1        TGCCTGGAACATAATCACCGT  21
Template        3177060  ............G........  3177080

Reverse primer  1        GTCGGTAGATAGCCTGTCGC  20
Template        3177261  ....................  3177242
```

>CP053917.1 Pseudomonas aeruginosa strain PSE6684 chromosome, complete genome

```
product length = 202
Forward primer  1        TGCCTGGAACATAATCACCGT  21
Template        6431313  ............G........  6431293

Reverse primer  1        GTCGGTAGATAGCCTGTCGC  20
Template        6431112  ....................  6431131
```

>CP053706.1 Pseudomonas aeruginosa strain PAC1 chromosome, complete genome

```
product length = 202
Forward primer  1        TGCCTGGAACATAATCACCGT  21
Template        1362334  ............G........  1362314

Reverse primer  1        GTCGGTAGATAGCCTGTCGC  20
Template        1362133  ....................  1362152
```

>CP053705.1 Pseudomonas aeruginosa strain PAC6 chromosome, complete genome

```
product length = 202
Forward primer  1        TGCCTGGAACATAATCACCGT  21
Template        3179932  ............G........  3179952

Reverse primer  1        GTCGGTAGATAGCCTGTCGC  20
Template        3180133  ....................  3180114
```

>CP053687.1 Pseudomonas aeruginosa strain K19PSE24 chromosome

```
product length = 202
Forward primer  1        TGCCTGGAACATAATCACCGT  21
Template        2510538  ............G........  2510558

Reverse primer  1        GTCGGTAGATAGCCTGTCGC  20
Template        2510739  ....................  2510720
```

>CP053686.1 Pseudomonas aeruginosa strain SCAID PHRX1-2019 chromosome

```
product length = 202
Forward primer  1        TGCCTGGAACATAATCACCGT  21
Template        6324176  ............G........  6324196

Reverse primer  1        GTCGGTAGATAGCCTGTCGC  20
Template        6324377  ....................  6324358
```

>CP044533.1 Pseudomonas aeruginosa strain Ps33 chromosome

```
product length = 202
Forward primer  1        TGCCTGGAACATAATCACCGT  21
Template        2567866  ............G........  2567886

Reverse primer  1        GTCGGTAGATAGCCTGTCGC  20
Template        2568067  ....................  2568048
```

>CP051770.1 Pseudomonas aeruginosa strain GIMC5021:PA52Ts17, complete sequence

```
product length = 202
Forward primer  1        TGCCTGGAACATAATCACCGT  21
Template        3613030  ............G........  3613010

Reverse primer  1        GTCGGTAGATAGCCTGTCGC  20
Template        3612829  ....................  3612848
```

>CP051768.1 Pseudomonas aeruginosa strain GIMC5020:PA52Ts2, complete sequence

```
product length = 202
Forward primer  1        TGCCTGGAACATAATCACCGT  21
Template        3614816  ............G........  3614796

Reverse primer  1        GTCGGTAGATAGCCTGTCGC  20
Template        3614615  ....................  3614634
```

>CP051766.1 Pseudomonas aeruginosa strain GIMC5019:PA52Ts1, complete sequence

```
product length = 202
Forward primer  1        TGCCTGGAACATAATCACCGT  21
Template        3614831  ............G........  3614811

Reverse primer  1        GTCGGTAGATAGCCTGTCGC  20
Template        3614630  ....................  3614649
```

>CP051547.1 Pseudomonas aeruginosa strain AA2 chromosome, complete genome

```
product length = 202
Forward primer  1        TGCCTGGAACATAATCACCGT  21
Template        3058019  ............G........  3058039

Reverse primer  1        GTCGGTAGATAGCCTGTCGC  20
Template        3058220  ....................  3058201
```

>CP046602.1 Pseudomonas aeruginosa strain CMC-115 chromosome, complete genome

```
product length = 202
Forward primer  1        TGCCTGGAACATAATCACCGT  21
Template        2491172  ............G........  2491152

Reverse primer  1        GTCGGTAGATAGCCTGTCGC  20
Template        2490971  ....................  2490990
```

>CP045916.1 Pseudomonas aeruginosa strain CF39S chromosome, complete genome

```
product length = 202
Forward primer  1        TGCCTGGAACATAATCACCGT  21
Template        3321962  ............G........  3321982

Reverse primer  1        GTCGGTAGATAGCCTGTCGC  20
Template        3322163  ....................  3322144
```

>CP045002.1 Pseudomonas aeruginosa strain PAG5 chromosome, complete genome

```
product length = 202
Forward primer  1        TGCCTGGAACATAATCACCGT  21
Template        3213495  ............G........  3213515

Reverse primer  1        GTCGGTAGATAGCCTGTCGC  20
Template        3213696  ....................  3213677
```

>CP021380.2 Pseudomonas aeruginosa strain CCBH4851 genome

```
product length = 202
Forward primer  1        TGCCTGGAACATAATCACCGT  21
Template        3369112  ............G........  3369132

Reverse primer  1        GTCGGTAGATAGCCTGTCGC  20
Template        3369313  ....................  3369294
```

>CP049161.1 Pseudomonas aeruginosa strain MS14403 chromosome, complete genome

```
product length = 202
Forward primer  1        TGCCTGGAACATAATCACCGT  21
Template        3033816  ............G........  3033836

Reverse primer  1        GTCGGTAGATAGCCTGTCGC  20
Template        3034017  ....................  3033998
```

>CP048791.1 Pseudomonas aeruginosa strain VIT PC9 chromosome, complete genome

```
product length = 202
Forward primer  1        TGCCTGGAACATAATCACCGT  21
Template        3967256  ............G........  3967236

Reverse primer  1        GTCGGTAGATAGCCTGTCGC  20
Template        3967055  ....................  3967074
```

>LR739069.1 Pseudomonas aeruginosa strain PcyII-40 genome assembly, chromosome: PcyII-40

```
product length = 202
Forward primer  1        TGCCTGGAACATAATCACCGT  21
Template        3301408  ............G........  3301428

Reverse primer  1        GTCGGTAGATAGCCTGTCGC  20
Template        3301609  ....................  3301590
```

>LR739068.1 Pseudomonas aeruginosa strain PcyII-29 genome assembly, chromosome: PcyII-29

```
product length = 202
Forward primer  1        TGCCTGGAACATAATCACCGT  21
Template        3168869  ............G........  3168889

Reverse primer  1        GTCGGTAGATAGCCTGTCGC  20
Template        3169070  ....................  3169051
```

>CP047697.1 Pseudomonas aeruginosa strain RD1-3 chromosome, complete genome

```
product length = 202
Forward primer  1        TGCCTGGAACATAATCACCGT  21
Template        3736253  ............G........  3736233

Reverse primer  1        GTCGGTAGATAGCCTGTCGC  20
Template        3736052  ....................  3736071
```

>CP047592.1 Pseudomonas aeruginosa strain INP-43 chromosome, complete genome

```
product length = 202
Forward primer  1        TGCCTGGAACATAATCACCGT  21
Template        4261841  ............G........  4261861

Reverse primer  1        GTCGGTAGATAGCCTGTCGC  20
Template        4262042  ....................  4262023
```

>CP028132.1 Pseudomonas aeruginosa strain YB01 chromosome, complete genome

```
product length = 202
Forward primer  1        TGCCTGGAACATAATCACCGT  21
Template        3033842  ............G........  3033862

Reverse primer  1        GTCGGTAGATAGCCTGTCGC  20
Template        3034043  ....................  3034024
```

>CP025056.3 Pseudomonas aeruginosa strain PB367 chromosome, complete genome

```
product length = 202
Forward primer  1        TGCCTGGAACATAATCACCGT  21
Template        3335832  ............G........  3335852

Reverse primer  1        GTCGGTAGATAGCCTGTCGC  20
Template        3336033  ....................  3336014
```

>CP025055.2 Pseudomonas aeruginosa strain PB350 chromosome, complete genome

```
product length = 202
Forward primer  1        TGCCTGGAACATAATCACCGT  21
Template        3335832  ............G........  3335852

Reverse primer  1        GTCGGTAGATAGCCTGTCGC  20
Template        3336033  ....................  3336014
```

>CP047069.1 Pseudomonas aeruginosa strain Environ_1 chromosome

```
product length = 202
Forward primer  1        TGCCTGGAACATAATCACCGT  21
Template        2380717  ............G........  2380697

Reverse primer  1        GTCGGTAGATAGCCTGTCGC  20
Template        2380516  ....................  2380535
```

>CP047070.1 Pseudomonas aeruginosa strain Environ_2 chromosome

```
product length = 202
Forward primer  1        TGCCTGGAACATAATCACCGT  21
Template        2453814  ............G........  2453794

Reverse primer  1        GTCGGTAGATAGCCTGTCGC  20
Template        2453613  ....................  2453632
```

>CP039988.1 Pseudomonas aeruginosa strain T2436 chromosome, complete genome

```
product length = 202
Forward primer  1        TGCCTGGAACATAATCACCGT  21
Template        3172895  ............G........  3172915

Reverse primer  1        GTCGGTAGATAGCCTGTCGC  20
Template        3173096  ....................  3173077
```

>CP046069.1 Pseudomonas aeruginosa strain KRP1 chromosome, complete genome

```
product length = 202
Forward primer  1        TGCCTGGAACATAATCACCGT  21
Template        3225807  ............G........  3225827

Reverse primer  1        GTCGGTAGATAGCCTGTCGC  20
Template        3226008  ....................  3225989
```

>CP046060.1 Pseudomonas aeruginosa strain 1811-18R001 chromosome, complete genome

```
product length = 202
Forward primer  1        TGCCTGGAACATAATCACCGT  21
Template        3526379  ............G........  3526399

Reverse primer  1        GTCGGTAGATAGCCTGTCGC  20
Template        3526580  ....................  3526561
```

>CP046061.1 Pseudomonas aeruginosa strain 1811-13R031 chromosome, complete genome

```
product length = 202
Forward primer  1        TGCCTGGAACATAATCACCGT  21
Template        3526379  ............G........  3526399

Reverse primer  1        GTCGGTAGATAGCCTGTCGC  20
Template        3526580  ....................  3526561
```

>CP041945.1 Pseudomonas aeruginosa strain ST773 chromosome, complete genome

```
product length = 202
Forward primer  1        TGCCTGGAACATAATCACCGT  21
Template        3233067  ............G........  3233087

Reverse primer  1        GTCGGTAGATAGCCTGTCGC  20
Template        3233268  ....................  3233249
```

>CP045739.1 Pseudomonas aeruginosa strain AG1 chromosome, complete genome

```
product length = 202
Forward primer  1        TGCCTGGAACATAATCACCGT  21
Template        3519729  ............G........  3519749

Reverse primer  1        GTCGGTAGATAGCCTGTCGC  20
Template        3519930  ....................  3519911
```

>CP045768.1 Pseudomonas aeruginosa strain CFSAN084950 chromosome, complete genome

```
product length = 202
Forward primer  1        TGCCTGGAACATAATCACCGT  21
Template        2085232  ............G........  2085252

Reverse primer  1        GTCGGTAGATAGCCTGTCGC  20
Template        2085433  ....................  2085414
```

>CP042967.1 Pseudomonas aeruginosa PA99 chromosome, complete genome

```
product length = 202
Forward primer  1        TGCCTGGAACATAATCACCGT  21
Template        6778903  ............G........  6778883

Reverse primer  1        GTCGGTAGATAGCCTGTCGC  20
Template        6778702  ....................  6778721
```

>CP024630.1 Pseudomonas aeruginosa strain PA59 chromosome, complete genome

```
product length = 202
Forward primer  1        TGCCTGGAACATAATCACCGT  21
Template        3326468  ............G........  3326488

Reverse primer  1        GTCGGTAGATAGCCTGTCGC  20
Template        3326669  ....................  3326650
```

>CP044006.1 Pseudomonas aeruginosa strain E90 chromosome, complete genome

```
product length = 202
Forward primer  1        TGCCTGGAACATAATCACCGT  21
Template        3332418  ............G........  3332438

Reverse primer  1        GTCGGTAGATAGCCTGTCGC  20
Template        3332619  ....................  3332600
```

>CP043549.1 Pseudomonas aeruginosa strain GIMC5002:PAT-169 chromosome

```
product length = 202
Forward primer  1        TGCCTGGAACATAATCACCGT  21
Template        3144595  ............G........  3144575

Reverse primer  1        GTCGGTAGATAGCCTGTCGC  20
Template        3144394  ....................  3144413
```

>LR700248.1 Pseudomonas aeruginosa isolate ID40 genome assembly, chromosome: ID40_omosome

```
product length = 202
Forward primer  1       TGCCTGGAACATAATCACCGT  21
Template        339322  ............G........  339342

Reverse primer  1       GTCGGTAGATAGCCTGTCGC  20
Template        339523  ....................  339504
```

>CP042269.1 Pseudomonas aeruginosa strain HOU1 chromosome, complete genome

```
product length = 202
Forward primer  1        TGCCTGGAACATAATCACCGT  21
Template        2956145  ............G........  2956165

Reverse primer  1        GTCGGTAGATAGCCTGTCGC  20
Template        2956346  ....................  2956327
```

>CP043328.1 Pseudomonas aeruginosa strain CCUG 51971 chromosome, complete genome

```
product length = 202
Forward primer  1        TGCCTGGAACATAATCACCGT  21
Template        3329104  ............G........  3329124

Reverse primer  1        GTCGGTAGATAGCCTGTCGC  20
Template        3329305  ....................  3329286
```

>CP028959.1 Pseudomonas aeruginosa strain IMP66 chromosome, complete genome

```
product length = 202
Forward primer  1        TGCCTGGAACATAATCACCGT  21
Template        3120310  ............G........  3120330

Reverse primer  1        GTCGGTAGATAGCCTGTCGC  20
Template        3120511  ....................  3120492
```

>CP028848.1 Pseudomonas aeruginosa strain IMP67 chromosome, complete genome

```
product length = 202
Forward primer  1        TGCCTGGAACATAATCACCGT  21
Template        3105388  ............G........  3105408

Reverse primer  1        GTCGGTAGATAGCCTGTCGC  20
Template        3105589  ....................  3105570
```

>CP028849.1 Pseudomonas aeruginosa strain IMP68 chromosome, complete genome

```
product length = 202
Forward primer  1        TGCCTGGAACATAATCACCGT  21
Template        3105394  ............G........  3105414

Reverse primer  1        GTCGGTAGATAGCCTGTCGC  20
Template        3105595  ....................  3105576
```

>CP040684.1 Pseudomonas aeruginosa strain C79 chromosome, complete genome

```
product length = 202
Forward primer  1        TGCCTGGAACATAATCACCGT  21
Template        1505101  ............G........  1505081

Reverse primer  1        GTCGGTAGATAGCCTGTCGC  20
Template        1504900  ....................  1504919
```

>CP041785.1 Pseudomonas aeruginosa strain SCAID WND3-2019 chromosome

```
product length = 202
Forward primer  1        TGCCTGGAACATAATCACCGT  21
Template        2069467  ............G........  2069487

Reverse primer  1        GTCGGTAGATAGCCTGTCGC  20
Template        2069668  ....................  2069649
```

>CP041787.1 Pseudomonas aeruginosa strain SCAID WND1-2019 chromosome

```
product length = 202
Forward primer  1        TGCCTGGAACATAATCACCGT  21
Template        1023288  ............G........  1023308

Reverse primer  1        GTCGGTAGATAGCCTGTCGC  20
Template        1023489  ....................  1023470
```

>CP041786.1 Pseudomonas aeruginosa strain SCAID WND2-2019 chromosome

```
product length = 202
Forward primer  1        TGCCTGGAACATAATCACCGT  21
Template        2400019  ............G........  2399999

Reverse primer  1        GTCGGTAGATAGCCTGTCGC  20
Template        2399818  ....................  2399837
```

>CP041772.1 Pseudomonas aeruginosa strain 243931 chromosome, complete genome

```
product length = 202
Forward primer  1        TGCCTGGAACATAATCACCGT  21
Template        5532012  ............G........  5532032

Reverse primer  1        GTCGGTAGATAGCCTGTCGC  20
Template        5532213  ....................  5532194
```

>CP041771.1 Pseudomonas aeruginosa strain A681 chromosome, complete genome

```
product length = 202
Forward primer  1        TGCCTGGAACATAATCACCGT  21
Template        3209692  ............G........  3209712

Reverse primer  1        GTCGGTAGATAGCCTGTCGC  20
Template        3209893  ....................  3209874
```

>CP041774.1 Pseudomonas aeruginosa strain 60503 chromosome, complete genome

```
product length = 202
Forward primer  1        TGCCTGGAACATAATCACCGT  21
Template        3119857  ............G........  3119877

Reverse primer  1        GTCGGTAGATAGCCTGTCGC  20
Template        3120058  ....................  3120039
```

>LR657304.1 Pseudomonas aeruginosa strain PAK genome assembly, chromosome: 1

```
product length = 202
Forward primer  1        TGCCTGGAACATAATCACCGT  21
Template        3100468  ............G........  3100488

Reverse primer  1        GTCGGTAGATAGCCTGTCGC  20
Template        3100669  ....................  3100650
```

>CP041013.1 Pseudomonas aeruginosa strain FDAARGOS_610 chromosome, complete genome

```
product length = 202
Forward primer  1        TGCCTGGAACATAATCACCGT  21
Template        3452522  ............G........  3452502

Reverse primer  1        GTCGGTAGATAGCCTGTCGC  20
Template        3452321  ....................  3452340
```

>CP040127.1 Pseudomonas aeruginosa strain PA298 chromosome, complete genome

```
product length = 202
Forward primer  1        TGCCTGGAACATAATCACCGT  21
Template        3192094  ............G........  3192114

Reverse primer  1        GTCGGTAGATAGCCTGTCGC  20
Template        3192295  ....................  3192276
```

>LR590474.1 Pseudomonas aeruginosa strain NCTC13618 genome assembly, chromosome: 1

```
product length = 202
Forward primer  1         TGCCTGGAACATAATCACCGT  21
Template        3269344   ............G........  3269364

Reverse primer  1         GTCGGTAGATAGCCTGTCGC  20
Template        3269545   ....................  3269526
```

>LR590473.1 Pseudomonas aeruginosa strain NCTC13359 genome assembly, chromosome: 1

```
product length = 202
Forward primer  1         TGCCTGGAACATAATCACCGT  21
Template        3776132   ............G........  3776152

Reverse primer  1         GTCGGTAGATAGCCTGTCGC  20
Template        3776333   ....................  3776314
```

>CP039749.1 Pseudomonas aeruginosa strain PRD-10 chromosome

```
product length = 202
Forward primer  1         TGCCTGGAACATAATCACCGT  21
Template        2665212   ............G........  2665232

Reverse primer  1         GTCGGTAGATAGCCTGTCGC  20
Template        2665413   ....................  2665394
```

>CP039293.1 Pseudomonas aeruginosa strain PABL048 chromosome, complete genome

```
product length = 202
Forward primer  1         TGCCTGGAACATAATCACCGT  21
Template        3339067   ............G........  3339087

Reverse primer  1         GTCGGTAGATAGCCTGTCGC  20
Template        3339268   ....................  3339249
```

>CP038661.1 Pseudomonas aeruginosa strain AJ D 2 chromosome

```
product length = 202
Forward primer  1         TGCCTGGAACATAATCACCGT  21
Template        2460607   ............G........  2460587

Reverse primer  1         GTCGGTAGATAGCCTGTCGC  20
Template        2460406   ....................  2460425
```

>CP037925.1 Pseudomonas aeruginosa strain AES1M chromosome, complete genome

```
product length = 202
Forward primer  1         TGCCTGGAACATAATCACCGT  21
Template        2954283   ............G........  2954263

Reverse primer  1         GTCGGTAGATAGCCTGTCGC  20
Template        2954082   ....................  2954101
```

>CP037926.1 Pseudomonas aeruginosa strain AES1R chromosome, complete genome

```
product length = 202
Forward primer  1        TGCCTGGAACATAATCACCGT  21
Template        3123246  ............G........  3123266

Reverse primer  1        GTCGGTAGATAGCCTGTCGC  20
Template        3123447  ....................  3123428
```

>CP028332.1 Pseudomonas aeruginosa strain PA-VAP-1 chromosome

```
product length = 202
Forward primer  1        TGCCTGGAACATAATCACCGT  21
Template        5367706  ............G........  5367726

Reverse primer  1        GTCGGTAGATAGCCTGTCGC  20
Template        5367907  ....................  5367888
```

>CP028331.1 Pseudomonas aeruginosa strain PA-VAP-2 chromosome

```
product length = 202
Forward primer  1        TGCCTGGAACATAATCACCGT  21
Template        2689971  ............G........  2689951

Reverse primer  1        GTCGGTAGATAGCCTGTCGC  20
Template        2689770  ....................  2689789
```

>CP028330.1 Pseudomonas aeruginosa strain PA-VAP-3 chromosome

```
product length = 202
Forward primer  1        TGCCTGGAACATAATCACCGT  21
Template        6465882  ............G........  6465862

Reverse primer  1        GTCGGTAGATAGCCTGTCGC  20
Template        6465681  ....................  6465700
```

>CP028368.1 Pseudomonas aeruginosa strain PA-VAP-4 chromosome

```
product length = 202
Forward primer  1        TGCCTGGAACATAATCACCGT  21
Template        2483831  ............G........  2483811

Reverse primer  1        GTCGGTAGATAGCCTGTCGC  20
Template        2483630  ....................  2483649
```

>CP031677.1 Pseudomonas aeruginosa strain E80 chromosome, complete genome

```
product length = 202
Forward primer  1        TGCCTGGAACATAATCACCGT  21
Template        1733154  ............G........  1733134

Reverse primer  1        GTCGGTAGATAGCCTGTCGC  20
Template        1732953  ....................  1732972
```

>CP022478.1 Pseudomonas aeruginosa strain LW chromosome, complete genome

```
product length = 202
Forward primer  1        TGCCTGGAACATAATCACCGT  21
Template        1907562  ............G........  1907582

Reverse primer  1        GTCGGTAGATAGCCTGTCGC  20
Template        1907763  ....................  1907744
```

>LR134342.1 Pseudomonas aeruginosa strain NCTC10728 genome assembly, chromosome: 1

```
product length = 202
Forward primer  1        TGCCTGGAACATAATCACCGT  21
Template        6061259  ............G........  6061279

Reverse primer  1        GTCGGTAGATAGCCTGTCGC  20
Template        6061460  ....................  6061441
```

>LR134330.1 Pseudomonas aeruginosa strain NCTC13715 genome assembly, chromosome: 1

```
product length = 202
Forward primer  1        TGCCTGGAACATAATCACCGT  21
Template        5053885  ............G........  5053905

Reverse primer  1        GTCGGTAGATAGCCTGTCGC  20
Template        5054086  ....................  5054067
```

>LR134309.1 Pseudomonas aeruginosa strain NCTC12903 genome assembly, chromosome: 1

```
product length = 202
Forward primer  1        TGCCTGGAACATAATCACCGT  21
Template        3207744  ............G........  3207764

Reverse primer  1        GTCGGTAGATAGCCTGTCGC  20
Template        3207945  ....................  3207926
```

>LR134308.1 Pseudomonas aeruginosa strain NCTC11445 genome assembly, chromosome: 1

```
product length = 202
Forward primer  1        TGCCTGGAACATAATCACCGT  21
Template        2277265  ............G........  2277285

Reverse primer  1        GTCGGTAGATAGCCTGTCGC  20
Template        2277466  ....................  2277447
```

>CP034434.1 Pseudomonas aeruginosa strain SP2230 chromosome, complete genome

```
product length = 202
Forward primer  1        TGCCTGGAACATAATCACCGT  21
Template        2213809  ............G........  2213829

Reverse primer  1        GTCGGTAGATAGCCTGTCGC  20
Template        2214010  ....................  2213991
```

>CP034436.1 Pseudomonas aeruginosa strain B17932 chromosome, complete genome

```
product length = 202
Forward primer  1        TGCCTGGAACATAATCACCGT  21
Template        3142340  ............G........  3142320

Reverse primer  1        GTCGGTAGATAGCCTGTCGC  20
Template        3142139  ....................  3142158
```

>CP034409.1 Pseudomonas aeruginosa strain SP4527 chromosome, complete genome

```
product length = 202
Forward primer  1        TGCCTGGAACATAATCACCGT  21
Template        1636016  ............G........  1636036

Reverse primer  1        GTCGGTAGATAGCCTGTCGC  20
Template        1636217  ....................  1636198
```

>CP034369.1 Pseudomonas aeruginosa strain SP4371 chromosome, complete genome

```
product length = 202
Forward primer  1        TGCCTGGAACATAATCACCGT  21
Template        2542555  ............G........  2542575

Reverse primer  1        GTCGGTAGATAGCCTGTCGC  20
Template        2542756  ....................  2542737
```

>CP034368.1 Pseudomonas aeruginosa strain B41226 chromosome, complete genome

```
product length = 202
Forward primer  1        TGCCTGGAACATAATCACCGT  21
Template        2868745  ............G........  2868725

Reverse primer  1        GTCGGTAGATAGCCTGTCGC  20
Template        2868544  ....................  2868563
```

>CP034354.1 Pseudomonas aeruginosa strain IMP-13 chromosome, complete genome

```
product length = 202
Forward primer  1        TGCCTGGAACATAATCACCGT  21
Template        6461212  ............G........  6461232

Reverse primer  1        GTCGGTAGATAGCCTGTCGC  20
Template        6461413  ....................  6461394
```

>LR130537.1 Pseudomonas aeruginosa isolate paerg012 genome assembly, chromosome: 0

```
product length = 202
Forward primer  1        TGCCTGGAACATAATCACCGT  21
Template        3067435  ............G........  3067455

Reverse primer  1        GTCGGTAGATAGCCTGTCGC  20
Template        3067636  ....................  3067617
```

>LR130536.1 Pseudomonas aeruginosa isolate paerg010 genome assembly, chromosome: 0

```
product length = 202
Forward primer  1        TGCCTGGAACATAATCACCGT  21
Template        3067419  ............G........  3067439

Reverse primer  1        GTCGGTAGATAGCCTGTCGC  20
Template        3067620  ....................  3067601
```

>LR130535.1 Pseudomonas aeruginosa isolate paerg011 genome assembly, chromosome: 0

```
product length = 202
Forward primer  1        TGCCTGGAACATAATCACCGT  21
Template        3067542  ............G........  3067562

Reverse primer  1        GTCGGTAGATAGCCTGTCGC  20
Template        3067743  ....................  3067724
```

>LR130534.1 Pseudomonas aeruginosa isolate paerg005 genome assembly, chromosome: 0

```
product length = 202
Forward primer  1        TGCCTGGAACATAATCACCGT  21
Template        3348100  ............G........  3348120

Reverse primer  1        GTCGGTAGATAGCCTGTCGC  20
Template        3348301  ....................  3348282
```

>LR130533.1 Pseudomonas aeruginosa isolate paerg009 genome assembly, chromosome: 0

```
product length = 202
Forward primer  1        TGCCTGGAACATAATCACCGT  21
Template        6662954  ............G........  6662974

Reverse primer  1        GTCGGTAGATAGCCTGTCGC  20
Template        6663155  ....................  6663136
```

>LR130531.1 Pseudomonas aeruginosa isolate paerg004 genome assembly, chromosome: 0

```
product length = 202
Forward primer  1       TGCCTGGAACATAATCACCGT  21
Template        383926  ............G........  383946

Reverse primer  1       GTCGGTAGATAGCCTGTCGC  20
Template        384127  ....................  384108
```

>LR130530.1 Pseudomonas aeruginosa isolate paerg003 genome assembly, chromosome: 0

```
product length = 202
Forward primer  1        TGCCTGGAACATAATCACCGT  21
Template        3067457  ............G........  3067477

Reverse primer  1        GTCGGTAGATAGCCTGTCGC  20
Template        3067658  ....................  3067639
```

>LR130528.1 Pseudomonas aeruginosa isolate paerg000 genome assembly, chromosome: 0

```
product length = 202
Forward primer  1        TGCCTGGAACATAATCACCGT  21
Template        3772398  ............G........  3772378

Reverse primer  1        GTCGGTAGATAGCCTGTCGC  20
Template        3772197  ....................  3772216
```

>LR130527.1 Pseudomonas aeruginosa isolate paerg002 genome assembly, chromosome: 0

```
product length = 202
Forward primer  1        TGCCTGGAACATAATCACCGT  21
Template        6181870  ............G........  6181890

Reverse primer  1        GTCGGTAGATAGCCTGTCGC  20
Template        6182071  ....................  6182052
```

>CP033832.1 Pseudomonas aeruginosa strain FDAARGOS_505 chromosome, complete genome

```
product length = 202
Forward primer  1        TGCCTGGAACATAATCACCGT  21
Template        4913468  ............G........  4913488

Reverse primer  1        GTCGGTAGATAGCCTGTCGC  20
Template        4913669  ....................  4913650
```

>CP033835.1 Pseudomonas aeruginosa strain FDAARGOS_570 chromosome, complete genome

```
product length = 202
Forward primer  1        TGCCTGGAACATAATCACCGT  21
Template        3863604  ............G........  3863584

Reverse primer  1        GTCGGTAGATAGCCTGTCGC  20
Template        3863403  ....................  3863422
```

>CP033833.1 Pseudomonas aeruginosa strain FDAARGOS_571 chromosome, complete genome

```
product length = 202
Forward primer  1        TGCCTGGAACATAATCACCGT  21
Template        6578363  ............G........  6578383

Reverse primer  1        GTCGGTAGATAGCCTGTCGC  20
Template        6578564  ....................  6578545
```

>CP033843.1 Pseudomonas aeruginosa strain FDAARGOS_501 chromosome, complete genome

```
product length = 202
Forward primer  1        TGCCTGGAACATAATCACCGT  21
Template        5816326  ............G........  5816346

Reverse primer  1        GTCGGTAGATAGCCTGTCGC  20
Template        5816527  ....................  5816508
```

>CP033771.1 Pseudomonas aeruginosa strain FDAARGOS_532 chromosome, complete genome

```
product length = 202
Forward primer  1        TGCCTGGAACATAATCACCGT  21
Template        6833457  ............G........  6833437

Reverse primer  1        GTCGGTAGATAGCCTGTCGC  20
Template        6833256  ....................  6833275
```

>CP033684.1 Pseudomonas aeruginosa strain H26027 chromosome, complete genome

```
product length = 202
Forward primer  1        TGCCTGGAACATAATCACCGT  21
Template        3513257  ............G........  3513277

Reverse primer  1        GTCGGTAGATAGCCTGTCGC  20
Template        3513458  ....................  3513439
```

>CP033686.1 Pseudomonas aeruginosa strain H25883 chromosome, complete genome

```
product length = 202
Forward primer  1        TGCCTGGAACATAATCACCGT  21
Template        3191161  ............G........  3191181

Reverse primer  1        GTCGGTAGATAGCCTGTCGC  20
Template        3191362  ....................  3191343
```

>CP033685.1 Pseudomonas aeruginosa strain H26023 chromosome, complete genome

```
product length = 202
Forward primer  1        TGCCTGGAACATAATCACCGT  21
Template        3288732  ............G........  3288752

Reverse primer  1        GTCGGTAGATAGCCTGTCGC  20
Template        3288933  ....................  3288914
```

>CP033439.1 Pseudomonas aeruginosa strain SP4528 chromosome, complete genome

```
product length = 202
Forward primer  1        TGCCTGGAACATAATCACCGT  21
Template        3322533  ............G........  3322513

Reverse primer  1        GTCGGTAGATAGCCTGTCGC  20
Template        3322332  ....................  3322351
```

>CP033432.1 Pseudomonas aeruginosa strain BA15561 chromosome, complete genome

```
product length = 202
Forward primer  1        TGCCTGGAACATAATCACCGT  21
Template        1563381  ............G........  1563361

Reverse primer  1        GTCGGTAGATAGCCTGTCGC  20
Template        1563180  ....................  1563199
```

>CP033084.1 Pseudomonas aeruginosa strain PA-3 chromosome, complete genome

```
product length = 202
Forward primer  1        TGCCTGGAACATAATCACCGT  21
Template        1422401  ............G........  1422381

Reverse primer  1        GTCGGTAGATAGCCTGTCGC  20
Template        1422200  ....................  1422219
```

>CP030075.1 Pseudomonas aeruginosa strain 6762 chromosome

```
product length = 202
Forward primer  1        TGCCTGGAACATAATCACCGT  21
Template        5226633  ............G........  5226653

Reverse primer  1        GTCGGTAGATAGCCTGTCGC  20
Template        5226834  ....................  5226815
```

>CP032552.1 Pseudomonas aeruginosa strain PA34 chromosome, complete genome

```
product length = 202
Forward primer  1        TGCCTGGAACATAATCACCGT  21
Template        3249045  ............G........  3249065

Reverse primer  1        GTCGGTAGATAGCCTGTCGC  20
Template        3249246  ....................  3249227
```

>CP032761.1 Pseudomonas aeruginosa strain 268 chromosome, complete genome

```
product length = 202
Forward primer  1        TGCCTGGAACATAATCACCGT  21
Template        3084996  ............G........  3084976

Reverse primer  1        GTCGGTAGATAGCCTGTCGC  20
Template        3084795  ....................  3084814
```

>CP028584.2 Pseudomonas aeruginosa strain WCHPA075019 chromosome, complete genome

```
product length = 202
Forward primer  1        TGCCTGGAACATAATCACCGT  21
Template        3514341  ............G........  3514361

Reverse primer  1        GTCGGTAGATAGCCTGTCGC  20
Template        3514542  ....................  3514523
```

>CP031877.1 Pseudomonas aeruginosa strain WPB100 chromosome

```
product length = 202
Forward primer  1        TGCCTGGAACATAATCACCGT  21
Template        3495935  ............G........  3495955

Reverse primer  1        GTCGGTAGATAGCCTGTCGC  20
Template        3496136  ....................  3496117
```

>CP031876.1 Pseudomonas aeruginosa strain WPB101 chromosome

```
product length = 202
Forward primer  1        TGCCTGGAACATAATCACCGT  21
Template        3361984  ............G........  3362004

Reverse primer  1        GTCGGTAGATAGCCTGTCGC  20
Template        3362185  ....................  3362166
```

>CP031878.1 Pseudomonas aeruginosa strain WPB099 chromosome

```
product length = 202
Forward primer  1        TGCCTGGAACATAATCACCGT  21
Template        2989936  ............G........  2989956

Reverse primer  1        GTCGGTAGATAGCCTGTCGC  20
Template        2990137  ....................  2990118
```

>CP031879.1 Pseudomonas aeruginosa strain WPB098 chromosome

```
product length = 202
Forward primer  1        TGCCTGGAACATAATCACCGT  21
Template        3100070  ............G........  3100090

Reverse primer  1        GTCGGTAGATAGCCTGTCGC  20
Template        3100271  ....................  3100252
```

>CP029605.1 Pseudomonas aeruginosa strain 24Pae112 chromosome, complete genome

```
product length = 202
Forward primer  1        TGCCTGGAACATAATCACCGT  21
Template        3345645  ............G........  3345665

Reverse primer  1        GTCGGTAGATAGCCTGTCGC  20
Template        3345846  ....................  3345827
```

>CP031660.1 Pseudomonas aeruginosa strain PABL017 chromosome, complete genome

```
product length = 202
Forward primer  1        TGCCTGGAACATAATCACCGT  21
Template        3117275  ............G........  3117295

Reverse primer  1        GTCGGTAGATAGCCTGTCGC  20
Template        3117476  ....................  3117457
```

>CP031659.1 Pseudomonas aeruginosa strain PABL012 chromosome, complete genome

```
product length = 202
Forward primer  1        TGCCTGGAACATAATCACCGT  21
Template        3226563  ............G........  3226583

Reverse primer  1        GTCGGTAGATAGCCTGTCGC  20
Template        3226764  ....................  3226745
```

>CP035739.1 Pseudomonas aeruginosa strain 1334/14 chromosome, complete genome

```
product length = 202
Forward primer  1       TGCCTGGAACATAATCACCGT  21
Template        779612  ............G........  779592

Reverse primer  1       GTCGGTAGATAGCCTGTCGC  20
Template        779411  ....................  779430
```

>CP031449.2 Pseudomonas aeruginosa strain 97 chromosome, complete genome

```
product length = 202
Forward primer  1        TGCCTGGAACATAATCACCGT  21
Template        3380131  ............G........  3380151

Reverse primer  1        GTCGGTAGATAGCCTGTCGC  20
Template        3380332  ....................  3380313
```

>LS998783.1 Pseudomonas aeruginosa isolate 1 genome assembly, chromosome: 1

```
product length = 202
Forward primer  1        TGCCTGGAACATAATCACCGT  21
Template        3674623  ............G........  3674603

Reverse primer  1        GTCGGTAGATAGCCTGTCGC  20
Template        3674422  ....................  3674441
```

>CP030913.1 Pseudomonas aeruginosa strain Y89 chromosome, complete genome

```
product length = 202
Forward primer  1        TGCCTGGAACATAATCACCGT  21
Template        3305307  ............G........  3305327

Reverse primer  1        GTCGGTAGATAGCCTGTCGC  20
Template        3305508  ....................  3305489
```

>CP030912.1 Pseudomonas aeruginosa strain Y82 chromosome, complete genome

```
product length = 202
Forward primer  1        TGCCTGGAACATAATCACCGT  21
Template        3407755  ............G........  3407775

Reverse primer  1        GTCGGTAGATAGCCTGTCGC  20
Template        3407956  ....................  3407937
```

>CP030910.1 Pseudomonas aeruginosa strain Y31 chromosome, complete genome

```
product length = 202
Forward primer  1        TGCCTGGAACATAATCACCGT  21
Template        4100649  ............G........  4100669

Reverse primer  1        GTCGGTAGATAGCCTGTCGC  20
Template        4100850  ....................  4100831
```

>CP030861.1 Pseudomonas aeruginosa strain HS9 chromosome, complete genome

```
product length = 202
Forward primer  1        TGCCTGGAACATAATCACCGT  21
Template        6099340  ............G........  6099320

Reverse primer  1        GTCGGTAGATAGCCTGTCGC  20
Template        6099139  ....................  6099158
```

>CP030327.1 Pseudomonas aeruginosa strain AR_458 chromosome, complete genome

```
product length = 202
Forward primer  1        TGCCTGGAACATAATCACCGT  21
Template        6511520  ............G........  6511540

Reverse primer  1        GTCGGTAGATAGCCTGTCGC  20
Template        6511721  ....................  6511702
```

>CP030351.1 Pseudomonas aeruginosa strain AR_460 chromosome, complete genome

```
product length = 202
Forward primer  1        TGCCTGGAACATAATCACCGT  21
Template        1427407  ............G........  1427427

Reverse primer  1        GTCGGTAGATAGCCTGTCGC  20
Template        1427608  ....................  1427589
```

>CP030328.1 Pseudomonas aeruginosa strain AR_455 chromosome, complete genome

```
product length = 202
Forward primer  1        TGCCTGGAACATAATCACCGT  21
Template        4755646  ............G........  4755666

Reverse primer  1        GTCGGTAGATAGCCTGTCGC  20
Template        4755847  ....................  4755828
```

>LS483497.1 Pseudomonas aeruginosa strain NCTC9433 genome assembly, chromosome: 1

```
product length = 202
Forward primer  1        TGCCTGGAACATAATCACCGT  21
Template        3062133  ............G........  3062153

Reverse primer  1        GTCGGTAGATAGCCTGTCGC  20
Template        3062334  ....................  3062315
```

>CP023255.1 Pseudomonas aeruginosa strain CCUG 70744 chromosome, complete genome

```
product length = 202
Forward primer  1        TGCCTGGAACATAATCACCGT  21
Template        1244053  ............G........  1244073

Reverse primer  1        GTCGGTAGATAGCCTGTCGC  20
Template        1244254  ....................  1244235
```

>CP029660.1 Pseudomonas aeruginosa strain AR_0446 chromosome, complete genome

```
product length = 202
Forward primer  1        TGCCTGGAACATAATCACCGT  21
Template        1845250  ............G........  1845270

Reverse primer  1        GTCGGTAGATAGCCTGTCGC  20
Template        1845451  ....................  1845432
```

>CP029148.1 Pseudomonas aeruginosa strain AR_0440 chromosome

```
product length = 202
Forward primer  1        TGCCTGGAACATAATCACCGT  21
Template        2318213  ............G........  2318233

Reverse primer  1        GTCGGTAGATAGCCTGTCGC  20
Template        2318414  ....................  2318395
```

>CP029147.1 Pseudomonas aeruginosa strain AR_0443 chromosome

```
product length = 202
Forward primer  1        TGCCTGGAACATAATCACCGT  21
Template        6749654  ............G........  6749674

Reverse primer  1        GTCGGTAGATAGCCTGTCGC  20
Template        6749855  ....................  6749836
```

>CP029097.1 Pseudomonas aeruginosa strain AR439 chromosome, complete genome

```
product length = 202
Forward primer  1        TGCCTGGAACATAATCACCGT  21
Template        3830075  ............G........  3830055

Reverse primer  1        GTCGGTAGATAGCCTGTCGC  20
Template        3829874  ....................  3829893
```

>CP029090.1 Pseudomonas aeruginosa strain AR442 chromosome, complete genome

```
product length = 202
Forward primer  1        TGCCTGGAACATAATCACCGT  21
Template        2449325  ............G........  2449345

Reverse primer  1        GTCGGTAGATAGCCTGTCGC  20
Template        2449526  ....................  2449507
```

>CP029088.1 Pseudomonas aeruginosa strain AR445 chromosome, complete genome

```
product length = 202
Forward primer  1        TGCCTGGAACATAATCACCGT  21
Template        3494887  ............G........  3494907

Reverse primer  1        GTCGGTAGATAGCCTGTCGC  20
Template        3495088  ....................  3495069
```

>CP028917.1 Pseudomonas aeruginosa strain JB2 chromosome, complete genome

```
product length = 202
Forward primer  1        TGCCTGGAACATAATCACCGT  21
Template        3892014  ............G........  3891994

Reverse primer  1        GTCGGTAGATAGCCTGTCGC  20
Template        3891813  ....................  3891832
```

>CP023316.1 Pseudomonas aeruginosa strain PPF-1 chromosome, complete genome

```
product length = 202
Forward primer  1        TGCCTGGAACATAATCACCGT  21
Template        3452156  ............G........  3452176

Reverse primer  1        GTCGGTAGATAGCCTGTCGC  20
Template        3452357  ....................  3452338
```

>CP028162.1 Pseudomonas aeruginosa strain MRSN12280 chromosome, complete genome

```
product length = 202
Forward primer  1        TGCCTGGAACATAATCACCGT  21
Template        2661487  ............G........  2661467

Reverse primer  1        GTCGGTAGATAGCCTGTCGC  20
Template        2661286  ....................  2661305
```

>CP027538.1 Pseudomonas aeruginosa strain AR_0095 chromosome, complete genome

```
product length = 202
Forward primer  1        TGCCTGGAACATAATCACCGT  21
Template        6400662  ............G........  6400642

Reverse primer  1        GTCGGTAGATAGCCTGTCGC  20
Template        6400461  ....................  6400480
```

>CP027166.1 Pseudomonas aeruginosa strain AR_0357 chromosome, complete genome

```
product length = 202
Forward primer  1        TGCCTGGAACATAATCACCGT  21
Template        5420018  ............G........  5419998

Reverse primer  1        GTCGGTAGATAGCCTGTCGC  20
Template        5419817  ....................  5419836
```

>CP027172.1 Pseudomonas aeruginosa strain AR_0353 chromosome, complete genome

```
product length = 202
Forward primer  1        TGCCTGGAACATAATCACCGT  21
Template        3805762  ............G........  3805742

Reverse primer  1        GTCGGTAGATAGCCTGTCGC  20
Template        3805561  ....................  3805580
```

>CP027171.1 Pseudomonas aeruginosa strain AR_0354 chromosome, complete genome

```
product length = 202
Forward primer  1        TGCCTGGAACATAATCACCGT  21
Template        1119668  ............G........  1119688

Reverse primer  1        GTCGGTAGATAGCCTGTCGC  20
Template        1119869  ....................  1119850
```

>LT969520.1 Pseudomonas aeruginosa isolate RW109 genome assembly, chromosome: Main_chromosome

```
product length = 202
Forward primer  1        TGCCTGGAACATAATCACCGT  21
Template        3408390  ............G........  3408410

Reverse primer  1        GTCGGTAGATAGCCTGTCGC  20
Template        3408591  ....................  3408572
```

>CP025051.1 Pseudomonas aeruginosa strain PB353 chromosome, complete genome

```
product length = 202
Forward primer  1        TGCCTGGAACATAATCACCGT  21
Template        3143178  ............G........  3143198

Reverse primer  1        GTCGGTAGATAGCCTGTCGC  20
Template        3143379  ....................  3143360
```

>CP025049.1 Pseudomonas aeruginosa strain PB369 chromosome, complete genome

```
product length = 202
Forward primer  1        TGCCTGGAACATAATCACCGT  21
Template        3231235  ............G........  3231215

Reverse primer  1        GTCGGTAGATAGCCTGTCGC  20
Template        3231034  ....................  3231053
```

>CP025053.1 Pseudomonas aeruginosa strain PB354 chromosome, complete genome

```
product length = 202
Forward primer  1        TGCCTGGAACATAATCACCGT  21
Template        3143178  ............G........  3143198

Reverse primer  1        GTCGGTAGATAGCCTGTCGC  20
Template        3143379  ....................  3143360
```

>CP024477.1 Pseudomonas aeruginosa strain 12939 chromosome, complete genome

```
product length = 202
Forward primer  1        TGCCTGGAACATAATCACCGT  21
Template        3078110  ............G........  3078130

Reverse primer  1        GTCGGTAGATAGCCTGTCGC  20
```

```
Template        3078311  ....................  3078292
```

>KY860571.1 Pseudomonas aeruginosa strain 31448cz

```
product length = 202
Forward primer  1     TGCCTGGAACATAATCACCGT  21
Template        6865  ............G........  6885

Reverse primer  1     GTCGGTAGATAGCCTGTCGC  20
Template        7066  ....................  7047
```

>CP022526.1 Pseudomonas aeruginosa strain Ocean-1155, complete genome

```
product length = 202
Forward primer  1      TGCCTGGAACATAATCACCGT  21
Template        10785  ............G........  10765

Reverse primer  1      GTCGGTAGATAGCCTGTCGC  20
Template        10584  ....................  10603
```

>CP022525.1 Pseudomonas aeruginosa strain Ocean-1175, complete genome

```
product length = 202
Forward primer  1        TGCCTGGAACATAATCACCGT  21
Template        5469317  ............G........  5469297

Reverse primer  1        GTCGGTAGATAGCCTGTCGC  20
Template        5469116  ....................  5469135
```

>CP019338.1 Pseudomonas aeruginosa strain L10, complete genome

```
product length = 202
Forward primer  1        TGCCTGGAACATAATCACCGT  21
Template        3236228  ............G........  3236248

Reverse primer  1        GTCGGTAGATAGCCTGTCGC  20
Template        3236429  ....................  3236410
```

>CP022002.1 Pseudomonas aeruginosa strain Pa1242, complete genome

```
product length = 202
Forward primer  1        TGCCTGGAACATAATCACCGT  21
Template        4007197  ............G........  4007177

Reverse primer  1        GTCGGTAGATAGCCTGTCGC  20
Template        4006996  ....................  4007015
```

>CP022001.1 Pseudomonas aeruginosa strain Pa1207, complete genome

```
product length = 202
Forward primer  1        TGCCTGGAACATAATCACCGT  21
Template        4054490  ............G........  4054470
```

```
Reverse primer  1        GTCGGTAGATAGCCTGTCGC  20
Template        4054289  ....................  4054308
```

>CP022000.1 Pseudomonas aeruginosa strain Pa127, complete genome

```
product length = 202
Forward primer  1        TGCCTGGAACATAATCACCGT  21
Template        3415998  ............G........  3416018

Reverse primer  1        GTCGGTAGATAGCCTGTCGC  20
Template        3416199  ....................  3416180
```

>CP021999.1 Pseudomonas aeruginosa strain Pa84, complete genome

```
product length = 202
Forward primer  1        TGCCTGGAACATAATCACCGT  21
Template        3156005  ............G........  3156025

Reverse primer  1        GTCGGTAGATAGCCTGTCGC  20
Template        3156206  ....................  3156187
```

>LT883143.1 Pseudomonas aeruginosa C-NN2 isolate early isolate NN2 (clone C) genome assembly, chromosome: I

```
product length = 202
Forward primer  1        TGCCTGGAACATAATCACCGT  21
Template        3313934  ............G........  3313954

Reverse primer  1        GTCGGTAGATAGCCTGTCGC  20
Template        3314135  ....................  3314116
```

>CP021774.1 Pseudomonas aeruginosa strain Pa124, complete genome

```
product length = 202
Forward primer  1        TGCCTGGAACATAATCACCGT  21
Template        3367955  ............G........  3367975

Reverse primer  1        GTCGGTAGATAGCCTGTCGC  20
Template        3368156  ....................  3368137
```

>CP021775.1 Pseudomonas aeruginosa strain Pa58, complete genome

```
product length = 202
Forward primer  1        TGCCTGGAACATAATCACCGT  21
Template        3552849  ............G........  3552869

Reverse primer  1        GTCGGTAGATAGCCTGTCGC  20
Template        3553050  ....................  3553031
```

>CP015650.1 Pseudomonas aeruginosa strain Pb18 genome

```
product length = 202
Forward primer  1        TGCCTGGAACATAATCACCGT  21
```

```
Template        1098454  ............G........  1098474

Reverse primer  1        GTCGGTAGATAGCCTGTCGC  20
Template        1098655  ....................  1098636
```

>CP015649.1 Pseudomonas aeruginosa strain M28A1 genome

```
product length = 202
Forward primer  1        TGCCTGGAACATAATCACCGT  21
Template        6488603  ............G........  6488623

Reverse primer  1        GTCGGTAGATAGCCTGTCGC  20
Template        6488804  ....................  6488785
```

>CP015648.1 Pseudomonas aeruginosa strain M8A4 genome

```
product length = 202
Forward primer  1        TGCCTGGAACATAATCACCGT  21
Template        3132361  ............G........  3132341

Reverse primer  1        GTCGGTAGATAGCCTGTCGC  20
Template        3132160  ....................  3132179
```

>CP015647.1 Pseudomonas aeruginosa strain M8A1 genome

```
product length = 202
Forward primer  1        TGCCTGGAACATAATCACCGT  21
Template        3083282  ............G........  3083302

Reverse primer  1        GTCGGTAGATAGCCTGTCGC  20
Template        3083483  ....................  3083464
```

>CP020704.1 Pseudomonas aeruginosa strain PASGNDM699, complete genome

```
product length = 202
Forward primer  1        TGCCTGGAACATAATCACCGT  21
Template        3280371  ............G........  3280391

Reverse primer  1        GTCGGTAGATAGCCTGTCGC  20
Template        3280572  ....................  3280553
```

>CP020703.1 Pseudomonas aeruginosa strain PASGNDM345, complete genome

```
product length = 202
Forward primer  1        TGCCTGGAACATAATCACCGT  21
Template        3280363  ............G........  3280383

Reverse primer  1        GTCGGTAGATAGCCTGTCGC  20
Template        3280564  ....................  3280545
```

>CP008858.2 Pseudomonas aeruginosa strain F63912 chromosome, complete genome

```
product length = 202
```

```
Forward primer  1        TGCCTGGAACATAATCACCGT  21
Template        3165980  ............G........  3166000

Reverse primer  1        GTCGGTAGATAGCCTGTCGC  20
Template        3166181  ....................  3166162
```

>CP020659.1 Pseudomonas aeruginosa PAK chromosome, complete genome

```
product length = 202
Forward primer  1        TGCCTGGAACATAATCACCGT  21
Template        1771263  ............G........  1771243

Reverse primer  1        GTCGGTAGATAGCCTGTCGC  20
Template        1771062  ....................  1771081
```

>CP008872.2 Pseudomonas aeruginosa strain X78812 chromosome, complete genome

```
product length = 202
Forward primer  1        TGCCTGGAACATAATCACCGT  21
Template        3071381  ............G........  3071401

Reverse primer  1        GTCGGTAGATAGCCTGTCGC  20
Template        3071582  ....................  3071563
```

>CP008871.2 Pseudomonas aeruginosa strain W45909 chromosome, complete genome

```
product length = 202
Forward primer  1        TGCCTGGAACATAATCACCGT  21
Template        3228220  ............G........  3228240

Reverse primer  1        GTCGGTAGATAGCCTGTCGC  20
Template        3228421  ....................  3228402
```

>CP008870.2 Pseudomonas aeruginosa strain W36662 chromosome, complete genome

```
product length = 202
Forward primer  1        TGCCTGGAACATAATCACCGT  21
Template        2982758  ............G........  2982738

Reverse primer  1        GTCGGTAGATAGCCTGTCGC  20
Template        2982557  ....................  2982576
```

>CP008869.2 Pseudomonas aeruginosa strain W16407 chromosome, complete genome

```
product length = 202
Forward primer  1        TGCCTGGAACATAATCACCGT  21
Template        3337351  ............G........  3337371

Reverse primer  1        GTCGGTAGATAGCCTGTCGC  20
Template        3337552  ....................  3337533
```

>CP008866.2 Pseudomonas aeruginosa strain T38079 chromosome, complete genome

```
product length = 202
Forward primer  1        TGCCTGGAACATAATCACCGT  21
Template        3178718  ............G........  3178738

Reverse primer  1        GTCGGTAGATAGCCTGTCGC  20
Template        3178919  ....................  3178900
```

>CP008865.2 Pseudomonas aeruginosa strain S86968 chromosome, complete genome

```
product length = 202
Forward primer  1        TGCCTGGAACATAATCACCGT  21
Template        3303770  ............G........  3303790

Reverse primer  1        GTCGGTAGATAGCCTGTCGC  20
Template        3303971  ....................  3303952
```

>CP008864.2 Pseudomonas aeruginosa strain W60856 chromosome, complete genome

```
product length = 202
Forward primer  1        TGCCTGGAACATAATCACCGT  21
Template        3852598  ............G........  3852618

Reverse primer  1        GTCGGTAGATAGCCTGTCGC  20
Template        3852799  ....................  3852780
```

>CP008862.2 Pseudomonas aeruginosa strain M1608 chromosome, complete genome

```
product length = 202
Forward primer  1        TGCCTGGAACATAATCACCGT  21
Template        3194032  ............G........  3194012

Reverse primer  1        GTCGGTAGATAGCCTGTCGC  20
Template        3193831  ....................  3193850
```

>CP008859.2 Pseudomonas aeruginosa strain H5708 chromosome, complete genome

```
product length = 202
Forward primer  1        TGCCTGGAACATAATCACCGT  21
Template        3118972  ............G........  3118992

Reverse primer  1        GTCGGTAGATAGCCTGTCGC  20
Template        3119173  ....................  3119154
```

>CP008856.2 Pseudomonas aeruginosa strain F23197 chromosome, complete genome

```
product length = 202
Forward primer  1        TGCCTGGAACATAATCACCGT  21
Template        3108665  ............G........  3108685

Reverse primer  1        GTCGGTAGATAGCCTGTCGC  20
Template        3108866  ....................  3108847
```

>CP020603.1 Pseudomonas aeruginosa strain E6130952, complete genome

```
product length = 202
Forward primer  1        TGCCTGGAACATAATCACCGT  21
Template        3315030  ............G........  3315050

Reverse primer  1        GTCGGTAGATAGCCTGTCGC  20
Template        3315231  ....................  3315212
```

>CP016955.1 Pseudomonas aeruginosa strain RIVM-EMC2982, complete genome

```
product length = 202
Forward primer  1        TGCCTGGAACATAATCACCGT  21
Template        3713603  ............G........  3713583

Reverse primer  1        GTCGGTAGATAGCCTGTCGC  20
Template        3713402  ....................  3713421
```

>CP014866.1 Pseudomonas aeruginosa strain PA_154197 chromosome, complete genome

```
product length = 202
Forward primer  1        TGCCTGGAACATAATCACCGT  21
Template        3171709  ............G........  3171729

Reverse primer  1        GTCGGTAGATAGCCTGTCGC  20
Template        3171910  ....................  3171891
```

>LT673656.1 Pseudomonas aeruginosa isolate PcyII-10 genome assembly, chromosome: PcyII-10

```
product length = 202
Forward primer  1        TGCCTGGAACATAATCACCGT  21
Template        3047685  ............G........  3047705

Reverse primer  1        GTCGGTAGATAGCCTGTCGC  20
Template        3047886  ....................  3047867
```

>CP013479.1 Pseudomonas aeruginosa strain NHmuc chromosome, complete genome

```
product length = 202
Forward primer  1        TGCCTGGAACATAATCACCGT  21
Template        2880221  ............G........  2880201

Reverse primer  1        GTCGGTAGATAGCCTGTCGC  20
Template        2880020  ....................  2880039
```

>CP013478.1 Pseudomonas aeruginosa strain SCVJan chromosome, complete genome

```
product length = 202
Forward primer  1        TGCCTGGAACATAATCACCGT  21
Template        3055298  ............G........  3055318

Reverse primer  1        GTCGGTAGATAGCCTGTCGC  20
Template        3055499  ....................  3055480
```

>CP013477.1 Pseudomonas aeruginosa strain SCVFeb chromosome, complete genome

```
product length = 202
Forward primer  1        TGCCTGGAACATAATCACCGT  21
Template        3055298  ............G........  3055318

Reverse primer  1        GTCGGTAGATAGCCTGTCGC  20
Template        3055499  ....................  3055480
```

>CP013113.1 Pseudomonas aeruginosa strain PAER4_119 chromosome, complete genome

```
product length = 202
Forward primer  1        TGCCTGGAACATAATCACCGT  21
Template        3241781  ............G........  3241801

Reverse primer  1        GTCGGTAGATAGCCTGTCGC  20
Template        3241982  ....................  3241963
```

>CP017969.1 Pseudomonas aeruginosa isolate B10W chromosome, complete genome

```
product length = 202
Forward primer  1        TGCCTGGAACATAATCACCGT  21
Template        2986837  ............G........  2986857

Reverse primer  1        GTCGGTAGATAGCCTGTCGC  20
Template        2987038  ....................  2987019
```

>CP014999.1 Pseudomonas aeruginosa strain PA7790, complete genome

```
product length = 202
Forward primer  1        TGCCTGGAACATAATCACCGT  21
Template        3383018  ............G........  3383038

Reverse primer  1        GTCGGTAGATAGCCTGTCGC  20
Template        3383219  ....................  3383200
```

>CP015003.1 Pseudomonas aeruginosa strain PA11803 chromosome, complete genome

```
product length = 202
Forward primer  1        TGCCTGGAACATAATCACCGT  21
Template        3519702  ............G........  3519722

Reverse primer  1        GTCGGTAGATAGCCTGTCGC  20
Template        3519903  ....................  3519884
```

>CP015002.1 Pseudomonas aeruginosa strain PA8281 chromosome, complete genome

```
product length = 202
Forward primer  1        TGCCTGGAACATAATCACCGT  21
Template        3401792  ............G........  3401812

Reverse primer  1        GTCGGTAGATAGCCTGTCGC  20
Template        3401993  ....................  3401974
```

>CP015001.1 Pseudomonas aeruginosa strain PA1088 chromosome, complete genome

```
product length = 202
Forward primer  1        TGCCTGGAACATAATCACCGT  21
Template        3271669  ............G........  3271689

Reverse primer  1        GTCGGTAGATAGCCTGTCGC  20
Template        3271870  ....................  3271851
```

>CP017353.1 Pseudomonas aeruginosa strain FA-HZ1 chromosome, complete genome

```
product length = 202
Forward primer  1        TGCCTGGAACATAATCACCGT  21
Template        6006463  ............G........  6006483

Reverse primer  1        GTCGGTAGATAGCCTGTCGC  20
Template        6006664  ....................  6006645
```

>CP012582.1 Pseudomonas aeruginosa strain PA_D21, complete genome

```
product length = 202
Forward primer  1        TGCCTGGAACATAATCACCGT  21
Template        3186804  ............G........  3186824

Reverse primer  1        GTCGGTAGATAGCCTGTCGC  20
Template        3187005  ....................  3186986
```

>CP012579.1 Pseudomonas aeruginosa strain PA_D5, complete genome

```
product length = 202
Forward primer  1        TGCCTGGAACATAATCACCGT  21
Template        3186804  ............G........  3186824

Reverse primer  1        GTCGGTAGATAGCCTGTCGC  20
Template        3187005  ....................  3186986
```

>CP017099.1 Pseudomonas aeruginosa strain DN1, complete genome

```
product length = 202
Forward primer  1        TGCCTGGAACATAATCACCGT  21
Template        1237515  ............G........  1237495

Reverse primer  1        GTCGGTAGATAGCCTGTCGC  20
Template        1237314  ....................  1237333
```

>CP012584.1 Pseudomonas aeruginosa strain PA_D25, complete genome

```
product length = 202
Forward primer  1        TGCCTGGAACATAATCACCGT  21
Template        3188040  ............G........  3188060

Reverse primer  1        GTCGGTAGATAGCCTGTCGC  20
Template        3188241  ....................  3188222
```

>CP012583.1 Pseudomonas aeruginosa strain PA_D22, complete genome

```
product length = 202
Forward primer  1        TGCCTGGAACATAATCACCGT  21
Template        3186805  ............G........  3186825

Reverse primer  1        GTCGGTAGATAGCCTGTCGC  20
Template        3187006  ....................  3186987
```

>CP012581.1 Pseudomonas aeruginosa strain PA_D16, complete genome

```
product length = 202
Forward primer  1        TGCCTGGAACATAATCACCGT  21
Template        3186805  ............G........  3186825

Reverse primer  1        GTCGGTAGATAGCCTGTCGC  20
Template        3187006  ....................  3186987
```

>CP012580.1 Pseudomonas aeruginosa strain PA_D9, complete genome

```
product length = 202
Forward primer  1        TGCCTGGAACATAATCACCGT  21
Template        3770500  ............G........  3770520

Reverse primer  1        GTCGGTAGATAGCCTGTCGC  20
Template        3770701  ....................  3770682
```

>CP012578.1 Pseudomonas aeruginosa strain PA_D2, complete genome

```
product length = 202
Forward primer  1        TGCCTGGAACATAATCACCGT  21
Template        3186805  ............G........  3186825

Reverse primer  1        GTCGGTAGATAGCCTGTCGC  20
Template        3187006  ....................  3186987
```

>CP012585.1 Pseudomonas aeruginosa strain PA_D1, complete genome

```
product length = 202
Forward primer  1        TGCCTGGAACATAATCACCGT  21
Template        3186805  ............G........  3186825

Reverse primer  1        GTCGGTAGATAGCCTGTCGC  20
Template        3187006  ....................  3186987
```

>CP011857.1 Pseudomonas aeruginosa strain ATCC 27853, complete genome

```
product length = 202
Forward primer  1        TGCCTGGAACATAATCACCGT  21
Template        3207736  ............G........  3207756

Reverse primer  1        GTCGGTAGATAGCCTGTCGC  20
Template        3207937  ....................  3207918
```

>CP015877.1 Pseudomonas aeruginosa SJTD-1 chromosome, complete genome

```
product length = 202
Forward primer  1        TGCCTGGAACATAATCACCGT  21
Template        4208383  ............G........  4208363

Reverse primer  1        GTCGGTAGATAGCCTGTCGC  20
Template        4208182  ....................  4208201
```

>CP015377.1 Pseudomonas aeruginosa strain BAMCPA07-48 chromosome, complete genome

```
product length = 202
Forward primer  1        TGCCTGGAACATAATCACCGT  21
Template        5438508  ............G........  5438528

Reverse primer  1        GTCGGTAGATAGCCTGTCGC  20
Template        5438709  ....................  5438690
```

>CP015117.1 Pseudomonas aeruginosa strain ATCC 27853 chromosome, complete genome

```
product length = 202
Forward primer  1        TGCCTGGAACATAATCACCGT  21
Template        6797456  ............G........  6797476

Reverse primer  1        GTCGGTAGATAGCCTGTCGC  20
Template        6797657  ....................  6797638
```

>OX638610.1 Pseudomonas aeruginosa strain 3541 genome assembly, chromosome: 3541

```
product length = 202
Forward primer  1        TGCCTGGAACATAATCACCGT  21
Template        4201602  ............G........  4201582

Reverse primer  1        GTCGGTAGATAGCCTGTCGC  20
Template        4201401  ....................  4201420
```

>OX638564.1 Pseudomonas aeruginosa strain 3796A genome assembly, chromosome: 3796A

```
product length = 202
Forward primer  1        TGCCTGGAACATAATCACCGT  21
Template        3454205  ............G........  3454225

Reverse primer  1        GTCGGTAGATAGCCTGTCGC  20
Template        3454406  ....................  3454387
```

>CP008873.1 Pseudomonas aeruginosa strain F9670 chromosome, complete genome

```
product length = 202
Forward primer  1        TGCCTGGAACATAATCACCGT  21
Template        5000638  ............G........  5000618

Reverse primer  1        GTCGGTAGATAGCCTGTCGC  20
Template        5000437  ....................  5000456
```

>CP013993.1 Pseudomonas aeruginosa DHS01 chromosome, complete genome

```
product length = 202
Forward primer  1        TGCCTGGAACATAATCACCGT  21
Template        3418335  ............G........  3418355

Reverse primer  1        GTCGGTAGATAGCCTGTCGC  20
Template        3418536  ....................  3418517
```

>CP013989.1 Pseudomonas aeruginosa strain USDA-ARS-USMARC-41639 chromosome, complete genome

```
product length = 202
Forward primer  1        TGCCTGGAACATAATCACCGT  21
Template        3132389  ............G........  3132409

Reverse primer  1        GTCGGTAGATAGCCTGTCGC  20
Template        3132590  ....................  3132571
```

>CP008868.1 Pseudomonas aeruginosa strain T63266 chromosome, complete genome

```
product length = 202
Forward primer  1        TGCCTGGAACATAATCACCGT  21
Template        4776867  ............G........  4776887

Reverse primer  1        GTCGGTAGATAGCCTGTCGC  20
Template        4777068  ....................  4777049
```

>CP008867.1 Pseudomonas aeruginosa strain T52373 chromosome, complete genome

```
product length = 202
Forward primer  1        TGCCTGGAACATAATCACCGT  21
Template        5563467  ............G........  5563487

Reverse primer  1        GTCGGTAGATAGCCTGTCGC  20
Template        5563668  ....................  5563649
```

>CP008863.1 Pseudomonas aeruginosa strain M37351 chromosome, complete genome

```
product length = 202
Forward primer  1       TGCCTGGAACATAATCACCGT  21
Template        119462  ............G........  119482

Reverse primer  1       GTCGGTAGATAGCCTGTCGC  20
Template        119663  ....................  119644
```

>CP008861.1 Pseudomonas aeruginosa strain H47921 chromosome, complete genome

```
product length = 202
Forward primer  1       TGCCTGGAACATAATCACCGT  21
Template        465340  ............G........  465320

Reverse primer  1       GTCGGTAGATAGCCTGTCGC  20
Template        465139  ....................  465158
```

>CP008857.1 Pseudomonas aeruginosa strain F30658 chromosome, complete genome

```
product length = 202
Forward primer  1       TGCCTGGAACATAATCACCGT  21
Template        977006  ............G........  977026

Reverse primer  1       GTCGGTAGATAGCCTGTCGC  20
Template        977207  ....................  977188
```

>CP012901.1 Pseudomonas aeruginosa strain N15-01092 chromosome, complete genome

```
product length = 202
Forward primer  1        TGCCTGGAACATAATCACCGT  21
Template        3557634  ............G........  3557614

Reverse primer  1        GTCGGTAGATAGCCTGTCGC  20
Template        3557433  ....................  3557452
```

>CP124672.1 Pseudomonas aeruginosa strain 2022CK-00451 chromosome, complete genome

```
product length = 202
Forward primer  1        TGCCTGGAACATAATCACCGT  21
Template        5067576  ............G........  5067596

Reverse primer  1        GTCGGTAGATAGCCTGTCGC  20
Template        5067777  ....................  5067758
```

>CP125367.1 Pseudomonas aeruginosa strain ZY1710 chromosome, complete genome

```
product length = 202
Forward primer  1        TGCCTGGAACATAATCACCGT  21
Template        3407331  ............G........  3407351

Reverse primer  1        GTCGGTAGATAGCCTGTCGC  20
Template        3407532  ....................  3407513
```

>CP125365.1 Pseudomonas aeruginosa strain ZY36 chromosome, complete genome

```
product length = 202
Forward primer  1        TGCCTGGAACATAATCACCGT  21
Template        3407583  ............G........  3407603

Reverse primer  1        GTCGGTAGATAGCCTGTCGC  20
Template        3407784  ....................  3407765
```

>CP125363.1 Pseudomonas aeruginosa strain ZY156 chromosome, complete genome

```
product length = 202
Forward primer  1        TGCCTGGAACATAATCACCGT  21
Template        3406806  ............G........  3406826

Reverse primer  1        GTCGGTAGATAGCCTGTCGC  20
Template        3407007  ....................  3406988
```

>CP125361.1 Pseudomonas aeruginosa strain ZY94 chromosome, complete genome

```
product length = 202
Forward primer  1        TGCCTGGAACATAATCACCGT  21
Template        3406800  ............G........  3406820

Reverse primer  1        GTCGGTAGATAGCCTGTCGC  20
Template        3407001  ....................  3406982
```

>CP125288.1 Pseudomonas aeruginosa strain SF416 chromosome, complete genome

```
product length = 202
Forward primer  1        TGCCTGGAACATAATCACCGT  21
Template        3181738  ............G........  3181758

Reverse primer  1        GTCGGTAGATAGCCTGTCGC  20
Template        3181939  ....................  3181920
```

>CP013144.1 Pseudomonas aeruginosa strain Cu1510 chromosome, complete genome

```
product length = 202
Forward primer  1        TGCCTGGAACATAATCACCGT  21
Template        1330604  ............G........  1330584

Reverse primer  1        GTCGGTAGATAGCCTGTCGC  20
Template        1330403  ....................  1330422
```

>AP017302.1 Pseudomonas aeruginosa DNA, complete genome, strain: IOMTU 133

```
product length = 202
Forward primer  1        TGCCTGGAACATAATCACCGT  21
Template        3430753  ............G........  3430773

Reverse primer  1        GTCGGTAGATAGCCTGTCGC  20
Template        3430954  ....................  3430935
```

>CP013245.1 Pseudomonas aeruginosa strain VA-134 chromosome, complete genome

```
product length = 202
Forward primer  1        TGCCTGGAACATAATCACCGT  21
Template        5552275  ............G........  5552255

Reverse primer  1        GTCGGTAGATAGCCTGTCGC  20
Template        5552074  ....................  5552093
```

>LN870292.1 Pseudomonas aeruginosa DK1 genome assembly Pseudomonas aeruginosa DK1 substr. NH57388A, chromosome : I

```
product length = 202
Forward primer  1        TGCCTGGAACATAATCACCGT  21
Template        3056760  ............G........  3056780

Reverse primer  1        GTCGGTAGATAGCCTGTCGC  20
```

```
Template        3056961  ....................  3056942
```

>CP012679.1 Pseudomonas aeruginosa strain PA1RG chromosome, complete genome

```
product length = 202
Forward primer  1        TGCCTGGAACATAATCACCGT  21
Template        3116070  ............G........  3116090

Reverse primer  1        GTCGGTAGATAGCCTGTCGC  20
Template        3116271  ....................  3116252
```

>CP004054.2 Pseudomonas aeruginosa PA1, complete genome

```
product length = 202
Forward primer  1        TGCCTGGAACATAATCACCGT  21
Template        3116070  ............G........  3116090

Reverse primer  1        GTCGGTAGATAGCCTGTCGC  20
Template        3116271  ....................  3116252
```

>AP014839.2 Pseudomonas aeruginosa DNA, complete genome, strain: 8380

```
product length = 202
Forward primer  1        TGCCTGGAACATAATCACCGT  21
Template        3305524  ............G........  3305544

Reverse primer  1        GTCGGTAGATAGCCTGTCGC  20
Template        3305725  ....................  3305706
```

>CP012001.1 Pseudomonas aeruginosa DSM 50071, complete genome

```
product length = 202
Forward primer  1        TGCCTGGAACATAATCACCGT  21
Template        3033408  ............G........  3033428

Reverse primer  1        GTCGGTAGATAGCCTGTCGC  20
Template        3033609  ....................  3033590
```

>CP011369.1 Pseudomonas aeruginosa strain S04 90 chromosome

```
product length = 202
Forward primer  1        TGCCTGGAACATAATCACCGT  21
Template        3386028  ............G........  3386048

Reverse primer  1        GTCGGTAGATAGCCTGTCGC  20
Template        3386229  ....................  3386210
```

>CP011317.1 Pseudomonas aeruginosa strain Carb01 63, complete genome

```
product length = 202
Forward primer  1        TGCCTGGAACATAATCACCGT  21
Template        3705781  ............G........  3705801
```

```
Reverse primer  1        GTCGGTAGATAGCCTGTCGC  20
Template        3705982  ....................  3705963
```

>LN831024.1 Pseudomonas aeruginosa genome assembly NCTC10332, chromosome : 1

```
product length = 202
Forward primer  1        TGCCTGGAACATAATCACCGT  21
Template        3032866  ............G........  3032886

Reverse primer  1        GTCGGTAGATAGCCTGTCGC  20
Template        3033067  ....................  3033048
```

>AP014651.1 Pseudomonas aeruginosa DNA, complete genome, strain: NCGM257

```
product length = 202
Forward primer  1        TGCCTGGAACATAATCACCGT  21
Template        3445473  ............G........  3445493

Reverse primer  1        GTCGGTAGATAGCCTGTCGC  20
Template        3445674  ....................  3445655
```

>CP010555.1 Pseudomonas aeruginosa strain FRD1, complete genome

```
product length = 202
Forward primer  1       TGCCTGGAACATAATCACCGT  21
Template        908164  ............G........  908144

Reverse primer  1       GTCGGTAGATAGCCTGTCGC  20
Template        907963  ....................  907982
```

>AP014646.1 Pseudomonas aeruginosa DNA, complete genome, strain: NCGM 1984

```
product length = 202
Forward primer  1        TGCCTGGAACATAATCACCGT  21
Template        3258509  ............G........  3258529

Reverse primer  1        GTCGGTAGATAGCCTGTCGC  20
Template        3258710  ....................  3258691
```

>HG974234.1 Pseudomonas aeruginosa strain PSE305, genome

```
product length = 202
Forward primer  1        TGCCTGGAACATAATCACCGT  21
Template        2390855  ............G........  2390835

Reverse primer  1        GTCGGTAGATAGCCTGTCGC  20
Template        2390654  ....................  2390673
```

>CP089067.2 Pseudomonas aeruginosa strain UNC_PaerCF19 chromosome, complete genome

```
product length = 202
Forward primer  1        TGCCTGGAACATAATCACCGT  21
Template        4415398  ............G........  4415378
```

```
Reverse primer  1        GTCGGTAGATAGCCTGTCGC  20
Template        4415197  ....................  4415216
```

>CP089065.2 Pseudomonas aeruginosa strain UNC_PaerCF34 chromosome, complete genome

```
product length = 202
Forward primer  1        TGCCTGGAACATAATCACCGT  21
Template        4155594  ............G........  4155574

Reverse primer  1        GTCGGTAGATAGCCTGTCGC  20
Template        4155393  ....................  4155412
```

>CP069337.1 Pseudomonas aeruginosa strain E04 chromosome, complete genome

```
product length = 202
Forward primer  1        TGCCTGGAACATAATCACCGT  21
Template        3179643  ............G........  3179663

Reverse primer  1        GTCGGTAGATAGCCTGTCGC  20
Template        3179844  ....................  3179825
```

>CP068678.1 Pseudomonas aeruginosa strain NCCP15783 chromosome, complete genome

```
product length = 202
Forward primer  1        TGCCTGGAACATAATCACCGT  21
Template        4903297  ............G........  4903277

Reverse primer  1        GTCGGTAGATAGCCTGTCGC  20
Template        4903096  ....................  4903115
```

>CP008739.2 Pseudomonas aeruginosa VRFPA04, complete genome

```
product length = 202
Forward primer  1        TGCCTGGAACATAATCACCGT  21
Template        3635070  ............G........  3635050

Reverse primer  1        GTCGGTAGATAGCCTGTCGC  20
Template        3634869  ....................  3634888
```

>AP014622.1 Pseudomonas aeruginosa DNA, complete genome, strain: NCGM 1900

```
product length = 202
Forward primer  1        TGCCTGGAACATAATCACCGT  21
Template        4543498  ............G........  4543518

Reverse primer  1        GTCGGTAGATAGCCTGTCGC  20
Template        4543699  ....................  4543680
```

>CP008749.1 Pseudomonas aeruginosa PAO1H2O genome

```
product length = 202
Forward primer  1        TGCCTGGAACATAATCACCGT  21
```

```
Template        2453879  ............G........  2453859

Reverse primer  1        GTCGGTAGATAGCCTGTCGC  20
Template        2453678  ....................  2453697
```

>CP007224.1 Pseudomonas aeruginosa PA96 genome

```
product length = 202
Forward primer  1        TGCCTGGAACATAATCACCGT  21
Template        3064226  ............G........  3064246

Reverse primer  1        GTCGGTAGATAGCCTGTCGC  20
Template        3064427  ....................  3064408
```

>CP006985.1 Pseudomonas aeruginosa LESlike4 sequence

```
product length = 202
Forward primer  1        TGCCTGGAACATAATCACCGT  21
Template        3313848  ............G........  3313868

Reverse primer  1        GTCGGTAGATAGCCTGTCGC  20
Template        3314049  ....................  3314030
```

>CP006984.1 Pseudomonas aeruginosa LESlike1 chromosome

```
product length = 202
Forward primer  1        TGCCTGGAACATAATCACCGT  21
Template        3329508  ............G........  3329528

Reverse primer  1        GTCGGTAGATAGCCTGTCGC  20
Template        3329709  ....................  3329690
```

>CP006983.1 Pseudomonas aeruginosa LESB65 sequence

```
product length = 202
Forward primer  1        TGCCTGGAACATAATCACCGT  21
Template        3317372  ............G........  3317392

Reverse primer  1        GTCGGTAGATAGCCTGTCGC  20
Template        3317573  ....................  3317554
```

>CP006982.1 Pseudomonas aeruginosa LES400 sequence

```
product length = 202
Forward primer  1        TGCCTGGAACATAATCACCGT  21
Template        3379426  ............G........  3379446

Reverse primer  1        GTCGGTAGATAGCCTGTCGC  20
Template        3379627  ....................  3379608
```

>CP006981.1 Pseudomonas aeruginosa LESlike7 sequence

```
product length = 202
```

```
Forward primer  1        TGCCTGGAACATAATCACCGT  21
Template        3287230  ............G........  3287250

Reverse primer  1        GTCGGTAGATAGCCTGTCGC  20
Template        3287431  ....................  3287412
```

>CP006980.1 Pseudomonas aeruginosa LESlike5 sequence

```
product length = 202
Forward primer  1        TGCCTGGAACATAATCACCGT  21
Template        3329674  ............G........  3329694

Reverse primer  1        GTCGGTAGATAGCCTGTCGC  20
Template        3329875  ....................  3329856
```

>HG530068.1 Pseudomonas aeruginosa PA38182, complete genome

```
product length = 202
Forward primer  1        TGCCTGGAACATAATCACCGT  21
Template        2523996  ............G........  2523976

Reverse primer  1        GTCGGTAGATAGCCTGTCGC  20
Template        2523795  ....................  2523814
```

>CP006931.1 Pseudomonas aeruginosa SCV20265, complete genome

```
product length = 202
Forward primer  1        TGCCTGGAACATAATCACCGT  21
Template        3297128  ............G........  3297148

Reverse primer  1        GTCGGTAGATAGCCTGTCGC  20
Template        3297329  ....................  3297310
```

>CP006937.1 Pseudomonas aeruginosa LES431, complete genome

```
product length = 202
Forward primer  1        TGCCTGGAACATAATCACCGT  21
Template        3337633  ............G........  3337653

Reverse primer  1        GTCGGTAGATAGCCTGTCGC  20
Template        3337834  ....................  3337815
```

>CP006853.1 Pseudomonas aeruginosa MTB-1, complete genome

```
product length = 202
Forward primer  1        TGCCTGGAACATAATCACCGT  21
Template        3066660  ............G........  3066680

Reverse primer  1        GTCGGTAGATAGCCTGTCGC  20
Template        3066861  ....................  3066842
```

>CP004055.1 Pseudomonas aeruginosa PA1R, complete genome

```
product length = 202
Forward primer  1      TGCCTGGAACATAATCACCGT  21
Template        39524  ............G........  39504

Reverse primer  1      GTCGGTAGATAGCCTGTCGC  20
Template        39323  ....................  39342
```

>CP081345.1 Pseudomonas aeruginosa strain F291007 chromosome, complete genome

```
product length = 202
Forward primer  1        TGCCTGGAACATAATCACCGT  21
Template        3700491  ............G........  3700471

Reverse primer  1        GTCGGTAGATAGCCTGTCGC  20
Template        3700290  ....................  3700309
```

>CP081346.1 Pseudomonas aeruginosa strain SE5419 chromosome, complete genome

```
product length = 202
Forward primer  1        TGCCTGGAACATAATCACCGT  21
Template        3396990  ............G........  3396970

Reverse primer  1        GTCGGTAGATAGCCTGTCGC  20
Template        3396789  ....................  3396808
```

>CP081287.1 Pseudomonas aeruginosa strain F092021 chromosome, complete genome

```
product length = 202
Forward primer  1        TGCCTGGAACATAATCACCGT  21
Template        3254274  ............G........  3254294

Reverse primer  1        GTCGGTAGATAGCCTGTCGC  20
Template        3254475  ....................  3254456
```

>CP081202.1 Pseudomonas aeruginosa strain P9W chromosome, complete genome

```
product length = 202
Forward primer  1        TGCCTGGAACATAATCACCGT  21
Template        2010370  ............G........  2010350

Reverse primer  1        GTCGGTAGATAGCCTGTCGC  20
Template        2010169  ....................  2010188
```

>CP006728.1 Pseudomonas aeruginosa c7447m genome

```
product length = 202
Forward primer  1        TGCCTGGAACATAATCACCGT  21
Template        2453080  ............G........  2453060

Reverse primer  1        GTCGGTAGATAGCCTGTCGC  20
Template        2452879  ....................  2452898
```

>CP006245.1 Pseudomonas aeruginosa RP73, complete genome

```
product length = 202
Forward primer  1        TGCCTGGAACATAATCACCGT  21
Template        2498505  ............G........  2498485

Reverse primer  1        GTCGGTAGATAGCCTGTCGC  20
Template        2498304  ....................  2498323
```

>CP074424.1 Pseudomonas aeruginosa strain 88A chromosome

```
product length = 202
Forward primer  1       TGCCTGGAACATAATCACCGT  21
Template        717681  ............G........  717701

Reverse primer  1       GTCGGTAGATAGCCTGTCGC  20
Template        717882  ....................  717863
```

>CP061780.1 Pseudomonas aeruginosa strain ZBX-P11 chromosome, complete genome

```
product length = 202
Forward primer  1        TGCCTGGAACATAATCACCGT  21
Template        5891225  ............G........  5891245

Reverse primer  1        GTCGGTAGATAGCCTGTCGC  20
Template        5891426  ....................  5891407
```

>CP061778.1 Pseudomonas aeruginosa strain ZBX-P13 chromosome, complete genome

```
product length = 202
Forward primer  1        TGCCTGGAACATAATCACCGT  21
Template        4942203  ............G........  4942223

Reverse primer  1        GTCGGTAGATAGCCTGTCGC  20
Template        4942404  ....................  4942385
```

>CP004061.1 Pseudomonas aeruginosa B136-33, complete genome

```
product length = 202
Forward primer  1        TGCCTGGAACATAATCACCGT  21
Template        3060288  ............G........  3060308

Reverse primer  1        GTCGGTAGATAGCCTGTCGC  20
Template        3060489  ....................  3060470
```

>CP070471.1 Pseudomonas aeruginosa strain B17932 chromosome, complete genome

```
product length = 202
Forward primer  1        TGCCTGGAACATAATCACCGT  21
Template        3681021  ............G........  3681001

Reverse primer  1        GTCGGTAGATAGCCTGTCGC  20
Template        3680820  ....................  3680839
```

>CP070467.1 Pseudomonas aeruginosa strain B17416 chromosome, complete genome

```
product length = 202
Forward primer  1        TGCCTGGAACATAATCACCGT  21
Template        3919661  ............G........  3919641

Reverse primer  1        GTCGGTAGATAGCCTGTCGC  20
Template        3919460  ....................  3919479
```

>CP003149.1 Pseudomonas aeruginosa DK2, complete genome

```
product length = 202
Forward primer  1        TGCCTGGAACATAATCACCGT  21
Template        3054619  ............G........  3054639

Reverse primer  1        GTCGGTAGATAGCCTGTCGC  20
Template        3054820  ....................  3054801
```

>CP063237.1 Pseudomonas aeruginosa strain mPA08-31 chromosome

```
product length = 202
Forward primer  1        TGCCTGGAACATAATCACCGT  21
Template        2370032  ............G........  2370012

Reverse primer  1        GTCGGTAGATAGCCTGTCGC  20
Template        2369831  ....................  2369850
```

>CP063047.1 Pseudomonas aeruginosa strain KC-Tt-1 chromosome, complete genome

```
product length = 202
Forward primer  1        TGCCTGGAACATAATCACCGT  21
Template        2286204  ............G........  2286224

Reverse primer  1        GTCGGTAGATAGCCTGTCGC  20
Template        2286405  ....................  2286386
```

>CP062219.1 Pseudomonas aeruginosa strain JT86 chromosome, complete genome

```
product length = 202
Forward primer  1        TGCCTGGAACATAATCACCGT  21
Template        1411118  ............G........  1411138

Reverse primer  1        GTCGGTAGATAGCCTGTCGC  20
Template        1411319  ....................  1411300
```

>CP061850.1 Pseudomonas aeruginosa strain R31 chromosome, complete genome

```
product length = 202
Forward primer  1        TGCCTGGAACATAATCACCGT  21
Template        2577971  ............G........  2577991

Reverse primer  1        GTCGGTAGATAGCCTGTCGC  20
Template        2578172  ....................  2578153
```

>CP059852.1 Pseudomonas aeruginosa strain ZM03 chromosome, complete genome

```
product length = 202
Forward primer  1     TGCCTGGAACATAATCACCGT  21
Template        9195  ............G........  9215

Reverse primer  1     GTCGGTAGATAGCCTGTCGC  20
Template        9396  ....................  9377
```

>AP012280.1 Pseudomonas aeruginosa NCGM2.S1 DNA, complete genome

```
product length = 202
Forward primer  1        TGCCTGGAACATAATCACCGT  21
Template        3463416  ............G........  3463396

Reverse primer  1        GTCGGTAGATAGCCTGTCGC  20
Template        3463215  ....................  3463234
```

>FM209186.1 Pseudomonas aeruginosa LESB58 complete genome sequence

```
product length = 202
Forward primer  1        TGCCTGGAACATAATCACCGT  21
Template        3390410  ............G........  3390430

Reverse primer  1        GTCGGTAGATAGCCTGTCGC  20
Template        3390611  ....................  3390592
```

>CP109657.1 Pseudomonas aeruginosa strain Zw26 chromosome, complete genome

```
product length = 202
Forward primer  1        TGCCTGGAACATAATCACCGT  21
Template        3009691  G...........G........  3009711

Reverse primer  1        GTCGGTAGATAGCCTGTCGC  20
Template        3009892  ....................  3009873
```

>CP068238.1 Pseudomonas aeruginosa strain A39-1 chromosome, complete genome

```
product length = 202
Forward primer  1        TGCCTGGAACATAATCACCGT  21
Template        3052023  G...........G........  3052043

Reverse primer  1        GTCGGTAGATAGCCTGTCGC  20
Template        3052224  ....................  3052205
```

>CP041354.1 Pseudomonas aeruginosa strain AZPAE15042 chromosome, complete genome

```
product length = 202
Forward primer  1        TGCCTGGAACATAATCACCGT  21
Template        3087928  G...........G........  3087948

Reverse primer  1        GTCGGTAGATAGCCTGTCGC  20
Template        3088129  ....................  3088110
```

>CP029093.1 Pseudomonas paraeruginosa strain AR441 chromosome, complete genome

```
product length = 202
Forward primer  1        TGCCTGGAACATAATCACCGT  21
Template        3074887  G...........G........  3074907

Reverse primer  1        GTCGGTAGATAGCCTGTCGC  20
Template        3075088  ....................  3075069
```

>CP020560.1 Pseudomonas paraeruginosa strain Cr1 chromosome, complete genome

```
product length = 202
Forward primer  1        TGCCTGGAACATAATCACCGT  21
Template        2911918  G...........G........  2911938

Reverse primer  1        GTCGGTAGATAGCCTGTCGC  20
Template        2912119  ....................  2912100
```

>CP027169.1 Pseudomonas paraeruginosa strain AR_0356 chromosome, complete genome

```
product length = 202
Forward primer  1        TGCCTGGAACATAATCACCGT  21
Template        4335258  G...........G........  4335278

Reverse primer  1        GTCGGTAGATAGCCTGTCGC  20
Template        4335459  ....................  4335440
```

>CP000744.1 Pseudomonas aeruginosa PA7, complete genome

```
product length = 202
Forward primer  1        TGCCTGGAACATAATCACCGT  21
Template        3104079  G...........G........  3104099

Reverse primer  1        GTCGGTAGATAGCCTGTCGC  20
Template        3104280  ....................  3104261
```

>CP075821.1 Pseudomonas aeruginosa strain PaLo35 chromosome, complete genome

```
product length = 202
Forward primer  1        TGCCTGGAACATAATCACCGT  21
Template        3240146  ............G........  3240126

Reverse primer  1        GTCGGTAGATAGCCTGTCGC  20
Template        3239945  .........A..........  3239964
```

>CP075817.1 Pseudomonas aeruginosa strain PaLo39 chromosome, complete genome

```
product length = 202
Forward primer  1        TGCCTGGAACATAATCACCGT  21
Template        3694385  ............G........  3694365

Reverse primer  1        GTCGGTAGATAGCCTGTCGC  20
Template        3694184  .........A..........  3694203
```

>CP075815.1 Pseudomonas aeruginosa strain PaLo43 chromosome, complete genome

```
product length = 202
Forward primer  1        TGCCTGGAACATAATCACCGT  21
Template        2918941  ............G........  2918921

Reverse primer  1        GTCGGTAGATAGCCTGTCGC  20
Template        2918740  .........A..........  2918759
```

>CP075814.1 Pseudomonas aeruginosa strain PaLo44 chromosome, complete genome

```
product length = 202
Forward primer  1        TGCCTGGAACATAATCACCGT  21
Template        3062461  ............G........  3062481

Reverse primer  1        GTCGGTAGATAGCCTGTCGC  20
Template        3062662  .........A..........  3062643
```

>CP075784.1 Pseudomonas aeruginosa strain PaLo507 chromosome, complete genome

```
product length = 202
Forward primer  1        TGCCTGGAACATAATCACCGT  21
Template        3097047  ............G........  3097067

Reverse primer  1        GTCGGTAGATAGCCTGTCGC  20
Template        3097248  .........A..........  3097229
```

>CP075764.1 Pseudomonas aeruginosa strain PaLo541 chromosome, complete genome

```
product length = 202
Forward primer  1        TGCCTGGAACATAATCACCGT  21
Template        3084643  ............G........  3084663

Reverse primer  1        GTCGGTAGATAGCCTGTCGC  20
Template        3084844  .........A..........  3084825
```

>CP075757.1 Pseudomonas aeruginosa strain PaLo552 chromosome, complete genome

```
product length = 202
Forward primer  1        TGCCTGGAACATAATCACCGT  21
Template        3254819  ............G........  3254839

Reverse primer  1        GTCGGTAGATAGCCTGTCGC  20
Template        3255020  .........A..........  3255001
```

>CP096822.1 Pseudomonas aeruginosa strain NY8709 chromosome, complete genome

```
product length = 202
Forward primer  1        TGCCTGGAACATAATCACCGT  21
Template        3285495  ............G........  3285515

Reverse primer  1        GTCGGTAGATAGCCTGTCGC  20
Template        3285696  .........A..........  3285677
```

>CP109919.1 Pseudomonas aeruginosa strain PALA56 chromosome, complete genome

```
product length = 202
Forward primer  1        TGCCTGGAACATAATCACCGT  21
Template        3107800  ............G........  3107820

Reverse primer  1        GTCGGTAGATAGCCTGTCGC  20
Template        3108001  .........A..........  3107982
```

>CP109856.1 Pseudomonas aeruginosa strain PALA53 chromosome, complete genome

```
product length = 202
Forward primer  1        TGCCTGGAACATAATCACCGT  21
Template        3080611  ............G........  3080631

Reverse primer  1        GTCGGTAGATAGCCTGTCGC  20
Template        3080812  .........A..........  3080793
```

>CP109844.1 Pseudomonas aeruginosa strain PALA32 chromosome, complete genome

```
product length = 202
Forward primer  1        TGCCTGGAACATAATCACCGT  21
Template        3171871  ............G........  3171891

Reverse primer  1        GTCGGTAGATAGCCTGTCGC  20
Template        3172072  .........A..........  3172053
```

>CP109834.1 Pseudomonas aeruginosa strain PALA25 chromosome, complete genome

```
product length = 202
Forward primer  1        TGCCTGGAACATAATCACCGT  21
Template        3077881  ............G........  3077901

Reverse primer  1        GTCGGTAGATAGCCTGTCGC  20
Template        3078082  .........A..........  3078063
```

>CP106743.1 Pseudomonas aeruginosa strain PALA15 chromosome, complete genome

```
product length = 202
Forward primer  1        TGCCTGGAACATAATCACCGT  21
Template        3135739  ............G........  3135759

Reverse primer  1        GTCGGTAGATAGCCTGTCGC  20
Template        3135940  .........A..........  3135921
```

>CP083360.1 Pseudomonas aeruginosa strain KPA83 chromosome, complete genome

```
product length = 202
Forward primer  1        TGCCTGGAACATAATCACCGT  21
Template        2295281  ............G........  2295261

Reverse primer  1        GTCGGTAGATAGCCTGTCGC  20
Template        2295080  .........A..........  2295099
```

>CP104586.1 Pseudomonas aeruginosa strain WTJH6 chromosome, complete genome

```
product length = 202
Forward primer  1        TGCCTGGAACATAATCACCGT  21
Template        6651536  ............G........  6651516

Reverse primer  1        GTCGGTAGATAGCCTGTCGC  20
Template        6651335  .........A..........  6651354
```

>CP094677.1 Pseudomonas aeruginosa strain Pa150 chromosome, complete genome

```
product length = 202
Forward primer  1        TGCCTGGAACATAATCACCGT  21
Template        6459169  ............G........  6459149

Reverse primer  1        GTCGGTAGATAGCCTGTCGC  20
Template        6458968  .........A..........  6458987
```

>CP097256.1 Pseudomonas aeruginosa strain D5 chromosome, complete genome

```
product length = 202
Forward primer  1        TGCCTGGAACATAATCACCGT  21
Template        3081426  ............G........  3081446

Reverse primer  1        GTCGGTAGATAGCCTGTCGC  20
Template        3081627  .........A..........  3081608
```

>CP063396.1 Pseudomonas aeruginosa strain ST167_d26burn chromosome, complete genome

```
product length = 202
Forward primer  1        TGCCTGGAACATAATCACCGT  21
Template        3257032  ............G........  3257052

Reverse primer  1        GTCGGTAGATAGCCTGTCGC  20
Template        3257233  .........A..........  3257214
```

>CP063395.1 Pseudomonas aeruginosa strain ST167_d57blood chromosome, complete genome

```
product length = 202
Forward primer  1        TGCCTGGAACATAATCACCGT  21
Template        3257032  ............G........  3257052

Reverse primer  1        GTCGGTAGATAGCCTGTCGC  20
Template        3257233  .........A..........  3257214
```

>CP063394.1 Pseudomonas aeruginosa strain ST167_d67burn1 chromosome, complete genome

```
product length = 202
Forward primer  1        TGCCTGGAACATAATCACCGT  21
Template        3257509  ............G........  3257529

Reverse primer  1        GTCGGTAGATAGCCTGTCGC  20
Template        3257710  .........A..........  3257691
```

>CP063393.1 Pseudomonas aeruginosa strain ST167_d67burn2 chromosome, complete genome

```
product length = 202
Forward primer  1        TGCCTGGAACATAATCACCGT  21
Template        3257020  ............G........  3257040

Reverse primer  1        GTCGGTAGATAGCCTGTCGC  20
Template        3257221  .........A..........  3257202
```

>CP063392.1 Pseudomonas aeruginosa strain ST167_d68blood1 chromosome, complete genome

```
product length = 202
Forward primer  1        TGCCTGGAACATAATCACCGT  21
Template        3257037  ............G........  3257057

Reverse primer  1        GTCGGTAGATAGCCTGTCGC  20
Template        3257238  .........A..........  3257219
```

>CP063391.1 Pseudomonas aeruginosa strain ST167_d68blood2 chromosome, complete genome

```
product length = 202
Forward primer  1        TGCCTGGAACATAATCACCGT  21
Template        3257033  ............G........  3257053

Reverse primer  1        GTCGGTAGATAGCCTGTCGC  20
Template        3257234  .........A..........  3257215
```

>CP089068.2 Pseudomonas aeruginosa strain UNC_PaerCF13 chromosome, complete genome

```
product length = 202
Forward primer  1        TGCCTGGAACATAATCACCGT  21
Template        4604247  ............G........  4604267

Reverse primer  1        GTCGGTAGATAGCCTGTCGC  20
Template        4604448  .........A..........  4604429
```

>CP089745.1 Pseudomonas aeruginosa strain Pa608 chromosome, complete genome

```
product length = 202
Forward primer  1        TGCCTGGAACATAATCACCGT  21
Template        3099250  ............G........  3099270

Reverse primer  1        GTCGGTAGATAGCCTGTCGC  20
Template        3099451  .........A..........  3099432
```

>CP064403.1 Pseudomonas aeruginosa strain WTJH12 chromosome, complete genome

```
product length = 202
Forward primer  1        TGCCTGGAACATAATCACCGT  21
Template        3106266  ............G........  3106286

Reverse primer  1        GTCGGTAGATAGCCTGTCGC  20
Template        3106467  .........A..........  3106448
```

>CP071947.1 Pseudomonas aeruginosa strain 2020HL-00861 chromosome, complete genome

```
product length = 202
Forward primer  1        TGCCTGGAACATAATCACCGT  21
Template        4620013  ............G........  4620033

Reverse primer  1        GTCGGTAGATAGCCTGTCGC  20
Template        4620214  .........A..........  4620195
```

>CP070355.1 Pseudomonas aeruginosa strain PDNC003 chromosome

```
product length = 202
Forward primer  1        TGCCTGGAACATAATCACCGT  21
Template        1850728  ............G........  1850748

Reverse primer  1        GTCGGTAGATAGCCTGTCGC  20
Template        1850929  .........A..........  1850910
```

>CP050335.1 Pseudomonas aeruginosa strain DVT401 chromosome, complete genome

```
product length = 202
Forward primer  1        TGCCTGGAACATAATCACCGT  21
Template        3069610  ............G........  3069630

Reverse primer  1        GTCGGTAGATAGCCTGTCGC  20
Template        3069811  .........A..........  3069792
```

>CP041773.1 Pseudomonas aeruginosa strain 519119 chromosome, complete genome

```
product length = 202
Forward primer  1        TGCCTGGAACATAATCACCGT  21
Template        5786033  ............G........  5786053

Reverse primer  1        GTCGGTAGATAGCCTGTCGC  20
Template        5786234  .........A..........  5786215
```

>LR134300.1 Pseudomonas fluorescens strain NCTC10783 genome assembly, chromosome: 1

```
product length = 202
Forward primer  1        TGCCTGGAACATAATCACCGT  21
Template        2134312  ............G........  2134332

Reverse primer  1        GTCGGTAGATAGCCTGTCGC  20
Template        2134513  .........A..........  2134494
```

>CP017306.1 Pseudomonas aeruginosa strain PA_150577 chromosome, complete genome

```
product length = 202
Forward primer  1        TGCCTGGAACATAATCACCGT  21
Template        3144504  ............G........  3144524

Reverse primer  1        GTCGGTAGATAGCCTGTCGC  20
Template        3144705  .........A..........  3144686
```

>CP008860.2 Pseudomonas aeruginosa strain H27930 chromosome, complete genome

```
product length = 202
Forward primer  1        TGCCTGGAACATAATCACCGT  21
Template        3130457  ............G........  3130477

Reverse primer  1        GTCGGTAGATAGCCTGTCGC  20
Template        3130658  .........A..........  3130639
```

>CP016214.1 Pseudomonas aeruginosa strain PA121617, complete genome

```
product length = 202
Forward primer  1        TGCCTGGAACATAATCACCGT  21
Template        6374325  ............G........  6374345

Reverse primer  1        GTCGGTAGATAGCCTGTCGC  20
Template        6374526  .........A..........  6374507
```

>CP013696.1 Pseudomonas aeruginosa strain 12-4-4(59) chromosome, complete genome

```
product length = 202
Forward primer  1        TGCCTGGAACATAATCACCGT  21
Template        4717647  ............G........  4717667

Reverse primer  1        GTCGGTAGATAGCCTGTCGC  20
Template        4717848  .........A..........  4717829
```

>CP012066.1 Pseudomonas aeruginosa strain F9676, complete genome

```
product length = 202
Forward primer  1        TGCCTGGAACATAATCACCGT  21
Template        2815965  ............G........  2815945

Reverse primer  1        GTCGGTAGATAGCCTGTCGC  20
Template        2815764  .........A..........  2815783
```

>CP007399.1 Pseudomonas aeruginosa strain F22031, complete genome

```
product length = 202
Forward primer  1        TGCCTGGAACATAATCACCGT  21
Template        5726192  ............G........  5726212

Reverse primer  1        GTCGGTAGATAGCCTGTCGC  20
Template        5726393  .........A..........  5726374
```

>CP002496.1 Pseudomonas aeruginosa M18, complete genome

```
product length = 202
Forward primer  1        TGCCTGGAACATAATCACCGT  21
Template        3137321  ............G........  3137341

Reverse primer  1        GTCGGTAGATAGCCTGTCGC  20
Template        3137522  .........A..........  3137503
```

>CP093012.1 Pseudomonas aeruginosa strain H20 chromosome, complete genome

```
product length = 202
Forward primer  1         TGCCTGGAACATAATCACCGT  21
Template        3099967   C...........G........  3099987

Reverse primer  1         GTCGGTAGATAGCCTGTCGC  20
Template        3100168   .........A..........  3100149
```

If you want to allow any of the unintended targets, check the box(es) next to the ones you accept and try again to re-search for specific primers Submit

Help

FOLLOW NCBI

Connect with NLM

National Library of Medicine
8600 Rockville Pike
Bethesda, MD 20894

Web Policies
FOIA
HHS Vulnerability Disclosure

Help
Accessibility
Careers

NLM NIH HHS USA.gov
