# Supplementary figures and images for: Phylogenetic Aspects of Antibiotic Resistance and Biofilm Formation of P. aeruginosa Isolated from Clinical Samples
Source: Can J Infect Dis Med Microbiol. 2024 Jan 13;2024:6213873. doi: 10.1155/2024/6213873 (PMC10799695; doi:10.1155/2024/6213873)

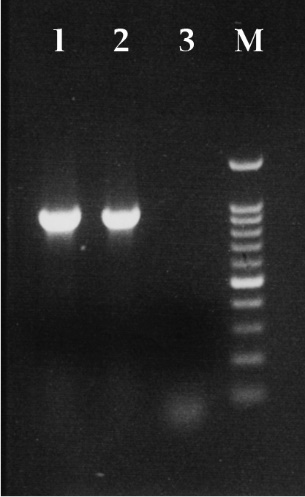

Supplement: Supplementary Materials — Original pictures and primer-blast results. [file 6213873.f1.zip › 1.docx]

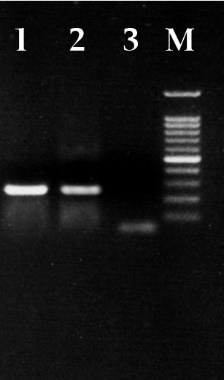

Supplement: Supplementary Materials — Original pictures and primer-blast results. [file 6213873.f1.zip › brlR.docx]

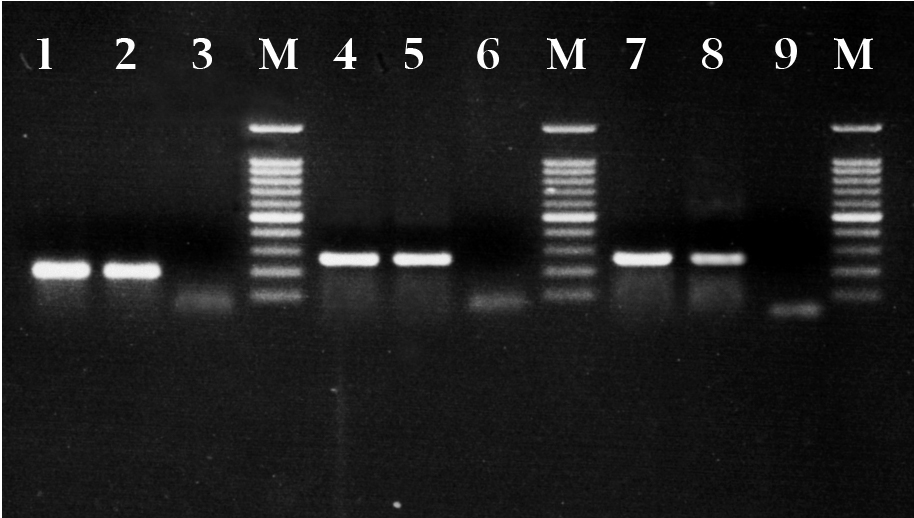

Supplement: Supplementary Materials — Original pictures and primer-blast results. [file 6213873.f1.zip › file 2.docx]

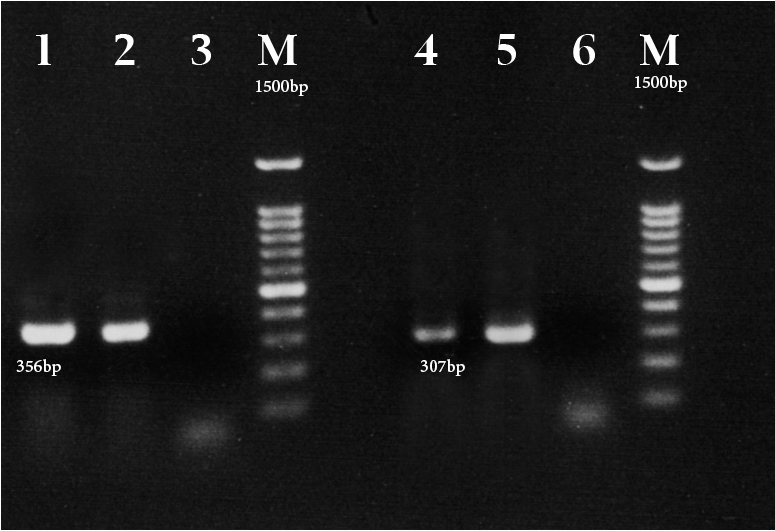

Supplement: Supplementary Materials — Original pictures and primer-blast results. [file 6213873.f1.zip › main.docx]

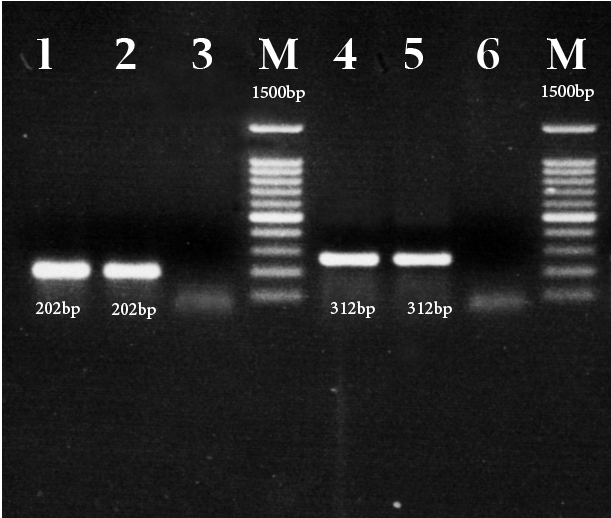

Supplement: Supplementary Materials — Original pictures and primer-blast results. [file 6213873.f1.zip › main.tif1.docx]

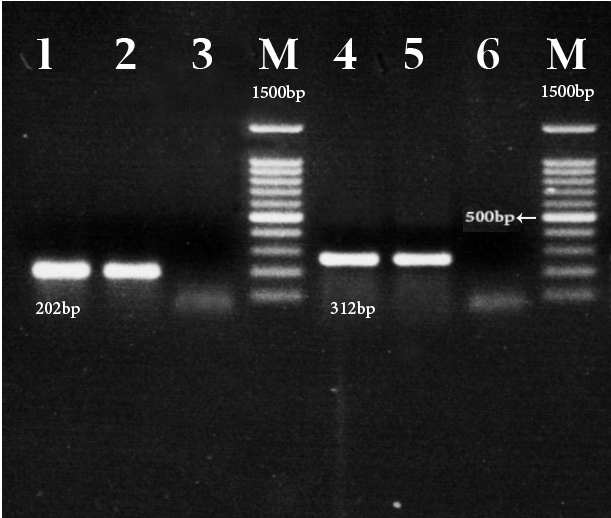

Supplement: Supplementary Materials — Original pictures and primer-blast results. [file 6213873.f1.zip › main.tif3.docx]

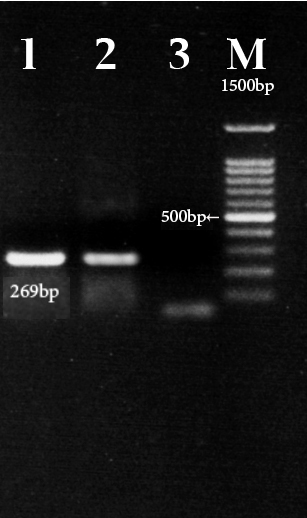

Supplement: Supplementary Materials — Original pictures and primer-blast results. [file 6213873.f1.zip › main5.docx]

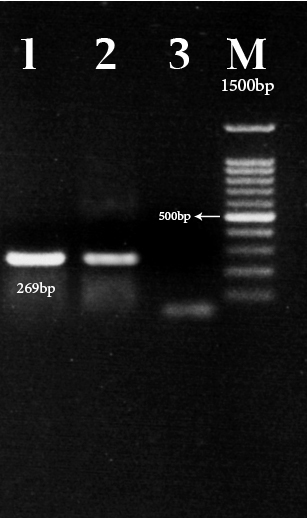

Supplement: Supplementary Materials — Original pictures and primer-blast results. [file 6213873.f1.zip › main6.docx]

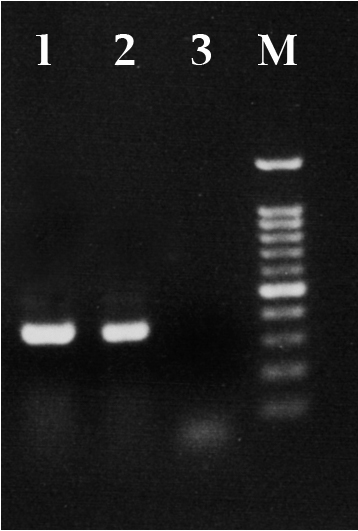

Supplement: Supplementary Materials — Original pictures and primer-blast results. [file 6213873.f1.zip › mex.A.docx]

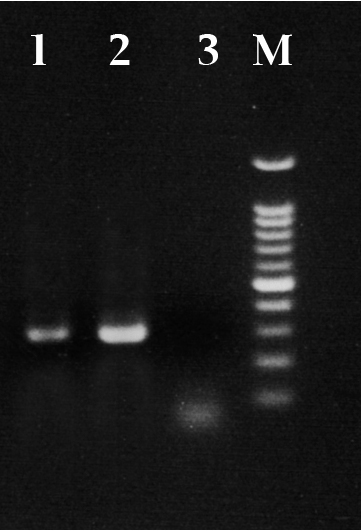

Supplement: Supplementary Materials — Original pictures and primer-blast results. [file 6213873.f1.zip › mex.B.docx]

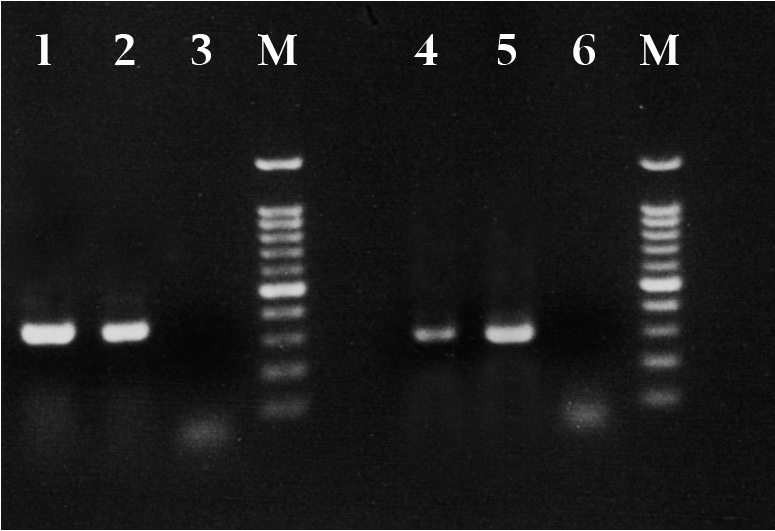

Supplement: Supplementary Materials — Original pictures and primer-blast results. [file 6213873.f1.zip › mex.docx]

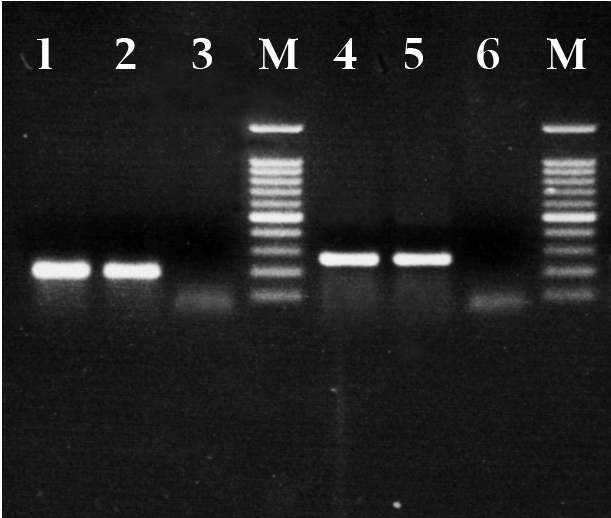

Supplement: Supplementary Materials — Original pictures and primer-blast results. [file 6213873.f1.zip › psl & pel.docx]
